# A New Genome-to-Genome Comparison Approach for Large-Scale Revisiting of Current Microbial Taxonomy

**DOI:** 10.3390/microorganisms7060161

**Published:** 2019-06-03

**Authors:** Ming-Hsin Tsai, Yen-Yi Liu, Von-Wun Soo, Chih-Chieh Chen

**Affiliations:** 1 Institute of Population Health Sciences, National Health Research Institutes, Miaoli County 35053, Taiwan; skypea@nhri.org.tw; 2 Central Regional Laboratory, Center for Diagnostics and Vaccine Development, Centers for Disease Control, Taichung 40855, Taiwan; current788@gmail.com; 3 Department of Computer Science, National Tsin Hua University, Hsinchu 30013, Taiwan; 4 Institute of Medical Science and Technology, National Sun Yat-sen University, Kaohsiung, 80424, Taiwan; 5 Rapid Screening Research Center for Toxicology and Biomedicine, National Sun Yat-sen University, Kaohsiung 80424, Taiwan

**Keywords:** microbial taxonomy, whole genome comparison, bacterial classification, bacterial identification

## Abstract

Microbial diversity has always presented taxonomic challenges. With the popularity of next-generation sequencing technology, more unculturable bacteria have been sequenced, facilitating the discovery of additional new species and complicated current microbial classification. The major challenge is to assign appropriate taxonomic names. Hence, assessing the consistency between taxonomy and genomic relatedness is critical. We proposed and applied a genome comparison approach to a large-scale survey to investigate the distribution of genomic differences among microorganisms. The approach applies a genome-wide criterion, homologous coverage ratio (HCR), for describing the homology between species. The survey included 7861 microbial genomes that excluded plasmids, and 1220 pairs of genera exhibited ambiguous classification. In this study, we also compared the performance of HCR and average nucleotide identity (ANI). The results indicated that HCR and ANI analyses yield comparable results, but a few examples suggested that HCR has a superior clustering effect. In addition, we used the Genome Taxonomy Database (GTDB), the gold standard for taxonomy, to validate our analysis. The GTDB offers 120 ubiquitous single-copy proteins as marker genes for species classification. We determined that the analysis of the GTDB still results in classification boundary blur between some genera and that the marker gene-based approach has limitations. Although the choice of marker genes has been quite rigorous, the bias of marker gene selection remains unavoidable. Therefore, methods based on genomic alignment should be considered for use for species classification in order to avoid the bias of marker gene selection. On the basis of our observations of microbial diversity, microbial classification should be re-examined using genome-wide comparisons.

## 1. Introduction

In the higher-level classification of organisms, species taxonomy is consistent with phylogenetic inferences—species with similar genotypes should have similar phenotypes. Such a philosophy of species classification is constantly being applied to microorganisms. Defining microbial species is challenged by microbial diversity. Microbial diversity is attributable to the need of microorganisms to rapidly mutate to adapt to the environment under different environmental conditions [1]. Horizontal gene transfer is also a factor that affects microbial diversity, and as long as it is beneficial to survival, a transferred gene is retained [2].

In sequence-based classification methods, the analysis of marker gene sequences is a key step in microbial taxonomy. Because selecting different marker genes can result in different classification results, determining how to select an appropriate marker gene is a challenge. Therefore, studies have employed rigorous conditions to screen marker genes in order to avoid bias caused by the selection of different combinations of marker genes [3]. In addition, marker genes can be used to discover new species; for example, ribosomal protein-related gene sets in metagenomic sequences were analyzed to discover new species [4]. The 16S rRNA sequence analysis is also commonly used for microbial classification and species identification because 16S rRNA is an essential microbial gene with a highly conserved region and essential microbial genes are convenient target genes to be used for inferring phylogenetic distances and are applied in microbial classification and species identification. Individual species have multiple 16S rRNAs [5], and the diversity in 16S rRNAs varies from species to species. However, the use of 16S rRNA similarity as a species identification method requires caution [6].

Whole-genome alignment is another method of microbial classification, and the terminology of the overall genome association index (OGRI) was proposed in 2014 [7]. The OGRI is used to identify the degree of similarity between any two genomic sequences. Average nucleotide identity (ANI) is the most widely used method for calculating OGRI values. ANI is conceptually a digital version of the DNA–DNA hybridization (DDH) method and has also been shown to have a strong linear relationship with DDH, which has been considered the gold standard for microbial classification over the past few decades, although this method is very labor intensive [8]. Thus, many other tools for calculating the OGRI degree of similarity have been developed to replace the DDH method [9]. In practice, ANI still consumes too many computing resources and thus cannot be used to perform large-scale genomic surveys. Therefore, we propose using a genome-wide criterion, homologous coverage ratio (HCR), to describe the homology between species. In the proposed method, distances in homologous relationships are evaluated on the basis of the results of whole-genome comparisons, and this eliminates the bias that can be caused by the presence of multiple alignments and the selection of different marker gene combinations. In this study, we developed rapid algorithms to perform a large-scale genomic comparison and provide a distribution profile for most known microorganisms. Then, we used the sequence alignment tool to perform a more precise genomic comparison and ascertain the evidence of ambiguous microbial classification.

## 2. Materials and Methods

### 2.1. Data Description

Data used in this study were mainly divided into two parts. The first part consisted of 7861 microbial genomes, which were downloaded from the National Center for Biotechnology Information and used as a reference data set to assess species homology. This data set included 2499 species of bacteria belonging to 940 genera, 209 Archaea belonging to 91 genera, and 18 strains belonging to 17 genera (Supplemental Table S1). It should be noted that all plasmids were excluded from our analysis. The other part involved data from the Genome Taxonomy Database (GTDB, refer to the website http://gtdb.ecogenomic.org/), which provides 120 ubiquitous single-copy proteins as marker genes for microbial classification, simply as BAC120 [3]. Because of the diversity of species, the complexity of species classification has increased, and the emergence of the BAC120 database provides a gold standard for microbial classification. For assessing similar genera, BAC120 provides a good standard to use for verifying the correctness of species cluster analysis.

### 2.2. Homologous Coverage Ratio (HCR)

In this study, we used the HCR between genomes to describe evolutionary relationships between species and to revisit existing microbial classifications. The HCR is relevant in two scenarios. The first scenario involves a change in the size of a microbial genome, as presented in Case 1 in Supplemental Figure S1. Although the length of the homologous sequences of A1 and B1 are shorter than that of the homologous sequences of A2 and B2, the HCRs of A1 and B1 are still greater than those of A2 and B2, indicating that similarities between genomes A1 and B1 are greater than those between genomes A2 and B2. The second scenario involves sequence repeats that occur in the genome. If repeats are homologous sequences, then the homologous coverage also increases, as depicted in Case 2 of Supplemental Figure S1. The HCR is used to describe the homologous relationship between genomes. A higher HCR indicates a smaller difference between two species. If genomes A and B are exactly the same, the HCR would be equal to 1.

LAST [10], which is one of the most-cited gene comparison tools, was chosen for use in this study. LAST can execute a genome alignment of approximately 40,000 bases within 20 seconds. Although LAST is a very rapid tool, it is still not adequate for performing large-scale sequence comparisons. Because 7861 microbial whole genomes were used in the present study, approximately 20 seconds would be required to achieve the alignment of each pair of genomes. The time required to complete all alignment tasks was approximately 7153 days [(7861 + 1) × 7861/2 × 20/3600 ≈ 171,675 hours ≈ 7153 days]. Therefore, we used the k-mer-based method to approximate the HCR as follows (simply as HCRkmer):

(1) $$approximate\_\mathrm{coverage}\_\mathrm{ratio}\left(A,B\right)=\frac{Hit_{A}+Hit_{B}}{HS_{A}+HS_{B}},$$

where $Hit_{A}$ and $Hit_{B}$ represent the hash keys of species A and B, respectively, mapped into the hash tables of species A and B, and $HS_{A}$ and $HS_{B}$ represent the hash table sizes of species A and B, respectively. Although the k-mer-based method is not as accurate as LAST, it can rapidly estimate the homology between species, and the genome comparison can be completed in approximately 1 day.

The k-mer-based method can rapidly screen out species with suspected classification ambiguities; then, LAST can be applied for a more precise genome comparison. All aligned sequences must have an E-value of less than 10^−10^ to be considered homologous sequences. The homologous sequence is divided by the genome size to obtain the HCR, which is defined to describe the homology between two species as follows (simply as HCRlast):

(2) $$\mathrm{homologous}\_\mathrm{coverage}\_\mathrm{ratio}\left(A,\;B\right)=\frac{H_{A}+H_{B}}{S_{A}+S_{B}},$$

where $H_{A}$ and $H_{B}$ represent the lengths of homologous sequences aligned on the genomes of species A and B, respectively, and $S_{A}$ and $S_{B}$ represent the lengths of sequences of species A and B, respectively.

The HCRkmer and HCRlast methods are designed to be used in different contexts. The HCRkmer method is characterized by fast computation. This method can be used to quickly estimate the homology between genomes. By contrast, HCRlast is slow to calculate, but this method can provide more accurate results. In this paper, we used HCRkmer to quickly screen for species with suspected classification ambiguity and then used HCRlast to perform more accurate calculations and confirm the ambiguity of the classification. A server with 2 CPUs, 32 GB RAM, and 300 GB hard disk space are recommended to run the HCRkmer and HCRlast methods.

### 2.3. ANI and Other Tools

Both ANI and HCR are OGRI-based methods that use genome-wide comparisons for microbial classification. OrthoANIu (OrthoANI using USEARCH, refer to the website https://www.ezbiocloud.net/tools/orthoaniu) [11] is one of the tools used for implementing the ANI method. OrthoANIu is a recently released tool that has exhibited outstanding performance; thus, we selected OrthoANIu to compare the performance with HCRlast. The output of HCRlast and OrthoANIu is the difference between each pair of genomes. These differences can be expressed by distances; thus, conceptually, the output is more akin to a graphic representation rather than the phylogenetic tree that is customarily used in this field. Therefore, we introduced the multidimensional scaling (MDS) method to visualize results and highlight the effect of clustering. In addition, for generating the inference of the phylogenetic tree, FastTree (http://www.microbesonline.org/fasttree/) [12] was used to analyze the BAC120 dataset, and the ETE toolkit (http://etetoolkit.org) [13] was used to generate the image file.

## 3. Results

### 3.1. Large-Scale Genome Comparison

**Figure 1:**
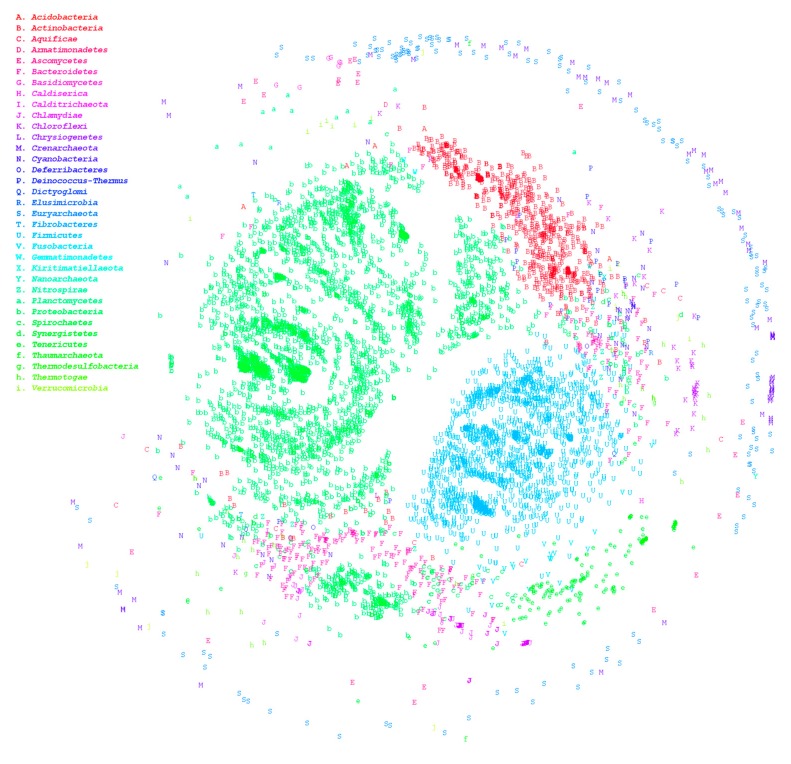
Multidimensional scaling ordination plot based on homologous distances among microorganisms. Genome-wide sequences of 7861 microbial strains were downloaded from the National Center for Biotechnology Information’s RefSeq, including 940 bacterial genera, 91 Archaea, and 17 fungal strains. Multidimensional scaling was drawn with homology between strains. Different colors represent different phyla.

**Figure 2:**
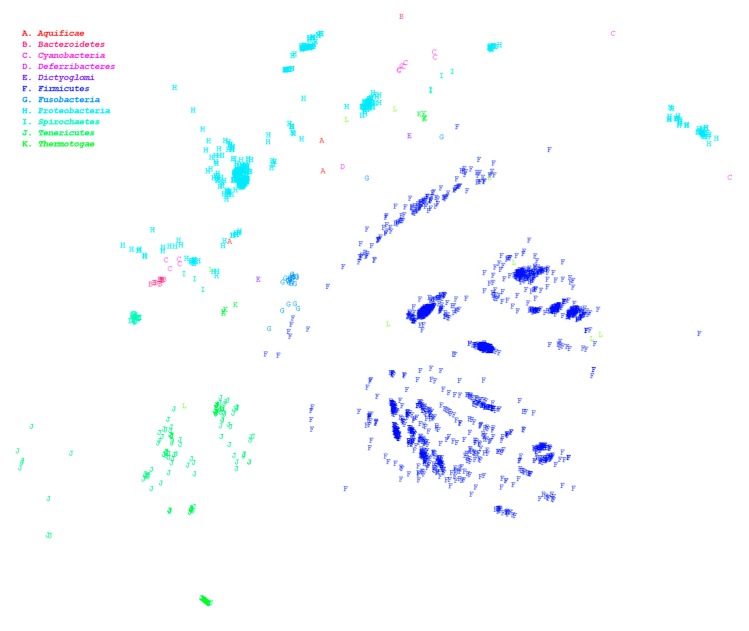
Multidimensional scaling ordination plot based on the homologous distances among microorganisms, including 2227 strains. Different colors represent different phyla.

The HCRkmer method can complete the comparison of the 7861 genomes within 3 days and screen out species with suspected classification ambiguities. Large-scale genome comparison enables us to acquire an overview of the distribution between microorganisms. In general, the homologous relationship between archaea and bacteria is relatively distant, which is consistent with the results of our analysis, as illustrated in Figure 1. In this figure, numerous data points falling under different phyla are overlapping, which signifies the existence of numerous misclassifications even at the phylum level. We then used HCRlast to verify whether taxonomic contradictions were present in these ambiguous species. After verification using HCRlast, we determined that 1220 genus pairs demonstrated ambiguous classification boundaries, signifying that the number of common intergenus homologous sequences was more than that of intragenus homologous sequences, as presented in Supplementary Table S2. The pair of *Mesoplasma* and *Mycoplasma* is an extreme example. The maximum HCR between *Mesoplasma* and *Mycoplasma* was approximately 0.393, and the minimum intragenus HCR in *Mycoplasma* was approximately 0.025. The intergenus HCR was 15 times more than the intragenus HCR. Even among the 1220 pairs, we observed that 292 genus pairs had ambiguous classification at the phylum level. *Mycoplasma* and *Sneathia* exhibited the most prominent ambiguity among the 292 pairs, and their maximum HCR was approximately 0.106. The interphylum HCR was four times the intraphylum HCR. Phylum represents the second-largest level of taxonomy. An ambiguous classification at the phylum level is a significant inconsistency in taxonomy. Therefore, in the present study, we re-evaluated all genera with interphylum and intraphylum HCR differences that were greater than 2. The phyla included Aquificae, Bacteroidetes, Cyanobacteria, Deferribacteres, Dictyoglomi, Firmicutes, Fusobacteria, Proteobacteria, Spirochaetes, Tenericutes, and Thermotogae are listed in Supplementary Table S3. A total of 2227 strains were used for genomic alignment. The results demonstrated that the minimum intraphylum HCRs in Proteobacteria, Tenericutes, Firmicutes, and Spirochaetes were low, as presented in Table 1, indicating that the intraphylum homology was relatively small and easily led to ambiguous classification. The HCR analysis results indicated that Proteobacteria and Spirochaetes were widely distributed and mixed with other phyla; these genera are illustrated as red and cyan points in Figure 2. Although Tenericutes and Firmicutes were also widely distributed, they were not mixed with other phyla. Table 2 presents strains with interphylum and intraphylum HCR differences greater than 4, and the maximum ratios could be up to 9 times the interphylum and intraphylum HCRs. For example, Assembly Accession GCA_002355135.1 and GCA_000090965.1 were determined to belong to Proteobacteria and Bacteroidetes, respectively. The two strains share more than 15% of the homologous sequences between them. By contrast, the minimum HCR in GCA_001417865.2 and GCA_001190755.1 belonging to Proteobacteria accounted for only approximately 1.5% of the homologous sequences.

### 3.2. Comparison of HCR and ANI Methods

The analysis detailed in the previous chapter demonstrated that the HCR of the cross-phylum species was greater than that of the intraphylum species, indicating that the cross-phylum species have more homologous sequences than the intraphylum species. The sharing of more homologous sequences by cross-phylum species does not prove that these species have a more intimate evolutionary relationship, and evolutionary homology is a taxonomically critical factor. Therefore, it is necessary to more carefully assess the classification ambiguity at the genus level. In the analysis and calculation, the scale of the cross-genus analysis is relatively small, and it is easy to observe the fuzzy classification boundary between the genera. Fifteen pairs of genera with fuzzy classification boundaries were screened from 7861 complete genomes by HCRkmer, as shown in Table 3. The analysis of 15 pairs of genera using HCRlast revealed that five pairs of genera were observed to have a cross-genus HCR greater than intragenus HCR, and these are *Clostridium* and *Ruminiclostridium*, *Corynebacterium* and *Brevibacterium*, *Kluyvera* and *Enterobacter*, *Kluyvera* and *Escherichia*, and *Lelliottia* and *Enterobacter*. The cross-genus HCR was greater than the intragenus HCR. The homology of the genome with some cross-genus strains was closer than that of intragenus strains. The pair of *Clostridium* and *Ruminiclostridium* is an obvious example. HCR = 0.121 for GCF_000620945.1 and GCF_000953215.1 and HCR = 0.192 for GCF_000620945.1 and GCF_002161175.1, where GCF_000620945.1 and GCF_000953215.1 belongs to *Ruminiclostridium* and GCF_002161175.1 belongs to *Clostridium*. The cross-genus HCR was 0.071 more than the intragenus HCR and was conceptually equivalent to approximately 193,674 bases of homologous sequences.

In this study, OrthoANIu was used to revalidate the 15 pairs of genera with inconspicuous classification boundaries, and results were roughly similar to those of HCRlast, as shown in Supplemental Figure S2A through Figure S16A. However, in a few cases, HCRlast performed better than OrthoANIu, namely for *Clostridium* and *Ruminiclostridium*, *Erythrobacter* and *Altererythrobacter*, and *Roseburia* and *Clostridium*. As shown in Supplemental Figure S4A, Figure S7A, and Figure S13A, the results of OrthoANIu revealed blurred boundaries between different genera. In the results of HCRlast, the classification boundaries were relatively clear. In addition, HCRlast was 12 times faster than OrthoANIu. On the same server, using 20 threads and comparing 192 genomes, HCRlast completed work in 255 minutes compared with the 3270 minutes taken by OrthoANIu to complete the work.

### 3.3. Validation by BAC120

**Figure 3:**
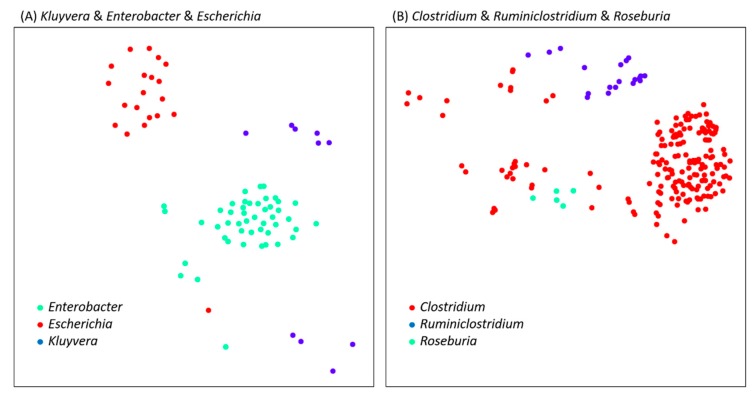
Multidimensional scaling ordination plot based on homologous distances among the dataset *Kluyvera* and *Enterobacter* and *Escherichia* (**A**) and *Clostridium* and *Ruminiclostridium* and *Roseburia* (**B**). Different colors represent different genera.

The results of HCRlast and OrthoANIu demonstrated category boundary ambiguities at the genus level, and the analysis of these boundary-blurred species in BAC120 also yielded the same conclusion, as shown in Supplemental Figure S2B to Figure S16B. Two groups of three genera with obvious classification boundary ambiguity were identified, namely *Clostridium* and *Ruminiclostridium*, *Roseburia* and *Kluyvera*, and *Enterobacter* and *Escherichia*. From the phylogenetic tree derived from BAC120, it can be determined that various genus strains are interdigitated with each other, as shown in Supplemental Figures S17 and S18. The HCRlast analysis reached the same conclusion as BAC120. No obvious grouping boundary appeared between genera, such as *Kluyvera* and *Enterobacter* and *Escherichia*, as depicted in Figure 3A. However, the different genera of the *Clostridium* and *Ruminiclostridium* and *Roseburia* dataset exhibited better clustering effects, as shown in Figure 3B. These analysis results can be divided into one of the following two categories: HCRlast and BAC120 analysis results—both exhibited boundary blur, but HCRlast resulted in clear boundaries, even without obvious clustering.

## 4. Discussion

Phylogeny, morphology, and chemotaxonomy constitute the fundamental basis for microbial taxonomy. In the present study, we did not intend to challenge the current status of microbial taxonomy but rather to highlight prevailing inconsistencies observed from whole genome–based analyses. Species should be re-evaluated to facilitate appropriate classification when common intragenus homologous sequences are more than intergenus homologous sequences. Whales are classified as mammals because they exhibit numerous mammalian traits. This raises the question whether we should revisit such ambiguous microbes in greater detail and assign new taxonomic names. Ambiguity in classification is not simply a taxonomic problem. It would further influence the accuracy of species identification.

In the preliminary analysis, we determined that most of the species exhibit an adequate clustering phenomenon and only a small number of species are scattered and have blurred boundaries. These border-blurred species were examined in a more precise manner, and results demonstrated that the maximum cross-genus HCR of these border-blurred species was greater than the minimum intragenus HCR, as shown in Supplementary Table S2. Cross-genus HCR was greater than intragenus HCR, indicating that at least two different genera had more homologous sequences than the same genus; for example, GCF_000702725.1 and GCF_000376625.1, which belong to *Mesoplasma* and *Mycoplasma*, respectively. In this case, the HCR was 0.367, while the HCR between GCF_000376625.1 and GCF_000238995.1, which is also a *Mycoplasma*, was 0.049, which is much smaller than the former. *Mycoplasma* is an extreme example. Among the top 10 HCRs in Supplementary Table S2, *Mycoplasma* accounts for three. This genus is widely distributed and has a blurred classification boundary with multiple genera, as shown in Supplemental Figure S19. As shown in Figure 2, some phyla have similar classification ambiguities, but the distance according to HCR does not easily prove the fuzzy classification of cross-phyla because there may be some neglected species between these phyla that can connect them. Therefore, we expended considerable effort to demonstrate that there is indeed a classification ambiguity at the genus level, as shown in Table 3 and Supplemental Figure S2 to Figure S16. The existence of genus-level classification ambiguity introduces the risk of misjudging the appropriate tool for making species identifications on the basis of genome-wide comparisons. Species identification must be performed in units of species to avoid the fuzzy interference of genus-level classification.

Our proposed method is a type of OGRI, and we used OrthoANIu for performance comparison. As mentioned in the previous section, HCRlast was 12 times faster than OrthoANIu. In most cases, the two tools are equally effective, but when the difference between the genomes is small, HCRlast is superior to OrthoANIu, as depicted in Supplemental Figure S4A, Figure S7A, and Figure S13A. Overall, the sensitivity of HCRlast was relatively high, and the algorithm was relatively simple. Unlike the ANI method, which needs to simulate the DDH method, the simple algorithm can directly reflect the difference between two genomes. In some cases, HCRlast can produce more reasonable results than BAC120, such as for *Clostridium* and *Ruminiclostridium*. These two genera were originally the same genus. In 2013, it was suggested to separate them into different genera [14]. As shown in Supplemental Figure S4, the phylogenetic tree of BAC120 indicates that the genomes of the two genera are interdigitated; OrthoANIu obtains similar results, but HCRlast can clearly separate them. Although choosing different combinations of genes in BAC120 may be possible to clearly separate *Clostridium* and *Ruminiclostridium*, it might create bias resulting from the selection of different marker genes. In addition, a large number of microorganisms have not been discovered. The more species of microorganisms are discovered, the more stringent the selection of marker genes will become.

Current microbial taxonomy activities use the phylogenetic inference of marker genes as a basis for classification, indicating that a homologous sequence that is not selected as a marker gene would be overlooked. Therefore, the selection of a marker gene affects classification results. The HCR can be used to determine the genetic distances between species. The HCR is a whole-genome comparison tool that can exclude the bias attributed to the marker gene selection. We re-examined current microbial taxonomy by using the HCR as a criterion to determine any inconsistencies. For example, species from different groups could share more homologous sequences than species within a group, indicating that taxonomy is ambiguous.

## Figures and Tables

**Table 1:** Top 10 pairs of assembly accessions with minimum intraphylum homologous coverage ratio.

Phylum	Minimum Homologous Coverage Ratio of Intragenus	Assembly Accession	Assembly Accession
Proteobacteria	0.015999617	GCA_001417865.2	GCA_001190755.1
Tenericutes	0.024333677	GCA_000186985.3	GCA_001886855.1
Firmicutes	0.026170854	GCA_900183405.1	GCA_001010825.1
Spirochaetes	0.048420481	GCA_001936255.1	GCA_000092845.1
Fusobacteria	0.140362380	GCA_002356455.1	GCA_000024565.1
Bacteroidetes	0.166804691	GCA_000348805.1	GCA_002369955.1
Cyanobacteria	0.216424948	GCA_000015705.1	GCA_000015665.1
Thermotogae	0.220607538	GCA_000953715.1	GCA_001941385.1
Aquificae	0.221942947	GCA_000021545.1	GCA_000191045.1
Dictyoglomi	0.749914757	GCA_000020965.1	GCA_000021645.1

**Table 2:** Top 13 pairs of assembly accessions with maximum interphylum homologous coverage ratio.

Phylum A	Phylum B	Minimum Homologous Coverage Ratio of Intragenus (Phylum A)	Maximum Homologous Coverage Ratio of intergenus	Assembly Accession	Assembly Accession
Proteobacteria	Bacteroidetes	0.015999617	0.151368772	GCA_002355135.1	GCA_000090965.1
Proteobacteria	Deferribacteres	0.015999617	0.112992155	GCA_000284355.1	GCA_000010985.1
Proteobacteria	Aquificae	0.015999617	0.112416204	GCA_000284355.1	GCA_000021545.1
Firmicutes	Dictyoglomi	0.026170854	0.170361824	GCA_000092965.1	GCA_000020965.1
Proteobacteria	Tenericutes	0.015999617	0.101309783	GCA_001262715.1	GCA_900016775.1
Proteobacteria	Firmicutes	0.015999617	0.096263109	GCA_000284355.1	GCA_000014125.1
Proteobacteria	Fusobacteria	0.015999617	0.093542786	GCA_000816185.1	GCA_001296125.1
Firmicutes	Deferribacteres	0.026170854	0.13067773	GCA_000165465.1	GCA_000010985.1
Proteobacteria	Cyanobacteria	0.015999617	0.077366777	GCA_000011465.1	GCA_000008885.1
Proteobacteria	Dictyoglomi	0.015999617	0.074348485	GCA_002220775.1	GCA_000021645.1
Firmicutes	Aquificae	0.026170854	0.119079275	GCA_000025645.1	GCA_000191045.1
Tenericutes	Firmicutes	0.024333677	0.106030272	GCA_001702115.1	GCA_002441935.1
Tenericutes	Fusobacteria	0.024333677	0.104966983	GCA_000439435.1	GCA_000024565.1

**Table 3:** Fifteen pairs of genera with ambiguous classification at the genus level. HCR: homologous coverage ratio.

Genus A	Genus B	Max HCR of Cross-Genus	Genome Comparison of Cross-Genus	Max HCR of Intragenus	Genome Comparison of Intragenus
*Alicycliphilus*	*Acidovorax*	0.521	GCF_000175235.1 & GCF_000204645.1	0.6	GCF_000175235.1 & GCF_002157165.1
*Chlorobium*	*Chlorobaculum*	0.324	GCF_000012585.1 & GCF_000006985.1	0.37	GCF_000012585.1 & GCF_000020645.1
*Clostridium*	*Ruminiclostridium*	0.192	GCF_000620945.1 & GCF_002161175.1	0.121	GCF_000620945.1 & GCF_000953215.1
*Corynebacterium*	*Brevibacterium*	0.228	GCF_000720035.1 & GCF_900184225.1	0.19	GCF_000720035.1 & GCF_001941505.1
*Diaphorobacter*	*Acidovorax*	0.515	GCF_000175235.1 & GCF_000015545.1	0.6	GCF_000175235.1 & GCF_002157165.1
*Erythrobacter*	*Altererythrobacter*	0.479	GCF_000013005.1 & GCF_900177715.1	0.493	GCF_000013005.1 & GCF_900115585.1
*Histophilus*	*Haemophilus*	0.481	GCF_002015075.1 & GCF_000027305.1	0.557	GCF_002015075.1 & GCF_000011785.1
*Kluyvera*	*Enterobacter*	0.598	GCF_000321045.1 & GCF_900168315.1	0.578	GCF_000321045.1 & GCF_001888805.2
*Kluyvera*	*Escherichia*	0.549	GCF_000759795.1 & GCF_900112785.1	0.506	GCF_000759795.1 & GCF_000350705.1
*Lelliottia*	*Enterobacter*	0.709	GCF_001652505.2 & GCF_001729725.1	0.702	GCF_001652505.2 & GCF_002811785.1
*Pseudodesulfovibrio*	*Desulfovibrio*	0.397	GCF_000422565.1 & GCF_000189295.2	0.397	GCF_000422565.1 & GCF_900188225.1
*Roseburia*	*Clostridium*	0.194	GCF_900111235.1 & GCF_001940165.1	0.2	GCF_900111235.1 & GCF_900112775.1
*Serratia*	*Chania*	0.575	GCF_001976145.1 & GCF_002588845.1	0.697	GCF_001976145.1 & GCF_000743365.1
*Sphingomonas*	*Rhizorhabdus*	0.33	GCF_000512205.2 & GCF_000715175.2	0.362	GCF_000512205.2 & GCF_001717955.1
*Vibrio*	*Aliivibrio*	0.312	GCF_002100145.1 & GCF_001691025.1	0.364	GCF_002100145.1 & GCF_000280885.2

## Supplementary Materials

The following are available online at https://www.mdpi.com/2076-2607/7/6/161/s1.
