# Supplementary material for: A New Genome-to-Genome Comparison Approach for Large-Scale Revisiting of Current Microbial Taxonomy
Source: Microorganisms. 2019 Jun 3;7(6):161. doi: 10.3390/microorganisms7060161 (PMC6616886; doi:10.3390/microorganisms7060161)
Supplement: Supplementary file 1 [file microorganisms-07-00161-s001.zip › supplementary_files/Supplemental_Figures.pdf]

Supplementary Information

A new genome-to-genome comparison approach for large-scale revisiting of current microbial taxonomy

Ming-Hsin Tsai, Yen-Yi Liu, and Von-Wun Soo, Chih-Chieh Chen

This PDF file includes:

Supplementary Figures 1-19

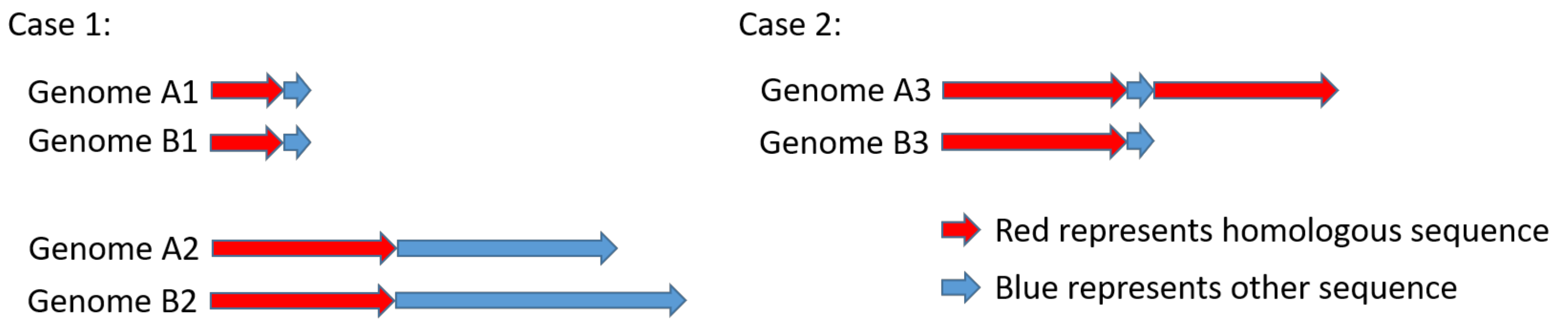

Supplemental figure 1. Two scenarios that consider the distance between homologous relationships between species. Case 1, the ratio of the length of the homologous sequence to the size of the genome should be considered, since the size of the microbial genome varies greatly. For example, the homologous coverage ratio of genomes A1 and B1 is greater than that of genomes A2 and B2, which is more biologically significant, even though the homologous coverage lengths of genomes A2 and B2 are longer than A1 and B1. Case 2, it should be considered that part of the repeat sequence in the genome is a homologous sequence.

(A)

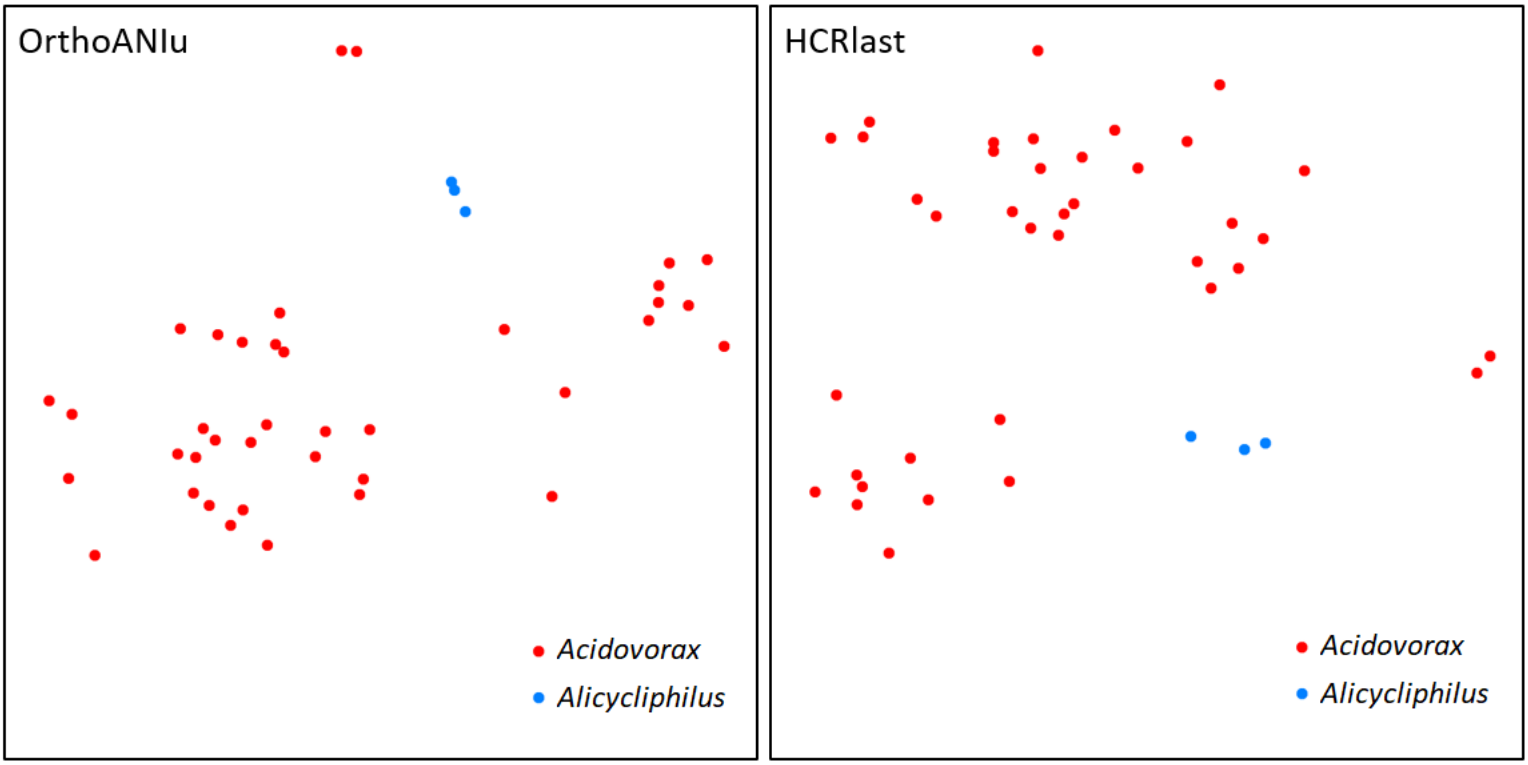

(B)

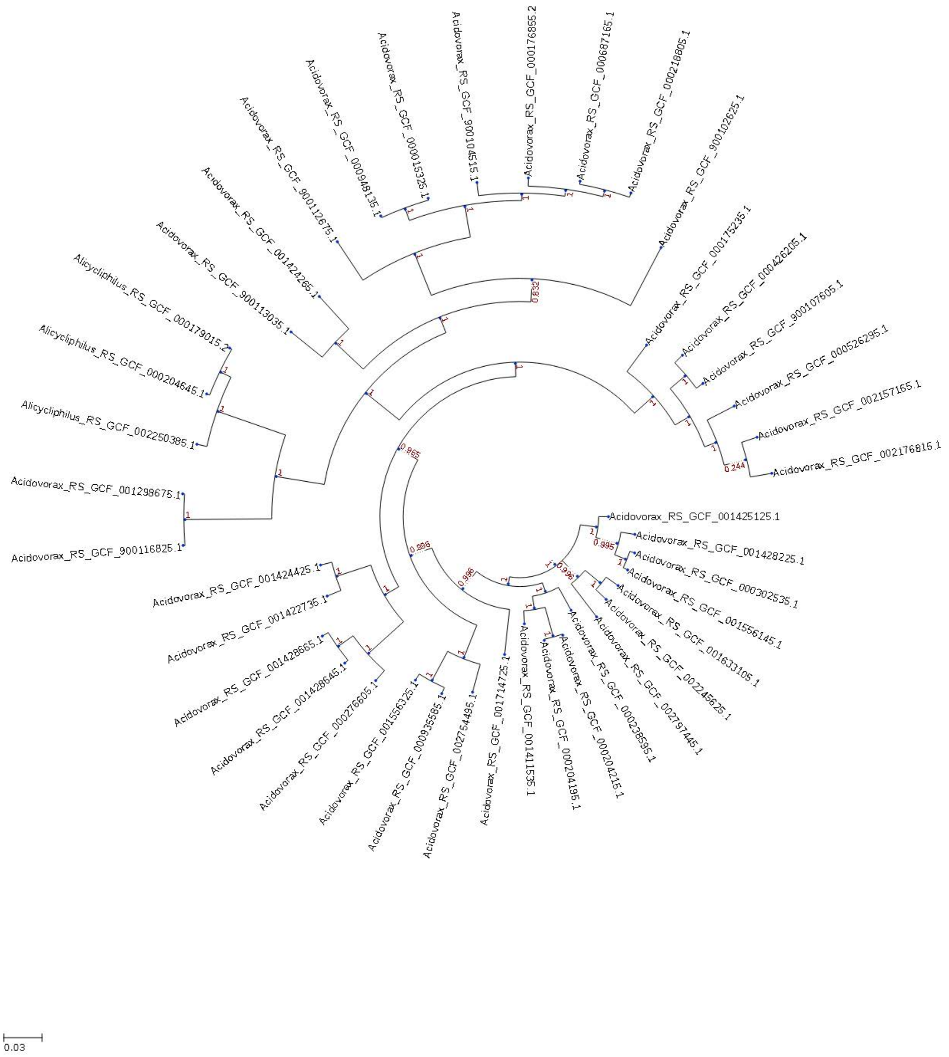

Supplemental figure 2. (A) MDS plot of *Alicycliphilus* and *Acidovorax* in methods OrthoANIu and HCRlast. (B) Phylogenetic tree of *Alicycliphilus* and *Acidovorax.*

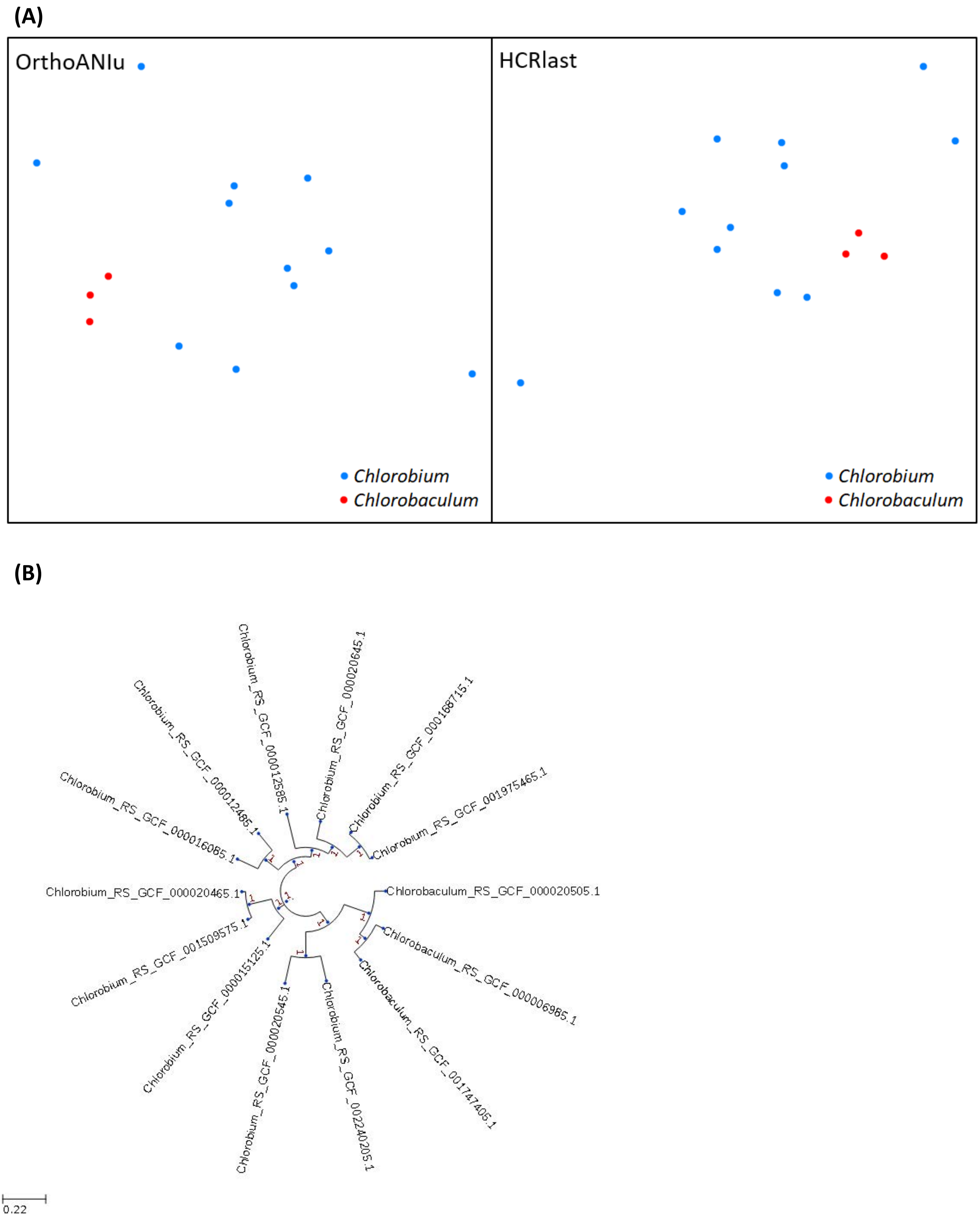

Supplemental figure 3. (A) MDS plot of *Chlorobium* and *Chlorobaculum* in methods OrthoANIu and HCRlast. (B) Phylogenetic tree of *Chlorobium* and *Chlorobaculum.*

(A)

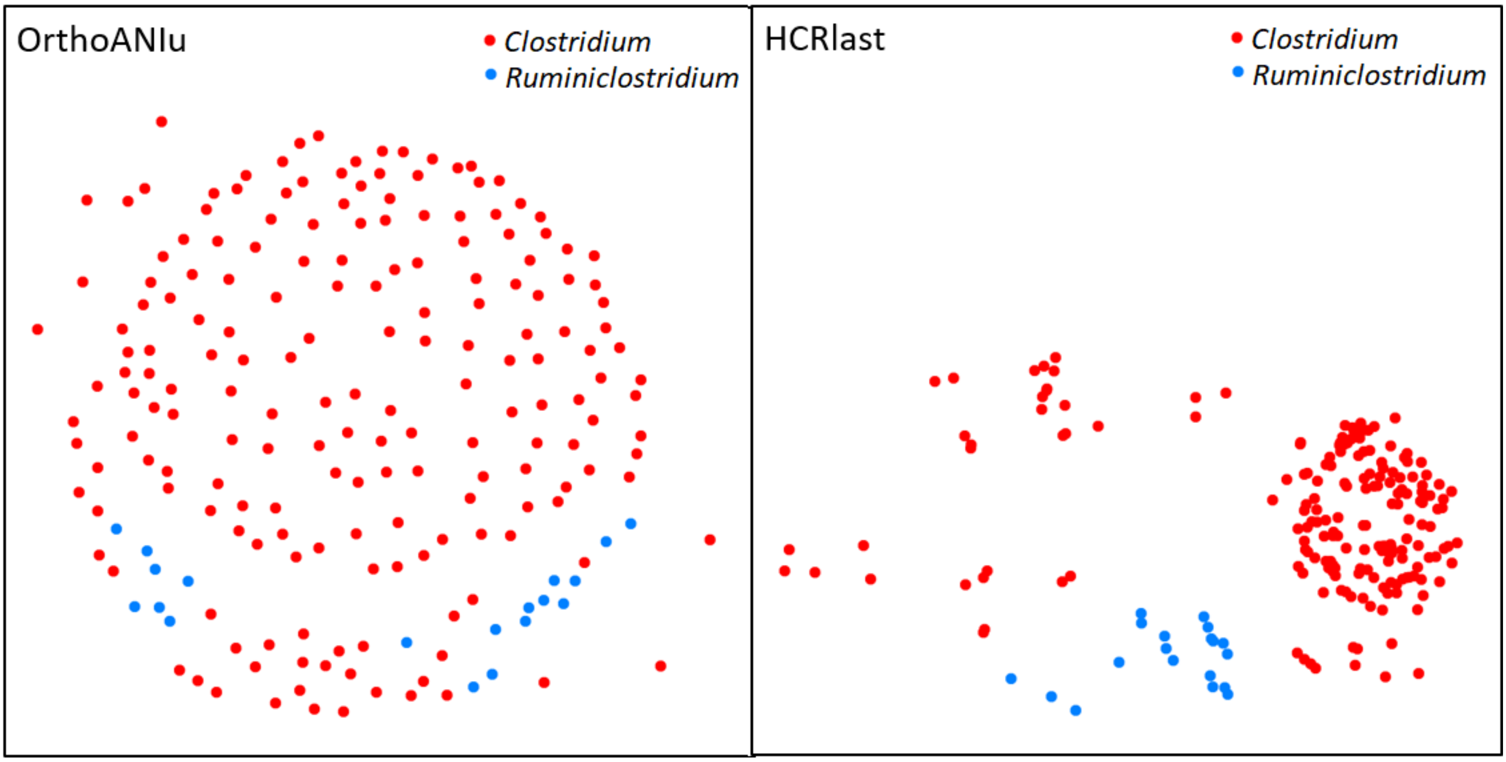

(B)

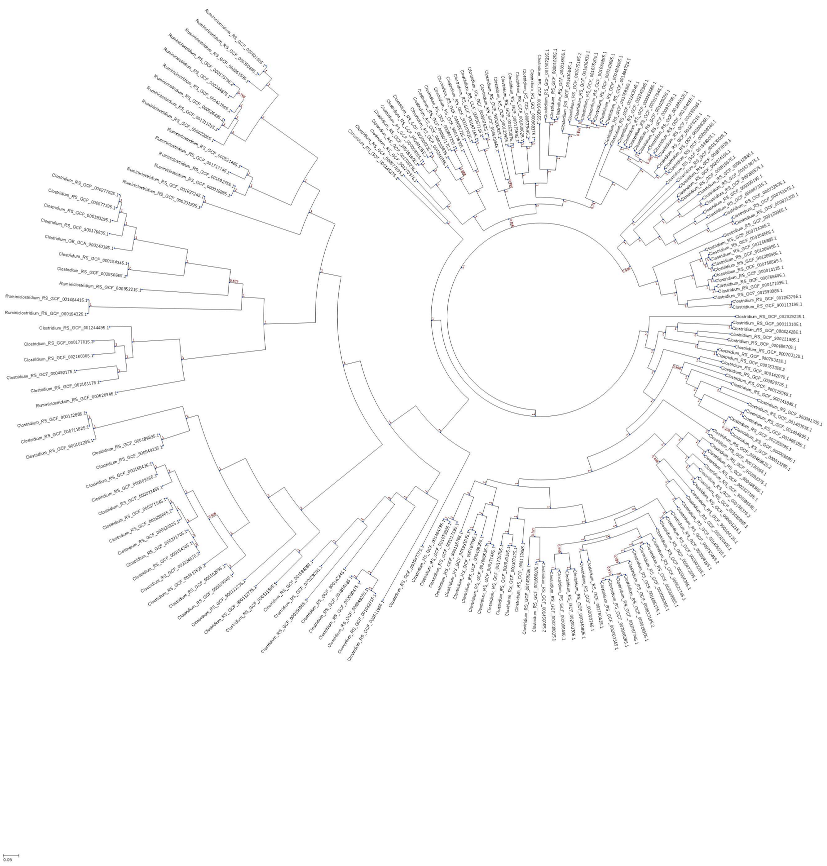

Supplemental figure 4. (A) MDS plot of *Clostridium* and *Ruminiclostridium* in methods OrthoANIu and HCRlast. (B) Phylogenetic tree of *Clostridium* and *Ruminiclostridium.*

(A)

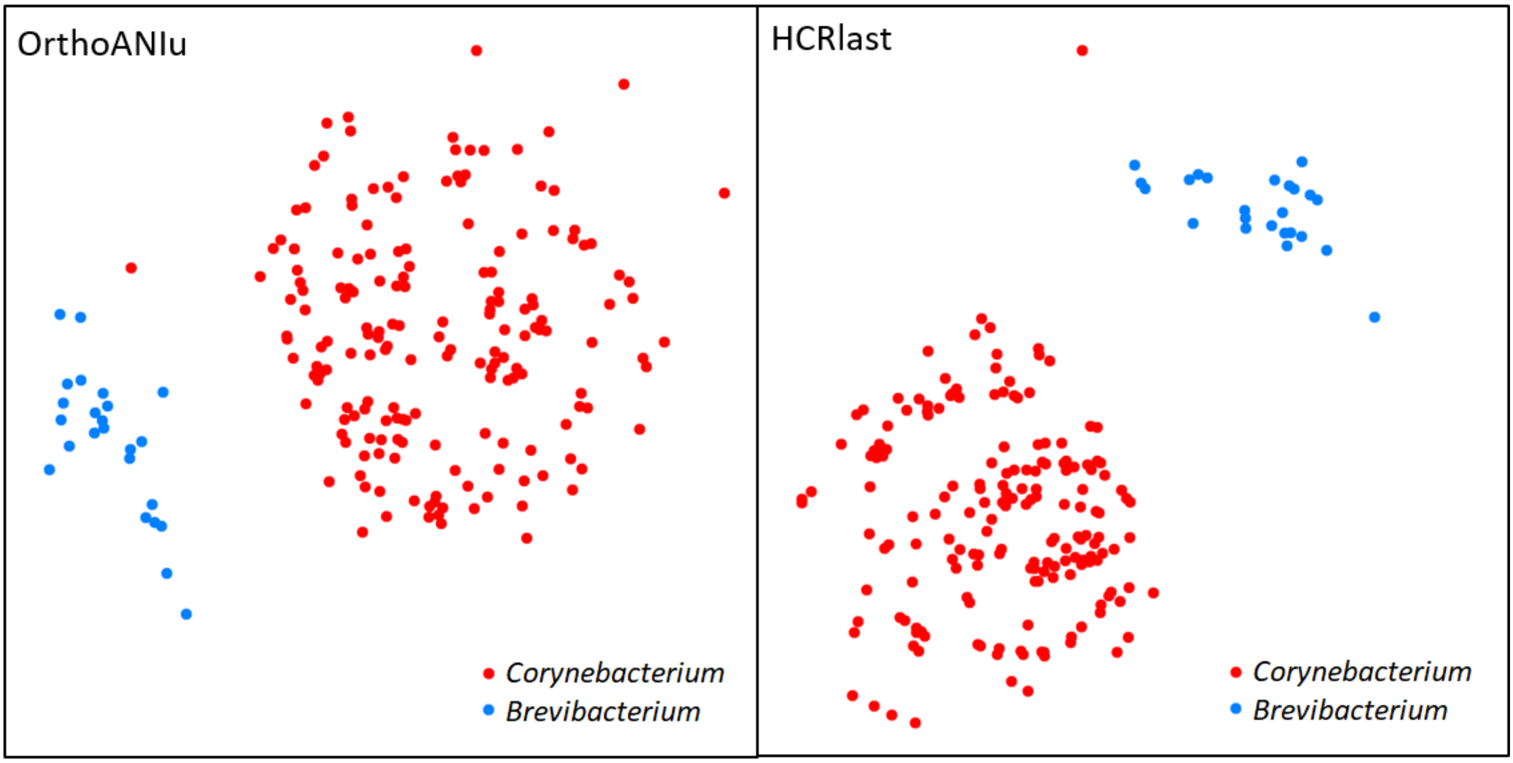

(B)

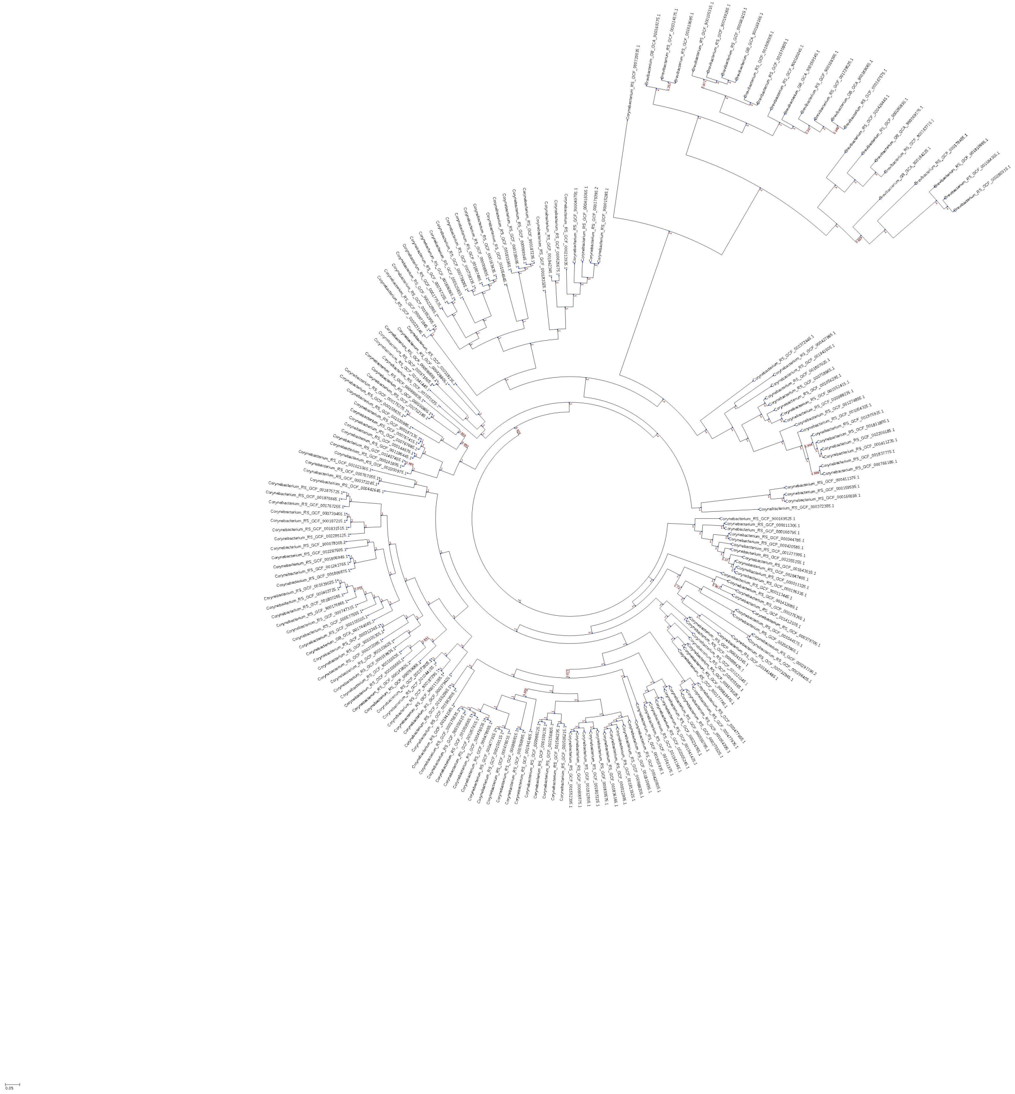

Supplemental figure 5. (A) MDS plot of *Corynebacterium* and *Brevibacterium* in methods OrthoANIu and HCRlast. (B) Phylogenetic tree of *Corynebacterium* and *Brevibacterium.*

(A)

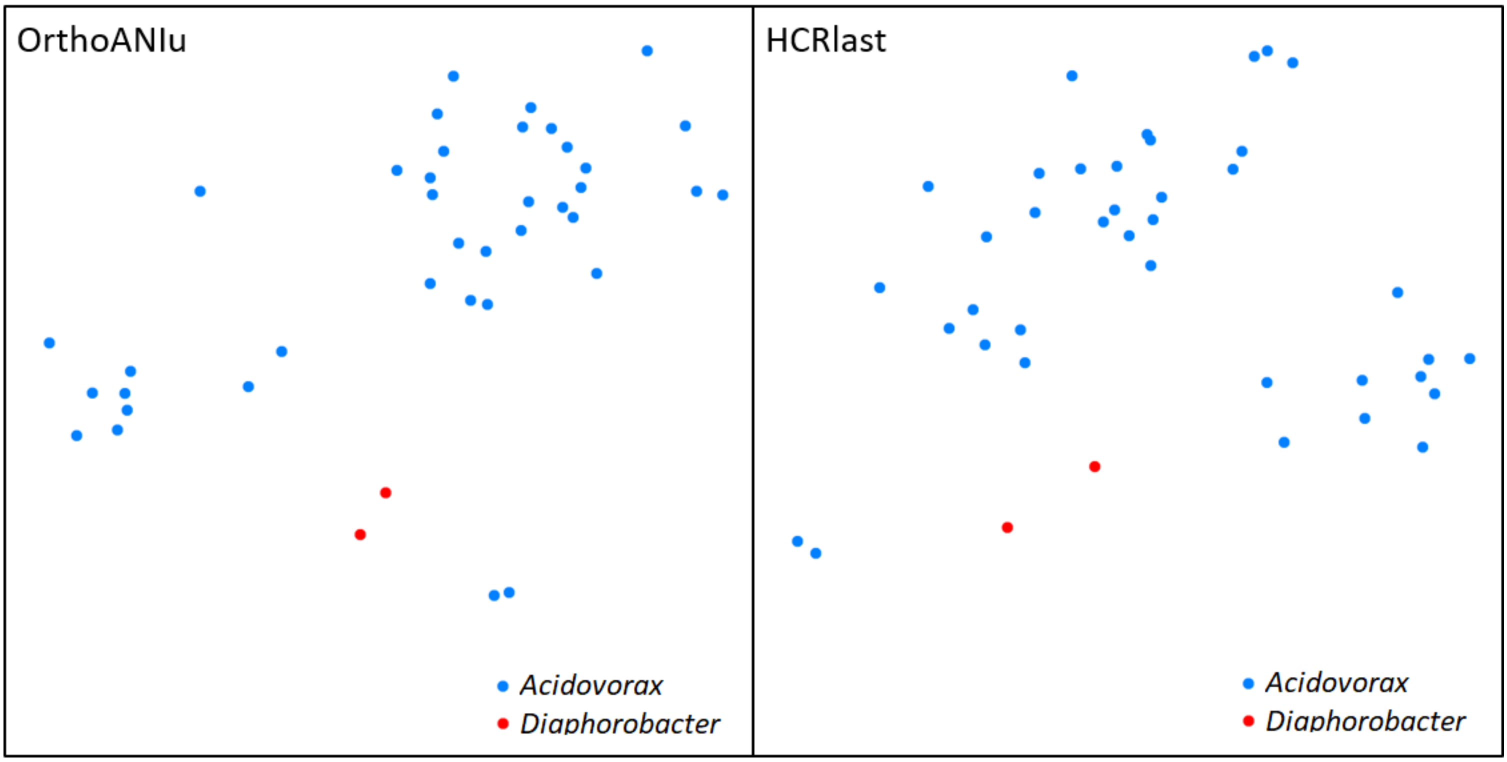

(B)

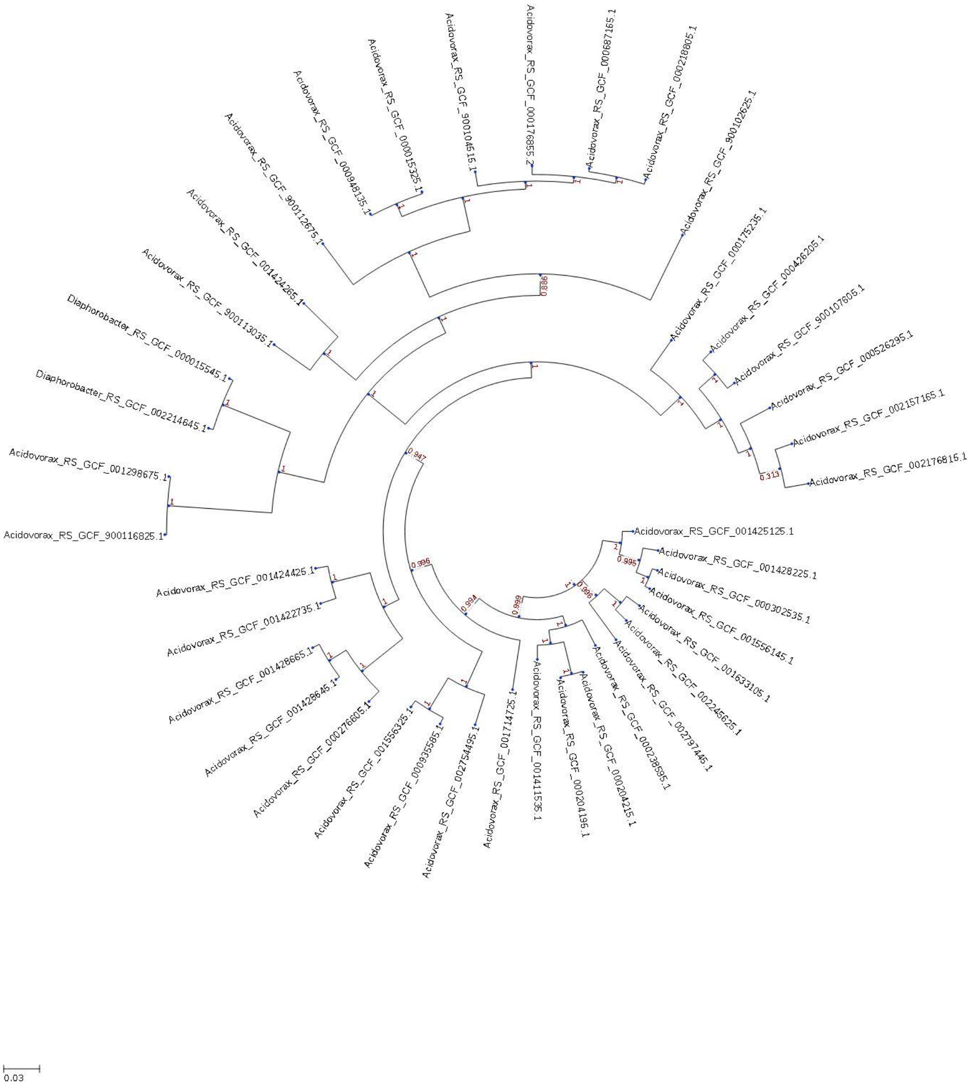

Supplemental figure 6. (A) MDS plot of *Diaphorobacter* and *Acidovorax* in methods OrthoANIu and HCRlast. (B) Phylogenetic tree of *Diaphorobacter* and *Acidovoraxb.*

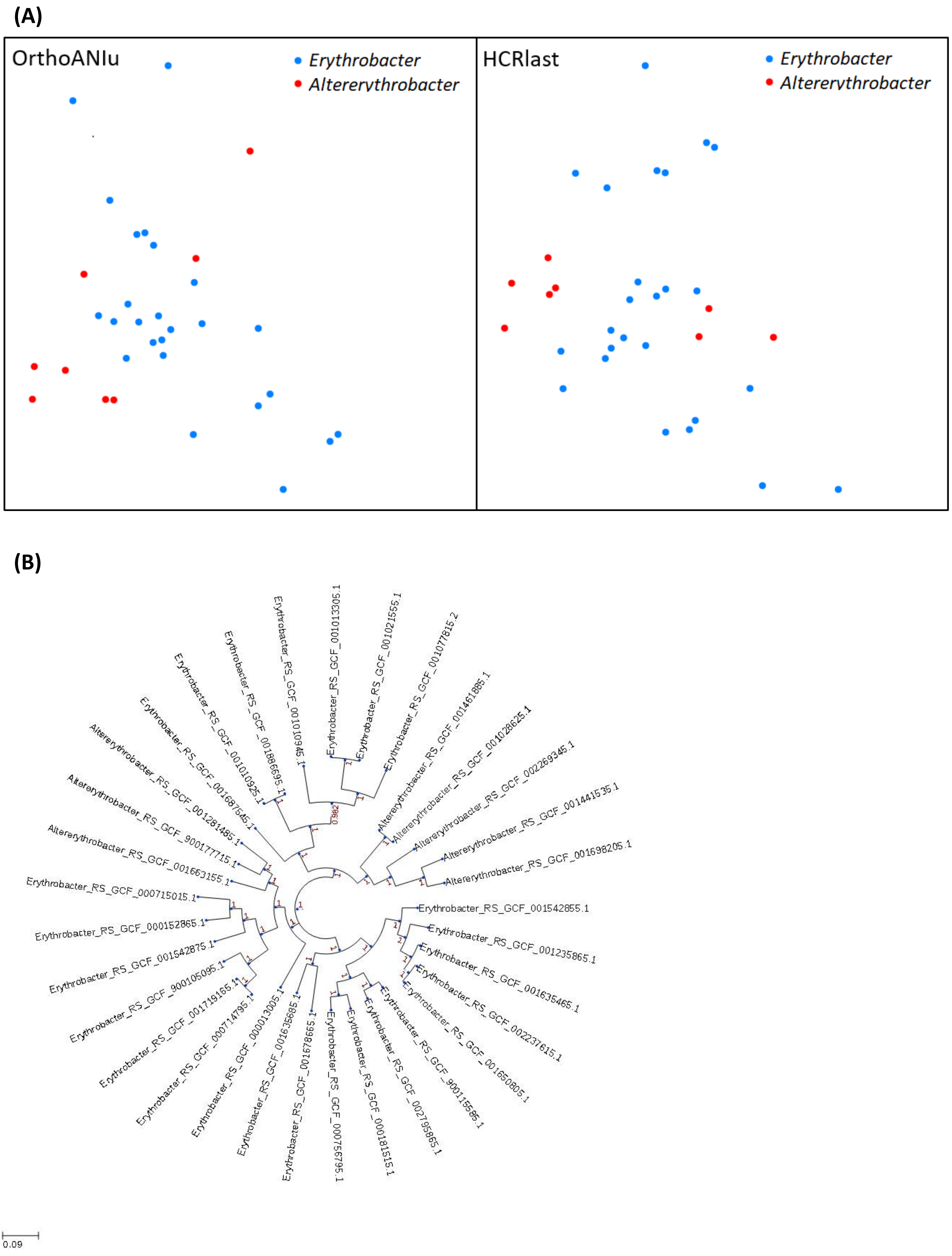

Supplemental figure 7. (A) MDS plot of *Erythrobacter* and *Altererythrobacter* in methods OrthoANIu and HCRlast. (B) Phylogenetic tree of *Erythrobacter* and *Altererythrobacter.*

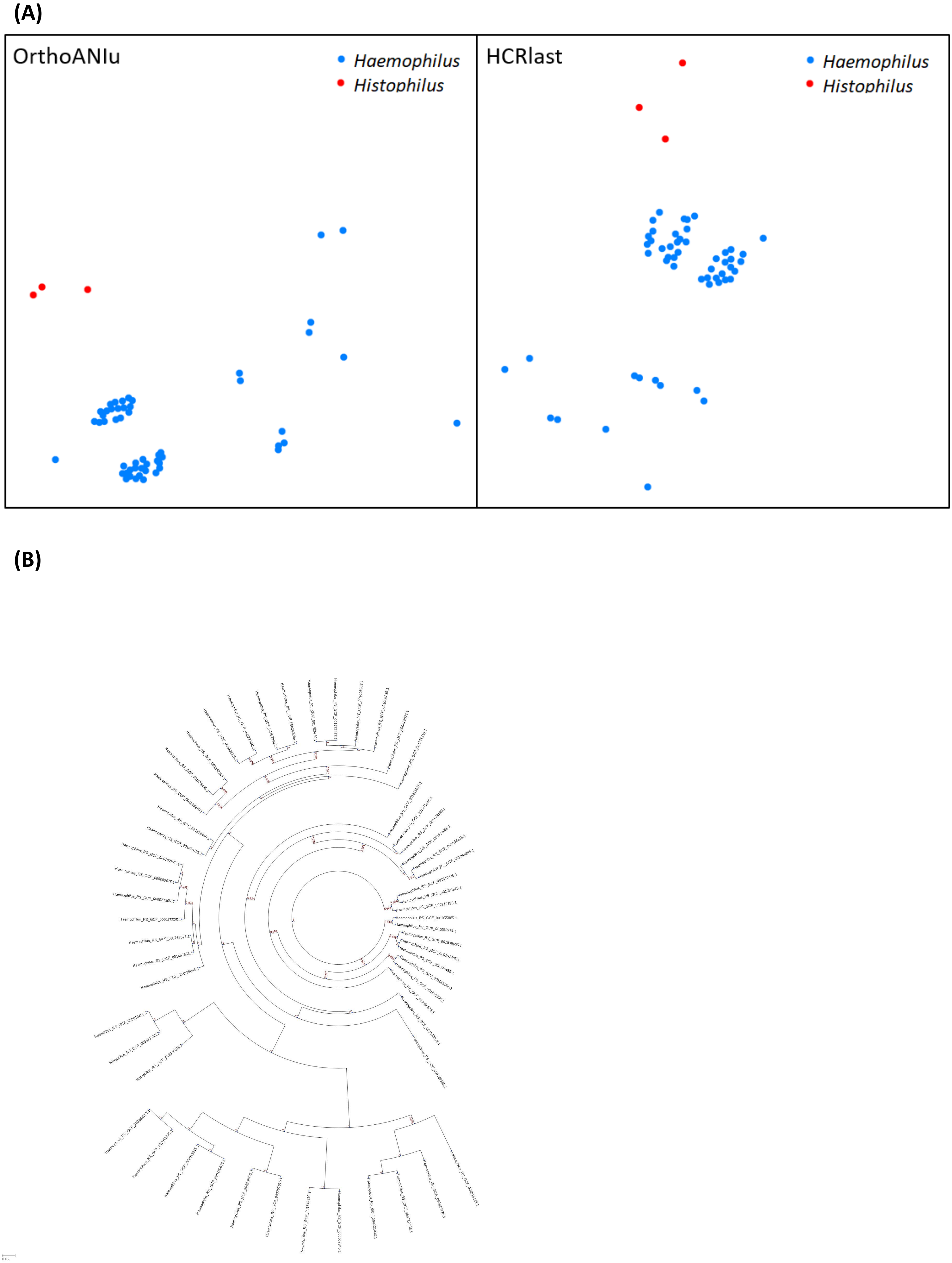

Supplemental figure 8. (A) MDS plot of *Histophilus* and *Haemophilus* in methods OrthoANIu and HCRlast. (B) Phylogenetic tree of *Erythrobacter* and *Altererythrobacter.*

(A)

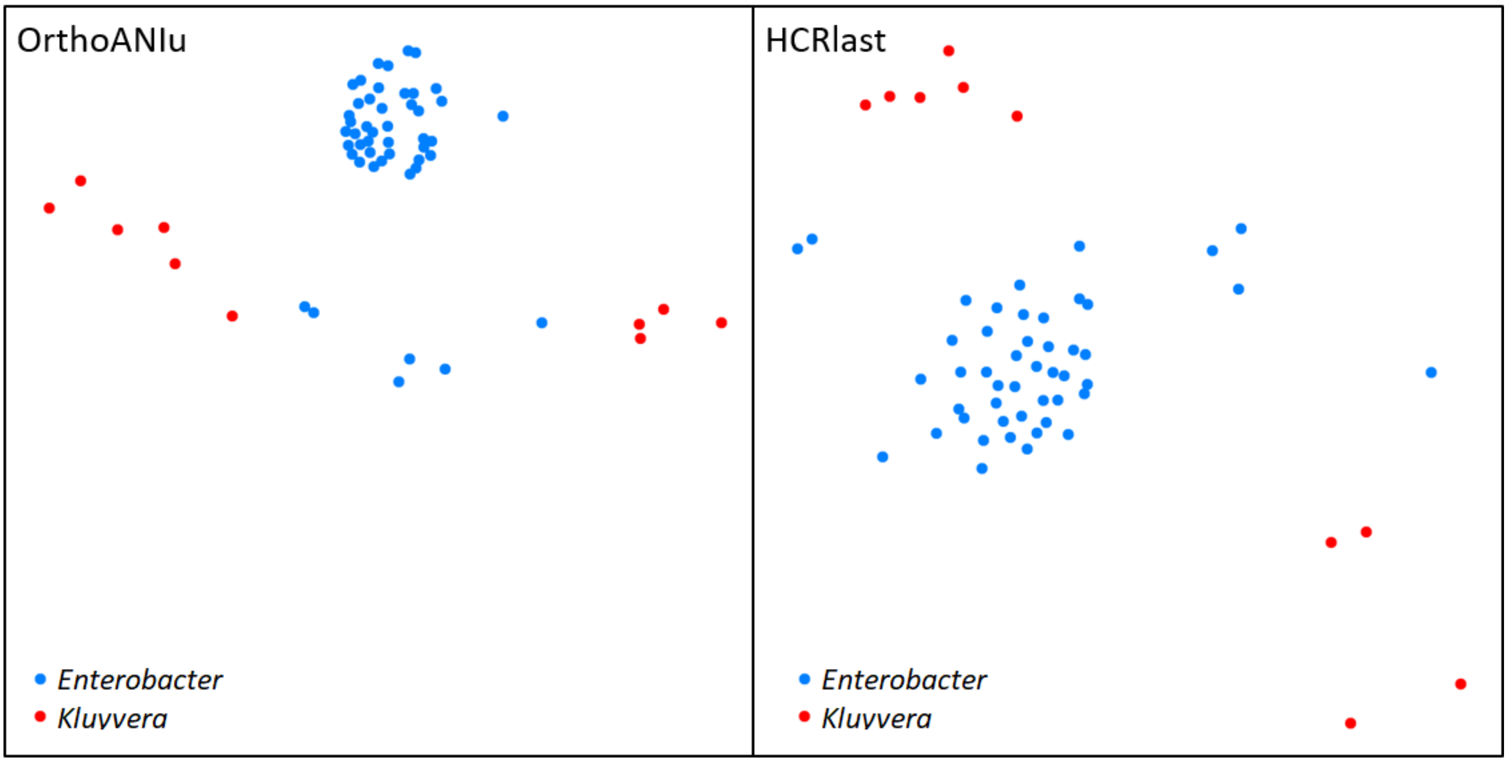

(B)

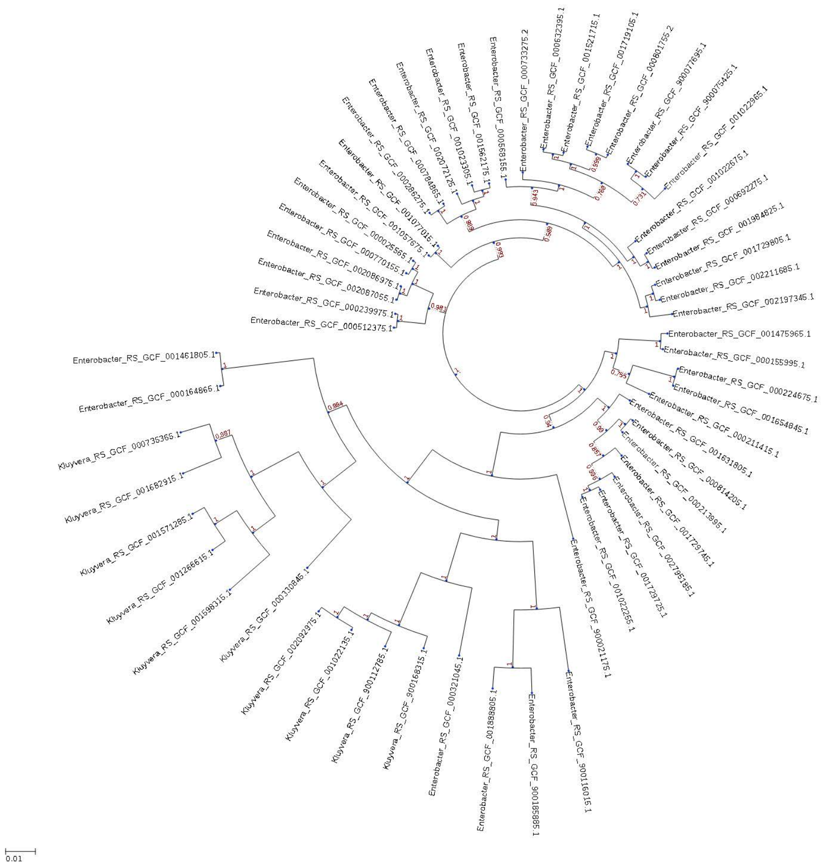

Supplemental figure 9. (A) MDS plot of *Kluyvera* and *Enterobacter* in methods OrthoANIu and HCRlast. (B) Phylogenetic tree of *Kluyvera* and *Enterobacter.*

(A)

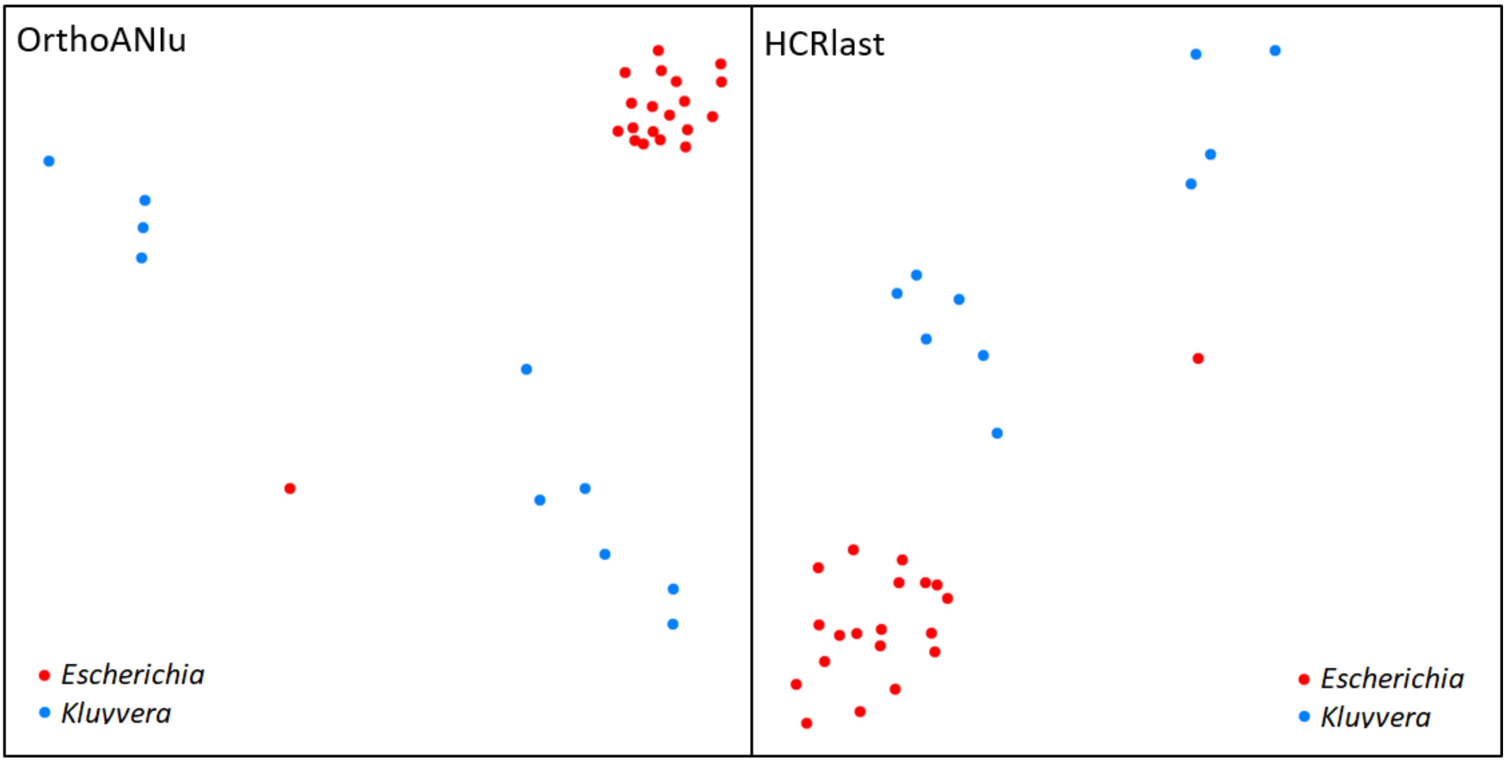

(B)

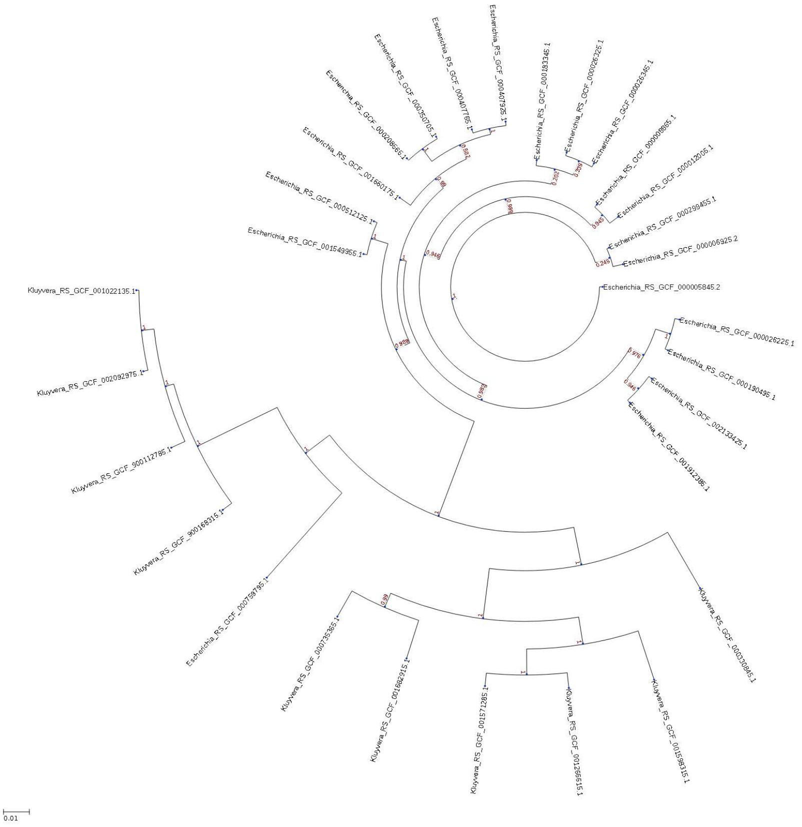

Supplemental figure 10. (A) MDS plot of *Kluyvera* and *Escherichia* in methods OrthoANIu and HCRlast. (B) Phylogenetic tree of *Kluyvera* and *Escherichia.*

(A)

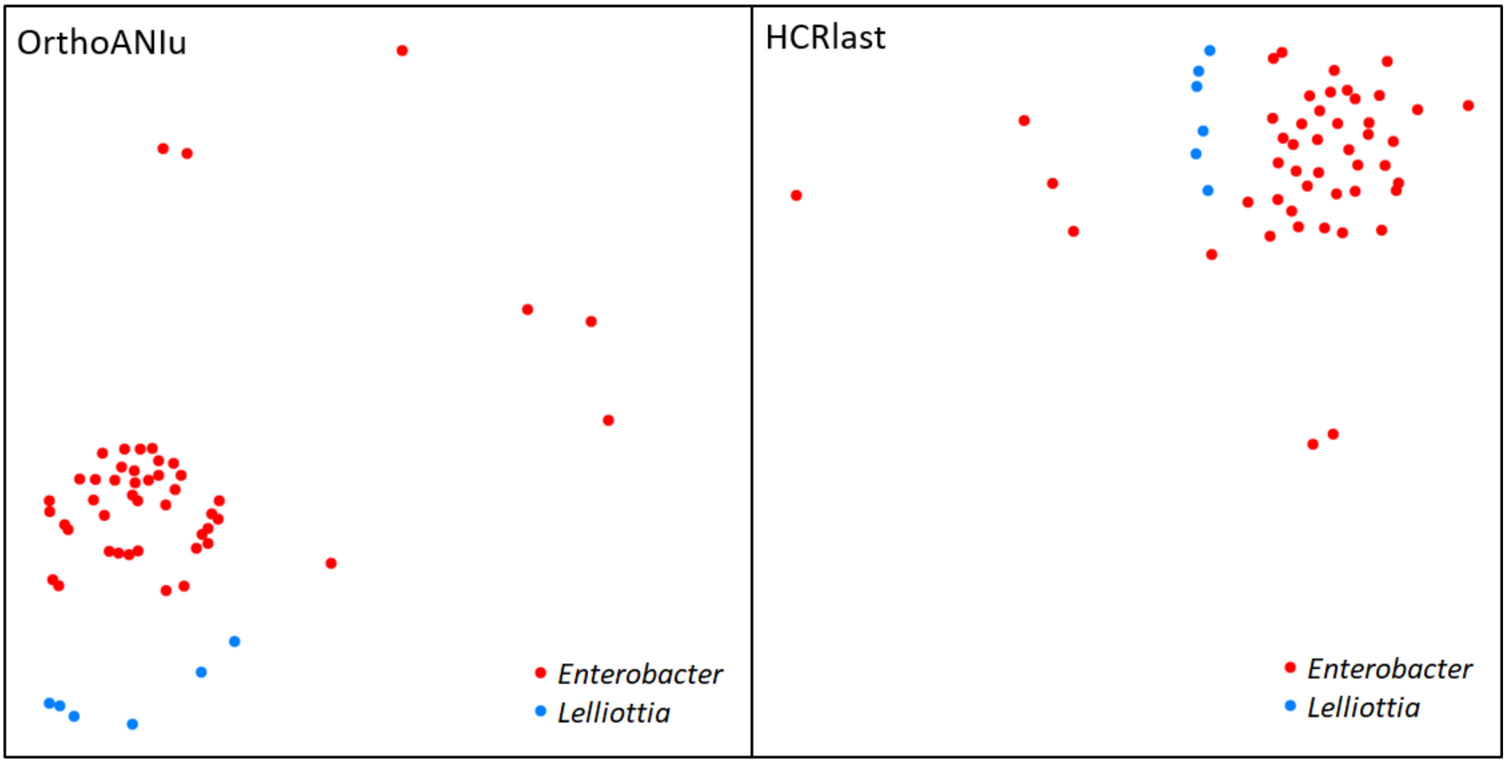

(B)

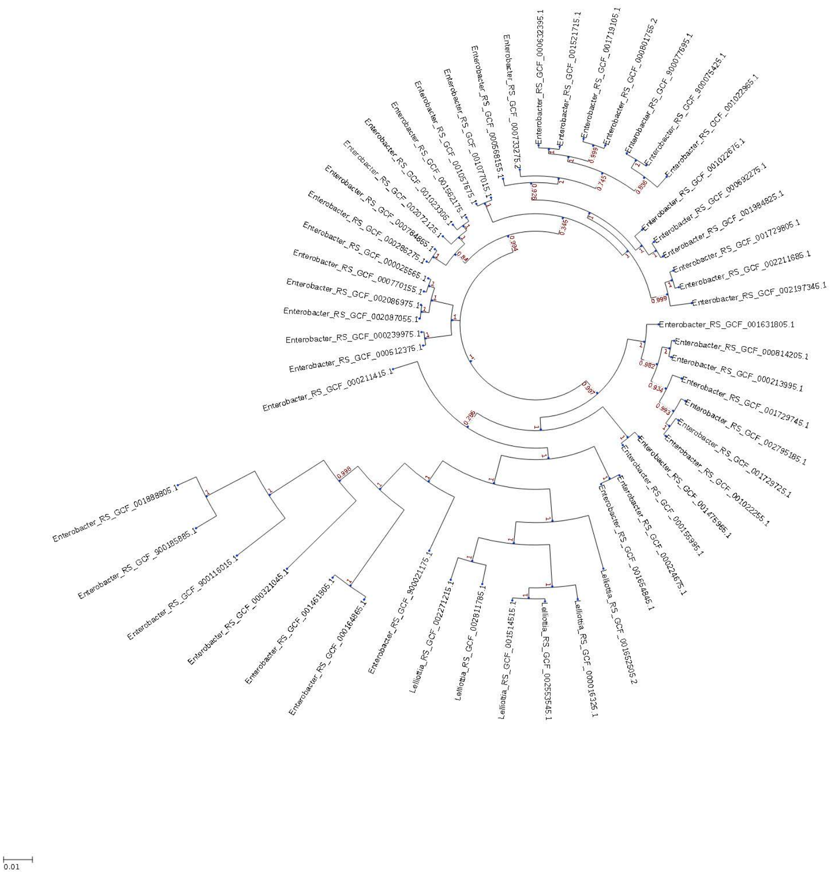

Supplemental figure 11. (A) MDS plot of *Lelliottia* and *Enterobacter* in methods OrthoANIu and HCRlast. (B) Phylogenetic tree of *Lelliottia* and *Enterobacter.*

(A)

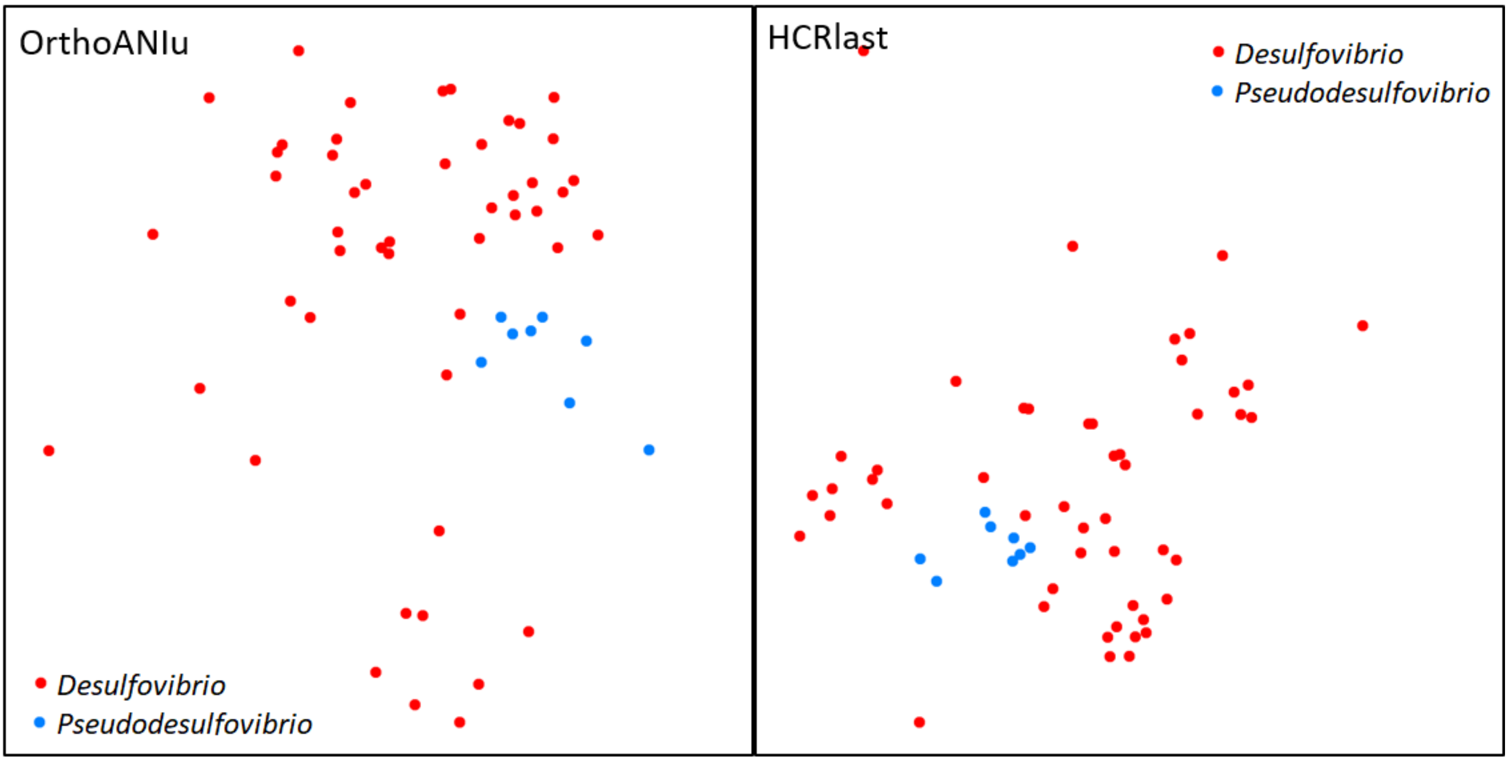

(B)

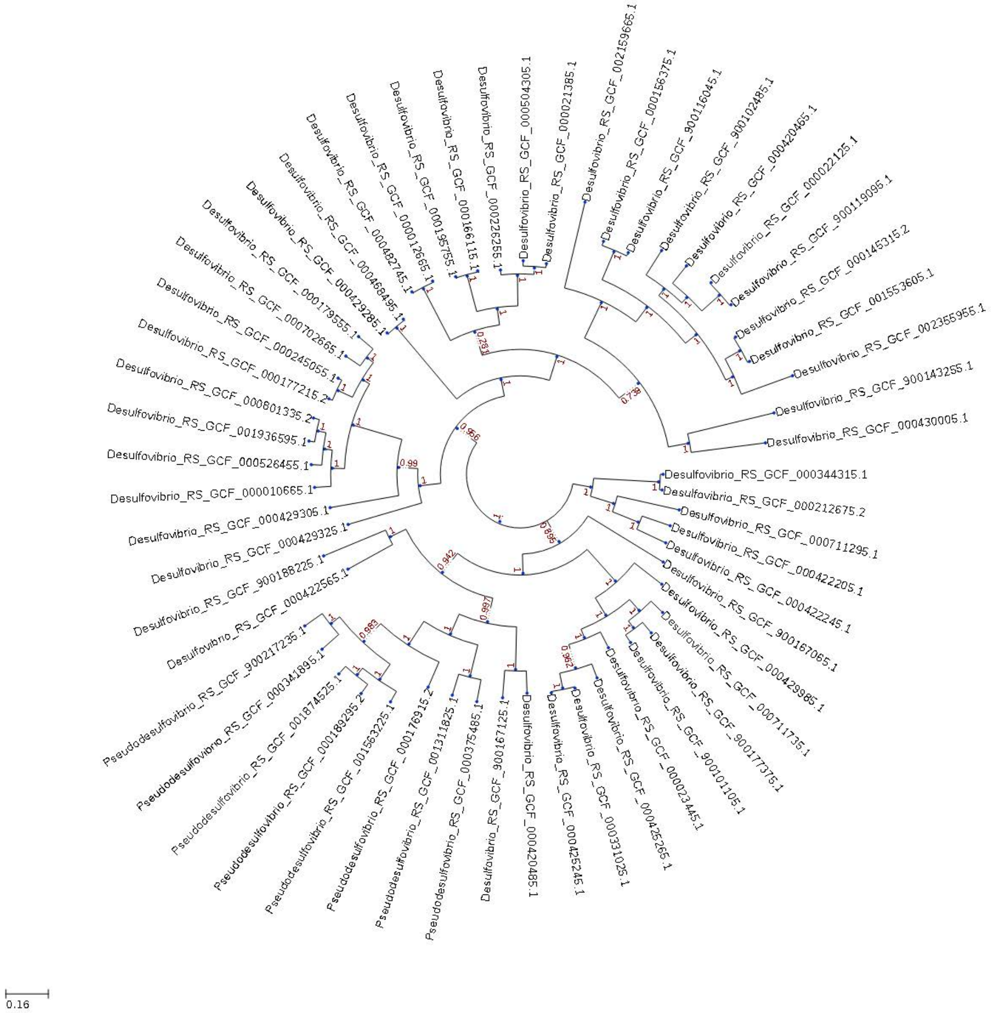

Supplemental figure 12. (A) MDS plot of *Pseudodesulfovibrio* and *Desulfovibrio* in methods OrthoANIu and HCRlast. (B) Phylogenetic tree of *Pseudodesulfovibrio* and *Desulfovibrio.*

(A)

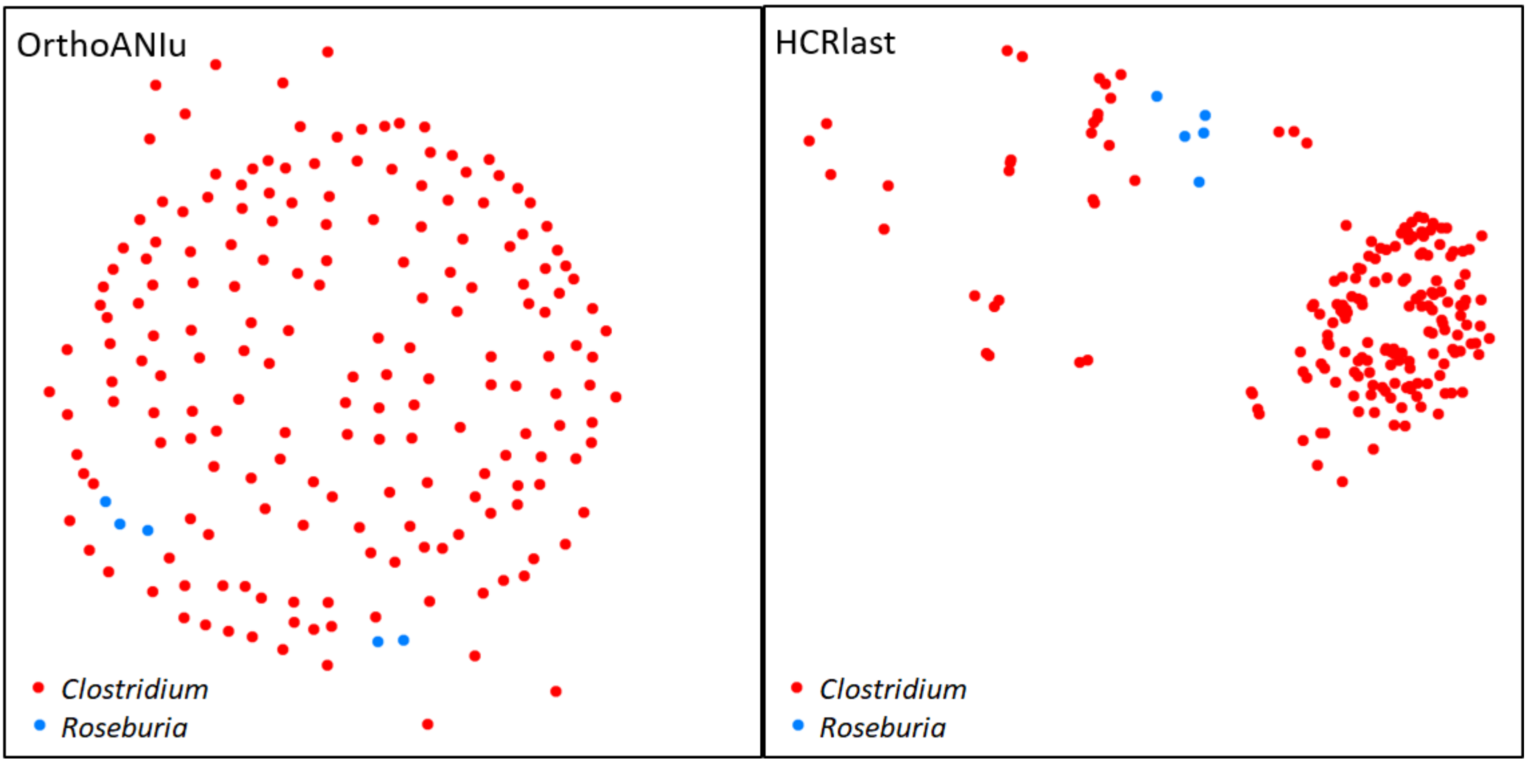

(B)

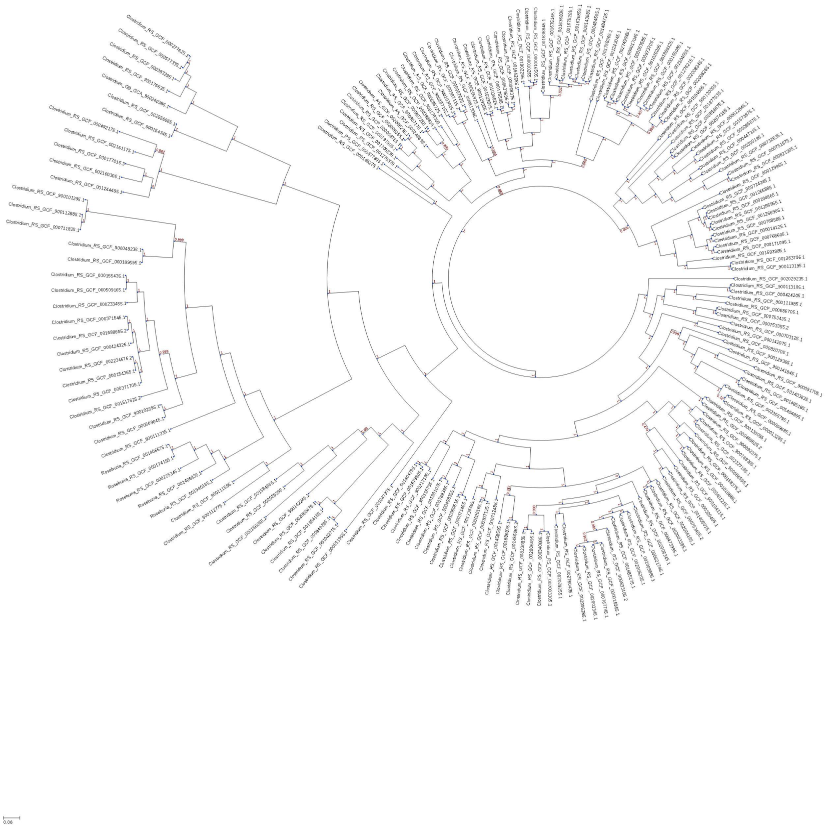

Supplemental figure 13. (A) MDS plot of *Roseburia* and *Clostridium* in methods OrthoANIu and HCRlast. (B) Phylogenetic tree of *Roseburia* and *Clostridium.*

(A)

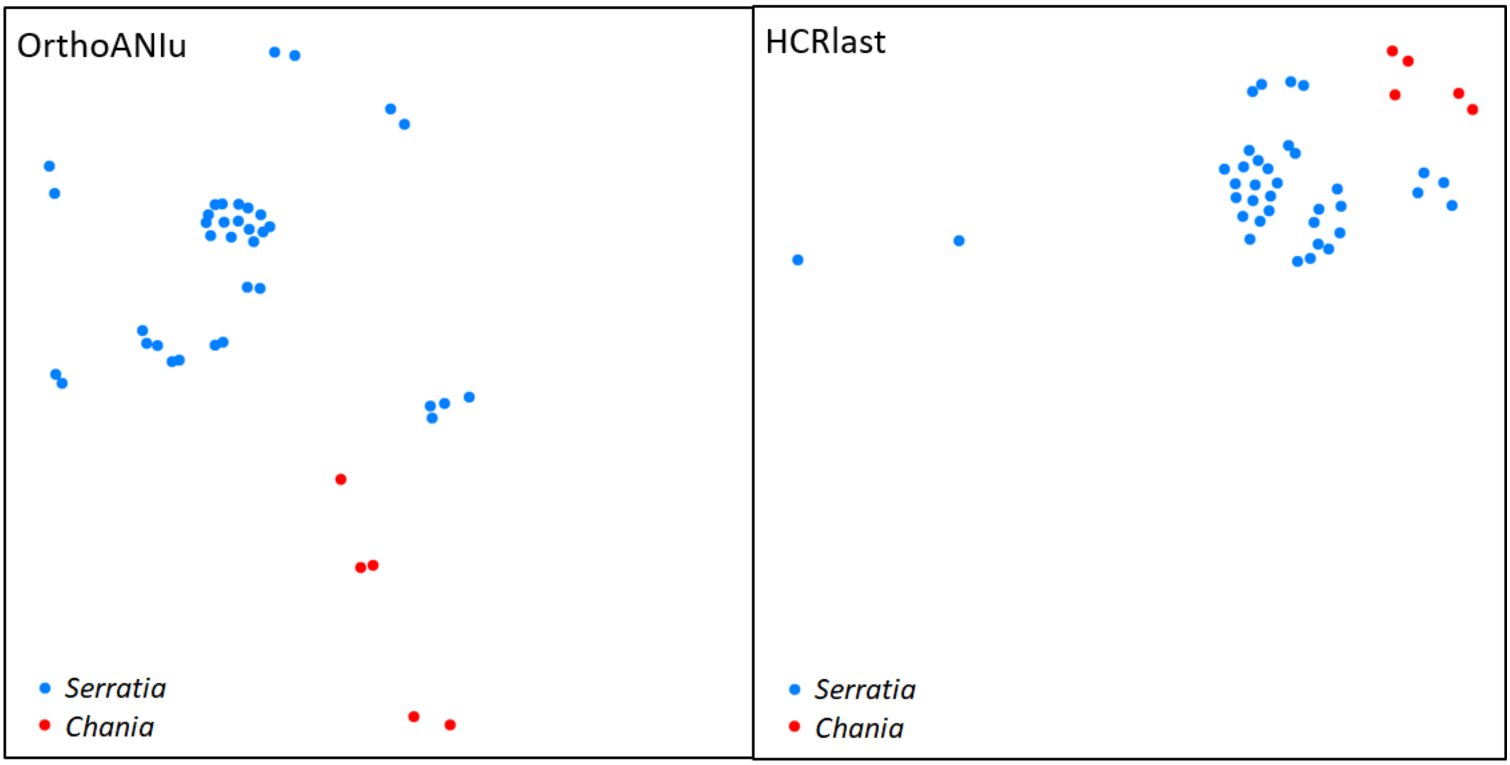

(B)

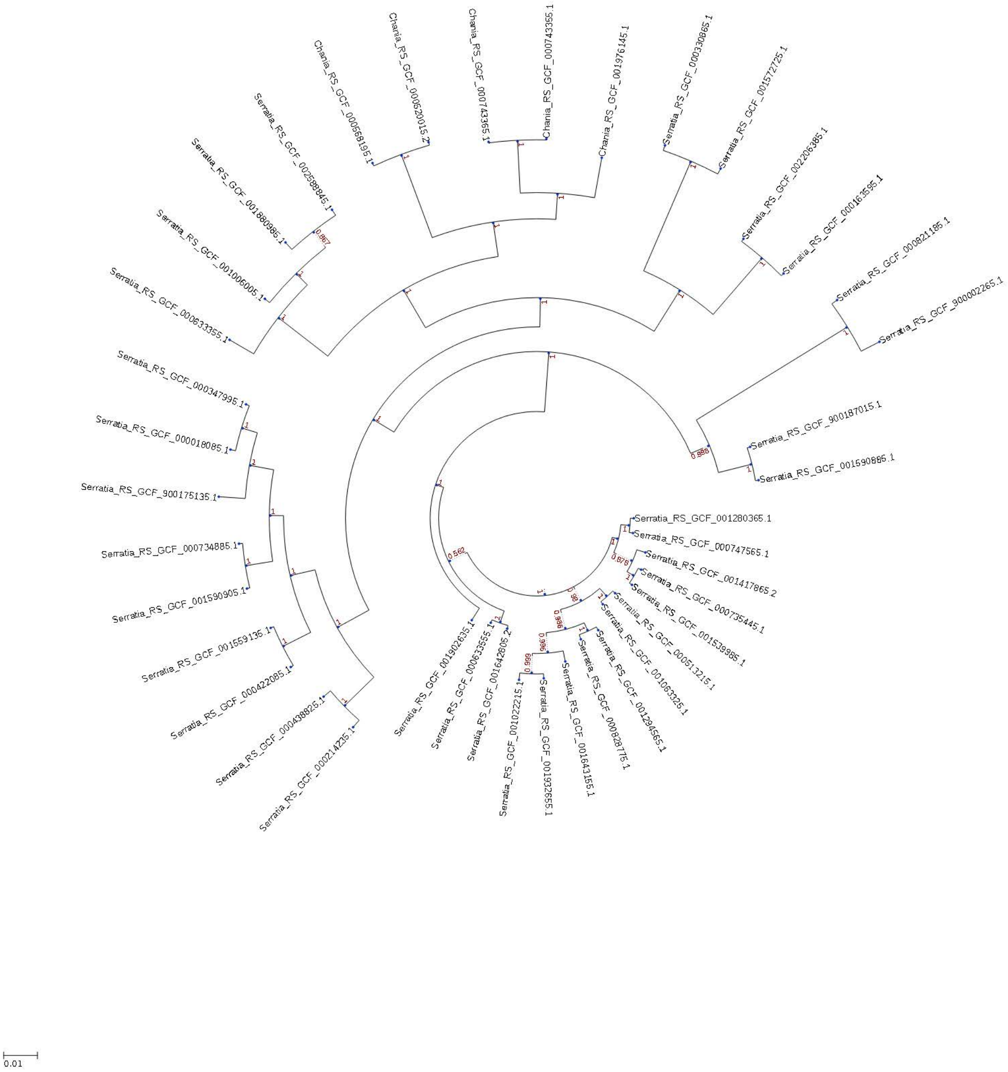

Supplemental figure 14. (A) MDS plot of *Serratia* and *Chania* in methods OrthoANIu and HCRlast. (B) Phylogenetic tree of *Serratia* and *Chania.*

(A)

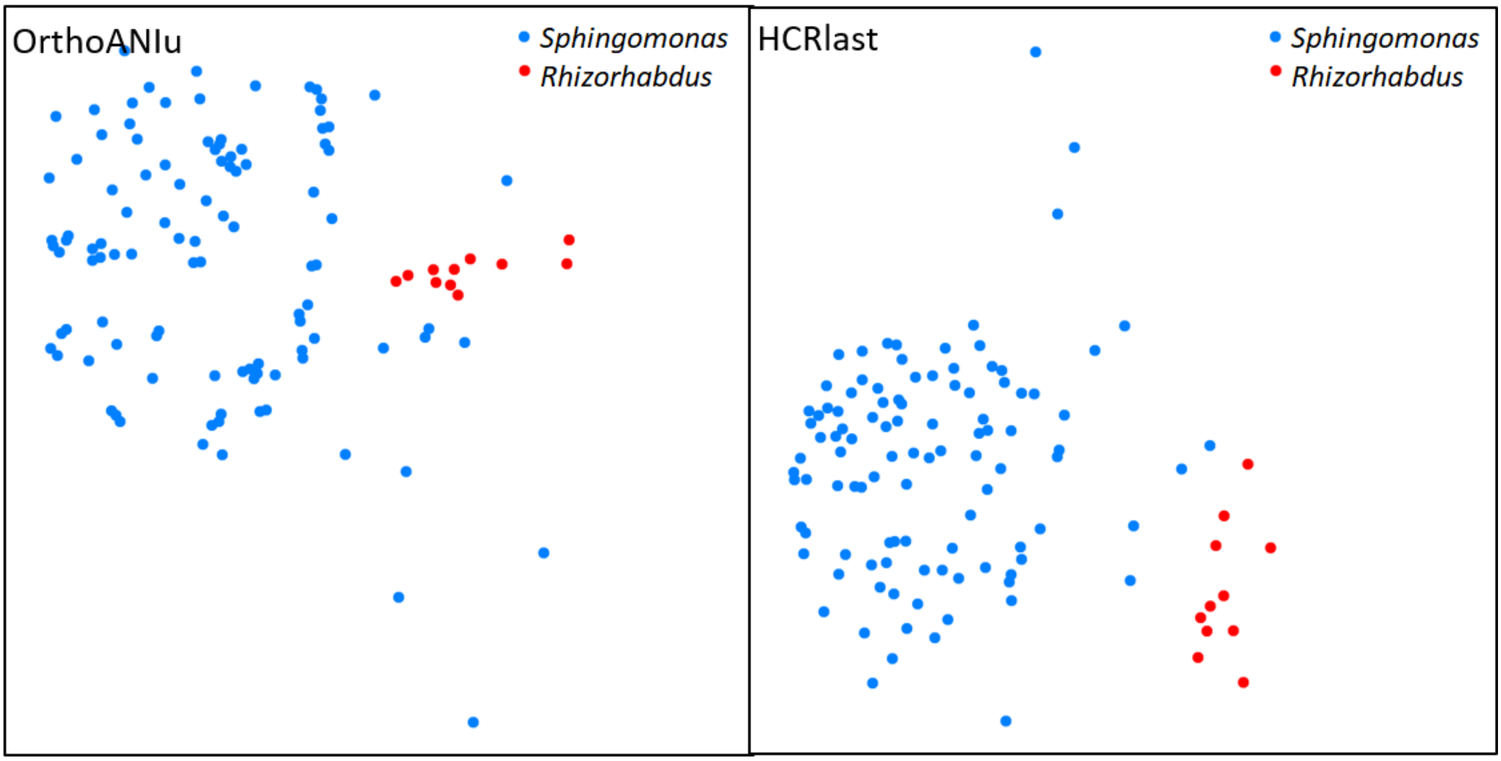

(B)

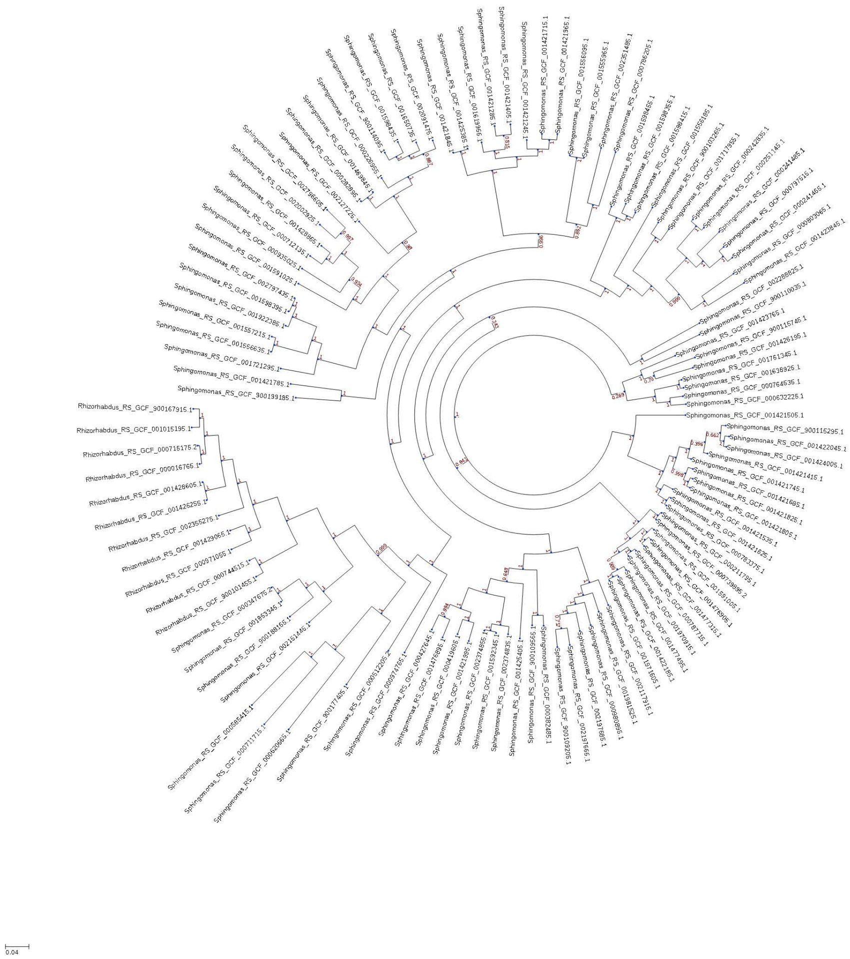

Supplemental figure 15. (A) MDS plot of *Sphingomonas* and *Rhizorhabdus* in methods OrthoANIu and HCRlast. (B) Phylogenetic tree of *Sphingomonas* and *Rhizorhabdus.*

(A)

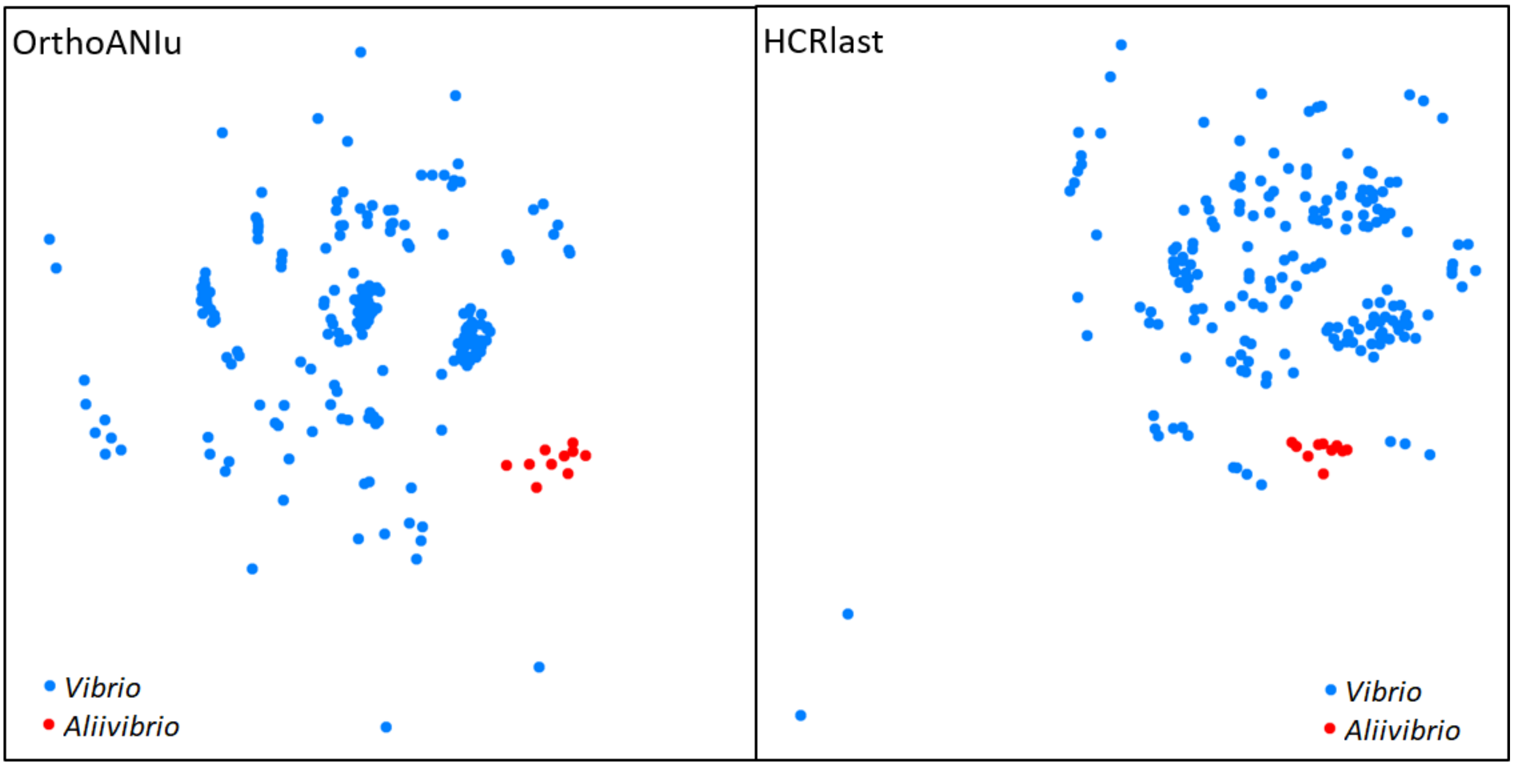

(B)

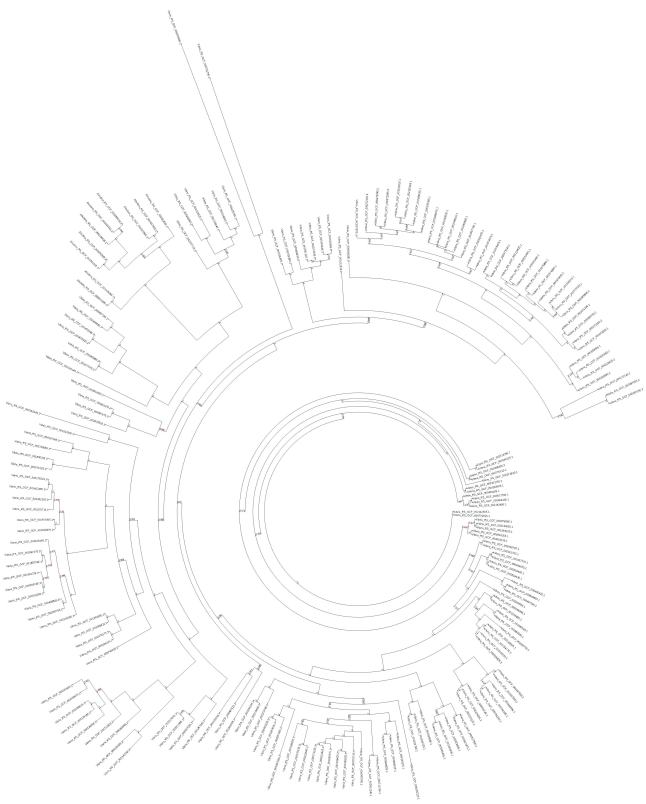

Supplemental figure 16. (A) MDS plot of *Vibrio* and *Aliivibrio* in methods OrthoANIu and HCRlast. (B) Phylogenetic tree of *Vibrio* and *Aliivibrio.*

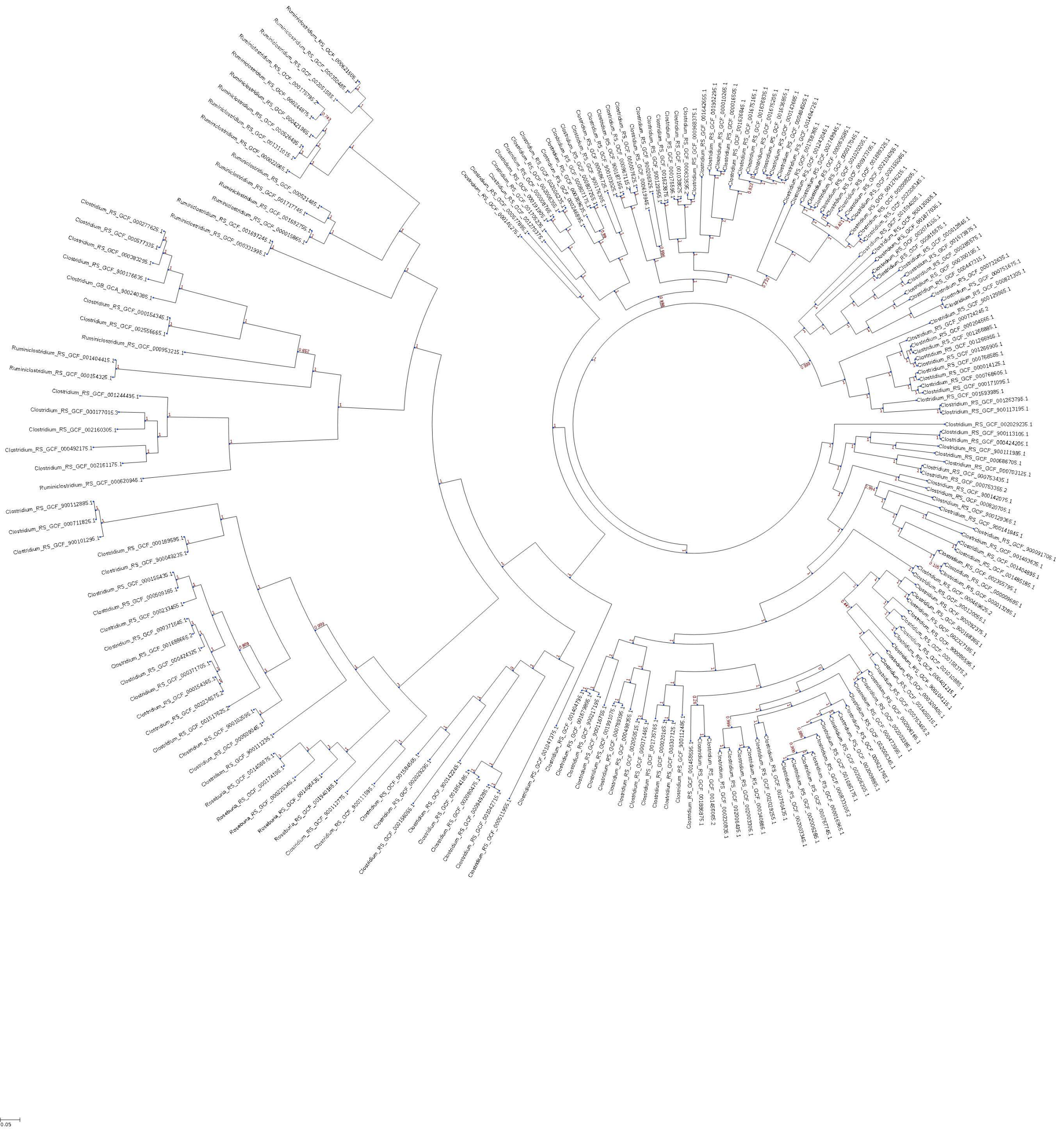

Supplemental figure 17. Phylogenetic tree of *Clostridium* and *Ruminiclostridium.*

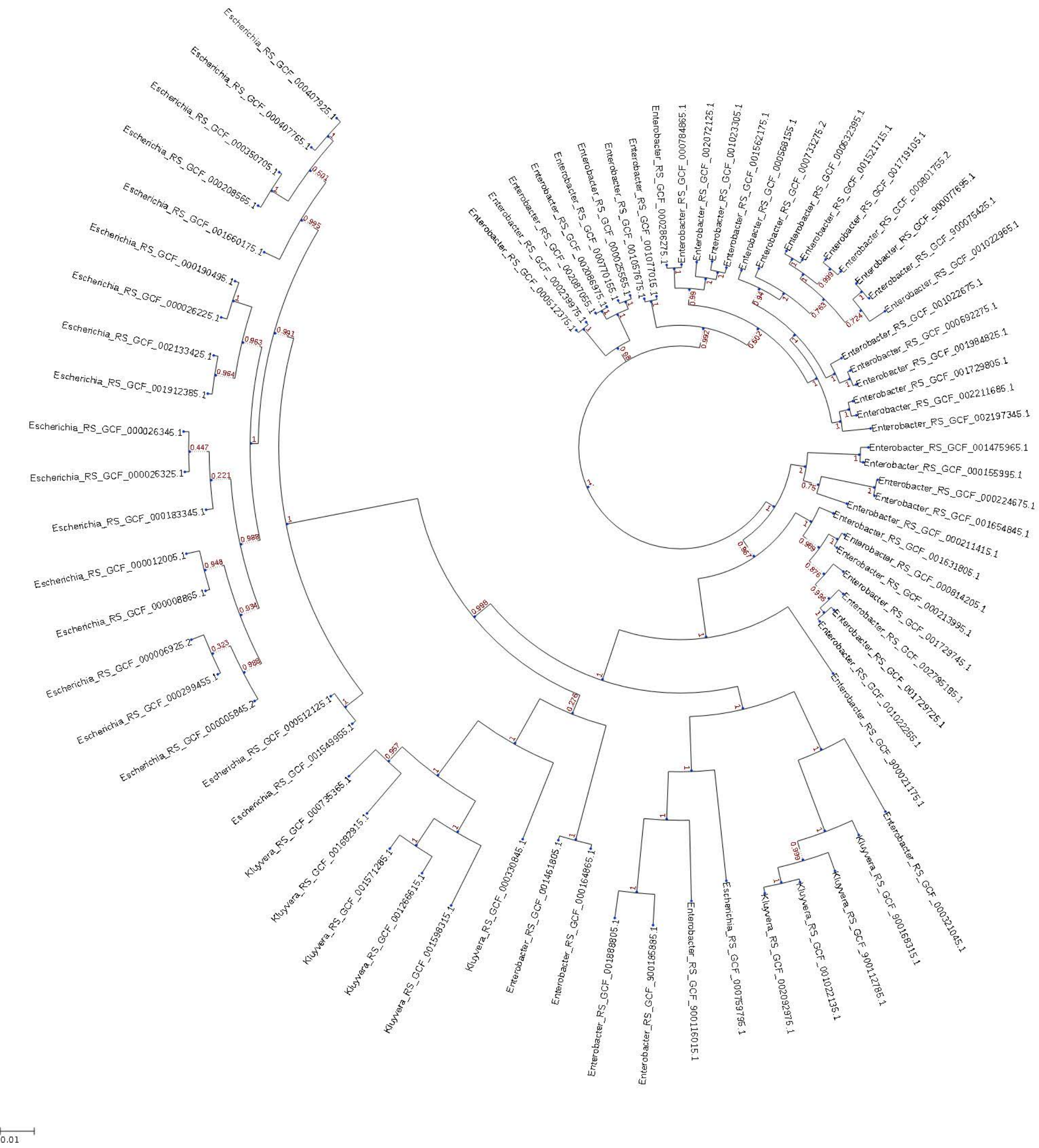

Supplemental figure 18. Phylogenetic tree of *Kluyvera, Enterobacter* and *Escherichia.*

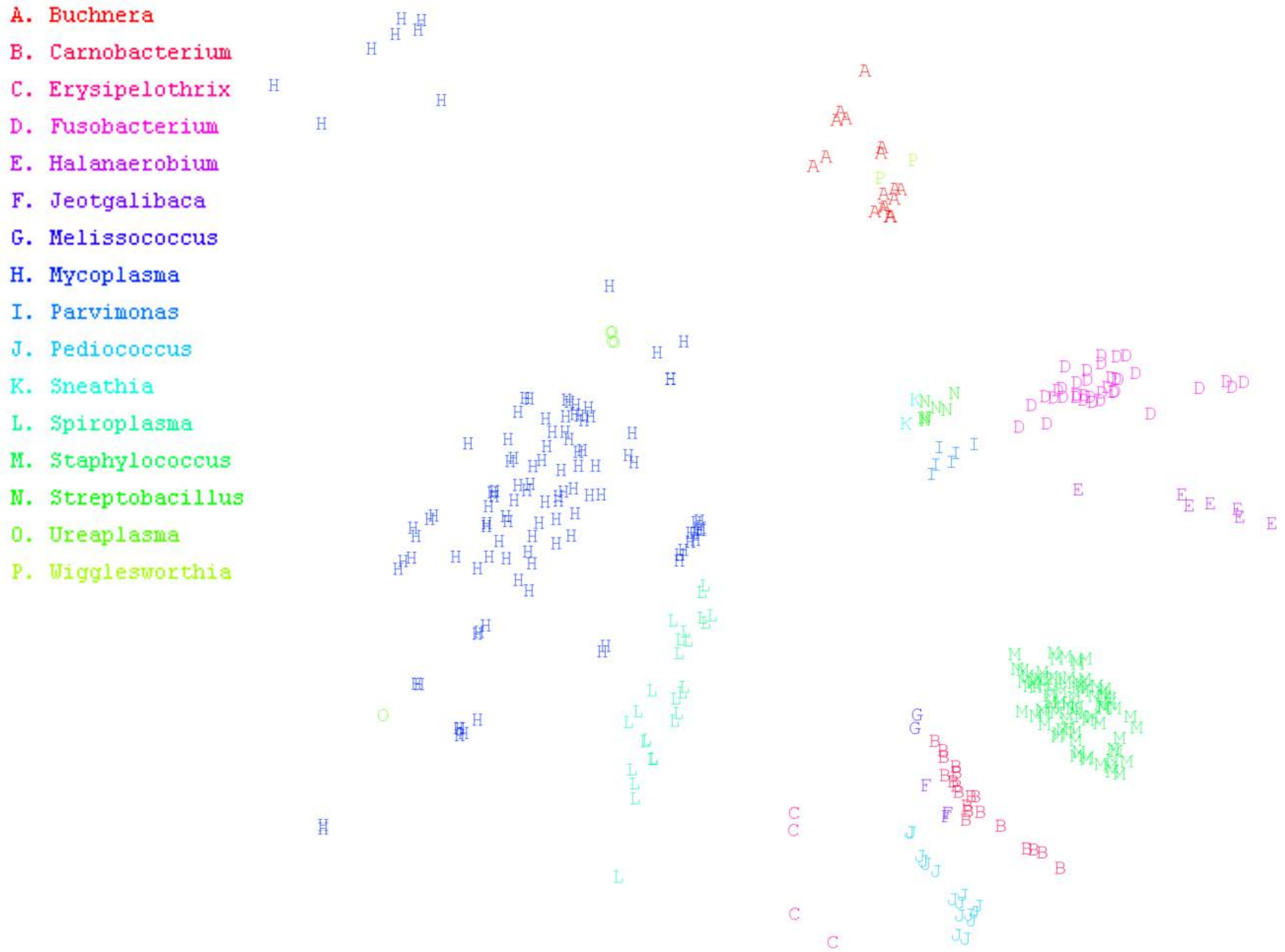

Supplemental figure 19. Multidimensional scaling ordination plot based on the homologous distances among microorganisms, including *Buchnera, Carnobacterium, Erysipelothrix, Fusobacterium, Halanaerobium, Jeotgalibaca, Melissococcus, Mycoplasma, Parvimonas, Pediococcus, Sneathia, Spiroplasma, Staphylococcus, Streptobacillus, Ureaplasma, Wigglesworthia*. Different colors represent different phyla.
