# Supplementary material for: A New Genome-to-Genome Comparison Approach for Large-Scale Revisiting of Current Microbial Taxonomy
Source: Microorganisms. 2019 Jun 3;7(6):161. doi: 10.3390/microorganisms7060161 (PMC6616886; doi:10.3390/microorganisms7060161)
Supplement: Supplementary file 1 [file microorganisms-07-00161-s001.zip › supplementary_files/Supplemental_TableS1.pdf]

Table S1. Reference data set, 7861 microbial genomes, downloaded from the National Center for Biotechnology Information.

ID	Species name	File name
1	Saccharomyces cerevisiae	GCA_000146045.2_R64_genomic.fna
2	Eremothecium gossypii	GCA_000091025.4_ASM9102v4_genomic.fna
3	Kluyveromyces lactis	GCA_000002515.1_ASM251v1_genomic.fna
4	Candida dubliniensis	GCA_000026945.1_ASM2694v1_genomic.fna
5	Botrytis cinerea	GCA_000832945.1_ASM83294v1_genomic.fna
6	Thermothelomyces thermophila	GCA_000226095.1_ASM22609v1_genomic.fna
7	Thielavia terrestris	GCA_000226115.1_ASM22611v1_genomic.fna
8	Kluyveromyces marxianus	GCA_001417885.1_Kmar_1.0_genomic.fna
9	Sporisorium scitamineum	GCA_001010845.1_ASM101084v1_genomic.fna
10	Talaromyces pinophilus	GCA_001571465.2_ASM157146v2_genomic.fna
11	Sugiyamaella lignohabitans	GCA_001640025.2_ASM164002v2_genomic.fna
12	Ustilago bromivora	GCA_900080155.1_UBRO_v3_genomic.fna
13	Zygosaccharomyces parabailii	GCA_001984395.2_ASM198439v2_genomic.fna
14	Saccharomycopsis fibuligera	GCA_001936155.1_KPH12_genomic.fna
15	Malassezia sympodialis	GCA_900149135.1_Msy_KS292_June2015_genomic.fna
16	Kluyveromyces marxianus	GCA_001417835.1_KM1777_03_genomic.fna
17	Verticillium dahliae	GCA_000400815.2_VDAG_JR2v.4.0_genomic.fna
18	Yarrowia lipolytica	GCA_000613145.1_YALI0_genomic.fna
19	Kluyveromyces lactis	GCA_002151565.1_ASM215156v1_genomic.fna
20	Eremothecium gossypii	GCA_000968835.1_ASM96883v1_genomic.fna
21	Saccharomyces cerevisiae	GCA_001051215.1_ASM105121v1_genomic.fna
22	Yarrowia lipolytica	GCA_001761485.1_ASM176148v1_genomic.fna
23	Verticillium dahliae	GCA_000952015.1_ASM95201v1_genomic.fna
24	Kluyveromyces marxianus	GCA_001854445.1_ASM185444v1_genomic.fna
25	Malassezia sympodialis	GCA_900149145.1_Msy_ATCC_42132_Feb2016_genomic.fna
26	Malassezia sympodialis	GCA_900149155.1_Msy_KS024_June2015_genomic.fna
27	Malassezia sympodialis	GCA_900149165.1_Msy_KS327_June2015_genomic.fna
28	Malassezia sympodialis	GCA_900149175.1_Msy_KS004_June2015_genomic.fna
29	Methanococcus maripaludis	GCA_000017225.1_ASM1722v1_genomic.fna
30	Sulfolobus islandicus	GCA_000022385.1_ASM2238v1_genomic.fna
31	Methanococcus voltae	GCA_000006175.2_ASM617v2_genomic.fna

32	Methanobrevibacter smithii	GCA_000016525.1_ASM1652v1_genomic.fna
33	Sulfolobus solfataricus	GCA_900079115.1_SSOP1_genomic.fna
34	Methanocaldococcus jannaschii	GCA_000091665.1_ASM9166v1_genomic.fna
35	Methanosarcina barkeri	GCA_000970025.1_ASM97002v1_genomic.fna
36	Archaeoglobus fulgidus	GCA_000008665.1_ASM866v1_genomic.fna
37	Thermoplasma acidophilum	GCA_000195915.1_ASM19591v1_genomic.fna
38	Pyrobaculum aerophilum	GCA_000007225.1_ASM722v1_genomic.fna
39	Pyrococcus abyssi	GCA_000195935.2_ASM19593v2_genomic.fna
40	Thermoplasma volcanium	GCA_000011185.1_ASM1118v1_genomic.fna
41	Pyrococcus horikoshii	GCA_000011105.1_ASM1110v1_genomic.fna
42	Aeropyrum pernix	GCA_000011125.1_ASM1112v1_genomic.fna
43	Halobacterium salinarum	GCA_000006805.1_ASM680v1_genomic.fna
44	Ferroplasma acidarmanus	GCA_000152265.2_ASM15226v2_genomic.fna
45	Sulfolobus tokodaii	GCA_000011205.1_ASM1120v1_genomic.fna
46	Methanosarcina mazei	GCA_000970205.1_ASM97020v1_genomic.fna
47	Pyrococcus furiosus	GCA_000007305.1_ASM730v1_genomic.fna
48	Methanothermobacter thermautotrophicus	GCA_000008645.1_ASM864v1_genomic.fna
49	Methanosarcina acetivorans	GCA_000007345.1_ASM734v1_genomic.fna
50	Methanopyrus kandleri	GCA_000007185.1_ASM718v1_genomic.fna
51	Haloarcula marismortui	GCA_000011085.1_ASM1108v1_genomic.fna
52	Hyperthermus butylicus	GCA_000015145.1_ASM1514v1_genomic.fna
53	Nanoarchaeum equitans	GCA_000008085.1_ASM808v1_genomic.fna
54	Haloferax volcanii	GCA_000025685.1_ASM2568v1_genomic.fna
55	Methanospirillum hungatei	GCA_000013445.1_ASM1344v1_genomic.fna
56	Thermococcus kodakarensis	GCA_000009965.1_ASM996v1_genomic.fna
57	Halorubrum lacusprofundi	GCA_000022205.1_ASM2220v1_genomic.fna
58	Pyrolobus fumarii	GCA_000223395.1_ASM22339v1_genomic.fna
59	Ignicoccus hospitalis	GCA_000017945.1_ASM1794v1_genomic.fna
60	Sulfolobus acidocaldarius	GCA_000012285.1_ASM1228v1_genomic.fna
61	Methanosphaera stadtmanae	GCA_000012545.1_ASM1254v1_genomic.fna
62	Pyrobaculum arsenaticum	GCA_000016385.1_ASM1638v1_genomic.fna
63	Pyrobaculum neutrophilum	GCA_000019805.1_ASM1980v1_genomic.fna
64	Natronomonas pharaonis	GCA_000026045.1_ASM2604v1_genomic.fna
65	Methanosaeta thermophila	GCA_000014945.1_ASM1494v1_genomic.fna

66	Thermofilum pendens	GCA_000015225.1_ASM1522v1_genomic.fna
67	Pyrobaculum islandicum	GCA_000015205.1_ASM1520v1_genomic.fna
68	Haloquadratum walsbyi	GCA_000237865.1_ASM23786v1_genomic.fna
69	Caldivirga maquilingensis	GCA_000018305.1_ASM1830v1_genomic.fna
70	Metallosphaera sedula	GCA_000016605.1_ASM1660v1_genomic.fna
71	Staphylothermus marinus	GCA_000015945.1_ASM1594v1_genomic.fna
72	Methanococcus vannielii	GCA_000017165.1_ASM1716v1_genomic.fna
73	Methanocorpusculum labreanum	GCA_000015765.1_ASM1576v1_genomic.fna
74	Pyrobaculum calidifontis	GCA_000015805.1_ASM1580v1_genomic.fna
75	Methanoregula boonei	GCA_000017625.1_ASM1762v1_genomic.fna
76	Methanococcus aeolicus	GCA_000017185.1_ASM1718v1_genomic.fna
77	Nitrosopumilus maritimus	GCA_000018465.1_ASM1846v1_genomic.fna
78	Aciduliprofundum boonei	GCA_000025665.1_ASM2566v1_genomic.fna
79	Thermococcus barophilus	GCA_000151105.2_ASM15110v2_genomic.fna
80	Methanocella arvoryzae	GCA_000063445.1_ASM6344v1_genomic.fna
81	Thermococcus onnurineus	GCA_000018365.1_ASM1836v1_genomic.fna
82	Methanococcoides burtonii	GCA_000013725.1_ASM1372v1_genomic.fna
83	Picrophilus torridus	GCA_000008265.1_ASM826v1_genomic.fna
84	Methanoculleus marisnigri	GCA_000015825.1_ASM1582v1_genomic.fna
85	Methanosphaerula palustris	GCA_000021965.1_ASM2196v1_genomic.fna
86	Methanohalophilus mahii	GCA_000025865.1_ASM2586v1_genomic.fna
87	Natrialba magadii	GCA_000025625.1_ASM2562v1_genomic.fna
88	Archaeoglobus profundus	GCA_000025285.1_ASM2528v1_genomic.fna
89	Vulcanisaeta distributa	GCA_000148385.1_ASM14838v1_genomic.fna
90	Methanocaldococcus infernus	GCA_000092305.1_ASM9230v1_genomic.fna
91	Methanocaldococcus fervens	GCA_000023985.1_ASM2398v1_genomic.fna
92	Methanocaldococcus vulcanius	GCA_000024625.1_ASM2462v1_genomic.fna
93	Ignisphaera aggregans	GCA_000145985.1_ASM14598v1_genomic.fna
94	Ferroglobus placidus	GCA_000025505.1_ASM2550v1_genomic.fna
95	Methanothermus fervidus	GCA_000166095.1_ASM16609v1_genomic.fna
96	Thermococcus gammatolerans	GCA_000022365.1_ASM2236v1_genomic.fna
97	Staphylothermus hellenicus	GCA_000092465.1_ASM9246v1_genomic.fna
98	Methanobrevibacter ruminantium	GCA_000024185.1_ASM2418v1_genomic.fna
99	Thermococcus sibiricus	GCA_000022545.1_ASM2254v1_genomic.fna

100	Haloarcula hispanica	GCA_000223905.1_ASM22390v1_genomic.fna
101	Thermosphaera aggregans	GCA_000092185.1_ASM9218v1_genomic.fna
102	Acidilobus saccharovorans	GCA_000144915.1_ASM14491v1_genomic.fna
103	Methanosalsum zhilinae	GCA_000217995.1_ASM21799v1_genomic.fna
104	Methanolacinia petrolearia	GCA_000147875.1_ASM14787v1_genomic.fna
105	Methanohalobium evestigatum	GCA_000196655.1_ASM19665v1_genomic.fna
106	Haloterrigena turkmenica	GCA_000025325.1_ASM2532v1_genomic.fna
107	Methanocella paludicola	GCA_000011005.1_ASM1100v1_genomic.fna
108	Methanothermococcus okinawensis	GCA_000179575.2_ASM17957v2_genomic.fna
109	Methanothermobacter marburgensis	GCA_000145295.1_ASM14529v1_genomic.fna
110	Desulfurococcus mucosus	GCA_000186365.1_ASM18636v1_genomic.fna
111	Halalkalicoccus jeotgali	GCA_000196895.1_ASM19689v1_genomic.fna
112	Archaeoglobus veneficus	GCA_000194625.1_ASM19462v1_genomic.fna
113	Methanotorris igneus	GCA_000214415.1_ASM21441v1_genomic.fna
114	halophilic archaeon	GCA_000224475.1_ASM22447v1_genomic.fna
115	Halopiger xanaduensis	GCA_000217715.1_ASM21771v1_genomic.fna
116	Halovivax ruber	GCA_000328525.1_ASM32852v1_genomic.fna
117	Halogeometricum borinquense	GCA_000172995.2_ASM17299v2_genomic.fna
118	Halomicrobium mukohataei	GCA_000023965.1_ASM2396v1_genomic.fna
119	Halorhabdus utahensis	GCA_000023945.1_ASM2394v1_genomic.fna
120	Acidianus hospitalis	GCA_000213215.1_ASM21321v1_genomic.fna
121	Vulcanisaeta moutnovskia	GCA_000190315.1_ASM19031v1_genomic.fna
122	Methanosaeta concilii	GCA_000204415.1_ASM20441v1_genomic.fna
123	Thermoproteus uzoniensis	GCA_000193375.1_ASM19337v1_genomic.fna
124	Pyrococcus yayanosii	GCA_000215995.1_ASM21599v1_genomic.fna
125	Metallosphaera cuprina	GCA_000204925.1_ASM20492v1_genomic.fna
126	Halorhabdus tiamatea	GCA_000470655.1_HATI1_genomic.fna
127	Nitrososphaera viennensis	GCA_000698785.1_ASM69878v1_genomic.fna
128	Thermococcus nautili	GCA_000585495.1_ASM58549v1_genomic.fna
129	Halobiforma lacisalsi	GCA_000226975.3_ASM22697v3_genomic.fna
130	Haloferax mediterranei	GCA_000306765.2_ASM30676v2_genomic.fna
131	Pyrobaculum oguniense	GCA_000247545.1_ASM24754v1_genomic.fna
132	Natronococcus occultus	GCA_000328685.1_ASM32868v1_genomic.fna
133	Natronomonas moolapensis	GCA_000591055.1_ASM59105v1_genomic.fna

134	Methanomethylovorans hollandica	GCA_000328665.1_ASM32866v1_genomic.fna
135	Natrinema pellirubrum	GCA_000230735.3_ASM23073v3_genomic.fna
136	Natronobacterium gregoryi	GCA_000230715.3_ASM23071v3_genomic.fna
137	Ignicoccus islandicus	GCA_001481685.1_ASM148168v1_genomic.fna
138	Methanoregula formicica	GCA_000327485.1_ASM32748v1_genomic.fna
139	Caldisphaera lagunensis	GCA_000317795.1_ASM31779v1_genomic.fna
140	Halostagnicola larsenii	GCA_000517625.1_ASM51762v1_genomic.fna
141	Thermoplasmatales archaeon	GCA_000350305.1_ASM35030v1_genomic.fna
142	Methanocella conradii	GCA_000251105.1_ASM25110v1_genomic.fna
143	Thermoproteus tenax	GCA_000253055.1_ASM25305v1_genomic.fna
144	Methanolobus psychrophilus	GCA_000306725.1_ASM30672v1_genomic.fna
145	Methanosaeta harundinacea	GCA_000235565.1_ASM23556v1_genomic.fna
146	Thermococcus litoralis	GCA_000246985.3_ASM24698v3_genomic.fna
147	Fervidicoccus fontis	GCA_000258425.1_ASM25842v1_genomic.fna
148	Methanoculleus bourgensis	GCA_000304355.2_Mb_MS2_genomic.fna
149	Methanobacterium formicicum	GCA_000762265.1_ASM76226v1_genomic.fna
150	Haloferax gibbonsii	GCA_001190965.1_ASM119096v1_genomic.fna
151	Archaeoglobus sulfaticallidus	GCA_000385565.1_ASM38556v1_genomic.fna
152	Palaeococcus pacificus	GCA_000725425.1_ASM72542v1_genomic.fna
153	Thermogladius calderae	GCA_000264495.1_ASM26449v1_genomic.fna
154	Desulfurococcus amylolyticus	GCA_000020905.1_ASM2090v1_genomic.fna
155	Methanobacterium lacus	GCA_000191585.1_ASM19158v1_genomic.fna
156	Methanobacterium paludis	GCA_000214725.1_ASM21472v1_genomic.fna
157	Thermococcus eurythermalis	GCA_000769655.1_ASM76965v1_genomic.fna
158	Geoglobus acetivorans	GCA_000789255.1_ASM78925v1_genomic.fna
159	Thermofilum carboxyditrophus	GCA_000813245.1_ASM81324v1_genomic.fna
160	Thermococcus guaymasensis	GCA_000816105.1_ASM81610v1_genomic.fna
161	archaeon GW2011_AR10	GCA_000830275.1_ASM83027v1_genomic.fna
162	archaeon GW2011_AR20	GCA_000830315.1_ASM83031v1_genomic.fna
163	Methanosarcina thermophila	GCA_000969885.1_ASM96988v1_genomic.fna
164	Methanosarcina vacuolata	GCA_000969905.1_ASM96990v1_genomic.fna
165	Methanosarcina siciliae	GCA_000970085.1_ASM97008v1_genomic.fna
166	Methanosarcina lacustris	GCA_000970265.1_ASM97026v1_genomic.fna
167	Methanosarcina horonobensis	GCA_000970285.1_ASM97028v1_genomic.fna

168	Thermococcus paralvinellae	GCA_000517445.1_ASM51744v1_genomic.fna
169	Thermococcus cleftensis	GCA_000265525.1_ASM26552v1_genomic.fna
170	Geoglobus ahangari	GCA_001006045.1_ASM100604v1_genomic.fna
171	Halanaeroarchaeum sulfurireducens	GCA_001011115.1_ASM101111v1_genomic.fna
172	Thermococcus celer	GCA_002214365.1_ASM221436v1_genomic.fna
173	Thermococcus peptonophilus	GCA_001592435.1_ASM159243v1_genomic.fna
174	Pyrodictium delaneyi	GCA_001412615.1_ASM141261v1_genomic.fna
175	Thermococcus thioreducens	GCA_002214545.1_ASM221454v1_genomic.fna
176	Methanocaldococcus bathoardescens	GCA_000739065.1_ASM73906v1_genomic.fna
177	Pyrobaculum ferrireducens	GCA_000234805.1_ASM23480v1_genomic.fna
178	Methanobrevibacter millerae	GCA_001477655.1_ASM147765v1_genomic.fna
179	Halobacterium hubeiense	GCA_001488575.1_Halobacterium_hubeiense_JI20-1_genomic.fna
180	methanogenic archaeon	GCA_001560915.1_ASM156091v1_genomic.fna
181	Methanobrevibacter olleyae	GCA_001563245.1_ASM156324v1_genomic.fna
182	Thermococcus chitonophagus	GCA_002214605.1_ASM221460v1_genomic.fna
183	Thermococcus piezophilus	GCA_001647085.1_ASM164708v1_genomic.fna
184	Nanohaloarchaea archaeon	GCA_001761425.1_ASM176142v1_genomic.fna
185	Halopenitus persicus	GCA_002355635.1_ASM235563v1_genomic.fna
186	Methanohalophilus halophilus	GCA_001889405.1_ASM188940v1_genomic.fna
187	Methanobacterium congolense	GCA_900095295.1_MCBB_genomic.fna
188	Halodesulfurarchaeum formicicum	GCA_001886955.1_ASM188695v1_genomic.fna
189	Pyrococcus kukulkanii	GCA_001577775.1_ASM157777v1_genomic.fna
190	Methanothermobacter wolfeii	GCA_900095815.1_SIV6_genomic.fna
191	Thermofilum adornatus	GCA_000446015.1_ASM44601v1_genomic.fna
192	Cuniculiplasma divulgatum	GCA_900090055.1_C.divulgatum_PM4_genomic.fna
193	Haloterrigena daqingensis	GCA_001971705.1_ASM197170v1_genomic.fna
194	Thermofilum uzonense	GCA_000993805.1_ASM99380v1_genomic.fna
195	Ferroplasma acidiphilum	GCA_002078355.1_ASM207835v1_genomic.fna
196	Acidianus manzaensis	GCA_002116695.1_ASM211669v1_genomic.fna
197	Natrialbaceae archaeon	GCA_002156705.1_ASM215670v1_genomic.fna
198	Thermococcus gorgonarius	GCA_002214385.1_ASM221438v1_genomic.fna
199	Thermococcus barossii	GCA_002214465.1_ASM221446v1_genomic.fna
200	Thermococcus pacificus	GCA_002214485.1_ASM221448v1_genomic.fna
201	Thermococcus siculi	GCA_002214505.1_ASM221450v1_genomic.fna

202	Thermococcus radiotolerans	GCA_002214565.1_ASM221456v1_genomic.fna
203	Thermococcus profundus	GCA_002214585.1_ASM221458v1_genomic.fna
204	Halorubrum trapanicum	GCA_002355655.1_ASM235565v1_genomic.fna
205	Cuniculiplasma divulgatum	GCA_900083515.1_ASM90008351v1_genomic.fna
206	Halodesulfurarchaeum formicicum	GCA_001767315.1_ASM176731v1_genomic.fna
207	Thermococcus chitonophagus	GCA_900012635.1_Pyrococcus_chitonophagus_genome_sequence_genomic.fna
208	Halanaeroarchaeum sulfurireducens	GCA_001305655.1_ASM130565v1_genomic.fna
209	Methanosarcina siciliae	GCA_000970145.1_ASM97014v1_genomic.fna
210	Methanosarcina thermophila	GCA_000969925.1_ASM96992v1_genomic.fna
211	Methanococcoides methylutens	GCA_000970325.1_ASM97032v1_genomic.fna
212	Desulfurococcus amylolyticus	GCA_000231015.3_ASM23101v3_genomic.fna
213	Aeropyrum camini	GCA_000591035.1_ASM59103v1_genomic.fna
214	Methanoculleus bourgensis	GCA_900079125.1_MBBA_genomic.fna
215	Haloarcula hispanica	GCA_000504565.2_ASM50456v2_genomic.fna
216	Thermococcus barophilus	GCA_001433455.1_ASM143345v1_genomic.fna
217	Haloquadratum walsbyi	GCA_000009185.1_ASM918v1_genomic.fna
218	Metallosphaera sedula	GCA_001266655.1_ASM126665v1_genomic.fna
219	Sulfolobus acidocaldarius	GCA_000338775.1_ASM33877v1_genomic.fna
220	Methanosarcina mazei	GCA_000007065.1_ASM706v1_genomic.fna
221	Pyrococcus furiosus	GCA_000275605.1_ASM27560v1_genomic.fna
222	Halobacterium salinarum	GCA_000069025.1_ASM6902v1_genomic.fna
223	Archaeoglobus fulgidus	GCA_000734035.1_ASM73403v1_genomic.fna
224	Methanosarcina barkeri	GCA_000970305.1_ASM97030v1_genomic.fna
225	Sulfolobus solfataricus	GCA_000007005.1_ASM700v1_genomic.fna
226	Sulfolobus islandicus	GCA_000022405.1_ASM2240v1_genomic.fna
227	Methanococcus maripaludis	GCA_000220645.1_ASM22064v1_genomic.fna
228	Methanococcus maripaludis	GCA_000011585.1_ASM1158v1_genomic.fna
229	Sulfolobus islandicus	GCA_000022425.1_ASM2242v1_genomic.fna
230	Sulfolobus solfataricus	GCA_000024745.1_ASM2474v1_genomic.fna
231	Methanosarcina barkeri	GCA_000195895.1_ASM19589v1_genomic.fna
232	Methanosarcina mazei	GCA_000970165.1_ASM97016v1_genomic.fna
233	Sulfolobus acidocaldarius	GCA_000340315.1_ASM34031v1_genomic.fna
234	Metallosphaera sedula	GCA_001266675.1_ASM126667v1_genomic.fna
235	Methanobacterium formicicum	GCA_000953115.1_DSM1535_genomic.fna

236	Methanosarcina siciliae	GCA_000970125.1_ASM97012v1_genomic.fna
237	Methanobacterium formicicum	GCA_001458655.1_Mb9_genomic.fna
238	Metallosphaera sedula	GCA_001266695.1_ASM126669v1_genomic.fna
239	Sulfolobus acidocaldarius	GCA_002215405.1_ASM221540v1_genomic.fna
240	Methanosarcina mazei	GCA_000970185.1_ASM97018v1_genomic.fna
241	Methanosarcina barkeri	GCA_000969985.1_ASM96998v1_genomic.fna
242	Sulfolobus solfataricus	GCA_000968355.1_ASM96835v1_genomic.fna
243	Sulfolobus islandicus	GCA_000022445.1_ASM2244v1_genomic.fna
244	Methanococcus maripaludis	GCA_000016125.1_ASM1612v1_genomic.fna
245	Methanococcus maripaludis	GCA_000018485.1_ASM1848v1_genomic.fna
246	Sulfolobus islandicus	GCA_000022465.1_ASM2246v1_genomic.fna
247	Sulfolobus solfataricus	GCA_000968395.1_ASM96839v1_genomic.fna
248	Methanosarcina barkeri	GCA_000970065.1_ASM97006v1_genomic.fna
249	Methanosarcina mazei	GCA_000970225.1_ASM97022v1_genomic.fna
250	Sulfolobus acidocaldarius	GCA_002215445.1_ASM221544v1_genomic.fna
251	Metallosphaera sedula	GCA_001266715.1_ASM126671v1_genomic.fna
252	Metallosphaera sedula	GCA_001266735.1_ASM126673v1_genomic.fna
253	Sulfolobus acidocaldarius	GCA_002215485.1_ASM221548v1_genomic.fna
254	Methanosarcina mazei	GCA_000970245.1_ASM97024v1_genomic.fna
255	Methanosarcina barkeri	GCA_001027005.1_ASM102700v1_genomic.fna
256	Sulfolobus solfataricus	GCA_000968435.1_ASM96843v1_genomic.fna
257	Sulfolobus islandicus	GCA_000022485.1_ASM2248v1_genomic.fna
258	Sulfolobus islandicus	GCA_000189555.1_ASM18955v1_genomic.fna
259	Sulfolobus acidocaldarius	GCA_002215525.1_ASM221552v1_genomic.fna
260	Sulfolobus acidocaldarius	GCA_002215565.1_ASM221556v1_genomic.fna
261	Sulfolobus islandicus	GCA_000189575.1_ASM18957v1_genomic.fna
262	Sulfolobus islandicus	GCA_000364745.1_ASM36474v1_genomic.fna
263	Sulfolobus acidocaldarius	GCA_000508305.1_ASM50830v1_genomic.fna
264	Methanosarcina mazei	GCA_000341715.1_ASM34171v1_genomic.fna
265	Campylobacter jejuni	GCA_000009085.1_ASM908v1_genomic.fna
266	Pseudomonas fluorescens	GCA_000237065.1_ASM23706v1_genomic.fna
267	Salmonella enterica	GCA_000195995.1_ASM19599v1_genomic.fna
268	Xanthomonas campestris	GCA_000007145.1_ASM714v1_genomic.fna
269	Yersinia pestis	GCA_000009065.1_ASM906v1_genomic.fna

270	Staphylococcus aureus	GCA_000013425.1_ASM1342v1_genomic.fna
271	Staphylococcus epidermidis	GCA_000007645.1_ASM764v1_genomic.fna
272	Lactococcus lactis	GCA_000006865.1_ASM686v1_genomic.fna
273	Bacillus cereus	GCA_000007825.1_ASM782v1_genomic.fna
274	Clostridium perfringens	GCA_000013285.1_ASM1328v1_genomic.fna
275	Listeria monocytogenes	GCA_000196035.1_ASM19603v1_genomic.fna
276	Mycobacterium avium	GCA_000007865.1_ASM786v1_genomic.fna
277	Actinobacillus pleuropneumoniae	GCA_000015885.1_ASM1588v1_genomic.fna
278	Tropheryma whipplei	GCA_000007485.1_ASM748v1_genomic.fna
279	Prochlorococcus marinus	GCA_000007925.1_ASM792v1_genomic.fna
280	Haemophilus influenzae	GCA_000027305.1_ASM2730v1_genomic.fna
281	Mycobacterium tuberculosis	GCA_000195955.2_ASM19595v2_genomic.fna
282	Escherichia coli	GCA_000008865.1_ASM886v1_genomic.fna
283	Helicobacter pylori	GCA_000008525.1_ASM852v1_genomic.fna
284	Chlamydophila pneumoniae	GCA_000008745.1_ASM874v1_genomic.fna
285	Buchnera aphidicola	GCA_000007365.1_ASM736v1_genomic.fna
286	Neisseria meningitidis	GCA_000008805.1_ASM880v1_genomic.fna
287	Xylella fastidiosa	GCA_000006725.1_ASM672v1_genomic.fna
288	Streptococcus pyogenes	GCA_000006785.2_ASM678v2_genomic.fna
289	Streptococcus pneumoniae	GCA_000007045.1_ASM704v1_genomic.fna
290	Agrobacterium radiobacter	GCA_000016265.1_ASM1626v1_genomic.fna
291	Xanthomonas axonopodis	GCA_000348585.1_ASM34858v1_genomic.fna
292	Leptospira interrogans	GCA_000092565.1_ASM9256v1_genomic.fna
293	Fusobacterium nucleatum	GCA_000007325.1_ASM732v1_genomic.fna
294	Bacillus anthracis	GCA_000007845.1_ASM784v1_genomic.fna
295	Shigella flexneri	GCA_000006925.2_ASM692v2_genomic.fna
296	Bifidobacterium longum	GCA_000007525.1_ASM752v1_genomic.fna
297	Haemophilus somnus	GCA_000019405.1_ASM1940v1_genomic.fna
298	Pseudomonas syringae	GCA_000012245.1_ASM1224v1_genomic.fna
299	Streptococcus agalactiae	GCA_000007265.1_ASM726v1_genomic.fna
300	Pseudomonas aeruginosa	GCA_000006765.1_ASM676v1_genomic.fna
301	Vibrio vulnificus	GCA_002215135.1_ASM221513v1_genomic.fna
302	Mycoplasma hyopneumoniae	GCA_000008205.1_ASM820v1_genomic.fna
303	Streptococcus suis	GCA_000026745.1_ASM2674v1_genomic.fna

304	Chlorobium phaeobacteroides	GCA_000015125.1_ASM1512v1_genomic.fna
305	Paenarthrobacter aurescens	GCA_000014925.1_ASM1492v1_genomic.fna
306	Acinetobacter baumannii	GCA_000746645.1_ASM74664v1_genomic.fna
307	Anaplasma marginale	GCA_000020305.1_ASM2030v1_genomic.fna
308	Bacillus licheniformis	GCA_000011645.1_ASM1164v1_genomic.fna
309	Bacteroides fragilis	GCA_000009925.1_ASM992v1_genomic.fna
310	Shigella dysenteriae	GCA_000012005.1_ASM1200v1_genomic.fna
311	Legionella pneumophila	GCA_000008485.1_ASM848v1_genomic.fna
312	Shigella sonnei	GCA_000092525.1_ASM9252v1_genomic.fna
313	Streptococcus thermophilus	GCA_000253395.1_ASM25339v1_genomic.fna
314	Wolbachia endosymbiont	GCA_000008385.1_ASM838v1_genomic.fna
315	Shewanella baltica	GCA_000178875.2_ASM17887v2_genomic.fna
316	Synechococcus elongatus	GCA_000010065.1_ASM1006v1_genomic.fna
317	Shewanella putrefaciens	GCA_000016585.1_ASM1658v1_genomic.fna
318	Ehrlichia ruminantium	GCA_000026005.1_ASM2600v1_genomic.fna
319	Lactobacillus reuteri	GCA_000016825.1_ASM1682v1_genomic.fna
320	Bacillus pumilus	GCA_001578205.1_ASM157820v1_genomic.fna
321	Thermus thermophilus	GCA_000091545.1_ASM9154v1_genomic.fna
322	Corynebacterium glutamicum	GCA_000011325.1_ASM1132v1_genomic.fna
323	Chlamydia trachomatis	GCA_000008725.1_ASM872v1_genomic.fna
324	Mycoplasma genitalium	GCA_000027325.1_ASM2732v1_genomic.fna
325	Burkholderia cenocepacia	GCA_000009485.1_ASM948v1_genomic.fna
326	Burkholderia pseudomallei	GCA_000011545.1_ASM1154v1_genomic.fna
327	Burkholderia mallei	GCA_000011705.1_ASM1170v1_genomic.fna
328	Corynebacterium jeikeium	GCA_000006605.1_ASM660v1_genomic.fna
329	Ehrlichia chaffeensis	GCA_000013145.1_ASM1314v1_genomic.fna
330	Nitrosococcus oceani	GCA_000012805.1_ASM1280v1_genomic.fna
331	Bacillus thuringiensis	GCA_000008505.1_ASM850v1_genomic.fna
332	Ralstonia solanacearum	GCA_000009125.1_ASM912v1_genomic.fna
333	Shigella boydii	GCA_000012025.1_ASM1202v1_genomic.fna
334	Mycobacterium ulcerans	GCA_000013925.2_ASM1392v2_genomic.fna
335	Vibrio cholerae	GCA_000006745.1_ASM674v1_genomic.fna
336	Streptococcus salivarius	GCA_000785515.1_ASM78551v1_genomic.fna
337	Rhodopseudomonas palustris	GCA_000013365.1_ASM1336v1_genomic.fna

338	Rhodobacter sphaeroides	GCA_000016405.1_ASM1640v1_genomic.fna
339	Yersinia pseudotuberculosis	GCA_000016945.1_ASM1694v1_genomic.fna
340	Francisella tularensis	GCA_000008985.1_ASM898v1_genomic.fna
341	Lactobacillus delbrueckii	GCA_000056065.1_ASM5606v1_genomic.fna
342	Mycoplasma agalactiae	GCA_000063605.1_ASM6360v1_genomic.fna
343	Leptospira borgpetersenii	GCA_000013945.1_ASM1394v1_genomic.fna
344	Brucella abortus	GCA_000008145.1_ASM814v1_genomic.fna
345	Mycoplasma capricolum	GCA_000012765.1_ASM1276v1_genomic.fna
346	Bartonella bacilliformis	GCA_000015445.1_ASM1544v1_genomic.fna
347	Xanthomonas citri	GCA_002218245.1_ASM221824v1_genomic.fna
348	Campylobacter fetus	GCA_000495505.1_ASM49550v1_genomic.fna
349	Streptococcus mitis	GCA_000027165.1_ASM2716v1_genomic.fna
350	Xanthomonas oryzae	GCA_000019585.2_ASM1958v2_genomic.fna
351	Sodalis glossinidius	GCA_000010085.1_ASM1008v1_genomic.fna
352	Thermotoga neapolitana	GCA_000018945.1_ASM1894v1_genomic.fna
353	Salinispora tropica	GCA_000016425.1_ASM1642v1_genomic.fna
354	Clostridioides difficile	GCA_000009205.1_ASM920v1_genomic.fna
355	Roseobacter denitrificans	GCA_000014045.1_ASM1404v1_genomic.fna
356	Ralstonia eutropha	GCA_000203875.1_ASM20387v1_genomic.fna
357	Aeromonas salmonicida	GCA_000196395.1_ASM19639v1_genomic.fna
358	Desulfitobacterium hafniense	GCA_000021925.1_ASM2192v1_genomic.fna
359	Oenococcus oeni	GCA_000014385.1_ASM1438v1_genomic.fna
360	Coxiella burnetii	GCA_000007765.2_ASM776v2_genomic.fna
361	Lactobacillus casei	GCA_000019245.3_ASM1924v3_genomic.fna
362	Salinispora arenicola	GCA_000018265.1_ASM1826v1_genomic.fna
363	Desulfovibrio vulgaris	GCA_000021385.1_ASM2138v1_genomic.fna
364	Delftia acidovorans	GCA_000018665.1_ASM1866v1_genomic.fna
365	Rickettsia bellii	GCA_000012385.1_ASM1238v1_genomic.fna
366	Bacillus subtilis	GCA_000009045.1_ASM904v1_genomic.fna
367	Ralstonia pickettii	GCA_000020205.1_ASM2020v1_genomic.fna
368	Lactobacillus helveticus	GCA_001308285.1_ASM130828v1_genomic.fna
369	Geobacillus stearothermophilus	GCA_001274575.1_ASM127457v1_genomic.fna
370	Rickettsia rickettsii	GCA_000017445.3_ASM1744v3_genomic.fna
371	Photobacterium damselae	GCA_002142615.1_ASM214261v1_genomic.fna

372	Borrelia afzelii	GCA_000304735.1_ASM30473v1_genomic.fna
373	Bifidobacterium adolescentis	GCA_000010425.1_ASM1042v1_genomic.fna
374	Vibrio harveyi	GCA_000770115.2_ASM77011v2_genomic.fna
375	Saccharopolyspora erythraea	GCA_000062885.1_ASM6288v1_genomic.fna
376	Vibrio parahaemolyticus	GCA_000196095.1_ASM19609v1_genomic.fna
377	Lactococcus garvieae	GCA_000269925.1_ASM26992v1_genomic.fna
378	Methylobacterium extorquens	GCA_000018845.1_ASM1884v1_genomic.fna
379	Methylobacterium radiotolerans	GCA_000019725.1_ASM1972v1_genomic.fna
380	Orientia tsutsugamushi	GCA_000063545.1_ASM6354v1_genomic.fna
381	Lactobacillus fermentum	GCA_000010145.1_ASM1014v1_genomic.fna
382	Clostridium kluyveri	GCA_000016505.1_ASM1650v1_genomic.fna
383	Porphyromonas gingivalis	GCA_000010505.1_ASM1050v1_genomic.fna
384	Gramella forsetii	GCA_000060345.1_ASM6034v1_genomic.fna
385	Ureaplasma parvum	GCA_000019345.1_ASM1934v1_genomic.fna
386	Ureaplasma urealyticum	GCA_000021265.1_ASM2126v1_genomic.fna
387	Mycoplasma mycoides	GCA_000011445.1_ASM1144v1_genomic.fna
388	Burkholderia thailandensis	GCA_000012365.1_ASM1236v1_genomic.fna
389	Phaeobacter gallaeciensis	GCA_000511385.1_ASM51138v1_genomic.fna
390	Octadecabacter antarcticus	GCA_000155675.2_ASM15567v2_genomic.fna
391	Vibrio fischeri	GCA_000011805.1_ASM1180v1_genomic.fna
392	Burkholderia oklahomensis	GCA_000755985.1_ASM75598v1_genomic.fna
393	Clostridium botulinum	GCA_000017045.1_ASM1704v1_genomic.fna
394	Burkholderia multivorans	GCA_000010545.1_ASM1054v1_genomic.fna
395	Ochrobactrum anthropi	GCA_000017405.1_ASM1740v1_genomic.fna
396	Rickettsia prowazekii	GCA_000195735.1_ASM19573v1_genomic.fna
397	Treponema pallidum	GCA_000604125.1_ASM60412v1_genomic.fna
398	Borrelia burgdorferi	GCA_000008685.2_ASM868v2_genomic.fna
399	Rhizobium leguminosarum	GCA_000021345.1_ASM2134v1_genomic.fna
400	Anaeromyxobacter dehalogenans	GCA_000022145.1_ASM2214v1_genomic.fna
401	Clavibacter michiganensis	GCA_000069225.1_ASM6922v1_genomic.fna
402	Leptospira biflexa	GCA_000017685.1_ASM1768v1_genomic.fna
403	Mycobacterium marinum	GCA_000723425.2_E11_genomic.fna
404	Brucella canis	GCA_000018525.1_ASM1852v1_genomic.fna
405	Burkholderia ambifaria	GCA_000019925.1_ASM1992v1_genomic.fna

406	Brucella suis	GCA_000007505.1_ASM750v1_genomic.fna
407	Enterococcus faecalis	GCA_000007785.1_ASM778v1_genomic.fna
408	Bradyrhizobium japonicum	GCA_000284375.1_ASM28437v1_genomic.fna
409	Bifidobacterium dentium	GCA_001042595.1_ASM104259v1_genomic.fna
410	Klebsiella pneumoniae	GCA_000240185.2_ASM24018v2_genomic.fna
411	Gluconacetobacter diazotrophicus	GCA_000021325.1_ASM2132v1_genomic.fna
412	Microcystis aeruginosa	GCA_000010625.1_ASM1062v1_genomic.fna
413	Streptococcus dysgalactiae	GCA_000317855.1_ASM31785v1_genomic.fna
414	Rhizobium etli	GCA_000092045.1_ASM9204v1_genomic.fna
415	Chromohalobacter salexigens	GCA_000055785.1_ASM5578v1_genomic.fna
416	Bartonella tribocorum	GCA_000196435.1_ASM19643v1_genomic.fna
417	Ruminiclostridium thermocellum	GCA_000015865.1_ASM1586v1_genomic.fna
418	Streptomyces clavuligerus	GCA_001693675.1_ASM169367v1_genomic.fna
419	Borreliella garinii	GCA_001922545.1_ASM192254v1_genomic.fna
420	Chlamydia psittaci	GCA_000204255.1_ASM20425v1_genomic.fna
421	Bifidobacterium animalis	GCA_000022965.1_ASM2296v1_genomic.fna
422	Bacillus amyloliquefaciens	GCA_000196735.1_ASM19673v1_genomic.fna
423	Streptococcus mutans	GCA_000007465.2_ASM746v2_genomic.fna
424	Comamonas testosteroni	GCA_000739375.1_ASM73937v1_genomic.fna
425	Enterococcus faecium	GCA_000174395.2_ASM17439v2_genomic.fna
426	Neisseria gonorrhoeae	GCA_000006845.1_ASM684v1_genomic.fna
427	Bacillus mycoides	GCA_000832605.1_ASM83260v1_genomic.fna
428	Yersinia frederiksenii	GCA_000834215.1_ASM83421v1_genomic.fna
429	Yersinia kristensenii	GCA_000834865.1_ASM83486v1_genomic.fna
430	Alteromonas macleodii	GCA_000172635.2_ASM17263v2_genomic.fna
431	Stenotrophomonas maltophilia	GCA_000072485.1_ASM7248v1_genomic.fna
432	Bifidobacterium bifidum	GCA_000165905.1_ASM16590v1_genomic.fna
433	Micrococcus luteus	GCA_000023205.1_ASM2320v1_genomic.fna
434	Streptococcus equi	GCA_000026605.1_ASM2660v1_genomic.fna
435	Zymomonas mobilis	GCA_000218875.1_ASM21887v1_genomic.fna
436	Haemophilus parasuis	GCA_000021885.1_ASM2188v1_genomic.fna
437	Mycobacterium leprae	GCA_000195855.1_ASM19585v1_genomic.fna
438	Acetobacter pasteurianus	GCA_001766235.1_ASM176623v1_genomic.fna
439	Francisella tularensis	GCA_000834965.1_ASM83496v1_genomic.fna

440	Pasteurella multocida	GCA_000754275.1_ASM75427v1_genomic.fna
441	Lactobacillus rhamnosus	GCA_000026505.1_ASM2650v1_genomic.fna
442	Francisella philomiragia	GCA_000019285.1_ASM1928v1_genomic.fna
443	Thermomicrobium roseum	GCA_000021685.1_ASM2168v1_genomic.fna
444	Fibrobacter succinogenes	GCA_000146505.1_ASM14650v1_genomic.fna
445	Brucella pinnipedialis	GCA_000221005.1_ASM22100v1_genomic.fna
446	Brucella ceti	GCA_000590795.1_ASM59079v1_genomic.fna
447	Enterococcus gallinarum	GCA_001558875.1_ASM155887v1_genomic.fna
448	Enterococcus casseliflavus	GCA_000157355.2_ASM15735v2_genomic.fna
449	Caulobacter crescentus	GCA_000006905.1_ASM690v1_genomic.fna
450	Brucella melitensis	GCA_000007125.1_ASM712v1_genomic.fna
451	Dickeya dadantii	GCA_000147055.1_ASM14705v1_genomic.fna
452	Burkholderia glumae	GCA_000022645.2_ASM2264v1_genomic.fna
453	Clostridium sporogenes	GCA_000973705.1_ASM97370v1_genomic.fna
454	Streptococcus infantarius	GCA_000246835.1_ASM24683v1_genomic.fna
455	Providencia stuartii	GCA_000259175.1_ASM25917v1_genomic.fna
456	Parvimonas micra	GCA_000800295.1_ASM80029v1_genomic.fna
457	Desulfovibrio piger	GCA_900116045.1_DESPIGER_genomic.fna
458	Bacteroides caccae	GCA_002222615.2_ASM222261v2_genomic.fna
459	Bacteroides ovatus	GCA_001314995.1_ASM131499v1_genomic.fna
460	Treponema denticola	GCA_000008185.1_ASM818v1_genomic.fna
461	Azotobacter vinelandii	GCA_000380335.1_ASM38033v1_genomic.fna
462	Mesorhizobium loti	GCA_000009625.1_ASM962v1_genomic.fna
463	Sinorhizobium meliloti	GCA_000006965.1_ASM696v1_genomic.fna
464	Methylococcus capsulatus	GCA_000008325.1_ASM832v1_genomic.fna
465	Bordetella bronchiseptica	GCA_000318015.1_ASM31801v1_genomic.fna
466	Bordetella pertussis	GCA_000195715.1_ASM19571v1_genomic.fna
467	Bordetella parapertussis	GCA_000317935.1_ASM31793v1_genomic.fna
468	Haemophilus ducreyi	GCA_000007945.1_ASM794v1_genomic.fna
469	Rickettsia conorii	GCA_000007025.1_ASM702v1_genomic.fna
470	Dichelobacter nodosus	GCA_000015345.1_ASM1534v1_genomic.fna
471	Nitrosomonas europaea	GCA_000009145.1_ASM914v1_genomic.fna
472	Acidithiobacillus ferrooxidans	GCA_000021485.1_ASM2148v1_genomic.fna
473	Rhodospirillum rubrum	GCA_000013085.1_ASM1308v1_genomic.fna

474	Chloroflexus aurantiacus	GCA_000018865.1_ASM1886v1_genomic.fna
475	Ruminococcus albus	GCA_000179635.2_ASM17963v2_genomic.fna
476	Cytophaga hutchinsonii	GCA_000014145.1_ASM1414v1_genomic.fna
477	Deinococcus radiodurans	GCA_000008565.1_ASM856v1_genomic.fna
478	Streptococcus gordonii	GCA_000017005.1_ASM1700v1_genomic.fna
479	Clostridium acetobutylicum	GCA_000008765.1_ASM876v1_genomic.fna
480	Lactobacillus gasseri	GCA_000014425.1_ASM1442v1_genomic.fna
481	Corynebacterium diphtheriae	GCA_001457455.1_NCTC11397_genomic.fna
482	Listeria innocua	GCA_000195795.1_ASM19579v1_genomic.fna
483	Mycobacterium smegmatis	GCA_000015005.1_ASM1500v1_genomic.fna
484	Thermobifida fusca	GCA_000012405.1_ASM1240v1_genomic.fna
485	Mycoplasma pneumoniae	GCA_000027345.1_ASM2734v1_genomic.fna
486	Mycoplasma pulmonis	GCA_000195875.1_ASM19587v1_genomic.fna
487	Thermotoga maritima	GCA_000008545.1_ASM854v1_genomic.fna
488	Mycoplasma penetrans	GCA_000011225.1_ASM1122v1_genomic.fna
489	Geobacter metallireducens	GCA_000012925.1_ASM1292v1_genomic.fna
490	Streptomyces avermitilis	GCA_000009765.2_ASM976v2_genomic.fna
491	Yersinia enterocolitica	GCA_000009345.1_ASM934v1_genomic.fna
492	Geobacter sulfurreducens	GCA_000007985.2_ASM798v2_genomic.fna
493	Spiroplasma kunkelii	GCA_001274875.1_ASM127487v1_genomic.fna
494	Novosphingobium aromaticivorans	GCA_000013325.1_ASM1332v1_genomic.fna
495	Dehalococcoides mccartyi	GCA_000011905.1_ASM1190v1_genomic.fna
496	Aquifex aeolicus	GCA_000008625.1_ASM862v1_genomic.fna
497	Nostoc punctiforme	GCA_000020025.1_ASM2002v1_genomic.fna
498	Chlamydophila caviae	GCA_000007605.1_ASM760v1_genomic.fna
499	Chlamydia muridarum	GCA_000006685.1_ASM668v1_genomic.fna
500	Bacillus halodurans	GCA_000011145.1_ASM1114v1_genomic.fna
501	Streptomyces coelicolor	GCA_000203835.1_ASM20383v1_genomic.fna
502	Cupriavidus metallidurans	GCA_000196015.1_ASM19601v1_genomic.fna
503	Carboxydothermus hydrogenoformans	GCA_000012865.1_ASM1286v1_genomic.fna
504	Paraburkholderia xenovorans	GCA_000013645.1_ASM1364v1_genomic.fna
505	Wolbachia endosymbiont	GCA_000008025.1_ASM802v1_genomic.fna
506	Wigglesworthia glossinidia	GCA_000008885.1_ASM888v1_genomic.fna
507	Ruegeria pomeroyi	GCA_000011965.2_ASM1196v2_genomic.fna

508	Oceanobacillus iheyensis	GCA_000011245.1_ASM1124v1_genomic.fna
509	Colwellia psychrerythraea	GCA_000012325.1_ASM1232v1_genomic.fna
510	Corynebacterium efficiens	GCA_000011305.1_ASM1130v1_genomic.fna
511	Thermosynechococcus elongatus	GCA_000011345.1_ASM1134v1_genomic.fna
512	Chlorobium tepidum	GCA_000006985.1_ASM698v1_genomic.fna
513	Leuconostoc mesenteroides	GCA_000014445.1_ASM1444v1_genomic.fna
514	Saccharophagus degradans	GCA_000013665.1_ASM1366v1_genomic.fna
515	Trichodesmium erythraeum	GCA_000014265.1_ASM1426v1_genomic.fna
516	Shewanella oneidensis	GCA_000146165.2_ASM14616v2_genomic.fna
517	Anaplasma phagocytophilum	GCA_000013125.1_ASM1312v1_genomic.fna
518	Bordetella avium	GCA_000070465.1_ASM7046v1_genomic.fna
519	Chlamydia abortus	GCA_000026025.1_ASM2602v1_genomic.fna
520	Acidobacterium capsulatum	GCA_000022565.1_ASM2256v1_genomic.fna
521	Neorickettsia sennetsu	GCA_000013165.1_ASM1316v1_genomic.fna
522	Pectobacterium atrosepticum	GCA_000011605.1_ASM1160v1_genomic.fna
523	Salmonella bongori	GCA_000252995.1_ASM25299v1_genomic.fna
524	Streptococcus uberis	GCA_000009545.1_ASM954v1_genomic.fna
525	Chlamydia felis	GCA_000009945.1_ASM994v1_genomic.fna
526	Bacteroides thetaiotaomicron	GCA_000011065.1_ASM1106v1_genomic.fna
527	Rhodopirellula baltica	GCA_000196115.1_ASM19611v1_genomic.fna
528	Bartonella quintana	GCA_000046685.1_ASM4668v1_genomic.fna
529	Rhodobacter capsulatus	GCA_000021865.1_ASM2186v1_genomic.fna
530	Clostridium tetani	GCA_000007625.1_ASM762v1_genomic.fna
531	Lactobacillus acidophilus	GCA_000011985.1_ASM1198v1_genomic.fna
532	Lawsonia intracellularis	GCA_000055945.1_ASM5594v1_genomic.fna
533	Helicobacter hepaticus	GCA_000007905.1_ASM790v1_genomic.fna
534	Bartonella henselae	GCA_000046705.1_ASM4670v1_genomic.fna
535	Leifsonia xyli	GCA_000470775.1_ASM47077v1_genomic.fna
536	Mannheimia haemolytica	GCA_000376645.1_ASM37664v1_genomic.fna
537	Lactobacillus plantarum	GCA_000203855.3_ASM20385v3_genomic.fna
538	Pediococcus pentosaceus	GCA_000014505.1_ASM1450v1_genomic.fna
539	Lactobacillus brevis	GCA_000014465.1_ASM1446v1_genomic.fna
540	Brevibacterium linens	GCA_001606005.1_ASM160600v1_genomic.fna
541	Serratia marcescens	GCA_000513215.1_DB11_genomic.fna

542	Mycoplasma gallisepticum	GCA_000092585.1_ASM9258v1_genomic.fna
543	Wolinella succinogenes	GCA_000196135.1_ASM19613v1_genomic.fna
544	Chromobacterium violaceum	GCA_000007705.1_ASM770v1_genomic.fna
545	Myxococcus xanthus	GCA_000012685.1_ASM1268v1_genomic.fna
546	Mycoplasma arthritidis	GCA_000020065.1_ASM2006v1_genomic.fna
547	Photorhabdus luminescens	GCA_000196155.1_ASM19615v1_genomic.fna
548	Gloeobacter violaceus	GCA_000011385.1_ASM1138v1_genomic.fna
549	Onion yellows	GCA_000009845.1_ASM984v1_genomic.fna
550	Methylobacillus flagellatus	GCA_000013705.1_ASM1370v1_genomic.fna
551	Moorella thermoacetica	GCA_000013105.1_ASM1310v1_genomic.fna
552	Exiguobacterium sibiricum	GCA_000019905.1_ASM1990v1_genomic.fna
553	Mesoplasma florum	GCA_000008305.1_ASM830v1_genomic.fna
554	Rubrobacter xylanophilus	GCA_000014185.1_ASM1418v1_genomic.fna
555	Kineococcus radiotolerans	GCA_000017305.1_ASM1730v1_genomic.fna
556	Ehrlichia canis	GCA_000012565.1_ASM1256v1_genomic.fna
557	Burkholderia vietnamiensis	GCA_000959445.1_ASM95944v1_genomic.fna
558	Methylibium petroleiphilum	GCA_000015725.1_ASM1572v1_genomic.fna
559	Propionibacterium acnes	GCA_000008345.1_ASM834v1_genomic.fna
560	Baumannia cicadellinicola	GCA_000013185.1_ASM1318v1_genomic.fna
561	Staphylococcus haemolyticus	GCA_000009865.1_ASM986v1_genomic.fna
562	Brucella ovis	GCA_000016845.1_ASM1684v1_genomic.fna
563	Campylobacter coli	GCA_002024185.1_ASM202418v1_genomic.fna
564	Mycoplasma bovis	GCA_000183385.1_ASM18338v1_genomic.fna
565	Campylobacter lari	GCA_000019205.1_ASM1920v1_genomic.fna
566	Persephonella marina	GCA_000021565.1_ASM2156v1_genomic.fna
567	Simkania negevensis	GCA_000237205.1_ASM23720v1_genomic.fna
568	Sulfurihydrogenibium azorense	GCA_000021545.1_ASM2154v1_genomic.fna
569	Erwinia amylovora	GCA_000091565.1_ASM9156v1_genomic.fna
570	Chlorobium limicola	GCA_000020465.1_ASM2046v1_genomic.fna
571	Chlorobium phaeovibrioides	GCA_000016085.1_ASM1608v1_genomic.fna
572	Halobacteriovorax marinus	GCA_000210915.2_ASM21091v2_genomic.fna
573	Proteus mirabilis	GCA_000069965.1_ASM6996v1_genomic.fna
574	Klebsiella oxytoca	GCA_001022195.1_ASM102219v1_genomic.fna
575	Clostridium beijerinckii	GCA_000833105.2_ASM83310v2_genomic.fna

576	Cronobacter sakazakii	GCA_000982825.1_ASM98282v1_genomic.fna
577	Citrobacter koseri	GCA_000018045.1_ASM1804v1_genomic.fna
578	Rickettsia canadensis	GCA_000014345.1_ASM1434v1_genomic.fna
579	Rickettsia akari	GCA_000018205.1_ASM1820v1_genomic.fna
580	Streptomyces scabiei	GCA_000091305.1_ASM9130v1_genomic.fna
581	Acaryochloris marina	GCA_000018105.1_ASM1810v1_genomic.fna
582	Pelodictyon phaeoclathratiforme	GCA_000020645.1_ASM2064v1_genomic.fna
583	Syntrophobacter fumaroxidans	GCA_000014965.1_ASM1496v1_genomic.fna
584	Syntrophomonas wolfei	GCA_000014725.1_ASM1472v1_genomic.fna
585	Hydrogenovibrio crunogenus	GCA_000012605.1_ASM1260v1_genomic.fna
586	Sulfurimonas denitrificans	GCA_000012965.1_ASM1296v1_genomic.fna
587	Prosthecochloris aestuarii	GCA_000020625.1_ASM2062v1_genomic.fna
588	Desulfotalea psychrophila	GCA_000025945.1_ASM2594v1_genomic.fna
589	Thiobacillus denitrificans	GCA_000012745.1_ASM1274v1_genomic.fna
590	Mannheimia succiniciproducens	GCA_000007745.1_ASM774v1_genomic.fna
591	Nocardia farcinica	GCA_001182745.2_NCTC11134_genomic.fna
592	Eubacterium eligens	GCA_000146185.1_ASM14618v1_genomic.fna
593	Photobacterium profundum	GCA_000196255.1_ASM19625v1_genomic.fna
594	Marinobacter hydrocarbonoclasticus	GCA_000284615.1_ASM28461v1_genomic.fna
595	Nautilia profundicola	GCA_000021725.1_ASM2172v1_genomic.fna
596	Aromatoleum aromaticum	GCA_000025965.1_ASM2596v1_genomic.fna
597	Lactobacillus salivarius	GCA_000008925.1_ASM892v1_genomic.fna
598	Bacillus clausii	GCA_000009825.1_ASM982v1_genomic.fna
599	Gluconobacter oxydans	GCA_000011685.1_ASM1168v1_genomic.fna
600	Pelobacter carbinolicus	GCA_000012885.1_ASM1288v1_genomic.fna
601	Wolbachia endosymbiont	GCA_000376585.1_ASM37658v1_genomic.fna
602	Actinobacillus succinogenes	GCA_000017245.1_ASM1724v1_genomic.fna
603	Bacillus selenitireducens	GCA_000093085.1_ASM9308v1_genomic.fna
604	Agrobacterium vitis	GCA_000016285.1_ASM1628v1_genomic.fna
605	Enterobacter cloacae	GCA_000025565.1_ASM2556v1_genomic.fna
606	Bacteroides vulgatus	GCA_000012825.1_ASM1282v1_genomic.fna
607	Pelobacter propionicus	GCA_000015045.1_ASM1504v1_genomic.fna
608	Shewanella amazonensis	GCA_000015245.1_ASM1524v1_genomic.fna
609	Shewanella denitrificans	GCA_000013765.1_ASM1376v1_genomic.fna

610	Shewanella frigidimarina	GCA_000014705.1_ASM1470v1_genomic.fna
611	Xenorhabdus bovienii	GCA_000027225.1_ASM2722v1_genomic.fna
612	Xenorhabdus nematophila	GCA_000953355.1_XNC2_genomic.fna
613	Spiroplasma citri	GCA_001886855.1_ASM188685v1_genomic.fna
614	Moraxella catarrhalis	GCA_000092265.1_ASM9226v1_genomic.fna
615	Mycoplasma fermentans	GCA_000186005.1_ASM18600v1_genomic.fna
616	Polaromonas naphthalenivorans	GCA_000015505.1_ASM1550v1_genomic.fna
617	Citrobacter rodentium	GCA_000027085.1_ASM2708v1_genomic.fna
618	Desulfotomaculum reducens	GCA_000016165.1_ASM1616v1_genomic.fna
619	Helicobacter mustelae	GCA_000091985.1_ASM9198v1_genomic.fna
620	Heliobacterium modesticaldum	GCA_000019165.1_ASM1916v1_genomic.fna
621	Listeria ivanovii	GCA_000252975.1_ASM25297v1_genomic.fna
622	Deinococcus geothermalis	GCA_000196275.1_ASM19627v1_genomic.fna
623	Listeria seeligeri	GCA_000027145.1_ASM2714v1_genomic.fna
624	Listeria welshimeri	GCA_000060285.1_ASM6028v1_genomic.fna
625	Pseudoalteromonas atlantica	GCA_000014225.1_ASM1422v1_genomic.fna
626	Roseiflexus castenholzii	GCA_000017805.1_ASM1780v1_genomic.fna
627	Caldicellulosiruptor saccharolyticus	GCA_000016545.1_ASM1654v1_genomic.fna
628	Herminiimonas arsenicoxydans	GCA_000026125.1_ASM2612v1_genomic.fna
629	Edwardsiella ictaluri	GCA_000022885.2_ASM2288v2_genomic.fna
630	Neisseria lactamica	GCA_000196295.1_ASM19629v1_genomic.fna
631	Nitrobacter hamburgensis	GCA_000013885.1_ASM1388v1_genomic.fna
632	Nitrobacter winogradskyi	GCA_000012725.1_ASM1272v1_genomic.fna
633	Aster yellows	GCA_000012225.1_ASM1222v1_genomic.fna
634	Erythrobacter litoralis	GCA_000013005.1_ASM1300v1_genomic.fna
635	Parabacteroides distasonis	GCA_000012845.1_ASM1284v1_genomic.fna
636	Bifidobacterium breve	GCA_001025175.1_ASM102517v1_genomic.fna
637	Psychroflexus torquis	GCA_000153485.2_ASM15348v2_genomic.fna
638	Clostridium ljungdahlii	GCA_000143685.1_ASM14368v1_genomic.fna
639	Bacillus mycoides	GCA_000775975.1_ASM77597v1_genomic.fna
640	Croceibacter atlanticus	GCA_000196315.1_ASM19631v1_genomic.fna
641	Rhodococcus jostii	GCA_000014565.1_ASM1456v1_genomic.fna
642	Rickettsia felis	GCA_000012145.1_ASM1214v1_genomic.fna
643	Shewanella loihica	GCA_000016065.1_ASM1606v1_genomic.fna

644	Sphingopyxis alaskensis	GCA_000013985.1_ASM1398v1_genomic.fna
645	Rhodoferax ferrireducens	GCA_000013605.1_ASM1360v1_genomic.fna
646	Nitrosospira multiformis	GCA_000196355.1_ASM19635v1_genomic.fna
647	Nitrosomonas eutropha	GCA_000014765.1_ASM1476v1_genomic.fna
648	Psychrobacter cryohalolentis	GCA_000013905.1_ASM1390v1_genomic.fna
649	Chlorobium chlorochromatii	GCA_000012585.1_ASM1258v1_genomic.fna
650	Streptococcus sanguinis	GCA_000014205.1_ASM1420v1_genomic.fna
651	Staphylococcus saprophyticus	GCA_000010125.1_ASM1012v1_genomic.fna
652	Pseudothermotoga lettingae	GCA_000017865.1_ASM1786v1_genomic.fna
653	Stigmatella aurantiaca	GCA_000165485.1_ASM16548v1_genomic.fna
654	Bacillus coagulans	GCA_000832905.1_ASM83290v1_genomic.fna
655	Mycobacterium abscessus	GCA_000069185.1_ASM6918v1_genomic.fna
656	Desulfovibrio magneticus	GCA_000010665.1_ASM1066v1_genomic.fna
657	Herbaspirillum seropedicae	GCA_001040945.1_ASM104094v1_genomic.fna
658	Alkalilimnicola ehrlichii	GCA_000014785.1_ASM1478v1_genomic.fna
659	Mycobacterium vanbaalenii	GCA_000015305.1_ASM1530v1_genomic.fna
660	Halorhodospira halophila	GCA_000015585.1_ASM1558v1_genomic.fna
661	Geobacter uraniireducens	GCA_000016745.1_ASM1674v1_genomic.fna
662	Acidiphilium cryptum	GCA_000016725.1_ASM1672v1_genomic.fna
663	Hahella chejuensis	GCA_000012985.1_ASM1298v1_genomic.fna
664	Shewanella violacea	GCA_000091325.1_ASM9132v1_genomic.fna
665	Xanthobacter autotrophicus	GCA_000017645.1_ASM1764v1_genomic.fna
666	Paenibacillus polymyxa	GCA_000164985.2_ASM16498v2_genomic.fna
667	Leuconostoc citreum	GCA_000026405.1_ASM2640v1_genomic.fna
668	Alkaliphilus oremlandii	GCA_000018325.1_ASM1832v1_genomic.fna
669	Flavobacterium johnsoniae	GCA_000016645.1_ASM1664v1_genomic.fna
670	Collimonas fungivorans	GCA_000221045.1_ASM22104v1_genomic.fna
671	Legionella longbeachae	GCA_000091785.1_ASM9178v1_genomic.fna
672	Acidothermus cellulolyticus	GCA_000015025.1_ASM1502v1_genomic.fna
673	Zobellia galactanivorans	GCA_000973105.1_ASM97310v1_genomic.fna
674	Yersinia intermedia	GCA_000834515.1_ASM83451v1_genomic.fna
675	Salinibacter ruber	GCA_000013045.1_ASM1304v1_genomic.fna
676	Lachnoclostridium phytofermentans	GCA_000018685.1_ASM1868v1_genomic.fna
677	Psychromonas ingrahamii	GCA_000015285.1_ASM1528v1_genomic.fna

678	Syntrophus aciditrophicus	GCA_000013405.1_ASM1340v1_genomic.fna
679	Ramlibacter tataouinensis	GCA_000215705.1_ASM21570v1_genomic.fna
680	Polynucleobacter necessarius	GCA_000019745.1_ASM1974v1_genomic.fna
681	Deinococcus deserti	GCA_000020685.1_ASM2068v1_genomic.fna
682	Burkholderia dolosa	GCA_000959505.1_ASM95950v1_genomic.fna
683	Herpetosiphon aurantiacus	GCA_000018565.1_ASM1856v1_genomic.fna
684	Halothermothrix orenii	GCA_000020485.1_ASM2048v1_genomic.fna
685	Sinorhizobium medicae	GCA_000017145.1_ASM1714v1_genomic.fna
686	Aeromonas hydrophila	GCA_000014805.1_ASM1480v1_genomic.fna
687	Propionibacterium freudenreichii	GCA_000940845.1_ASM94084v1_genomic.fna
688	Chloroflexus aggregans	GCA_000021945.1_ASM2194v1_genomic.fna
689	Fervidobacterium nodosum	GCA_000017545.1_ASM1754v1_genomic.fna
690	Pseudomonas entomophila	GCA_000026105.1_ASM2610v1_genomic.fna
691	Pseudomonas stutzeri	GCA_000590475.1_ASM59047v1_genomic.fna
692	Clostridium novyi	GCA_000014125.1_ASM1412v1_genomic.fna
693	Thermotoga petrophila	GCA_000016785.1_ASM1678v1_genomic.fna
694	Streptococcus iniae	GCA_000831485.1_ASM83148v1_genomic.fna
695	Granulibacter bethesdensis	GCA_000014285.2_ASM1428v2_genomic.fna
696	Campylobacter concisus	GCA_001298465.1_ASM129846v1_genomic.fna
697	Campylobacter curvus	GCA_000017465.2_ASM1746v2_genomic.fna
698	Verminephrobacter eiseniae	GCA_000015565.1_ASM1556v1_genomic.fna
699	Thermosipho melanesiensis	GCA_000016905.1_ASM1690v1_genomic.fna
700	Sphingomonas wittichii	GCA_000016765.1_ASM1676v1_genomic.fna
701	Frankia alni	GCA_000058485.1_ASM5848v1_genomic.fna
702	Paraburkholderia phymatum	GCA_000020045.1_ASM2004v1_genomic.fna
703	Shewanella pealeana	GCA_000018285.1_ASM1828v1_genomic.fna
704	Dinoroseobacter shibae	GCA_000018145.1_ASM1814v1_genomic.fna
705	Clostridium cellulolyticum	GCA_000022065.1_ASM2206v1_genomic.fna
706	Geobacter lovleyi	GCA_000020385.1_ASM2038v1_genomic.fna
707	Shewanella woodyi	GCA_000019525.1_ASM1952v1_genomic.fna
708	Helicobacter acinonychis	GCA_000009305.1_ASM930v1_genomic.fna
709	Maricaulis maris	GCA_000014745.1_ASM1474v1_genomic.fna
710	Pseudomonas mendocina	GCA_000016565.1_ASM1656v1_genomic.fna
711	Serratia proteamaculans	GCA_000018085.1_ASM1808v1_genomic.fna

712	Petrotoga mobilis	GCA_000018605.1_ASM1860v1_genomic.fna
713	Geobacter bemidjiensis	GCA_000020725.1_ASM2072v1_genomic.fna
714	Paraburkholderia phytofirmans	GCA_000020125.1_ASM2012v1_genomic.fna
715	Parvibaculum lavamentivorans	GCA_000017565.1_ASM1756v1_genomic.fna
716	Shewanella piezotolerans	GCA_000014885.1_ASM1488v1_genomic.fna
717	Desulfococcus oleovorans	GCA_000018405.1_ASM1840v1_genomic.fna
718	Borrelia duttonii	GCA_000019685.1_ASM1968v1_genomic.fna
719	Borrelia recurrentis	GCA_000019705.1_ASM1970v1_genomic.fna
720	Rickettsia africae	GCA_000023005.1_ASM2300v1_genomic.fna
721	Rickettsia massiliae	GCA_000016625.1_ASM1662v1_genomic.fna
722	Rhodospirillum centenum	GCA_000016185.1_ASM1618v1_genomic.fna
723	Labrenzia aggregata	GCA_001999245.1_ASM199924v1_genomic.fna
724	Geobacillus thermodenitrificans	GCA_000015745.1_ASM1574v1_genomic.fna
725	Shewanella sediminis	GCA_000018025.1_ASM1802v1_genomic.fna
726	Finegoldia magna	GCA_000010185.1_ASM1018v1_genomic.fna
727	Burkholderia ubonensis	GCA_000959245.1_ASM95924v1_genomic.fna
728	Pelotomaculum thermopropionicum	GCA_000010565.1_ASM1056v1_genomic.fna
729	Magnetospirillum gryphiswaldense	GCA_000513295.1_MGMSRv2_genomic.fna
730	Neorickettsia risticii	GCA_000022525.1_ASM2252v1_genomic.fna
731	Renibacterium salmoninarum	GCA_000018885.1_ASM1888v1_genomic.fna
732	Acholeplasma laidlawii	GCA_000018785.1_ASM1878v1_genomic.fna
733	Azorhizobium caulinodans	GCA_000010525.1_ASM1052v1_genomic.fna
734	Caldanaerobacter subterraneus	GCA_000007085.1_ASM708v1_genomic.fna
735	Marinitoga piezophila	GCA_000255135.1_ASM25513v1_genomic.fna
736	Roseobacter litoralis	GCA_000154785.2_ASM15478v2_genomic.fna
737	Vibrio shilonii	GCA_002214345.1_ASM221434v1_genomic.fna
738	Methylobacterium populi	GCA_000019945.1_ASM1994v1_genomic.fna
739	Desulfatibacillum alkenivorans	GCA_000021905.1_ASM2190v1_genomic.fna
740	Lysinibacillus sphaericus	GCA_001753205.1_ASM175320v1_genomic.fna
741	Elusimicrobium minutum	GCA_000020145.1_ASM2014v1_genomic.fna
742	Borreliella valaisiana	GCA_000739475.1_ASM73947v1_genomic.fna
743	Phenylobacterium zucineum	GCA_000017265.1_ASM1726v1_genomic.fna
744	Flavobacterium psychrophilum	GCA_000064305.2_ASM6430v2_genomic.fna
745	Opitutus terrae	GCA_000019965.1_ASM1996v1_genomic.fna

746	Pseudarthrobacter chlorophenolicus	GCA_000022025.1_ASM2202v1_genomic.fna
747	Clostridium butyricum	GCA_001456065.2_ASM145606v2_genomic.fna
748	Leptothrix cholodnii	GCA_000019785.1_ASM1978v1_genomic.fna
749	Campylobacter hominis	GCA_000017585.1_ASM1758v1_genomic.fna
750	Pseudarthrobacter phenanthrenivorans	GCA_000189535.1_ASM18953v1_genomic.fna
751	Akkermansia muciniphila	GCA_000020225.1_ASM2022v1_genomic.fna
752	Mycobacterium liflandii	GCA_000026445.2_ASM2644v2_genomic.fna
753	Shewanella halifaxensis	GCA_000019185.1_ASM1918v1_genomic.fna
754	Methylobacterium nodulans	GCA_000022085.1_ASM2208v1_genomic.fna
755	Erwinia tasmaniensis	GCA_000026185.1_ASM2618v1_genomic.fna
756	Methylocella silvestris	GCA_000021745.1_ASM2174v1_genomic.fna
757	Beijerinckia indica	GCA_000019845.1_ASM1984v1_genomic.fna
758	Desulfobacterium autotrophicum	GCA_000020365.1_ASM2036v1_genomic.fna
759	Gemmatimonas aurantiaca	GCA_000010305.1_ASM1030v1_genomic.fna
760	Natranaerobius thermophilus	GCA_000020005.1_ASM2000v1_genomic.fna
761	Macrococcus caseolyticus	GCA_000010585.1_ASM1058v1_genomic.fna
762	Rhodococcus erythropolis	GCA_000454045.1_ASM45404v1_genomic.fna
763	Psychrobacter arcticus	GCA_000012305.1_ASM1230v1_genomic.fna
764	Dechloromonas aromatica	GCA_000012425.1_ASM1242v1_genomic.fna
765	Bdellovibrio bacteriovorus	GCA_000525675.1_ASM52567v1_genomic.fna
766	Lactobacillus johnsonii	GCA_000008065.1_ASM806v1_genomic.fna
767	Prevotella ruminicola	GCA_000025925.1_ASM2592v1_genomic.fna
768	Mycoplasma synoviae	GCA_000969765.1_ASM96976v1_genomic.fna
769	Idiomarina loihiensis	GCA_000008465.1_ASM846v1_genomic.fna
770	Ammonifex degensii	GCA_000024605.1_ASM2460v1_genomic.fna
771	Symbiobacterium thermophilum	GCA_000009905.1_ASM990v1_genomic.fna
772	Rickettsia typhi	GCA_000008045.1_ASM804v1_genomic.fna
773	Mycoplasma mobile	GCA_000008365.1_ASM836v1_genomic.fna
774	Alcanivorax borkumensis	GCA_000009365.1_ASM936v1_genomic.fna
775	Alkaliphilus metalliredigens	GCA_000016985.1_ASM1698v1_genomic.fna
776	Chlorobium luteolum	GCA_000012485.1_ASM1248v1_genomic.fna
777	Paracoccus denitrificans	GCA_000203895.1_ASM20389v1_genomic.fna
778	Geobacillus kaustophilus	GCA_000009785.1_ASM978v1_genomic.fna
779	Mycoplasma haemofelis	GCA_000186985.3_ASM18698v3_genomic.fna

780	Lactobacillus sakei	GCA_002224565.1_ASM222456v1_genomic.fna
781	Loktanella vestfoldensis	GCA_002158905.1_ASM215890v1_genomic.fna
782	Robiginitalea biformata	GCA_000024125.1_ASM2412v1_genomic.fna
783	Paenibacillus larvae	GCA_002003265.1_ASM200326v1_genomic.fna
784	Dokdonia donghaensis	GCA_001653755.1_ASM165375v1_genomic.fna
785	Borrelia turicatae	GCA_000012085.2_ASM1208v2_genomic.fna
786	Acidovorax avenae	GCA_000176855.2_ASM17685v2_genomic.fna
787	Parvularcula bermudensis	GCA_000152825.2_ASM15282v2_genomic.fna
788	Hyphomonas neptunium	GCA_000013025.1_ASM1302v1_genomic.fna
789	Pseudoalteromonas haloplanktis	GCA_000026085.1_ASM2608v1_genomic.fna
790	Arcobacter butzleri	GCA_000014025.1_ASM1402v1_genomic.fna
791	Xanthomonas albilineans	GCA_000087965.1_ASM8796v1_genomic.fna
792	Mycoplasma leachii	GCA_000183365.1_ASM18336v1_genomic.fna
793	Thermosipho africanus	GCA_000021285.1_ASM2128v1_genomic.fna
794	Kocuria rhizophila	GCA_000010285.1_ASM1028v1_genomic.fna
795	Myxococcus fulvus	GCA_000219105.1_ASM21910v1_genomic.fna
796	Mycobacterium intracellulare	GCA_000277125.1_ASM27712v1_genomic.fna
797	Sorangium cellulosum	GCA_000067165.1_ASM6716v1_genomic.fna
798	Bordetella petrii	GCA_000067205.1_ASM6720v1_genomic.fna
799	Cellvibrio japonicus	GCA_000019225.1_ASM1922v1_genomic.fna
800	Mycobacterium gilvum	GCA_000184435.1_ASM18443v1_genomic.fna
801	Edwardsiella tarda	GCA_000020865.1_ASM2086v1_genomic.fna
802	Anoxybacillus flavithermus	GCA_000019045.1_ASM1904v1_genomic.fna
803	Streptomyces pristinaespiralis	GCA_001278075.1_ASM127807v1_genomic.fna
804	Acetoanaerobium sticklandii	GCA_000196455.1_ASM19645v1_genomic.fna
805	Thermus aquaticus	GCA_001399775.1_ASM139977v1_genomic.fna
806	Oligotropha carboxidovorans	GCA_000218565.1_ASM21856v1_genomic.fna
807	Escherichia albertii	GCA_000512125.1_ASM51212v1_genomic.fna
808	Bacillus pseudofirmus	GCA_000005825.2_ASM582v2_genomic.fna
809	Methylacidiphilum infernorum	GCA_000019665.1_ASM1966v1_genomic.fna
810	Lactobacillus buchneri	GCA_000298115.2_ASM29811v2_genomic.fna
811	Mycoplasma crocodyli	GCA_000025845.1_ASM2584v1_genomic.fna
812	Eubacterium rectale	GCA_000020605.1_ASM2060v1_genomic.fna
813	Brevibacillus brevis	GCA_000010165.1_ASM1016v1_genomic.fna

814	Thioalkalivibrio sulfidiphilus	GCA_000021985.1_ASM2198v1_genomic.fna
815	Corynebacterium urealyticum	GCA_000069945.1_ASM6994v1_genomic.fna
816	Dictyoglomus turgidum	GCA_000021645.1_ASM2164v1_genomic.fna
817	Chlorobaculum parvum	GCA_000020505.1_ASM2050v1_genomic.fna
818	Chloroherpeton thalassium	GCA_000020525.1_ASM2052v1_genomic.fna
819	Caldicellulosiruptor bescii	GCA_000022325.1_ASM2232v1_genomic.fna
820	Borrelia hermsii	GCA_001660005.1_ASM166000v1_genomic.fna
821	Kosmotoga olearia	GCA_000023325.1_ASM2332v1_genomic.fna
822	Clostridium carboxidivorans	GCA_001038625.1_ASM103862v1_genomic.fna
823	Desulfovibrio salexigens	GCA_000023445.1_ASM2344v1_genomic.fna
824	Yersinia aldovae	GCA_000834395.1_ASM83439v1_genomic.fna
825	Yersinia rohdei	GCA_000834455.1_ASM83445v1_genomic.fna
826	Yersinia ruckeri	GCA_000964565.1_ASM96456v1_genomic.fna
827	Pirellula staleyi	GCA_000025185.1_ASM2518v1_genomic.fna
828	Acidovorax ebreus	GCA_000022305.1_ASM2230v1_genomic.fna
829	Treponema primitia	GCA_000214375.1_ASM21437v1_genomic.fna
830	Treponema azotonutricium	GCA_000214355.1_ASM21435v1_genomic.fna
831	Alicyclobacillus acidocaldarius	GCA_000024285.1_ASM2428v1_genomic.fna
832	Rhodococcus opacus	GCA_000010805.1_ASM1080v1_genomic.fna
833	Variovorax paradoxus	GCA_000184745.1_ASM18474v1_genomic.fna
834	Photorhabdus asymbiotica	GCA_000196475.1_ASM19647v1_genomic.fna
835	Aliivibrio salmonicida	GCA_000196495.1_ASM19649v1_genomic.fna
836	Coprothermobacter proteolyticus	GCA_000020945.1_ASM2094v1_genomic.fna
837	Dictyoglomus thermophilum	GCA_000020965.1_ASM2096v1_genomic.fna
838	Thermodesulfovibrio yellowstonii	GCA_000020985.1_ASM2098v1_genomic.fna
839	Laribacter hongkongensis	GCA_000021025.1_ASM2102v1_genomic.fna
840	Treponema paraluiscuniculi	GCA_000217655.1_ASM21765v1_genomic.fna
841	Teredinibacter turnerae	GCA_000023025.1_ASM2302v1_genomic.fna
842	Mycobacterium kansasii	GCA_000157895.2_ASM15789v2_genomic.fna
843	Ferrimonas balearica	GCA_000148645.1_ASM14864v1_genomic.fna
844	'Nostoc azollae'	GCA_000196515.1_ASM19651v1_genomic.fna
845	Thermosediminibacter oceani	GCA_000144645.1_ASM14464v1_genomic.fna
846	Halothiobacillus neapolitanus	GCA_000024765.1_ASM2476v1_genomic.fna
847	Pectobacterium carotovorum	GCA_000023605.1_ASM2360v1_genomic.fna

848	Brachyspira hyodysenteriae	GCA_001676785.2_ASM167678v2_genomic.fna
849	Pectobacterium wasabiae	GCA_001742185.1_ASM174218v1_genomic.fna
850	Bacillus vallismortis	GCA_002113805.1_ASM211380v1_genomic.fna
851	Rickettsia peacockii	GCA_000021525.1_ASM2152v1_genomic.fna
852	Kyrpidia tusciae	GCA_000092905.1_ASM9290v1_genomic.fna
853	Sulfurimonas autotrophica	GCA_000147355.1_ASM14735v1_genomic.fna
854	Rothia mucilaginosa	GCA_000011025.1_ASM1102v1_genomic.fna
855	Dickeya zeae	GCA_000025065.1_ASM2506v1_genomic.fna
856	Aggregatibacter actinomycetemcomitans	GCA_001594265.1_ASM159426v1_genomic.fna
857	Allochromatium vinosum	GCA_000025485.1_ASM2548v1_genomic.fna
858	Ilyobacter polytropus	GCA_000165505.1_ASM16550v1_genomic.fna
859	Thermoanaerobacter brockii	GCA_000175295.2_ASM17529v2_genomic.fna
860	Aminobacterium colombiense	GCA_000025885.1_ASM2588v1_genomic.fna
861	Brucella microti	GCA_000022745.1_ASM2274v1_genomic.fna
862	Halanaerobium praevalens	GCA_000165465.1_ASM16546v1_genomic.fna
863	Arcobacter nitrofigilis	GCA_000092245.1_ASM9224v1_genomic.fna
864	Asticcacaulis excentricus	GCA_000175215.2_ASM17521v2_genomic.fna
865	Sediminispirochaeta smaragdinae	GCA_000143985.1_ASM14398v1_genomic.fna
866	Anaplasma centrale	GCA_000024505.1_ASM2450v1_genomic.fna
867	Acetohalobium arabaticum	GCA_000144695.1_ASM14469v1_genomic.fna
868	Isosphaera pallida	GCA_000186345.1_ASM18634v1_genomic.fna
869	Gallionella capsiferriformans	GCA_000145255.1_ASM14525v1_genomic.fna
870	Desulfobulbus propionicus	GCA_000186885.1_ASM18688v1_genomic.fna
871	Thermoanaerobacter italicus	GCA_000025645.1_ASM2564v1_genomic.fna
872	Sideroxydans lithotrophicus	GCA_000025705.1_ASM2570v1_genomic.fna
873	Thermoanaerobacterium thermosaccharolyticun	GCA_000145615.1_ASM14561v1_genomic.fna
874	Clostridium cellulovorans	GCA_000145275.1_ASM14527v1_genomic.fna
875	Hirschia baltica	GCA_000023785.1_ASM2378v1_genomic.fna
876	Methylovorus glucosetrophus	GCA_000023745.1_ASM2374v1_genomic.fna
877	Hyphomicrobium denitrificans	GCA_000143145.1_ASM14314v1_genomic.fna
878	Thermoanaerobacter mathranii	GCA_000092965.1_ASM9296v1_genomic.fna
879	Coraliomargarita akajimensis	GCA_000025905.1_ASM2590v1_genomic.fna
880	Escherichia fergusonii	GCA_000026225.1_ASM2622v1_genomic.fna
881	Streptococcus intermedius	GCA_000463355.1_ASM46335v1_genomic.fna

882	Streptomyces lividans	GCA_000739105.1_ASM73910v1_genomic.fna
883	Streptomyces hygroscopicus	GCA_000245355.1_ASM24535v1_genomic.fna
884	Desulfurivibrio alkaliphilus	GCA_000092205.1_ASM9220v1_genomic.fna
885	Thiomonas intermedia	GCA_002028405.1_ASM202840v1_genomic.fna
886	Thermotoga naphthophila	GCA_000025105.1_ASM2510v1_genomic.fna
887	Cellulomonas fimi	GCA_000212695.1_ASM21269v1_genomic.fna
888	Cellulomonas gilvus	GCA_000218545.1_ASM21854v1_genomic.fna
889	Mesorhizobium opportunistum	GCA_000176035.2_ASM17603v2_genomic.fna
890	Tolumonas auensis	GCA_000023065.1_ASM2306v1_genomic.fna
891	Hydrogenobacter thermophilus	GCA_000010785.1_ASM1078v1_genomic.fna
892	Acidaminococcus fermentans	GCA_000025305.1_ASM2530v1_genomic.fna
893	Erwinia pyrifoliae	GCA_000027265.1_ASM2726v1_genomic.fna
894	Erwinia billingiae	GCA_000196615.1_ASM19661v1_genomic.fna
895	Bartonella grahamii	GCA_000022725.1_ASM2272v1_genomic.fna
896	Staphylococcus carnosus	GCA_000009405.1_ASM940v1_genomic.fna
897	Mycoplasma hyorhinis	GCA_000313635.1_ASM31363v1_genomic.fna
898	Acidithiobacillus caldus	GCA_000175575.2_ASM17557v2_genomic.fna
899	Aggregatibacter aphrophilus	GCA_000022985.1_ASM2298v1_genomic.fna
900	Nitrosococcus halophilus	GCA_000024725.1_ASM2472v1_genomic.fna
901	Olsenella uli	GCA_000143845.1_ASM14384v1_genomic.fna
902	Thermocrinis albus	GCA_000025605.1_ASM2560v1_genomic.fna
903	Caulobacter segnis	GCA_000092285.1_ASM9228v1_genomic.fna
904	Brevundimonas subvibrioides	GCA_000144605.1_ASM14460v1_genomic.fna
905	Bacteroides dorei	GCA_001640865.1_ASM164086v1_genomic.fna
906	Lactobacillus ruminis	GCA_000224985.1_ASM22498v1_genomic.fna
907	Lactobacillus jensenii	GCA_001936235.1_ASM193623v1_genomic.fna
908	Gardnerella vaginalis	GCA_000025205.1_ASM2520v1_genomic.fna
909	Prevotella melaninogenica	GCA_000144405.1_ASM14440v1_genomic.fna
910	Rothia dentocariosa	GCA_000164695.2_ASM16469v2_genomic.fna
911	Bifidobacterium angulatum	GCA_001025155.1_ASM102515v1_genomic.fna
912	Providencia rettgeri	GCA_001874625.1_ASM187462v1_genomic.fna
913	Bifidobacterium pseudocatenulatum	GCA_001025215.1_ASM102521v1_genomic.fna
914	Neisseria elongata	GCA_000818035.1_ASM81803v1_genomic.fna
915	Neisseria sicca	GCA_002073715.1_ASM207371v1_genomic.fna

916	Filifactor alocis	GCA_000163895.2_ASM16389v2_genomic.fna
917	Capnocytophaga sputigena	GCA_002302395.1_ASM230239v1_genomic.fna
918	Lactobacillus paracasei	GCA_000014525.1_ASM1452v1_genomic.fna
919	Mobiluncus curtisii	GCA_000196535.1_ASM19653v1_genomic.fna
920	Bacteroides cellulosilyticus	GCA_001318345.1_ASM131834v1_genomic.fna
921	Helicobacter cinaedi	GCA_000349975.1_ASM34997v1_genomic.fna
922	Staphylococcus capitis	GCA_001028645.1_ASM102864v1_genomic.fna
923	Campylobacter gracilis	GCA_001190745.1_ASM119074v1_genomic.fna
924	Selenomonas sputigena	GCA_000208405.1_ASM20840v1_genomic.fna
925	Staphylococcus warneri	GCA_000332735.1_ASM33273v1_genomic.fna
926	Corynebacterium striatum	GCA_002156805.1_ASM215680v1_genomic.fna
927	Corynebacterium aurimucosum	GCA_000022905.1_ASM2290v1_genomic.fna
928	Bifidobacterium catenulatum	GCA_001025195.1_ASM102519v1_genomic.fna
929	Fusobacterium varium	GCA_002356455.1_ASM235645v1_genomic.fna
930	Rhodococcus hoagii	GCA_000196695.1_ASM19669v1_genomic.fna
931	Helicobacter bilis	GCA_001999985.1_ASM199998v1_genomic.fna
932	Erysipelothrix rhusiopathiae	GCA_000270085.1_ASM27008v1_genomic.fna
933	Lactobacillus amylolyticus	GCA_002075105.1_ASM207510v1_genomic.fna
934	Starkeya novella	GCA_000092925.1_ASM9292v1_genomic.fna
935	Klebsiella variicola	GCA_000025465.1_ASM2546v1_genomic.fna
936	Bacillus atrophaeus	GCA_002173495.1_ASM217349v1_genomic.fna
937	Segniliparus rotundus	GCA_000092825.1_ASM9282v1_genomic.fna
938	Desulfovibrio desulfuricans	GCA_000189295.2_ASM18929v2_genomic.fna
939	Syntrophothermus lipocalidus	GCA_000092405.1_ASM9240v1_genomic.fna
940	Marivirga tractuosa	GCA_000183425.1_ASM18342v1_genomic.fna
941	Arcanobacterium haemolyticum	GCA_000092365.1_ASM9236v1_genomic.fna
942	Desulfarculus baarsii	GCA_000143965.1_ASM14396v1_genomic.fna
943	Micromonospora aurantiaca	GCA_000145235.1_ASM14523v1_genomic.fna
944	Corynebacterium kroppenstedtii	GCA_000023145.1_ASM2314v1_genomic.fna
945	Flavobacteriaceae bacterium	GCA_000023725.1_ASM2372v1_genomic.fna
946	Pseudodesulfovibrio aespoeensis	GCA_000176915.2_ASM17691v2_genomic.fna
947	Thermaerobacter marianensis	GCA_000184705.1_ASM18470v1_genomic.fna
948	Syntrophobotulus glycolicus	GCA_000190635.1_ASM19063v1_genomic.fna
949	Leadbetterella byssophila	GCA_000166395.1_ASM16639v1_genomic.fna

950	Parachlamydia acanthamoebae	GCA_000253035.1_ASM25303v1_genomic.fna
951	Truepera radiovictrix	GCA_000092425.1_ASM9242v1_genomic.fna
952	Bacillus cellulosilyticus	GCA_000177235.2_ASM17723v2_genomic.fna
953	Acinetobacter nosocomialis	GCA_000814165.3_ASM81416v3_genomic.fna
954	Streptomyces griseus	GCA_000010605.1_ASM1060v1_genomic.fna
955	Desulfurispirillum indicum	GCA_000177635.2_ASM17763v2_genomic.fna
956	Rhodomicrobium vannielii	GCA_000166055.1_ASM16605v1_genomic.fna
957	Streptococcus oralis	GCA_000253155.1_ASM25315v1_genomic.fna
958	Aerococcus viridans	GCA_001543285.1_ASM154328v1_genomic.fna
959	Achromobacter xylosoxidans	GCA_001457475.1_NCTC10807_genomic.fna
960	Atopobium parvulum	GCA_000024225.1_ASM2422v1_genomic.fna
961	Frankia symbiont	GCA_000177615.2_ASM17761v2_genomic.fna
962	Zunongwangia profunda	GCA_000023465.1_ASM2346v1_genomic.fna
963	Thermoanaerobacter pseudethanolicus	GCA_000019085.1_ASM1908v1_genomic.fna
964	Acidovorax citrulli	GCA_000015325.1_ASM1532v1_genomic.fna
965	Magnetospirillum magneticum	GCA_000009985.1_ASM998v1_genomic.fna
966	Xanthomonas fuscans	GCA_000969685.1_ASM96968v1_genomic.fna
967	Pseudomonas syringae	GCA_000007805.1_ASM780v1_genomic.fna
968	Pseudomonas savastanoi	GCA_000012205.1_ASM1220v1_genomic.fna
969	Corynebacterium ammoniagenes	GCA_001941425.1_ASM194142v1_genomic.fna
970	Acinetobacter calcoaceticus	GCA_002055515.1_ASM205551v1_genomic.fna
971	Acinetobacter johnsonii	GCA_001484935.1_ASM148493v1_genomic.fna
972	Acinetobacter junii	GCA_001941805.1_ASM194180v1_genomic.fna
973	Methylotenera mobilis	GCA_000023705.1_ASM2370v1_genomic.fna
974	Ethanoligenens harbinense	GCA_000178115.2_ASM17811v2_genomic.fna
975	Corynebacterium resistens	GCA_000177535.2_ASM17753v2_genomic.fna
976	Vibrio fluvialis	GCA_001558415.1_ASM155841v1_genomic.fna
977	Pantoea stewartii	GCA_002082215.1_ASM208221v1_genomic.fna
978	Spirochaeta thermophila	GCA_000184345.2_ASM18434v2_genomic.fna
979	Cronobacter turicensis	GCA_000027065.2_ASM2706v1_genomic.fna
980	Methylotenera versatilis	GCA_000093025.1_ASM9302v1_genomic.fna
981	Bacillus smithii	GCA_001050115.1_ASM105011v1_genomic.fna
982	Pasteurella dagmatis	GCA_900186835.1_49950_F01_genomic.fna
983	Caldicellulosiruptor lactoaceticus	GCA_000193435.3_ASM19343v3_genomic.fna

984	Dehalogenimonas lykanthroporepellens	GCA_000143165.1_ASM14316v1_genomic.fna
985	Oceanithermus profundus	GCA_000183745.1_ASM18374v1_genomic.fna
986	Bacteroides salanitronis	GCA_000190575.1_ASM19057v1_genomic.fna
987	Thermodesulfatator indicus	GCA_000217795.1_ASM21779v1_genomic.fna
988	Vibrio mimicus	GCA_001558475.1_ASM155847v1_genomic.fna
989	Komagataeibacter xylinus	GCA_000550765.1_ASM55076v1_genomic.fna
990	Vibrio alginolyticus	GCA_000354175.2_ASM35417v2_genomic.fna
991	Nitrosococcus watsonii	GCA_000143085.1_ASM14308v1_genomic.fna
992	Caldicellulosiruptor obsidiansis	GCA_000145215.1_ASM14521v1_genomic.fna
993	Pediococcus acidilactici	GCA_001767275.1_ASM176727v1_genomic.fna
994	Vibrio coralliilyticus	GCA_000763535.2_ASM76353v2_genomic.fna
995	Grimontia hollisae	GCA_001558255.1_ASM155825v1_genomic.fna
996	Vibrio furnissii	GCA_000184325.1_ASM18432v1_genomic.fna
997	Pseudonocardia dioxanivorans	GCA_000196675.1_ASM19667v1_genomic.fna
998	Sulfobacillus acidophilus	GCA_000219855.1_ASM21985v1_genomic.fna
999	Parageobacillus thermoglucosidasius	GCA_001295365.1_ASM129536v1_genomic.fna
1000	Caldicellulosiruptor hydrothermalis	GCA_000166355.1_ASM16635v1_genomic.fna
1001	Caldicellulosiruptor owensensis	GCA_000166335.1_ASM16633v1_genomic.fna
1002	Leuconostoc kimchii	GCA_000092505.1_ASM9250v1_genomic.fna
1003	Corynebacterium pseudotuberculosis	GCA_000144675.1_ASM14467v1_genomic.fna
1004	Thermincola potens	GCA_000092945.1_ASM9294v1_genomic.fna
1005	Enterobacter asburiae	GCA_000224675.1_ASM22467v1_genomic.fna
1006	Clostridium lentocellum	GCA_000178835.2_ASM17883v2_genomic.fna
1007	Pseudothermotoga thermarum	GCA_000217815.1_ASM21781v1_genomic.fna
1008	Cellulophaga algicola	GCA_000186265.1_ASM18626v1_genomic.fna
1009	Veillonella parvula	GCA_000024945.1_ASM2494v1_genomic.fna
1010	Alicycliphilus denitrificans	GCA_000204645.1_ASM20464v1_genomic.fna
1011	Cupriavidus necator	GCA_000219215.1_ASM21921v1_genomic.fna
1012	Bacillus cytotoxicus	GCA_000017425.1_ASM1742v1_genomic.fna
1013	Borreliella bavariensis	GCA_000196215.1_ASM19621v1_genomic.fna
1014	Pseudomonas amygdali	GCA_002068135.1_ASM206813v1_genomic.fna
1015	Deinococcus proteolyticus	GCA_000190555.1_ASM19055v1_genomic.fna
1016	Bacteroides helcogenes	GCA_000186225.1_ASM18622v1_genomic.fna
1017	Xanthomonas campestris	GCA_000009165.1_ASM916v1_genomic.fna

1018	Acinetobacter pittii	GCA_000191145.1_ASM19114v1_genomic.fna
1019	Scardovia inopinata	GCA_001042695.1_ASM104269v1_genomic.fna
1020	Riemerella anatipestifer	GCA_000252855.1_ASM25285v1_genomic.fna
1021	Paludibacter propionicigenes	GCA_000183135.1_ASM18313v1_genomic.fna
1022	Lactobacillus amylovorus	GCA_000191545.1_ASM19154v1_genomic.fna
1023	Clostridium saccharolyticum	GCA_000144625.1_ASM14462v1_genomic.fna
1024	Mahella australiensis	GCA_000213255.1_ASM21325v1_genomic.fna
1025	Thermoanaerobacter wiegelii	GCA_000147695.3_ASM14769v3_genomic.fna
1026	Staphylococcus lugdunensis	GCA_000025085.1_ASM2508v1_genomic.fna
1027	Streptococcus constellatus	GCA_000463395.1_ASM46339v1_genomic.fna
1028	Parascardovia denticolens	GCA_001042675.1_ASM104267v1_genomic.fna
1029	Caldicellulosiruptor kristjanssonii	GCA_000166695.1_ASM16669v1_genomic.fna
1030	Coriobacterium glomerans	GCA_000195315.1_ASM19531v1_genomic.fna
1031	Niastella koreensis	GCA_000246855.1_ASM24685v1_genomic.fna
1032	Streptomyces violaceusniger	GCA_000147815.3_ASM14781v3_genomic.fna
1033	Halanaerobium hydrogeniformans	GCA_000166415.1_ASM16641v1_genomic.fna
1034	Pantoea ananatis	GCA_000025405.2_ASM2540v1_genomic.fna
1035	Streptococcus anginosus	GCA_000463505.1_ASM46350v1_genomic.fna
1036	Desulfomonile tiedjei	GCA_000266945.1_ASM26694v1_genomic.fna
1037	Acinetobacter haemolyticus	GCA_001932555.1_ASM193255v1_genomic.fna
1038	Mycoplasma suis	GCA_000179035.2_ASM17903v2_genomic.fna
1039	Deinococcus maricopensis	GCA_000186385.1_ASM18638v1_genomic.fna
1040	Odoribacter splanchnicus	GCA_000190535.1_ASM19053v1_genomic.fna
1041	Amycolatopsis mediterranei	GCA_000196835.1_ASM19683v1_genomic.fna
1042	Sulfuricurvum kujiense	GCA_000183725.1_ASM18372v1_genomic.fna
1043	Pantoea vagans	GCA_000148935.1_ASM14893v1_genomic.fna
1044	Lactobacillus sanfranciscensis	GCA_000225325.1_ASM22532v1_genomic.fna
1045	Lysinibacillus fusiformis	GCA_000724775.3_ASM72477v3_genomic.fna
1046	Streptococcus gallolyticus	GCA_002000985.1_ASM200098v1_genomic.fna
1047	Roseburia hominis	GCA_000225345.1_ASM22534v1_genomic.fna
1048	Intrasporangium calvum	GCA_000184685.1_ASM18468v1_genomic.fna
1049	Waddlia chondrophila	GCA_000092785.1_ASM9278v1_genomic.fna
1050	Acinetobacter oleivorans	GCA_000196795.1_ASM19679v1_genomic.fna
1051	Flexistipes sinusarabici	GCA_000218625.1_ASM21862v1_genomic.fna

1052	Marinobacter adhaerens	GCA_000166295.1_ASM16629v1_genomic.fna
1053	Deferribacter desulfuricans	GCA_000010985.1_ASM1098v1_genomic.fna
1054	Thermus scotoductus	GCA_000187005.1_ASM18700v1_genomic.fna
1055	Nitrospira defluvii	GCA_000196815.1_ASM19681v1_genomic.fna
1056	Mycoplasma putrefaciens	GCA_000224105.1_ASM22410v1_genomic.fna
1057	Taylorella equigenitalis	GCA_000276685.1_ASM27668v1_genomic.fna
1058	Treponema caldarium	GCA_000219725.1_ASM21972v1_genomic.fna
1059	Thermodesulfobium narugense	GCA_000212395.1_ASM21239v1_genomic.fna
1060	Desulfotomaculum nigrificans	GCA_000214435.1_ASM21443v1_genomic.fna
1061	Nitratifractor salsuginis	GCA_000186245.1_ASM18624v1_genomic.fna
1062	Sphingobium japonicum	GCA_000091125.1_ASM9112v1_genomic.fna
1063	Rubinisphaera brasiliensis	GCA_000165715.3_ASM16571v3_genomic.fna
1064	Streptomyces bingchenggensis	GCA_000092385.1_ASM9238v1_genomic.fna
1065	Leptospira santarosai	GCA_000313175.2_ASM31317v2_genomic.fna
1066	Fluviicola taffensis	GCA_000194605.1_ASM19460v1_genomic.fna
1067	Desulfotomaculum ruminis	GCA_000215085.1_ASM21508v1_genomic.fna
1068	Granulicella mallensis	GCA_000178955.2_ASM17895v2_genomic.fna
1069	Sphaerochaeta globosa	GCA_000190435.1_ASM19043v1_genomic.fna
1070	Ketogulonicigenium vulgare	GCA_000223375.1_ASM22337v1_genomic.fna
1071	Citrobacter freundii	GCA_000648515.1_ASM64851v1_genomic.fna
1072	Eggerthella lenta	GCA_000024265.1_ASM2426v1_genomic.fna
1073	Sphaerochaeta coccoides	GCA_000208385.1_ASM20838v1_genomic.fna
1074	Hippea maritima	GCA_000194135.1_ASM19413v1_genomic.fna
1075	Haliscomenobacter hydrossis	GCA_000212735.1_ASM21273v1_genomic.fna
1076	Brachyspira pilosicoli	GCA_000325665.1_ASM32566v1_genomic.fna
1077	Streptococcus parasanguinis	GCA_000164675.2_ASM16467v2_genomic.fna
1078	Terriglobus saanensis	GCA_000179915.2_ASM17991v2_genomic.fna
1079	Mesotoga prima	GCA_000147715.3_ASM14771v3_genomic.fna
1080	Runella slithyformis	GCA_000218895.1_ASM21889v1_genomic.fna
1081	Pseudopropionibacterium propionicum	GCA_000277715.1_ASM27771v1_genomic.fna
1082	Prevotella denticola	GCA_000193395.1_ASM19339v1_genomic.fna
1083	Pseudopedobacter saltans	GCA_000190735.2_ASM19073v2_genomic.fna
1084	Thermovibrio ammonificans	GCA_000185805.1_ASM18580v1_genomic.fna
1085	Calditerrivibrio nitroreducens	GCA_000183405.1_ASM18340v1_genomic.fna

1086	Isoptericola variabilis	GCA_000215105.1_ASM21510v1_genomic.fna
1087	Pseudomonas fulva	GCA_000213805.1_ASM21380v1_genomic.fna
1088	Sphingobium chlorophenolicum	GCA_000147835.3_ASM14783v3_genomic.fna
1089	Capnocytophaga canimorsus	GCA_000220625.1_ASM22062v1_genomic.fna
1090	Thermoanaerobacterium xylanolyticum	GCA_000189775.3_ASM18977v3_genomic.fna
1091	Haemophilus parainfluenzae	GCA_000210895.1_ASM21089v1_genomic.fna
1092	Weeksella virosa	GCA_000189415.1_ASM18941v1_genomic.fna
1093	Rahnella aquatilis	GCA_000255535.1_ASM25553v1_genomic.fna
1094	Vibrio tubiashii	GCA_000772105.1_ASM77210v1_genomic.fna
1095	Cellulophaga lytica	GCA_000190595.1_ASM19059v1_genomic.fna
1096	Treponema succinifaciens	GCA_000195275.1_ASM19527v1_genomic.fna
1097	Marinithermus hydrothermalis	GCA_000195335.1_ASM19533v1_genomic.fna
1098	Aerococcus urinae	GCA_001543175.1_ASM154317v1_genomic.fna
1099	Veillonella atypica	GCA_002082765.1_ASM208276v1_genomic.fna
1100	Vibrio scophthalmi	GCA_001685465.1_ASM168546v1_genomic.fna
1101	Desulfurobacterium thermolithotrophum	GCA_000191045.1_ASM19104v1_genomic.fna
1102	Porphyromonas asaccharolytica	GCA_000212375.1_ASM21237v1_genomic.fna
1103	Capnocytophaga ochracea	GCA_000023285.1_ASM2328v1_genomic.fna
1104	Actinomyces oris	GCA_001553935.1_ASM155393v1_genomic.fna
1105	Sinorhizobium fredii	GCA_000018545.1_ASM1854v1_genomic.fna
1106	Vibrio anguillarum	GCA_000217675.1_ASM21767v1_genomic.fna
1107	Anaerococcus prevotii	GCA_000024105.1_ASM2410v1_genomic.fna
1108	Mycoplasma hominis	GCA_000085865.1_ASM8586v1_genomic.fna
1109	Staphylococcus simulans	GCA_001559115.1_ASM155911v1_genomic.fna
1110	Myroides odoratimimus	GCA_001481655.1_ASM148165v1_genomic.fna
1111	Eubacterium limosum	GCA_000807675.2_ASM80767v2_genomic.fna
1112	Muricauda ruestringensis	GCA_000224085.1_ASM22408v1_genomic.fna
1113	Marinomonas posidonica	GCA_000214215.1_ASM21421v1_genomic.fna
1114	Desulfotomaculum kuznetsovii	GCA_000214705.1_ASM21470v1_genomic.fna
1115	Streptococcus parauberis	GCA_000213825.1_ASM21382v1_genomic.fna
1116	Leuconostoc lactis	GCA_001698145.1_ASM169814v1_genomic.fna
1117	Marinomonas mediterranea	GCA_000192865.1_ASM19286v1_genomic.fna
1118	Thiomicrospira cyclica	GCA_000214825.1_ASM21482v1_genomic.fna
1119	Acetobacter aceti	GCA_002005445.1_ASM200544v1_genomic.fna

1120	gamma proteobacterium	GCA_000198515.1_ASM19851v1_genomic.fna
1121	Desulfovibrio africanus	GCA_000212675.2_ASM21267v2_genomic.fna
1122	Rhodothermus marinus	GCA_000024845.1_ASM2484v1_genomic.fna
1123	Enterobacter hormaechei	GCA_001729725.1_ASM172972v1_genomic.fna
1124	Streptococcus cristatus	GCA_000385925.1_ASM38592v1_genomic.fna
1125	Streptococcus pseudopneumoniae	GCA_000221985.1_ASM22198v1_genomic.fna
1126	Glutamicibacter arilaitensis	GCA_000197735.1_ASM19773v1_genomic.fna
1127	Desulfobacca acetoxidans	GCA_000195295.1_ASM19529v1_genomic.fna
1128	Caldicellulosiruptor kronotskyensis	GCA_000166775.1_ASM16677v1_genomic.fna
1129	Treponema brennaborense	GCA_000212415.1_ASM21241v1_genomic.fna
1130	Methylomonas methanica	GCA_000214665.1_ASM21466v1_genomic.fna
1131	Thermobacillus composti	GCA_000227705.3_ASM22770v3_genomic.fna
1132	Plautia stali	GCA_000180175.2_ASM18017v2_genomic.fna
1133	Weissella cibaria	GCA_001308145.2_ASM130814v2_genomic.fna
1134	Desulfotomaculum gibsoniae	GCA_000233715.3_ASM23371v3_genomic.fna
1135	Xanthomonas vesicatoria	GCA_001908725.1_ASM190872v1_genomic.fna
1136	Xanthomonas perforans	GCA_000192045.3_ASM19204v3_genomic.fna
1137	Anaerolinea thermophila	GCA_000199675.1_ASM19967v1_genomic.fna
1138	Paraburkholderia rhizoxinica	GCA_000198775.1_ASM19877v1_genomic.fna
1139	Leuconostoc gelidum	GCA_000196855.1_ASM19685v1_genomic.fna
1140	Sanguibacter keddieii	GCA_000024925.1_ASM2492v1_genomic.fna
1141	Gordonia bronchialis	GCA_000024785.1_ASM2478v1_genomic.fna
1142	Haliangium ochraceum	GCA_000024805.1_ASM2480v1_genomic.fna
1143	Sulfurospirillum deleyianum	GCA_000024885.1_ASM2488v1_genomic.fna
1144	Streptobacillus moniliformis	GCA_000024565.1_ASM2456v1_genomic.fna
1145	Sebaldella termitidis	GCA_000024405.1_ASM2440v1_genomic.fna
1146	Thermomonospora curvata	GCA_000024385.1_ASM2438v1_genomic.fna
1147	Thermanaerovibrio acidaminovorans	GCA_000024905.1_ASM2490v1_genomic.fna
1148	Xylanimonas cellulosilytica	GCA_000024965.1_ASM2496v1_genomic.fna
1149	Sphaerobacter thermophilus	GCA_000024985.1_ASM2498v1_genomic.fna
1150	Thermobaculum terrenum	GCA_000025005.1_ASM2500v1_genomic.fna
1151	Streptosporangium roseum	GCA_000024865.1_ASM2486v1_genomic.fna
1152	Spirosoma linguale	GCA_000024525.1_ASM2452v1_genomic.fna
1153	Kribbella flavida	GCA_000024345.1_ASM2434v1_genomic.fna

1154	Geodermatophilus obscurus	GCA_000025345.1_ASM2534v1_genomic.fna
1155	Denitrovibrio acetiphilus	GCA_000025725.1_ASM2572v1_genomic.fna
1156	Meiothermus ruber	GCA_000024425.1_ASM2442v1_genomic.fna
1157	Stackebrandtia nassauensis	GCA_000024545.1_ASM2454v1_genomic.fna
1158	Planctopirus limnophila	GCA_000092105.1_ASM9210v1_genomic.fna
1159	Brachyspira murdochii	GCA_000092845.1_ASM9284v1_genomic.fna
1160	Cellulomonas flavigena	GCA_000092865.1_ASM9286v1_genomic.fna
1161	Tsukamurella paurometabola	GCA_000092225.1_ASM9222v1_genomic.fna
1162	Thermobispora bispora	GCA_000092645.1_ASM9264v1_genomic.fna
1163	Nocardiopsis dassonvillei	GCA_000092985.1_ASM9298v1_genomic.fna
1164	Brachybacterium faecium	GCA_000023405.1_ASM2340v1_genomic.fna
1165	Actinosynnema mirum	GCA_000023245.1_ASM2324v1_genomic.fna
1166	Cryptobacterium curtum	GCA_000023845.1_ASM2384v1_genomic.fna
1167	Beutenbergia cavernae	GCA_000023105.1_ASM2310v1_genomic.fna
1168	Dyadobacter fermentans	GCA_000023125.1_ASM2312v1_genomic.fna
1169	Slackia heliotrinireducens	GCA_000023885.1_ASM2388v1_genomic.fna
1170	Jonesia denitrificans	GCA_000024065.1_ASM2406v1_genomic.fna
1171	Meiothermus silvanus	GCA_000092125.1_ASM9212v1_genomic.fna
1172	Kytococcus sedentarius	GCA_000023925.1_ASM2392v1_genomic.fna
1173	Catenulispora acidiphila	GCA_000024025.1_ASM2402v1_genomic.fna
1174	Desulfotomaculum acetoxidans	GCA_000024205.1_ASM2420v1_genomic.fna
1175	Pedobacter heparinus	GCA_000023825.1_ASM2382v1_genomic.fna
1176	Chitinophaga pinensis	GCA_000024005.1_ASM2400v1_genomic.fna
1177	Desulfohalobium retbaense	GCA_000024325.1_ASM2432v1_genomic.fna
1178	Kangiella koreensis	GCA_000024085.1_ASM2408v1_genomic.fna
1179	Leptotrichia buccalis	GCA_000023905.1_ASM2390v1_genomic.fna
1180	Acidimicrobium ferrooxidans	GCA_000023265.1_ASM2326v1_genomic.fna
1181	Desulfomicrobium baculatum	GCA_000023225.1_ASM2322v1_genomic.fna
1182	Nakamurella multipartita	GCA_000024365.1_ASM2436v1_genomic.fna
1183	Klebsiella aerogenes	GCA_000215745.1_ASM21574v1_genomic.fna
1184	Saccharomonospora viridis	GCA_000023865.1_ASM2386v1_genomic.fna
1185	Desulfitobacterium dehalogenans	GCA_000243155.3_ASM24315v3_genomic.fna
1186	Helicobacter felis	GCA_000200595.1_ASM20059v1_genomic.fna
1187	Staphylococcus pseudintermedius	GCA_000185885.1_ASM18588v1_genomic.fna

1188	Desulfitobacterium metallireducens	GCA_000231405.3_ASM23140v3_genomic.fna
1189	Planococcus donghaensis	GCA_001687665.2_ASM168766v2_genomic.fna
1190	Acidithiobacillus ferrivorans	GCA_000214095.3_ASM21409v3_genomic.fna
1191	Acetobacter pomorum	GCA_002456135.1_ASM245613v1_genomic.fna
1192	Desulfitobacterium dichloroeliminans	GCA_000243135.3_ASM24313v3_genomic.fna
1193	Desulfosporosinus acidiphilus	GCA_000255115.3_ASM25511v3_genomic.fna
1194	Xanthomonas gardneri	GCA_001908775.1_ASM190877v1_genomic.fna
1195	Corynebacterium ulcerans	GCA_000215645.1_ASM21564v1_genomic.fna
1196	Marichromatium purpuratum	GCA_000224005.3_ASM22400v3_genomic.fna
1197	Bartonella clarridgeiae	GCA_000253015.1_ASM25301v1_genomic.fna
1198	Microbacterium testaceum	GCA_000202635.1_ASM20263v1_genomic.fna
1199	Verrucosispora maris	GCA_000204155.1_ASM20415v1_genomic.fna
1200	Chlamydophila pecorum	GCA_000204135.1_ASM20413v1_genomic.fna
1201	Vibrio rotiferianus	GCA_002214395.1_ASM221439v1_genomic.fna
1202	Hoyosella subflava	GCA_000214175.1_ASM21417v1_genomic.fna
1203	Aeromonas veronii	GCA_000204115.1_ASM20411v1_genomic.fna
1204	Terriglobus roseus	GCA_000265425.1_ASM26542v1_genomic.fna
1205	Polymorphum gilvum	GCA_000192745.1_ASM19274v1_genomic.fna
1206	Acidiphilium multivorum	GCA_000202835.1_ASM20283v1_genomic.fna
1207	Paenibacillus mucilaginosus	GCA_000250655.1_ASM25065v1_genomic.fna
1208	Burkholderia gladioli	GCA_000959725.1_ASM95972v1_genomic.fna
1209	Mycobacterium abscessus	GCA_000758225.1_ASM75822v1_genomic.fna
1210	Echinicola vietnamensis	GCA_000325705.1_ASM32570v1_genomic.fna
1211	Pseudomonas brassicacearum	GCA_000194805.1_ASM19480v1_genomic.fna
1212	Gallibacterium anatis	GCA_000209675.1_ASM20967v1_genomic.fna
1213	Owenweeksia hongkongensis	GCA_000236705.1_ASM23670v1_genomic.fna
1214	Rickettsia amblyommatis	GCA_001273795.1_ASM127379v1_genomic.fna
1215	Melissococcus plutonius	GCA_000747585.1_ASM74758v1_genomic.fna
1216	Microlunatus phosphovorus	GCA_000270245.1_ASM27024v1_genomic.fna
1217	Haemophilus pittmaniae	GCA_900186995.1_50465_A02_genomic.fna
1218	Advenella mimigardefordensis	GCA_000521505.1_ASM52150v1_genomic.fna
1219	Rickettsia heilongjiangensis	GCA_000221205.1_ASM22120v1_genomic.fna
1220	Lactobacillus kefiranofaciens	GCA_000214785.1_ASM21478v1_genomic.fna
1221	Streptomyces xinghaiensis	GCA_000220705.2_ASM22070v2_genomic.fna

1222	Streptococcus pasteurianus	GCA_000270165.1_ASM27016v1_genomic.fna
1223	Mycobacterium colombiense	GCA_002105755.1_ASM210575v1_genomic.fna
1224	Brevibacillus laterosporus	GCA_000219535.3_ASM21953v3_genomic.fna
1225	Brachyspira intermedia	GCA_000223215.1_ASM22321v1_genomic.fna
1226	Weissella koreensis	GCA_000219805.1_ASM21980v1_genomic.fna
1227	Neisseria weaveri	GCA_900086555.1_34903_D01_genomic.fna
1228	Mycobacterium africanum	GCA_000253355.1_ASM25335v1_genomic.fna
1229	Pantoea agglomerans	GCA_001709315.1_ASM170931v1_genomic.fna
1230	Cyclobacterium marinum	GCA_000222485.1_ASM22248v1_genomic.fna
1231	Mycobacterium canettii	GCA_000253375.1_ASM25337v1_genomic.fna
1232	Burkholderia cepacia	GCA_001411495.1_ASM141149v1_genomic.fna
1233	Paenibacillus riograndensis	GCA_000981585.1_SBR5_T__genomic.fna
1234	Borreliella bissettii	GCA_000222305.1_ASM22230v1_genomic.fna
1235	Vibrio nigripulchritudo	GCA_000801275.1_ASM80127v1_genomic.fna
1236	Desulfosporosinus meridiei	GCA_000231385.3_ASM23138v3_genomic.fna
1237	Fervidobacterium pennivorans	GCA_000235405.3_ASM23540v3_genomic.fna
1238	Xanthomonas axonopodis	GCA_000225915.1_ASM22591v1_genomic.fna
1239	Nitrospirillum amazonense	GCA_000730165.2_ASM73016v2_genomic.fna
1240	Xanthomonas sacchari	GCA_000815185.1_ASM81518v1_genomic.fna
1241	Tetragenococcus halophilus	GCA_000283615.1_ASM28361v1_genomic.fna
1242	Mycoplasma yeatsii	GCA_000875755.1_ASM87575v1_genomic.fna
1243	Enterococcus hirae	GCA_000271405.2_ASM27140v2_genomic.fna
1244	Bacillus methanolicus	GCA_000724485.1_ASM72448v1_genomic.fna
1245	Ornithobacterium rhinotracheale	GCA_000265465.1_ASM26546v1_genomic.fna
1246	Hafnia alvei	GCA_000597785.2_ASM59778v2_genomic.fna
1247	Streptomyces venezuelae	GCA_001443625.1_ASM144362v1_genomic.fna
1248	Lactobacillus pentosus	GCA_002211885.1_ASM221188v1_genomic.fna
1249	Lactobacillus acidipiscis	GCA_900116935.1_Chromosome_genomic.fna
1250	Rhodococcus aetherivorans	GCA_000982715.1_ASM98271v1_genomic.fna
1251	Lactobacillus curvatus	GCA_002224425.1_ASM222442v1_genomic.fna
1252	Staphylococcus sciuri	GCA_002209165.1_ASM220916v1_genomic.fna
1253	Proteus vulgaris	GCA_001281565.1_ASM128156v1_genomic.fna
1254	Leptospirillum ferrooxidans	GCA_000284315.1_ASM28431v1_genomic.fna
1255	Tatumella citrea	GCA_002163585.1_ASM216358v1_genomic.fna

1256	Paracoccus aminophilus	GCA_000444995.1_ASM44499v1_genomic.fna
1257	Acetomicrobium mobile	GCA_000266925.1_ASM26692v1_genomic.fna
1258	Solitalea canadensis	GCA_000242635.3_ASM24263v3_genomic.fna
1259	Saprospira grandis	GCA_000250635.1_ASM25063v1_genomic.fna
1260	Niabella soli	GCA_000243115.3_ASM24311v3_genomic.fna
1261	Deinococcus peraridilitoris	GCA_000317835.1_ASM31783v1_genomic.fna
1262	Micavibrio aeruginosavorus	GCA_000226315.1_ASM22631v1_genomic.fna
1263	Chloracidobacterium thermophilum	GCA_000226295.1_ASM22629v1_genomic.fna
1264	Flavobacterium branchiophilum	GCA_000253275.1_ASM25327v1_genomic.fna
1265	Singulisphaera acidiphila	GCA_000242455.3_ASM24245v3_genomic.fna
1266	Pediococcus damnosus	GCA_001611135.1_ASM161113v1_genomic.fna
1267	Glaciecola nitratireducens	GCA_000226565.1_ASM22656v1_genomic.fna
1268	Taylorella asinigenitalis	GCA_000226625.1_ASM22662v1_genomic.fna
1269	Morganella morganii	GCA_000286435.2_ASM28643v2_genomic.fna
1270	Cutibacterium avidum	GCA_000367205.1_ASM36720v1_genomic.fna
1271	Komagataeibacter europaeus	GCA_002173515.1_ASM217351v1_genomic.fna
1272	Thiomicrospira aerophila	GCA_000227665.3_ASM22766v3_genomic.fna
1273	Thiocystis violascens	GCA_000227745.3_ASM22774v3_genomic.fna
1274	Tannerella forsythia	GCA_000238215.1_ASM23821v1_genomic.fna
1275	Prevotella intermedia	GCA_001953955.1_ASM195395v1_genomic.fna
1276	Streptomyces ambofaciens	GCA_001267885.1_ASM126788v1_genomic.fna
1277	Actinomyces naeslundii	GCA_002355915.1_ASM235591v1_genomic.fna
1278	Thermodesulfobacterium commune	GCA_000734015.1_ASM73401v1_genomic.fna
1279	Clostridium pasteurianum	GCA_000807255.1_ASM80725v1_genomic.fna
1280	Mycobacterium microti	GCA_001544815.1_ASM154481v1_genomic.fna
1281	Wolbachia endosymbiont	GCA_000530755.1_W_O_volvulus_Cameroon_v3_genomic.fna
1282	Legionella hackeliae	GCA_000953655.1_LHA_genomic.fna
1283	Azospirillum brasilense	GCA_001315015.1_ASM131501v1_genomic.fna
1284	Rhizobium tropici	GCA_000330885.1_ASM33088v1_genomic.fna
1285	Mycobacterium chelonae	GCA_001632805.1_ASM163280v1_genomic.fna
1286	Vibrio campbellii	GCA_000017705.1_ASM1770v1_genomic.fna
1287	Maize bushy	GCA_001712875.1_ASM171287v1_genomic.fna
1288	Rickettsia slovaca	GCA_000237845.1_ASM23784v1_genomic.fna
1289	Staphylococcus xylosus	GCA_000706685.1_ASM70668v1_genomic.fna

1290	Pseudomonas pseudoalcaligenes	GCA_000297075.2_Ppseudo_final_genomic.fna
1291	Acidaminococcus intestini	GCA_000230275.1_ASM23027v1_genomic.fna
1292	Nonlabens dokdonensis	GCA_000332115.1_ASM33211v1_genomic.fna
1293	Thermovirga lienii	GCA_000233775.1_ASM23377v1_genomic.fna
1294	Shimwellia blattae	GCA_000262305.1_ASM26230v1_genomic.fna
1295	Aerococcus christensenii	GCA_001543105.1_ASM154310v1_genomic.fna
1296	Chroococcidiopsis thermalis	GCA_000317125.1_ASM31712v1_genomic.fna
1297	Desulfobacula toluolica	GCA_000307105.1_ASM30710v1_genomic.fna
1298	Rickettsia japonica	GCA_000283595.1_ASM28359v1_genomic.fna
1299	Anaerostipes hadrus	GCA_001998765.1_ASM199876v1_genomic.fna
1300	Dechlorosoma suillum	GCA_000236665.1_ASM23666v1_genomic.fna
1301	Rubrobacter radiotolerans	GCA_000661895.1_ASM66189v1_genomic.fna
1302	Flavonifractor plautii	GCA_001688625.2_ASM168862v2_genomic.fna
1303	Cyanobacterium stanieri	GCA_000317655.1_ASM31765v1_genomic.fna
1304	Alistipes finegoldii	GCA_000265365.1_ASM26536v1_genomic.fna
1305	Bacillus simplex	GCA_001578185.1_ASM157818v1_genomic.fna
1306	Mycobacterium indicus	GCA_000298095.1_ASM29809v1_genomic.fna
1307	Actinobacillus suis	GCA_000739435.1_ASM73943v1_genomic.fna
1308	Amphibacillus xylanus	GCA_000307165.1_ASM30716v1_genomic.fna
1309	Pediococcus claussenii	GCA_000237995.2_ASM23799v2_genomic.fna
1310	Planktomarina temperata	GCA_000738435.1_ASM73843v1_genomic.fna
1311	Caldisericum exile	GCA_000284335.1_ASM28433v1_genomic.fna
1312	Oscillibacter valericigenes	GCA_000283575.1_ASM28357v1_genomic.fna
1313	Anabaena cylindrica	GCA_000317695.1_ASM31769v1_genomic.fna
1314	Pseudomonas viridiflava	GCA_900184295.1_Chr_1_genomic.fna
1315	Spiroplasma eriocheiris	GCA_001029265.1_ASM102926v1_genomic.fna
1316	Mycoplasma bovoculi	GCA_000524555.1_ASM52455v1_genomic.fna
1317	Mycoplasma flocculare	GCA_000815065.1_ASM81506v1_genomic.fna
1318	Moraxella bovoculi	GCA_000988605.1_ASM98860v1_genomic.fna
1319	Deinococcus gobiensis	GCA_000252445.1_ASM25244v1_genomic.fna
1320	Aequorivita sublithincola	GCA_000265385.1_ASM26538v1_genomic.fna
1321	Halobacteroides halobius	GCA_000328625.1_ASM32862v1_genomic.fna
1322	Burkholderia plantarii	GCA_001411805.1_ASM141180v1_genomic.fna
1323	Spiroplasma mirum	GCA_000565195.1_ASM56519v1_genomic.fna

1324	Sphaerochaeta pleomorpha	GCA_000236685.1_ASM23668v1_genomic.fna
1325	Sphingobium indicum	GCA_000264945.2_ASM26494v2_genomic.fna
1326	Herbaspirillum frisingense	GCA_002025725.1_ASM202572v1_genomic.fna
1327	Xanthomonas hortorum	GCA_002285515.1_ASM228551v1_genomic.fna
1328	Belliella baltica	GCA_000265405.1_ASM26540v1_genomic.fna
1329	Halobacillus halophilus	GCA_000284515.1_ASM28451v1_genomic.fna
1330	Turneriella parva	GCA_000266885.1_ASM26688v1_genomic.fna
1331	Actinotignum schaalii	GCA_000724605.1_ASM72460v1_genomic.fna
1332	Caldithrix abyssi	GCA_001886815.1_ASM188681v1_genomic.fna
1333	Spirochaeta africana	GCA_000242595.3_ASM24259v3_genomic.fna
1334	Desulfomicrobium orale	GCA_001553625.1_ASM155362v1_genomic.fna
1335	Eubacterium sulci	GCA_001189495.1_ASM118949v1_genomic.fna
1336	Mycobacterium chubuense	GCA_000266905.1_ASM26690v1_genomic.fna
1337	Leclercia adecarboxylata	GCA_001518835.1_ASM151883v1_genomic.fna
1338	Mycobacterium rhodesiae	GCA_000230895.3_ASM23089v3_genomic.fna
1339	Kitasatospora setae	GCA_000269985.1_ASM26998v1_genomic.fna
1340	Emticicia oligotrophica	GCA_000263195.1_ASM26319v1_genomic.fna
1341	Alcanivorax dieselolei	GCA_000300005.1_ASM30000v1_genomic.fna
1342	Acetobacterium woodii	GCA_000247605.1_ASM24760v1_genomic.fna
1343	Thioflavicoccus mobilis	GCA_000327045.1_ASM32704v1_genomic.fna
1344	Clostridium saccharoperbutylacetonicum	GCA_000340885.1_ASM34088v1_genomic.fna
1345	Ignavibacterium album	GCA_000258405.1_ASM25840v1_genomic.fna
1346	Streptomyces cattleya	GCA_000240165.1_ASM24016v1_genomic.fna
1347	Desulfosporosinus orientis	GCA_000235605.1_ASM23560v1_genomic.fna
1348	Dehalobacter restrictus	GCA_000512895.1_ASM51289v1_genomic.fna
1349	Neorhizobium galegae	GCA_000731315.1_RG540_Ch_pA_genomic.fna
1350	Enterococcus durans	GCA_001267395.1_ASM126739v1_genomic.fna
1351	Campylobacter lanienae	GCA_002139935.1_ASM213993v1_genomic.fna
1352	Campylobacter insulaenigrae	GCA_000816185.1_ASM81618v1_genomic.fna
1353	Campylobacter avium	GCA_002238335.1_ASM223833v1_genomic.fna
1354	Campylobacter helveticus	GCA_002080395.1_ASM208039v1_genomic.fna
1355	Campylobacter cuniculorum	GCA_002104335.1_ASM210433v1_genomic.fna
1356	Campylobacter hyointestinalis	GCA_001643955.1_ASM164395v1_genomic.fna
1357	Campylobacter sputorum	GCA_002220735.1_ASM222073v1_genomic.fna

1358	Campylobacter volucris	GCA_000816345.1_ASM81634v1_genomic.fna
1359	Rhodococcus pyridinivorans	GCA_000511305.1_ASM51130v1_genomic.fna
1360	Flavobacterium columnare	GCA_000240075.2_ASM24007v2_genomic.fna
1361	Pseudoxanthomonas spadix	GCA_000233915.4_ASM23391v4_genomic.fna
1362	Leptospirillum ferriphilum	GCA_000299235.1_ASM29923v1_genomic.fna
1363	Leuconostoc carnosum	GCA_000300135.1_ASM30013v1_genomic.fna
1364	Enterococcus mundtii	GCA_000504125.1_ASM50412v1_genomic.fna
1365	Bacillus horikoshii	GCA_002157855.1_ASM215785v1_genomic.fna
1366	Streptococcus lutetiensis	GCA_000441535.1_ASM44153v1_genomic.fna
1367	Herbaspirillum rubrisubalbicans	GCA_001483945.1_ASM148394v1_genomic.fna
1368	Clostridium clariflavum	GCA_000237085.1_ASM23708v1_genomic.fna
1369	Pelagibacterium halotolerans	GCA_000230555.1_ASM23055v1_genomic.fna
1370	Mesorhizobium amorphae	GCA_001686985.1_ASM168698v1_genomic.fna
1371	Francisella persica	GCA_001653955.1_ASM165395v1_genomic.fna
1372	Exiguobacterium antarcticum	GCA_000299435.1_ASM29943v1_genomic.fna
1373	Mycoplasma wenyonii	GCA_000277795.1_ASM27779v1_genomic.fna
1374	Carnobacterium maltaromaticum	GCA_000317975.2_ASM31797v2_genomic.fna
1375	Paenibacillus peoriae	GCA_001272655.2_ASM127265v2_genomic.fna
1376	Francisella noatunensis	GCA_000262205.1_ASM26220v1_genomic.fna
1377	Francisella hispaniensis	GCA_001885235.1_ASM188523v1_genomic.fna
1378	Piscirickettsia salmonis	GCA_000300295.4_ASM30029v4_genomic.fna
1379	Methylomicrobium alcaliphilum	GCA_000968535.1_ASM96853v1_genomic.fna
1380	Streptomyces xiamenensis	GCA_000993785.2_ASM99378v2_genomic.fna
1381	Flavobacterium indicum	GCA_000455605.1_GPTSA100-9_genomic.fna
1382	Leisingera methylohalidivorans	GCA_000511355.1_ASM51135v1_genomic.fna
1383	Thermocrinis ruber	GCA_000512735.1_ASM51273v1_genomic.fna
1384	Rickettsia philipii	GCA_000283995.1_ASM28399v1_genomic.fna
1385	Rickettsia parkeri	GCA_000284195.1_ASM28419v1_genomic.fna
1386	Rickettsia australis	GCA_000284155.1_ASM28415v1_genomic.fna
1387	Rickettsia montanensis	GCA_000284175.1_ASM28417v1_genomic.fna
1388	Rickettsia rhipicephali	GCA_000284075.1_ASM28407v1_genomic.fna
1389	Bartonella vinsonii	GCA_000341385.1_ASM34138v1_genomic.fna
1390	Bartonella australis	GCA_000341355.1_ASM34135v1_genomic.fna
1391	Thermoanaerobacterium saccharolyticum	GCA_000307585.2_ASM30758v2_genomic.fna

1392	Rhodoferax antarcticus	GCA_001955735.1_ASM195573v1_genomic.fna
1393	Mycoplasma haemocanis	GCA_000238995.1_ASM23899v1_genomic.fna
1394	Desulfurella acetivorans	GCA_000517565.1_ASM51756v1_genomic.fna
1395	Corynebacterium casei	GCA_000550785.1_ASM55078v1_genomic.fna
1396	Tistrella mobilis	GCA_000264455.2_ASM26445v2_genomic.fna
1397	Pseudomonas psychrotolerans	GCA_001913135.1_ASM191313v1_genomic.fna
1398	Gordonia polyisoprenivorans	GCA_000247715.1_ASM24771v1_genomic.fna
1399	Rhodococcus rhodochrous	GCA_900187265.1_52295_G01_genomic.fna
1400	Staphylococcus hyicus	GCA_000816085.1_ASM81608v1_genomic.fna
1401	Elizabethkingia anophelis	GCA_000495935.2_ASM49593v2_genomic.fna
1402	Simiduia agarivorans	GCA_000305785.2_ASM30578v2_genomic.fna
1403	Mycoplasma canis	GCA_001553195.1_ASM155319v1_genomic.fna
1404	Pseudoalteromonas arctica	GCA_000238395.4_ASM23839v4_genomic.fna
1405	Pseudoalteromonas marina	GCA_002407085.1_ASM240708v1_genomic.fna
1406	Pseudoalteromonas piscicida	GCA_002208135.1_ASM220813v1_genomic.fna
1407	Pseudoalteromonas rubra	GCA_001482385.1_ASM148238v1_genomic.fna
1408	Pseudoalteromonas spongiae	GCA_000238255.4_ASM23825v4_genomic.fna
1409	Citrobacter farmeri	GCA_002249995.1_ASM224999v1_genomic.fna
1410	Citrobacter werkmanii	GCA_002025225.1_ASM202522v1_genomic.fna
1411	Novosphingobium pentaromativorans	GCA_000767465.1_ASM76746v1_genomic.fna
1412	Pseudomonas monteilii	GCA_001534745.1_ASM153474v1_genomic.fna
1413	Rothia aeria	GCA_002355935.1_ASM235593v1_genomic.fna
1414	Nocardia cyriacigeorgica	GCA_000284035.1_ASM28403v1_genomic.fna
1415	Gottschalkia acidurici	GCA_000299355.1_ASM29935v1_genomic.fna
1416	Rhizobium phaseoli	GCA_000172715.2_ASM17271v2_genomic.fna
1417	Cupriavidus basilensis	GCA_000832305.1_ASM83230v1_genomic.fna
1418	Pseudoalteromonas luteoviolacea	GCA_001750165.1_ASM175016v1_genomic.fna
1419	Cronobacter malonaticus	GCA_000504545.1_ASM50454v1_genomic.fna
1420	Lactobacillus mucosae	GCA_000248095.3_ASM24809v3_genomic.fna
1421	Xanthomonas fragariae	GCA_001705565.1_ASM170556v1_genomic.fna
1422	Thioalkalivibrio paradoxus	GCA_000227685.3_ASM22768v3_genomic.fna
1423	Advenella kashmirensis	GCA_000219915.3_ASM21991v3_genomic.fna
1424	Thermacetogenium phaeum	GCA_000305935.1_ASM30593v1_genomic.fna
1425	Paenibacillus terrae	GCA_000235585.1_ASM23558v1_genomic.fna

1426	Megasphaera elsdenii	GCA_001304715.1_ASM130471v1_genomic.fna
1427	Basilea psittacipulmonis	GCA_000743945.1_ASM74394v1_genomic.fna
1428	Gordonia terrae	GCA_001698225.1_ASM169822v1_genomic.fna
1429	Streptococcus macedonicus	GCA_000283635.1_ASM28363v1_genomic.fna
1430	Pseudomonas fragi	GCA_001543265.1_ASM154326v1_genomic.fna
1431	Nocardia brasiliensis	GCA_000250675.3_ASM25067v3_genomic.fna
1432	Wolbachia endosymbiont	GCA_000306885.1_ASM30688v1_genomic.fna
1433	Rubrivivax gelatinosus	GCA_000284255.1_ASM28425v1_genomic.fna
1434	Moraxella osloensis	GCA_001553955.1_ASM155395v1_genomic.fna
1435	Sphingomonas melonis	GCA_001761345.1_ASM176134v1_genomic.fna
1436	Corallococcus coralloides	GCA_000255295.1_ASM25529v1_genomic.fna
1437	Caldilinea aerophila	GCA_000281175.1_ASM28117v1_genomic.fna
1438	Amycolatopsis methanolica	GCA_000739085.1_ASM73908v1_genomic.fna
1439	Geobacillus thermoleovorans	GCA_001610955.1_ASM161095v1_genomic.fna
1440	Marinovum algicola	GCA_001046955.1_ASM104695v1_genomic.fna
1441	Thermus oshimai	GCA_000309885.1_ASM30988v1_genomic.fna
1442	Anaerotignum propionicum	GCA_001561955.1_ASM156195v1_genomic.fna
1443	Sulfurospirillum barnesii	GCA_000265295.1_ASM26529v1_genomic.fna
1444	Opitutaceae bacterium	GCA_000242935.3_ASM24293v3_genomic.fna
1445	Frateuria aurantia	GCA_000242255.3_ASM24225v3_genomic.fna
1446	Burkholderia seminalis	GCA_001718535.1_ASM171853v1_genomic.fna
1447	Solibacillus silvestris	GCA_001586195.1_ASM158619v1_genomic.fna
1448	Fimbriimonas ginsengisoli	GCA_000724625.1_ASM72462v1_genomic.fna
1449	Photorhabdus temperata	GCA_001010285.1_ASM101028v1_genomic.fna
1450	Pseudomonas chlororaphis	GCA_000698865.1_ASM69886v1_genomic.fna
1451	Campylobacter ureolyticus	GCA_001190755.1_ASM119075v1_genomic.fna
1452	Barnesiella viscericola	GCA_000512915.1_ASM51291v1_genomic.fna
1453	Bernardetia litoralis	GCA_000265505.1_ASM26550v1_genomic.fna
1454	Phycisphaera mikurensis	GCA_000284115.1_ASM28411v1_genomic.fna
1455	Bifidobacterium asteroides	GCA_000304215.1_ASM30421v1_genomic.fna
1456	Blastococcus saxobsidens	GCA_000284015.1_ASM28401v1_genomic.fna
1457	Pseudomonas mandelii	GCA_000257545.3_ASM25754v3_genomic.fna
1458	Pelosinus fermentans	GCA_000271665.2_ASM27166v2_genomic.fna
1459	Sulfurospirillum multivorans	GCA_000568815.1_ASM56881v1_genomic.fna

1460	Pararhodospirillum photometricum	GCA_000284415.2_ASM28441v2_genomic.fna
1461	Serratia plymuthica	GCA_000176835.2_ASM17683v2_genomic.fna
1462	Amycolatopsis orientalis	GCA_000400635.2_ASM40063v2_genomic.fna
1463	Methylacidiphilum fumariolicum	GCA_000953475.1_MfumSolVChr1_genomic.fna
1464	Alcaligenes faecalis	GCA_000967305.2_ASM96730v2_genomic.fna
1465	Phaeobacter inhibens	GCA_000154745.2_ASM15474v2_genomic.fna
1466	Cronobacter dublinensis	GCA_001277235.1_ASM127723v1_genomic.fna
1467	Cronobacter universalis	GCA_001277175.1_ASM127717v1_genomic.fna
1468	Mycobacterium haemophilum	GCA_000340435.3_ASM34043v3_genomic.fna
1469	Alteromonas stellipolaris	GCA_001562115.1_ASM156211v1_genomic.fna
1470	Staphylococcus equorum	GCA_001432245.1_ASM143224v1_genomic.fna
1471	Paraburkholderia caribensis	GCA_000522545.2_ASM52254v2_genomic.fna
1472	Cronobacter muytjensii	GCA_001277195.1_ASM127719v1_genomic.fna
1473	Cronobacter condimenti	GCA_001277255.1_ASM127725v1_genomic.fna
1474	Helicobacter cetorum	GCA_000259275.1_ASM25927v1_genomic.fna
1475	Desulfovibrio alaskensis	GCA_000012665.1_ASM1266v1_genomic.fna
1476	Pseudomonas knackmussii	GCA_000689415.1_PKB13_genomic.fna
1477	Desulfocapsa sulfexigens	GCA_000341395.1_ASM34139v1_genomic.fna
1478	Actinosynnema pretiosum	GCA_002354875.1_ASM235487v1_genomic.fna
1479	Actinoplanes missouriensis	GCA_000284295.1_ASM28429v1_genomic.fna
1480	Lactococcus piscium	GCA_000981525.1_L_piscium_genomic.fna
1481	Mycobacterium phlei	GCA_001583415.1_ASM158341v1_genomic.fna
1482	Acidipropionibacterium acidipropionici	GCA_001441165.1_ASM144116v1_genomic.fna
1483	Leptolyngbya boryana	GCA_002142495.1_ASM214249v1_genomic.fna
1484	Streptomyces globisporus	GCA_000261345.2_ASM26134v2_genomic.fna
1485	Cyanobium gracile	GCA_000316515.1_ASM31651v1_genomic.fna
1486	Dactylococcopsis salina	GCA_000317615.1_ASM31761v1_genomic.fna
1487	Oscillatoria acuminata	GCA_000317105.1_ASM31710v1_genomic.fna
1488	Crinalium epipsammum	GCA_000317495.1_ASM31749v1_genomic.fna
1489	Stanieria cyanosphaera	GCA_000317575.1_ASM31757v1_genomic.fna
1490	Bordetella holmesii	GCA_000612485.1_ASM61248v1_genomic.fna
1491	Kosakonia radicincitans	GCA_000280495.2_ASM28049v2_genomic.fna
1492	Thalassospira xiamenensis	GCA_000300235.2_ASM30023v2_genomic.fna
1493	Bacillus altitudinis	GCA_002443015.1_ASM244301v1_genomic.fna

1494	Bacillus safensis	GCA_001895885.1_ASM189588v1_genomic.fna
1495	Agrobacterium fabrum	GCA_000092025.1_ASM9202v1_genomic.fna
1496	Bdellovibrio exovorus	GCA_000348725.1_ASM34872v1_genomic.fna
1497	Granulicella tundricola	GCA_000178975.2_ASM17897v2_genomic.fna
1498	Planococcus antarcticus	GCA_001687565.2_ASM168756v2_genomic.fna
1499	Mycoplasma arginini	GCA_001547975.1_ASM154797v1_genomic.fna
1500	Vibrio breoganii	GCA_001677275.1_ASM167727v1_genomic.fna
1501	Rhodovulum sulfidophilum	GCA_001633165.1_ASM163316v1_genomic.fna
1502	Myxococcus macrosporus	GCA_002305895.1_ASM230589v1_genomic.fna
1503	Vibrio tasmaniensis	GCA_000091465.1_ASM9146v1_genomic.fna
1504	Melioribacter roseus	GCA_000279145.1_ASM27914v1_genomic.fna
1505	Granulosicoccus antarcticus	GCA_002215215.1_ASM221521v1_genomic.fna
1506	Sulfurospirillum halorespirans	GCA_001723605.1_ASM172360v1_genomic.fna
1507	Pseudomonas alcaliphila	GCA_001941865.1_ASM194186v1_genomic.fna
1508	Mycobacterium vaccae	GCA_001655245.1_ASM165524v1_genomic.fna
1509	Xanthomonas translucens	GCA_001021935.1_ASM102193v1_genomic.fna
1510	Snodgrassella alvi	GCA_000600005.1_ASM60000v1_genomic.fna
1511	Virgibacillus halodenitrificans	GCA_001878675.1_ASM187867v1_genomic.fna
1512	Bacillus endophyticus	GCA_000972245.3_ASM97224v3_genomic.fna
1513	Bacillus oceanisediminis	GCA_000294775.2_ASM29477v2_genomic.fna
1514	Corynebacterium terpenotabidum	GCA_000418365.1_ASM41836v1_genomic.fna
1515	Corynebacterium doosanense	GCA_000767055.1_ASM76705v1_genomic.fna
1516	Nocardiopsis alba	GCA_000294515.1_ASM29451v1_genomic.fna
1517	Rhizobium gallicum	GCA_000816845.1_ASM81684v1_genomic.fna
1518	Martelella mediterranea	GCA_002043005.1_ASM204300v1_genomic.fna
1519	Sulfuricella denitrificans	GCA_000297055.2_ASM29705v2_genomic.fna
1520	Aeromonas media	GCA_000287215.3_ASM28721v3_genomic.fna
1521	Thalassolituus oleivorans	GCA_000355675.1_ASM35567v1_genomic.fna
1522	Clostridium tyrobutyricum	GCA_001642655.1_ASM164265v1_genomic.fna
1523	Burkholderia pyrrocinia	GCA_001028665.1_ASM102866v1_genomic.fna
1524	Nocardia nova	GCA_000523235.1_ASM52323v1_genomic.fna
1525	Nocardia seriolae	GCA_001865855.1_ASM186585v1_genomic.fna
1526	Nocardia soli	GCA_001886715.1_ASM188671v1_genomic.fna
1527	Planococcus halocryophilus	GCA_001687585.2_ASM168758v2_genomic.fna

1528	Mycobacterium fortuitum	GCA_001307545.1_ASM130754v1_genomic.fna
1529	Pseudomonas alcaligenes	GCA_001597285.1_ASM159728v1_genomic.fna
1530	Pseudomonas azotoformans	GCA_001579805.1_ASM157980v1_genomic.fna
1531	Pseudomonas citronellolis	GCA_001654435.1_ASM165443v1_genomic.fna
1532	Pseudomonas cremoricolorata	GCA_000759535.1_ASM75953v1_genomic.fna
1533	Pseudomonas oryzihabitans	GCA_001518815.1_ASM151881v1_genomic.fna
1534	Pseudomonas parafulva	GCA_000800255.1_ASM80025v1_genomic.fna
1535	Pseudomonas plecoglossicida	GCA_000831585.1_ASM83158v1_genomic.fna
1536	Edwardsiella hoshinae	GCA_001761405.1_ASM176140v1_genomic.fna
1537	Elizabethkingia meningoseptica	GCA_002022105.1_ASM202210v1_genomic.fna
1538	Cedecea neteri	GCA_000757825.1_ASM75782v1_genomic.fna
1539	Paraburkholderia fungorum	GCA_000961515.1_ASM96151v1_genomic.fna
1540	Serratia rubidaea	GCA_001572725.1_ASM157272v1_genomic.fna
1541	Kluyvera intermedia	GCA_001022135.1_ASM102213v1_genomic.fna
1542	Sphingopyxis macrogoltabida	GCA_001314325.1_ASM131432v1_genomic.fna
1543	Sphingobium herbicidovorans	GCA_002080435.1_ASM208043v1_genomic.fna
1544	Sphingomonas koreensis	GCA_001922385.1_ASM192238v1_genomic.fna
1545	Sphingopyxis terrae	GCA_001610975.1_ASM161097v1_genomic.fna
1546	Sphingopyxis granuli	GCA_001559015.1_ASM155901v1_genomic.fna
1547	Vibrio natriegens	GCA_001456255.1_ASM145625v1_genomic.fna
1548	Vibrio gazogenes	GCA_002196515.1_ASM219651v1_genomic.fna
1549	Pseudothermotoga elfii	GCA_000504085.1_ASM50408v1_genomic.fna
1550	Pluralibacter gergoviae	GCA_000757785.1_ASM75778v1_genomic.fna
1551	Pseudothermotoga hypogea	GCA_000816145.1_ASM81614v1_genomic.fna
1552	Lelliottia amnigena	GCA_001652505.2_ASM165250v2_genomic.fna
1553	Delftia tsuruhatensis	GCA_001753225.1_ASM175322v1_genomic.fna
1554	Bacillus krulwichiae	GCA_002109385.1_ASM210938v1_genomic.fna
1555	Bacillus sonorensis	GCA_002202015.1_ASM220201v1_genomic.fna
1556	Acinetobacter schindleri	GCA_001971565.1_ASM197156v1_genomic.fna
1557	Legionella fallonii	GCA_000953135.1_LFA_genomic.fna
1558	Corynebacterium marinum	GCA_000835165.1_ASM83516v1_genomic.fna
1559	Corynebacterium maris	GCA_000442645.1_ASM44264v1_genomic.fna
1560	Corynebacterium humireducens	GCA_000819445.1_ASM81944v1_genomic.fna
1561	Corynebacterium vitaeruminis	GCA_000550805.1_ASM55080v1_genomic.fna

1562	Dickeya solani	GCA_001644705.1_ASM164470v1_genomic.fna
1563	Thioalkalivibrio versutus	GCA_001020955.1_ASM102095v1_genomic.fna
1564	Paraburkholderia phenoliruptrix	GCA_000300095.1_ASM30009v1_genomic.fna
1565	Paraglaciecola psychrophila	GCA_000347635.1_ASM34763v1_genomic.fna
1566	Kocuria palustris	GCA_001275345.1_ASM127534v1_genomic.fna
1567	Pseudomonas protegens	GCA_000397205.1_ASM39720v1_genomic.fna
1568	Leptospira alstonii	GCA_001729245.1_ASM172924v1_genomic.fna
1569	Octadecabacter arcticus	GCA_000155735.2_ASM15573v2_genomic.fna
1570	Staphylococcus pettenkoferi	GCA_002208805.1_ASM220880v1_genomic.fna
1571	Moorea producens	GCA_001767235.1_ASM176723v1_genomic.fna
1572	Pseudomonas resinovorans	GCA_000412695.1_ASM41269v1_genomic.fna
1573	Actinoplanes friuliensis	GCA_000494755.1_ASM49475v1_genomic.fna
1574	Streptomyces collinus	GCA_000444875.1_ASM44487v1_genomic.fna
1575	Thioalkalivibrio nitratireducens	GCA_000321415.2_ASM32141v2_genomic.fna
1576	Achromobacter denitrificans	GCA_001514355.1_ASM151435v1_genomic.fna
1577	Clostridium stercorarium	GCA_000331995.1_ASM33199v1_genomic.fna
1578	Citrobacter amalonaticus	GCA_000981805.1_ASM98180v1_genomic.fna
1579	Bibersteinia trehalosi	GCA_000347595.1_ASM34759v1_genomic.fna
1580	Herbaspirillum hiltneri	GCA_001267925.1_ASM126792v1_genomic.fna
1581	Mycobacterium neoaurum	GCA_000317305.3_ASM31730v3_genomic.fna
1582	Oscillatoria nigro-viridis	GCA_000317475.1_ASM31747v1_genomic.fna
1583	Nitrosospira briensis	GCA_000619905.2_ASM61990v2_genomic.fna
1584	Cyanobacterium aponinum	GCA_000317675.1_ASM31767v1_genomic.fna
1585	Streptococcus oralis	GCA_002356415.1_ASM235641v1_genomic.fna
1586	Lactococcus raffinolactis	GCA_002310475.1_ASM231047v1_genomic.fna
1587	Acinetobacter soli	GCA_001953195.1_ASM195319v1_genomic.fna
1588	Mycoplasma cynos	GCA_000328725.1_ASM32872v1_genomic.fna
1589	Rickettsia monacensis	GCA_000499665.2_RMONA_1_genomic.fna
1590	Tepidanaerobacter acetatoxydans	GCA_000328765.2_ASM32876v2_genomic.fna
1591	Liberibacter crescens	GCA_000325745.1_ASM32574v1_genomic.fna
1592	Saccharothrix espanaensis	GCA_000328705.1_ASM32870v1_genomic.fna
1593	Serratia liquefaciens	GCA_000422085.1_ASM42208v1_genomic.fna
1594	Raoultella ornithinolytica	GCA_000367425.1_ASM36742v1_genomic.fna
1595	Corynebacterium flavescens	GCA_001941465.1_ASM194146v1_genomic.fna

1596	Stenotrophomonas rhizophila	GCA_001704155.1_ASM170415v1_genomic.fna
1597	Corynebacterium stationis	GCA_001941345.1_ASM194134v1_genomic.fna
1598	Lactobacillus crustorum	GCA_001951175.1_ASM195117v1_genomic.fna
1599	Weissella ceti	GCA_000750535.1_ASM75053v1_genomic.fna
1600	Spiroplasma taiwanense	GCA_000439435.1_ASM43943v1_genomic.fna
1601	Spiroplasma diminutum	GCA_000439455.1_ASM43945v1_genomic.fna
1602	Spiroplasma chrysopicola	GCA_000400935.1_ASM40093v1_genomic.fna
1603	Spiroplasma syrphidicola	GCA_000400955.1_ASM40095v1_genomic.fna
1604	Spiroplasma culicicola	GCA_000565175.1_ASM56517v1_genomic.fna
1605	Spiroplasma sabaudiense	GCA_000565215.1_ASM56521v1_genomic.fna
1606	Spiroplasma apis	GCA_000500935.1_ASM50093v1_genomic.fna
1607	Ruegeria mobilis	GCA_000376545.2_ASM37654v2_genomic.fna
1608	Myxococcus stipitatus	GCA_000331735.1_ASM33173v1_genomic.fna
1609	Corynebacterium callunae	GCA_000344785.1_ASM34478v1_genomic.fna
1610	Bifidobacterium pseudolongum	GCA_000800475.2_ASM80047v2_genomic.fna
1611	Pannonibacter phragmitetus	GCA_001484065.1_ASM148406v1_genomic.fna
1612	Neorickettsia helminthoeca	GCA_000632985.1_ASM63298v1_genomic.fna
1613	Lysobacter antibioticus	GCA_001442535.1_ASM144253v1_genomic.fna
1614	Streptomyces albulus	GCA_000695235.1_ASM69523v1_genomic.fna
1615	Mannheimia varigena	GCA_000521655.1_ASM52165v1_genomic.fna
1616	Staphylococcus pasteuri	GCA_000494875.1_ASM49487v1_genomic.fna
1617	Trueperella pyogenes	GCA_000612055.1_ASM61205v1_genomic.fna
1618	Streptomyces violaceoruber	GCA_002082175.1_ASM208217v1_genomic.fna
1619	Streptomyces laurentii	GCA_002355495.1_ASM235549v1_genomic.fna
1620	Sporosarcina ureae	GCA_002082015.1_ASM208201v1_genomic.fna
1621	Roseovarius mucosus	GCA_002080415.1_ASM208041v1_genomic.fna
1622	Pseudomonas poae	GCA_000336465.1_ASM33646v1_genomic.fna
1623	Aerococcus urinaeequi	GCA_001518795.1_ASM151879v1_genomic.fna
1624	Brevundimonas naejangsanensis	GCA_000635915.2_ASM63591v2_genomic.fna
1625	Rathayibacter toxicus	GCA_001465855.1_ASM146585v1_genomic.fna
1626	Treponema pedis	GCA_000447675.1_ASM44767v1_genomic.fna
1627	Borrelia miyamotoi	GCA_000445425.4_ASM44542v4_genomic.fna
1628	Photobacterium gaetbulicola	GCA_000940995.1_ASM94099v1_genomic.fna
1629	Melittangium boletus	GCA_002305855.1_ASM230585v1_genomic.fna

1630	Mycobacterium yongonense	GCA_000418535.2_ASM41853v2_genomic.fna
1631	Dokdonella koreensis	GCA_001632775.1_ASM163277v1_genomic.fna
1632	Isoptericola dokdonensis	GCA_001636295.1_ASM163629v1_genomic.fna
1633	Bradyrhizobium oligotrophicum	GCA_000344805.1_ASM34480v1_genomic.fna
1634	Nocardioides dokdonensis	GCA_001653335.1_ASM165333v1_genomic.fna
1635	Streptomyces fulvissimus	GCA_000385945.1_ASM38594v1_genomic.fna
1636	Pseudoalteromonas agarivorans	GCA_002310855.1_ASM231085v1_genomic.fna
1637	Ilumatobacter coccineus	GCA_000348785.1_ASM34878v1_genomic.fna
1638	Pseudomonas veronii	GCA_002028325.1_ASM202832v1_genomic.fna
1639	Bacillus flexus	GCA_002024265.1_ASM202426v1_genomic.fna
1640	Cupriavidus gilardii	GCA_001281465.1_ASM128146v1_genomic.fna
1641	Plesiomonas shigelloides	GCA_900087055.1_31289_E01_genomic.fna
1642	Variovorax boronicumulans	GCA_002291405.1_ASM229140v1_genomic.fna
1643	Rathayibacter tritici	GCA_001641005.1_ASM164100v1_genomic.fna
1644	Azotobacter chroococcum	GCA_000817975.1_ASM81797v1_genomic.fna
1645	Cycloclasticus zancles	GCA_000442595.1_ASM44259v1_genomic.fna
1646	Bordetella hinzii	GCA_001078275.1_ASM107827v1_genomic.fna
1647	Serratia fonticola	GCA_001006005.1_ASM100600v1_genomic.fna
1648	Paenibacillus durus	GCA_000756615.1_ASM75661v1_genomic.fna
1649	Vibrio owensii	GCA_002021755.1_ASM202175v1_genomic.fna
1650	Paenibacillus graminis	GCA_000758705.1_ASM75870v1_genomic.fna
1651	Enterobacter lignolyticus	GCA_000164865.1_ASM16486v1_genomic.fna
1652	Burkholderia contaminans	GCA_001029145.1_ASM102914v1_genomic.fna
1653	Spiribacter salinus	GCA_000319575.2_ASM31957v2_genomic.fna
1654	Thermodesulfobacterium geofontis	GCA_000215975.1_ASM21597v1_genomic.fna
1655	Clostridium autoethanogenum	GCA_000484505.1_ASM48450v1_genomic.fna
1656	Clostridium saccharobutylicum	GCA_000473995.1_ASM47399v1_genomic.fna
1657	Roseomonas gilardii	GCA_001941945.1_ASM194194v1_genomic.fna
1658	Sphingobium baderi	GCA_001456115.1_ASM145611v1_genomic.fna
1659	Corynebacterium argentoratense	GCA_000590555.1_ASM59055v1_genomic.fna
1660	Cellulophaga baltica	GCA_000468615.2_ASM46861v2_genomic.fna
1661	Microbulbifer agarilyticus	GCA_001999945.1_ASM199994v1_genomic.fna
1662	Bradyrhizobium diazoefficiens	GCA_000011365.1_ASM1136v1_genomic.fna
1663	Clostridium chauvoei	GCA_900168365.1_JF4335_genomic.fna

1664	Campylobacter subantarcticus	GCA_000816265.1_ASM81626v1_genomic.fna
1665	Adlercreutzia equolifaciens	GCA_000478885.1_ASM47888v1_genomic.fna
1666	Desulfococcus multivorans	GCA_001854245.1_ASM185424v1_genomic.fna
1667	Pragia fontium	GCA_001026985.1_ASM102698v1_genomic.fna
1668	Pseudomonas mosselii	GCA_000498975.2_ASM49897v2_genomic.fna
1669	Yersinia similis	GCA_000582515.1_ASM58251v1_genomic.fna
1670	Streptomyces lydicus	GCA_001729485.1_ASM172948v1_genomic.fna
1671	Bacillus infantis	GCA_000473245.1_ASM47324v1_genomic.fna
1672	Acholeplasma brassicae	GCA_000967915.1_ASM96791v1_genomic.fna
1673	Acholeplasma palmae	GCA_000968055.1_ASM96805v1_genomic.fna
1674	Mycoplasma parvum	GCA_000477415.1_ASM47741v1_genomic.fna
1675	Bacillus toyonensis	GCA_000496285.1_ASM49628v1_genomic.fna
1676	Capnocytophaga haemolytica	GCA_001553545.1_ASM155354v1_genomic.fna
1677	Streptomyces niveus	GCA_002009175.1_ASM200917v1_genomic.fna
1678	Hyphomicrobium nitrativorans	GCA_000503895.1_ASM50389v1_genomic.fna
1679	Pandoraea pnomenusa	GCA_000767615.3_ASM76761v3_genomic.fna
1680	Ehrlichia muris	GCA_000508225.1_ASM50822v1_genomic.fna
1681	Mycoplasma ovis	GCA_000508245.1_ASM50824v1_genomic.fna
1682	Legionella oakridgensis	GCA_000512355.1_ASM51235v1_genomic.fna
1683	Sphingomonas sanxanigenens	GCA_000512205.2_ASM51220v2_genomic.fna
1684	Bifidobacterium kashiwanohense	GCA_000800455.1_ASM80045v1_genomic.fna
1685	Kosakonia sacchari	GCA_000300455.4_ASM30045v4_genomic.fna
1686	Aeromonas dhakensis	GCA_002285935.1_ASM228593v1_genomic.fna
1687	Chlamydia gallinacea	GCA_000471025.2_ASM47102v2_genomic.fna
1688	Borrelia anserina	GCA_001936255.1_ASM193625v1_genomic.fna
1689	Borrelia parkeri	GCA_000512145.2_ASM51214v2_genomic.fna
1690	Komagataeibacter medellinensis	GCA_000182745.1_ASM18274v1_genomic.fna
1691	Cardinium endosymbiont	GCA_000304455.1_CCh_cEper1_genomic.fna
1692	Modestobacter marinus	GCA_000306785.1_ASM30678v1_genomic.fna
1693	Enterobacteriaceae bacterium	GCA_000330845.1_ASM33084v1_genomic.fna
1694	beta proteobacterium	GCA_000342265.1_ASM34226v1_genomic.fna
1695	Burkholderia lata	GCA_000012945.1_ASM1294v1_genomic.fna
1696	Magnetococcus marinus	GCA_000014865.1_ASM1486v1_genomic.fna
1697	Mycobacterium gilvum	GCA_000016365.1_ASM1636v1_genomic.fna

1698	Geobacter daltonii	GCA_000022265.1_ASM2226v1_genomic.fna
1699	Bordetella trematum	GCA_900078695.1_H044680328_genomic.fna
1700	Paraburkholderia sprentiae	GCA_001865575.1_ASM186557v1_genomic.fna
1701	Gloeobacter kilaueensis	GCA_000484535.1_ASM48453v1_genomic.fna
1702	Alcanivorax pacificus	GCA_000299335.2_ASM29933v2_genomic.fna
1703	Bacteroidales bacterium	GCA_000473305.1_ASM47330v1_genomic.fna
1704	Methylophaga frappieri	GCA_000260965.1_ASM26096v1_genomic.fna
1705	Methylophaga nitratireducenticrescens	GCA_000260985.3_ASM26098v3_genomic.fna
1706	Rhodanobacter denitrificans	GCA_000230695.3_ASM23069v3_genomic.fna
1707	Bacillus velezensis	GCA_000015785.1_ASM1578v1_genomic.fna
1708	Pseudomonas cichorii	GCA_000517305.1_ASM51730v1_genomic.fna
1709	Pseudomonas corrugata	GCA_001708425.1_ASM170842v1_genomic.fna
1710	Gemmatirosa kalamazoonesis	GCA_000522985.1_ASM52298v1_genomic.fna
1711	Kutzneria albida	GCA_000525635.1_ASM52563v1_genomic.fna
1712	Corynebacterium falsenii	GCA_000525655.1_ASM52565v1_genomic.fna
1713	Hymenobacter swuensis	GCA_000576555.1_ASM57655v1_genomic.fna
1714	Clostridium cellulosi	GCA_000953215.1_DG5_genomic.fna
1715	Ensifer adhaerens	GCA_000697965.2_ASM69796v2_genomic.fna
1716	Chlamydia avium	GCA_000583875.1_ASM58387v1_genomic.fna
1717	Roseibacterium elongatum	GCA_000590925.1_ASM59092v1_genomic.fna
1718	Peptoclostridium acidaminophilum	GCA_000597865.1_ASM59786v1_genomic.fna
1719	Flammeovirgaceae bacterium	GCA_000597885.1_ASM59788v1_genomic.fna
1720	Gilliamella apicola	GCA_000599985.1_ASM59998v1_genomic.fna
1721	Pseudomonas protegens	GCA_000012265.1_ASM1226v1_genomic.fna
1722	Lysobacter capsici	GCA_001442785.1_ASM144278v1_genomic.fna
1723	Paenibacillus sabinae	GCA_000612505.1_ASM61250v1_genomic.fna
1724	Lactobacillus gallinarum	GCA_001314245.2_ASM131424v2_genomic.fna
1725	Halotalea alkalilenta	GCA_001648175.1_ASM164817v1_genomic.fna
1726	Paracoccus yeei	GCA_002073635.1_ASM207363v1_genomic.fna
1727	Castellaniella defragrans	GCA_000612685.1_scf7180000000004_genomic.fna
1728	Prevotella enoeca	GCA_001444445.1_ASM144444v1_genomic.fna
1729	Prevotella fusca	GCA_001262015.1_ASM126201v1_genomic.fna
1730	Geobacillus thermocatenulatus	GCA_002243665.1_ASM224366v1_genomic.fna
1731	Draconibacterium orientale	GCA_000626635.1_ASM62663v1_genomic.fna

1732	Corynebacterium glyciniphilum	GCA_000626675.1_ASM62667v1_genomic.fna
1733	Novosphingobium resinovorum	GCA_001742225.1_ASM174222v1_genomic.fna
1734	Chlamydia suis	GCA_900169085.1_SWA-2_genomic.fna
1735	Clostridium scatologenes	GCA_000968375.1_ASM96837v1_genomic.fna
1736	Dyella jiangningensis	GCA_000632805.1_ASM63280v1_genomic.fna
1737	Acetobacter persici	GCA_002006565.1_ASM200656v1_genomic.fna
1738	Streptomyces pratensis	GCA_000176115.2_ASM17611v2_genomic.fna
1739	Lactobacillus kunkeei	GCA_001314945.1_ASM131494v1_genomic.fna
1740	Edwardsiella piscicida	GCA_000348565.1_ASM34856v1_genomic.fna
1741	Mycoplasma californicum	GCA_000695835.1_ASM69583v1_genomic.fna
1742	Halomonas campaniensis	GCA_000696485.1_ASM69648v1_genomic.fna
1743	Kozakia baliensis	GCA_001787335.1_ASM178733v1_genomic.fna
1744	Rhodoluna lacicola	GCA_000699505.1_ASM69950v1_genomic.fna
1745	Sinorhizobium americanum	GCA_000705595.2_ASM70559v2_genomic.fna
1746	Bifidobacterium indicum	GCA_000706765.1_ASM70676v1_genomic.fna
1747	Bacillus lehensis	GCA_000706725.1_ASM70672v1_genomic.fna
1748	Streptomyces alboflavus	GCA_002189675.1_ASM218967v1_genomic.fna
1749	Kitasatospora aureofaciens	GCA_002082605.1_ASM208260v1_genomic.fna
1750	Janthinobacterium agaricidamnosum	GCA_000723165.1_JAG1_genomic.fna
1751	Mucinivorans hirudinis	GCA_000723505.1_Mucivorans_M3_Nelson-Bomar-Fixed_genomic.fna
1752	Terribacillus aidingensis	GCA_000725365.1_ASM72536v1_genomic.fna
1753	Dermacoccus nishinomiyaensis	GCA_000725405.1_ASM72540v1_genomic.fna
1754	Alteromonas australica	GCA_000730385.1_ASM73038v1_genomic.fna
1755	Arenibacter algicola	GCA_002234495.1_ASM223449v1_genomic.fna
1756	Amycolatopsis japonica	GCA_000732925.1_ASM73292v1_genomic.fna
1757	Corynebacterium atypicum	GCA_000732945.1_ASM73294v1_genomic.fna
1758	Sandaracinus amylolyticus	GCA_000737325.2_ASM73732v2_genomic.fna
1759	Staphylococcus agnetis	GCA_001442815.1_ASM144281v1_genomic.fna
1760	Bifidobacterium coryneforme	GCA_000737865.1_ASM73786v1_genomic.fna
1761	Corynebacterium imitans	GCA_000739455.1_ASM73945v1_genomic.fna
1762	Bifidobacterium actinocoloniiforme	GCA_001263395.1_ASM126339v1_genomic.fna
1763	Bifidobacterium scardovii	GCA_001042635.1_ASM104263v1_genomic.fna
1764	Geobacillus subterraneus	GCA_001618685.1_ASM161868v1_genomic.fna
1765	Pseudomonas alkylphenolica	GCA_000746525.1_ASM74652v1_genomic.fna

1766	Rickettsiales bacterium	GCA_000746585.2_ASM74658v2_genomic.fna
1767	Corynebacterium ureicelerivorans	GCA_000747315.1_ASM74731v1_genomic.fna
1768	Carnobacterium inhibens	GCA_000493735.1_ASM49373v1_genomic.fna
1769	Ruminococcus bicirculans	GCA_000723465.1_Rb803_genomic.fna
1770	Streptomyces leeuwenhoekii	GCA_001013905.1_sleC34_genomic.fna
1771	Cobetia marina	GCA_001720485.1_ASM172048v1_genomic.fna
1772	Klebsiella michiganensis	GCA_000240325.1_ASM24032v1_genomic.fna
1773	Treponema putidum	GCA_000755145.1_ASM75514v1_genomic.fna
1774	Nonlabens sediminis	GCA_002117085.1_ASM211708v1_genomic.fna
1775	Paenibacillus stellifer	GCA_000758685.1_ASM75868v1_genomic.fna
1776	Paenibacillus odorifer	GCA_000758725.1_ASM75872v1_genomic.fna
1777	Lactobacillus paraplantarum	GCA_001443645.1_ASM144364v1_genomic.fna
1778	Lysinibacillus varians	GCA_000600105.1_ASM60010v1_genomic.fna
1779	Paenibacillus borealis	GCA_000758665.1_ASM75866v1_genomic.fna
1780	Pseudomonas rhizosphaerae	GCA_000761155.1_ASM76115v1_genomic.fna
1781	Streptomyces glaucescens	GCA_000761215.1_ASM76121v1_genomic.fna
1782	Actinopolyspora erythraea	GCA_002263515.1_ASM226351v1_genomic.fna
1783	Thermoanaerobacter kivui	GCA_000763575.1_ASM76357v1_genomic.fna
1784	Sphingomonas taxi	GCA_000764535.1_ASM76453v1_genomic.fna
1785	Intestinimonas butyriciproducens	GCA_001454945.1_ASM145494v1_genomic.fna
1786	Helicobacter typhlonius	GCA_001460635.1_htyphlonius_mit97-6810_genomic.fna
1787	Pantoea rwandensis	GCA_000759475.1_ASM75947v1_genomic.fna
1788	Corynebacterium camporealensis	GCA_000980815.1_ASM98081v1_genomic.fna
1789	Fervidobacterium islandicum	GCA_000767275.3_ASM76727v3_genomic.fna
1790	Anoxybacillus gonensis	GCA_001187595.1_ASM118759v1_genomic.fna
1791	Elizabethkingia miricola	GCA_000955665.1_ASM95566v1_genomic.fna
1792	Bacillus xiamenensis	GCA_001857925.1_ASM185792v1_genomic.fna
1793	Lactobacillus curieae	GCA_000785105.2_ASM78510v2_genomic.fna
1794	Pimelobacter simplex	GCA_000785495.1_ASM78549v1_genomic.fna
1795	Citrobacter braakii	GCA_002073755.1_ASM207375v1_genomic.fna
1796	Clostridium baratii	GCA_000789395.1_ASM78939v1_genomic.fna
1797	Collimonas arenae	GCA_000786695.1_ASM78669v1_genomic.fna
1798	Halomonas hydrothermalis	GCA_002442575.1_ASM244257v1_genomic.fna
1799	Deinococcus swuensis	GCA_000800395.1_ASM80039v1_genomic.fna

1800	Fusobacterium hwasookii	GCA_001455085.1_ASM145508v1_genomic.fna
1801	Actinobacillus equuli	GCA_000801145.1_ASM80114v1_genomic.fna
1802	Pseudomonas frederiksbergensis	GCA_001874645.1_ASM187464v1_genomic.fna
1803	Streptomyces pluripotens	GCA_000802245.2_ASM80224v2_genomic.fna
1804	Borreliella chilensis	GCA_000808095.1_ASM80809v1_genomic.fna
1805	Frischella perrara	GCA_000807275.1_ASM80727v1_genomic.fna
1806	Sulfurospirillum cavolei	GCA_001548055.1_ASM154805v1_genomic.fna
1807	Coriobacteriaceae bacterium	GCA_000814825.1_ASM81482v1_genomic.fna
1808	Pandoraea sputorum	GCA_000814845.2_ASM81484v2_genomic.fna
1809	Coxiella endosymbiont	GCA_000815025.1_ASM81502v1_genomic.fna
1810	Pandoraea pulmonicola	GCA_000815105.2_ASM81510v2_genomic.fna
1811	Allofrancisella guangzhouensis	GCA_000815225.1_ASM81522v1_genomic.fna
1812	Methylomonas denitrificans	GCA_000785705.2_ASM78570v2_genomic.fna
1813	Campylobacter peloridis	GCA_000816785.1_ASM81678v1_genomic.fna
1814	Geobacter pickeringii	GCA_000817955.1_ASM81795v1_genomic.fna
1815	Pseudomonas balearica	GCA_000818015.1_ASM81801v1_genomic.fna
1816	Clostridium argentinense	GCA_002074155.1_ASM207415v1_genomic.fna
1817	Celeribacter indicus	GCA_000819565.1_ASM81956v1_genomic.fna
1818	Enterobacter ludwigii	GCA_001750725.1_ASM175072v1_genomic.fna
1819	Aeromonas schubertii	GCA_001447335.1_ASM144733v1_genomic.fna
1820	Pandoraea apista	GCA_000826965.3_ASM82696v3_genomic.fna
1821	Geoalkalibacter subterraneus	GCA_000827125.1_ASM82712v1_genomic.fna
1822	Methyloceanibacter caenitepidi	GCA_000828475.1_ASM82847v1_genomic.fna
1823	Thiolapillus brandeum	GCA_000828615.1_ASM82861v1_genomic.fna
1824	Sulfuritalea hydrogenivorans	GCA_000828635.1_ASM82863v1_genomic.fna
1825	Thermotoga caldifontis	GCA_000828655.1_ASM82865v1_genomic.fna
1826	Thermotoga profunda	GCA_000828675.1_ASM82867v1_genomic.fna
1827	Streptomyces vietnamensis	GCA_000830005.1_ASM83000v1_genomic.fna
1828	Bacillus bombysepticus	GCA_000831065.1_ASM83106v1_genomic.fna
1829	Marinobacter similis	GCA_000830985.1_ASM83098v1_genomic.fna
1830	Marinobacter salarius	GCA_000831005.1_ASM83100v1_genomic.fna
1831	Thioploca ingrica	GCA_000828835.1_ASM82883v1_genomic.fna
1832	Mycoplasma canadense	GCA_000828855.1_ASM82885v1_genomic.fna
1833	Comamonadaceae bacterium	GCA_000828895.1_ASM82889v1_genomic.fna

1834	Comamonadaceae bacterium	GCA_000828915.1_ASM82891v1_genomic.fna
1835	Burkholderiales bacterium	GCA_000828975.1_ASM82897v1_genomic.fna
1836	cyanobacterium endosymbiont	GCA_000829235.1_ASM82923v1_genomic.fna
1837	Wolbachia endosymbiont	GCA_000829315.1_ASM82931v1_genomic.fna
1838	Lactobacillus hokkaidonensis	GCA_000829395.1_ASM82939v1_genomic.fna
1839	Lactobacillus heilongjiangensis	GCA_000831645.3_ASM83164v3_genomic.fna
1840	Corynebacterium singulare	GCA_000833575.1_ASM83357v1_genomic.fna
1841	Salinispira pacifica	GCA_000507245.1_ASM50724v1_genomic.fna
1842	Myroides profundi	GCA_000833025.1_ASM83302v1_genomic.fna
1843	Mycobacterium immunogenum	GCA_001605725.1_ASM160572v1_genomic.fna
1844	Campylobacter iguaniorum	GCA_000736415.1_ASM73641v1_genomic.fna
1845	Streptomyces cyaneogriseus	GCA_000931445.1_ASM93144v1_genomic.fna
1846	Rugosibacter aromaticivorans	GCA_000934545.1_ASM93454v1_genomic.fna
1847	Pandoraea vervacti	GCA_000934605.2_ASM93460v2_genomic.fna
1848	Sulfitobacter pseudonitzschiae	GCA_002222635.1_ASM222263v1_genomic.fna
1849	Siansivirga zeaxanthinifaciens	GCA_000941055.1_ASM94105v1_genomic.fna
1850	Mycoplasma dispar	GCA_000941075.1_ASM94107v1_genomic.fna
1851	Clostridium aceticum	GCA_001042715.1_ASM104271v1_genomic.fna
1852	Gynuella sunshinyii	GCA_000940805.1_ASM94080v1_genomic.fna
1853	Acholeplasma oculi	GCA_000953195.1_Acholeplasma_oculi_strain_19L_genomic.fna
1854	Fermentimonas caenicola	GCA_000953535.1_E5B_genomic.fna
1855	Tatlockia micdadei	GCA_000953635.1_LMI_genomic.fna
1856	Aliivibrio wodanis	GCA_000953695.1_AWOD1_genomic.fna
1857	Defluviitoga tunisiensis	GCA_000953715.1_DTL3_genomic.fna
1858	Moritella viscosa	GCA_000953735.1_MVIS1_genomic.fna
1859	Ralstonia mannitolilytica	GCA_000954135.2_ASM95413v2_genomic.fna
1860	Azospirillum thiophilum	GCA_001305595.1_ASM130559v1_genomic.fna
1861	Kosakonia oryzae	GCA_001658025.1_ASM165802v1_genomic.fna
1862	Martelella endophytica	GCA_000960975.1_ASM96097v1_genomic.fna
1863	Paenibacillus beijingensis	GCA_000961095.1_ASM96109v1_genomic.fna
1864	Muricauda lutaonensis	GCA_000963865.1_ASM96386v1_genomic.fna
1865	Mageeibacillus indolicus	GCA_000025225.2_ASM2522v1_genomic.fna
1866	Oleispira antarctica	GCA_000967895.1_ASM96789v1_genomic.fna
1867	Xenorhabdus poinarii	GCA_000968175.1_ASM96817v1_genomic.fna

1868	Xenorhabdus doucetiae	GCA_000968195.1_ASM96819v1_genomic.fna
1869	Chania multitudinisentens	GCA_000520015.2_ASM52001v2_genomic.fna
1870	Chromobacterium vaccinii	GCA_001855275.1_ASM185527v1_genomic.fna
1871	Verrucomicrobia bacterium	GCA_000972765.1_ASM97276v1_genomic.fna
1872	Pandoraea oxalativorans	GCA_000972785.3_ASM97278v3_genomic.fna
1873	Sodalis praecaptivus	GCA_000517425.1_ASM51742v1_genomic.fna
1874	Blochmannia endosymbiont	GCA_000973505.1_ASM97350v1_genomic.fna
1875	Blochmannia endosymbiont	GCA_000973545.1_ASM97354v1_genomic.fna
1876	Pontibacter korlensis	GCA_000973725.1_ASM97372v1_genomic.fna
1877	Spirosoma radiotolerans	GCA_000974425.1_ASM97442v1_genomic.fna
1878	Corynebacterium kutscheri	GCA_000980835.1_ASM98083v1_genomic.fna
1879	Salimicrobium jeotgali	GCA_001685435.3_ASM168543v3_genomic.fna
1880	Kangiella geojedonensis	GCA_000981765.1_ASM98176v1_genomic.fna
1881	Sulfurovum lithotrophicum	GCA_000987835.1_ASM98783v1_genomic.fna
1882	Brevibacterium flavum	GCA_000987865.1_ASM98786v1_genomic.fna
1883	Halomonas huangheensis	GCA_001431725.1_ASM143172v1_genomic.fna
1884	Clostridium bornimense	GCA_000577895.1_M2_40_genomic.fna
1885	Salinicoccus halodurans	GCA_001005905.1_ASM100590v1_genomic.fna
1886	Staphylococcus argenteus	GCA_000236925.1_ASM23692v1_genomic.fna
1887	Nitrosospira lacus	GCA_000355765.4_ASM35576v4_genomic.fna
1888	Gemmatimonas phototrophica	GCA_000695095.2_ASM69509v2_genomic.fna
1889	Nitrosomonas communis	GCA_001007935.1_ASM100793v1_genomic.fna
1890	Deinococcus soli	GCA_001007995.1_ASM100799v1_genomic.fna
1891	Altererythrobacter atlanticus	GCA_001008165.2_ASM100816v2_genomic.fna
1892	Staphylococcus succinus	GCA_001902315.1_ASM190231v1_genomic.fna
1893	Erythrobacter gangjinensis	GCA_001886695.1_ASM188669v1_genomic.fna
1894	Vibrio antiquarius	GCA_000024825.1_ASM2482v1_genomic.fna
1895	Brevibacillus formosus	GCA_002215075.1_ASM221507v1_genomic.fna
1896	Mycoplasma gallinaceum	GCA_000965765.1_ASM96576v1_genomic.fna
1897	Polyangium brachysporum	GCA_001017435.1_ASM101743v1_genomic.fna
1898	Staphylococcus condimenti	GCA_001618885.1_ASM161888v1_genomic.fna
1899	Pandoraea thiooxydans	GCA_001931675.1_ASM193167v1_genomic.fna
1900	Klebsiella quasipneumoniae	GCA_001596075.2_ASM159607v2_genomic.fna
1901	Aeromonas aquatica	GCA_001895965.1_ASM189596v1_genomic.fna

1902	Sphingopyxis fribergensis	GCA_000803645.1_ASM80364v1_genomic.fna
1903	Corynebacterium mustelae	GCA_001020985.1_ASM102098v1_genomic.fna
1904	Corynebacterium epidermidicanis	GCA_001021025.1_ASM102102v1_genomic.fna
1905	Corynebacterium testudinoris	GCA_001021045.1_ASM102104v1_genomic.fna
1906	Corynebacterium uterequi	GCA_001021065.1_ASM102106v1_genomic.fna
1907	Chryseobacterium gallinarum	GCA_001021975.1_ASM102197v1_genomic.fna
1908	Actinobacteria bacterium	GCA_001023575.1_ASM102357v1_genomic.fna
1909	Kosmotoga pacifica	GCA_001027025.1_ASM102702v1_genomic.fna
1910	Archangium gephyra	GCA_001027285.1_ASM102728v1_genomic.fna
1911	Endomicrobium proavitum	GCA_001027545.1_ASM102754v1_genomic.fna
1912	Croceicoccus naphthovorans	GCA_001028705.1_ASM102870v1_genomic.fna
1913	Pandoraea faecigallinarum	GCA_001029105.3_ASM102910v3_genomic.fna
1914	Spiroplasma atrichopogonis	GCA_001029245.1_ASM102924v1_genomic.fna
1915	Berkelbacteria bacterium	GCA_001029735.1_ASM102973v1_genomic.fna
1916	Altererythrobacter marensis	GCA_001028625.1_ASM102862v1_genomic.fna
1917	Mycobacterium sinense	GCA_000214155.1_ASM21415v1_genomic.fna
1918	Lactobacillus acetotolerans	GCA_001042405.1_ASM104240v1_genomic.fna
1919	Marinobacter psychrophilus	GCA_001043175.1_ASM104317v1_genomic.fna
1920	Methylobacterium aquaticum	GCA_001548015.1_ASM154801v1_genomic.fna
1921	Yersinia aleksiciae	GCA_001047675.1_ASM104767v1_genomic.fna
1922	Cyclobacterium amurskyense	GCA_001050135.1_ASM105013v1_genomic.fna
1923	Lactobacillus koreensis	GCA_001050435.1_ASM105043v1_genomic.fna
1924	Lactobacillus ginsenosidimutans	GCA_001050475.1_ASM105047v1_genomic.fna
1925	Thauera humireducens	GCA_001051995.2_ASM105199v2_genomic.fna
1926	Coxiella-like endosymbiont	GCA_001077715.1_ASM107771v1_genomic.fna
1927	Pseudomonas trivialis	GCA_001186335.1_ASM118633v1_genomic.fna
1928	Mycobacterium goodii	GCA_001187505.1_ASM118750v1_genomic.fna
1929	Wenzhouxiangella marina	GCA_001187785.1_ASM118778v1_genomic.fna
1930	Octadecabacter temperatus	GCA_001187845.1_ASM118784v1_genomic.fna
1931	Staphylococcus schleiferi	GCA_001188855.1_ASM118885v1_genomic.fna
1932	Chondromyces crocatus	GCA_001189295.1_ASM118929v1_genomic.fna
1933	Luteipulveratus mongoliensis	GCA_001190945.1_ASM119094v1_genomic.fna
1934	Jeotgalibaca dankookensis	GCA_002005405.1_ASM200540v1_genomic.fna
1935	Lactobacillus agilis	GCA_002240375.1_ASM224037v1_genomic.fna

1936	Spiroplasma turonicum	GCA_001262715.1_ASM126271v1_genomic.fna
1937	Vulgatibacter incomptus	GCA_001263175.1_ASM126317v1_genomic.fna
1938	Labilithrix luteola	GCA_001263205.1_ASM126320v1_genomic.fna
1939	Microcystis panniformis	GCA_001264245.1_ASM126424v1_genomic.fna
1940	Actinomyces meyeri	GCA_001262055.1_ASM126205v1_genomic.fna
1941	Spiroplasma litorale	GCA_001267155.1_ASM126715v1_genomic.fna
1942	Oblitimonas alkaliphila	GCA_001267175.1_ASM126717v1_genomic.fna
1943	Nitrospira moscoviensis	GCA_001273775.1_ASM127377v1_genomic.fna
1944	Corynebacterium lactis	GCA_001274895.1_ASM127489v1_genomic.fna
1945	Corynebacterium deserti	GCA_001277995.1_ASM127799v1_genomic.fna
1946	Microbacterium chocolatum	GCA_001652465.1_ASM165246v1_genomic.fna
1947	Bartonella ancashensis	GCA_001281405.1_ASM128140v1_genomic.fna
1948	Altererythrobacter epoxidivorans	GCA_001281485.1_ASM128148v1_genomic.fna
1949	Spiroplasma cantharicola	GCA_001281045.1_ASM128104v1_genomic.fna
1950	Lawsonella clevelandensis	GCA_001293125.1_ASM129312v1_genomic.fna
1951	Comamonas kerstersii	GCA_002056725.1_ASM205672v1_genomic.fna
1952	Arthrobacter alpinus	GCA_001294625.1_ASM129462v1_genomic.fna
1953	Pseudoalteromonas tetraodonis	GCA_002310835.1_ASM231083v1_genomic.fna
1954	Psychrobacter urativorans	GCA_001298525.1_ASM129852v1_genomic.fna
1955	Kibdelosporangium phytohabitans	GCA_001302585.1_ASM130258v1_genomic.fna
1956	Beggiatoa leptomitiformis	GCA_001305575.2_ASM130557v2_genomic.fna
1957	Sphingomonas hengshuiensis	GCA_000935025.1_ASM93502v1_genomic.fna
1958	Rufibacter tibetensis	GCA_001310085.1_ASM131008v1_genomic.fna
1959	Mycobacterium chimaera	GCA_001750065.2_ASM175006v2_genomic.fna
1960	Celeribacter marinus	GCA_001308265.1_ASM130826v1_genomic.fna
1961	Lactobacillus lindneri	GCA_001702135.1_ASM170213v1_genomic.fna
1962	Lactobacillus paracollinoides	GCA_001702175.1_ASM170217v1_genomic.fna
1963	Lactobacillus amylophilus	GCA_001936335.1_ASM193633v1_genomic.fna
1964	Methylobacterium phyllosphaerae	GCA_001936175.1_ASM193617v1_genomic.fna
1965	Kosakonia cowanii	GCA_001975225.1_ASM197522v1_genomic.fna
1966	Stenotrophomonas acidaminiphila	GCA_001314305.1_ASM131430v1_genomic.fna
1967	Mycobacterium pallens	GCA_002335685.1_ASM233568v1_genomic.fna
1968	Blastochloris viridis	GCA_001402875.1_ASM140287v1_genomic.fna
1969	Streptomyces pactum	GCA_001767375.1_ASM176737v1_genomic.fna

1970	Streptomyces puniciscabiei	GCA_001735805.1_ASM173580v1_genomic.fna
1971	Streptomyces silaceus	GCA_001975025.1_ASM197502v1_genomic.fna
1972	Agarivorans gilvus	GCA_001420915.1_ASM142091v1_genomic.fna
1973	Bordetella pseudohinzii	GCA_001698185.1_ASM169818v1_genomic.fna
1974	Stenotrophomonas nitritireducens	GCA_001700965.1_ASM170096v1_genomic.fna
1975	Lactobacillus parabuchneri	GCA_001922025.1_ASM192202v1_genomic.fna
1976	Lactobacillus zymae	GCA_900183405.1_ASM90018340v1_genomic.fna
1977	Bacillus paralicheniformis	GCA_000408885.1_ASM40888v1_genomic.fna
1978	Bacillus muralis	GCA_001645685.2_ASM164568v2_genomic.fna
1979	Sneathia amnii	GCA_000973085.1_ASM97308v1_genomic.fna
1980	Lentibacillus amyloliquefaciens	GCA_001307805.2_ASM130780v2_genomic.fna
1981	Bacillus glycinifermentans	GCA_900093775.1_EVONIK_BGLY_genomic.fna
1982	Lysobacter gummosus	GCA_001442805.1_ASM144280v1_genomic.fna
1983	Salinivirga cyanobacteriivorans	GCA_001443605.1_ASM144360v1_genomic.fna
1984	Pseudoalteromonas phenolica	GCA_001444405.1_ASM144440v1_genomic.fna
1985	Pseudohongiella spirulinae	GCA_001444425.1_ASM144442v1_genomic.fna
1986	Halocynthiibacter arcticus	GCA_000812665.2_ASM81266v2_genomic.fna
1987	Acinetobacter equi	GCA_001307195.1_ASM130719v1_genomic.fna
1988	Nitrosomonas ureae	GCA_001455205.1_ASM145520v1_genomic.fna
1989	Pseudoalteromonas issachenkonii	GCA_001455325.1_ASM145532v1_genomic.fna
1990	Mycobacterium saopaulense	GCA_001456355.1_ASM145635v1_genomic.fna
1991	Devriesea agamarum	GCA_900070355.1_S2_rcS3_S1_rcS3_genomic.fna
1992	Pseudoalteromonas translucida	GCA_001465295.1_ASM146529v1_genomic.fna
1993	Pandoraea norimbergensis	GCA_001465545.3_ASM146554v3_genomic.fna
1994	Planococcus rifietoensis	GCA_001465795.2_ASM146579v2_genomic.fna
1995	Planococcus kocurii	GCA_001465835.2_ASM146583v2_genomic.fna
1996	Lacimicrobium alkaliphilum	GCA_001466725.1_ASM146672v1_genomic.fna
1997	Paenibacillus naphthalenovorans	GCA_001465255.1_ASM146525v1_genomic.fna
1998	Alteromonas mediterranea	GCA_000020585.3_ASM2058v3_genomic.fna
1999	Enterobacter xiangfangensis	GCA_001729785.1_ASM172978v1_genomic.fna
2000	Kocuria flava	GCA_001482365.1_ASM148236v1_genomic.fna
2001	Roseateles depolymerans	GCA_001483865.1_ASM148386v1_genomic.fna
2002	Pseudarthrobacter sulfonivorans	GCA_001484605.1_ASM148460v1_genomic.fna
2003	Tenacibaculum dicentrarchi	GCA_001483385.1_ASM148338v1_genomic.fna

2004	Acetobacter senegalensis	GCA_001499615.1_Acetobacter_senegalensis_108B_genomic.fna
2005	Thermus parvatiensis	GCA_001535545.1_ASM153554v1_genomic.fna
2006	Deinococcus actinosclerus	GCA_001507665.1_ASM150766v1_genomic.fna
2007	Aeromicrobium erythreum	GCA_001509405.1_ASM150940v1_genomic.fna
2008	Streptomyces reticuli	GCA_001511815.1_TUE45_genomic.fna
2009	Paenibacillus bovis	GCA_001421015.2_ASM142101v2_genomic.fna
2010	Rhodothermaceae bacterium	GCA_001518995.2_ASM151899v2_genomic.fna
2011	Burkholderia pseudomultivorans	GCA_001718415.1_ASM171841v1_genomic.fna
2012	Erwinia gerundensis	GCA_001517405.1_EM595_genomic.fna
2013	Burkholderia territorii	GCA_001718335.1_ASM171833v1_genomic.fna
2014	Burkholderia diffusa	GCA_001718315.1_ASM171831v1_genomic.fna
2015	Burkholderia stagnalis	GCA_001718955.1_ASM171895v1_genomic.fna
2016	Burkholderia latens	GCA_001718795.1_ASM171879v1_genomic.fna
2017	Arsenophonus symbiont	GCA_001534665.1_ASM153466v1_genomic.fna
2018	Streptomyces griseochromogenes	GCA_001542625.2_ASM154262v2_genomic.fna
2019	Microterricola viridarii	GCA_001542775.1_ASM154277v1_genomic.fna
2020	Aerococcus sanguinicola	GCA_001543145.1_ASM154314v1_genomic.fna
2021	Aerococcus urinaehominis	GCA_001543245.1_ASM154324v1_genomic.fna
2022	Caldimicrobium thiodismutans	GCA_001548275.1_ASM154827v1_genomic.fna
2023	Actinomyces radicidentis	GCA_001553565.1_ASM155356v1_genomic.fna
2024	Desulfovibrio fairfieldensis	GCA_001553605.1_ASM155360v1_genomic.fna
2025	Limnochorda pilosa	GCA_001544015.1_ASM154401v1_genomic.fna
2026	Halomonas chromatireducens	GCA_001545155.1_ASM154515v1_genomic.fna
2027	Thermosulfidibacter takaii	GCA_001547735.1_ASM154773v1_genomic.fna
2028	Vibrio tritonius	GCA_001547935.1_ASM154793v1_genomic.fna
2029	Asaia bogorensis	GCA_001547995.1_ASM154799v1_genomic.fna
2030	Faecalibaculum rodentium	GCA_001564455.1_ASM156445v1_genomic.fna
2031	Myxococcus hansupus	GCA_000280925.3_ASM28092v3_genomic.fna
2032	Alteromonas addita	GCA_001562195.1_ASM156219v1_genomic.fna
2033	Sinomonas atrocyanea	GCA_001577305.1_ASM157730v1_genomic.fna
2034	Steroidobacter denitrificans	GCA_001579945.1_ASM157994v1_genomic.fna
2035	Corynebacterium simulans	GCA_001586215.1_ASM158621v1_genomic.fna
2036	Brucella vulpis	GCA_900000005.1_BVF60_genomic.fna
2037	Enterobacter cloacae	GCA_001729805.1_ASM172980v1_genomic.fna

2038	Enterobacter cloacae	GCA_000750225.1_ASM75022v1_genomic.fna
2039	Gluconobacter albidus	GCA_002005485.1_ASM200548v1_genomic.fna
2040	Collimonas pratensis	GCA_001584185.1_ASM158418v1_genomic.fna
2041	Obesumbacterium proteus	GCA_001586165.1_ASM158616v1_genomic.fna
2042	Zhongshania aliphaticivorans	GCA_001586255.1_ASM158625v1_genomic.fna
2043	Pedobacter cryoconitis	GCA_001590605.1_ASM159060v1_genomic.fna
2044	Sporosarcina psychrophila	GCA_001590685.1_ASM159068v1_genomic.fna
2045	Serratia ficaria	GCA_900187015.1_50465_F01_genomic.fna
2046	Brevundimonas vesicularis	GCA_002208825.1_ASM220882v1_genomic.fna
2047	Planococcus maritimus	GCA_001999905.1_ASM199990v1_genomic.fna
2048	Edwardsiella anguillarum	GCA_000264765.2_ASM26476v2_genomic.fna
2049	Aminobacter aminovorans	GCA_001605015.1_ASM160501v1_genomic.fna
2050	Helicobacter himalayensis	GCA_001602095.1_ASM160209v1_genomic.fna
2051	Pseudomonas koreensis	GCA_001605965.1_ASM160596v1_genomic.fna
2052	Psychrobacter alimentarius	GCA_001606025.1_ASM160602v1_genomic.fna
2053	Bacteroidetes bacterium	GCA_001602415.1_ASM160241v1_genomic.fna
2054	Hyphomonadaceae bacterium	GCA_001602435.1_ASM160243v1_genomic.fna
2055	Betaproteobacteria bacterium	GCA_001602455.1_ASM160245v1_genomic.fna
2056	Pontibacter akesuensis	GCA_001611675.1_ASM161167v1_genomic.fna
2057	Haematospirillum jordaniae	GCA_001611975.1_ASM161197v1_genomic.fna
2058	Jeotgalibacillus malaysiensis	GCA_000818095.1_ASM81809v1_genomic.fna
2059	Rummeliibacillus stabekisii	GCA_001617605.1_ASM161760v1_genomic.fna
2060	Sedimenticola thiotaurini	GCA_001007875.1_ASM100787v1_genomic.fna
2061	Microbulbifer thermotolerans	GCA_001617625.1_ASM161762v1_genomic.fna
2062	Fictibacillus phosphorivorans	GCA_001629705.2_ASM162970v2_genomic.fna
2063	Polynucleobacter duraquae	GCA_000973625.1_ASM97362v1_genomic.fna
2064	Defluviimonas alba	GCA_001620265.1_ASM162026v1_genomic.fna
2065	Chelatococcus daeguensis	GCA_001887265.1_ASM188726v1_genomic.fna
2066	Geobacter anodireducens	GCA_001628815.1_ASM162881v1_genomic.fna
2067	Anoxybacillus amylolyticus	GCA_001634285.1_ASM163428v1_genomic.fna
2068	Erythrobacter atlanticus	GCA_001077815.2_ASM107781v2_genomic.fna
2069	Algibacter alginicilyticus	GCA_001310225.1_ASM131022v1_genomic.fna
2070	Moraxella ovis	GCA_001636015.1_ASM163601v1_genomic.fna
2071	Azospirillum humicireducens	GCA_001639105.1_ASM163910v1_genomic.fna

2072	Fictibacillus arsenicus	GCA_001642935.2_ASM164293v2_genomic.fna
2073	Deinococcus puniceus	GCA_001644565.1_ASM164456v1_genomic.fna
2074	Paenibacillus swuensis	GCA_001644605.1_ASM164460v1_genomic.fna
2075	Dyella thiooxydans	GCA_001641285.1_ASM164128v1_genomic.fna
2076	Pseudomonas antarctica	GCA_001647715.1_ASM164771v1_genomic.fna
2077	Denitrobacterium detoxificans	GCA_001643775.1_ASM164377v1_genomic.fna
2078	Polynucleobacter asymbioticus	GCA_000016345.1_ASM1634v1_genomic.fna
2079	Dietzia timorensis	GCA_001659785.1_ASM165978v1_genomic.fna
2080	Ochrobactrum pseudogrignonense	GCA_001652485.1_ASM165248v1_genomic.fna
2081	Streptococcus pantholopis	GCA_001642085.1_ASM164208v1_genomic.fna
2082	Ralstonia insidiosa	GCA_001653935.1_ASM165393v1_genomic.fna
2083	Streptomyces parvulus	GCA_001660045.1_ASM166004v1_genomic.fna
2084	Croceicoccus marinus	GCA_001661675.2_ASM166167v2_genomic.fna
2085	Altererythrobacter ishigakiensis	GCA_001663155.1_ASM166315v1_genomic.fna
2086	Porphyrobacter neustonensis	GCA_001663175.1_ASM166317v1_genomic.fna
2087	Lactobacillus backii	GCA_001663675.1_ASM166367v1_genomic.fna
2088	Rhizobiales bacterium	GCA_001664005.1_ASM166400v1_genomic.fna
2089	Acinetobacter lactucae	GCA_002076935.1_ASM207693v1_genomic.fna
2090	Bordetella flabilis	GCA_001676725.1_ASM167672v1_genomic.fna
2091	Woeseia oceani	GCA_001677435.1_ASM167743v1_genomic.fna
2092	Dermabacter vaginalis	GCA_001678905.1_ASM167890v1_genomic.fna
2093	Pseudomonas cerasi	GCA_900074915.1_PCPL58T_genomic.fna
2094	Alteromonas naphthalenivorans	GCA_000213655.1_ASM21365v1_genomic.fna
2095	Bordetella bronchialis	GCA_001676705.1_ASM167670v1_genomic.fna
2096	Caulobacteraceae bacterium	GCA_001679665.1_ASM167966v1_genomic.fna
2097	Cryobacterium arcticum	GCA_001679725.1_ASM167972v1_genomic.fna
2098	Polaribacter reichenbachii	GCA_001975665.1_ASM197566v1_genomic.fna
2099	Azoarcus olearius	GCA_001682385.1_ASM168238v1_genomic.fna
2100	Streptomyces lincolnensis	GCA_001685355.1_ASM168535v1_genomic.fna
2101	Burkholderia stabilis	GCA_001742165.1_ASM174216v1_genomic.fna
2102	Altererythrobacter namhicola	GCA_001687545.1_ASM168754v1_genomic.fna
2103	Planococcus plakortidis	GCA_001687605.2_ASM168760v2_genomic.fna
2104	Burkholderiales bacterium	GCA_001688905.2_ASM168890v2_genomic.fna
2105	Erysipelotrichaceae bacterium	GCA_001688965.2_ASM168896v2_genomic.fna

2106	Bradyrhizobium icense	GCA_001693385.1_ASM169338v1_genomic.fna
2107	Desulfuromonas soudanensis	GCA_001278055.1_ASM127805v1_genomic.fna
2108	Streptococcus halotolerans	GCA_001598035.1_ASM159803v1_genomic.fna
2109	Streptococcus marmotae	GCA_001623565.1_ASM162356v1_genomic.fna
2110	Wenyingzhuangia fucanilytica	GCA_001697185.1_ASM169718v1_genomic.fna
2111	Immundisolibacter cernigliae	GCA_001697225.1_ASM169722v1_genomic.fna
2112	Altererythrobacter dongtanensis	GCA_001698205.1_ASM169820v1_genomic.fna
2113	Akkermansia glycaniphila	GCA_900097105.1_WK001_genomic.fna
2114	Cloacibacillus porcorum	GCA_001701045.1_ASM170104v1_genomic.fna
2115	Streptomyces sampsonii	GCA_001704195.1_ASM170419v1_genomic.fna
2116	Streptomyces noursei	GCA_001704275.1_ASM170427v1_genomic.fna
2117	Paenibacillus kribbensis	GCA_002240415.1_ASM224041v1_genomic.fna
2118	Paenibacillus yonginensis	GCA_001685395.1_ASM168539v1_genomic.fna
2119	Kangiella sediminilitoris	GCA_001708405.1_ASM170840v1_genomic.fna
2120	Spiroplasma helicoides	GCA_001715535.1_ASM171553v1_genomic.fna
2121	Dialister pneumosintes	GCA_001717505.1_ASM171750v1_genomic.fna
2122	Anaerolineaceae bacterium	GCA_001717545.1_ASM171754v1_genomic.fna
2123	Sphingomonas panacis	GCA_001717955.1_ASM171795v1_genomic.fna
2124	Burkholderia metallica	GCA_001718555.1_ASM171855v1_genomic.fna
2125	Bacillus gibsonii	GCA_001719185.1_ASM171918v1_genomic.fna
2126	Xenorhabdus hominickii	GCA_001721185.1_ASM172118v1_genomic.fna
2127	Pedobacter steynii	GCA_001721645.1_ASM172164v1_genomic.fna
2128	Bacillus beveridgei	GCA_001721685.1_ASM172168v1_genomic.fna
2129	Frankia casuarinae	GCA_000013345.1_ASM1334v1_genomic.fna
2130	Clostridium taeniosporum	GCA_001735765.1_ASM173576v1_genomic.fna
2131	bacterium AB1	GCA_001729625.1_ASM172962v1_genomic.fna
2132	Lacunisphaera limnophila	GCA_001746835.1_ASM174683v1_genomic.fna
2133	Chlorobaculum limnaeum	GCA_001747405.1_ASM174740v1_genomic.fna
2134	Actinoalloteichus hymeniacidonis	GCA_001747425.1_ASM174742v1_genomic.fna
2135	Pectobacterium parmentieri	GCA_000024645.1_ASM2464v1_genomic.fna
2136	Geosporobacter ferrireducens	GCA_001750685.1_ASM175068v1_genomic.fna
2137	Streptomyces rubrolavendulae	GCA_001750785.1_ASM175078v1_genomic.fna
2138	Flavobacterium gilvum	GCA_001761465.1_ASM176146v1_genomic.fna
2139	Streptococcus himalayensis	GCA_001708305.1_ASM170830v1_genomic.fna

2140	Clostridium formicaceticum	GCA_001854185.1_ASM185418v1_genomic.fna
2141	Prauserella marina	GCA_002240355.1_ASM224035v1_genomic.fna
2142	Tessaracoccus flavus	GCA_001997295.1_ASM199729v1_genomic.fna
2143	Petrimonas mucosa	GCA_900095795.1_E5A_genomic.fna
2144	Niabella ginsenosidivorans	GCA_001654455.1_ASM165445v1_genomic.fna
2145	Frankia inefficax	GCA_000166135.1_ASM16613v1_genomic.fna
2146	Planococcus faecalis	GCA_002009235.1_ASM200923v1_genomic.fna
2147	Polaribacter vadi	GCA_001761365.1_ASM176136v1_genomic.fna
2148	Vagococcus teuberi	GCA_001870205.1_ASM187020v1_genomic.fna
2149	Lentzea guizhouensis	GCA_001701025.1_ASM170102v1_genomic.fna
2150	Clostridium estertheticum	GCA_001877035.1_ASM187703v1_genomic.fna
2151	Flavobacteriaceae bacterium	GCA_001880285.1_ASM188028v1_genomic.fna
2152	Thermus brockianus	GCA_001880325.1_ASM188032v1_genomic.fna
2153	Corynebacterium crudilactis	GCA_001643015.1_ASM164301v1_genomic.fna
2154	Neomicrococcus aestuarii	GCA_001887245.1_ASM188724v1_genomic.fna
2155	Microbacterium paludicola	GCA_001887285.1_ASM188728v1_genomic.fna
2156	Agromyces aureus	GCA_001660485.1_ASM166048v1_genomic.fna
2157	Pelobacter acetylenicus	GCA_001888165.1_ASM188816v1_genomic.fna
2158	Bacteroides caecimuris	GCA_001688725.2_ASM168872v2_genomic.fna
2159	Mycoplasma pullorum	GCA_001900245.1_ASM190024v1_genomic.fna
2160	Paenibacillus xylanexedens	GCA_001908275.1_ASM190827v1_genomic.fna
2161	Kiritimatiella glycovorans	GCA_001017655.1_ASM101765v1_genomic.fna
2162	Minicystis rosea	GCA_001931535.1_ASM193153v1_genomic.fna
2163	Weissella jogaejeotgali	GCA_001932615.1_ASM193261v1_genomic.fna
2164	Borrelia mayonii	GCA_001945665.1_ASM194566v1_genomic.fna
2165	Streptococcus acidominimus	GCA_900187245.1_51470_D02_genomic.fna
2166	Thauera chlorobenzoica	GCA_001922305.1_ASM192230v1_genomic.fna
2167	Ndongobacter massiliensis	GCA_900120375.1_PRJEB18029_genomic.fna
2168	Corynebacterium aquilae	GCA_001941445.1_ASM194144v1_genomic.fna
2169	Corynebacterium frankenforstense	GCA_001941485.1_ASM194148v1_genomic.fna
2170	Corynebacterium sphenisci	GCA_001941505.1_ASM194150v1_genomic.fna
2171	Corynebacterium phocae	GCA_001941565.1_ASM194156v1_genomic.fna
2172	Massilia putida	GCA_001941825.1_ASM194182v1_genomic.fna
2173	Gramella flava	GCA_001951155.1_ASM195115v1_genomic.fna

2174	Paludisphaera borealis	GCA_001956985.1_ASM195698v1_genomic.fna
2175	Bartonella apis	GCA_002007565.1_ASM200756v1_genomic.fna
2176	Ezakiella massiliensis	GCA_900120165.1_PRJEB15652_genomic.fna
2177	Lachnoclostridium phocaeense	GCA_900120345.1_PRJEB18024_genomic.fna
2178	Halioglobus pacificus	GCA_001953075.1_ASM195307v1_genomic.fna
2179	Betaproteobacteria bacterium	GCA_001953215.1_ASM195321v1_genomic.fna
2180	Negativicoccus massiliensis	GCA_900155405.1_PRJEB18760_genomic.fna
2181	Rhodoferax saidenbachensis	GCA_001955715.1_ASM195571v1_genomic.fna
2182	Tateyamaria omphalii	GCA_001969365.1_ASM196936v1_genomic.fna
2183	Thiobacimonas profunda	GCA_001969385.1_ASM196938v1_genomic.fna
2184	Spiribacter curvatus	GCA_000485905.1_ASM48590v1_genomic.fna
2185	Hymenobacter sedentarius	GCA_001507645.1_ASM150764v1_genomic.fna
2186	Dehalogenimonas formicexedens	GCA_001953175.1_ASM195317v1_genomic.fna
2187	Bacillus weihaiensis	GCA_001889165.1_ASM188916v1_genomic.fna
2188	Achromobacter insolitus	GCA_001971645.1_ASM197164v1_genomic.fna
2189	Lutibacter profundi	GCA_001543325.1_ASM154332v1_genomic.fna
2190	Fuerstia marisgermanicae	GCA_001983935.1_ASM198393v1_genomic.fna
2191	Streptomyces autolyticus	GCA_001983975.1_ASM198397v1_genomic.fna
2192	Halioglobus japonicus	GCA_001983995.1_ASM198399v1_genomic.fna
2193	Lacinutrix venerupis	GCA_001971745.1_ASM197174v1_genomic.fna
2194	Proteiniphilum saccharofermentans	GCA_900095135.1_M36_genomic.fna
2195	Microbacterium aurum	GCA_001974985.1_ASM197498v1_genomic.fna
2196	Pelagibaca abyssi	GCA_001975705.1_ASM197570v1_genomic.fna
2197	Pseudodesulfovibrio indicus	GCA_001563225.1_ASM156322v1_genomic.fna
2198	Flavobacterium crassostreae	GCA_001831475.1_ASM183147v1_genomic.fna
2199	Acinetobacter larvae	GCA_001704115.1_ASM170411v1_genomic.fna
2200	Spirosoma montaniterrae	GCA_001988955.1_ASM198895v1_genomic.fna
2201	Auricoccus indicus	GCA_001989575.1_ASM198957v1_genomic.fna
2202	Phycisphaerae bacterium	GCA_001997385.1_ASM199738v1_genomic.fna
2203	Erysipelothrix larvae	GCA_001545095.1_ASM154509v1_genomic.fna
2204	Marinobacter salinus	GCA_001854125.1_ASM185412v1_genomic.fna
2205	Leuconostoc garlicum	GCA_001998805.1_ASM199880v1_genomic.fna
2206	Tessaracoccus flavescens	GCA_001998865.1_ASM199886v1_genomic.fna
2207	Vagococcus penaei	GCA_001998885.1_ASM199888v1_genomic.fna

2208	Pseudoalteromonas aliena	GCA_001999225.1_ASM199922v1_genomic.fna
2209	Phycisphaerae bacterium	GCA_001999965.1_ASM199996v1_genomic.fna
2210	Desulfotomaculum ferrireducens	GCA_002005145.1_ASM200514v1_genomic.fna
2211	Novibacillus thermophilus	GCA_002005165.1_ASM200516v1_genomic.fna
2212	Shewanella psychrophila	GCA_002005305.1_ASM200530v1_genomic.fna
2213	Paenalcaligenes hominis	GCA_002005365.1_ASM200536v1_genomic.fna
2214	Phycisphaerae bacterium	GCA_002007645.1_ASM200764v1_genomic.fna
2215	Mycobacterium litorale	GCA_002007745.1_ASM200774v1_genomic.fna
2216	Komagataeibacter nataicola	GCA_002009295.1_ASM200929v1_genomic.fna
2217	Neoasaia chiangmaiensis	GCA_002005465.1_ASM200546v1_genomic.fna
2218	Anaerococcus mediterraneensis	GCA_900128415.1_PRJEB15655_genomic.fna
2219	Campylobacter pinnipediorum	GCA_002021925.1_ASM202192v1_genomic.fna
2220	Elizabethkingia endophytica	GCA_002022045.1_ASM202204v1_genomic.fna
2221	Ochrobactrum pituitosum	GCA_002025625.1_ASM202562v1_genomic.fna
2222	Curtobacterium pusillum	GCA_002025645.1_ASM202564v1_genomic.fna
2223	Alkalitalea saponilacus	GCA_002201795.1_ASM220179v1_genomic.fna
2224	Phoenicibacter massiliensis	GCA_900169485.1_PRJEB19959_genomic.fna
2225	Spirosoma aerolatum	GCA_002056795.1_ASM205679v1_genomic.fna
2226	Nitrospira japonica	GCA_900169565.1_NSJP_Ch1_genomic.fna
2227	Halomicronema hongdechloris	GCA_002075285.2_ASM207528v2_genomic.fna
2228	Shewanella japonica	GCA_002075795.1_ASM207579v1_genomic.fna
2229	Mycobacterium paraintracellulare	GCA_000276825.1_ASM27682v1_genomic.fna
2230	Parasaccharibacter apium	GCA_002079945.1_ASM207994v1_genomic.fna
2231	Streptomyces gilvosporeus	GCA_002082195.1_ASM208219v1_genomic.fna
2232	Kitasatospora albolonga	GCA_002082585.1_ASM208258v1_genomic.fna
2233	Christensenella massiliensis	GCA_900155415.1_PRJEB13909b_genomic.fna
2234	Spirosoma rigui	GCA_002067135.1_ASM206713v1_genomic.fna
2235	Blattabacterium cuenoti	GCA_000348805.1_ASM34880v1_genomic.fna
2236	Chromatiaceae bacterium	GCA_002009425.1_ASM200942v1_genomic.fna
2237	Mycobacterium marseillense	GCA_002285715.1_ASM228571v1_genomic.fna
2238	Pseudomonas versuta	GCA_001294575.1_ASM129457v1_genomic.fna
2239	Cnuibacter physcomitrellae	GCA_002096055.1_ASM209605v1_genomic.fna
2240	Halobacillus mangrovi	GCA_002097535.1_ASM209753v1_genomic.fna
2241	Pseudomonas mesoacidophila	GCA_002097715.1_ASM209771v1_genomic.fna

2242	Staphylococcus lutrae	GCA_002101335.1_ASM210133v1_genomic.fna
2243	Pantoea alhagi	GCA_002101395.1_ASM210139v1_genomic.fna
2244	Alcanivorax xenomutans	GCA_002072815.1_ASM207281v1_genomic.fna
2245	Paracoccus contaminans	GCA_002105555.1_ASM210555v1_genomic.fna
2246	Rhizobacter gummiphilus	GCA_002116905.1_ASM211690v1_genomic.fna
2247	Phycisphaerae bacterium	GCA_002117005.1_ASM211700v1_genomic.fna
2248	Oceanicoccus sagamiensis	GCA_002117105.1_ASM211710v1_genomic.fna
2249	Methylocystis bryophila	GCA_002117405.1_ASM211740v1_genomic.fna
2250	Ketogulonicigenium robustum	GCA_002117445.1_ASM211744v1_genomic.fna
2251	Methylobacterium zatmanii	GCA_002117815.1_ASM211781v1_genomic.fna
2252	Ruminococcaceae bacterium	GCA_002119605.1_ASM211960v1_genomic.fna
2253	Pseudorhodoplanes sinuspersici	GCA_002119765.1_ASM211976v1_genomic.fna
2254	Macrococcus canis	GCA_002119805.1_ASM211980v1_genomic.fna
2255	Sphingobacteriaceae bacterium	GCA_002151545.1_ASM215154v1_genomic.fna
2256	Kushneria marisflavi	GCA_002157205.1_ASM215720v1_genomic.fna
2257	Agarilytica rhodophyticola	GCA_002157225.1_ASM215722v1_genomic.fna
2258	Halomonas aestuarii	GCA_001886615.1_ASM188661v1_genomic.fna
2259	Gordonibacter massiliensis	GCA_900170005.1_PRJEB19975_genomic.fna
2260	Glutamicibacter halophytocola	GCA_001302565.1_ASM130256v1_genomic.fna
2261	Comamonas serinivorans	GCA_002158865.1_ASM215886v1_genomic.fna
2262	Oceanisphaera profunda	GCA_002157895.1_ASM215789v1_genomic.fna
2263	Gordonibacter urolithinfaciens	GCA_900199375.1_G142160_polished_assembly_ID17483_genomic.fna
2264	Spiroplasma clarkii	GCA_002163565.1_ASM216356v1_genomic.fna
2265	Nostocales cyanobacterium	GCA_002163975.1_ASM216397v1_genomic.fna
2266	Luteitalea pratensis	GCA_001618865.1_ASM161886v1_genomic.fna
2267	Herbinix luporum	GCA_900070325.1_SD1D_genomic.fna
2268	Oleiphilus messinensis	GCA_002162375.1_ASM216237v1_genomic.fna
2269	Thioclava nitratireducens	GCA_001940525.2_ASM194052v2_genomic.fna
2270	Brevefilum fermentans	GCA_900184705.1_CAMBI-1_genomic.fna
2271	Paenibacillus donghaensis	GCA_002192415.1_ASM219241v1_genomic.fna
2272	Streptomyces albireticuli	GCA_002192455.1_ASM219245v1_genomic.fna
2273	Methylovulum psychrotolerans	GCA_002209385.1_ASM220938v1_genomic.fna
2274	Idiomarina piscisalsi	GCA_002211765.1_ASM221176v1_genomic.fna
2275	Francisella halioticida	GCA_002211785.1_ASM221178v1_genomic.fna

2276	Diaphorobacter polyhydroxybutyrativorans	GCA_002214645.1_ASM221464v1_genomic.fna
2277	Shewanella marisflavi	GCA_002215585.1_ASM221558v1_genomic.fna
2278	Thalassotalea crassostreae	GCA_001831495.1_ASM183149v1_genomic.fna
2279	Phaeobacter porticola	GCA_001888185.1_ASM188818v1_genomic.fna
2280	Paraphotobacterium marinum	GCA_002216855.1_ASM221685v1_genomic.fna
2281	Shewanella bicestrii	GCA_002216875.1_ASM221687v1_genomic.fna
2282	Sphingorhabdus flavimaris	GCA_002218195.1_ASM221819v1_genomic.fna
2283	Pseudoalteromonas nigrifaciens	GCA_002221505.1_ASM222150v1_genomic.fna
2284	Pseudoalteromonas espejiana	GCA_002221525.1_ASM222152v1_genomic.fna
2285	Vitreoscilla filiformis	GCA_002222655.1_ASM222265v1_genomic.fna
2286	Virgibacillus necropolis	GCA_002224365.1_ASM222436v1_genomic.fna
2287	Dehalobacterium formicoaceticum	GCA_002224645.1_ASM222464v1_genomic.fna
2288	Legionella clemsonensis	GCA_002240035.1_ASM224003v1_genomic.fna
2289	Flavobacterium communis	GCA_001857965.1_ASM185796v1_genomic.fna
2290	Herminiimonas arsenitoxidans	GCA_900130075.1_Herminiimonas_arsenitoxidans_genomic.fna
2291	Pseudoalteromonas piratica	GCA_000788395.1_ASM78839v1_genomic.fna
2292	Geobacillus lituanicus	GCA_002243605.1_ASM224360v1_genomic.fna
2293	Tumebacillus algifaecis	GCA_002243515.1_ASM224351v1_genomic.fna
2294	Actinoalloteichus hoggarensis	GCA_002234535.1_ASM223453v1_genomic.fna
2295	Zobellella denitrificans	GCA_002407485.1_ASM240748v1_genomic.fna
2296	Bacterioplanes sanyensis	GCA_002237535.1_ASM223753v1_genomic.fna
2297	Spiroplasma corruscae	GCA_002237575.1_ASM223757v1_genomic.fna
2298	Erythrobacter flavus	GCA_002237615.1_ASM223761v1_genomic.fna
2299	Marinobacterium aestuarii	GCA_001651805.1_ASM165180v1_genomic.fna
2300	Staphylococcus muscae	GCA_900187005.1_50569_B01_genomic.fna
2301	Staphylococcus stepanovicii	GCA_900187075.1_50432_F01_genomic.fna
2302	Chryseobacterium taklimakanense	GCA_900187185.1_50618_E01_genomic.fna
2303	Veillonella rodentium	GCA_900187285.1_51342_C02_genomic.fna
2304	Staphylococcus piscifermentans	GCA_900186985.1_50377_C01_genomic.fna
2305	Tenacibaculum jejuense	GCA_900198195.1_Tjejuense_V1_genomic.fna
2306	Bacillus kochii	GCA_002276165.1_ASM227616v1_genomic.fna
2307	Actinobacteria bacterium	GCA_002284855.1_ASM228485v1_genomic.fna
2308	Actinobacteria bacterium	GCA_002284875.1_ASM228487v1_genomic.fna
2309	Actinobacteria bacterium	GCA_002284895.1_ASM228489v1_genomic.fna

2310	Actinobacteria bacterium	GCA_002284915.1_ASM228491v1_genomic.fna
2311	Actinomyces gaoshouyii	GCA_002076915.1_ASM207691v1_genomic.fna
2312	Thermogutta terrifontis	GCA_002277955.1_ASM227795v1_genomic.fna
2313	Mycobacterium dioxanotrophicus	GCA_002157835.1_ASM215783v1_genomic.fna
2314	Corynebacterium glaucum	GCA_002287505.1_ASM228750v1_genomic.fna
2315	Kushneria konosiri	GCA_002155145.1_ASM215514v1_genomic.fna
2316	Gibbsiella quercinecans	GCA_002291425.1_ASM229142v1_genomic.fna
2317	Mycobacterium lepraemurium	GCA_002291465.1_ASM229146v1_genomic.fna
2318	Capnocytophaga leadbetteri	GCA_002302615.1_ASM230261v1_genomic.fna
2319	Capnocytophaga stomatis	GCA_002302635.1_ASM230263v1_genomic.fna
2320	Verrucomicrobia bacterium	GCA_002310495.1_ASM231049v1_genomic.fna
2321	Microbulbifer aggregans	GCA_001750105.1_ASM175010v1_genomic.fna
2322	Janthinobacterium svalbardensis	GCA_002327145.1_ASM232714v1_genomic.fna
2323	Trichormus variabilis	GCA_000204075.1_ASM20407v1_genomic.fna
2324	Hydrogenophaga crassostreae	GCA_001761385.1_ASM176138v1_genomic.fna
2325	Streptococcus troglodytae	GCA_002355215.1_ASM235521v1_genomic.fna
2326	Rhizorhabdus dicambivorans	GCA_002355275.1_ASM235527v1_genomic.fna
2327	Variibacter gotjawalensis	GCA_002355335.1_ASM235533v1_genomic.fna
2328	Aneurinibacillus soli	GCA_002355375.1_ASM235537v1_genomic.fna
2329	Microcella alkaliphila	GCA_002355395.1_Microcella_alkaliphila_JAM_AC0309_assembly_01_genomic.fna
2330	Sulfurifustis variabilis	GCA_002355415.1_ASM235541v1_genomic.fna
2331	Mucilaginibacter gotjawali	GCA_002355435.1_ASM235543v1_genomic.fna
2332	Aurantimicrobium minutum	GCA_002355535.1_ASM235553v1_genomic.fna
2333	Sulfuricaulis limicola	GCA_002355735.1_ASM235573v1_genomic.fna
2334	Marinifilaceae bacterium	GCA_002356295.1_ASM235629v1_genomic.fna
2335	Mycobacterium shigaense	GCA_002356315.1_ASM235631v1_genomic.fna
2336	Mycobacterium stephanolepidis	GCA_002356335.1_ASM235633v1_genomic.fna
2337	Thiohalobacter thiocyanaticus	GCA_002356355.1_ASM235635v1_genomic.fna
2338	Rhodobacteraceae bacterium	GCA_002356635.1_ASM235663v1_genomic.fna
2339	Ichthyobacterium seriolicida	GCA_002369955.1_ASM236995v1_genomic.fna
2340	Murdochiella vaginalis	GCA_900119705.1_PRJEB14245_genomic.fna
2341	Mesoplasma lactucae	GCA_002441935.1_ASM244193v1_genomic.fna
2342	Staphylococcus nepalensis	GCA_002442895.1_ASM244289v1_genomic.fna
2343	Pseudodesulfovibrio profundus	GCA_900217235.1_DPRO_PRJEB22548_v1_genomic.fna

2344	Sodalis endosymbiont	GCA_900161835.1_HBAv1_genomic.fna
2345	Celeribacter manganoxidans	GCA_002504165.1_ASM250416v1_genomic.fna
2346	Altererythrobacter mangrovi	GCA_002269345.1_ASM226934v1_genomic.fna
2347	Brachybacterium ginsengisoli	GCA_002407065.1_ASM240706v1_genomic.fna
2348	Phaeobacter piscinae	GCA_002407245.1_ASM240724v1_genomic.fna
2349	Phaeobacter piscinae	GCA_002412025.1_ASM241202v1_genomic.fna
2350	Staphylococcus nepalensis	GCA_002442935.1_ASM244293v1_genomic.fna
2351	Antarctobacter heliothermus	GCA_002237555.1_ASM223755v1_genomic.fna
2352	Neisseria zoodegmatis	GCA_900187305.1_51470_F02_genomic.fna
2353	Mycobacterium terrae	GCA_900187145.1_50818_H01_genomic.fna
2354	Janibacter indicus	GCA_001889125.1_ASM188912v1_genomic.fna
2355	Blattabacterium cuenoti	GCA_002355135.1_ASM235513v1_genomic.fna
2356	Celeribacter ethanolicus	GCA_002407265.1_ASM240726v1_genomic.fna
2357	Elizabethkingia endophytica	GCA_002022085.1_ASM202208v1_genomic.fna
2358	Campylobacter pinnipediorum	GCA_002021945.1_ASM202194v1_genomic.fna
2359	Filimonas lacunae	GCA_002355595.1_F._lacunae_YT21-2_genomic.fna
2360	Bartonella apis	GCA_002007485.1_ASM200748v1_genomic.fna
2361	Borrelia mayonii	GCA_001936295.1_ASM193629v1_genomic.fna
2362	Corynebacterium cystitidis	GCA_900187295.1_52191_E01_genomic.fna
2363	Clostridium cochlearium	GCA_900187165.1_52121_G02_genomic.fna
2364	Clostridium formicaceticum	GCA_002080475.1_ASM208047v1_genomic.fna
2365	Pectobacterium parmentieri	GCA_001742145.1_ASM174214v1_genomic.fna
2366	Burkholderia stabilis	GCA_002356275.1_ASM235627v1_genomic.fna
2367	Bordetella bronchialis	GCA_001676745.1_ASM167674v1_genomic.fna
2368	Lactobacillus backii	GCA_001663655.1_ASM166365v1_genomic.fna
2369	Enterococcus thailandicus	GCA_002290025.1_ASM229002v1_genomic.fna
2370	Kluyvera georgiana	GCA_002211745.1_ASM221174v1_genomic.fna
2371	Ralstonia insidiosa	GCA_001663855.1_ASM166385v1_genomic.fna
2372	Polynucleobacter asymbioticus	GCA_001874545.1_ASM187454v1_genomic.fna
2373	Pseudomonas koreensis	GCA_001654515.1_ASM165451v1_genomic.fna
2374	Planococcus maritimus	GCA_001687625.2_ASM168762v2_genomic.fna
2375	Collimonas pratensis	GCA_001584225.1_ASM158422v1_genomic.fna
2376	Enterobacter cloacae	GCA_000750275.1_ASM75027v1_genomic.fna
2377	Corynebacterium simulans	GCA_001586235.1_ASM158623v1_genomic.fna

2378	Paracoccus aminovorans	GCA_900005615.1_JCM7685_genomic.fna
2379	Enterobacter kobei	GCA_001729765.1_ASM172976v1_genomic.fna
2380	Alteromonas mediterranea	GCA_000310085.1_ASM31008v1_genomic.fna
2381	Legionella spiritensis	GCA_900186965.1_50592_C01_genomic.fna
2382	Legionella waltersii	GCA_900187095.1_51699_A01_genomic.fna
2383	Enterococcus silesiacus	GCA_001465115.1_ASM146511v1_genomic.fna
2384	Pseudoalteromonas issachenkonii	GCA_002310795.1_ASM231079v1_genomic.fna
2385	Bacillus glycinifermentans	GCA_002443095.1_ASM244309v1_genomic.fna
2386	Lysobacter enzymogenes	GCA_002355975.1_ASM235597v1_genomic.fna
2387	Bacillus paralicheniformis	GCA_000876525.1_ASM87652v1_genomic.fna
2388	Streptomyces pactum	GCA_002005225.1_ASM200522v1_genomic.fna
2389	Blastochloris viridis	GCA_001459775.1_Blastochloris_viridis_genome_genomic.fna
2390	Lactobacillus paracollinoides	GCA_001702155.1_ASM170215v1_genomic.fna
2391	Lactobacillus lindneri	GCA_001702115.1_ASM170211v1_genomic.fna
2392	Mycobacterium chimaera	GCA_002166795.1_ASM216679v1_genomic.fna
2393	Bosea vaviloviae	GCA_001741865.1_ASM174186v1_genomic.fna
2394	Arthrobacter alpinus	GCA_001445575.1_ASM144557v1_genomic.fna
2395	Lawsonella clevelandensis	GCA_001281505.1_ASM128150v1_genomic.fna
2396	Oblitimonas alkaliphila	GCA_001267195.1_ASM126719v1_genomic.fna
2397	Spiroplasma turonicum	GCA_001513715.1_ASM151371v1_genomic.fna
2398	Staphylococcus schleiferi	GCA_001188875.1_ASM118887v1_genomic.fna
2399	Mycobacterium aurum	GCA_900078775.1_ASM90007877v1_genomic.fna
2400	Brenneria goodwinii	GCA_002291445.1_ASM229144v1_genomic.fna
2401	Klebsiella quasipneumoniae	GCA_002240295.1_ASM224029v1_genomic.fna
2402	Staphylococcus condimenti	GCA_001922405.1_ASM192240v1_genomic.fna
2403	Pandoraea thiooxydans	GCA_001017775.3_ASM101777v3_genomic.fna
2404	Brevibacterium flavum	GCA_001683055.1_ASM168305v1_genomic.fna
2405	Ralstonia mannitolilytica	GCA_001628775.1_ASM162877v1_genomic.fna
2406	Tatlockia micdadei	GCA_002085715.1_ASM208571v1_genomic.fna
2407	Campylobacter iguaniorum	GCA_001483985.1_ASM148398v1_genomic.fna
2408	Mycobacterium immunogenum	GCA_001677135.1_ASM167713v1_genomic.fna
2409	Marinobacter salarius	GCA_002116735.1_ASM211673v1_genomic.fna
2410	Pandoraea apista	GCA_001010785.1_ASM101078v1_genomic.fna
2411	Pandoraea sputorum	GCA_900187205.1_52191_D01_genomic.fna

2412	Streptomyces pluripotens	GCA_000816465.4_ASM81646v4_genomic.fna
2413	Pseudomonas frederiksbergensis	GCA_001952935.1_ASM195293v1_genomic.fna
2414	Fusobacterium hwasookii	GCA_001455105.1_ASM145510v1_genomic.fna
2415	Clostridium baratii	GCA_001991075.1_ASM199107v1_genomic.fna
2416	Collimonas arenae	GCA_001584165.1_ASM158416v1_genomic.fna
2417	Citrobacter braakii	GCA_002208845.1_ASM220884v1_genomic.fna
2418	Klebsiella michiganensis	GCA_000276705.2_ASM27670v2_genomic.fna
2419	Amycolatopsis lurida	GCA_000749465.2_ASM74946v2_genomic.fna
2420	Caulobacter henricii	GCA_001414055.1_ASM141405v1_genomic.fna
2421	Corynebacterium imitans	GCA_900187215.1_52295_D01_genomic.fna
2422	Chryseobacterium piperi	GCA_002285635.1_ASM228563v1_genomic.fna
2423	Alteromonas australica	GCA_000934525.1_ASM93452v1_genomic.fna
2424	Sinorhizobium americanum	GCA_001889105.1_ASM188910v1_genomic.fna
2425	Kozakia baliensis	GCA_001787355.1_ASM178735v1_genomic.fna
2426	Edwardsiella piscicida	GCA_001729265.1_ASM172926v1_genomic.fna
2427	Mycoplasma californicum	GCA_000829335.1_ASM82933v1_genomic.fna
2428	Legionella lansingensis	GCA_900187355.1_51342_E02_genomic.fna
2429	Lysobacter capsici	GCA_002355295.1_ASM235529v1_genomic.fna
2430	Bacillus velezensis	GCA_000283695.1_ASM28369v1_genomic.fna
2431	Ensifer sojae	GCA_002288525.1_ASM228852v1_genomic.fna
2432	Burkholderia lata	GCA_001718575.1_ASM171857v1_genomic.fna
2433	Chlamydia gallinacea	GCA_002007725.1_ASM200772v1_genomic.fna
2434	Kosakonia sacchari	GCA_001683395.1_ASM168339v1_genomic.fna
2435	Bifidobacterium kashiwanohense	GCA_001042615.1_ASM104261v1_genomic.fna
2436	Pandoraea pnomenusa	GCA_000504585.2_ASM50458v2_genomic.fna
2437	Pseudomonas mosselii	GCA_002309555.1_ASM230955v1_genomic.fna
2438	Capnocytophaga haemolytica	GCA_900187255.1_51184_F02_genomic.fna
2439	Streptomyces lydicus	GCA_000952035.2_ASM95203v2_genomic.fna
2440	Desulfococcus multivorans	GCA_002009335.2_ASM200933v2_genomic.fna
2441	Campylobacter subantarcticus	GCA_000816305.1_ASM81630v1_genomic.fna
2442	Clostridium chauvoei	GCA_002327185.1_ASM232718v1_genomic.fna
2443	Bradyrhizobium diazoefficiens	GCA_001549695.1_ASM154969v1_genomic.fna
2444	Cellulophaga baltica	GCA_000477035.2_ASM47703v2_genomic.fna
2445	Clostridium saccharobutylicum	GCA_002003285.1_ASM200328v1_genomic.fna

2446	Clostridium autoethanogenum	GCA_001484725.1_ASM148472v1_genomic.fna
2447	Mesoplasma chauliocola	GCA_002290085.1_ASM229008v1_genomic.fna
2448	Enterobacter lignolyticus	GCA_001461805.1_ASM146180v1_genomic.fna
2449	Paenibacillus durus	GCA_000993825.1_ASM99382v1_genomic.fna
2450	Serratia fonticola	GCA_001514455.1_ASM151445v1_genomic.fna
2451	Bordetella hinzii	GCA_001078295.1_ASM107829v1_genomic.fna
2452	Azotobacter chroococcum	GCA_002220155.1_ASM222015v1_genomic.fna
2453	Dyella japonica	GCA_000725385.1_ASM72538v1_genomic.fna
2454	Bacillus cohnii	GCA_002250055.1_ASM225005v1_genomic.fna
2455	Bifidobacterium thermophilum	GCA_000347695.1_ASM34769v1_genomic.fna
2456	Mycobacterium yongonense	GCA_002148175.1_ASM214817v1_genomic.fna
2457	Nocardiopsis gilva	GCA_002263495.1_ASM226349v1_genomic.fna
2458	Borrelia miyamotoi	GCA_000807295.1_ASM80729v1_genomic.fna
2459	Rathayibacter toxicus	GCA_000875675.1_ASM87567v1_genomic.fna
2460	Aerococcus urinaeequi	GCA_001543205.1_ASM154320v1_genomic.fna
2461	Sporosarcina ureae	GCA_002081995.1_ASM208199v1_genomic.fna
2462	Staphylococcus pasteuri	GCA_002442915.1_ASM244291v1_genomic.fna
2463	Mannheimia varigena	GCA_000521685.1_ASM52168v1_genomic.fna
2464	Streptomyces albulus	GCA_000963515.1_ASM96351v1_genomic.fna
2465	Lysobacter antibioticus	GCA_001442745.1_ASM144274v1_genomic.fna
2466	Bifidobacterium pseudolongum	GCA_002282915.1_ASM228291v1_genomic.fna
2467	Butyrivibrio hungatei	GCA_001858005.1_ASM185800v1_genomic.fna
2468	Weissella ceti	GCA_000732905.1_ASM73290v1_genomic.fna
2469	Corynebacterium stationis	GCA_001561975.1_ASM156197v1_genomic.fna
2470	Brochothrix thermosphacta	GCA_002381265.1_ASM238126v1_genomic.fna
2471	Raoultella ornithinolytica	GCA_000829965.1_ASM82996v1_genomic.fna
2472	Serratia liquefaciens	GCA_000975245.1_ASM97524v1_genomic.fna
2473	Tepidanaerobacter acetatoxydans	GCA_000213235.1_ASM21323v1_genomic.fna
2474	Liberibacter crescens	GCA_001543305.1_ASM154330v1_genomic.fna
2475	Desulfovibrio desulfuricans	GCA_000022125.1_ASM2212v1_genomic.fna
2476	Cellulosimicrobium cellulans	GCA_002162335.1_ASM216233v1_genomic.fna
2477	Cystobacter fuscus	GCA_002305875.1_ASM230587v1_genomic.fna
2478	Bibersteinia trehalosi	GCA_000521725.1_ASM52172v1_genomic.fna
2479	Clostridium stercorarium	GCA_000349885.2_ASM34988v2_genomic.fna

2480	Citrobacter amalonaticus	GCA_001558935.1_ASM155893v1_genomic.fna
2481	Achromobacter denitrificans	GCA_002205315.1_ASM220531v1_genomic.fna
2482	Pseudomonas protegens	GCA_000828695.1_ASM82869v1_genomic.fna
2483	Nitratireductor basaltis	GCA_001953055.1_ASM195305v1_genomic.fna
2484	Sphingobium cloacae	GCA_002355855.1_ASM235585v1_genomic.fna
2485	Dickeya solani	GCA_000758345.1_ASM75834v1_genomic.fna
2486	Sphingobacterium mizutaii	GCA_900187125.1_50592_G01_genomic.fna
2487	Lelliottia amnigena	GCA_002393405.1_ASM239340v1_genomic.fna
2488	Pseudothermotoga hypogea	GCA_000504105.1_ASM50410v1_genomic.fna
2489	Vibrio natriegens	GCA_001680025.1_ASM168002v1_genomic.fna
2490	Sphingopyxis macrogoltabida	GCA_001307295.1_ASM130729v1_genomic.fna
2491	Chryseobacterium indologenes	GCA_002025665.1_ASM202566v1_genomic.fna
2492	Cedecea neteri	GCA_000758305.1_ASM75830v1_genomic.fna
2493	Elizabethkingia meningoseptica	GCA_002022145.1_ASM202214v1_genomic.fna
2494	Pseudomonas parafulva	GCA_002021815.1_ASM202181v1_genomic.fna
2495	Pseudomonas citronellolis	GCA_001586155.1_ASM158615v1_genomic.fna
2496	Pseudomonas azotoformans	GCA_002007785.1_ASM200778v1_genomic.fna
2497	Nocardia seriolae	GCA_002356035.1_ASM235603v1_genomic.fna
2498	Thalassolituus oleivorans	GCA_000582555.1_ASM58255v1_genomic.fna
2499	Rhizobium gallicum	GCA_001908615.1_ASM190861v1_genomic.fna
2500	Corynebacterium halotolerans	GCA_000341345.1_ASM34134v1_genomic.fna
2501	Streptococcus merionis	GCA_900187085.1_50624_E01_genomic.fna
2502	Aeribacillus pallidus	GCA_002243625.1_ASM224362v1_genomic.fna
2503	Xanthomonas translucens	GCA_900094325.1_PBasm1_genomic.fna
2504	Capnocytophaga cynodegmi	GCA_002302475.1_ASM230247v1_genomic.fna
2505	Mycoplasma bovigenitalium	GCA_002356075.1_ASM235607v1_genomic.fna
2506	Bacillus safensis	GCA_001938665.1_ASM193866v1_genomic.fna
2507	Kosakonia radicincitans	GCA_001887675.1_ASM188767v1_genomic.fna
2508	Bordetella holmesii	GCA_000341465.2_ASM34146v2_genomic.fna
2509	Leptolyngbya boryana	GCA_002142475.1_ASM214247v1_genomic.fna
2510	Acidipropionibacterium acidipropionici	GCA_000310065.1_ASM31006v1_genomic.fna
2511	Helicobacter cetorum	GCA_000259255.1_ASM25925v1_genomic.fna
2512	Paraburkholderia caribensis	GCA_001449005.1_ASM144900v1_genomic.fna
2513	Staphylococcus equorum	GCA_001682235.1_ASM168223v1_genomic.fna

2514	Alteromonas stellipolaris	GCA_001433715.1_ASM143371v1_genomic.fna
2515	Cutibacterium granulosum	GCA_900186975.1_50569_F01_genomic.fna
2516	Phaeobacter inhibens	GCA_000154765.2_ASM15476v2_genomic.fna
2517	Alcaligenes faecalis	GCA_001641975.2_ASM164197v2_genomic.fna
2518	Amycolatopsis orientalis	GCA_000943515.2_ASM94351v2_genomic.fna
2519	Serratia plymuthica	GCA_000214235.1_ASM21423v1_genomic.fna
2520	Dermatophilus congolensis	GCA_900187045.1_51485_B01_genomic.fna
2521	Pseudomonas chlororaphis	GCA_000761195.1_ASM76119v1_genomic.fna
2522	Solibacillus silvestris	GCA_000271325.1_ASM27132v1_genomic.fna
2523	Geobacillus thermoleovorans	GCA_000236605.1_ASM23660v1_genomic.fna
2524	Sphingomonas melonis	GCA_002504265.1_ASM250426v1_genomic.fna
2525	Moraxella osloensis	GCA_002355615.1_ASM235561v1_genomic.fna
2526	Nocardia brasiliensis	GCA_002209125.1_ASM220912v1_genomic.fna
2527	Pseudomonas fragi	GCA_002128325.1_ASM212832v1_genomic.fna
2528	Xanthomonas fragariae	GCA_001705545.1_ASM170554v1_genomic.fna
2529	Pseudoalteromonas luteoviolacea	GCA_001750185.1_ASM175018v1_genomic.fna
2530	Cronobacter malonaticus	GCA_001277215.2_ASM127721v2_genomic.fna
2531	Rhizobium phaseoli	GCA_001664105.1_ASM166410v1_genomic.fna
2532	Citrobacter werkmanii	GCA_002386385.1_ASM238638v1_genomic.fna
2533	Pseudomonas monteilii	GCA_000510285.1_ASM51028v1_genomic.fna
2534	Mycoplasma canis	GCA_000988065.1_ASM98806v1_genomic.fna
2535	Elizabethkingia anophelis	GCA_000240095.3_ASM24009v3_genomic.fna
2536	Rickettsia rhipicephali	GCA_001442475.1_ASM144247v1_genomic.fna
2537	Piscirickettsia salmonis	GCA_000756415.3_ASM75641v3_genomic.fna
2538	Francisella noatunensis	GCA_000505725.1_ASM50572v1_genomic.fna
2539	Francisella persica	GCA_001275365.1_ASM127536v1_genomic.fna
2540	Mycobacterium thermoresistibile	GCA_900187065.1_50465_H02_genomic.fna
2541	Enterococcus mundtii	GCA_002216705.1_ASM221670v1_genomic.fna
2542	Rhodococcus fascians	GCA_001620305.1_ASM162030v1_genomic.fna
2543	Leptospirillum ferriphilum	GCA_000695975.1_ASM69597v1_genomic.fna
2544	Flavobacterium columnare	GCA_001534645.1_ASM153464v1_genomic.fna
2545	Rhodococcus pyridinivorans	GCA_002269365.1_ASM226936v1_genomic.fna
2546	Campylobacter sputorum	GCA_002220755.1_ASM222075v1_genomic.fna
2547	Campylobacter hyointestinalis	GCA_001643975.1_ASM164397v1_genomic.fna

2548	Enterococcus durans	GCA_001267865.1_ASM126786v1_genomic.fna
2549	Neorhizobium galegae	GCA_000731295.1_RG1141_Ch_pA_pB_genomic.fna
2550	Clostridium saccharoperbutylacetonicum	GCA_002003305.1_ASM200330v1_genomic.fna
2551	Streptomyces cattleya	GCA_000237305.1_ASM23730v1_genomic.fna
2552	Staphylococcus simiae	GCA_900187055.1_50377_D01_genomic.fna
2553	Halobacillus halophilus	GCA_002209425.1_ASM220942v1_genomic.fna
2554	Azospirillum lipoferum	GCA_000283655.1_ASM28365v1_genomic.fna
2555	Mesorhizobium australicum	GCA_000230995.3_ASM23099v3_genomic.fna
2556	Spiroplasma mirum	GCA_000517365.1_ASM51736v1_genomic.fna
2557	Moraxella bovoculi	GCA_000988615.1_ASM98861v1_genomic.fna
2558	Spiroplasma eriocheiris	GCA_002028345.1_ASM202834v1_genomic.fna
2559	Actinobacillus suis	GCA_900187155.1_51726_C01_genomic.fna
2560	Pediococcus claussenii	GCA_001702215.1_ASM170221v1_genomic.fna
2561	Bacillus simplex	GCA_002243645.1_ASM224364v1_genomic.fna
2562	Rickettsia japonica	GCA_002356695.1_ASM235669v1_genomic.fna
2563	Pseudomonas pseudoalcaligenes	GCA_000953455.1_Ppseudo_Pac_genomic.fna
2564	Staphylococcus xylosus	GCA_000709415.1_ASM70941v1_genomic.fna
2565	Rickettsia slovaca	GCA_000252365.1_ASM25236v1_genomic.fna
2566	Vibrio campbellii	GCA_000464435.1_ASM46443v1_genomic.fna
2567	Azospirillum brasilense	GCA_000632475.2_ASM63247v2_genomic.fna
2568	Streptomyces ambofaciens	GCA_001632865.1_ASM163286v1_genomic.fna
2569	Clostridium pasteurianum	GCA_000389635.1_ASM38963v1_genomic.fna
2570	Tannerella forsythia	GCA_001547855.1_ASM154785v1_genomic.fna
2571	Prevotella intermedia	GCA_000261025.1_ASM26102v1_genomic.fna
2572	Cutibacterium avidum	GCA_001714705.1_ASM171470v1_genomic.fna
2573	Morganella morganii	GCA_001558895.1_ASM155889v1_genomic.fna
2574	Pediococcus damnosus	GCA_001611035.1_ASM161103v1_genomic.fna
2575	Micavibrio aeruginosavorus	GCA_000348745.1_ASM34874v1_genomic.fna
2576	Tatumella citrea	GCA_002163605.1_ASM216360v1_genomic.fna
2577	Lactobacillus curvatus	GCA_001663835.1_ASM166383v1_genomic.fna
2578	Streptomyces venezuelae	GCA_001406115.1_Streptomyces_venezuelae_ATCC_15439_genomic.fna
2579	Hafnia alvei	GCA_001559255.1_ASM155925v1_genomic.fna
2580	Selenomonas ruminantium	GCA_000284095.1_ASM28409v1_genomic.fna
2581	Ornithobacterium rhinotracheale	GCA_000756505.1_ASM75650v1_genomic.fna

2582	Tetragenococcus halophilus	GCA_001712815.1_ASM171281v1_genomic.fna
2583	Enterococcus hirae	GCA_001641305.1_ASM164130v1_genomic.fna
2584	Burkholderia cepacia	GCA_000292915.1_ASM29291v1_genomic.fna
2585	Fervidobacterium pennivorans	GCA_001644665.1_ASM164466v1_genomic.fna
2586	Pantoea agglomerans	GCA_001558735.1_ASM155873v1_genomic.fna
2587	Melissococcus plutonius	GCA_000283975.1_ASM28397v1_genomic.fna
2588	Rickettsia amblyommatis	GCA_000284055.1_ASM28405v1_genomic.fna
2589	Pseudomonas brassicacearum	GCA_000585995.1_ASM58599v1_genomic.fna
2590	Mycobacterium abscessus	GCA_000758405.1_ASM75840v1_genomic.fna
2591	Paenibacillus mucilaginosus	GCA_000218915.1_ASM21891v1_genomic.fna
2592	Burkholderia gladioli	GCA_000194745.1_ASM19474v1_genomic.fna
2593	Aeromonas veronii	GCA_001593245.1_ASM159324v1_genomic.fna
2594	Chlamydia pecorum	GCA_000470765.1_ASM47076v1_genomic.fna
2595	Xanthomonas gardneri	GCA_001908755.1_ASM190875v1_genomic.fna
2596	Corynebacterium ulcerans	GCA_000215665.1_ASM21566v1_genomic.fna
2597	Staphylococcus pseudintermedius	GCA_000189495.1_ASM18949v1_genomic.fna
2598	Lactobacillus farciminis	GCA_002706745.1_ASM270674v1_genomic.fna
2599	Klebsiella aerogenes	GCA_000334515.1_ASM33451v1_genomic.fna
2600	Jonesia denitrificans	GCA_002209145.1_ASM220914v1_genomic.fna
2601	Nocardiopsis dassonvillei	GCA_001877055.1_ASM187705v1_genomic.fna
2602	Conexibacter woesei	GCA_000025265.1_ASM2526v1_genomic.fna
2603	Lactobacillus coryniformis	GCA_002706425.1_ASM270642v1_genomic.fna
2604	Leuconostoc gelidum	GCA_000298875.1_ASM29887v1_genomic.fna
2605	Xanthomonas perforans	GCA_001908855.1_ASM190885v1_genomic.fna
2606	Xanthomonas vesicatoria	GCA_001908815.1_ASM190881v1_genomic.fna
2607	Weissella cibaria	GCA_001932695.1_ASM193269v1_genomic.fna
2608	Prevotella dentalis	GCA_000242335.3_ASM24233v3_genomic.fna
2609	Enterobacter hormaechei	GCA_000807405.4_ASM80740v4_genomic.fna
2610	Rhodothermus marinus	GCA_000224745.1_ASM22474v1_genomic.fna
2611	Halomonas elongata	GCA_000196875.1_ASM19687v1_genomic.fna
2612	Pseudoxanthomonas suwonensis	GCA_000185965.1_ASM18596v1_genomic.fna
2613	Sphingobium yanoikuyae	GCA_002504085.1_ASM250408v1_genomic.fna
2614	Eubacterium limosum	GCA_000152245.2_ASM15224v2_genomic.fna
2615	Staphylococcus simulans	GCA_002386185.1_ASM238618v1_genomic.fna

2616	Mycoplasma hominis	GCA_000767725.1_ASM76772v1_genomic.fna
2617	Vibrio anguillarum	GCA_000462975.1_ASM46297v1_genomic.fna
2618	Vibrio scophthalmi	GCA_001687805.1_ASM168780v1_genomic.fna
2619	Aerococcus urinae	GCA_000193205.1_ASM19320v1_genomic.fna
2620	Cellulophaga lytica	GCA_000750195.1_ASM75019v1_genomic.fna
2621	Capnocytophaga canimorsus	GCA_002302445.1_ASM230244v1_genomic.fna
2622	Corynebacterium variabile	GCA_000179395.2_ASM17939v2_genomic.fna
2623	Pseudomonas fulva	GCA_002688705.1_ASM268870v1_genomic.fna
2624	Streptococcus parasanguinis	GCA_000262145.1_ASM26214v1_genomic.fna
2625	Brachyspira pilosicoli	GCA_000143725.1_ASM14372v1_genomic.fna
2626	Eggerthella lenta	GCA_002148255.1_ASM214825v1_genomic.fna
2627	Citrobacter freundii	GCA_001022155.1_ASM102215v1_genomic.fna
2628	Ketogulonicigenium vulgare	GCA_000164885.1_ASM16488v1_genomic.fna
2629	Taylorella equigenitalis	GCA_000185745.1_ASM18574v1_genomic.fna
2630	Mycoplasma putrefaciens	GCA_000376625.1_ASM37662v1_genomic.fna
2631	Streptococcus gallolyticus	GCA_000027185.1_ASM2718v1_genomic.fna
2632	Pantoea vagans	GCA_001506165.1_ASM150616v1_genomic.fna
2633	Odoribacter splanchnicus	GCA_900187175.1_51342_A02_genomic.fna
2634	Amycolatopsis mediterranei	GCA_000220945.1_ASM22094v1_genomic.fna
2635	Mycoplasma suis	GCA_000203215.1_ASM20321v1_genomic.fna
2636	Streptococcus anginosus	GCA_000463465.1_ASM46346v1_genomic.fna
2637	Pantoea ananatis	GCA_000233595.1_ASM23359v1_genomic.fna
2638	Streptococcus constellatus	GCA_000463425.1_ASM46342v1_genomic.fna
2639	Staphylococcus lugdunensis	GCA_000270465.1_ASM27046v1_genomic.fna
2640	Lactobacillus amylovorus	GCA_000194115.1_ASM19411v1_genomic.fna
2641	Riemerella anatipestifer	GCA_000183155.1_ASM18315v1_genomic.fna
2642	Acinetobacter pittii	GCA_001577285.1_ASM157728v1_genomic.fna
2643	Xanthomonas campestris	GCA_001854165.1_ASM185416v1_genomic.fna
2644	Mesorhizobium ciceri	GCA_000185905.1_ASM18590v1_genomic.fna
2645	Ralstonia eutropha	GCA_000009285.2_ASM928v2_genomic.fna
2646	Alicycliphilus denitrificans	GCA_000179015.2_ASM17901v2_genomic.fna
2647	Veillonella parvula	GCA_002005185.1_ASM200518v1_genomic.fna
2648	Enterobacter asburiae	GCA_000632395.1_ASM63239v1_genomic.fna
2649	Corynebacterium pseudotuberculosis	GCA_000143705.1_ASM14370v1_genomic.fna

2650	Parageobacillus thermoglucosidasius	GCA_000178395.2_ASM17839v2_genomic.fna
2651	Sulfobacillus acidophilus	GCA_000237975.1_ASM23797v1_genomic.fna
2652	Vibrio coralliilyticus	GCA_000772065.1_ASM77206v1_genomic.fna
2653	Pediococcus acidilactici	GCA_001922325.1_ASM192232v1_genomic.fna
2654	Vibrio mimicus	GCA_001767355.1_ASM176735v1_genomic.fna
2655	Vibrio fluvialis	GCA_001952955.1_ASM195295v1_genomic.fna
2656	Spirochaeta thermophila	GCA_000147075.1_ASM14707v1_genomic.fna
2657	Acinetobacter calcoaceticus	GCA_900093475.1_36734_D01_2_genomic.fna
2658	Corynebacterium ammoniagenes	GCA_002005245.1_ASM200524v1_genomic.fna
2659	Pseudomonas savastanoi	GCA_000164015.3_ASM16401v3_genomic.fna
2660	Xanthomonas fuscans	GCA_001610795.1_ASM161079v1_genomic.fna
2661	Acidovorax citrulli	GCA_002441975.1_ASM244197v1_genomic.fna
2662	Megamonas hypermegale	GCA_900187035.1_50279_G02_genomic.fna
2663	Achromobacter xylosoxidans	GCA_000165835.1_ASM16583v1_genomic.fna
2664	Streptococcus oralis	GCA_001983955.1_ASM198395v1_genomic.fna
2665	Acinetobacter nosocomialis	GCA_002082665.1_ASM208266v1_genomic.fna
2666	Klebsiella variicola	GCA_000812205.2_ASM81220v2_genomic.fna
2667	Bacillus atrophaeus	GCA_000165925.1_ASM16592v1_genomic.fna
2668	Erysipelothrix rhusiopathiae	GCA_000404205.1_ASM40420v1_genomic.fna
2669	Oxalobacter formigenes	GCA_002127965.1_ASM212796v1_genomic.fna
2670	Eikenella corrodens	GCA_900187105.1_50618_F01_genomic.fna
2671	Staphylococcus capitis	GCA_002356175.1_ASM235617v1_genomic.fna
2672	Helicobacter cinaedi	GCA_000284635.1_ASM28463v1_genomic.fna
2673	Blautia hansenii	GCA_002222595.2_ASM222259v2_genomic.fna
2674	Lactobacillus paracasei	GCA_000829035.1_ASM82903v1_genomic.fna
2675	Capnocytophaga sputigena	GCA_002302495.1_ASM230249v1_genomic.fna
2676	Weissella paramesenteroides	GCA_002386265.1_ASM238626v1_genomic.fna
2677	Bifidobacterium angulatum	GCA_000966445.2_ASM96644v2_genomic.fna
2678	Providencia alcalifaciens	GCA_002393505.1_ASM239350v1_genomic.fna
2679	Capnocytophaga gingivalis	GCA_002302415.1_ASM230241v1_genomic.fna
2680	Prevotella melaninogenica	GCA_002208725.1_ASM220872v1_genomic.fna
2681	Gardnerella vaginalis	GCA_000159155.2_ASM15915v2_genomic.fna
2682	Acidithiobacillus caldus	GCA_000221025.1_ASM22102v1_genomic.fna
2683	Staphylococcus carnosus	GCA_001701005.1_ASM170100v1_genomic.fna

2684	Aggregatibacter aphrophilus	GCA_001188835.1_ASM118883v1_genomic.fna
2685	Mycoplasma hyorhinis	GCA_000145705.1_ASM14570v1_genomic.fna
2686	Hydrogenobacter thermophilus	GCA_000164905.1_ASM16490v1_genomic.fna
2687	Thiomonas intermedia	GCA_000092605.1_ASM9260v1_genomic.fna
2688	Streptomyces hygroscopicus	GCA_000340845.1_ASM34084v1_genomic.fna
2689	Streptococcus intermedius	GCA_000306805.1_ASM30680v1_genomic.fna
2690	Hyphomicrobium denitrificans	GCA_000230975.3_ASM23097v3_genomic.fna
2691	Thermoanaerobacterium thermosaccharolyticun	GCA_000328545.1_ASM32854v1_genomic.fna
2692	Aggregatibacter actinomycetemcomitans	GCA_000146265.4_ASM14626v4_genomic.fna
2693	Rothia mucilaginosa	GCA_001548235.1_ASM154823v1_genomic.fna
2694	Dickeya zeae	GCA_000816045.1_ASM81604v1_genomic.fna
2695	Brachyspira hyodysenteriae	GCA_000022105.1_ASM2210v1_genomic.fna
2696	Pectobacterium carotovorum	GCA_000294535.1_ASM29453v1_genomic.fna
2697	Mycobacterium kansasii	GCA_000831305.1_ASM83130v1_genomic.fna
2698	Laribacter hongkongensis	GCA_002215055.1_ASM221505v1_genomic.fna
2699	Rhodococcus opacus	GCA_000599545.1_ASM59954v1_genomic.fna
2700	Alicyclobacillus acidocaldarius	GCA_000219875.1_ASM21987v1_genomic.fna
2701	Yersinia ruckeri	GCA_000834255.1_ASM83425v1_genomic.fna
2702	Borrelia hermsii	GCA_000956315.1_ASM95631v1_genomic.fna
2703	Strawberry lethal	GCA_000397185.1_ASM39718v1_genomic.fna
2704	Corynebacterium urealyticum	GCA_000338095.1_ASM33809v1_genomic.fna
2705	Butyrivibrio proteoclasticus	GCA_000145035.1_ASM14503v1_genomic.fna
2706	Lactobacillus buchneri	GCA_000211375.1_ASM21137v1_genomic.fna
2707	Oligotropha carboxidovorans	GCA_000021365.1_ASM2136v1_genomic.fna
2708	Escherichia albertii	GCA_001549955.1_ASM154995v1_genomic.fna
2709	Anoxybacillus flavithermus	GCA_002197485.1_ASM219748v1_genomic.fna
2710	Edwardsiella tarda	GCA_000146305.1_ASM14630v1_genomic.fna
2711	Sorangium cellulosum	GCA_000418325.1_ASM41832v1_genomic.fna
2712	Mycobacterium intracellulare	GCA_000277145.1_ASM27714v1_genomic.fna
2713	Myxococcus fulvus	GCA_000988565.1_ASM98856v1_genomic.fna
2714	Kocuria rhizophila	GCA_002208685.1_ASM220868v1_genomic.fna
2715	Arcobacter butzleri	GCA_000215345.3_ASM21534v3_genomic.fna
2716	Paenibacillus larvae	GCA_000511405.1_ASM51140v1_genomic.fna
2717	Mycoplasma haemofelis	GCA_000200735.1_ASM20073v1_genomic.fna

2718	Lactobacillus sakei	GCA_000026065.1_ASM2606v1_genomic.fna
2719	Idiomarina loihiensis	GCA_000401175.1_ASM40117v1_genomic.fna
2720	Mycoplasma synoviae	GCA_000008245.1_ASM824v1_genomic.fna
2721	Rickettsia typhi	GCA_000277285.1_ASM27728v1_genomic.fna
2722	Bdellovibrio bacteriovorus	GCA_000196175.1_ASM19617v1_genomic.fna
2723	Lactobacillus johnsonii	GCA_000091405.1_ASM9140v1_genomic.fna
2724	Rhodococcus erythropolis	GCA_000010105.1_ASM1010v1_genomic.fna
2725	Macrococcus caseolyticus	GCA_002119825.1_ASM211982v1_genomic.fna
2726	Akkermansia muciniphila	GCA_001688765.2_ASM168876v2_genomic.fna
2727	Clostridium butyricum	GCA_001465175.1_ASM146517v1_genomic.fna
2728	Flavobacterium psychrophilum	GCA_000739395.1_ASM73939v1_genomic.fna
2729	Lysinibacillus sphaericus	GCA_001581875.1_ASM158187v1_genomic.fna
2730	Methylobacterium populi	GCA_002355515.1_ASM235551v1_genomic.fna
2731	Burkholderia ubonensis	GCA_001718355.1_ASM171835v1_genomic.fna
2732	Geobacillus thermodenitrificans	GCA_002072065.1_ASM207206v1_genomic.fna
2733	Pseudomonas mendocina	GCA_000204295.1_ASM20429v1_genomic.fna
2734	Thermosipho melanesiensis	GCA_001941385.1_ASM194138v1_genomic.fna
2735	Campylobacter concisus	GCA_000017725.2_ASM1772v2_genomic.fna
2736	Streptococcus iniae	GCA_001885145.1_ASM188514v1_genomic.fna
2737	Granulibacter bethesdensis	GCA_000576085.2_ASM57608v2_genomic.fna
2738	Pseudomonas stutzeri	GCA_000219605.1_ASM21960v1_genomic.fna
2739	Propionibacterium freudenreichii	GCA_000091725.1_ASM9172v1_genomic.fna
2740	Salinibacter ruber	GCA_000090405.1_ASM9040v2_genomic.fna
2741	Aeromonas hydrophila	GCA_000401555.1_ASM40155v1_genomic.fna
2742	Ramlibacter tataouinensis	GCA_001580455.1_ASM158045v1_genomic.fna
2743	Legionella longbeachae	GCA_002073455.1_ASM207345v1_genomic.fna
2744	Collimonas fungivorans	GCA_001584145.1_ASM158414v1_genomic.fna
2745	Flavobacterium johnsoniae	GCA_001705175.1_ASM170517v1_genomic.fna
2746	Paenibacillus polymyxa	GCA_000146875.2_ASM14687v2_genomic.fna
2747	Herbaspirillum seropedicae	GCA_000143225.1_ASM14322v1_genomic.fna
2748	Mycobacterium abscessus	GCA_000277775.2_ASM27777v2_genomic.fna
2749	Bacillus coagulans	GCA_000169195.2_ASM16919v2_genomic.fna
2750	Psychrobacter cryohalolentis	GCA_002208765.1_ASM220876v1_genomic.fna
2751	Erythrobacter litoralis	GCA_001719165.1_ASM171916v1_genomic.fna

2752	Bifidobacterium breve	GCA_000213865.1_ASM21386v1_genomic.fna
2753	Neisseria lactamica	GCA_002022745.1_ASM202274v1_genomic.fna
2754	Edwardsiella ictaluri	GCA_002076875.1_ASM207687v1_genomic.fna
2755	Pseudoalteromonas tunicata	GCA_002310815.1_ASM231081v1_genomic.fna
2756	Pseudoalteromonas atlantica	GCA_002374815.1_ASM237481v1_genomic.fna
2757	Listeria welshimeri	GCA_900187315.1_52087_C01_genomic.fna
2758	Listeria ivanovii	GCA_000565155.1_ASM56515v1_genomic.fna
2759	Mycoplasma fermentans	GCA_000148625.1_ASM14862v1_genomic.fna
2760	Moraxella catarrhalis	GCA_000740455.1_ASM74045v1_genomic.fna
2761	Xenorhabdus bovienii	GCA_000973125.1_ASM97312v1_genomic.fna
2762	Enterobacter cloacae	GCA_000235765.3_ASM23576v3_genomic.fna
2763	Wolbachia endosymbiont	GCA_000376605.1_ASM37660v1_genomic.fna
2764	Gluconobacter oxydans	GCA_000311765.1_ASM31176v1_genomic.fna
2765	Bacillus clausii	GCA_000737305.2_ASM73730v2_genomic.fna
2766	Lactobacillus salivarius	GCA_000143435.1_ASM14343v1_genomic.fna
2767	Marinobacter hydrocarbonoclasticus	GCA_000015365.1_ASM1536v1_genomic.fna
2768	Nocardia farcinica	GCA_000009805.1_ASM980v1_genomic.fna
2769	Rickettsia canadensis	GCA_000283915.1_ASM28391v1_genomic.fna
2770	Cronobacter sakazakii	GCA_000017665.1_ASM1766v1_genomic.fna
2771	Clostridium beijerinckii	GCA_000016965.1_ASM1696v1_genomic.fna
2772	Citrobacter koseri	GCA_002393245.1_ASM239324v1_genomic.fna
2773	Klebsiella oxytoca	GCA_000714655.1_ASM71465v1_genomic.fna
2774	Proteus mirabilis	GCA_000444425.1_ASM44442v1_genomic.fna
2775	Erwinia amylovora	GCA_000027205.1_ASM2720v1_genomic.fna
2776	Halobacteriovorax marinus	GCA_002442875.1_ASM244287v1_genomic.fna
2777	Mycoplasma bovis	GCA_000219375.1_ASM21937v1_genomic.fna
2778	Campylobacter lari	GCA_000816225.1_ASM81622v1_genomic.fna
2779	Campylobacter coli	GCA_000465235.1_ASM46523v1_genomic.fna
2780	Staphylococcus haemolyticus	GCA_001611955.1_ASM161195v1_genomic.fna
2781	Propionibacterium acnes	GCA_000025765.1_ASM2576v1_genomic.fna
2782	Burkholderia vietnamiensis	GCA_001718495.1_ASM171849v1_genomic.fna
2783	Mesoplasma florum	GCA_000479355.1_ASM47935v1_genomic.fna
2784	Moorella thermoacetica	GCA_001267405.1_ASM126740v1_genomic.fna
2785	Chromobacterium violaceum	GCA_002735945.1_ASM273594v1_genomic.fna

2786	Mycoplasma gallisepticum	GCA_000025365.1_ASM2536v1_genomic.fna
2787	Serratia marcescens	GCA_000330865.1_ASM33086v1_genomic.fna
2788	Brevibacterium linens	GCA_001729525.1_ASM172952v1_genomic.fna
2789	Pediococcus pentosaceus	GCA_000496265.1_ASM49626v1_genomic.fna
2790	Lactobacillus brevis	GCA_000359625.1_ASM35962v1_genomic.fna
2791	Mannheimia haemolytica	GCA_000349765.2_ASM34976v2_genomic.fna
2792	Bartonella henselae	GCA_000612765.1_PRJEB5998_assembly_1_genomic.fna
2793	Lawsonia intracellularis	GCA_000331715.1_ASM33171v1_genomic.fna
2794	Lactobacillus acidophilus	GCA_000389675.2_ASM38967v2_genomic.fna
2795	Clostridium tetani	GCA_000967115.1_CT1_genomic.fna
2796	Bacteroides thetaiotaomicron	GCA_001314975.1_ASM131497v1_genomic.fna
2797	Chlamydia abortus	GCA_000952935.1_CAAB7_genomic.fna
2798	Pectobacterium atrosepticum	GCA_000696465.1_ASM69646v1_genomic.fna
2799	Salmonella bongori	GCA_000439255.1_ASM43925v1_genomic.fna
2800	Anaplasma phagocytophilum	GCA_000439755.1_ASM43975v1_genomic.fna
2801	Lactobacillus plantarum	GCA_000023085.1_ASM2308v1_genomic.fna
2802	Leuconostoc mesenteroides	GCA_000234825.3_ASM23482v3_genomic.fna
2803	Wigglesworthia glossinidia	GCA_000247565.1_ASM24756v1_genomic.fna
2804	Paraburkholderia xenovorans	GCA_000756045.1_ASM75604v1_genomic.fna
2805	Chlamydia muridarum	GCA_000709455.1_ASM70945v1_genomic.fna
2806	Dehalococcoides mccartyi	GCA_000830885.1_ASM83088v1_genomic.fna
2807	Geobacter sulfurreducens	GCA_000210155.1_ASM21015v1_genomic.fna
2808	Yersinia enterocolitica	GCA_000192105.1_ASM19210v1_genomic.fna
2809	Thermotoga maritima	GCA_000230655.3_ASM23065v3_genomic.fna
2810	Mycoplasma pneumoniae	GCA_000143945.1_ASM14394v1_genomic.fna
2811	Mycobacterium smegmatis	GCA_000283295.1_ASM28329v1_genomic.fna
2812	Corynebacterium diphtheriae	GCA_000195815.1_ASM19581v1_genomic.fna
2813	Lactobacillus gasseri	GCA_002158885.1_ASM215888v1_genomic.fna
2814	Streptococcus gordonii	GCA_000970665.2_ASM97066v2_genomic.fna
2815	Clostridium acetobutylicum	GCA_000191905.1_ASM19190v1_genomic.fna
2816	Deinococcus radiodurans	GCA_001638825.1_ASM163882v1_genomic.fna
2817	Rhodospirillum rubrum	GCA_000225955.1_ASM22595v1_genomic.fna
2818	Acidithiobacillus ferrooxidans	GCA_000020825.1_ASM2082v1_genomic.fna
2819	Haemophilus ducreyi	GCA_001647655.1_ASM164765v1_genomic.fna

2820	Bordetella pertussis	GCA_000193595.3_ASM19359v3_genomic.fna
2821	Bordetella parapertussis	GCA_000195695.1_ASM19569v1_genomic.fna
2822	Azotobacter vinelandii	GCA_000021045.1_ASM2104v1_genomic.fna
2823	Mesorhizobium loti	GCA_001676765.1_ASM167676v1_genomic.fna
2824	Bordetella bronchiseptica	GCA_000195675.1_ASM19567v1_genomic.fna
2825	Sinorhizobium meliloti	GCA_000147775.3_ASM14777v3_genomic.fna
2826	Clostridium bolteae	GCA_002234575.2_ASM223457v2_genomic.fna
2827	Collinsella aerofaciens	GCA_002736145.1_ASM273614v1_genomic.fna
2828	Bacteroides ovatus	GCA_900095495.1_B_ovatus_V975_genomic.fna
2829	Eubacterium hallii	GCA_900209925.1_EH1_genomic.fna
2830	Providencia stuartii	GCA_000754345.1_ASM75434v1_genomic.fna
2831	Clostridium sporogenes	GCA_001020205.1_ASM102020v1_genomic.fna
2832	Streptococcus infantarius	GCA_001477615.1_ASM147761v1_genomic.fna
2833	Burkholderia glumae	GCA_000835205.1_ASM83520v1_genomic.fna
2834	Bacillus megaterium	GCA_000832985.1_ASM83298v1_genomic.fna
2835	Brucella melitensis	GCA_000022625.1_ASM2262v1_genomic.fna
2836	Caulobacter crescentus	GCA_000022005.1_ASM2200v1_genomic.fna
2837	Enterococcus gallinarum	GCA_002393265.1_ASM239326v1_genomic.fna
2838	Brucella pinnipedialis	GCA_000740275.1_ASM74027v1_genomic.fna
2839	Fibrobacter succinogenes	GCA_000024665.1_ASM2466v1_genomic.fna
2840	Francisella philomiragia	GCA_000833195.1_ASM83319v1_genomic.fna
2841	Pasteurella multocida	GCA_000006825.1_ASM682v1_genomic.fna
2842	Lactobacillus rhamnosus	GCA_000011045.1_ASM1104v1_genomic.fna
2843	Francisella tularensis	GCA_001880205.1_ASM188020v1_genomic.fna
2844	Acetobacter pasteurianus	GCA_000010825.1_ASM1082v1_genomic.fna
2845	Mycobacterium leprae	GCA_000026685.1_ASM2668v1_genomic.fna
2846	Haemophilus parasuis	GCA_000740985.1_ASM74098v1_genomic.fna
2847	Streptococcus equi	GCA_000020765.1_ASM2076v1_genomic.fna
2848	Zymomonas mobilis	GCA_000175255.2_ASM17525v2_genomic.fna
2849	Bifidobacterium bifidum	GCA_000164965.1_ASM16496v1_genomic.fna
2850	Stenotrophomonas maltophilia	GCA_000020665.1_ASM2066v1_genomic.fna
2851	Alteromonas macleodii	GCA_000299955.1_ASM29995v1_genomic.fna
2852	Yersinia frederiksenii	GCA_002591095.1_ASM259109v1_genomic.fna
2853	Bacillus mycoides	GCA_000018825.1_ASM1882v1_genomic.fna

2854	Neisseria gonorrhoeae	GCA_000020105.1_ASM2010v1_genomic.fna
2855	Enterococcus faecium	GCA_000250945.1_ASM25094v1_genomic.fna
2856	Comamonas testosteroni	GCA_000093145.2_ASM9314v2_genomic.fna
2857	Streptococcus mutans	GCA_000091645.1_ASM9164v1_genomic.fna
2858	Bifidobacterium animalis	GCA_000021425.1_ASM2142v1_genomic.fna
2859	Bacillus amyloliquefaciens	GCA_000195515.1_ASM19551v1_genomic.fna
2860	Chlamydia psittaci	GCA_000191925.1_ASM19192v1_genomic.fna
2861	Borrelia garinii	GCA_000239475.1_ASM23947v1_genomic.fna
2862	Bartonella tribocorum	GCA_000689355.1_PRJEB5999_assembly_1_genomic.fna
2863	Ruminiclostridium thermocellum	GCA_000184925.1_ASM18492v1_genomic.fna
2864	Rhizobium etli	GCA_000020265.1_ASM2026v1_genomic.fna
2865	Streptococcus dysgalactiae	GCA_000010705.1_ASM1070v1_genomic.fna
2866	Gluconacetobacter diazotrophicus	GCA_000067045.1_ASM6704v1_genomic.fna
2867	Microcystis aeruginosa	GCA_000981785.1_ASM98178v1_genomic.fna
2868	Bradyrhizobium japonicum	GCA_000807315.1_ASM80731v1_genomic.fna
2869	Bifidobacterium dentium	GCA_000024445.1_ASM2444v1_genomic.fna
2870	Brucella suis	GCA_000018905.1_ASM1890v1_genomic.fna
2871	Burkholderia ambifaria	GCA_000203915.1_ASM20391v1_genomic.fna
2872	Mycobacterium marinum	GCA_000018345.1_ASM1834v1_genomic.fna
2873	Leptospira biflexa	GCA_000017605.1_ASM1760v1_genomic.fna
2874	Brucella canis	GCA_000238195.1_ASM23819v1_genomic.fna
2875	Anaeromyxobacter dehalogenans	GCA_000013385.1_ASM1338v1_genomic.fna
2876	Clavibacter michiganensis	GCA_000063485.1_ASM6348v1_genomic.fna
2877	Borrelia burgdorferi	GCA_000021405.1_ASM2140v1_genomic.fna
2878	Rhizobium leguminosarum	GCA_000517605.1_ASM51760v1_genomic.fna
2879	Klebsiella pneumoniae	GCA_000009885.1_ASM988v1_genomic.fna
2880	Rickettsia prowazekii	GCA_000022785.1_ASM2278v1_genomic.fna
2881	Ochrobactrum anthropi	GCA_000742955.1_ASM74295v1_genomic.fna
2882	Burkholderia multivorans	GCA_000018505.1_ASM1850v1_genomic.fna
2883	Clostridium botulinum	GCA_000020165.1_ASM2016v1_genomic.fna
2884	Burkholderia oklahomensis	GCA_000959365.1_ASM95936v1_genomic.fna
2885	Phaeobacter gallaeciensis	GCA_001678945.1_ASM167894v1_genomic.fna
2886	Mycoplasma mycoides	GCA_000023165.1_ASM2316v1_genomic.fna
2887	Burkholderia thailandensis	GCA_000385525.1_ASM38552v1_genomic.fna

2888	Ureaplasma parvum	GCA_000006625.1_ASM662v1_genomic.fna
2889	Porphyromonas gingivalis	GCA_000007585.1_ASM758v1_genomic.fna
2890	Clostridium kluyveri	GCA_000010265.1_ASM1026v1_genomic.fna
2891	Orientia tsutsugamushi	GCA_000010205.1_ASM1020v1_genomic.fna
2892	Lactobacillus fermentum	GCA_000210515.1_ASM21051v1_genomic.fna
2893	Methylobacterium extorquens	GCA_000021845.1_ASM2184v1_genomic.fna
2894	Lactococcus garvieae	GCA_000269945.1_ASM26994v1_genomic.fna
2895	Vibrio parahaemolyticus	GCA_000328405.1_ASM32840v1_genomic.fna
2896	Vibrio harveyi	GCA_001558435.1_ASM155843v1_genomic.fna
2897	Borrelia afzelii	GCA_000222835.1_ASM22283v1_genomic.fna
2898	Photobacterium damselae	GCA_002356235.1_ASM235623v1_genomic.fna
2899	Faecalibacterium prausnitzii	GCA_002586945.1_ASM258694v1_genomic.fna
2900	Bifidobacterium adolescentis	GCA_000737885.1_ASM73788v1_genomic.fna
2901	Rickettsia rickettsii	GCA_000018225.1_ASM1822v1_genomic.fna
2902	Geobacillus stearothermophilus	GCA_002300135.1_ASM230013v1_genomic.fna
2903	Lactobacillus helveticus	GCA_000015385.1_ASM1538v1_genomic.fna
2904	Ralstonia pickettii	GCA_000023425.1_ASM2342v1_genomic.fna
2905	Rickettsia bellii	GCA_000018245.1_ASM1824v1_genomic.fna
2906	Desulfovibrio vulgaris	GCA_000195755.1_ASM19575v1_genomic.fna
2907	Bacillus subtilis	GCA_000146565.1_ASM14656v1_genomic.fna
2908	Lactobacillus casei	GCA_000026485.1_ASM2648v1_genomic.fna
2909	Coxiella burnetii	GCA_000017105.1_ASM1710v1_genomic.fna
2910	Desulfitobacterium hafniense	GCA_000010045.1_ASM1004v1_genomic.fna
2911	Aeromonas salmonicida	GCA_002214245.1_ASM221424v1_genomic.fna
2912	Clostridioides difficile	GCA_000085225.1_ASM8522v1_genomic.fna
2913	Xanthomonas oryzae	GCA_000010025.1_ASM1002v1_genomic.fna
2914	Streptococcus mitis	GCA_001281025.1_ASM128102v1_genomic.fna
2915	Xanthomonas axonopodis	GCA_000007165.1_ASM716v1_genomic.fna
2916	Mycoplasma capricolum	GCA_000192395.2_ASM19239v2_genomic.fna
2917	Brucella abortus	GCA_000018725.1_ASM1872v1_genomic.fna
2918	Leptospira borgpetersenii	GCA_000013965.1_ASM1396v1_genomic.fna
2919	Lactobacillus delbrueckii	GCA_000014405.1_ASM1440v1_genomic.fna
2920	Mycoplasma agalactiae	GCA_000089865.1_ASM8986v1_genomic.fna
2921	Francisella tularensis	GCA_000009245.1_ASM924v1_genomic.fna

2922	Yersinia pseudotuberculosis	GCA_000019465.1_ASM1946v1_genomic.fna
2923	Rhodopseudomonas palustris	GCA_000013745.1_ASM1374v1_genomic.fna
2924	Streptococcus salivarius	GCA_000253315.1_ASM25331v1_genomic.fna
2925	Vibrio cholerae	GCA_000016245.1_ASM1624v1_genomic.fna
2926	Mycobacterium ulcerans	GCA_002355775.1_ASM235577v1_genomic.fna
2927	Shigella boydii	GCA_000020185.1_ASM2018v1_genomic.fna
2928	Ralstonia solanacearum	GCA_000215325.1_ASM21532v1_genomic.fna
2929	Corynebacterium jeikeium	GCA_002209185.1_ASM220918v1_genomic.fna
2930	Bacillus thuringiensis	GCA_000497525.2_ASM49752v2_genomic.fna
2931	Ehrlichia chaffeensis	GCA_000632815.1_ASM63281v1_genomic.fna
2932	Burkholderia mallei	GCA_000015465.1_ASM1546v1_genomic.fna
2933	Burkholderia pseudomallei	GCA_000012785.1_ASM1278v1_genomic.fna
2934	Burkholderia cenocepacia	GCA_000014085.1_ASM1408v1_genomic.fna
2935	Mycoplasma genitalium	GCA_000292405.1_ASM29240v1_genomic.fna
2936	Chlamydia trachomatis	GCA_000068585.1_ASM6858v1_genomic.fna
2937	Corynebacterium glutamicum	GCA_000010225.1_ASM1022v1_genomic.fna
2938	Thermus thermophilus	GCA_000008125.1_ASM812v1_genomic.fna
2939	Bacillus pumilus	GCA_000017885.4_ASM1788v4_genomic.fna
2940	Lactobacillus reuteri	GCA_000010005.1_ASM1000v1_genomic.fna
2941	Ehrlichia ruminantium	GCA_000050405.1_ASM5040v1_genomic.fna
2942	Synechococcus elongatus	GCA_000012525.1_ASM1252v1_genomic.fna
2943	Shewanella putrefaciens	GCA_000169215.2_ASM16921v2_genomic.fna
2944	Shewanella baltica	GCA_000015845.1_ASM1584v1_genomic.fna
2945	Streptococcus thermophilus	GCA_000011825.1_ASM1182v1_genomic.fna
2946	Shigella sonnei	GCA_000283715.1_ASM28371v1_genomic.fna
2947	Legionella pneumophila	GCA_000048645.1_ASM4864v1_genomic.fna
2948	Bacteroides fragilis	GCA_000025985.1_ASM2598v1_genomic.fna
2949	Bacillus licheniformis	GCA_000008425.1_ASM842v1_genomic.fna
2950	Anaplasma marginale	GCA_000011945.1_ASM1194v1_genomic.fna
2951	Acinetobacter baumannii	GCA_000018445.1_ASM1844v1_genomic.fna
2952	Chlorobium phaeobacteroides	GCA_000020545.1_ASM2054v1_genomic.fna
2953	Streptococcus suis	GCA_000018185.1_ASM1818v1_genomic.fna
2954	Mycoplasma hyopneumoniae	GCA_000008225.1_ASM822v1_genomic.fna
2955	Vibrio vulnificus	GCA_000009745.1_ASM974v1_genomic.fna

2956	Pseudomonas aeruginosa	GCA_000014625.1_ASM1462v1_genomic.fna
2957	Streptococcus agalactiae	GCA_000012705.1_ASM1270v1_genomic.fna
2958	Haemophilus somnus	GCA_000011785.1_ASM1178v1_genomic.fna
2959	Bifidobacterium longum	GCA_000008945.1_ASM894v1_genomic.fna
2960	Shigella flexneri	GCA_000007405.1_ASM740v1_genomic.fna
2961	Bacillus anthracis	GCA_000008165.1_ASM816v1_genomic.fna
2962	Pseudomonas syringae	GCA_000344335.2_ASM34433v2_genomic.fna
2963	Fusobacterium nucleatum	GCA_000158275.2_ASM15827v2_genomic.fna
2964	Leptospira interrogans	GCA_000007685.1_ASM768v1_genomic.fna
2965	Agrobacterium tumefaciens	GCA_000971565.1_ASM97156v1_genomic.fna
2966	Streptococcus pneumoniae	GCA_000006885.1_ASM688v1_genomic.fna
2967	Streptococcus pyogenes	GCA_000007285.1_ASM728v1_genomic.fna
2968	Pseudomonas putida	GCA_000007565.2_ASM756v2_genomic.fna
2969	Xylella fastidiosa	GCA_000007245.1_ASM724v1_genomic.fna
2970	Neisseria meningitidis	GCA_000009105.1_ASM910v1_genomic.fna
2971	Buchnera aphidicola	GCA_000007725.1_ASM772v1_genomic.fna
2972	Chlamydophila pneumoniae	GCA_000007205.1_ASM720v1_genomic.fna
2973	Helicobacter pylori	GCA_000008785.1_ASM878v1_genomic.fna
2974	Escherichia coli	GCA_000026345.1_ASM2634v1_genomic.fna
2975	Haemophilus influenzae	GCA_000012185.1_ASM1218v1_genomic.fna
2976	Prochlorococcus marinus	GCA_000011465.1_ASM1146v1_genomic.fna
2977	Actinobacillus pleuropneumoniae	GCA_000016685.1_ASM1668v1_genomic.fna
2978	Tropheryma whipplei	GCA_000196075.1_ASM19607v1_genomic.fna
2979	Mycobacterium bovis	GCA_000009445.1_ASM944v1_genomic.fna
2980	Mycobacterium tuberculosis	GCA_000008585.1_ASM858v1_genomic.fna
2981	Clostridium perfringens	GCA_000009685.1_ASM968v1_genomic.fna
2982	Bacillus cereus	GCA_000008005.1_ASM800v1_genomic.fna
2983	Listeria monocytogenes	GCA_000008285.1_ASM828v1_genomic.fna
2984	Lactococcus lactis	GCA_000009425.1_ASM942v1_genomic.fna
2985	Staphylococcus epidermidis	GCA_000011925.1_ASM1192v1_genomic.fna
2986	Staphylococcus aureus	GCA_000009005.1_ASM900v1_genomic.fna
2987	Yersinia pestis	GCA_000006645.1_ASM664v1_genomic.fna
2988	Xanthomonas campestris	GCA_000012105.1_ASM1210v1_genomic.fna
2989	Pseudomonas fluorescens	GCA_000009225.1_ASM922v1_genomic.fna

2990	Salmonella enterica	GCA_000006945.2_ASM694v2_genomic.fna
2991	Salmonella enterica	GCA_000007545.1_ASM754v1_genomic.fna
2992	Staphylococcus aureus	GCA_000009585.1_ASM958v1_genomic.fna
2993	Pseudomonas fluorescens	GCA_000012445.1_ASM1244v1_genomic.fna
2994	Xanthomonas campestris	GCA_000070605.1_ASM7060v1_genomic.fna
2995	Campylobacter jejuni	GCA_000011865.1_ASM1186v1_genomic.fna
2996	Yersinia pestis	GCA_000007885.1_ASM788v1_genomic.fna
2997	Staphylococcus epidermidis	GCA_000751035.1_PM221_genomic.fna
2998	Lactococcus lactis	GCA_000014545.1_ASM1454v1_genomic.fna
2999	Listeria monocytogenes	GCA_000021185.1_ASM2118v1_genomic.fna
3000	Bacillus cereus	GCA_000011625.1_ASM1162v1_genomic.fna
3001	Clostridium perfringens	GCA_000013845.2_ASM1384v2_genomic.fna
3002	Mycobacterium tuberculosis	GCA_000016145.1_ASM1614v1_genomic.fna
3003	Mycobacterium bovis	GCA_000010685.1_ASM1068v1_genomic.fna
3004	Mycobacterium avium	GCA_000014985.1_ASM1498v1_genomic.fna
3005	Actinobacillus pleuropneumoniae	GCA_000020405.1_ASM2040v1_genomic.fna
3006	Prochlorococcus marinus	GCA_000011485.1_ASM1148v1_genomic.fna
3007	Haemophilus influenzae	GCA_000165525.1_ASM16552v1_genomic.fna
3008	Escherichia coli	GCA_000005845.2_ASM584v2_genomic.fna
3009	Chlamydophila pneumoniae	GCA_000011165.1_ASM1116v1_genomic.fna
3010	Buchnera aphidicola	GCA_000009605.1_ASM960v1_genomic.fna
3011	Helicobacter pylori	GCA_000011725.1_ASM1172v1_genomic.fna
3012	Neisseria meningitidis	GCA_000009465.1_ASM946v1_genomic.fna
3013	Xylella fastidiosa	GCA_000019325.1_ASM1932v1_genomic.fna
3014	Pseudomonas putida	GCA_000016865.1_ASM1686v1_genomic.fna
3015	Streptococcus pyogenes	GCA_000007425.1_ASM742v1_genomic.fna
3016	Streptococcus pneumoniae	GCA_000014365.1_ASM1436v1_genomic.fna
3017	Agrobacterium tumefaciens	GCA_001551895.1_ASM155189v1_genomic.fna
3018	Leptospira interrogans	GCA_000231175.1_ASM23117v1_genomic.fna
3019	Fusobacterium nucleatum	GCA_000162235.2_ASM16223v2_genomic.fna
3020	Streptococcus agalactiae	GCA_000196055.1_ASM19605v1_genomic.fna
3021	Bacillus anthracis	GCA_000008445.1_ASM844v1_genomic.fna
3022	Shigella flexneri	GCA_000013585.1_ASM1358v1_genomic.fna
3023	Bifidobacterium longum	GCA_000020425.1_ASM2042v1_genomic.fna

3024	Histophilus somni	GCA_002162175.1_ASM216217v1_genomic.fna
3025	Pseudomonas syringae	GCA_000344355.2_ASM34435v2_genomic.fna
3026	Pseudomonas aeruginosa	GCA_000017205.1_ASM1720v1_genomic.fna
3027	Vibrio vulnificus	GCA_000039765.1_ASM3976v1_genomic.fna
3028	Mycoplasma hyopneumoniae	GCA_000008405.1_ASM840v1_genomic.fna
3029	Streptococcus suis	GCA_000026725.1_ASM2672v1_genomic.fna
3030	Acinetobacter baumannii	GCA_000021145.1_ASM2114v1_genomic.fna
3031	Bacillus licheniformis	GCA_001596055.1_ASM159605v1_genomic.fna
3032	Bacteroides fragilis	GCA_000210835.1_ASM21083v1_genomic.fna
3033	Legionella pneumophila	GCA_000048665.1_ASM4866v1_genomic.fna
3034	Streptococcus thermophilus	GCA_000011845.1_ASM1184v1_genomic.fna
3035	Shewanella baltica	GCA_000017325.1_ASM1732v1_genomic.fna
3036	Ehrlichia ruminantium	GCA_000050425.1_ASM5042v1_genomic.fna
3037	Lactobacillus reuteri	GCA_000159455.2_ASM15945v2_genomic.fna
3038	Bacillus pumilus	GCA_000590455.1_ASM59045v1_genomic.fna
3039	Thermus thermophilus	GCA_000214845.1_ASM21484v1_genomic.fna
3040	Corynebacterium glutamicum	GCA_000196335.1_ASM19633v1_genomic.fna
3041	Mycoplasma genitalium	GCA_000292445.1_ASM29244v1_genomic.fna
3042	Chlamydia trachomatis	GCA_000012125.1_ASM1212v1_genomic.fna
3043	Burkholderia cenocepacia	GCA_000019505.1_ASM1950v1_genomic.fna
3044	Shigella sonnei	GCA_001518855.1_ASM151885v1_genomic.fna
3045	Burkholderia pseudomallei	GCA_000015905.1_ASM1590v1_genomic.fna
3046	Burkholderia mallei	GCA_000015605.1_ASM1560v1_genomic.fna
3047	Ehrlichia chaffeensis	GCA_000632865.1_ASM63286v1_genomic.fna
3048	Ralstonia solanacearum	GCA_000283475.1_ASM28347v1_genomic.fna
3049	Bacillus thuringiensis	GCA_000015065.1_ASM1506v1_genomic.fna
3050	Shigella boydii	GCA_001027225.1_ASM102722v1_genomic.fna
3051	Vibrio cholerae	GCA_000021605.1_ASM2160v1_genomic.fna
3052	Streptococcus salivarius	GCA_000253335.1_ASM25333v1_genomic.fna
3053	Rhodopseudomonas palustris	GCA_000014825.1_ASM1482v1_genomic.fna
3054	Rhodobacter sphaeroides	GCA_000015985.1_ASM1598v1_genomic.fna
3055	Yersinia pseudotuberculosis	GCA_000020085.1_ASM2008v1_genomic.fna
3056	Francisella tularensis	GCA_000009325.1_ASM932v1_genomic.fna
3057	Brucella abortus	GCA_000054005.1_ASM5400v1_genomic.fna

3058	Lactobacillus delbrueckii	GCA_000182835.1_ASM18283v1_genomic.fna
3059	Leptospira borgpetersenii	GCA_001444465.1_ASM144446v1_genomic.fna
3060	Mycoplasma capricolum	GCA_000835085.1_ASM83508v1_genomic.fna
3061	Bartonella bacilliformis	GCA_001559035.1_ASM155903v1_genomic.fna
3062	Xanthomonas citri	GCA_000349225.1_ASM34922v1_genomic.fna
3063	Campylobacter fetus	GCA_000015085.1_ASM1508v1_genomic.fna
3064	Streptococcus mitis	GCA_001560895.1_ASM156089v1_genomic.fna
3065	Xanthomonas oryzae	GCA_000168315.3_ASM16831v3_genomic.fna
3066	Clostridioides difficile	GCA_000210435.1_ASM21043v1_genomic.fna
3067	Aeromonas salmonicida	GCA_002214265.1_ASM221426v1_genomic.fna
3068	Coxiella burnetii	GCA_000018745.1_ASM1874v1_genomic.fna
3069	Lactobacillus casei	GCA_000194765.1_ASM19476v1_genomic.fna
3070	Bacillus subtilis	GCA_000186745.1_ASM18674v1_genomic.fna
3071	Desulfovibrio vulgaris	GCA_000015485.1_ASM1548v1_genomic.fna
3072	Rickettsia bellii	GCA_002078315.1_ASM207831v1_genomic.fna
3073	Ralstonia pickettii	GCA_002393485.1_ASM239348v1_genomic.fna
3074	Lactobacillus helveticus	GCA_000165775.3_ASM16577v3_genomic.fna
3075	Rickettsia rickettsii	GCA_000283775.1_ASM28377v1_genomic.fna
3076	Bifidobacterium adolescentis	GCA_000817995.1_ASM81799v1_genomic.fna
3077	Borrelia afzelii	GCA_000741005.1_ASM74100v1_genomic.fna
3078	Vibrio harveyi	GCA_001908435.2_ASM190843v2_genomic.fna
3079	Methylobacterium extorquens	GCA_000022685.1_ASM2268v1_genomic.fna
3080	Lactococcus garvieae	GCA_002355575.1_ASM235557v1_genomic.fna
3081	Vibrio parahaemolyticus	GCA_000430405.1_ASM43040v1_genomic.fna
3082	Lactobacillus fermentum	GCA_000397165.1_ASM39716v1_genomic.fna
3083	Clostridium kluyveri	GCA_001902295.1_ASM190229v1_genomic.fna
3084	Porphyromonas gingivalis	GCA_000270225.1_ASM27022v1_genomic.fna
3085	Burkholderia thailandensis	GCA_000567905.1_ASM56790v1_genomic.fna
3086	Mycoplasma mycoides	GCA_000023685.1_ASM2368v1_genomic.fna
3087	Phaeobacter gallaeciensis	GCA_002381285.1_ASM238128v1_genomic.fna
3088	Ureaplasma parvum	GCA_000828735.1_ASM82873v1_genomic.fna
3089	Clostridium botulinum	GCA_000063585.1_ASM6358v1_genomic.fna
3090	Burkholderia multivorans	GCA_000756005.1_ASM75600v1_genomic.fna
3091	Rickettsia prowazekii	GCA_000277165.1_ASM27716v1_genomic.fna

3092	Klebsiella pneumoniae	GCA_000016305.1_ASM1630v1_genomic.fna
3093	Borrelia burgdorferi	GCA_000166635.2_ASM16663v1_genomic.fna
3094	Brucella canis	GCA_000691585.1_ASM69158v1_genomic.fna
3095	Clavibacter michiganensis	GCA_000355695.1_ASM35569v1_genomic.fna
3096	Brucella suis	GCA_000223195.1_ASM22319v1_genomic.fna
3097	Burkholderia ambifaria	GCA_000959545.1_ASM95954v1_genomic.fna
3098	Enterococcus faecalis	GCA_000172575.2_ASM17257v2_genomic.fna
3099	Bradyrhizobium japonicum	GCA_001887695.1_ASM188769v1_genomic.fna
3100	Microcystis aeruginosa	GCA_001704955.1_ASM170495v1_genomic.fna
3101	Streptococcus dysgalactiae	GCA_000188715.1_ASM18871v1_genomic.fna
3102	Rhizobium etli	GCA_000442435.1_ASM44243v1_genomic.fna
3103	Ruminiclostridium thermocellum	GCA_000255615.3_ASM25561v3_genomic.fna
3104	Borrelia garinii	GCA_000300045.1_ASM30004v1_genomic.fna
3105	Chlamydia psittaci	GCA_000270385.1_ASM27038v1_genomic.fna
3106	Bifidobacterium animalis	GCA_000022705.1_ASM2270v1_genomic.fna
3107	Bacillus amyloliquefaciens	GCA_000204275.1_ASM20427v1_genomic.fna
3108	Streptococcus mutans	GCA_000271865.1_ASM27186v1_genomic.fna
3109	Neisseria gonorrhoeae	GCA_000156855.2_ASM15685v2_genomic.fna
3110	Comamonas testosteroni	GCA_001406795.1_Comamonas_testosteroni_P19_genomic.fna
3111	Enterococcus faecium	GCA_000336405.1_ASM33640v1_genomic.fna
3112	Bacillus mycoides	GCA_000742855.1_ASM74285v1_genomic.fna
3113	Yersinia frederiksenii	GCA_002591195.1_ASM259119v1_genomic.fna
3114	Alteromonas macleodii	GCA_000299995.1_ASM29999v1_genomic.fna
3115	Stenotrophomonas maltophilia	GCA_000223885.1_ASM22388v1_genomic.fna
3116	Bifidobacterium bifidum	GCA_000265095.1_ASM26509v1_genomic.fna
3117	Zymomonas mobilis	GCA_000007105.1_ASM710v1_genomic.fna
3118	Streptococcus equi	GCA_000026585.1_ASM2658v1_genomic.fna
3119	Haemophilus parasuis	GCA_000742795.1_ASM74279v1_genomic.fna
3120	Acetobacter pasteurianus	GCA_000010845.1_ASM1084v1_genomic.fna
3121	Francisella tularensis	GCA_001880245.1_ASM188024v1_genomic.fna
3122	Pasteurella multocida	GCA_000234745.1_ASM23474v1_genomic.fna
3123	Francisella philomiragia	GCA_000833215.1_ASM83321v1_genomic.fna
3124	Lactobacillus rhamnosus	GCA_000026525.1_ASM2652v1_genomic.fna
3125	Brucella melitensis	GCA_000192725.1_ASM19272v1_genomic.fna

3126	Bacillus megaterium	GCA_000025805.1_ASM2580v1_genomic.fna
3127	Burkholderia glumae	GCA_000960995.1_ASM96099v1_genomic.fna
3128	Providencia stuartii	GCA_001558855.1_ASM155885v1_genomic.fna
3129	Sinorhizobium meliloti	GCA_000147795.3_ASM14779v3_genomic.fna
3130	Azotobacter vinelandii	GCA_000380365.1_ASM38036v1_genomic.fna
3131	Bordetella bronchiseptica	GCA_000317955.1_ASM31795v1_genomic.fna
3132	Bordetella pertussis	GCA_000212975.1_ASM21297v1_genomic.fna
3133	Bordetella parapertussis	GCA_001704255.1_ASM170425v1_genomic.fna
3134	Haemophilus ducreyi	GCA_001647695.1_ASM164769v1_genomic.fna
3135	Streptococcus gordonii	GCA_001281105.1_ASM128110v1_genomic.fna
3136	Clostridium acetobutylicum	GCA_000218855.1_ASM21885v1_genomic.fna
3137	Lactobacillus gasseri	GCA_002287905.1_ASM228790v1_genomic.fna
3138	Corynebacterium diphtheriae	GCA_000241875.1_ASM24187v1_genomic.fna
3139	Mycobacterium smegmatis	GCA_000767605.1_ASM76760v1_genomic.fna
3140	Mycoplasma pneumoniae	GCA_000283755.1_ASM28375v1_genomic.fna
3141	Thermotoga maritima	GCA_000390265.1_ASM39026v1_genomic.fna
3142	Yersinia enterocolitica	GCA_000253175.1_ASM25317v1_genomic.fna
3143	Dehalococcoides mccartyi	GCA_000009025.1_ASM902v1_genomic.fna
3144	Chlamydia muridarum	GCA_000767405.1_ASM76740v1_genomic.fna
3145	Leuconostoc mesenteroides	GCA_000512955.1_ASM51295v1_genomic.fna
3146	Anaplasma phagocytophilum	GCA_000439775.1_ASM43977v1_genomic.fna
3147	Pectobacterium atrosepticum	GCA_000740965.1_ASM74096v1_genomic.fna
3148	Salmonella bongori	GCA_000709535.1_ASM70953v1_genomic.fna
3149	Chlamydia abortus	GCA_002205755.1_ASM220575v1_genomic.fna
3150	Lactobacillus acidophilus	GCA_000934625.1_ASM93462v1_genomic.fna
3151	Bartonella henselae	GCA_000612965.1_PRJEB4971_assembly_1_genomic.fna
3152	Mannheimia haemolytica	GCA_000349785.2_ASM34978v2_genomic.fna
3153	Leifsonia xyli	GCA_001647635.1_ASM164763v1_genomic.fna
3154	Lactobacillus plantarum	GCA_000148815.2_ASM14881v1_genomic.fna
3155	Pediococcus pentosaceus	GCA_001411765.2_ASM141176v2_genomic.fna
3156	Lactobacillus brevis	GCA_001676805.1_ASM167680v1_genomic.fna
3157	Serratia marcescens	GCA_000336425.1_ASM33642v1_genomic.fna
3158	Mycoplasma gallisepticum	GCA_000025385.1_ASM2538v1_genomic.fna
3159	Moorella thermoacetica	GCA_001267435.1_ASM126743v1_genomic.fna

3160	Mesoplasma florum	GCA_002504365.1_ASM250436v1_genomic.fna
3161	Burkholderia vietnamiensis	GCA_001718775.1_ASM171877v1_genomic.fna
3162	Propionibacterium acnes	GCA_000213155.1_ASM21315v1_genomic.fna
3163	Campylobacter coli	GCA_000494775.1_ASM49477v1_genomic.fna
3164	Campylobacter lari	GCA_000816365.1_ASM81636v1_genomic.fna
3165	Mycoplasma bovis	GCA_000270525.1_ASM27052v1_genomic.fna
3166	Klebsiella oxytoca	GCA_001022115.1_ASM102211v1_genomic.fna
3167	Proteus mirabilis	GCA_001281545.1_ASM128154v1_genomic.fna
3168	Clostridium beijerinckii	GCA_000506785.3_ASM50678v3_genomic.fna
3169	Cronobacter sakazakii	GCA_000263215.1_ASM26321v1_genomic.fna
3170	Lactobacillus salivarius	GCA_000758365.1_ASM75836v1_genomic.fna
3171	Gluconobacter oxydans	GCA_000583855.1_ASM58385v1_genomic.fna
3172	Bacillus clausii	GCA_002250115.1_ASM225011v1_genomic.fna
3173	Wolbachia endosymbiont	GCA_000953315.1_Wau001_genomic.fna
3174	Enterobacter cloacae	GCA_000239975.1_ASM23997v1_genomic.fna
3175	Moraxella catarrhalis	GCA_000766665.1_ASM76666v1_genomic.fna
3176	Listeria ivanovii	GCA_000763475.1_ASM76347v1_genomic.fna
3177	Bifidobacterium breve	GCA_000220135.1_ASM22013v1_genomic.fna
3178	Staphylococcus saprophyticus	GCA_001558275.1_ASM155827v1_genomic.fna
3179	Bacillus coagulans	GCA_000217835.1_ASM21783v1_genomic.fna
3180	Herbaspirillum seropedicae	GCA_001721865.1_ASM172186v1_genomic.fna
3181	Mycobacterium abscessus	GCA_000445035.1_ASM44503v1_genomic.fna
3182	Paenibacillus polymyxa	GCA_000237325.1_ASM23732v1_genomic.fna
3183	Legionella longbeachae	GCA_002113845.1_ASM211384v1_genomic.fna
3184	Aeromonas hydrophila	GCA_000512185.1_ASM51218v1_genomic.fna
3185	Propionibacterium freudenreichii	GCA_002117425.1_ASM211742v1_genomic.fna
3186	Pseudomonas stutzeri	GCA_000013785.1_ASM1378v1_genomic.fna
3187	Streptococcus iniae	GCA_002220115.1_ASM222011v1_genomic.fna
3188	Granulibacter bethesdensis	GCA_000576145.2_ASM57614v2_genomic.fna
3189	Pseudomonas mendocina	GCA_000733715.2_ASM73371v2_genomic.fna
3190	Geobacillus thermodenitrificans	GCA_002119625.1_ASM211962v1_genomic.fna
3191	Burkholderia ubonensis	GCA_001718655.1_ASM171865v1_genomic.fna
3192	Lysinibacillus sphaericus	GCA_001598075.1_ASM159807v1_genomic.fna
3193	Flavobacterium psychrophilum	GCA_000754365.1_ASM75436v1_genomic.fna

3194	Clostridium butyricum	GCA_001646605.1_ASM164660v1_genomic.fna
3195	Rhodococcus erythropolis	GCA_000696675.2_ASM69667v2_genomic.fna
3196	Lactobacillus johnsonii	GCA_000204985.1_ASM20498v1_genomic.fna
3197	Rickettsia typhi	GCA_000277305.1_ASM27730v1_genomic.fna
3198	Lactobacillus sakei	GCA_002250035.1_ASM225003v1_genomic.fna
3199	Paenibacillus larvae	GCA_002007765.1_ASM200776v1_genomic.fna
3200	Arcobacter butzleri	GCA_000284355.1_ASM28435v1_genomic.fna
3201	Mycobacterium intracellulare	GCA_000767485.1_ASM76748v1_genomic.fna
3202	Anoxybacillus flavithermus	GCA_002243705.1_ASM224370v1_genomic.fna
3203	Edwardsiella tarda	GCA_001011055.1_ASM101105v1_genomic.fna
3204	Oligotropha carboxidovorans	GCA_000218585.1_ASM21858v1_genomic.fna
3205	Escherichia albertii	GCA_002285455.1_ASM228545v1_genomic.fna
3206	Corynebacterium urealyticum	GCA_900187235.1_52184_D01_genomic.fna
3207	Yersinia ruckeri	GCA_002192595.1_ASM219259v1_genomic.fna
3208	Variovorax paradoxus	GCA_000023345.1_ASM2334v1_genomic.fna
3209	Rhodococcus opacus	GCA_001685605.1_ASM168560v1_genomic.fna
3210	Pectobacterium carotovorum	GCA_001932635.1_ASM193263v1_genomic.fna
3211	Rothia mucilaginosa	GCA_002386365.1_ASM238636v1_genomic.fna
3212	Streptomyces albus	GCA_000827005.1_ASM82700v1_genomic.fna
3213	Aggregatibacter actinomycetemcomitans	GCA_000163615.3_ASM16361v3_genomic.fna
3214	Thermoanaerobacterium thermosaccharolyticun	GCA_002250075.1_ASM225007v1_genomic.fna
3215	Streptococcus intermedius	GCA_000463385.1_ASM46338v1_genomic.fna
3216	Streptomyces hygroscopicus	GCA_001447075.1_ASM144707v1_genomic.fna
3217	Mycoplasma hyorhinis	GCA_000211295.1_ASM21129v1_genomic.fna
3218	Aggregatibacter aphrophilus	GCA_001262035.1_ASM126203v1_genomic.fna
3219	Gardnerella vaginalis	GCA_000213955.1_ASM21395v1_genomic.fna
3220	Capnocytophaga sputigena	GCA_002302595.1_ASM230259v1_genomic.fna
3221	Lactobacillus paracasei	GCA_000155515.2_ASM15551v2_genomic.fna
3222	Staphylococcus capitis	GCA_002591175.1_ASM259117v1_genomic.fna
3223	Oxalobacter formigenes	GCA_002128345.1_ASM212834v1_genomic.fna
3224	Erysipelothrix rhusiopathiae	GCA_001602155.1_ASM160215v1_genomic.fna
3225	Bacillus atrophaeus	GCA_000830075.1_ASM83007v1_genomic.fna
3226	Klebsiella variicola	GCA_000828055.2_ASM82805v2_genomic.fna
3227	Streptococcus oralis	GCA_002386345.1_ASM238634v1_genomic.fna

3228	Achromobacter xylosoxidans	GCA_000758265.1_ASM75826v1_genomic.fna
3229	Xanthomonas fuscans	GCA_001610815.1_ASM161081v1_genomic.fna
3230	Vibrio alginolyticus	GCA_001469735.1_ASM146973v1_genomic.fna
3231	Pediococcus acidilactici	GCA_002173575.1_ASM217357v1_genomic.fna
3232	Vibrio coralliilyticus	GCA_001693615.1_ASM169361v1_genomic.fna
3233	Parageobacillus thermoglucosidasius	GCA_001700985.1_ASM170098v1_genomic.fna
3234	Corynebacterium pseudotuberculosis	GCA_000144935.2_ASM14493v2_genomic.fna
3235	Enterobacter asburiae	GCA_001022095.1_ASM102209v1_genomic.fna
3236	Veillonella parvula	GCA_900186885.1_48903_D01_genomic.fna
3237	Mesorhizobium ciceri	GCA_001618825.1_ASM161882v1_genomic.fna
3238	Xanthomonas euvesicatoria	GCA_001908795.1_ASM190879v1_genomic.fna
3239	Acinetobacter pittii	GCA_001617545.1_ASM161754v1_genomic.fna
3240	Riemerella anatipestifer	GCA_000191565.1_ASM19156v1_genomic.fna
3241	Lactobacillus amylovorus	GCA_002706375.1_ASM270637v1_genomic.fna
3242	Streptococcus constellatus	GCA_000463445.1_ASM46344v1_genomic.fna
3243	Staphylococcus lugdunensis	GCA_001558775.1_ASM155877v1_genomic.fna
3244	Streptococcus anginosus	GCA_000478925.1_ASM47892v1_genomic.fna
3245	Pantoea ananatis	GCA_000270125.2_ASM27012v2_genomic.fna
3246	Amycolatopsis mediterranei	GCA_000282715.1_ASM28271v1_genomic.fna
3247	Streptococcus gallolyticus	GCA_000203195.1_ASM20319v1_genomic.fna
3248	Taylorella equigenitalis	GCA_002288025.1_ASM228802v1_genomic.fna
3249	Ketogulonicigenium vulgare	GCA_001399515.1_ASM139951v1_genomic.fna
3250	Citrobacter freundii	GCA_001022275.1_ASM102227v1_genomic.fna
3251	Brachyspira pilosicoli	GCA_000296575.1_ASM29657v1_genomic.fna
3252	Capnocytophaga canimorsus	GCA_002302565.1_ASM230256v1_genomic.fna
3253	Cellulophaga lytica	GCA_001941605.1_ASM194160v1_genomic.fna
3254	Sinorhizobium fredii	GCA_002288485.1_ASM228848v1_genomic.fna
3255	Vibrio anguillarum	GCA_000786425.1_Vibrio_anguillarum_NB10_serovar_O1_genomic.fna
3256	Mycoplasma hominis	GCA_000947915.1_ASM94791v1_genomic.fna
3257	Eubacterium limosum	GCA_001481725.1_ASM148172v1_genomic.fna
3258	Pseudoxanthomonas suwonensis	GCA_000972865.1_ASM97286v1_genomic.fna
3259	Enterobacter hormaechei	GCA_000807425.4_ASM80742v4_genomic.fna
3260	Weissella cibaria	GCA_001951075.1_ASM195107v1_genomic.fna
3261	Lactobacillus coryniformis	GCA_002706705.1_ASM270670v1_genomic.fna

3262	Leuconostoc gelidum	GCA_001536305.1_Leuconostoc_gelidum_subsp._gasicomitatum_KG16-1_genomic.fna
3263	Nocardiopsis dassonvillei	GCA_002260685.1_ASM226068v1_genomic.fna
3264	Klebsiella aerogenes	GCA_001021995.1_ASM102199v1_genomic.fna
3265	Staphylococcus pseudintermedius	GCA_001682335.1_ASM168233v1_genomic.fna
3266	Corynebacterium ulcerans	GCA_000306825.1_ASM30682v1_genomic.fna
3267	Chlamydia pecorum	GCA_000470805.1_ASM47080v1_genomic.fna
3268	Aeromonas veronii	GCA_001634325.1_ASM163432v1_genomic.fna
3269	Paenibacillus mucilaginosus	GCA_000258535.2_ASM25853v2_genomic.fna
3270	Burkholderia gladioli	GCA_002208175.1_ASM220817v1_genomic.fna
3271	Mycobacterium abscessus	GCA_000770125.1_ASM77012v1_genomic.fna
3272	Pseudomonas brassicacearum	GCA_001449085.1_ASM144908v1_genomic.fna
3273	Rickettsia amblyommatis	GCA_002078335.1_ASM207833v1_genomic.fna
3274	Burkholderia cepacia	GCA_000701165.2_ASM70116v2_genomic.fna
3275	Tetragenococcus halophilus	GCA_002243535.1_ASM224353v1_genomic.fna
3276	Enterococcus hirae	GCA_002278015.1_ASM227801v1_genomic.fna
3277	Hafnia alvei	GCA_001636255.1_ASM163625v1_genomic.fna
3278	Streptomyces venezuelae	GCA_001886595.1_ASM188659v1_genomic.fna
3279	Lactobacillus curvatus	GCA_001698165.1_ASM169816v1_genomic.fna
3280	Pediococcus damnosus	GCA_001611075.1_ASM161107v1_genomic.fna
3281	Agrobacterium rhizogenes	GCA_002005205.2_ASM200520v2_genomic.fna
3282	Morganella morganii	GCA_002386305.1_ASM238630v1_genomic.fna
3283	Prevotella intermedia	GCA_001548195.1_ASM154819v1_genomic.fna
3284	Tannerella forsythia	GCA_001547875.1_ASM154787v1_genomic.fna
3285	Vibrio campbellii	GCA_001969325.1_ASM196932v1_genomic.fna
3286	Staphylococcus xylosus	GCA_000953575.1_Staphylococcus_xylosus_C2a_genomic.fna
3287	Pseudomonas pseudoalcaligenes	GCA_002355475.1_ASM235547v1_genomic.fna
3288	Rickettsia japonica	GCA_002357115.1_ASM235711v1_genomic.fna
3289	Pediococcus claussenii	GCA_001702235.1_ASM170223v1_genomic.fna
3290	Moraxella bovoculi	GCA_000988655.1_ASM98865v1_genomic.fna
3291	Campylobacter sputorum	GCA_002220775.1_ASM222077v1_genomic.fna
3292	Enterococcus durans	GCA_002277935.1_ASM227793v1_genomic.fna
3293	Flavobacterium columnare	GCA_001641185.1_ASM164118v1_genomic.fna
3294	Francisella noatunensis	GCA_001042525.2_ASM104252v2_genomic.fna
3295	Piscirickettsia salmonis	GCA_000756435.3_ASM75643v3_genomic.fna

3296	Elizabethkingia anophelis	GCA_001011675.1_ASM101167v1_genomic.fna
3297	Pseudomonas monteilii	GCA_000510325.1_ASM51032v1_genomic.fna
3298	Rhizobium phaseoli	GCA_001664125.1_ASM166412v1_genomic.fna
3299	Pseudoalteromonas luteoviolacea	GCA_001750405.1_ASM175040v1_genomic.fna
3300	Xanthomonas fragariae	GCA_900183975.1_PD885.1_genomic.fna
3301	Geobacillus thermoleovorans	GCA_001719205.1_ASM171920v1_genomic.fna
3302	Pseudomonas chlororaphis	GCA_000963835.1_ASM96383v1_genomic.fna
3303	Serratia plymuthica	GCA_000261045.2_ASM26104v2_genomic.fna
3304	Alcaligenes faecalis	GCA_002242175.1_ASM224217v1_genomic.fna
3305	Phaeobacter inhibens	GCA_001969345.1_ASM196934v1_genomic.fna
3306	Alteromonas stellipolaris	GCA_001632765.1_ASM163276v1_genomic.fna
3307	Staphylococcus equorum	GCA_001683315.1_ASM168331v1_genomic.fna
3308	Paraburkholderia caribensis	GCA_001611015.1_ASM161101v1_genomic.fna
3309	Acidipropionibacterium acidipropionici	GCA_001602115.1_ASM160211v1_genomic.fna
3310	Leptolyngbya boryana	GCA_002368255.1_ASM236825v1_genomic.fna
3311	Bordetella holmesii	GCA_000341485.2_ASM34148v2_genomic.fna
3312	Bacillus safensis	GCA_001938685.1_ASM193868v1_genomic.fna
3313	Rhodovulum sulfidophilum	GCA_001548075.1_ASM154807v1_genomic.fna
3314	Thalassolituus oleivorans	GCA_001932535.1_ASM193253v1_genomic.fna
3315	Cedecea neteri	GCA_000758325.1_ASM75832v1_genomic.fna
3316	Chryseobacterium indologenes	GCA_002208925.1_ASM220892v1_genomic.fna
3317	Sphingopyxis macrogoltabida	GCA_001610995.1_ASM161099v1_genomic.fna
3318	Vibrio natriegens	GCA_001680045.1_ASM168004v1_genomic.fna
3319	Pseudomonas protegens	GCA_002006545.1_ASM200654v1_genomic.fna
3320	Clostridium stercorarium	GCA_001697245.1_ASM169724v1_genomic.fna
3321	Citrobacter amalonaticus	GCA_001559075.1_ASM155907v1_genomic.fna
3322	Bibersteinia trehalosi	GCA_000521745.1_ASM52174v1_genomic.fna
3323	Serratia liquefaciens	GCA_001559135.1_ASM155913v1_genomic.fna
3324	Raoultella ornithinolytica	GCA_001455225.1_ASM145522v1_genomic.fna
3325	Brochothrix thermosphacta	GCA_002442515.1_ASM244251v1_genomic.fna
3326	Corynebacterium stationis	GCA_002024345.1_ASM202434v1_genomic.fna
3327	Weissella ceti	GCA_000750515.1_ASM75051v1_genomic.fna
3328	Mannheimia varigena	GCA_000521695.1_ASM52169v1_genomic.fna
3329	Trueperella pyogenes	GCA_000816065.1_ASM81606v1_genomic.fna

3330	Sporosarcina ureae	GCA_002101375.1_ASM210137v1_genomic.fna
3331	Mycobacterium yongonense	GCA_002148195.1_ASM214819v1_genomic.fna
3332	Bordetella hinzii	GCA_900186895.1_48450_A02_genomic.fna
3333	Clostridium saccharobutylicum	GCA_002003325.1_ASM200332v1_genomic.fna
3334	Bradyrhizobium diazoefficiens	GCA_001642675.1_ASM164267v1_genomic.fna
3335	Clostridium chauvoei	GCA_002327205.1_ASM232720v1_genomic.fna
3336	Streptomyces lydicus	GCA_001984445.1_ASM198444v1_genomic.fna
3337	Pandoraea pnomenusa	GCA_000590495.2_ASM59049v2_genomic.fna
3338	Bacillus velezensis	GCA_000284395.1_ASM28439v1_genomic.fna
3339	Klebsiella michiganensis	GCA_000632415.1_ASM63241v1_genomic.fna
3340	Acidihalobacter prosperus	GCA_001753245.1_ASM175324v1_genomic.fna
3341	Collimonas arenae	GCA_001584205.1_ASM158420v1_genomic.fna
3342	Fusobacterium hwasookii	GCA_001455145.1_ASM145514v1_genomic.fna
3343	Pseudomonas frederiksbergensis	GCA_002355315.1_ASM235531v1_genomic.fna
3344	Pandoraea apista	GCA_001027265.1_ASM102726v1_genomic.fna
3345	Campylobacter iguaniorum	GCA_001643995.1_ASM164399v1_genomic.fna
3346	Tatlockia micdadei	GCA_002085735.1_ASM208573v1_genomic.fna
3347	Luteibacter rhizovicinus	GCA_001887595.1_ASM188759v1_genomic.fna
3348	Klebsiella quasipneumoniae	GCA_002240315.1_ASM224031v1_genomic.fna
3349	Staphylococcus schleiferi	GCA_001188895.1_ASM118889v1_genomic.fna
3350	Oblitimonas alkaliphila	GCA_001267215.1_ASM126721v1_genomic.fna
3351	Mycobacterium chimaera	GCA_002219245.1_ASM221924v1_genomic.fna
3352	Lactobacillus paracollinoides	GCA_001702195.1_ASM170219v1_genomic.fna
3353	Methylomonas koyamae	GCA_002441955.1_ASM244195v1_genomic.fna
3354	Blastochloris viridis	GCA_001548155.2_BV133_assembly_1.0_genomic.fna
3355	Bacillus paralicheniformis	GCA_002068155.1_ASM206815v1_genomic.fna
3356	Alteromonas mediterranea	GCA_000439495.1_ASM43949v1_genomic.fna
3357	Enterobacter cloacae	GCA_001729745.1_ASM172974v1_genomic.fna
3358	Polynucleobacter asymbioticus	GCA_001874565.1_ASM187456v1_genomic.fna
3359	Lactobacillus backii	GCA_001663715.1_ASM166371v1_genomic.fna
3360	Bartonella apis	GCA_002007505.1_ASM200750v1_genomic.fna
3361	Campylobacter pinnipediorum	GCA_002021965.1_ASM202196v1_genomic.fna
3362	Phaeobacter piscinae	GCA_002412045.1_ASM241204v1_genomic.fna
3363	Campylobacter pinnipediorum	GCA_002021985.1_ASM202198v1_genomic.fna

3364	Lactobacillus backii	GCA_001663735.1_ASM166373v1_genomic.fna
3365	Polynucleobacter asymbioticus	GCA_001874585.1_ASM187458v1_genomic.fna
3366	Alteromonas mediterranea	GCA_000439535.1_ASM43953v1_genomic.fna
3367	Bacillus paralicheniformis	GCA_002393225.1_ASM239322v1_genomic.fna
3368	Mycobacterium chimaera	GCA_002219265.1_ASM221926v1_genomic.fna
3369	Oblitimonas alkaliphila	GCA_001267255.1_ASM126725v1_genomic.fna
3370	Staphylococcus schleiferi	GCA_001188915.1_ASM118891v1_genomic.fna
3371	Klebsiella quasipneumoniae	GCA_002240335.1_ASM224033v1_genomic.fna
3372	Pandoraea apista	GCA_001465595.2_ASM146559v2_genomic.fna
3373	Klebsiella michiganensis	GCA_000724525.1_ASM72452v1_genomic.fna
3374	Bacillus velezensis	GCA_000319475.1_ASM31947v1_genomic.fna
3375	Pandoraea pnomenusa	GCA_000604065.3_ASM60406v3_genomic.fna
3376	Bradyrhizobium diazoefficiens	GCA_001908315.1_ASM190831v1_genomic.fna
3377	Clostridium saccharobutylicum	GCA_002003365.1_ASM200336v1_genomic.fna
3378	Sporosarcina ureae	GCA_002109325.1_ASM210932v1_genomic.fna
3379	Trueperella pyogenes	GCA_001281085.1_ASM128108v1_genomic.fna
3380	Raoultella ornithinolytica	GCA_001700855.1_ASM170085v1_genomic.fna
3381	Bibersteinia trehalosi	GCA_000521765.1_ASM52176v1_genomic.fna
3382	Clostridium stercorarium	GCA_001697285.1_ASM169728v1_genomic.fna
3383	Pseudomonas protegens	GCA_002208745.1_ASM220874v1_genomic.fna
3384	Vibrio natriegens	GCA_001680065.1_ASM168006v1_genomic.fna
3385	Cedecea neteri	GCA_002393445.1_ASM239344v1_genomic.fna
3386	Rhodovulum sulfidophilum	GCA_001633145.1_ASM163314v1_genomic.fna
3387	Enterobacter cloacae	GCA_000807415.4_ASM80741v4_genomic.fna
3388	Bacillus safensis	GCA_001938705.1_ASM193870v1_genomic.fna
3389	Bordetella holmesii	GCA_000765395.1_ASM76539v1_genomic.fna
3390	Acidipropionibacterium acidipropionici	GCA_001855335.1_ASM185533v1_genomic.fna
3391	Alteromonas stellipolaris	GCA_001632825.1_ASM163282v1_genomic.fna
3392	Alcaligenes faecalis	GCA_002443155.1_ASM244315v1_genomic.fna
3393	Serratia plymuthica	GCA_000438825.1_ASM43882v1_genomic.fna
3394	Pseudomonas chlororaphis	GCA_001023535.1_ASM102353v1_genomic.fna
3395	Xanthomonas fragariae	GCA_900183985.1_NBC2815.1_genomic.fna
3396	Rhizobium phaseoli	GCA_001664165.1_ASM166416v1_genomic.fna
3397	Elizabethkingia anophelis	GCA_001596175.2_ASM159617v2_genomic.fna

3398	Francisella noatunensis	GCA_001042545.2_ASM104254v2_genomic.fna
3399	Piscirickettsia salmonis	GCA_001514395.1_ASM151439v1_genomic.fna
3400	Flavobacterium columnare	GCA_001677395.1_ASM167739v1_genomic.fna
3401	Campylobacter sputorum	GCA_002220795.1_ASM222079v1_genomic.fna
3402	Moraxella bovoculi	GCA_000988685.1_ASM98868v1_genomic.fna
3403	Rickettsia japonica	GCA_002357135.1_ASM235713v1_genomic.fna
3404	Staphylococcus xylosus	GCA_002078255.1_ASM207825v1_genomic.fna
3405	Vibrio campbellii	GCA_002140055.1_ASM214005v1_genomic.fna
3406	Clostridium pasteurianum	GCA_000807175.1_ASM80717v1_genomic.fna
3407	Prevotella intermedia	GCA_001953935.1_ASM195393v1_genomic.fna
3408	Pediococcus damnosus	GCA_001611115.1_ASM161111v1_genomic.fna
3409	Lactobacillus curvatus	GCA_001723545.1_ASM172354v1_genomic.fna
3410	Burkholderia cepacia	GCA_000755805.1_ASM75580v1_genomic.fna
3411	Pseudomonas brassicacearum	GCA_001746815.1_ASM174681v1_genomic.fna
3412	Burkholderia gladioli	GCA_002393425.1_ASM239342v1_genomic.fna
3413	Chlamydia pecorum	GCA_000470825.1_ASM47082v1_genomic.fna
3414	Corynebacterium ulcerans	GCA_000499805.2_ASM49980v2_genomic.fna
3415	Staphylococcus pseudintermedius	GCA_001682435.2_ASM168243v2_genomic.fna
3416	Klebsiella aerogenes	GCA_001559215.1_ASM155921v1_genomic.fna
3417	Weissella cibaria	GCA_001951095.1_ASM195109v1_genomic.fna
3418	Enterobacter hormaechei	GCA_000814125.3_ASM81412v3_genomic.fna
3419	Mycoplasma hominis	GCA_001017595.1_ASM101759v1_genomic.fna
3420	Vibrio anguillarum	GCA_001660505.1_ASM166050v1_genomic.fna
3421	Capnocytophaga canimorsus	GCA_002302655.1_ASM230265v1_genomic.fna
3422	Citrobacter freundii	GCA_001281005.1_ASM128100v1_genomic.fna
3423	Ketogulonicigenium vulgare	GCA_001693655.1_ASM169365v1_genomic.fna
3424	Taylorella equigenitalis	GCA_002288125.1_ASM228812v1_genomic.fna
3425	Streptococcus gallolyticus	GCA_000270145.1_ASM27014v1_genomic.fna
3426	Amycolatopsis mediterranei	GCA_000454025.1_ASM45402v1_genomic.fna
3427	Pantoea ananatis	GCA_000283875.1_ASM28387v1_genomic.fna
3428	Streptococcus anginosus	GCA_000831165.1_ASM83116v1_genomic.fna
3429	Staphylococcus lugdunensis	GCA_001558815.1_ASM155881v1_genomic.fna
3430	Riemerella anatipestifer	GCA_000295655.1_ASM29565v1_genomic.fna
3431	Acinetobacter pittii	GCA_002012285.1_ASM201228v1_genomic.fna

3432	Mesorhizobium ciceri	GCA_001618845.1_ASM161884v1_genomic.fna
3433	Enterobacter asburiae	GCA_001521715.1_ASM152171v1_genomic.fna
3434	Corynebacterium pseudotuberculosis	GCA_000152065.2_ASM15206v2_genomic.fna
3435	Parageobacillus thermoglucosidasius	GCA_001902495.1_ASM190249v1_genomic.fna
3436	Vibrio coralliilyticus	GCA_002073995.1_ASM207399v1_genomic.fna
3437	Pediococcus acidilactici	GCA_002173595.1_ASM217359v1_genomic.fna
3438	Vibrio alginolyticus	GCA_001679745.1_ASM167974v1_genomic.fna
3439	Xanthomonas fuscans	GCA_001610915.1_ASM161091v1_genomic.fna
3440	Achromobacter xylosoxidans	GCA_001051055.1_ASM105105v1_genomic.fna
3441	Bacillus atrophaeus	GCA_002243495.1_ASM224349v1_genomic.fna
3442	Klebsiella variicola	GCA_001278905.1_ASM127890v1_genomic.fna
3443	Erysipelothrix rhusiopathiae	GCA_001723625.1_ASM172362v1_genomic.fna
3444	Lactobacillus paracasei	GCA_000582665.1_ASM58266v1_genomic.fna
3445	Gardnerella vaginalis	GCA_001042655.1_ASM104265v1_genomic.fna
3446	Mycoplasma hyorhinis	GCA_000241125.1_ASM24112v1_genomic.fna
3447	Streptomyces hygroscopicus	GCA_002021875.1_ASM202187v1_genomic.fna
3448	Streptococcus intermedius	GCA_002073355.1_ASM207335v1_genomic.fna
3449	Serratia symbiotica	GCA_000238975.1_ASM23897v1_genomic.fna
3450	Aggregatibacter actinomycetemcomitans	GCA_000604045.1_ASM60404v1_genomic.fna
3451	Streptomyces albus	GCA_000359525.1_ASM35952v1_genomic.fna
3452	Pectobacterium carotovorum	GCA_002068115.1_ASM206811v1_genomic.fna
3453	Variovorax paradoxus	GCA_000463015.1_ASM46301v1_genomic.fna
3454	Yersinia ruckeri	GCA_002442495.1_ASM244249v1_genomic.fna
3455	Escherichia albertii	GCA_002285475.1_ASM228547v1_genomic.fna
3456	Edwardsiella tarda	GCA_002504285.1_ASM250428v1_genomic.fna
3457	Mycobacterium intracellulare	GCA_002285675.1_ASM228567v1_genomic.fna
3458	Arcobacter butzleri	GCA_900187115.1_51699_F01_genomic.fna
3459	Paenibacillus larvae	GCA_002043025.1_ASM204302v1_genomic.fna
3460	Lactobacillus sakei	GCA_002370355.1_ASM237035v1_genomic.fna
3461	Lactobacillus johnsonii	GCA_000498675.1_ASM49867v1_genomic.fna
3462	Rhodococcus erythropolis	GCA_000975175.1_ASM97517v1_genomic.fna
3463	Clostridium butyricum	GCA_001746555.1_ASM174655v1_genomic.fna
3464	Flavobacterium psychrophilum	GCA_000754405.1_ASM75440v1_genomic.fna
3465	Lysinibacillus sphaericus	GCA_001629735.1_ASM162973v1_genomic.fna

3466	Burkholderia ubonensis	GCA_001718695.1_ASM171869v1_genomic.fna
3467	Pseudomonas mendocina	GCA_002442555.1_ASM244255v1_genomic.fna
3468	Granulibacter bethesdensis	GCA_000576185.2_ASM57618v2_genomic.fna
3469	Propionibacterium freudenreichii	GCA_900016155.1_PFREUDJS1_genomic.fna
3470	Aeromonas hydrophila	GCA_000612075.2_ASM61207v2_genomic.fna
3471	Pseudomonas stutzeri	GCA_000195105.1_ASM19510v1_genomic.fna
3472	Paenibacillus polymyxa	GCA_000507205.2_ASM50720v2_genomic.fna
3473	Mycobacterium abscessus	GCA_000497265.2_ASM49726v2_genomic.fna
3474	Bacillus coagulans	GCA_000876545.1_ASM87654v1_genomic.fna
3475	Staphylococcus saprophyticus	GCA_001558375.1_ASM155837v1_genomic.fna
3476	Bifidobacterium breve	GCA_000568955.1_ASM56895v1_genomic.fna
3477	Listeria ivanovii	GCA_000763495.1_ASM76349v1_genomic.fna
3478	Moraxella catarrhalis	GCA_002073215.1_ASM207321v1_genomic.fna
3479	Enterobacter cloacae	GCA_000286275.1_ASM28627v1_genomic.fna
3480	Gluconobacter oxydans	GCA_001680825.1_ASM168082v1_genomic.fna
3481	Lactobacillus salivarius	GCA_001011095.1_ASM101109v1_genomic.fna
3482	Cronobacter sakazakii	GCA_000339015.1_ASM33901v1_genomic.fna
3483	Clostridium beijerinckii	GCA_000767745.1_ASM76774v1_genomic.fna
3484	Proteus mirabilis	GCA_001640985.1_ASM164098v1_genomic.fna
3485	Klebsiella oxytoca	GCA_001022295.1_ASM102229v1_genomic.fna
3486	Campylobacter lari	GCA_000816385.2_ASM81638v2_genomic.fna
3487	Mycoplasma bovis	GCA_000696015.1_ASM69601v1_genomic.fna
3488	Campylobacter coli	GCA_000583755.1_ASM58375v1_genomic.fna
3489	Propionibacterium acnes	GCA_000217615.1_ASM21761v1_genomic.fna
3490	Burkholderia vietnamiensis	GCA_001718815.1_ASM171881v1_genomic.fna
3491	Mesoplasma florum	GCA_002504385.1_ASM250438v1_genomic.fna
3492	Moorella thermoacetica	GCA_001729945.1_ASM172994v1_genomic.fna
3493	Mycoplasma gallisepticum	GCA_000211545.6_ASM21154v6_genomic.fna
3494	Serratia marcescens	GCA_000828775.1_ASM82877v1_genomic.fna
3495	Lactobacillus brevis	GCA_002117225.1_ASM211722v1_genomic.fna
3496	Pediococcus pentosaceus	GCA_002173535.1_ASM217353v1_genomic.fna
3497	Mannheimia haemolytica	GCA_000422095.1_ASM42209v1_genomic.fna
3498	Bartonella henselae	GCA_001291465.1_Bartonella_henselae_MVT02_genomic.fna
3499	Lactobacillus acidophilus	GCA_002224305.1_ASM222430v1_genomic.fna

3500	Salmonella bongori	GCA_002211925.1_ASM221192v1_genomic.fna
3501	Chlamydia abortus	GCA_900002505.1_CA1H_genomic.fna
3502	Anaplasma phagocytophilum	GCA_000689635.2_ASM68963v2_genomic.fna
3503	Lactobacillus plantarum	GCA_000338115.2_ASM33811v2_genomic.fna
3504	Leifsonia xyli	GCA_000007665.1_ASM766v1_genomic.fna
3505	Leuconostoc mesenteroides	GCA_001047695.1_ASM104769v1_genomic.fna
3506	Chlamydia muridarum	GCA_000767445.1_ASM76744v1_genomic.fna
3507	Dehalococcoides mccartyi	GCA_000016705.1_ASM1670v1_genomic.fna
3508	Yersinia enterocolitica	GCA_000834195.1_ASM83419v1_genomic.fna
3509	Thermotoga maritima	GCA_000978535.1_ASM97853v1_genomic.fna
3510	Mycoplasma pneumoniae	GCA_000319655.2_ASM31965v2_genomic.fna
3511	Mycobacterium smegmatis	GCA_000767665.1_ASM76766v1_genomic.fna
3512	Corynebacterium diphtheriae	GCA_000241895.1_ASM24189v1_genomic.fna
3513	Streptococcus gordonii	GCA_002073435.1_ASM207343v1_genomic.fna
3514	Haemophilus ducreyi	GCA_001647735.1_ASM164773v1_genomic.fna
3515	Bordetella pertussis	GCA_000306945.1_ASM30694v1_genomic.fna
3516	Bordetella bronchiseptica	GCA_000829175.1_ASM82917v1_genomic.fna
3517	Sinorhizobium meliloti	GCA_000218265.1_ASM21826v1_genomic.fna
3518	Providencia stuartii	GCA_001888205.1_ASM188820v1_genomic.fna
3519	Bacillus megaterium	GCA_000025825.1_ASM2582v1_genomic.fna
3520	Brucella melitensis	GCA_000192885.1_ASM19288v1_genomic.fna
3521	Caulobacter vibrioides	GCA_002310295.1_ASM231029v1_genomic.fna
3522	Francisella philomiragia	GCA_000833255.1_ASM83325v1_genomic.fna
3523	Lactobacillus rhamnosus	GCA_000233755.1_ASM23375v1_genomic.fna
3524	Pasteurella multocida	GCA_000255915.1_ASM25591v1_genomic.fna
3525	Acetobacter pasteurianus	GCA_000010865.1_ASM1086v1_genomic.fna
3526	Haemophilus parasuis	GCA_001611775.1_ASM161177v1_genomic.fna
3527	Zymomonas mobilis	GCA_000024245.1_ASM2424v1_genomic.fna
3528	Streptococcus equi	GCA_000219765.1_ASM21976v1_genomic.fna
3529	Bifidobacterium bifidum	GCA_001025135.1_ASM102513v1_genomic.fna
3530	Stenotrophomonas maltophilia	GCA_000284595.1_ASM28459v1_genomic.fna
3531	Alteromonas macleodii	GCA_000300175.1_ASM30017v1_genomic.fna
3532	Bacillus mycoides	GCA_000832885.1_ASM83288v1_genomic.fna
3533	Enterococcus faecium	GCA_000444405.1_ASM44440v1_genomic.fna

3534	Streptococcus mutans	GCA_000284575.1_ASM28457v1_genomic.fna
3535	Neisseria gonorrhoeae	GCA_001047225.1_ASM104722v1_genomic.fna
3536	Bacillus amyloliquefaciens	GCA_000221645.1_ASM22164v1_genomic.fna
3537	Chlamydia psittaci	GCA_000270405.1_ASM27040v1_genomic.fna
3538	Bifidobacterium animalis	GCA_000025245.1_ASM2524v1_genomic.fna
3539	Borrelia garinii	GCA_000691545.1_ASM69154v1_genomic.fna
3540	Ruminiclostridium thermocellum	GCA_001692755.1_ASM169275v1_genomic.fna
3541	Rhizobium etli	GCA_000816125.1_ASM81612v1_genomic.fna
3542	Streptococcus dysgalactiae	GCA_000307185.1_ASM30718v1_genomic.fna
3543	Microcystis aeruginosa	GCA_002095975.1_ASM209597v1_genomic.fna
3544	Enterococcus faecalis	GCA_000211255.2_ASM21125v2_genomic.fna
3545	Brucella suis	GCA_000236255.1_ASM23625v1_genomic.fna
3546	Rhizobium leguminosarum	GCA_000009265.1_ASM926v1_genomic.fna
3547	Brucella canis	GCA_000740335.1_ASM74033v1_genomic.fna
3548	Clavibacter michiganensis	GCA_000958465.1_ASM95846v1_genomic.fna
3549	Borrelia burgdorferi	GCA_000166655.2_ASM16665v1_genomic.fna
3550	Klebsiella pneumoniae	GCA_000019565.1_ASM1956v1_genomic.fna
3551	Rickettsia prowazekii	GCA_000277185.1_ASM27718v1_genomic.fna
3552	Burkholderia multivorans	GCA_000959525.1_ASM95952v1_genomic.fna
3553	Clostridium botulinum	GCA_000204565.1_ASM20456v1_genomic.fna
3554	Phaeobacter gallaeciensis	GCA_002381305.1_ASM238130v1_genomic.fna
3555	Burkholderia thailandensis	GCA_000567925.1_ASM56792v1_genomic.fna
3556	Mycoplasma mycoides	GCA_000143865.1_ASM14386v1_genomic.fna
3557	Ureaplasma parvum	GCA_002205515.1_ASM220551v1_genomic.fna
3558	Porphyromonas gingivalis	GCA_001263815.1_ASM126381v1_genomic.fna
3559	Lactobacillus fermentum	GCA_000466785.3_ASM46678v3_genomic.fna
3560	Vibrio parahaemolyticus	GCA_000430425.1_ASM43042v1_genomic.fna
3561	Methylobacterium extorquens	GCA_000083545.1_ASM8354v1_genomic.fna
3562	Borrelia afzelii	GCA_000962775.1_ASM96277v1_genomic.fna
3563	Rickettsia rickettsii	GCA_000283795.1_ASM28379v1_genomic.fna
3564	Lactobacillus helveticus	GCA_000189515.1_ASM18951v1_genomic.fna
3565	Bacillus subtilis	GCA_000209795.2_ASM20979v2_genomic.fna
3566	Desulfovibrio vulgaris	GCA_000166115.1_ASM16611v1_genomic.fna
3567	Lactobacillus casei	GCA_000194785.1_ASM19478v1_genomic.fna

3568	Coxiella burnetii	GCA_000019865.1_ASM1986v1_genomic.fna
3569	Clostridioides difficile	GCA_000932055.2_ASM93205v2_genomic.fna
3570	Aeromonas salmonicida	GCA_002214305.1_ASM221430v1_genomic.fna
3571	Xanthomonas oryzae	GCA_000940825.1_ASM94082v1_genomic.fna
3572	Campylobacter fetus	GCA_000512745.2_ASM51274v2_genomic.fna
3573	Xanthomonas citri	GCA_000816885.1_ASM81688v1_genomic.fna
3574	Mycoplasma capricolum	GCA_000952915.1_9231-Abomsa_assembly_V8_genomic.fna
3575	Lactobacillus delbrueckii	GCA_000191165.1_ASM19116v1_genomic.fna
3576	Brucella abortus	GCA_000238175.1_ASM23817v1_genomic.fna
3577	Francisella tularensis	GCA_000011405.1_ASM1140v1_genomic.fna
3578	Yersinia pseudotuberculosis	GCA_000047365.1_ASM4736v1_genomic.fna
3579	Rhodobacter sphaeroides	GCA_000021005.1_ASM2100v1_genomic.fna
3580	Streptococcus salivarius	GCA_000305335.2_ASM30533v2_genomic.fna
3581	Vibrio cholerae	GCA_000021625.1_ASM2162v1_genomic.fna
3582	Ralstonia solanacearum	GCA_001299555.1_ASM129955v1_genomic.fna
3583	Bacillus thuringiensis	GCA_000092165.1_ASM9216v1_genomic.fna
3584	Ehrlichia chaffeensis	GCA_000632885.1_ASM63288v1_genomic.fna
3585	Burkholderia mallei	GCA_000015625.1_ASM1562v1_genomic.fna
3586	Burkholderia pseudomallei	GCA_000015925.1_ASM1592v1_genomic.fna
3587	Shigella sonnei	GCA_001558295.1_ASM155829v1_genomic.fna
3588	Chlamydia trachomatis	GCA_000026905.1_ASM2690v1_genomic.fna
3589	Mycoplasma genitalium	GCA_000292485.1_ASM29248v1_genomic.fna
3590	Burkholderia cenocepacia	GCA_000203955.1_ASM20395v1_genomic.fna
3591	Corynebacterium glutamicum	GCA_000382905.1_ASM38290v1_genomic.fna
3592	Thermus thermophilus	GCA_000258245.1_ASM25824v1_genomic.fna
3593	Bacillus pumilus	GCA_000972685.1_ASM97268v1_genomic.fna
3594	Lactobacillus reuteri	GCA_000236455.2_ASM23645v2_genomic.fna
3595	Shewanella baltica	GCA_000018765.1_ASM1876v1_genomic.fna
3596	Streptococcus thermophilus	GCA_000014485.1_ASM1448v1_genomic.fna
3597	Legionella pneumophila	GCA_000092545.1_ASM9254v1_genomic.fna
3598	Bacteroides fragilis	GCA_000965785.1_ASM96578v1_genomic.fna
3599	Bacillus licheniformis	GCA_001726125.1_ASM172612v1_genomic.fna
3600	Acinetobacter baumannii	GCA_000021245.2_ASM2124v2_genomic.fna
3601	Streptococcus suis	GCA_000091905.1_ASM9190v1_genomic.fna

3602	Mycoplasma hyopneumoniae	GCA_000183185.1_ASM18318v1_genomic.fna
3603	Vibrio vulnificus	GCA_000186585.1_ASM18658v1_genomic.fna
3604	Pseudomonas aeruginosa	GCA_000026645.1_ASM2664v1_genomic.fna
3605	Histophilus somni	GCA_002162195.1_ASM216219v1_genomic.fna
3606	Pseudomonas syringae	GCA_000452705.3_ASM45270v3_genomic.fna
3607	Bifidobacterium longum	GCA_000092325.1_ASM9232v1_genomic.fna
3608	Shigella flexneri	GCA_000022245.1_ASM2224v1_genomic.fna
3609	Bacillus anthracis	GCA_000021445.1_ASM2144v1_genomic.fna
3610	Streptococcus agalactiae	GCA_000299135.1_ASM29913v1_genomic.fna
3611	Fusobacterium nucleatum	GCA_000163915.2_ASM16391v2_genomic.fna
3612	Leptospira interrogans	GCA_000941035.1_ASM94103v1_genomic.fna
3613	Streptococcus pneumoniae	GCA_000018965.1_ASM1896v1_genomic.fna
3614	Streptococcus pyogenes	GCA_000009385.1_ASM938v1_genomic.fna
3615	Pseudomonas putida	GCA_000019125.1_ASM1912v1_genomic.fna
3616	Xylella fastidiosa	GCA_000019765.1_ASM1976v1_genomic.fna
3617	Neisseria meningitidis	GCA_000014105.1_ASM1410v1_genomic.fna
3618	Chlamydophila pneumoniae	GCA_000024145.1_ASM2414v1_genomic.fna
3619	Buchnera aphidicola	GCA_000521565.1_ASM52156v1_genomic.fna
3620	Helicobacter pylori	GCA_000013245.1_ASM1324v1_genomic.fna
3621	Escherichia coli	GCA_000183345.1_ASM18334v1_genomic.fna
3622	Haemophilus influenzae	GCA_000165575.1_ASM16557v1_genomic.fna
3623	Prochlorococcus marinus	GCA_000012465.1_ASM1246v1_genomic.fna
3624	Actinobacillus pleuropneumoniae	GCA_001460855.1_MIDG2331_genomic.fna
3625	Mycobacterium avium	GCA_000390085.1_ASM39008v1_genomic.fna
3626	Mycobacterium bovis	GCA_000194075.3_ASM19407v3_genomic.fna
3627	Mycobacterium tuberculosis	GCA_000016925.1_ASM1692v1_genomic.fna
3628	Clostridium perfringens	GCA_001304735.1_ASM130473v1_genomic.fna
3629	Listeria monocytogenes	GCA_000022925.1_ASM2292v1_genomic.fna
3630	Bacillus cereus	GCA_000013065.1_ASM1306v1_genomic.fna
3631	Staphylococcus epidermidis	GCA_000759555.1_ASM75955v1_genomic.fna
3632	Lactococcus lactis	GCA_000025045.1_ASM2504v1_genomic.fna
3633	Staphylococcus aureus	GCA_000009645.1_ASM964v1_genomic.fna
3634	Yersinia pestis	GCA_000013805.1_ASM1380v1_genomic.fna
3635	Campylobacter jejuni	GCA_000015525.1_ASM1552v1_genomic.fna

3636	Xanthomonas campestris	GCA_000221965.1_ASM22196v1_genomic.fna
3637	Pseudomonas fluorescens	GCA_000262325.2_ASM26232v2_genomic.fna
3638	Salmonella enterica	GCA_000008105.1_ASM810v1_genomic.fna
3639	Salmonella enterica	GCA_000009505.1_ASM950v1_genomic.fna
3640	Pseudomonas fluorescens	GCA_000293885.3_ASM29388v3_genomic.fna
3641	Xanthomonas campestris	GCA_000972745.1_ASM97274v1_genomic.fna
3642	Campylobacter jejuni	GCA_000017485.1_ASM1748v1_genomic.fna
3643	Yersinia pestis	GCA_000013825.1_ASM1382v1_genomic.fna
3644	Staphylococcus aureus	GCA_000009665.1_ASM966v1_genomic.fna
3645	Lactococcus lactis	GCA_000143205.1_ASM14320v1_genomic.fna
3646	Staphylococcus epidermidis	GCA_001956655.2_ASM195665v2_genomic.fna
3647	Bacillus cereus	GCA_000021205.1_ASM2120v1_genomic.fna
3648	Clostridium perfringens	GCA_001579765.1_ASM157976v1_genomic.fna
3649	Listeria monocytogenes	GCA_000026705.1_ASM2670v1_genomic.fna
3650	Mycobacterium tuberculosis	GCA_000023625.1_ASM2362v1_genomic.fna
3651	Mycobacterium bovis	GCA_000234725.1_ASM23472v1_genomic.fna
3652	Mycobacterium avium	GCA_000758285.1_ASM75828v1_genomic.fna
3653	Prochlorococcus marinus	GCA_000012645.1_ASM1264v1_genomic.fna
3654	Haemophilus influenzae	GCA_000197875.1_ASM19787v1_genomic.fna
3655	Escherichia coli	GCA_000299455.1_ASM29945v1_genomic.fna
3656	Helicobacter pylori	GCA_000020245.1_ASM2024v1_genomic.fna
3657	Buchnera aphidicola	GCA_000090965.1_ASM9096v1_genomic.fna
3658	Chlamydophila pneumoniae	GCA_000091085.2_ASM9108v2_genomic.fna
3659	Neisseria meningitidis	GCA_000026965.1_ASM2696v1_genomic.fna
3660	Xylella fastidiosa	GCA_000148405.1_ASM14840v1_genomic.fna
3661	Pseudomonas putida	GCA_000019445.1_ASM1944v1_genomic.fna
3662	Streptococcus pyogenes	GCA_000011285.1_ASM1128v1_genomic.fna
3663	Streptococcus pneumoniae	GCA_000018985.1_ASM1898v1_genomic.fna
3664	Leptospira interrogans	GCA_001010765.1_ASM101076v1_genomic.fna
3665	Fusobacterium nucleatum	GCA_000400875.1_ASM40087v1_genomic.fna
3666	Bacillus anthracis	GCA_000022865.1_ASM2286v1_genomic.fna
3667	Shigella flexneri	GCA_000743955.1_ASM74395v1_genomic.fna
3668	Bifidobacterium longum	GCA_000166315.1_ASM16631v1_genomic.fna
3669	Histophilus somni	GCA_002162215.1_ASM216221v1_genomic.fna

3670	Pseudomonas syringae	GCA_000648735.3_ASM64873v3_genomic.fna
3671	Vibrio vulnificus	GCA_000746665.1_ASM74666v1_genomic.fna
3672	Mycoplasma hyopneumoniae	GCA_000400855.1_ASM40085v1_genomic.fna
3673	Streptococcus suis	GCA_000168355.3_ASM16835v3_genomic.fna
3674	Acinetobacter baumannii	GCA_000069245.1_ASM6924v1_genomic.fna
3675	Streptococcus agalactiae	GCA_000302475.3_ASM30247v3_genomic.fna
3676	Pseudomonas aeruginosa	GCA_000226155.1_ASM22615v1_genomic.fna
3677	Bacillus licheniformis	GCA_002074075.1_ASM207407v1_genomic.fna
3678	Bacteroides fragilis	GCA_001286525.1_BFBE1.1_genomic.fna
3679	Legionella pneumophila	GCA_000092625.1_ASM9262v1_genomic.fna
3680	Streptococcus thermophilus	GCA_000182875.1_ASM18287v1_genomic.fna
3681	Shigella sonnei	GCA_002142635.1_ASM214263v1_genomic.fna
3682	Shewanella baltica	GCA_000021665.1_ASM2166v1_genomic.fna
3683	Lactobacillus reuteri	GCA_000410995.1_ASM41099v1_genomic.fna
3684	Bacillus pumilus	GCA_001191605.1_ASM119160v1_genomic.fna
3685	Corynebacterium glutamicum	GCA_000404145.1_ASM40414v1_genomic.fna
3686	Thermus thermophilus	GCA_002355995.1_ASM235599v1_genomic.fna
3687	Burkholderia cenocepacia	GCA_000236215.4_ASM23621v4_genomic.fna
3688	Mycoplasma genitalium	GCA_000292505.1_ASM29250v1_genomic.fna
3689	Chlamydia trachomatis	GCA_000026925.1_ASM2692v1_genomic.fna
3690	Burkholderia mallei	GCA_000755785.1_ASM75578v1_genomic.fna
3691	Ehrlichia chaffeensis	GCA_000632905.1_ASM63290v1_genomic.fna
3692	Bacillus thuringiensis	GCA_000190515.1_ASM19051v1_genomic.fna
3693	Burkholderia pseudomallei	GCA_000260515.1_ASM26051v1_genomic.fna
3694	Ralstonia solanacearum	GCA_001586135.1_ASM158613v1_genomic.fna
3695	Rhodopseudomonas palustris	GCA_000195775.1_ASM19577v1_genomic.fna
3696	Streptococcus salivarius	GCA_000448685.2_ASM44868v2_genomic.fna
3697	Vibrio cholerae	GCA_000022585.1_ASM2258v1_genomic.fna
3698	Rhodobacter sphaeroides	GCA_001576595.1_ASM157659v1_genomic.fna
3699	Yersinia pseudotuberculosis	GCA_000750315.1_ASM75031v1_genomic.fna
3700	Francisella tularensis	GCA_000014605.1_ASM1460v1_genomic.fna
3701	Brucella abortus	GCA_000740135.1_ASM74013v1_genomic.fna
3702	Lactobacillus delbrueckii	GCA_001469775.1_ASM146977v1_genomic.fna
3703	Mycoplasma capricolum	GCA_000953235.1_ILRI181.1_genomic.fna

3704	Xanthomonas citri	GCA_000961155.1_ASM96115v1_genomic.fna
3705	Campylobacter fetus	GCA_000759485.1_ASM75948v1_genomic.fna
3706	Xanthomonas oryzae	GCA_000948075.1_ASM94807v1_genomic.fna
3707	Clostridioides difficile	GCA_000953275.1_CD630DERM_genomic.fna
3708	Aeromonas salmonicida	GCA_002313065.1_ASM231306v1_genomic.fna
3709	Coxiella burnetii	GCA_000019885.1_ASM1988v1_genomic.fna
3710	Lactobacillus casei	GCA_000309565.2_ASM30956v2_genomic.fna
3711	Bacillus subtilis	GCA_000227465.1_ASM22746v1_genomic.fna
3712	Rickettsia rickettsii	GCA_000283815.1_ASM28381v1_genomic.fna
3713	Lactobacillus helveticus	GCA_000422165.1_ASM42216v1_genomic.fna
3714	Vibrio parahaemolyticus	GCA_000568495.1_ASM56849v1_genomic.fna
3715	Methylobacterium extorquens	GCA_001971665.1_ASM197166v1_genomic.fna
3716	Lactobacillus fermentum	GCA_001742205.1_ASM174220v1_genomic.fna
3717	Porphyromonas gingivalis	GCA_001274615.1_ASM127461v1_genomic.fna
3718	Ureaplasma parvum	GCA_002205535.1_ASM220553v1_genomic.fna
3719	Mycoplasma mycoides	GCA_000253075.1_ASM25307v1_genomic.fna
3720	Burkholderia thailandensis	GCA_000567945.1_ASM56794v1_genomic.fna
3721	Phaeobacter gallaeciensis	GCA_002381325.1_ASM238132v1_genomic.fna
3722	Burkholderia multivorans	GCA_001718755.1_ASM171875v1_genomic.fna
3723	Rickettsia prowazekii	GCA_000277205.1_ASM27720v1_genomic.fna
3724	Clostridium botulinum	GCA_000017025.1_ASM1702v1_genomic.fna
3725	Borrelia burgdorferi	GCA_000444465.1_ASM44446v1_genomic.fna
3726	Rhizobium leguminosarum	GCA_000023185.1_ASM2318v1_genomic.fna
3727	Clavibacter michiganensis	GCA_001280205.1_ASM128020v1_genomic.fna
3728	Brucella canis	GCA_001715365.1_ASM171536v1_genomic.fna
3729	Brucella suis	GCA_000600055.1_ASM60005v1_genomic.fna
3730	Enterococcus faecalis	GCA_000281195.1_ASM28119v1_genomic.fna
3731	Klebsiella pneumoniae	GCA_000220485.1_ASM22048v1_genomic.fna
3732	Rhizobium etli	GCA_001664265.1_ASM166426v1_genomic.fna
3733	Bifidobacterium animalis	GCA_000092765.1_ASM9276v1_genomic.fna
3734	Chlamydia psittaci	GCA_000270425.1_ASM27042v1_genomic.fna
3735	Bacillus amyloliquefaciens	GCA_000242855.2_ASM24285v2_genomic.fna
3736	Neisseria gonorrhoeae	GCA_001047255.1_ASM104725v1_genomic.fna
3737	Streptococcus mutans	GCA_000817065.1_ASM81706v1_genomic.fna

3738	Enterococcus faecium	GCA_000737555.1_ASM73755v1_genomic.fna
3739	Bacillus mycoides	GCA_002104575.1_ASM210457v1_genomic.fna
3740	Alteromonas macleodii	GCA_001562235.1_ASM156223v1_genomic.fna
3741	Stenotrophomonas maltophilia	GCA_001274595.1_ASM127459v1_genomic.fna
3742	Bifidobacterium bifidum	GCA_001281345.1_ASM128134v1_genomic.fna
3743	Streptococcus equi	GCA_000696505.1_ASM69650v1_genomic.fna
3744	Zymomonas mobilis	GCA_000277755.1_ASM27775v1_genomic.fna
3745	Haemophilus parasuis	GCA_002055565.1_ASM205556v1_genomic.fna
3746	Acetobacter pasteurianus	GCA_000010885.1_ASM1088v1_genomic.fna
3747	Pasteurella multocida	GCA_000259545.1_ASM25954v1_genomic.fna
3748	Francisella philomiragia	GCA_000833295.1_ASM83329v1_genomic.fna
3749	Lactobacillus rhamnosus	GCA_000418475.1_ASM41847v1_genomic.fna
3750	Caulobacter vibrioides	GCA_002310375.2_ASM231037v2_genomic.fna
3751	Brucella melitensis	GCA_000227645.1_ASM22764v1_genomic.fna
3752	Bacillus megaterium	GCA_000225265.1_ASM22526v1_genomic.fna
3753	Sinorhizobium meliloti	GCA_000304415.1_RM41_genomic.fna
3754	Bordetella bronchiseptica	GCA_001704355.1_ASM170435v1_genomic.fna
3755	Haemophilus ducreyi	GCA_001647765.1_ASM164776v1_genomic.fna
3756	Streptococcus gordonii	GCA_002386285.1_ASM238628v1_genomic.fna
3757	Bordetella pertussis	GCA_000812165.1_ASM81216v1_genomic.fna
3758	Corynebacterium diphtheriae	GCA_000241915.1_ASM24191v1_genomic.fna
3759	Mycobacterium smegmatis	GCA_000767705.1_ASM76770v1_genomic.fna
3760	Mycoplasma pneumoniae	GCA_000319675.2_ASM31967v2_genomic.fna
3761	Thermotoga maritima	GCA_000978555.1_ASM97855v1_genomic.fna
3762	Yersinia enterocolitica	GCA_000834735.1_ASM83473v1_genomic.fna
3763	Dehalococcoides mccartyi	GCA_000025025.1_ASM2502v1_genomic.fna
3764	Chlamydia muridarum	GCA_000772145.1_ASM77214v1_genomic.fna
3765	Leuconostoc mesenteroides	GCA_001583825.1_ASM158382v1_genomic.fna
3766	Lactobacillus plantarum	GCA_000392485.2_ASM39248v2_genomic.fna
3767	Chlamydia abortus	GCA_900002515.1_1BCEVAC_genomic.fna
3768	Lactobacillus acidophilus	GCA_002286215.1_ASM228621v1_genomic.fna
3769	Mannheimia haemolytica	GCA_000422145.1_ASM42214v1_genomic.fna
3770	Pediococcus pentosaceus	GCA_002202155.1_ASM220215v1_genomic.fna
3771	Lactobacillus brevis	GCA_002117325.1_ASM211732v1_genomic.fna

3772	Serratia marcescens	GCA_001022215.1_ASM102221v1_genomic.fna
3773	Moorella thermoacetica	GCA_001874605.1_ASM187460v1_genomic.fna
3774	Mycoplasma gallisepticum	GCA_000286675.1_ASM28667v1_genomic.fna
3775	Burkholderia vietnamiensis	GCA_001718915.1_ASM171891v1_genomic.fna
3776	Propionibacterium acnes	GCA_000231215.1_ASM23121v1_genomic.fna
3777	Campylobacter coli	GCA_000583795.1_ASM58379v1_genomic.fna
3778	Mycoplasma bovis	GCA_001043135.1_ASM104313v1_genomic.fna
3779	Campylobacter lari	GCA_000816405.1_ASM81640v1_genomic.fna
3780	Proteus mirabilis	GCA_002055685.1_ASM205568v1_genomic.fna
3781	Klebsiella oxytoca	GCA_001548355.1_ASM154835v1_genomic.fna
3782	Clostridium beijerinckii	GCA_002003345.1_ASM200334v1_genomic.fna
3783	Cronobacter sakazakii	GCA_001277275.1_ASM127727v1_genomic.fna
3784	Lactobacillus salivarius	GCA_001723525.1_ASM172352v1_genomic.fna
3785	Gluconobacter oxydans	GCA_900175995.1_ASM90017599v1_genomic.fna
3786	Enterobacter cloacae	GCA_000512375.1_ASM51237v1_genomic.fna
3787	Moraxella catarrhalis	GCA_002080125.1_ASM208012v1_genomic.fna
3788	Listeria ivanovii	GCA_000763515.1_ASM76351v1_genomic.fna
3789	Bifidobacterium breve	GCA_000568975.1_ASM56897v1_genomic.fna
3790	Mycobacterium abscessus	GCA_000758385.1_ASM75838v1_genomic.fna
3791	Bacillus coagulans	GCA_001039495.1_ASM103949v1_genomic.fna
3792	Paenibacillus polymyxa	GCA_000597985.1_ASM59798v1_genomic.fna
3793	Aeromonas hydrophila	GCA_000633175.1_ASM63317v1_genomic.fna
3794	Pseudomonas stutzeri	GCA_000267545.1_ASM26754v1_genomic.fna
3795	Granulibacter bethesdensis	GCA_001889445.1_ASM188944v1_genomic.fna
3796	Burkholderia ubonensis	GCA_001718855.1_ASM171885v1_genomic.fna
3797	Flavobacterium psychrophilum	GCA_000767095.1_ASM76709v1_genomic.fna
3798	Clostridium butyricum	GCA_001886875.1_ASM188687v1_genomic.fna
3799	Bdellovibrio bacteriovorus	GCA_000317895.1_ASM31789v1_genomic.fna
3800	Lactobacillus johnsonii	GCA_001714745.1_ASM171474v1_genomic.fna
3801	Lactobacillus sakei	GCA_002370375.1_ASM237037v1_genomic.fna
3802	Paenibacillus larvae	GCA_002082155.1_ASM208215v1_genomic.fna
3803	Mycobacterium intracellulare	GCA_002285695.1_ASM228569v1_genomic.fna
3804	Streptomyces albus	GCA_001577385.1_ASM157738v1_genomic.fna
3805	Aggregatibacter actinomycetemcomitans	GCA_001594245.1_ASM159424v1_genomic.fna

3806	Serratia symbiotica	GCA_900016775.1_STsPazieg1.0_genomic.fna
3807	Streptococcus intermedius	GCA_002356055.1_ASM235605v1_genomic.fna
3808	Mycoplasma hyorhinis	GCA_000496815.1_ASM49681v1_genomic.fna
3809	Gardnerella vaginalis	GCA_001953155.1_ASM195315v1_genomic.fna
3810	Lactobacillus paracasei	GCA_001191565.1_ASM119156v1_genomic.fna
3811	Bacillus atrophaeus	GCA_002688645.1_ASM268864v1_genomic.fna
3812	Klebsiella variicola	GCA_001989495.1_ASM198949v1_genomic.fna
3813	Achromobacter xylosoxidans	GCA_001558755.1_ASM155875v1_genomic.fna
3814	Vibrio alginolyticus	GCA_002119505.1_ASM211950v1_genomic.fna
3815	Pediococcus acidilactici	GCA_002174215.1_ASM217421v1_genomic.fna
3816	Corynebacterium pseudotuberculosis	GCA_000221625.1_ASM22162v1_genomic.fna
3817	Acinetobacter pittii	GCA_002158945.1_ASM215894v1_genomic.fna
3818	Riemerella anatipestifer	GCA_000331695.1_ASM33169v1_genomic.fna
3819	Staphylococcus lugdunensis	GCA_002073395.1_ASM207339v1_genomic.fna
3820	Streptococcus anginosus	GCA_001412635.1_ASM141263v1_genomic.fna
3821	Pantoea ananatis	GCA_001543055.1_ASM154305v1_genomic.fna
3822	Streptococcus gallolyticus	GCA_001477575.1_ASM147757v1_genomic.fna
3823	Taylorella equigenitalis	GCA_002288155.1_ASM228815v1_genomic.fna
3824	Ketogulonicigenium vulgare	GCA_001855295.1_ASM185529v1_genomic.fna
3825	Citrobacter freundii	GCA_001702455.1_ASM170245v1_genomic.fna
3826	Vibrio anguillarum	GCA_002163795.1_ASM216379v1_genomic.fna
3827	Enterobacter hormaechei	GCA_000814205.1_ASM81420v1_genomic.fna
3828	Weissella cibaria	GCA_002243305.1_ASM224330v1_genomic.fna
3829	Klebsiella aerogenes	GCA_001571545.2_ASM157154v2_genomic.fna
3830	Staphylococcus pseudintermedius	GCA_001685665.2_ASM168566v2_genomic.fna
3831	Corynebacterium ulcerans	GCA_000767415.1_ASM76741v1_genomic.fna
3832	Burkholderia cepacia	GCA_000974835.2_ASM97483v2_genomic.fna
3833	Lactobacillus curvatus	GCA_002224505.1_ASM222450v1_genomic.fna
3834	Pediococcus damnosus	GCA_001611155.1_ASM161115v1_genomic.fna
3835	Prevotella intermedia	GCA_002355195.1_ASM235519v1_genomic.fna
3836	Clostridium pasteurianum	GCA_001856645.1_ASM185664v1_genomic.fna
3837	Vibrio campbellii	GCA_002142655.1_ASM214265v1_genomic.fna
3838	Rickettsia japonica	GCA_002357155.1_ASM235715v1_genomic.fna
3839	Moraxella bovoculi	GCA_000988705.1_ASM98870v1_genomic.fna

3840	Flavobacterium columnare	GCA_001936395.1_ASM193639v1_genomic.fna
3841	Piscirickettsia salmonis	GCA_001534725.1_ASM153472v1_genomic.fna
3842	Francisella noatunensis	GCA_001042565.2_ASM104256v2_genomic.fna
3843	Elizabethkingia anophelis	GCA_001618465.2_ASM161846v2_genomic.fna
3844	Rhizobium phaseoli	GCA_001664185.1_ASM166418v1_genomic.fna
3845	Xanthomonas fragariae	GCA_900183995.1_PD5205.1_genomic.fna
3846	Pseudomonas chlororaphis	GCA_001602135.1_ASM160213v1_genomic.fna
3847	Serratia plymuthica	GCA_001663115.1_ASM166311v1_genomic.fna
3848	Acidipropionibacterium acidipropionici	GCA_001975205.1_ASM197520v1_genomic.fna
3849	Bordetella holmesii	GCA_001704215.1_ASM170421v1_genomic.fna
3850	Bacillus safensis	GCA_002077215.1_ASM207721v1_genomic.fna
3851	Vibrio natriegens	GCA_001680085.1_ASM168008v1_genomic.fna
3852	Raoultella ornithinolytica	GCA_001866535.1_ASM186653v1_genomic.fna
3853	Clostridium saccharobutylicum	GCA_002003385.1_ASM200338v1_genomic.fna
3854	Pandoraea pnomenusa	GCA_001636275.1_ASM163627v1_genomic.fna
3855	Bacillus velezensis	GCA_000341875.1_ASM34187v1_genomic.fna
3856	Klebsiella michiganensis	GCA_000963575.1_ASM96357v1_genomic.fna
3857	Staphylococcus schleiferi	GCA_001548255.1_ASM154825v1_genomic.fna
3858	Oblitimonas alkaliphila	GCA_001267275.1_ASM126727v1_genomic.fna
3859	Mycobacterium chimaera	GCA_002219285.1_ASM221928v1_genomic.fna
3860	Alteromonas mediterranea	GCA_000439555.1_ASM43955v1_genomic.fna
3861	Lactobacillus backii	GCA_001663755.1_ASM166375v1_genomic.fna
3862	Campylobacter pinnipediorum	GCA_002022005.1_ASM202200v1_genomic.fna
3863	Alteromonas mediterranea	GCA_000439575.1_ASM43957v1_genomic.fna
3864	Mycobacterium chimaera	GCA_002240155.1_ASM224015v1_genomic.fna
3865	Oblitimonas alkaliphila	GCA_001267295.1_ASM126729v1_genomic.fna
3866	Klebsiella michiganensis	GCA_002216835.1_ASM221683v1_genomic.fna
3867	Bacillus velezensis	GCA_000455565.1_ucmb5033v1_genomic.fna
3868	Raoultella ornithinolytica	GCA_002214825.1_ASM221482v1_genomic.fna
3869	Serratia plymuthica	GCA_001663135.1_ASM166313v1_genomic.fna
3870	Pseudomonas chlororaphis	GCA_002355875.1_ASM235587v1_genomic.fna
3871	Rhizobium phaseoli	GCA_001664205.1_ASM166420v1_genomic.fna
3872	Elizabethkingia anophelis	GCA_001618505.2_ASM161850v2_genomic.fna
3873	Francisella noatunensis	GCA_001190905.2_ASM119090v2_genomic.fna

3874	Piscirickettsia salmonis	GCA_001746795.1_ASM174679v1_genomic.fna
3875	Moraxella bovoculi	GCA_000988745.1_ASM98874v1_genomic.fna
3876	Rickettsia japonica	GCA_002357175.1_ASM235717v1_genomic.fna
3877	Vibrio campbellii	GCA_002163755.1_ASM216375v1_genomic.fna
3878	Clostridium pasteurianum	GCA_001856695.1_ASM185669v1_genomic.fna
3879	Burkholderia cepacia	GCA_001718395.1_ASM171839v1_genomic.fna
3880	Corynebacterium ulcerans	GCA_000767645.1_ASM76764v1_genomic.fna
3881	Klebsiella aerogenes	GCA_001593585.1_ASM159358v1_genomic.fna
3882	Vibrio anguillarum	GCA_002211505.1_ASM221150v1_genomic.fna
3883	Enterobacter hormaechei	GCA_000814225.1_ASM81422v1_genomic.fna
3884	Citrobacter freundii	GCA_001718055.1_ASM171805v1_genomic.fna
3885	Taylorella equigenitalis	GCA_002288205.1_ASM228820v1_genomic.fna
3886	Pantoea ananatis	GCA_002224585.1_ASM222458v1_genomic.fna
3887	Streptococcus anginosus	GCA_001697145.1_ASM169714v1_genomic.fna
3888	Staphylococcus lugdunensis	GCA_002250095.2_ASM225009v2_genomic.fna
3889	Riemerella anatipestifer	GCA_000734055.1_ASM73405v1_genomic.fna
3890	Corynebacterium pseudotuberculosis	GCA_000227175.1_ASM22717v1_genomic.fna
3891	Vibrio alginolyticus	GCA_002119525.1_ASM211952v1_genomic.fna
3892	Klebsiella variicola	GCA_001989515.1_ASM198951v1_genomic.fna
3893	Lactobacillus paracasei	GCA_001244395.1_ASM124439v1_genomic.fna
3894	Mycoplasma hyorhinis	GCA_001705605.1_ASM170560v1_genomic.fna
3895	Aggregatibacter actinomycetemcomitans	GCA_001690155.1_ASM169015v1_genomic.fna
3896	Streptomyces albus	GCA_001753425.1_ASM175342v1_genomic.fna
3897	Bdellovibrio bacteriovorus	GCA_000691605.1_ASM69160v1_genomic.fna
3898	Lactobacillus johnsonii	GCA_002176835.1_ASM217683v1_genomic.fna
3899	Flavobacterium psychrophilum	GCA_000831185.1_ASM83118v1_genomic.fna
3900	Burkholderia ubonensis	GCA_001718975.1_ASM171897v1_genomic.fna
3901	Granulibacter bethesdensis	GCA_001889465.1_ASM188946v1_genomic.fna
3902	Pseudomonas stutzeri	GCA_000279165.1_ASM27916v1_genomic.fna
3903	Aeromonas hydrophila	GCA_000635955.1_ASM63595v1_genomic.fna
3904	Paenibacillus polymyxa	GCA_000819665.1_ASM81966v1_genomic.fna
3905	Bacillus coagulans	GCA_001870065.1_ASM187006v1_genomic.fna
3906	Mycobacterium abscessus	GCA_000770175.1_ASM77017v1_genomic.fna
3907	Bifidobacterium breve	GCA_000569015.1_ASM56901v1_genomic.fna

3908	Listeria ivanovii	GCA_900187025.1_49923_F01_genomic.fna
3909	Enterobacter cloacae	GCA_000724505.1_ASM72450v1_genomic.fna
3910	Lactobacillus salivarius	GCA_002162055.1_ASM216205v1_genomic.fna
3911	Klebsiella oxytoca	GCA_001870185.1_ASM187018v1_genomic.fna
3912	Proteus mirabilis	GCA_002180115.1_ASM218011v1_genomic.fna
3913	Campylobacter lari	GCA_000816425.1_ASM81642v1_genomic.fna
3914	Mycoplasma bovis	GCA_001611865.1_ASM161186v1_genomic.fna
3915	Campylobacter coli	GCA_000954195.1_ASM95419v1_genomic.fna
3916	Propionibacterium acnes	GCA_000240015.1_ASM24001v1_genomic.fna
3917	Mycoplasma gallisepticum	GCA_000286695.1_ASM28669v1_genomic.fna
3918	Lactobacillus brevis	GCA_002117345.1_ASM211734v1_genomic.fna
3919	Mannheimia haemolytica	GCA_000427275.1_ASM42727v1_genomic.fna
3920	Lactobacillus plantarum	GCA_000412205.1_ASM41220v1_genomic.fna
3921	Serratia marcescens	GCA_001280365.1_ASM128036v1_genomic.fna
3922	Leuconostoc mesenteroides	GCA_001886915.1_ASM188691v1_genomic.fna
3923	Chlamydia muridarum	GCA_000830945.1_ASM83094v1_genomic.fna
3924	Dehalococcoides mccartyi	GCA_000025585.1_ASM2558v1_genomic.fna
3925	Yersinia enterocolitica	GCA_000968115.1_ASM96811v1_genomic.fna
3926	Thermotoga maritima	GCA_000978575.1_ASM97857v1_genomic.fna
3927	Mycobacterium smegmatis	GCA_001457595.1_NCTC8159_genomic.fna
3928	Mycoplasma pneumoniae	GCA_000331085.2_ASM33108v2_genomic.fna
3929	Corynebacterium diphtheriae	GCA_000241935.1_ASM24193v1_genomic.fna
3930	Haemophilus ducreyi	GCA_001647795.1_ASM164779v1_genomic.fna
3931	Bordetella pertussis	GCA_001013565.1_BPD420_genomic.fna
3932	Sinorhizobium meliloti	GCA_000320385.2_ASM32038v2_genomic.fna
3933	Bacillus megaterium	GCA_001050455.1_ASM105045v1_genomic.fna
3934	Caulobacter vibrioides	GCA_002310455.1_ASM231045v1_genomic.fna
3935	Brucella melitensis	GCA_000740355.1_ASM74035v1_genomic.fna
3936	Lactobacillus rhamnosus	GCA_000418495.1_ASM41849v1_genomic.fna
3937	Francisella philomiragia	GCA_000833315.1_ASM83331v1_genomic.fna
3938	Pasteurella multocida	GCA_000512395.1_ASM51239v1_genomic.fna
3939	Acetobacter pasteurianus	GCA_000010905.1_ASM1090v1_genomic.fna
3940	Zymomonas mobilis	GCA_000498655.1_ASM49865v1_genomic.fna
3941	Stenotrophomonas maltophilia	GCA_001274655.1_ASM127465v1_genomic.fna

3942	Alteromonas macleodii	GCA_001578515.1_ASM157851v1_genomic.fna
3943	Enterococcus faecium	GCA_001298485.1_ASM129848v1_genomic.fna
3944	Streptococcus mutans	GCA_001558215.1_ASM155821v1_genomic.fna
3945	Neisseria gonorrhoeae	GCA_001047275.1_ASM104727v1_genomic.fna
3946	Bacillus amyloliquefaciens	GCA_000262385.1_ASM26238v1_genomic.fna
3947	Chlamydia psittaci	GCA_000270445.1_ASM27044v1_genomic.fna
3948	Bifidobacterium animalis	GCA_000220885.1_ASM22088v1_genomic.fna
3949	Rhizobium etli	GCA_001908375.1_ASM190837v1_genomic.fna
3950	Enterococcus faecalis	GCA_000317915.1_ASM31791v1_genomic.fna
3951	Brucella suis	GCA_000698245.1_ASM69824v1_genomic.fna
3952	Brucella canis	GCA_001715405.1_ASM171540v1_genomic.fna
3953	Borreliella burgdorferi	GCA_002151465.1_ASM215146v1_genomic.fna
3954	Rhizobium leguminosarum	GCA_000520875.1_ASM52087v1_genomic.fna
3955	Klebsiella pneumoniae	GCA_000281435.2_ASM28143v2_genomic.fna
3956	Clostridium botulinum	GCA_000017065.1_ASM1706v1_genomic.fna
3957	Rickettsia prowazekii	GCA_000277245.1_ASM27724v1_genomic.fna
3958	Burkholderia multivorans	GCA_001718995.1_ASM171899v1_genomic.fna
3959	Phaeobacter gallaeciensis	GCA_002381365.1_ASM238136v1_genomic.fna
3960	Burkholderia thailandensis	GCA_000706745.1_ASM70674v1_genomic.fna
3961	Ureaplasma parvum	GCA_002205595.1_ASM220559v1_genomic.fna
3962	Mycoplasma mycoides	GCA_000800785.1_ASM80078v1_genomic.fna
3963	Porphyromonas gingivalis	GCA_001314265.1_ASM131426v1_genomic.fna
3964	Lactobacillus fermentum	GCA_001941785.1_ASM194178v1_genomic.fna
3965	Vibrio parahaemolyticus	GCA_001244315.1_ASM124431v1_genomic.fna
3966	Rickettsia rickettsii	GCA_000283935.1_ASM28393v1_genomic.fna
3967	Lactobacillus helveticus	GCA_000525715.1_ASM52571v1_genomic.fna
3968	Bacillus subtilis	GCA_000227485.1_ASM22748v1_genomic.fna
3969	Lactobacillus casei	GCA_000318035.1_ASM31803v1_genomic.fna
3970	Coxiella burnetii	GCA_000168875.3_ASM16887v3_genomic.fna
3971	Clostridioides difficile	GCA_001577795.1_ASM157779v1_genomic.fna
3972	Xanthomonas oryzae	GCA_001021915.1_ASM102191v1_genomic.fna
3973	Campylobacter fetus	GCA_000759515.1_ASM75951v1_genomic.fna
3974	Xanthomonas citri	GCA_000961175.1_ASM96117v1_genomic.fna
3975	Mycoplasma capricolum	GCA_000953375.1_F38.1_genomic.fna

3976	Lactobacillus delbrueckii	GCA_001888905.1_ASM188890v1_genomic.fna
3977	Brucella abortus	GCA_000740155.1_ASM74015v1_genomic.fna
3978	Francisella tularensis	GCA_000014645.1_ASM1464v1_genomic.fna
3979	Rhodobacter sphaeroides	GCA_001685625.1_ASM168562v1_genomic.fna
3980	Yersinia pseudotuberculosis	GCA_000834295.1_ASM83429v1_genomic.fna
3981	Streptococcus salivarius	GCA_001543085.1_ASM154308v1_genomic.fna
3982	Vibrio cholerae	GCA_000166455.2_ASM16645v2_genomic.fna
3983	Ralstonia solanacearum	GCA_001587135.1_ASM158713v1_genomic.fna
3984	Burkholderia pseudomallei	GCA_000294635.1_ASM29463v1_genomic.fna
3985	Bacillus thuringiensis	GCA_000193355.1_ASM19335v1_genomic.fna
3986	Ehrlichia chaffeensis	GCA_000632925.1_ASM63292v1_genomic.fna
3987	Burkholderia mallei	GCA_000755845.1_ASM75584v1_genomic.fna
3988	Burkholderia cenocepacia	GCA_000755725.1_ASM75572v1_genomic.fna
3989	Chlamydia trachomatis	GCA_000068525.2_ASM6852v2_genomic.fna
3990	Corynebacterium glutamicum	GCA_000404185.1_ASM40418v1_genomic.fna
3991	Bacillus pumilus	GCA_001431145.1_ASM143114v1_genomic.fna
3992	Shewanella baltica	GCA_000147735.3_ASM14773v3_genomic.fna
3993	Lactobacillus reuteri	GCA_000439275.1_ASM43927v1_genomic.fna
3994	Shigella sonnei	GCA_002150905.1_ASM215090v1_genomic.fna
3995	Streptococcus thermophilus	GCA_000262675.1_ASM26267v1_genomic.fna
3996	Legionella pneumophila	GCA_000239175.1_ASM23917v1_genomic.fna
3997	Bacteroides fragilis	GCA_001682215.1_ASM168221v1_genomic.fna
3998	Bacillus licheniformis	GCA_002074095.1_ASM207409v1_genomic.fna
3999	Pseudomonas aeruginosa	GCA_000271365.1_ASM27136v1_genomic.fna
4000	Streptococcus agalactiae	GCA_000427035.1_09mas018883_genomic.fna
4001	Acinetobacter baumannii	GCA_000186665.4_ASM18666v4_genomic.fna
4002	Streptococcus suis	GCA_000186405.1_ASM18640v1_genomic.fna
4003	Mycoplasma hyopneumoniae	GCA_000427215.1_ASM42721v1_genomic.fna
4004	Vibrio vulnificus	GCA_001433435.1_ASM143343v1_genomic.fna
4005	Histophilus somni	GCA_002162235.1_ASM216223v1_genomic.fna
4006	Pseudomonas syringae	GCA_000988395.1_ASM98839v1_genomic.fna
4007	Bifidobacterium longum	GCA_000196555.1_ASM19655v1_genomic.fna
4008	Shigella flexneri	GCA_000743995.1_ASM74399v1_genomic.fna
4009	Leptospira interrogans	GCA_001047635.1_ASM104763v1_genomic.fna

4010	Fusobacterium nucleatum	GCA_001296085.1_ASM129608v1_genomic.fna
4011	Bacillus anthracis	GCA_000258885.1_ASM25888v1_genomic.fna
4012	Streptococcus pneumoniae	GCA_000019005.1_ASM1900v1_genomic.fna
4013	Streptococcus pyogenes	GCA_000011665.1_ASM1166v1_genomic.fna
4014	Xylella fastidiosa	GCA_000698805.1_ASM69880v1_genomic.fna
4015	Pseudomonas putida	GCA_000183645.1_ASM18364v1_genomic.fna
4016	Neisseria meningitidis	GCA_000083565.1_ASM8356v1_genomic.fna
4017	Buchnera aphidicola	GCA_000217635.1_ASM21763v1_genomic.fna
4018	Chlamydia pneumoniae	GCA_001007025.1_Wien1_genomic.fna
4019	Helicobacter pylori	GCA_000021165.1_ASM2116v1_genomic.fna
4020	Haemophilus influenzae	GCA_000200475.1_ASM20047v1_genomic.fna
4021	Prochlorococcus marinus	GCA_000015645.1_ASM1564v1_genomic.fna
4022	Mycobacterium tuberculosis	GCA_000153685.2_ASM15368v2_genomic.fna
4023	Mycobacterium avium	GCA_000770235.1_ASM77023v1_genomic.fna
4024	Mycobacterium bovis	GCA_000338715.2_ASM33871v2_genomic.fna
4025	Clostridium perfringens	GCA_001579785.1_ASM157978v1_genomic.fna
4026	Listeria monocytogenes	GCA_000093125.2_ASM9312v2_genomic.fna
4027	Bacillus cereus	GCA_000021225.1_ASM2122v1_genomic.fna
4028	Lactococcus lactis	GCA_000192705.1_ASM19270v1_genomic.fna
4029	Staphylococcus epidermidis	GCA_002085695.1_ASM208569v1_genomic.fna
4030	Staphylococcus aureus	GCA_000010445.1_ASM1044v1_genomic.fna
4031	Yersinia pestis	GCA_000016445.1_ASM1644v1_genomic.fna
4032	Campylobacter jejuni	GCA_000017905.1_ASM1790v1_genomic.fna
4033	Xanthomonas campestris	GCA_001186415.1_ASM118641v1_genomic.fna
4034	Pseudomonas fluorescens	GCA_000730425.1_ASM73042v1_genomic.fna
4035	Salmonella enterica	GCA_000009525.1_ASM952v1_genomic.fna
4036	Salmonella enterica	GCA_000011885.1_ASM1188v1_genomic.fna
4037	Campylobacter jejuni	GCA_000025425.1_ASM2542v1_genomic.fna
4038	Xanthomonas campestris	GCA_001186465.1_ASM118646v1_genomic.fna
4039	Pseudomonas fluorescens	GCA_000934565.1_ASM93456v1_genomic.fna
4040	Yersinia pestis	GCA_000018805.1_ASM1880v1_genomic.fna
4041	Staphylococcus aureus	GCA_000010465.1_ASM1046v1_genomic.fna
4042	Lactococcus lactis	GCA_000236475.1_ASM23647v1_genomic.fna
4043	Staphylococcus epidermidis	GCA_002215535.1_ASM221553v1_genomic.fna

4044	Bacillus cereus	GCA_000021305.1_ASM2130v1_genomic.fna
4045	Clostridium perfringens	GCA_001854085.1_ASM185408v1_genomic.fna
4046	Listeria monocytogenes	GCA_000168575.2_ASM16857v2_genomic.fna
4047	Mycobacterium avium	GCA_000829075.1_ASM82907v1_genomic.fna
4048	Mycobacterium bovis	GCA_000758245.1_ASM75824v1_genomic.fna
4049	Mycobacterium tuberculosis	GCA_000154585.2_ASM15458v2_genomic.fna
4050	Haemophilus influenzae	GCA_000210875.1_ASM21087v1_genomic.fna
4051	Prochlorococcus marinus	GCA_000015665.1_ASM1566v1_genomic.fna
4052	Escherichia coli	GCA_000007445.1_ASM744v1_genomic.fna
4053	Helicobacter pylori	GCA_000021465.1_ASM2146v1_genomic.fna
4054	Chlamydia pneumoniae	GCA_001007045.1_K7_genomic.fna
4055	Buchnera aphidicola	GCA_000225445.1_ASM22544v1_genomic.fna
4056	Neisseria meningitidis	GCA_000152165.1_ASM15216v1_genomic.fna
4057	Pseudomonas putida	GCA_000219705.1_ASM21970v1_genomic.fna
4058	Xylella fastidiosa	GCA_000698825.1_ASM69882v1_genomic.fna
4059	Streptococcus pyogenes	GCA_000011765.2_ASM1176v2_genomic.fna
4060	Streptococcus pneumoniae	GCA_000019025.1_ASM1902v1_genomic.fna
4061	Fusobacterium nucleatum	GCA_001296145.1_ASM129614v1_genomic.fna
4062	Leptospira interrogans	GCA_001047655.1_ASM104765v1_genomic.fna
4063	Shigella flexneri	GCA_000953035.1_NCTC1_genomic.fna
4064	Bacillus anthracis	GCA_000512835.2_ASM51283v2_genomic.fna
4065	Bifidobacterium longum	GCA_000196575.1_ASM19657v1_genomic.fna
4066	Pseudomonas syringae	GCA_000988485.1_ASM98848v1_genomic.fna
4067	Histophilus somni	GCA_002162255.1_ASM216225v1_genomic.fna
4068	Vibrio vulnificus	GCA_001558515.1_ASM155851v1_genomic.fna
4069	Mycoplasma hyopneumoniae	GCA_002257505.1_ASM225750v1_genomic.fna
4070	Streptococcus suis	GCA_000204625.1_ASM20462v1_genomic.fna
4071	Acinetobacter baumannii	GCA_000187205.4_ASM18720v4_genomic.fna
4072	Streptococcus agalactiae	GCA_000427075.1_ILRI005_genomic.fna
4073	Pseudomonas aeruginosa	GCA_000271985.2_ASM27198v2_genomic.fna
4074	Bacillus licheniformis	GCA_002074115.1_ASM207411v1_genomic.fna
4075	Legionella pneumophila	GCA_000306845.1_ASM30684v1_genomic.fna
4076	Streptococcus thermophilus	GCA_000698885.1_ASM69888v1_genomic.fna
4077	Lactobacillus reuteri	GCA_001046835.1_ASM104683v1_genomic.fna

4078	Shewanella baltica	GCA_000215895.1_ASM21589v1_genomic.fna
4079	Bacillus pumilus	GCA_001431785.1_ASM143178v1_genomic.fna
4080	Corynebacterium glutamicum	GCA_000445015.1_ASM44501v1_genomic.fna
4081	Chlamydia trachomatis	GCA_000092485.1_ASM9248v1_genomic.fna
4082	Burkholderia cenocepacia	GCA_000764955.1_ASM76495v1_genomic.fna
4083	Shigella sonnei	GCA_002224605.1_ASM222460v1_genomic.fna
4084	Burkholderia mallei	GCA_000755865.1_ASM75586v1_genomic.fna
4085	Burkholderia pseudomallei	GCA_000439695.1_ASM43969v1_genomic.fna
4086	Ehrlichia chaffeensis	GCA_000632945.1_ASM63294v1_genomic.fna
4087	Bacillus thuringiensis	GCA_000292455.1_ASM29245v1_genomic.fna
4088	Ralstonia solanacearum	GCA_001587155.1_ASM158715v1_genomic.fna
4089	Vibrio cholerae	GCA_000195065.1_ASM19506v1_genomic.fna
4090	Rhodopseudomonas palustris	GCA_000013685.1_ASM1368v1_genomic.fna
4091	Streptococcus salivarius	GCA_002073835.1_ASM207383v1_genomic.fna
4092	Rhodobacter sphaeroides	GCA_002706325.1_ASM270632v1_genomic.fna
4093	Yersinia pseudotuberculosis	GCA_000834355.1_ASM83435v1_genomic.fna
4094	Francisella tularensis	GCA_000016105.1_ASM1610v1_genomic.fna
4095	Brucella abortus	GCA_000740175.1_ASM74017v1_genomic.fna
4096	Lactobacillus delbrueckii	GCA_001888925.1_ASM188892v1_genomic.fna
4097	Mycoplasma capricolum	GCA_002009215.1_ASM200921v1_genomic.fna
4098	Xanthomonas citri	GCA_000961195.1_ASM96119v1_genomic.fna
4099	Campylobacter fetus	GCA_000814265.1_ASM81426v1_genomic.fna
4100	Xanthomonas oryzae	GCA_001042735.1_ASM104273v1_genomic.fna
4101	Clostridioides difficile	GCA_002007885.1_ASM200788v1_genomic.fna
4102	Coxiella burnetii	GCA_000367725.3_Z3055_genomic.fna
4103	Bacillus subtilis	GCA_000293765.1_ASM29376v1_genomic.fna
4104	Lactobacillus casei	GCA_000418515.1_ASM41851v1_genomic.fna
4105	Lactobacillus helveticus	GCA_000961015.1_ASM96101v1_genomic.fna
4106	Rickettsia rickettsii	GCA_000283955.1_ASM28395v1_genomic.fna
4107	Vibrio parahaemolyticus	GCA_001304775.1_ASM130477v1_genomic.fna
4108	Lactobacillus fermentum	GCA_002119645.1_ASM211964v1_genomic.fna
4109	Mycoplasma mycoides	GCA_001267815.1_ASM126781v1_genomic.fna
4110	Ureaplasma parvum	GCA_002205735.1_ASM220573v1_genomic.fna
4111	Burkholderia thailandensis	GCA_000764595.1_ASM76459v1_genomic.fna

4112	Phaeobacter gallaeciensis	GCA_002381385.1_ASM238138v1_genomic.fna
4113	Rickettsia prowazekii	GCA_000277265.1_ASM27726v1_genomic.fna
4114	Clostridium botulinum	GCA_000019305.1_ASM1930v1_genomic.fna
4115	Klebsiella pneumoniae	GCA_000281535.2_ASM28153v2_genomic.fna
4116	Borreliella burgdorferi	GCA_002151485.1_ASM215148v1_genomic.fna
4117	Rhizobium leguminosarum	GCA_001890425.1_ASM189042v1_genomic.fna
4118	Brucella canis	GCA_002591255.1_ASM259125v1_genomic.fna
4119	Brucella suis	GCA_000698285.1_ASM69828v1_genomic.fna
4120	Enterococcus faecalis	GCA_000550745.1_ASM55074v1_genomic.fna
4121	Rhizobium etli	GCA_002119845.1_ASM211984v1_genomic.fna
4122	Chlamydia psittaci	GCA_000298415.1_ASM29841v1_genomic.fna
4123	Bifidobacterium animalis	GCA_000224965.2_ASM22496v2_genomic.fna
4124	Bacillus amyloliquefaciens	GCA_000494835.1_ASM49483v1_genomic.fna
4125	Neisseria gonorrhoeae	GCA_001661085.1_ASM166108v1_genomic.fna
4126	Enterococcus faecium	GCA_001412695.1_ASM141269v1_genomic.fna
4127	Stenotrophomonas maltophilia	GCA_001274675.1_ASM127467v1_genomic.fna
4128	Zymomonas mobilis	GCA_000576125.1_ASM57612v1_genomic.fna
4129	Acetobacter pasteurianus	GCA_000010925.1_ASM1092v1_genomic.fna
4130	Pasteurella multocida	GCA_000973525.1_ASM97352v1_genomic.fna
4131	Francisella philomiragia	GCA_000833455.1_ASM83345v1_genomic.fna
4132	Lactobacillus rhamnosus	GCA_001721925.1_ASM172192v1_genomic.fna
4133	Brucella melitensis	GCA_000740415.1_ASM74041v1_genomic.fna
4134	Bacillus megaterium	GCA_002009195.1_ASM200919v1_genomic.fna
4135	Sinorhizobium meliloti	GCA_000346065.1_ASM34606v1_genomic.fna
4136	Bordetella pertussis	GCA_001307525.1_ASM130752v1_genomic.fna
4137	Haemophilus ducreyi	GCA_001647815.1_ASM164781v1_genomic.fna
4138	Corynebacterium diphtheriae	GCA_000242775.1_ASM24277v1_genomic.fna
4139	Mycoplasma pneumoniae	GCA_000387745.2_ASM38774v2_genomic.fna
4140	Yersinia enterocolitica	GCA_000987925.1_ASM98792v1_genomic.fna
4141	Dehalococcoides mccartyi	GCA_000341655.1_ASM34165v1_genomic.fna
4142	Chlamydia muridarum	GCA_000830965.1_ASM83096v1_genomic.fna
4143	Leuconostoc mesenteroides	GCA_001891125.1_ASM189112v1_genomic.fna
4144	Serratia marcescens	GCA_001294565.1_ASM129456v1_genomic.fna
4145	Lactobacillus plantarum	GCA_000604105.1_ASM60410v1_genomic.fna

4146	Mannheimia haemolytica	GCA_000439735.1_ASM43973v1_genomic.fna
4147	Lactobacillus brevis	GCA_002117375.1_ASM211737v1_genomic.fna
4148	Mycoplasma gallisepticum	GCA_000286715.1_ASM28671v1_genomic.fna
4149	Propionibacterium acnes	GCA_000240035.1_ASM24003v1_genomic.fna
4150	Mycoplasma bovis	GCA_001611895.1_ASM161189v1_genomic.fna
4151	Campylobacter lari	GCA_001017575.1_ASM101757v1_genomic.fna
4152	Proteus mirabilis	GCA_002180235.1_ASM218023v1_genomic.fna
4153	Klebsiella oxytoca	GCA_001970835.1_ASM197083v1_genomic.fna
4154	Campylobacter coli	GCA_001417635.1_ASM141763v1_genomic.fna
4155	Lactobacillus salivarius	GCA_900094615.1_ASM90009461v1_genomic.fna
4156	Enterobacter cloacae	GCA_000770155.1_ASM77015v1_genomic.fna
4157	Listeria ivanovii	GCA_900187195.1_51485_F01_genomic.fna
4158	Bifidobacterium breve	GCA_000569035.1_ASM56903v1_genomic.fna
4159	Mycobacterium abscessus	GCA_000770215.1_ASM77021v1_genomic.fna
4160	Paenibacillus polymyxa	GCA_001719045.1_ASM171904v1_genomic.fna
4161	Pseudomonas stutzeri	GCA_000327065.1_ASM32706v1_genomic.fna
4162	Aeromonas hydrophila	GCA_000819505.1_ASM81950v1_genomic.fna
4163	Granulibacter bethesdensis	GCA_001889485.1_ASM188948v1_genomic.fna
4164	Flavobacterium psychrophilum	GCA_000831205.1_ASM83120v1_genomic.fna
4165	Lactobacillus johnsonii	GCA_002176855.1_ASM217685v1_genomic.fna
4166	Bdellovibrio bacteriovorus	GCA_002208115.1_ASM220811v1_genomic.fna
4167	Lactobacillus paracasei	GCA_001514415.1_ASM151441v1_genomic.fna
4168	Klebsiella variicola	GCA_001989535.1_ASM198953v1_genomic.fna
4169	Vibrio alginolyticus	GCA_002119545.1_ASM211954v1_genomic.fna
4170	Corynebacterium pseudotuberculosis	GCA_000227605.2_ASM22760v2_genomic.fna
4171	Riemerella anatipestifer	GCA_001051115.1_ASM105111v1_genomic.fna
4172	Staphylococcus lugdunensis	GCA_002407165.1_ASM240716v1_genomic.fna
4173	Taylorella equigenitalis	GCA_002288245.1_ASM228824v1_genomic.fna
4174	Citrobacter freundii	GCA_002211705.1_ASM221170v1_genomic.fna
4175	Vibrio anguillarum	GCA_002211985.1_ASM221198v1_genomic.fna
4176	Enterobacter hormaechei	GCA_001729705.1_ASM172970v1_genomic.fna
4177	Klebsiella aerogenes	GCA_002591115.1_ASM259111v1_genomic.fna
4178	Corynebacterium ulcerans	GCA_000767685.1_ASM76768v1_genomic.fna
4179	Burkholderia cepacia	GCA_001718835.1_ASM171883v1_genomic.fna

4180	Rickettsia japonica	GCA_002357195.1_ASM235719v1_genomic.fna
4181	Piscirickettsia salmonis	GCA_001932595.1_ASM193259v1_genomic.fna
4182	Francisella noatunensis	GCA_001885275.1_ASM188527v1_genomic.fna
4183	Elizabethkingia anophelis	GCA_001618545.2_ASM161854v2_genomic.fna
4184	Rhizobium phaseoli	GCA_001664225.1_ASM166422v1_genomic.fna
4185	Bacillus velezensis	GCA_000455585.1_ucmb5113v1_genomic.fna
4186	Klebsiella michiganensis	GCA_002290285.1_ASM229028v1_genomic.fna
4187	Mycobacterium chimaera	GCA_002287605.1_ASM228760v1_genomic.fna
4188	Alteromonas mediterranea	GCA_000439595.1_ASM43959v1_genomic.fna
4189	Alteromonas mediterranea	GCA_001562315.1_ASM156231v1_genomic.fna
4190	Mycobacterium chimaera	GCA_900116695.1_ASM90011669v1_genomic.fna
4191	Oblitimonas alkaliphila	GCA_001267235.1_ASM126723v1_genomic.fna
4192	Bacillus velezensis	GCA_000493375.1_BAPNAU_WGS_genomic.fna
4193	Enterobacter cloacae	GCA_002208095.1_ASM220809v1_genomic.fna
4194	Rhizobium phaseoli	GCA_001664245.1_ASM166424v1_genomic.fna
4195	Elizabethkingia anophelis	GCA_002022025.1_ASM202202v1_genomic.fna
4196	Francisella noatunensis	GCA_002214225.1_ASM221422v1_genomic.fna
4197	Piscirickettsia salmonis	GCA_001932735.1_ASM193273v1_genomic.fna
4198	Rickettsia japonica	GCA_002357215.1_ASM235721v1_genomic.fna
4199	Burkholderia cepacia	GCA_002209085.1_ASM220908v1_genomic.fna
4200	Corynebacterium ulcerans	GCA_000769635.1_ASM76963v1_genomic.fna
4201	Enterobacter hormaechei	GCA_001874505.1_ASM187450v1_genomic.fna
4202	Vibrio anguillarum	GCA_002212005.1_ASM221200v1_genomic.fna
4203	Citrobacter freundii	GCA_002215385.1_ASM221538v1_genomic.fna
4204	Staphylococcus lugdunensis	GCA_002591215.1_ASM259121v1_genomic.fna
4205	Riemerella anatipestifer	GCA_001077795.1_ASM107779v1_genomic.fna
4206	Corynebacterium pseudotuberculosis	GCA_000233735.1_ASM23373v1_genomic.fna
4207	Vibrio alginolyticus	GCA_002119565.1_ASM211956v1_genomic.fna
4208	Lactobacillus paracasei	GCA_002079285.1_ASM207928v1_genomic.fna
4209	Flavobacterium psychrophilum	GCA_000831225.1_ASM83122v1_genomic.fna
4210	Granulibacter bethesdensis	GCA_001889505.1_ASM188950v1_genomic.fna
4211	Aeromonas hydrophila	GCA_000940915.1_ASM94091v1_genomic.fna
4212	Pseudomonas stutzeri	GCA_000661915.1_ASM66191v1_genomic.fna
4213	Paenibacillus polymyxa	GCA_001874405.2_ASM187440v2_genomic.fna

4214	Mycobacterium abscessus	GCA_001050395.1_ASM105039v1_genomic.fna
4215	Bifidobacterium breve	GCA_000569055.1_ASM56905v1_genomic.fna
4216	Enterobacter cloacae	GCA_000784865.1_ASM78486v1_genomic.fna
4217	Campylobacter coli	GCA_001483845.1_ASM148384v1_genomic.fna
4218	Klebsiella oxytoca	GCA_002072655.1_ASM207265v1_genomic.fna
4219	Proteus mirabilis	GCA_002197405.1_ASM219740v1_genomic.fna
4220	Mycoplasma bovis	GCA_001611935.1_ASM161193v1_genomic.fna
4221	Propionibacterium acnes	GCA_000240055.1_ASM24005v1_genomic.fna
4222	Mycoplasma gallisepticum	GCA_000286735.1_ASM28673v1_genomic.fna
4223	Serratia marcescens	GCA_001417865.2_ASM141786v2_genomic.fna
4224	Lactobacillus brevis	GCA_002138395.1_ASM213839v1_genomic.fna
4225	Mannheimia haemolytica	GCA_000819525.2_ASM81952v2_genomic.fna
4226	Leuconostoc mesenteroides	GCA_002009375.1_ASM200937v1_genomic.fna
4227	Lactobacillus plantarum	GCA_000931425.1_ASM93142v1_genomic.fna
4228	Dehalococcoides mccartyi	GCA_000341695.1_ASM34169v1_genomic.fna
4229	Yersinia enterocolitica	GCA_001304755.1_ASM130475v1_genomic.fna
4230	Mycoplasma pneumoniae	GCA_000733995.1_ASM73399v1_genomic.fna
4231	Corynebacterium diphtheriae	GCA_000255155.1_ASM25515v1_genomic.fna
4232	Haemophilus ducreyi	GCA_001647835.1_ASM164783v1_genomic.fna
4233	Bordetella pertussis	GCA_001307565.1_ASM130756v1_genomic.fna
4234	Sinorhizobium meliloti	GCA_000747295.1_ASM74729v1_genomic.fna
4235	Brucella melitensis	GCA_001307475.2_ASM130747v2_genomic.fna
4236	Brucella ceti	GCA_000590815.1_ASM59081v1_genomic.fna
4237	Lactobacillus rhamnosus	GCA_001988935.1_ASM198893v1_genomic.fna
4238	Pasteurella multocida	GCA_000973565.1_ASM97356v1_genomic.fna
4239	Acetobacter pasteurianus	GCA_000010945.1_ASM1094v1_genomic.fna
4240	Zymomonas mobilis	GCA_000576165.1_ASM57616v1_genomic.fna
4241	Stenotrophomonas maltophilia	GCA_002025605.1_ASM202560v1_genomic.fna
4242	Enterococcus faecium	GCA_001518735.1_ASM151873v1_genomic.fna
4243	Neisseria gonorrhoeae	GCA_001661115.1_ASM166111v1_genomic.fna
4244	Bacillus amyloliquefaciens	GCA_000508265.1_ASM50826v1_genomic.fna
4245	Bifidobacterium animalis	GCA_000260715.1_ASM26071v1_genomic.fna
4246	Chlamydia psittaci	GCA_000298435.2_ASM29843v2_genomic.fna
4247	Klebsiella pneumoniae	GCA_000294365.1_ASM29436v1_genomic.fna

4248	Brucella suis	GCA_000698325.1_ASM69832v1_genomic.fna
4249	Rhizobium leguminosarum	GCA_002240185.1_ASM224018v1_genomic.fna
4250	Borreliella burgdorferi	GCA_002151505.1_ASM215150v1_genomic.fna
4251	Enterococcus faecalis	GCA_000742975.1_ASM74297v1_genomic.fna
4252	Clostridium botulinum	GCA_000019545.1_ASM1954v1_genomic.fna
4253	Rickettsia prowazekii	GCA_000363905.1_ASM36390v1_genomic.fna
4254	Phaeobacter gallaeciensis	GCA_002393525.1_ASM239352v1_genomic.fna
4255	Burkholderia thailandensis	GCA_000765375.1_ASM76537v1_genomic.fna
4256	Mycoplasma mycoides	GCA_001554055.1_ASM155405v1_genomic.fna
4257	Lactobacillus fermentum	GCA_002192435.1_ASM219243v1_genomic.fna
4258	Vibrio parahaemolyticus	GCA_001433415.1_ASM143341v1_genomic.fna
4259	Rickettsia rickettsii	GCA_000831525.1_ASM83152v1_genomic.fna
4260	Lactobacillus helveticus	GCA_001006025.1_ASM100602v1_genomic.fna
4261	Lactobacillus casei	GCA_000829055.1_ASM82905v1_genomic.fna
4262	Bacillus subtilis	GCA_000321395.1_ASM32139v1_genomic.fna
4263	Coxiella burnetii	GCA_001572745.1_ASM157274v1_genomic.fna
4264	Clostridioides difficile	GCA_002073735.1_ASM207373v1_genomic.fna
4265	Xanthomonas oryzae	GCA_001042745.1_ASM104274v1_genomic.fna
4266	Campylobacter fetus	GCA_000967135.1_CFV_genomic.fna
4267	Xanthomonas citri	GCA_000961215.1_ASM96121v1_genomic.fna
4268	Lactobacillus delbrueckii	GCA_001888945.1_ASM188894v1_genomic.fna
4269	Brucella abortus	GCA_000740195.1_ASM74019v1_genomic.fna
4270	Francisella tularensis	GCA_000017785.1_ASM1778v1_genomic.fna
4271	Yersinia pseudotuberculosis	GCA_000834375.1_ASM83437v1_genomic.fna
4272	Streptococcus salivarius	GCA_002094955.1_ASM209495v1_genomic.fna
4273	Rhodopseudomonas palustris	GCA_000020445.1_ASM2044v1_genomic.fna
4274	Vibrio cholerae	GCA_000250855.1_ASM25085v1_genomic.fna
4275	Ralstonia solanacearum	GCA_001663415.1_ASM166341v1_genomic.fna
4276	Bacillus thuringiensis	GCA_000292705.1_ASM29270v1_genomic.fna
4277	Ehrlichia chaffeensis	GCA_000632965.1_ASM63296v1_genomic.fna
4278	Burkholderia mallei	GCA_000755885.1_ASM75588v1_genomic.fna
4279	Burkholderia pseudomallei	GCA_000511895.1_ASM51189v1_genomic.fna
4280	Burkholderia cenocepacia	GCA_001484665.1_ASM148466v1_genomic.fna
4281	Shigella sonnei	GCA_002224625.1_ASM222462v1_genomic.fna

4282	Chlamydia trachomatis	GCA_000092665.1_ASM9266v1_genomic.fna
4283	Corynebacterium glutamicum	GCA_000742715.1_ASM74271v1_genomic.fna
4284	Bacillus pumilus	GCA_001548215.1_ASM154821v1_genomic.fna
4285	Lactobacillus reuteri	GCA_001618905.1_ASM161890v1_genomic.fna
4286	Streptococcus thermophilus	GCA_000971665.1_ASM97166v1_genomic.fna
4287	Legionella pneumophila	GCA_000306865.1_ASM30686v1_genomic.fna
4288	Bacillus licheniformis	GCA_002074135.1_ASM207413v1_genomic.fna
4289	Pseudomonas aeruginosa	GCA_000284555.1_ASM28455v1_genomic.fna
4290	Acinetobacter baumannii	GCA_000188215.1_ASM18821v1_genomic.fna
4291	Streptococcus suis	GCA_000231865.1_ASM23186v1_genomic.fna
4292	Vibrio vulnificus	GCA_001653775.1_ASM165377v1_genomic.fna
4293	Histophilus somni	GCA_002162275.1_ASM216227v1_genomic.fna
4294	Pseudomonas syringae	GCA_001281365.1_ASM128136v1_genomic.fna
4295	Bifidobacterium longum	GCA_000219455.1_ASM21945v1_genomic.fna
4296	Shigella flexneri	GCA_001021855.1_ASM102185v1_genomic.fna
4297	Streptococcus agalactiae	GCA_000689235.1_GBCO_p1_genomic.fna
4298	Leptospira interrogans	GCA_001293065.1_ASM129306v1_genomic.fna
4299	Fusobacterium nucleatum	GCA_001296165.1_ASM129616v1_genomic.fna
4300	Bacillus anthracis	GCA_000583105.1_ASM58310v1_genomic.fna
4301	Streptococcus pneumoniae	GCA_000019265.1_ASM1926v1_genomic.fna
4302	Streptococcus pyogenes	GCA_000012165.1_ASM1216v1_genomic.fna
4303	Xylella fastidiosa	GCA_001456195.1_ASM145619v1_genomic.fna
4304	Pseudomonas putida	GCA_000226035.3_ASM22603v3_genomic.fna
4305	Neisseria meningitidis	GCA_000191425.1_ASM19142v1_genomic.fna
4306	Buchnera aphidicola	GCA_000225465.1_ASM22546v1_genomic.fna
4307	Chlamydia pneumoniae	GCA_001007065.1_CWL011_genomic.fna
4308	Helicobacter pylori	GCA_000023805.1_ASM2380v1_genomic.fna
4309	Escherichia coli	GCA_000009565.2_ASM956v1_genomic.fna
4310	Haemophilus influenzae	GCA_000465255.1_ASM46525v1_genomic.fna
4311	Mycobacterium tuberculosis	GCA_000154605.2_ASM15460v2_genomic.fna
4312	Mycobacterium bovis	GCA_000934325.3_ASM93432v3_genomic.fna
4313	Mycobacterium avium	GCA_000831285.1_ASM83128v1_genomic.fna
4314	Listeria monocytogenes	GCA_000168595.2_ASM16859v2_genomic.fna
4315	Clostridium perfringens	GCA_002312985.1_ASM231298v1_genomic.fna

4316	Bacillus cereus	GCA_000021785.1_ASM2178v1_genomic.fna
4317	Lactococcus lactis	GCA_000312685.1_ASM31268v1_genomic.fna
4318	Staphylococcus epidermidis	GCA_900086615.1_BPH0662_genomic.fna
4319	Staphylococcus aureus	GCA_000011265.1_ASM1126v1_genomic.fna
4320	Yersinia pestis	GCA_000022805.1_ASM2280v1_genomic.fna
4321	Pseudomonas fluorescens	GCA_000963495.1_ASM96349v1_genomic.fna
4322	Campylobacter jejuni	GCA_000148705.1_ASM14870v1_genomic.fna
4323	Salmonella enterica	GCA_000016045.1_ASM1604v1_genomic.fna
4324	Salmonella enterica	GCA_000018385.1_ASM1838v1_genomic.fna
4325	Campylobacter jejuni	GCA_000171795.2_ASM17179v2_genomic.fna
4326	Pseudomonas fluorescens	GCA_000968415.1_ASM96841v1_genomic.fna
4327	Yersinia pestis	GCA_000022825.1_ASM2282v1_genomic.fna
4328	Staphylococcus aureus	GCA_000011505.1_ASM1150v1_genomic.fna
4329	Lactococcus lactis	GCA_000344575.1_ASM34457v1_genomic.fna
4330	Bacillus cereus	GCA_000022505.1_ASM2250v1_genomic.fna
4331	Clostridium perfringens	GCA_002355795.1_ASM235579v1_genomic.fna
4332	Listeria monocytogenes	GCA_000168635.2_ASM16863v2_genomic.fna
4333	Mycobacterium avium	GCA_000835225.1_ASM83522v1_genomic.fna
4334	Mycobacterium bovis	GCA_000967285.1_ASM96728v1_genomic.fna
4335	Haemophilus influenzae	GCA_000698365.1_ASM69836v1_genomic.fna
4336	Mycobacterium tuberculosis	GCA_000193185.2_ASM19318v2_genomic.fna
4337	Escherichia coli	GCA_000010245.1_ASM1024v1_genomic.fna
4338	Helicobacter pylori	GCA_000091345.1_ASM9134v1_genomic.fna
4339	Chlamydia pneumoniae	GCA_001007085.1_Wien3_genomic.fna
4340	Buchnera aphidicola	GCA_001280225.1_ASM128022v1_genomic.fna
4341	Neisseria meningitidis	GCA_000191445.1_ASM19144v1_genomic.fna
4342	Xylella fastidiosa	GCA_001456235.1_ASM145623v1_genomic.fna
4343	Pseudomonas putida	GCA_000264665.1_ASM26466v1_genomic.fna
4344	Streptococcus pyogenes	GCA_000013485.1_ASM1348v1_genomic.fna
4345	Streptococcus pneumoniae	GCA_000019825.1_ASM1982v1_genomic.fna
4346	Fusobacterium nucleatum	GCA_001433955.1_ASM143395v1_genomic.fna
4347	Leptospira interrogans	GCA_002073495.1_ASM207349v1_genomic.fna
4348	Streptococcus agalactiae	GCA_000730215.2_ASM73021v2_genomic.fna
4349	Bacillus anthracis	GCA_000725325.1_ASM72532v1_genomic.fna

4350	Bifidobacterium longum	GCA_000269965.1_ASM26996v1_genomic.fna
4351	Shigella flexneri	GCA_001578125.1_ASM157812v1_genomic.fna
4352	Histophilus somni	GCA_002162295.1_ASM216229v1_genomic.fna
4353	Pseudomonas syringae	GCA_001482725.1_ASM148272v1_genomic.fna
4354	Vibrio vulnificus	GCA_001675245.1_ASM167524v1_genomic.fna
4355	Streptococcus suis	GCA_000231885.1_ASM23188v1_genomic.fna
4356	Acinetobacter baumannii	GCA_000226275.1_ASM22627v1_genomic.fna
4357	Pseudomonas aeruginosa	GCA_000359505.1_ASM35950v1_genomic.fna
4358	Bacillus licheniformis	GCA_002173615.1_ASM217361v1_genomic.fna
4359	Legionella pneumophila	GCA_000347615.1_ASM34761v1_genomic.fna
4360	Shigella sonnei	GCA_002442535.1_ASM244253v1_genomic.fna
4361	Streptococcus thermophilus	GCA_001008015.1_ASM100801v1_genomic.fna
4362	Bacillus pumilus	GCA_001578165.1_ASM157816v1_genomic.fna
4363	Lactobacillus reuteri	GCA_001688685.2_ASM168868v2_genomic.fna
4364	Corynebacterium glutamicum	GCA_000742735.1_ASM74273v1_genomic.fna
4365	Chlamydia trachomatis	GCA_000092685.1_ASM9268v1_genomic.fna
4366	Burkholderia cenocepacia	GCA_001606115.1_ASM160611v1_genomic.fna
4367	Burkholderia pseudomallei	GCA_000511915.1_ASM51191v1_genomic.fna
4368	Bacillus thuringiensis	GCA_000300475.1_ASM30047v1_genomic.fna
4369	Burkholderia mallei	GCA_000756025.2_ASM75602v2_genomic.fna
4370	Ralstonia solanacearum	GCA_001708525.1_ASM170852v1_genomic.fna
4371	Vibrio cholerae	GCA_000765415.1_ASM76541v1_genomic.fna
4372	Rhodopseudomonas palustris	GCA_000177255.2_ASM17725v2_genomic.fna
4373	Streptococcus salivarius	GCA_002094975.1_ASM209497v1_genomic.fna
4374	Yersinia pseudotuberculosis	GCA_000834415.1_ASM83441v1_genomic.fna
4375	Francisella tularensis	GCA_000018925.1_ASM1892v1_genomic.fna
4376	Brucella abortus	GCA_000740215.1_ASM74021v1_genomic.fna
4377	Lactobacillus delbrueckii	GCA_001888965.1_ASM188896v1_genomic.fna
4378	Xanthomonas citri	GCA_000961235.1_ASM96123v1_genomic.fna
4379	Campylobacter fetus	GCA_001484645.1_ASM148464v1_genomic.fna
4380	Xanthomonas oryzae	GCA_001042775.1_ASM104277v1_genomic.fna
4381	Clostridioides difficile	GCA_002080065.1_ASM208006v1_genomic.fna
4382	Coxiella burnetii	GCA_002094935.1_ASM209493v1_genomic.fna
4383	Bacillus subtilis	GCA_000338735.1_ASM33873v1_genomic.fna

4384	Lactobacillus casei	GCA_002192215.1_ASM219221v1_genomic.fna
4385	Delftia acidovorans	GCA_001969245.1_ASM196924v1_genomic.fna
4386	Lactobacillus helveticus	GCA_001702095.1_ASM170209v1_genomic.fna
4387	Rickettsia rickettsii	GCA_000831545.1_ASM83154v1_genomic.fna
4388	Vibrio parahaemolyticus	GCA_001558495.1_ASM155849v1_genomic.fna
4389	Lactobacillus fermentum	GCA_002356135.1_ASM235613v1_genomic.fna
4390	Mycoplasma mycoides	GCA_001554075.1_ASM155407v1_genomic.fna
4391	Burkholderia thailandensis	GCA_000808035.2_ASM80803v2_genomic.fna
4392	Rickettsia prowazekii	GCA_000367405.1_ASM36740v1_genomic.fna
4393	Clostridium botulinum	GCA_000020285.1_ASM2028v1_genomic.fna
4394	Borreliella burgdorferi	GCA_002442595.1_ASM244259v1_genomic.fna
4395	Rhizobium leguminosarum	GCA_002243365.1_ASM224336v1_genomic.fna
4396	Brucella suis	GCA_000698345.1_ASM69834v1_genomic.fna
4397	Enterococcus faecalis	GCA_001598635.1_ASM159863v1_genomic.fna
4398	Klebsiella pneumoniae	GCA_000364385.3_ASM36438v3_genomic.fna
4399	Chlamydia psittaci	GCA_000298455.2_ASM29845v2_genomic.fna
4400	Bifidobacterium animalis	GCA_000277325.1_ASM27732v1_genomic.fna
4401	Bacillus amyloliquefaciens	GCA_000833005.1_ASM83300v1_genomic.fna
4402	Neisseria gonorrhoeae	GCA_001661135.1_ASM166113v1_genomic.fna
4403	Enterococcus faecium	GCA_001587115.1_ASM158711v1_genomic.fna
4404	Stenotrophomonas maltophilia	GCA_002138415.1_ASM213841v1_genomic.fna
4405	Zymomonas mobilis	GCA_002158845.1_ASM215884v1_genomic.fna
4406	Acetobacter pasteurianus	GCA_000010965.1_ASM1096v1_genomic.fna
4407	Pasteurella multocida	GCA_001578435.2_ASM157843v2_genomic.fna
4408	Lactobacillus rhamnosus	GCA_002076955.1_ASM207695v1_genomic.fna
4409	Brucella melitensis	GCA_001431745.1_ASM143174v1_genomic.fna
4410	Sinorhizobium meliloti	GCA_001050915.2_ASM105091v2_genomic.fna
4411	Bordetella pertussis	GCA_001307585.1_ASM130758v1_genomic.fna
4412	Haemophilus ducreyi	GCA_001647855.1_ASM164785v1_genomic.fna
4413	Corynebacterium diphtheriae	GCA_000255175.1_ASM25517v1_genomic.fna
4414	Mycoplasma pneumoniae	GCA_001272715.1_ASM127271v1_genomic.fna
4415	Yersinia enterocolitica	GCA_001305635.1_ASM130563v1_genomic.fna
4416	Dehalococcoides mccartyi	GCA_000499365.1_ASM49936v1_genomic.fna
4417	Leuconostoc mesenteroides	GCA_002117185.1_ASM211718v1_genomic.fna

4418	Mannheimia haemolytica	GCA_000963635.1_ASM96363v1_genomic.fna
4419	Lactobacillus plantarum	GCA_001272315.2_ASM127231v2_genomic.fna
4420	Lactobacillus brevis	GCA_002173555.1_ASM217355v1_genomic.fna
4421	Serratia marcescens	GCA_001672055.1_ASM167205v1_genomic.fna
4422	Mycoplasma gallisepticum	GCA_000286755.1_ASM28675v1_genomic.fna
4423	Propionibacterium acnes	GCA_000302515.1_ASM30251v1_genomic.fna
4424	Mycoplasma bovis	GCA_002009275.1_ASM200927v1_genomic.fna
4425	Campylobacter coli	GCA_001639125.1_ASM163912v1_genomic.fna
4426	Proteus mirabilis	GCA_002310875.1_ASM231087v1_genomic.fna
4427	Citrobacter koseri	GCA_001931885.1_ASM193188v1_genomic.fna
4428	Enterobacter cloacae	GCA_000784905.1_ASM78490v1_genomic.fna
4429	Bifidobacterium breve	GCA_000569075.1_ASM56907v1_genomic.fna
4430	Mycobacterium abscessus	GCA_001430775.1_ASM143077v1_genomic.fna
4431	Paenibacillus polymyxa	GCA_001874425.2_ASM187442v2_genomic.fna
4432	Pseudomonas stutzeri	GCA_001038645.1_ASM103864v1_genomic.fna
4433	Aeromonas hydrophila	GCA_000963645.1_ASM96364v1_genomic.fna
4434	Granulibacter bethesdensis	GCA_001889525.1_ASM188952v1_genomic.fna
4435	Bacteroides dorei	GCA_000738045.1_ASM73804v1_genomic.fna
4436	Lactobacillus paracasei	GCA_002257625.1_ASM225762v1_genomic.fna
4437	Vibrio alginolyticus	GCA_002119585.1_ASM211958v1_genomic.fna
4438	Corynebacterium pseudotuberculosis	GCA_000241855.1_ASM24185v1_genomic.fna
4439	Riemerella anatipestifer	GCA_002025185.1_ASM202518v1_genomic.fna
4440	Vibrio anguillarum	GCA_002212025.1_ASM221202v1_genomic.fna
4441	Corynebacterium ulcerans	GCA_000968945.1_ASM96894v1_genomic.fna
4442	Rickettsia japonica	GCA_002357235.1_ASM235723v1_genomic.fna
4443	Piscirickettsia salmonis	GCA_001932755.1_ASM193275v1_genomic.fna
4444	Elizabethkingia anophelis	GCA_002022065.1_ASM202206v1_genomic.fna
4445	Rhizobium phaseoli	GCA_001664285.1_ASM166428v1_genomic.fna
4446	Bacillus velezensis	GCA_000583065.1_ASM58306v1_genomic.fna
4447	Alteromonas mediterranea	GCA_001886395.1_ASM188639v1_genomic.fna
4448	Alteromonas mediterranea	GCA_001886415.1_ASM188641v1_genomic.fna
4449	Bacillus velezensis	GCA_000685725.1_ASM68572v1_genomic.fna
4450	Clostridium tyrobutyricum	GCA_001679705.1_ASM167970v1_genomic.fna
4451	Rhizobium phaseoli	GCA_001664365.1_ASM166436v1_genomic.fna

4452	Elizabethkingia anophelis	GCA_002023665.2_ASM202366v2_genomic.fna
4453	Piscirickettsia salmonis	GCA_001932775.1_ASM193277v1_genomic.fna
4454	Rickettsia japonica	GCA_002357255.1_ASM235725v1_genomic.fna
4455	Corynebacterium ulcerans	GCA_001281445.1_ASM128144v1_genomic.fna
4456	Vibrio anguillarum	GCA_002287545.1_ASM228754v1_genomic.fna
4457	Riemerella anatipestifer	GCA_900186945.1_48903_E01_genomic.fna
4458	Corynebacterium pseudotuberculosis	GCA_000248375.1_ASM24837v1_genomic.fna
4459	Vibrio alginolyticus	GCA_002149065.1_ASM214906v1_genomic.fna
4460	Lactobacillus paracasei	GCA_002442835.1_ASM244283v1_genomic.fna
4461	Bacteroides dorei	GCA_000738065.1_ASM73806v1_genomic.fna
4462	Granulibacter bethesdensis	GCA_001889545.1_ASM188954v1_genomic.fna
4463	Streptococcus iniae	GCA_000403625.1_ASM40362v1_genomic.fna
4464	Aeromonas hydrophila	GCA_001019645.1_ASM101964v1_genomic.fna
4465	Pseudomonas stutzeri	GCA_001648195.1_ASM164819v1_genomic.fna
4466	Paenibacillus polymyxa	GCA_001922145.1_ASM192214v1_genomic.fna
4467	Mycobacterium abscessus	GCA_001606195.1_ASM160619v1_genomic.fna
4468	Bifidobacterium breve	GCA_001281425.1_ASM128142v1_genomic.fna
4469	Enterobacter cloacae	GCA_001022015.1_ASM102201v1_genomic.fna
4470	Citrobacter koseri	GCA_002208985.1_ASM220898v1_genomic.fna
4471	Proteus mirabilis	GCA_002310895.1_ASM231089v1_genomic.fna
4472	Campylobacter coli	GCA_001717605.1_ASM171760v1_genomic.fna
4473	Propionibacterium acnes	GCA_000376705.1_ASM37670v1_genomic.fna
4474	Mycoplasma gallisepticum	GCA_000286775.1_ASM28677v1_genomic.fna
4475	Serratia marcescens	GCA_002220515.1_ASM222051v1_genomic.fna
4476	Lactobacillus brevis	GCA_002174235.1_ASM217423v1_genomic.fna
4477	Mannheimia haemolytica	GCA_000963675.1_ASM96367v1_genomic.fna
4478	Leuconostoc mesenteroides	GCA_002148235.1_ASM214823v1_genomic.fna
4479	Lactobacillus plantarum	GCA_001278015.1_ASM127801v1_genomic.fna
4480	Dehalococcoides mccartyi	GCA_000830905.1_ASM83090v1_genomic.fna
4481	Yersinia enterocolitica	GCA_001708555.1_ASM170855v1_genomic.fna
4482	Mycoplasma pneumoniae	GCA_001272735.1_ASM127273v1_genomic.fna
4483	Corynebacterium diphtheriae	GCA_000255195.1_ASM25519v1_genomic.fna
4484	Haemophilus ducreyi	GCA_001647875.1_ASM164787v1_genomic.fna
4485	Bordetella pertussis	GCA_001307605.1_ASM130760v1_genomic.fna

4486	Sinorhizobium meliloti	GCA_002197025.1_ASM219702v1_genomic.fna
4487	Brucella melitensis	GCA_001715485.1_ASM171548v1_genomic.fna
4488	Lactobacillus rhamnosus	GCA_002158925.1_ASM215892v1_genomic.fna
4489	Pasteurella multocida	GCA_001663095.1_ASM166309v1_genomic.fna
4490	Acetobacter pasteurianus	GCA_000241585.3_ASM24158v3_genomic.fna
4491	Stenotrophomonas maltophilia	GCA_002208885.1_ASM220888v1_genomic.fna
4492	Enterococcus faecium	GCA_001635875.1_ASM163587v1_genomic.fna
4493	Neisseria gonorrhoeae	GCA_002073535.1_ASM207353v1_genomic.fna
4494	Bacillus amyloliquefaciens	GCA_000835145.1_ASM83514v1_genomic.fna
4495	Bifidobacterium animalis	GCA_000277345.1_ASM27734v1_genomic.fna
4496	Chlamydia psittaci	GCA_000298475.2_ASM29847v2_genomic.fna
4497	Klebsiella pneumoniae	GCA_000406765.2_ASM40676v2_genomic.fna
4498	Enterococcus faecalis	GCA_001689055.2_ASM168905v2_genomic.fna
4499	Brucella suis	GCA_000699025.1_ASM69902v1_genomic.fna
4500	Clostridium botulinum	GCA_000020345.1_ASM2034v1_genomic.fna
4501	Rickettsia prowazekii	GCA_001602215.1_ASM160221v1_genomic.fna
4502	Burkholderia thailandensis	GCA_000959425.1_ASM95942v1_genomic.fna
4503	Mycoplasma mycoides	GCA_001554155.1_ASM155415v1_genomic.fna
4504	Vibrio parahaemolyticus	GCA_001636035.1_ASM163603v1_genomic.fna
4505	Rickettsia rickettsii	GCA_001950995.1_ASM195099v1_genomic.fna
4506	Lactobacillus helveticus	GCA_001746265.2_ASM174626v2_genomic.fna
4507	Bacillus subtilis	GCA_000344745.1_ASM34474v1_genomic.fna
4508	Coxiella burnetii	GCA_002109345.1_ASM210934v1_genomic.fna
4509	Clostridioides difficile	GCA_002234355.1_ASM223435v1_genomic.fna
4510	Xanthomonas oryzae	GCA_001042795.1_ASM104279v1_genomic.fna
4511	Campylobacter fetus	GCA_001686885.1_ASM168688v1_genomic.fna
4512	Xanthomonas citri	GCA_000961255.1_ASM96125v1_genomic.fna
4513	Lactobacillus delbrueckii	GCA_001888985.1_ASM188898v1_genomic.fna
4514	Brucella abortus	GCA_000740295.1_ASM74029v1_genomic.fna
4515	Yersinia pseudotuberculosis	GCA_000834435.1_ASM83443v1_genomic.fna
4516	Francisella tularensis	GCA_000023305.1_ASM2330v1_genomic.fna
4517	Vibrio cholerae	GCA_000788715.2_ASM78871v2_genomic.fna
4518	Ralstonia solanacearum	GCA_001879565.1_ASM187956v1_genomic.fna
4519	Bacillus thuringiensis	GCA_000306745.1_ASM30674v1_genomic.fna

4520	Burkholderia mallei	GCA_000762285.1_ASM76228v1_genomic.fna
4521	Burkholderia pseudomallei	GCA_000520895.1_ASM52089v1_genomic.fna
4522	Burkholderia cenocepacia	GCA_001606135.1_ASM160613v1_genomic.fna
4523	Chlamydia trachomatis	GCA_000092705.1_ASM9270v1_genomic.fna
4524	Corynebacterium glutamicum	GCA_000828015.1_ASM82801v1_genomic.fna
4525	Bacillus pumilus	GCA_001700735.1_ASM170073v1_genomic.fna
4526	Streptococcus thermophilus	GCA_001280285.1_ASM128028v1_genomic.fna
4527	Bacillus licheniformis	GCA_002173675.1_ASM217367v1_genomic.fna
4528	Legionella pneumophila	GCA_001590615.1_ASM159061v1_genomic.fna
4529	Pseudomonas aeruginosa	GCA_000414035.1_ASM41403v1_genomic.fna
4530	Acinetobacter baumannii	GCA_000302575.1_ASM30257v1_genomic.fna
4531	Streptococcus suis	GCA_000231905.1_ASM23190v1_genomic.fna
4532	Vibrio vulnificus	GCA_002117205.1_ASM211720v1_genomic.fna
4533	Bifidobacterium longum	GCA_000730205.1_ASM73020v1_genomic.fna
4534	Bacillus anthracis	GCA_000742655.1_ASM74265v1_genomic.fna
4535	Shigella flexneri	GCA_001580175.1_ASM158017v1_genomic.fna
4536	Streptococcus agalactiae	GCA_000730255.1_ASM73025v1_genomic.fna
4537	Pseudomonas syringae	GCA_001913215.1_ASM191321v1_genomic.fna
4538	Fusobacterium nucleatum	GCA_001457555.1_NCTC10562_genomic.fna
4539	Streptococcus pneumoniae	GCA_000019985.1_ASM1998v1_genomic.fna
4540	Streptococcus pyogenes	GCA_000013505.1_ASM1350v1_genomic.fna
4541	Xylella fastidiosa	GCA_001456275.1_ASM145627v1_genomic.fna
4542	Pseudomonas putida	GCA_000271965.2_ASM27196v2_genomic.fna
4543	Neisseria meningitidis	GCA_000191465.1_ASM19146v1_genomic.fna
4544	Buchnera aphidicola	GCA_900016785.1_BTsPazieg1.0_genomic.fna
4545	Chlamydia pneumoniae	GCA_001007105.1_CV14_genomic.fna
4546	Helicobacter pylori	GCA_000093185.1_ASM9318v1_genomic.fna
4547	Escherichia coli	GCA_000010385.1_ASM1038v1_genomic.fna
4548	Haemophilus influenzae	GCA_000767075.1_ASM76707v1_genomic.fna
4549	Prochlorococcus marinus	GCA_000015685.1_ASM1568v1_genomic.fna
4550	Mycobacterium tuberculosis	GCA_000224435.1_ASM22443v1_genomic.fna
4551	Mycobacterium bovis	GCA_001043255.1_ASM104325v1_genomic.fna
4552	Mycobacterium avium	GCA_000835265.1_ASM83526v1_genomic.fna
4553	Listeria monocytogenes	GCA_000168695.2_ASM16869v2_genomic.fna

4554	Lactococcus lactis	GCA_000468955.1_ASM46895v1_genomic.fna
4555	Bacillus cereus	GCA_000143605.1_ASM14360v1_genomic.fna
4556	Staphylococcus aureus	GCA_000011525.1_ASM1152v1_genomic.fna
4557	Yersinia pestis	GCA_000022845.1_ASM2284v1_genomic.fna
4558	Pseudomonas fluorescens	GCA_001307155.1_ASM130715v1_genomic.fna
4559	Campylobacter jejuni	GCA_000184205.1_ASM18420v1_genomic.fna
4560	Salmonella enterica	GCA_000018625.1_ASM1862v1_genomic.fna
4561	Salmonella enterica	GCA_000018705.1_ASM1870v1_genomic.fna
4562	Pseudomonas fluorescens	GCA_001307275.1_ASM130727v1_genomic.fna
4563	Campylobacter jejuni	GCA_000302555.4_ASM30255v4_genomic.fna
4564	Yersinia pestis	GCA_000186725.1_ASM18672v1_genomic.fna
4565	Staphylococcus aureus	GCA_000012045.1_ASM1204v1_genomic.fna
4566	Bacillus cereus	GCA_000239195.1_ASM23919v1_genomic.fna
4567	Lactococcus lactis	GCA_000478255.2_ASM47825v2_genomic.fna
4568	Mycobacterium avium	GCA_001653355.1_ASM165335v1_genomic.fna
4569	Mycobacterium bovis	GCA_001078615.1_ASM107861v1_genomic.fna
4570	Mycobacterium tuberculosis	GCA_000270365.1_ASM27036v1_genomic.fna
4571	Prochlorococcus marinus	GCA_000015965.1_ASM1596v1_genomic.fna
4572	Haemophilus influenzae	GCA_000931575.1_ASM93157v1_genomic.fna
4573	Escherichia coli	GCA_000010485.1_ASM1048v1_genomic.fna
4574	Helicobacter pylori	GCA_000148665.1_ASM14866v1_genomic.fna
4575	Chlamydia pneumoniae	GCA_001007125.1_CM1_genomic.fna
4576	Buchnera aphidicola	GCA_001648115.1_ASM164811v1_genomic.fna
4577	Neisseria meningitidis	GCA_000191485.1_ASM19148v1_genomic.fna
4578	Xylella fastidiosa	GCA_001456295.1_ASM145629v1_genomic.fna
4579	Pseudomonas putida	GCA_000325725.1_ASM32572v1_genomic.fna
4580	Streptococcus pyogenes	GCA_000013525.1_ASM1352v1_genomic.fna
4581	Streptococcus pneumoniae	GCA_000026665.1_ASM2666v1_genomic.fna
4582	Fusobacterium nucleatum	GCA_002211605.1_ASM221160v1_genomic.fna
4583	Pseudomonas syringae	GCA_001913235.1_ASM191323v1_genomic.fna
4584	Streptococcus agalactiae	GCA_000782855.1_ASM78285v1_genomic.fna
4585	Bacillus anthracis	GCA_000742895.1_ASM74289v1_genomic.fna
4586	Shigella flexneri	GCA_002240075.1_ASM224007v1_genomic.fna
4587	Bifidobacterium longum	GCA_000772485.1_ASM77248v1_genomic.fna

4588	Vibrio vulnificus	GCA_002204915.1_ASM220491v1_genomic.fna
4589	Streptococcus suis	GCA_000231925.1_ASM23192v1_genomic.fna
4590	Pseudomonas aeruginosa	GCA_000473745.3_ASM47374v3_genomic.fna
4591	Legionella pneumophila	GCA_001590645.1_ASM159064v1_genomic.fna
4592	Bacillus licheniformis	GCA_002174255.1_ASM217425v1_genomic.fna
4593	Acinetobacter baumannii	GCA_000419385.1_ASM41938v1_genomic.fna
4594	Streptococcus thermophilus	GCA_001514435.1_ASM151443v1_genomic.fna
4595	Bacillus pumilus	GCA_001704975.1_ASM170497v1_genomic.fna
4596	Chlamydia trachomatis	GCA_000092725.1_ASM9272v1_genomic.fna
4597	Corynebacterium glutamicum	GCA_001447865.2_ASM144786v2_genomic.fna
4598	Burkholderia cenocepacia	GCA_001718895.1_ASM171889v1_genomic.fna
4599	Burkholderia pseudomallei	GCA_000521645.1_ASM52164v1_genomic.fna
4600	Bacillus thuringiensis	GCA_000338755.1_ASM33875v1_genomic.fna
4601	Burkholderia mallei	GCA_000959165.1_ASM95916v1_genomic.fna
4602	Ralstonia solanacearum	GCA_001887535.1_ASM188753v1_genomic.fna
4603	Vibrio cholerae	GCA_000829215.1_ASM82921v1_genomic.fna
4604	Francisella tularensis	GCA_000168775.2_ASM16877v2_genomic.fna
4605	Yersinia pseudotuberculosis	GCA_000834475.1_ASM83447v1_genomic.fna
4606	Brucella abortus	GCA_000740315.1_ASM74031v1_genomic.fna
4607	Lactobacillus delbrueckii	GCA_001908415.1_ASM190841v1_genomic.fna
4608	Xanthomonas citri	GCA_000961275.1_ASM96127v1_genomic.fna
4609	Xanthomonas oryzae	GCA_001042815.1_ASM104281v1_genomic.fna
4610	Bacillus subtilis	GCA_000349795.1_ASM34979v1_genomic.fna
4611	Rickettsia rickettsii	GCA_001951015.1_ASM195101v1_genomic.fna
4612	Vibrio parahaemolyticus	GCA_001682175.1_ASM168217v1_genomic.fna
4613	Ureaplasma parvum	GCA_002205575.1_ASM220557v1_genomic.fna
4614	Mycoplasma mycoides	GCA_001564435.1_ASM156443v1_genomic.fna
4615	Burkholderia thailandensis	GCA_000959605.1_ASM95960v1_genomic.fna
4616	Clostridium botulinum	GCA_000022765.1_ASM2276v1_genomic.fna
4617	Brucella suis	GCA_000740235.1_ASM74023v1_genomic.fna
4618	Enterococcus faecalis	GCA_001878735.1_ASM187873v1_genomic.fna
4619	Klebsiella pneumoniae	GCA_000417085.2_ASM41708v2_genomic.fna
4620	Chlamydia psittaci	GCA_000298495.2_ASM29849v2_genomic.fna
4621	Bifidobacterium animalis	GCA_000414215.1_ASM41421v1_genomic.fna

4622	Bacillus amyloliquefaciens	GCA_000973485.1_ASM97348v1_genomic.fna
4623	Neisseria gonorrhoeae	GCA_002073615.1_ASM207361v1_genomic.fna
4624	Enterococcus faecium	GCA_001720945.1_ASM172094v1_genomic.fna
4625	Stenotrophomonas maltophilia	GCA_900186865.1_49243_F02_genomic.fna
4626	Acetobacter pasteurianus	GCA_000723785.1_AP1_genomic.fna
4627	Pasteurella multocida	GCA_001721885.1_ASM172188v1_genomic.fna
4628	Lactobacillus rhamnosus	GCA_002286235.1_ASM228623v1_genomic.fna
4629	Brucella melitensis	GCA_002191235.1_ASM219123v1_genomic.fna
4630	Sinorhizobium meliloti	GCA_002197045.1_ASM219704v1_genomic.fna
4631	Bordetella pertussis	GCA_001307625.1_ASM130762v1_genomic.fna
4632	Haemophilus ducreyi	GCA_001647895.1_ASM164789v1_genomic.fna
4633	Corynebacterium diphtheriae	GCA_000255215.1_ASM25521v1_genomic.fna
4634	Mycoplasma pneumoniae	GCA_001272755.1_ASM127275v1_genomic.fna
4635	Yersinia enterocolitica	GCA_001708575.1_ASM170857v1_genomic.fna
4636	Dehalococcoides mccartyi	GCA_000830925.1_ASM83092v1_genomic.fna
4637	Lactobacillus plantarum	GCA_001296095.1_ASM129609v1_genomic.fna
4638	Leuconostoc mesenteroides	GCA_002370395.1_ASM237039v1_genomic.fna
4639	Mannheimia haemolytica	GCA_002285575.1_ASM228557v1_genomic.fna
4640	Serratia marcescens	GCA_002220535.1_ASM222053v1_genomic.fna
4641	Mycoplasma gallisepticum	GCA_000286795.1_ASM28679v1_genomic.fna
4642	Propionibacterium acnes	GCA_000709495.1_ASM70949v1_genomic.fna
4643	Campylobacter coli	GCA_001865455.1_ASM186545v1_genomic.fna
4644	Enterobacter cloacae	GCA_001022055.1_ASM102205v1_genomic.fna
4645	Bifidobacterium breve	GCA_001990225.1_ASM199022v1_genomic.fna
4646	Mycobacterium abscessus	GCA_001606215.1_ASM160621v1_genomic.fna
4647	Aeromonas hydrophila	GCA_001455365.1_ASM145536v1_genomic.fna
4648	Streptococcus iniae	GCA_000648525.1_ASM64852v1_genomic.fna
4649	Bacteroides dorei	GCA_001274835.1_ASM127483v1_genomic.fna
4650	Vibrio alginolyticus	GCA_002149085.1_ASM214908v1_genomic.fna
4651	Corynebacterium pseudotuberculosis	GCA_000255935.1_ASM25593v1_genomic.fna
4652	Vibrio anguillarum	GCA_002291265.1_ASM229126v1_genomic.fna
4653	Corynebacterium ulcerans	GCA_002162115.1_ASM216211v1_genomic.fna
4654	Rickettsia japonica	GCA_002357275.1_ASM235727v1_genomic.fna
4655	Piscirickettsia salmonis	GCA_001932795.1_ASM193279v1_genomic.fna

4656	Elizabethkingia anophelis	GCA_002024825.1_ASM202482v1_genomic.fna
4657	Rhizobium phaseoli	GCA_001664385.1_ASM166438v1_genomic.fna
4658	Bacillus velezensis	GCA_000769555.1_ASM76955v1_genomic.fna
4659	Alteromonas mediterranea	GCA_001886435.1_ASM188643v1_genomic.fna
4660	Alteromonas mediterranea	GCA_001886455.1_ASM188645v1_genomic.fna
4661	Bacillus velezensis	GCA_000973585.1_ASM97358v1_genomic.fna
4662	Rhizobium phaseoli	GCA_001664405.1_ASM166440v1_genomic.fna
4663	Elizabethkingia anophelis	GCA_002277995.1_ASM227799v1_genomic.fna
4664	Piscirickettsia salmonis	GCA_001932815.1_ASM193281v1_genomic.fna
4665	Rickettsia japonica	GCA_002357295.1_ASM235729v1_genomic.fna
4666	Corynebacterium ulcerans	GCA_900187135.1_51485_C02_genomic.fna
4667	Vibrio anguillarum	GCA_002310335.1_ASM231033v1_genomic.fna
4668	Corynebacterium pseudotuberculosis	GCA_000258385.1_ASM25838v1_genomic.fna
4669	Vibrio alginolyticus	GCA_002149105.1_ASM214910v1_genomic.fna
4670	Streptococcus iniae	GCA_000648555.1_ASM64855v1_genomic.fna
4671	Aeromonas hydrophila	GCA_001518775.1_ASM151877v1_genomic.fna
4672	Mycobacterium abscessus	GCA_001606235.1_ASM160623v1_genomic.fna
4673	Enterobacter cloacae	GCA_001022075.1_ASM102207v1_genomic.fna
4674	Campylobacter coli	GCA_001865475.1_ASM186547v1_genomic.fna
4675	Cutibacterium acnes	GCA_001281065.1_ASM128106v1_genomic.fna
4676	Mycoplasma gallisepticum	GCA_000286815.1_ASM28681v1_genomic.fna
4677	Serratia marcescens	GCA_002220555.1_ASM222055v1_genomic.fna
4678	Mannheimia haemolytica	GCA_002285595.1_ASM228559v1_genomic.fna
4679	Leuconostoc mesenteroides	GCA_002370415.1_ASM237041v1_genomic.fna
4680	Lactobacillus plantarum	GCA_001302645.1_ASM130264v1_genomic.fna
4681	Dehalococcoides mccartyi	GCA_001547795.1_ASM154779v1_genomic.fna
4682	Yersinia enterocolitica	GCA_001708595.1_ASM170859v1_genomic.fna
4683	Mycoplasma pneumoniae	GCA_001272775.1_ASM127277v1_genomic.fna
4684	Corynebacterium diphtheriae	GCA_000255235.1_ASM25523v1_genomic.fna
4685	Haemophilus ducreyi	GCA_001647915.1_ASM164791v1_genomic.fna
4686	Bordetella pertussis	GCA_001307645.1_ASM130764v1_genomic.fna
4687	Sinorhizobium meliloti	GCA_002197065.1_ASM219706v1_genomic.fna
4688	Brucella melitensis	GCA_002191295.1_ASM219129v1_genomic.fna
4689	Lactobacillus rhamnosus	GCA_002287945.1_ASM228794v1_genomic.fna

4690	Pasteurella multocida	GCA_001874445.1_ASM187444v1_genomic.fna
4691	Acetobacter pasteurianus	GCA_001183745.1_ASM118374v1_genomic.fna
4692	Enterococcus faecium	GCA_001721065.1_ASM172106v1_genomic.fna
4693	Neisseria gonorrhoeae	GCA_002073655.1_ASM207365v1_genomic.fna
4694	Bacillus amyloliquefaciens	GCA_001483885.1_ASM148388v1_genomic.fna
4695	Bifidobacterium animalis	GCA_000471945.1_ASM47194v1_genomic.fna
4696	Chlamydia psittaci	GCA_000298515.2_ASM29851v2_genomic.fna
4697	Klebsiella pneumoniae	GCA_000417225.2_ASM41722v2_genomic.fna
4698	Enterococcus faecalis	GCA_001886675.1_ASM188667v1_genomic.fna
4699	Brucella suis	GCA_000740255.1_ASM74025v1_genomic.fna
4700	Clostridium botulinum	GCA_000092345.1_ASM9234v1_genomic.fna
4701	Burkholderia thailandensis	GCA_001718295.1_ASM171829v1_genomic.fna
4702	Vibrio parahaemolyticus	GCA_001700835.1_ASM170083v1_genomic.fna
4703	Ralstonia pickettii	GCA_000471925.1_ASM47192v1_genomic.fna
4704	Bacillus subtilis	GCA_000497485.1_ASM49748v1_genomic.fna
4705	Xanthomonas oryzae	GCA_001042835.1_ASM104283v1_genomic.fna
4706	Xanthomonas citri	GCA_000961295.1_ASM96129v1_genomic.fna
4707	Lactobacillus delbrueckii	GCA_001953135.1_ASM195313v1_genomic.fna
4708	Brucella abortus	GCA_000740375.1_ASM74037v1_genomic.fna
4709	Yersinia pseudotuberculosis	GCA_000834945.1_ASM83494v1_genomic.fna
4710	Francisella cf.	GCA_000195535.1_ASM19553v1_genomic.fna
4711	Vibrio cholerae	GCA_000963555.1_ASM96355v1_genomic.fna
4712	Ralstonia solanacearum	GCA_001891105.1_ASM189110v1_genomic.fna
4713	Burkholderia mallei	GCA_000959405.1_ASM95940v1_genomic.fna
4714	Bacillus thuringiensis	GCA_000341665.1_ASM34166v1_genomic.fna
4715	Burkholderia pseudomallei	GCA_000583835.1_ASM58383v1_genomic.fna
4716	Burkholderia cenocepacia	GCA_001999785.1_ASM199978v1_genomic.fna
4717	Chlamydia trachomatis	GCA_000092745.1_ASM9274v1_genomic.fna
4718	Bacillus pumilus	GCA_001908475.1_ASM190847v1_genomic.fna
4719	Corynebacterium glutamicum	GCA_001518935.2_ASM151893v2_genomic.fna
4720	Streptococcus thermophilus	GCA_001663795.1_ASM166379v1_genomic.fna
4721	Acinetobacter baumannii	GCA_000419405.1_ASM41940v1_genomic.fna
4722	Bacillus licheniformis	GCA_002236895.1_ASM223689v1_genomic.fna
4723	Legionella pneumophila	GCA_001590695.1_ASM159069v1_genomic.fna

4724	Pseudomonas aeruginosa	GCA_000496455.2_ASM49645v2_genomic.fna
4725	Streptococcus suis	GCA_000233575.1_ASM23357v1_genomic.fna
4726	Vibrio vulnificus	GCA_002224265.1_ASM222426v1_genomic.fna
4727	Bifidobacterium longum	GCA_000829295.1_ASM82929v1_genomic.fna
4728	Shigella flexneri	GCA_002240095.1_ASM224009v1_genomic.fna
4729	Pseudomonas syringae	GCA_002024285.1_ASM202428v1_genomic.fna
4730	Bacillus anthracis	GCA_000830095.1_ASM83009v1_genomic.fna
4731	Streptococcus agalactiae	GCA_000831105.1_ASM83110v1_genomic.fna
4732	Fusobacterium nucleatum	GCA_002211625.1_ASM221162v1_genomic.fna
4733	Streptococcus pyogenes	GCA_000013545.1_ASM1354v1_genomic.fna
4734	Streptococcus pneumoniae	GCA_000146975.1_ASM14697v1_genomic.fna
4735	Pseudomonas putida	GCA_000410575.1_ASM41057v1_genomic.fna
4736	Xylella fastidiosa	GCA_001456315.1_ASM145631v1_genomic.fna
4737	Neisseria meningitidis	GCA_000191505.1_ASM19150v1_genomic.fna
4738	Buchnera aphidicola	GCA_001700895.1_ASM170089v1_genomic.fna
4739	Chlamydia pneumoniae	GCA_001007145.1_PB2_genomic.fna
4740	Helicobacter pylori	GCA_000148855.1_ASM14885v1_genomic.fna
4741	Escherichia coli	GCA_000010745.1_ASM1074v1_genomic.fna
4742	Haemophilus influenzae	GCA_000931605.1_ASM93160v1_genomic.fna
4743	Prochlorococcus marinus	GCA_000018065.1_ASM1806v1_genomic.fna
4744	Mycobacterium tuberculosis	GCA_000277735.2_ASM27773v2_genomic.fna
4745	Mycobacterium bovis	GCA_001274555.1_ASM127455v1_genomic.fna
4746	Mycobacterium avium	GCA_001683455.1_ASM168345v1_genomic.fna
4747	Listeria monocytogenes	GCA_000195435.4_ASM19543v4_genomic.fna
4748	Lactococcus lactis	GCA_000479375.2_ASM47937v2_genomic.fna
4749	Bacillus cereus	GCA_000283675.1_ASM28367v1_genomic.fna
4750	Staphylococcus aureus	GCA_000013465.1_ASM1346v1_genomic.fna
4751	Yersinia pestis	GCA_000222975.1_ASM22297v1_genomic.fna
4752	Pseudomonas fluorescens	GCA_001612705.1_ASM161270v1_genomic.fna
4753	Campylobacter jejuni	GCA_000304375.1_AINO_genomic.fna
4754	Salmonella enterica	GCA_000020705.1_ASM2070v1_genomic.fna
4755	Salmonella enterica	GCA_000020745.1_ASM2074v1_genomic.fna
4756	Pseudomonas fluorescens	GCA_001708445.1_ASM170844v1_genomic.fna
4757	Campylobacter jejuni	GCA_000430385.1_ASM43038v1_genomic.fna

4758	Yersinia pestis	GCA_000834235.1_ASM83423v1_genomic.fna
4759	Staphylococcus aureus	GCA_000016805.1_ASM1680v1_genomic.fna
4760	Bacillus cereus	GCA_000292415.1_ASM29241v1_genomic.fna
4761	Lactococcus lactis	GCA_000761115.1_ASM76111v1_genomic.fna
4762	Listeria monocytogenes	GCA_000197755.2_ASM19775v2_genomic.fna
4763	Mycobacterium avium	GCA_001865635.1_ASM186563v1_genomic.fna
4764	Mycobacterium bovis	GCA_001483905.1_ASM148390v1_genomic.fna
4765	Mycobacterium tuberculosis	GCA_000331445.1_ASM33144v1_genomic.fna
4766	Haemophilus influenzae	GCA_000931625.1_ASM93162v1_genomic.fna
4767	Escherichia coli	GCA_000010765.1_ASM1076v1_genomic.fna
4768	Helicobacter pylori	GCA_000148875.1_ASM14887v1_genomic.fna
4769	Buchnera aphidicola	GCA_900128595.1_BCipseudotaxifoliae_v1.0_genomic.fna
4770	Neisseria meningitidis	GCA_000191525.1_ASM19152v1_genomic.fna
4771	Xylella fastidiosa	GCA_001456335.2_ASM145633v2_genomic.fna
4772	Pseudomonas putida	GCA_000412675.1_ASM41267v1_genomic.fna
4773	Streptococcus pneumoniae	GCA_000147095.1_ASM14709v1_genomic.fna
4774	Streptococcus pyogenes	GCA_000018125.1_ASM1812v1_genomic.fna
4775	Streptococcus agalactiae	GCA_000831125.1_ASM83112v1_genomic.fna
4776	Bacillus anthracis	GCA_000832425.1_ASM83242v1_genomic.fna
4777	Pseudomonas syringae	GCA_002024305.1_ASM202430v1_genomic.fna
4778	Bifidobacterium longum	GCA_001051015.2_ASM105101v2_genomic.fna
4779	Shigella flexneri	GCA_002240115.1_ASM224011v1_genomic.fna
4780	Streptococcus suis	GCA_000294495.1_ASM29449v1_genomic.fna
4781	Pseudomonas aeruginosa	GCA_000496605.2_ASM49660v2_genomic.fna
4782	Legionella pneumophila	GCA_001592705.1_ASM159270v1_genomic.fna
4783	Shigella dysenteriae	GCA_000497505.1_ASM49750v1_genomic.fna
4784	Bacillus licheniformis	GCA_002504325.1_ASM250432v1_genomic.fna
4785	Acinetobacter baumannii	GCA_000419425.1_ASM41942v1_genomic.fna
4786	Streptococcus thermophilus	GCA_001685375.1_ASM168537v1_genomic.fna
4787	Corynebacterium glutamicum	GCA_001643035.1_ASM164303v1_genomic.fna
4788	Bacillus pumilus	GCA_900186955.1_49386_E02_genomic.fna
4789	Chlamydia trachomatis	GCA_000092805.1_ASM9280v1_genomic.fna
4790	Burkholderia cenocepacia	GCA_001999825.1_ASM199982v1_genomic.fna
4791	Burkholderia pseudomallei	GCA_000755765.1_ASM75576v1_genomic.fna

4792	Bacillus thuringiensis	GCA_000688795.1_ASM68879v1_genomic.fna
4793	Burkholderia mallei	GCA_000959465.1_ASM95946v1_genomic.fna
4794	Ralstonia solanacearum	GCA_002155245.1_ASM215524v1_genomic.fna
4795	Vibrio cholerae	GCA_000969235.1_ASM96923v1_genomic.fna
4796	Francisella cf.	GCA_000195555.1_ASM19555v1_genomic.fna
4797	Yersinia pseudotuberculosis	GCA_900092345.1_YP4713_genomic.fna
4798	Lactobacillus delbrueckii	GCA_002000885.1_ASM200088v1_genomic.fna
4799	Brucella abortus	GCA_001043295.1_ASM104329v1_genomic.fna
4800	Xanthomonas citri	GCA_000961315.1_ASM96131v1_genomic.fna
4801	Xanthomonas oryzae	GCA_001042855.1_ASM104285v1_genomic.fna
4802	Bacillus subtilis	GCA_000523045.1_ASM52304v1_genomic.fna
4803	Vibrio parahaemolyticus	GCA_001758605.1_ASM175860v1_genomic.fna
4804	Burkholderia thailandensis	GCA_001718595.1_ASM171859v1_genomic.fna
4805	Clostridium botulinum	GCA_000253195.1_ASM25319v1_genomic.fna
4806	Brucella suis	GCA_000740435.1_ASM74043v1_genomic.fna
4807	Enterococcus faecalis	GCA_001989555.1_ASM198955v1_genomic.fna
4808	Klebsiella pneumoniae	GCA_000417265.2_ASM41726v2_genomic.fna
4809	Chlamydia psittaci	GCA_000298535.2_ASM29853v2_genomic.fna
4810	Bacillus amyloliquefaciens	GCA_001586105.1_ASM158610v1_genomic.fna
4811	Bifidobacterium animalis	GCA_000695895.1_ASM69589v1_genomic.fna
4812	Neisseria gonorrhoeae	GCA_900186875.1_49677_F01_genomic.fna
4813	Enterococcus faecium	GCA_001721905.1_ASM172190v1_genomic.fna
4814	Acetobacter pasteurianus	GCA_001766255.1_ASM176625v1_genomic.fna
4815	Pasteurella multocida	GCA_001956795.1_ASM195679v1_genomic.fna
4816	Lactobacillus rhamnosus	GCA_900070175.1_Lactobacillus_rhamnosus_CECT_8800_genomic.fna
4817	Brucella melitensis	GCA_002191335.1_ASM219133v1_genomic.fna
4818	Sinorhizobium meliloti	GCA_002197085.1_ASM219708v1_genomic.fna
4819	Bordetella pertussis	GCA_001307665.1_ASM130766v1_genomic.fna
4820	Haemophilus ducreyi	GCA_002208665.1_ASM220866v1_genomic.fna
4821	Corynebacterium diphtheriae	GCA_000255255.1_ASM25525v1_genomic.fna
4822	Mycoplasma pneumoniae	GCA_001272795.1_ASM127279v1_genomic.fna
4823	Yersinia enterocolitica	GCA_001708615.1_ASM170861v1_genomic.fna
4824	Dehalococcoides mccartyi	GCA_001610775.1_ASM161077v1_genomic.fna
4825	Lactobacillus plantarum	GCA_001484005.1_ASM148400v1_genomic.fna

4826	Mannheimia haemolytica	GCA_002285615.1_ASM228561v1_genomic.fna
4827	Serratia marcescens	GCA_002220575.1_ASM222057v1_genomic.fna
4828	Cutibacterium acnes	GCA_001469565.1_ASM146956v1_genomic.fna
4829	Campylobacter coli	GCA_001865495.1_ASM186549v1_genomic.fna
4830	Enterobacter cloacae	GCA_001022255.1_ASM102225v1_genomic.fna
4831	Mycobacterium abscessus	GCA_001606255.1_ASM160625v1_genomic.fna
4832	Aeromonas hydrophila	GCA_001683535.1_ASM168353v1_genomic.fna
4833	Corynebacterium pseudotuberculosis	GCA_000259155.3_ASM25915v3_genomic.fna
4834	Piscirickettsia salmonis	GCA_001932835.1_ASM193283v1_genomic.fna
4835	Elizabethkingia anophelis	GCA_002310515.1_ASM231051v1_genomic.fna
4836	Rhizobium phaseoli	GCA_001664425.1_ASM166442v1_genomic.fna
4837	Bacillus velezensis	GCA_000987825.1_ASM98782v1_genomic.fna
4838	Alteromonas mediterranea	GCA_000439475.1_ASM43947v1_genomic.fna
4839	Alteromonas mediterranea	GCA_001562295.1_ASM156229v1_genomic.fna
4840	Bacillus velezensis	GCA_000988345.1_ASM98834v1_genomic.fna
4841	Rhizobium phaseoli	GCA_001664445.1_ASM166444v1_genomic.fna
4842	Elizabethkingia anophelis	GCA_002310535.1_ASM231053v1_genomic.fna
4843	Piscirickettsia salmonis	GCA_001932855.1_ASM193285v1_genomic.fna
4844	Corynebacterium pseudotuberculosis	GCA_000263755.2_ASM26375v2_genomic.fna
4845	Streptococcus intermedius	GCA_001296205.1_ASM129620v1_genomic.fna
4846	Aeromonas hydrophila	GCA_001687125.1_ASM168712v1_genomic.fna
4847	Mycobacterium abscessus	GCA_001606275.1_ASM160627v1_genomic.fna
4848	Enterobacter cloacae	GCA_001029645.1_ASM102964v1_genomic.fna
4849	Campylobacter coli	GCA_001865515.1_ASM186551v1_genomic.fna
4850	Cutibacterium acnes	GCA_001469595.1_ASM146959v1_genomic.fna
4851	Serratia marcescens	GCA_002220595.1_ASM222059v1_genomic.fna
4852	Mannheimia haemolytica	GCA_002285815.1_ASM228581v1_genomic.fna
4853	Bartonella quintana	GCA_000294715.1_ASM29471v1_genomic.fna
4854	Lactobacillus plantarum	GCA_001581895.1_ASM158189v1_genomic.fna
4855	Dehalococcoides mccartyi	GCA_001889305.1_ASM188930v1_genomic.fna
4856	Yersinia enterocolitica	GCA_001708635.1_ASM170863v1_genomic.fna
4857	Mycoplasma pneumoniae	GCA_001272815.1_ASM127281v1_genomic.fna
4858	Corynebacterium diphtheriae	GCA_000255275.1_ASM25527v1_genomic.fna
4859	Bordetella pertussis	GCA_001307685.1_ASM130768v1_genomic.fna

4860	Sinorhizobium meliloti	GCA_002197105.1_ASM219710v1_genomic.fna
4861	Brucella melitensis	GCA_002191355.1_ASM219135v1_genomic.fna
4862	Pasteurella multocida	GCA_002059225.1_ASM205922v1_genomic.fna
4863	Acetobacter pasteurianus	GCA_002173735.1_ASM217373v1_genomic.fna
4864	Enterococcus faecium	GCA_001750885.1_ASM175088v1_genomic.fna
4865	Neisseria gonorrhoeae	GCA_900186915.1_49699_A01_genomic.fna
4866	Bifidobacterium animalis	GCA_000816205.1_ASM81620v1_genomic.fna
4867	Bacillus amyloliquefaciens	GCA_001593765.1_ASM159376v1_genomic.fna
4868	Chlamydia psittaci	GCA_000298555.2_ASM29855v2_genomic.fna
4869	Klebsiella pneumoniae	GCA_000445405.1_ASM44540v1_genomic.fna
4870	Enterococcus faecalis	GCA_002163735.1_ASM216373v1_genomic.fna
4871	Brucella suis	GCA_000875695.1_ASM87569v1_genomic.fna
4872	Clostridium botulinum	GCA_000307125.1_ASM30712v1_genomic.fna
4873	Burkholderia thailandensis	GCA_001718615.1_ASM171861v1_genomic.fna
4874	Vibrio parahaemolyticus	GCA_001879585.1_ASM187958v1_genomic.fna
4875	Bacillus subtilis	GCA_000699465.1_ASM69946v1_genomic.fna
4876	Xanthomonas oryzae	GCA_001042875.1_ASM104287v1_genomic.fna
4877	Xanthomonas citri	GCA_000961335.1_ASM96133v1_genomic.fna
4878	Brucella abortus	GCA_001296965.1_ASM129696v1_genomic.fna
4879	Lactobacillus delbrueckii	GCA_002142575.1_ASM214257v1_genomic.fna
4880	Francisella tularensis	GCA_000248415.1_ASM24841v1_genomic.fna
4881	Vibrio cholerae	GCA_000969265.1_ASM96926v1_genomic.fna
4882	Ralstonia solanacearum	GCA_002162015.1_ASM216201v1_genomic.fna
4883	Burkholderia mallei	GCA_000959485.1_ASM95948v1_genomic.fna
4884	Bacillus thuringiensis	GCA_000717535.1_ASM71753v1_genomic.fna
4885	Burkholderia pseudomallei	GCA_000755825.1_ASM75582v1_genomic.fna
4886	Burkholderia cenocepacia	GCA_001999885.1_ASM199988v1_genomic.fna
4887	Chlamydia trachomatis	GCA_000093005.1_ASM9300v1_genomic.fna
4888	Corynebacterium glutamicum	GCA_001683115.1_ASM168311v1_genomic.fna
4889	Streptococcus thermophilus	GCA_001705585.1_ASM170558v1_genomic.fna
4890	Acinetobacter baumannii	GCA_000498375.2_ASM49837v2_genomic.fna
4891	Legionella pneumophila	GCA_001610735.1_ASM161073v1_genomic.fna
4892	Pseudomonas aeruginosa	GCA_000496645.1_ASM49664v1_genomic.fna
4893	Streptococcus suis	GCA_000344765.1_ASM34476v1_genomic.fna

4894	Shigella flexneri	GCA_002240135.1_ASM224013v1_genomic.fna
4895	Bifidobacterium longum	GCA_001281305.1_ASM128130v1_genomic.fna
4896	Bacillus anthracis	GCA_000832445.1_ASM83244v1_genomic.fna
4897	Streptococcus agalactiae	GCA_000831145.1_ASM83114v1_genomic.fna
4898	Streptococcus pneumoniae	GCA_000180515.2_ASM18051v2_genomic.fna
4899	Streptococcus pyogenes	GCA_000230295.1_ASM23029v1_genomic.fna
4900	Xylella fastidiosa	GCA_002117875.1_ASM211787v1_genomic.fna
4901	Pseudomonas putida	GCA_000495455.2_ASM49545v2_genomic.fna
4902	Neisseria meningitidis	GCA_000253215.1_ASM25321v1_genomic.fna
4903	Buchnera aphidicola	GCA_900128735.1_BCiconfinis_v1.0_genomic.fna
4904	Helicobacter pylori	GCA_000148895.1_ASM14889v1_genomic.fna
4905	Escherichia coli	GCA_000013265.1_ASM1326v1_genomic.fna
4906	Haemophilus influenzae	GCA_000968335.1_ASM96833v1_genomic.fna
4907	Mycobacterium tuberculosis	GCA_000350205.1_ASM35020v1_genomic.fna
4908	Mycobacterium bovis	GCA_001580385.1_ASM158038v1_genomic.fna
4909	Mycobacterium avium	GCA_001936215.1_ASM193621v1_genomic.fna
4910	Listeria monocytogenes	GCA_000209755.1_ASM20975v1_genomic.fna
4911	Lactococcus lactis	GCA_000807375.1_ASM80737v1_genomic.fna
4912	Bacillus cereus	GCA_000635895.2_ASM63589v2_genomic.fna
4913	Staphylococcus aureus	GCA_000017085.1_ASM1708v1_genomic.fna
4914	Yersinia pestis	GCA_000834275.1_ASM83427v1_genomic.fna
4915	Pseudomonas fluorescens	GCA_001708465.1_ASM170846v1_genomic.fna
4916	Campylobacter jejuni	GCA_000466065.2_ASM46606v2_genomic.fna
4917	Salmonella enterica	GCA_000020885.1_ASM2088v1_genomic.fna
4918	Salmonella enterica	GCA_000020925.1_ASM2092v1_genomic.fna
4919	Campylobacter jejuni	GCA_000466075.2_ASM46607v2_genomic.fna
4920	Pseudomonas fluorescens	GCA_001708485.1_ASM170848v1_genomic.fna
4921	Yersinia pestis	GCA_000834315.1_ASM83431v1_genomic.fna
4922	Staphylococcus aureus	GCA_000017125.1_ASM1712v1_genomic.fna
4923	Bacillus cereus	GCA_000789315.1_ASM78931v1_genomic.fna
4924	Lactococcus lactis	GCA_002078375.1_ASM207837v1_genomic.fna
4925	Listeria monocytogenes	GCA_000210815.2_ASM21081v2_genomic.fna
4926	Mycobacterium avium	GCA_002208705.1_ASM220870v1_genomic.fna
4927	Mycobacterium tuberculosis	GCA_000364825.1_ASM36482v1_genomic.fna

4928	Haemophilus influenzae	GCA_001457655.1_NCTC8143_genomic.fna
4929	Escherichia coli	GCA_000013305.1_ASM1330v1_genomic.fna
4930	Helicobacter pylori	GCA_000148915.1_ASM14891v1_genomic.fna
4931	Neisseria meningitidis	GCA_000626595.1_ASM62659v1_genomic.fna
4932	Buchnera aphidicola	GCA_900128725.1_BCifornacula_v1.0_genomic.fna
4933	Pseudomonas putida	GCA_000498395.3_ASM49839v3_genomic.fna
4934	Streptococcus pyogenes	GCA_000250905.1_ASM25090v1_genomic.fna
4935	Streptococcus pneumoniae	GCA_000196595.1_ASM19659v1_genomic.fna
4936	Streptococcus agalactiae	GCA_001026925.1_ASM102692v1_genomic.fna
4937	Bacillus anthracis	GCA_000832465.1_ASM83246v1_genomic.fna
4938	Shigella flexneri	GCA_002442995.1_ASM244299v1_genomic.fna
4939	Bifidobacterium longum	GCA_001293145.1_ASM129314v1_genomic.fna
4940	Streptococcus suis	GCA_000390245.1_ASM39024v1_genomic.fna
4941	Acinetobacter baumannii	GCA_000505685.2_ASM50568v2_genomic.fna
4942	Pseudomonas aeruginosa	GCA_000504045.1_ASM50404v1_genomic.fna
4943	Legionella pneumophila	GCA_001652645.1_ASM165264v1_genomic.fna
4944	Streptococcus thermophilus	GCA_001855705.1_ASM185570v1_genomic.fna
4945	Corynebacterium glutamicum	GCA_001687645.1_ASM168764v1_genomic.fna
4946	Chlamydia trachomatis	GCA_000210495.1_ASM21049v1_genomic.fna
4947	Burkholderia cenocepacia	GCA_002007585.1_ASM200758v1_genomic.fna
4948	Burkholderia pseudomallei	GCA_000755905.1_ASM75590v1_genomic.fna
4949	Bacillus thuringiensis	GCA_000747545.1_ASM74754v1_genomic.fna
4950	Burkholderia mallei	GCA_000959625.1_ASM95962v1_genomic.fna
4951	Ralstonia solanacearum	GCA_002220465.1_ASM222046v1_genomic.fna
4952	Vibrio cholerae	GCA_001250235.2_5174_7_8_v2_genomic.fna
4953	Francisella tularensis	GCA_000248435.1_ASM24843v1_genomic.fna
4954	Lactobacillus delbrueckii	GCA_002278095.1_ASM227809v1_genomic.fna
4955	Brucella abortus	GCA_002262975.1_ASM226297v1_genomic.fna
4956	Xanthomonas citri	GCA_000961355.1_ASM96135v1_genomic.fna
4957	Xanthomonas oryzae	GCA_001466505.1_ASM146650v1_genomic.fna
4958	Bacillus subtilis	GCA_000699525.1_ASM69952v1_genomic.fna
4959	Vibrio parahaemolyticus	GCA_001887055.1_ASM188705v1_genomic.fna
4960	Burkholderia thailandensis	GCA_001718635.1_ASM171863v1_genomic.fna
4961	Clostridium botulinum	GCA_000789355.1_ASM78935v1_genomic.fna

4962	Brucella suis	GCA_001715425.1_ASM171542v1_genomic.fna
4963	Enterococcus faecalis	GCA_002208945.1_ASM220894v1_genomic.fna
4964	Klebsiella pneumoniae	GCA_000465975.2_ASM46597v2_genomic.fna
4965	Bacillus amyloliquefaciens	GCA_001593785.1_ASM159378v1_genomic.fna
4966	Bifidobacterium animalis	GCA_000817045.1_ASM81704v1_genomic.fna
4967	Neisseria gonorrhoeae	GCA_900186935.1_49677_G01_genomic.fna
4968	Enterococcus faecium	GCA_001886635.1_ASM188663v1_genomic.fna
4969	Acetobacter pasteurianus	GCA_002173775.1_ASM217377v1_genomic.fna
4970	Pasteurella multocida	GCA_002073255.1_ASM207325v1_genomic.fna
4971	Brucella melitensis	GCA_002191375.1_ASM219137v1_genomic.fna
4972	Sinorhizobium meliloti	GCA_002197125.1_ASM219712v1_genomic.fna
4973	Bordetella pertussis	GCA_001307705.1_ASM130770v1_genomic.fna
4974	Mycoplasma pneumoniae	GCA_001272835.1_ASM127283v1_genomic.fna
4975	Corynebacterium diphtheriae	GCA_001902275.1_ASM190227v1_genomic.fna
4976	Yersinia enterocolitica	GCA_001708655.1_ASM170865v1_genomic.fna
4977	Dehalococcoides mccartyi	GCA_002007825.1_ASM200782v1_genomic.fna
4978	Lactobacillus plantarum	GCA_001596095.1_ASM159609v1_genomic.fna
4979	Serratia marcescens	GCA_002220615.1_ASM222061v1_genomic.fna
4980	Cutibacterium acnes	GCA_001469615.1_ASM146961v1_genomic.fna
4981	Campylobacter coli	GCA_001865535.1_ASM186553v1_genomic.fna
4982	Enterobacter cloacae	GCA_001922365.1_ASM192236v1_genomic.fna
4983	Mycobacterium abscessus	GCA_001606295.1_ASM160629v1_genomic.fna
4984	Corynebacterium pseudotuberculosis	GCA_000265545.2_ASM26554v2_genomic.fna
4985	Piscirickettsia salmonis	GCA_001932875.1_ASM193287v1_genomic.fna
4986	Staphylococcus cohnii	GCA_001990205.1_ASM199020v1_genomic.fna
4987	Bacillus velezensis	GCA_001023595.1_ASM102359v1_genomic.fna
4988	Bacillus velezensis	GCA_001536925.1_BMC_PRJEB12378v1_genomic.fna
4989	Piscirickettsia salmonis	GCA_001932895.1_ASM193289v1_genomic.fna
4990	Corynebacterium pseudotuberculosis	GCA_000730365.1_ASM73036v1_genomic.fna
4991	Lysinibacillus sphaericus	GCA_000017965.1_ASM1796v1_genomic.fna
4992	Mycobacterium abscessus	GCA_001606315.1_ASM160631v1_genomic.fna
4993	Enterobacter cloacae	GCA_002007805.1_ASM200780v1_genomic.fna
4994	Campylobacter coli	GCA_001865555.1_ASM186555v1_genomic.fna
4995	Cutibacterium acnes	GCA_001469655.1_ASM146965v1_genomic.fna

4996	Serratia marcescens	GCA_002220635.1_ASM222063v1_genomic.fna
4997	Lactobacillus plantarum	GCA_001617525.1_ASM161752v1_genomic.fna
4998	Dehalococcoides mccartyi	GCA_002007845.1_ASM200784v1_genomic.fna
4999	Mycoplasma pneumoniae	GCA_001272855.1_ASM127285v1_genomic.fna
5000	Corynebacterium diphtheriae	GCA_002073375.1_ASM207337v1_genomic.fna
5001	Bordetella pertussis	GCA_001307725.1_ASM130772v1_genomic.fna
5002	Sinorhizobium meliloti	GCA_002197145.1_ASM219714v1_genomic.fna
5003	Brucella melitensis	GCA_002191455.1_ASM219145v1_genomic.fna
5004	Pasteurella multocida	GCA_002073295.1_ASM207329v1_genomic.fna
5005	Acetobacter pasteurianus	GCA_002202135.1_ASM220213v1_genomic.fna
5006	Enterococcus faecium	GCA_001895905.1_ASM189590v1_genomic.fna
5007	Bifidobacterium animalis	GCA_000818055.1_ASM81805v1_genomic.fna
5008	Bacillus amyloliquefaciens	GCA_001596755.1_ASM159675v1_genomic.fna
5009	Enterococcus faecalis	GCA_002355755.1_ASM235575v1_genomic.fna
5010	Klebsiella pneumoniae	GCA_000474015.1_ASM47401v1_genomic.fna
5011	Clostridium botulinum	GCA_000817935.1_ASM81793v1_genomic.fna
5012	Burkholderia thailandensis	GCA_002386205.1_ASM238620v1_genomic.fna
5013	Mycoplasma mycoides	GCA_001554095.1_ASM155409v1_genomic.fna
5014	Vibrio parahaemolyticus	GCA_002073775.1_ASM207377v1_genomic.fna
5015	Bacillus subtilis	GCA_000706705.1_ASM70670v1_genomic.fna
5016	Xanthomonas oryzae	GCA_001518895.1_ASM151889v1_genomic.fna
5017	Xanthomonas citri	GCA_000961375.1_ASM96137v1_genomic.fna
5018	Brucella abortus	GCA_002262995.1_ASM226299v1_genomic.fna
5019	Lactobacillus delbrueckii	GCA_002285775.1_ASM228577v1_genomic.fna
5020	Francisella tularensis	GCA_000313385.1_ASM31338v1_genomic.fna
5021	Ralstonia solanacearum	GCA_002501565.1_ASM250156v1_genomic.fna
5022	Vibrio cholerae	GCA_001318185.1_ASM131818v1_genomic.fna
5023	Burkholderia mallei	GCA_001729545.1_ASM172954v1_genomic.fna
5024	Bacillus thuringiensis	GCA_000803665.1_ASM80366v1_genomic.fna
5025	Burkholderia pseudomallei	GCA_000755925.1_ASM75592v1_genomic.fna
5026	Chlamydia trachomatis	GCA_000220105.1_ASM22010v1_genomic.fna
5027	Corynebacterium glutamicum	GCA_001936195.1_ASM193619v1_genomic.fna
5028	Streptococcus thermophilus	GCA_002012365.1_ASM201236v1_genomic.fna
5029	Legionella pneumophila	GCA_001652665.1_ASM165266v1_genomic.fna

5030	Acinetobacter baumannii	GCA_000695855.3_ASM69585v3_genomic.fna
5031	Pseudomonas aeruginosa	GCA_000508765.1_ASM50876v1_genomic.fna
5032	Streptococcus agalactiae	GCA_001190805.1_ASM119080v1_genomic.fna
5033	Streptococcus suis	GCA_000471985.1_ASM47198v1_genomic.fna
5034	Bifidobacterium longum	GCA_001446255.1_ASM144625v1_genomic.fna
5035	Bacillus anthracis	GCA_000832505.1_ASM83250v1_genomic.fna
5036	Streptococcus pneumoniae	GCA_000210935.1_ASM21093v1_genomic.fna
5037	Streptococcus pyogenes	GCA_000250925.1_ASM25092v1_genomic.fna
5038	Pseudomonas putida	GCA_000691565.1_ASM69156v1_genomic.fna
5039	Neisseria meningitidis	GCA_000787195.2_ASM78719v2_genomic.fna
5040	Buchnera aphidicola	GCA_000021065.1_ASM2106v1_genomic.fna
5041	Helicobacter pylori	GCA_000178935.2_ASM17893v2_genomic.fna
5042	Escherichia coli	GCA_000014845.1_ASM1484v1_genomic.fna
5043	Mycobacterium tuberculosis	GCA_000389945.1_ASM38994v1_genomic.fna
5044	Haemophilus influenzae	GCA_001856725.1_ASM185672v1_genomic.fna
5045	Mycobacterium avium	GCA_002716905.1_ASM271690v1_genomic.fna
5046	Listeria monocytogenes	GCA_000218305.1_ASM21830v1_genomic.fna
5047	Lactococcus lactis	GCA_002078395.1_ASM207839v1_genomic.fna
5048	Bacillus cereus	GCA_000832385.1_ASM83238v1_genomic.fna
5049	Yersinia pestis	GCA_000834335.1_ASM83433v1_genomic.fna
5050	Salmonella enterica	GCA_000022165.1_ASM2216v1_genomic.fna
5051	Pseudomonas fluorescens	GCA_001747385.1_ASM174738v1_genomic.fna
5052	Campylobacter jejuni	GCA_000466105.2_ASM46610v2_genomic.fna
5053	Staphylococcus aureus	GCA_000024585.1_ASM2458v1_genomic.fna
5054	Staphylococcus aureus	GCA_000025145.2_ASM2514v1_genomic.fna
5055	Salmonella enterica	GCA_000026565.1_ASM2656v1_genomic.fna
5056	Campylobacter jejuni	GCA_000468915.2_ASM46891v2_genomic.fna
5057	Yersinia pestis	GCA_000834495.1_ASM83449v1_genomic.fna
5058	Bacillus cereus	GCA_000832405.1_ASM83240v1_genomic.fna
5059	Lactococcus lactis	GCA_002078415.1_ASM207841v1_genomic.fna
5060	Listeria monocytogenes	GCA_000258905.1_ASM25890v1_genomic.fna
5061	Mycobacterium avium	GCA_002716925.1_ASM271692v1_genomic.fna
5062	Haemophilus influenzae	GCA_002073475.1_ASM207347v1_genomic.fna
5063	Mycobacterium tuberculosis	GCA_000400615.1_ASM40061v1_genomic.fna

5064	Escherichia coli	GCA_000017745.1_ASM1774v1_genomic.fna
5065	Helicobacter pylori	GCA_000185185.1_ASM18518v1_genomic.fna
5066	Buchnera aphidicola	GCA_000021085.1_ASM2108v1_genomic.fna
5067	Neisseria meningitidis	GCA_000800235.1_ASM80023v1_genomic.fna
5068	Streptococcus pyogenes	GCA_000307535.1_ASM30753v1_genomic.fna
5069	Streptococcus pneumoniae	GCA_000210955.1_ASM21095v1_genomic.fna
5070	Pseudomonas putida	GCA_001515585.2_ASM151558v2_genomic.fna
5071	Bacillus anthracis	GCA_000832565.1_ASM83256v1_genomic.fna
5072	Bifidobacterium longum	GCA_001446275.1_ASM144627v1_genomic.fna
5073	Streptococcus agalactiae	GCA_001190885.1_ASM119088v1_genomic.fna
5074	Streptococcus suis	GCA_000494895.1_ASM49489v1_genomic.fna
5075	Acinetobacter baumannii	GCA_000746605.1_ASM74660v1_genomic.fna
5076	Pseudomonas aeruginosa	GCA_000510305.1_ASM51030v1_genomic.fna
5077	Legionella pneumophila	GCA_001652685.1_ASM165268v1_genomic.fna
5078	Streptococcus thermophilus	GCA_002286255.1_ASM228625v1_genomic.fna
5079	Corynebacterium glutamicum	GCA_002163915.1_ASM216391v1_genomic.fna
5080	Chlamydia trachomatis	GCA_000226605.1_ASM22660v1_genomic.fna
5081	Burkholderia pseudomallei	GCA_000755945.1_ASM75594v1_genomic.fna
5082	Bacillus thuringiensis	GCA_000832485.1_ASM83248v1_genomic.fna
5083	Burkholderia mallei	GCA_002345985.1_ASM234598v1_genomic.fna
5084	Ralstonia solanacearum	GCA_002549815.1_ASM254981v1_genomic.fna
5085	Vibrio cholerae	GCA_001683415.1_ASM168341v1_genomic.fna
5086	Francisella tularensis	GCA_000524575.1_ASM52457v1_genomic.fna
5087	Lactobacillus delbrueckii	GCA_900196735.1_Lactobacillus_bulgaricus_ACA-DC_87_genomic.fna
5088	Xanthomonas citri	GCA_000961395.1_ASM96139v1_genomic.fna
5089	Xanthomonas oryzae	GCA_001746595.1_ASM174659v1_genomic.fna
5090	Bacillus subtilis	GCA_000737405.1_ASM73740v1_genomic.fna
5091	Vibrio parahaemolyticus	GCA_002209725.2_ASM220972v2_genomic.fna
5092	Mycoplasma mycoides	GCA_001554115.1_ASM155411v1_genomic.fna
5093	Clostridium botulinum	GCA_000827935.1_ASM82793v1_genomic.fna
5094	Klebsiella pneumoniae	GCA_000512165.1_ASM51216v1_genomic.fna
5095	Bifidobacterium animalis	GCA_001688645.2_ASM168864v2_genomic.fna
5096	Bacillus amyloliquefaciens	GCA_001705195.1_ASM170519v1_genomic.fna
5097	Enterococcus faecium	GCA_001953235.1_ASM195323v1_genomic.fna

5098	Acetobacter pasteurianus	GCA_002220195.1_ASM222019v1_genomic.fna
5099	Pasteurella multocida	GCA_002393385.1_ASM239338v1_genomic.fna
5100	Brucella melitensis	GCA_002191575.1_ASM219157v1_genomic.fna
5101	Sinorhizobium meliloti	GCA_002197165.1_ASM219716v1_genomic.fna
5102	Bordetella pertussis	GCA_001307745.1_ASM130774v1_genomic.fna
5103	Mycoplasma pneumoniae	GCA_001272875.1_ASM127287v1_genomic.fna
5104	Dehalococcoides mccartyi	GCA_002007865.1_ASM200786v1_genomic.fna
5105	Lactobacillus plantarum	GCA_001659745.1_ASM165974v1_genomic.fna
5106	Serratia marcescens	GCA_002220655.1_ASM222065v1_genomic.fna
5107	Campylobacter coli	GCA_001936355.1_ASM193635v1_genomic.fna
5108	Enterobacter cloacae	GCA_002025685.1_ASM202568v1_genomic.fna
5109	Mycobacterium abscessus	GCA_001606335.1_ASM160633v1_genomic.fna
5110	Corynebacterium pseudotuberculosis	GCA_000730405.1_ASM73040v1_genomic.fna
5111	Staphylococcus sciuri	GCA_002072755.1_ASM207275v1_genomic.fna
5112	Piscirickettsia salmonis	GCA_001932915.1_ASM193291v1_genomic.fna
5113	Bacillus velezensis	GCA_001593395.2_ASM159339v2_genomic.fna
5114	Bacillus velezensis	GCA_001685645.1_ASM168564v1_genomic.fna
5115	Piscirickettsia salmonis	GCA_001932935.1_ASM193293v1_genomic.fna
5116	Corynebacterium pseudotuberculosis	GCA_000730445.1_ASM73044v1_genomic.fna
5117	Mycobacterium abscessus	GCA_001610615.1_ASM161061v1_genomic.fna
5118	Enterobacter cloacae	GCA_002055735.1_ASM205573v1_genomic.fna
5119	Campylobacter coli	GCA_002407145.1_ASM240714v1_genomic.fna
5120	Serratia marcescens	GCA_002220675.1_ASM222067v1_genomic.fna
5121	Lactobacillus plantarum	GCA_001660025.1_ASM166002v1_genomic.fna
5122	Dehalococcoides mccartyi	GCA_002007905.1_ASM200790v1_genomic.fna
5123	Mycoplasma pneumoniae	GCA_001272895.1_ASM127289v1_genomic.fna
5124	Bordetella pertussis	GCA_001509895.1_ASM150989v1_genomic.fna
5125	Sinorhizobium meliloti	GCA_002197445.1_ASM219744v1_genomic.fna
5126	Brucella melitensis	GCA_002191615.1_ASM219161v1_genomic.fna
5127	Pasteurella multocida	GCA_002591295.1_ASM259129v1_genomic.fna
5128	Enterococcus faecium	GCA_001953255.1_ASM195325v1_genomic.fna
5129	Bifidobacterium animalis	GCA_002220485.1_ASM222048v1_genomic.fna
5130	Bacillus amyloliquefaciens	GCA_001874385.1_ASM187438v1_genomic.fna
5131	Klebsiella pneumoniae	GCA_000597905.1_ASM59790v1_genomic.fna

5132	Clostridium botulinum	GCA_000827955.1_ASM82795v1_genomic.fna
5133	Vibrio parahaemolyticus	GCA_002220985.2_HGAP_genomic.fna
5134	Bacillus subtilis	GCA_000772125.1_ASM77212v1_genomic.fna
5135	Xanthomonas oryzae	GCA_001746615.1_ASM174661v1_genomic.fna
5136	Xanthomonas citri	GCA_000961415.1_ASM96141v1_genomic.fna
5137	Francisella tularensis	GCA_000833165.1_ASM83316v1_genomic.fna
5138	Vibrio cholerae	GCA_001854425.1_ASM185442v1_genomic.fna
5139	Burkholderia mallei	GCA_002346005.1_ASM234600v1_genomic.fna
5140	Bacillus thuringiensis	GCA_000832825.1_ASM83282v1_genomic.fna
5141	Burkholderia pseudomallei	GCA_000755965.1_ASM75596v1_genomic.fna
5142	Chlamydia trachomatis	GCA_000284475.1_ASM28447v1_genomic.fna
5143	Corynebacterium glutamicum	GCA_002220135.1_ASM222013v1_genomic.fna
5144	Streptococcus thermophilus	GCA_002443035.1_ASM244303v1_genomic.fna
5145	Legionella pneumophila	GCA_001677075.2_ASM167707v2_genomic.fna
5146	Pseudomonas aeruginosa	GCA_000524595.1_ASM52459v1_genomic.fna
5147	Streptococcus agalactiae	GCA_001266635.1_ASM126663v1_genomic.fna
5148	Acinetobacter baumannii	GCA_000761175.1_ASM76117v1_genomic.fna
5149	Streptococcus suis	GCA_000732355.1_ASM73235v1_genomic.fna
5150	Bifidobacterium longum	GCA_001719085.1_ASM171908v1_genomic.fna
5151	Bacillus anthracis	GCA_000832585.1_ASM83258v1_genomic.fna
5152	Streptococcus pneumoniae	GCA_000210975.1_ASM21097v1_genomic.fna
5153	Streptococcus pyogenes	GCA_000349925.2_ASM34992v2_genomic.fna
5154	Pseudomonas putida	GCA_001636055.1_ASM163605v1_genomic.fna
5155	Neisseria meningitidis	GCA_000800275.1_ASM80027v1_genomic.fna
5156	Buchnera aphidicola	GCA_000183225.1_ASM18322v1_genomic.fna
5157	Helicobacter pylori	GCA_000185205.1_ASM18520v1_genomic.fna
5158	Escherichia coli	GCA_000017765.1_ASM1776v1_genomic.fna
5159	Mycobacterium avium	GCA_002716965.1_ASM271696v1_genomic.fna
5160	Mycobacterium tuberculosis	GCA_000422125.1_ASM42212v1_genomic.fna
5161	Listeria monocytogenes	GCA_000306905.1_ASM30690v1_genomic.fna
5162	Lactococcus lactis	GCA_002078435.1_ASM207843v1_genomic.fna
5163	Bacillus cereus	GCA_000832525.1_ASM83252v1_genomic.fna
5164	Yersinia pestis	GCA_000834755.1_ASM83475v1_genomic.fna
5165	Salmonella enterica	GCA_000027025.1_ASM2702v1_genomic.fna

5166	Campylobacter jejuni	GCA_000493495.1_TS_genomic.fna
5167	Staphylococcus aureus	GCA_000027045.1_ASM2704v1_genomic.fna
5168	Staphylococcus aureus	GCA_000144955.1_ASM14495v1_genomic.fna
5169	Campylobacter jejuni	GCA_000632435.1_ASM63243v1_genomic.fna
5170	Salmonella enterica	GCA_000188735.1_ASM18873v1_genomic.fna
5171	Yersinia pestis	GCA_000834775.1_ASM83477v1_genomic.fna
5172	Bacillus cereus	GCA_000832765.1_ASM83276v1_genomic.fna
5173	Lactococcus lactis	GCA_002078455.1_ASM207845v1_genomic.fna
5174	Listeria monocytogenes	GCA_000306985.1_ASM30698v1_genomic.fna
5175	Mycobacterium tuberculosis	GCA_000572125.1_ASM57212v1_genomic.fna
5176	Escherichia coli	GCA_000017985.1_ASM1798v1_genomic.fna
5177	Helicobacter pylori	GCA_000185225.1_ASM18522v1_genomic.fna
5178	Buchnera aphidicola	GCA_000183245.1_ASM18324v1_genomic.fna
5179	Neisseria meningitidis	GCA_000800315.1_ASM80031v1_genomic.fna
5180	Pseudomonas putida	GCA_001767335.1_ASM176733v1_genomic.fna
5181	Streptococcus pyogenes	GCA_000422045.1_ASM42204v1_genomic.fna
5182	Streptococcus pneumoniae	GCA_000210995.1_ASM21099v1_genomic.fna
5183	Bacillus anthracis	GCA_000832635.1_ASM83263v1_genomic.fna
5184	Bifidobacterium longum	GCA_001725985.1_ASM172598v1_genomic.fna
5185	Pseudomonas aeruginosa	GCA_000568855.2_ASM56885v2_genomic.fna
5186	Streptococcus suis	GCA_000993745.1_ASM99374v1_genomic.fna
5187	Acinetobacter baumannii	GCA_000786735.1_ASM78673v1_genomic.fna
5188	Streptococcus agalactiae	GCA_001275545.2_ASM127554v2_genomic.fna
5189	Legionella pneumophila	GCA_001677115.2_ASM167711v2_genomic.fna
5190	Streptococcus thermophilus	GCA_900094135.1_ASM90009413v1_genomic.fna
5191	Corynebacterium glutamicum	GCA_002243555.1_ASM224355v1_genomic.fna
5192	Chlamydia trachomatis	GCA_000304495.1_Cm_ESW3_genomic.fna
5193	Burkholderia pseudomallei	GCA_000756065.1_ASM75606v1_genomic.fna
5194	Bacillus thuringiensis	GCA_000832925.1_ASM83292v1_genomic.fna
5195	Burkholderia mallei	GCA_002346025.1_ASM234602v1_genomic.fna
5196	Vibrio cholerae	GCA_001887395.1_ASM188739v1_genomic.fna
5197	Francisella tularensis	GCA_000833235.1_ASM83323v1_genomic.fna
5198	Xanthomonas citri	GCA_000961435.1_ASM96143v1_genomic.fna
5199	Xanthomonas oryzae	GCA_001746635.1_ASM174663v1_genomic.fna

5200	Bacillus subtilis	GCA_000772165.1_ASM77216v1_genomic.fna
5201	Clostridium botulinum	GCA_000829015.1_ASM82901v1_genomic.fna
5202	Klebsiella pneumoniae	GCA_000598005.1_ASM59800v1_genomic.fna
5203	Bacillus amyloliquefaciens	GCA_001889285.1_ASM188928v1_genomic.fna
5204	Enterococcus faecium	GCA_002007625.1_ASM200762v1_genomic.fna
5205	Pasteurella multocida	GCA_900187275.1_51765_F01_genomic.fna
5206	Brucella melitensis	GCA_002191655.1_ASM219165v1_genomic.fna
5207	Sinorhizobium meliloti	GCA_002197465.1_ASM219746v1_genomic.fna
5208	Bordetella pertussis	GCA_001509915.1_ASM150991v1_genomic.fna
5209	Mycoplasma pneumoniae	GCA_001272915.1_ASM127291v1_genomic.fna
5210	Dehalococcoides mccartyi	GCA_002021775.1_ASM202177v1_genomic.fna
5211	Lactobacillus plantarum	GCA_001672035.1_ASM167203v1_genomic.fna
5212	Serratia marcescens	GCA_002220695.1_ASM222069v1_genomic.fna
5213	Enterobacter cloacae	GCA_002192355.1_ASM219235v1_genomic.fna
5214	Mycobacterium abscessus	GCA_001610635.1_ASM161063v1_genomic.fna
5215	Corynebacterium pseudotuberculosis	GCA_000764045.2_ASM76404v2_genomic.fna
5216	Piscirickettsia salmonis	GCA_001932955.1_ASM193295v1_genomic.fna
5217	Bacillus velezensis	GCA_001687745.1_ASM168774v1_genomic.fna
5218	Bacillus velezensis	GCA_001723585.1_ASM172358v1_genomic.fna
5219	Corynebacterium pseudotuberculosis	GCA_000814865.1_ASM81486v1_genomic.fna
5220	Mycobacterium abscessus	GCA_001610655.1_ASM161065v1_genomic.fna
5221	Enterobacter cloacae	GCA_002192395.1_ASM219239v1_genomic.fna
5222	Serratia marcescens	GCA_002220715.1_ASM222071v1_genomic.fna
5223	Lactobacillus plantarum	GCA_001704315.1_ASM170431v1_genomic.fna
5224	Dehalococcoides mccartyi	GCA_002024205.1_ASM202420v1_genomic.fna
5225	Mycoplasma pneumoniae	GCA_001509195.1_ASM150919v1_genomic.fna
5226	Bordetella pertussis	GCA_001605035.1_ASM160503v1_genomic.fna
5227	Sinorhizobium meliloti	GCA_002215195.1_ASM221519v1_genomic.fna
5228	Brucella melitensis	GCA_002191675.1_ASM219167v1_genomic.fna
5229	Enterococcus faecium	GCA_002024245.1_ASM202424v1_genomic.fna
5230	Bacillus amyloliquefaciens	GCA_001922005.1_ASM192200v1_genomic.fna
5231	Klebsiella pneumoniae	GCA_000695935.1_ASM69593v1_genomic.fna
5232	Clostridium botulinum	GCA_001879605.1_ASM187960v1_genomic.fna
5233	Bacillus subtilis	GCA_000772205.1_ASM77220v1_genomic.fna

5234	Xanthomonas oryzae	GCA_001746655.1_ASM174665v1_genomic.fna
5235	Xanthomonas citri	GCA_000961455.1_ASM96145v1_genomic.fna
5236	Francisella tularensis	GCA_000833335.1_ASM83333v1_genomic.fna
5237	Vibrio cholerae	GCA_001887415.1_ASM188741v1_genomic.fna
5238	Burkholderia mallei	GCA_002346045.1_ASM234604v1_genomic.fna
5239	Bacillus thuringiensis	GCA_000833085.1_ASM83308v1_genomic.fna
5240	Burkholderia pseudomallei	GCA_000756085.1_ASM75608v1_genomic.fna
5241	Chlamydia trachomatis	GCA_000304515.1_Cm_FSW4_genomic.fna
5242	Corynebacterium glutamicum	GCA_002277975.1_ASM227797v1_genomic.fna
5243	Legionella pneumophila	GCA_001752705.1_ASM175270v1_genomic.fna
5244	Streptococcus agalactiae	GCA_001448985.1_ASM144898v1_genomic.fna
5245	Pseudomonas aeruginosa	GCA_000816985.1_ASM81698v1_genomic.fna
5246	Acinetobacter baumannii	GCA_000814345.1_ASM81434v1_genomic.fna
5247	Streptococcus suis	GCA_001272635.1_ASM127263v1_genomic.fna
5248	Bacillus anthracis	GCA_000832665.1_ASM83266v1_genomic.fna
5249	Streptococcus pneumoniae	GCA_000211015.1_ASM21101v1_genomic.fna
5250	Streptococcus pyogenes	GCA_000732425.1_ASM73242v1_genomic.fna
5251	Pseudomonas putida	GCA_001886975.1_ASM188697v1_genomic.fna
5252	Neisseria meningitidis	GCA_000800355.1_ASM80035v1_genomic.fna
5253	Buchnera aphidicola	GCA_000183285.1_ASM18328v1_genomic.fna
5254	Helicobacter pylori	GCA_000185245.1_ASM18524v1_genomic.fna
5255	Escherichia coli	GCA_000019385.1_ASM1938v1_genomic.fna
5256	Mycobacterium tuberculosis	GCA_000572155.1_ASM57215v1_genomic.fna
5257	Listeria monocytogenes	GCA_000307005.1_ASM30700v1_genomic.fna
5258	Lactococcus lactis	GCA_002078475.1_ASM207847v1_genomic.fna
5259	Bacillus cereus	GCA_000832805.1_ASM83280v1_genomic.fna
5260	Yersinia pestis	GCA_000834825.1_ASM83482v1_genomic.fna
5261	Campylobacter jejuni	GCA_000737085.1_ASM73708v1_genomic.fna
5262	Salmonella enterica	GCA_000188955.5_ASM18895v5_genomic.fna
5263	Staphylococcus aureus	GCA_000145595.1_ASM14559v1_genomic.fna
5264	Staphylococcus aureus	GCA_000159535.2_ASM15953v2_genomic.fna
5265	Campylobacter jejuni	GCA_000772225.1_ASM77222v1_genomic.fna
5266	Salmonella enterica	GCA_000210855.2_ASM21085v2_genomic.fna
5267	Yersinia pestis	GCA_000834845.1_ASM83484v1_genomic.fna

5268	Bacillus cereus	GCA_000832845.1_ASM83284v1_genomic.fna
5269	Lactococcus lactis	GCA_002078495.1_ASM207849v1_genomic.fna
5270	Listeria monocytogenes	GCA_000307025.1_ASM30702v1_genomic.fna
5271	Mycobacterium tuberculosis	GCA_000572175.1_ASM57217v1_genomic.fna
5272	Escherichia coli	GCA_000019425.1_ASM1942v1_genomic.fna
5273	Helicobacter pylori	GCA_000192315.1_ASM19231v1_genomic.fna
5274	Buchnera aphidicola	GCA_000183305.1_ASM18330v1_genomic.fna
5275	Neisseria meningitidis	GCA_000800415.1_ASM80041v1_genomic.fna
5276	Pseudomonas putida	GCA_002025705.1_ASM202570v1_genomic.fna
5277	Streptococcus pyogenes	GCA_000743015.1_ASM74301v1_genomic.fna
5278	Streptococcus pneumoniae	GCA_000211035.2_ASM21103v2_genomic.fna
5279	Bacillus anthracis	GCA_000832725.1_ASM83272v1_genomic.fna
5280	Streptococcus suis	GCA_001572685.1_ASM157268v1_genomic.fna
5281	Acinetobacter baumannii	GCA_000828795.1_ASM82879v1_genomic.fna
5282	Pseudomonas aeruginosa	GCA_000829255.1_ASM82925v1_genomic.fna
5283	Streptococcus agalactiae	GCA_001552035.1_ASM155203v1_genomic.fna
5284	Legionella pneumophila	GCA_001752725.1_ASM175272v1_genomic.fna
5285	Chlamydia trachomatis	GCA_000304535.1_Cm_FSW5_genomic.fna
5286	Corynebacterium glutamicum	GCA_002355155.1_ASM235515v1_genomic.fna
5287	Burkholderia pseudomallei	GCA_000756125.1_ASM75612v1_genomic.fna
5288	Bacillus thuringiensis	GCA_000835025.1_ASM83502v1_genomic.fna
5289	Burkholderia mallei	GCA_002346065.1_ASM234606v1_genomic.fna
5290	Vibrio cholerae	GCA_001887435.1_ASM188743v1_genomic.fna
5291	Francisella tularensis	GCA_000833355.1_ASM83335v1_genomic.fna
5292	Xanthomonas citri	GCA_000961475.1_ASM96147v1_genomic.fna
5293	Xanthomonas oryzae	GCA_001746675.1_ASM174667v1_genomic.fna
5294	Bacillus subtilis	GCA_000782835.1_ASM78283v1_genomic.fna
5295	Clostridium botulinum	GCA_001879625.1_ASM187962v1_genomic.fna
5296	Klebsiella pneumoniae	GCA_000714675.1_ASM71467v1_genomic.fna
5297	Bacillus amyloliquefaciens	GCA_002173635.1_ASM217363v1_genomic.fna
5298	Enterococcus faecium	GCA_002025045.1_ASM202504v1_genomic.fna
5299	Brucella melitensis	GCA_002191755.1_ASM219175v1_genomic.fna
5300	Sinorhizobium meliloti	GCA_002302355.1_ASM230235v1_genomic.fna
5301	Bordetella pertussis	GCA_001605055.1_ASM160505v1_genomic.fna

5302	Mycoplasma pneumoniae	GCA_001558175.1_ASM155817v1_genomic.fna
5303	Dehalococcoides mccartyi	GCA_002024225.1_ASM202422v1_genomic.fna
5304	Lactobacillus plantarum	GCA_001704335.1_ASM170433v1_genomic.fna
5305	Serratia marcescens	GCA_900029885.1_Sm_SMB2099_genomic.fna
5306	Enterobacter cloacae	GCA_002197345.1_ASM219734v1_genomic.fna
5307	Mycobacterium abscessus	GCA_001610675.1_ASM161067v1_genomic.fna
5308	Corynebacterium pseudotuberculosis	GCA_000972805.1_ASM97280v1_genomic.fna
5309	Bacillus velezensis	GCA_001752685.1_ASM175268v1_genomic.fna
5310	Bacillus velezensis	GCA_001854345.1_ASM185434v1_genomic.fna
5311	Corynebacterium pseudotuberculosis	GCA_001017615.1_ASM101761v1_genomic.fna
5312	Mycobacterium abscessus	GCA_001677095.1_ASM167709v1_genomic.fna
5313	Enterobacter cloacae	GCA_002201815.1_ASM220181v1_genomic.fna
5314	Lactobacillus plantarum	GCA_001715615.1_ASM171561v1_genomic.fna
5315	Dehalococcoides mccartyi	GCA_002028285.1_ASM202828v1_genomic.fna
5316	Mycoplasma pneumoniae	GCA_001901705.1_ASM190170v1_genomic.fna
5317	Bordetella pertussis	GCA_001605075.1_ASM160507v1_genomic.fna
5318	Sinorhizobium meliloti	GCA_002302375.1_ASM230237v1_genomic.fna
5319	Brucella melitensis	GCA_002191915.1_ASM219191v1_genomic.fna
5320	Enterococcus faecium	GCA_002025065.1_ASM202506v1_genomic.fna
5321	Bacillus amyloliquefaciens	GCA_002209305.1_ASM220930v1_genomic.fna
5322	Klebsiella pneumoniae	GCA_000717515.1_ASM71751v1_genomic.fna
5323	Clostridium botulinum	GCA_001886775.1_ASM188677v1_genomic.fna
5324	Bacillus subtilis	GCA_000789275.1_ASM78927v1_genomic.fna
5325	Xanthomonas oryzae	GCA_001746695.1_ASM174669v1_genomic.fna
5326	Xanthomonas citri	GCA_000961495.1_ASM96149v1_genomic.fna
5327	Francisella tularensis	GCA_000833375.1_ASM83337v1_genomic.fna
5328	Vibrio cholerae	GCA_001887455.1_ASM188745v1_genomic.fna
5329	Burkholderia mallei	GCA_002346085.1_ASM234608v1_genomic.fna
5330	Bacillus thuringiensis	GCA_000940785.1_ASM94078v1_genomic.fna
5331	Burkholderia pseudomallei	GCA_000756145.1_ASM75614v1_genomic.fna
5332	Corynebacterium glutamicum	GCA_002355675.1_ASM235567v1_genomic.fna
5333	Chlamydia trachomatis	GCA_000318525.2_ASM31852v2_genomic.fna
5334	Legionella pneumophila	GCA_001752745.1_ASM175274v1_genomic.fna
5335	Streptococcus agalactiae	GCA_001592385.1_ASM159238v1_genomic.fna

5336	Pseudomonas aeruginosa	GCA_000829275.1_ASM82927v1_genomic.fna
5337	Acinetobacter baumannii	GCA_000828935.1_ASM82893v1_genomic.fna
5338	Streptococcus suis	GCA_001610935.1_ASM161093v1_genomic.fna
5339	Bacillus anthracis	GCA_000832745.1_ASM83274v1_genomic.fna
5340	Streptococcus pneumoniae	GCA_000211055.2_ASM21105v2_genomic.fna
5341	Streptococcus pyogenes	GCA_000756485.1_ASM75648v1_genomic.fna
5342	Pseudomonas putida	GCA_002356095.1_ASM235609v1_genomic.fna
5343	Neisseria meningitidis	GCA_000816165.1_ASM81616v1_genomic.fna
5344	Buchnera aphidicola	GCA_000521525.1_ASM52152v1_genomic.fna
5345	Helicobacter pylori	GCA_000192335.1_ASM19233v1_genomic.fna
5346	Escherichia coli	GCA_000019645.1_ASM1964v1_genomic.fna
5347	Mycobacterium tuberculosis	GCA_000572195.1_ASM57219v1_genomic.fna
5348	Listeria monocytogenes	GCA_000307045.1_ASM30704v1_genomic.fna
5349	Lactococcus lactis	GCA_002078615.1_ASM207861v1_genomic.fna
5350	Bacillus cereus	GCA_000832865.1_ASM83286v1_genomic.fna
5351	Staphylococcus aureus	GCA_000160335.2_ASM16033v2_genomic.fna
5352	Yersinia pestis	GCA_000834885.1_ASM83488v1_genomic.fna
5353	Salmonella enterica	GCA_000213635.1_ASM21363v1_genomic.fna
5354	Campylobacter jejuni	GCA_000807355.1_ASM80735v1_genomic.fna
5355	Campylobacter jejuni	GCA_000830775.1_ASM83077v1_genomic.fna
5356	Salmonella enterica	GCA_000235545.1_ASM23554v1_genomic.fna
5357	Staphylococcus aureus	GCA_000204665.1_ASM20466v1_genomic.fna
5358	Yersinia pestis	GCA_000834925.1_ASM83492v1_genomic.fna
5359	Bacillus cereus	GCA_000833045.1_ASM83304v1_genomic.fna
5360	Lactococcus lactis	GCA_002078765.1_ASM207876v1_genomic.fna
5361	Listeria monocytogenes	GCA_000307065.1_ASM30706v1_genomic.fna
5362	Mycobacterium tuberculosis	GCA_000698475.1_ASM69847v1_genomic.fna
5363	Escherichia coli	GCA_000021125.1_ASM2112v1_genomic.fna
5364	Helicobacter pylori	GCA_000196755.1_ASM19675v1_genomic.fna
5365	Buchnera aphidicola	GCA_000521545.1_ASM52154v1_genomic.fna
5366	Neisseria meningitidis	GCA_001029815.1_ASM102981v1_genomic.fna
5367	Pseudomonas putida	GCA_002736045.1_ASM273604v1_genomic.fna
5368	Streptococcus pyogenes	GCA_000767505.1_ASM76750v1_genomic.fna
5369	Streptococcus pneumoniae	GCA_000211075.1_ASM21107v1_genomic.fna

5370	Bacillus anthracis	GCA_000832785.1_ASM83278v1_genomic.fna
5371	Streptococcus suis	GCA_001640765.1_ASM164076v1_genomic.fna
5372	Acinetobacter baumannii	GCA_000830055.1_ASM83005v1_genomic.fna
5373	Pseudomonas aeruginosa	GCA_000829885.1_ASM82988v1_genomic.fna
5374	Streptococcus agalactiae	GCA_001592425.1_ASM159242v1_genomic.fna
5375	Legionella pneumophila	GCA_001752765.1_ASM175276v1_genomic.fna
5376	Chlamydia trachomatis	GCA_000318565.1_ASM31856v1_genomic.fna
5377	Burkholderia pseudomallei	GCA_000756165.1_ASM75616v1_genomic.fna
5378	Bacillus thuringiensis	GCA_001017635.1_ASM101763v1_genomic.fna
5379	Burkholderia mallei	GCA_002346105.1_ASM234610v1_genomic.fna
5380	Vibrio cholerae	GCA_001887475.1_ASM188747v1_genomic.fna
5381	Francisella tularensis	GCA_000833475.1_ASM83347v1_genomic.fna
5382	Xanthomonas oryzae	GCA_001746715.1_ASM174671v1_genomic.fna
5383	Xanthomonas citri	GCA_001028285.3_ASM102828v3_genomic.fna
5384	Bacillus subtilis	GCA_000789295.1_ASM78929v1_genomic.fna
5385	Clostridium botulinum	GCA_001889325.1_ASM188932v1_genomic.fna
5386	Klebsiella pneumoniae	GCA_000733255.1_ASM73325v1_genomic.fna
5387	Enterococcus faecium	GCA_002334625.1_ASM233462v1_genomic.fna
5388	Brucella melitensis	GCA_002192095.1_ASM219209v1_genomic.fna
5389	Bordetella pertussis	GCA_001605095.1_ASM160509v1_genomic.fna
5390	Mycoplasma pneumoniae	GCA_002090215.1_ASM209021v1_genomic.fna
5391	Dehalococcoides mccartyi	GCA_002356375.1_ASM235637v1_genomic.fna
5392	Lactobacillus plantarum	GCA_001874125.1_ASM187412v1_genomic.fna
5393	Enterobacter cloacae	GCA_002204775.1_ASM220477v1_genomic.fna
5394	Mycobacterium abscessus	GCA_001677155.1_ASM167715v1_genomic.fna
5395	Corynebacterium pseudotuberculosis	GCA_001026945.1_ASM102694v1_genomic.fna
5396	Bacillus velezensis	GCA_001857985.1_ASM185798v1_genomic.fna
5397	Bacillus velezensis	GCA_002005345.1_ASM200534v1_genomic.fna
5398	Corynebacterium pseudotuberculosis	GCA_001047215.2_ASM104721v2_genomic.fna
5399	Mycobacterium abscessus	GCA_001677175.1_ASM167717v1_genomic.fna
5400	Enterobacter cloacae	GCA_002211685.1_ASM221168v1_genomic.fna
5401	Lactobacillus plantarum	GCA_001880185.1_ASM188018v1_genomic.fna
5402	Mycoplasma pneumoniae	GCA_002090235.1_ASM209023v1_genomic.fna
5403	Bordetella pertussis	GCA_001605115.1_ASM160511v1_genomic.fna

5404	Brucella melitensis	GCA_002192155.1_ASM219215v1_genomic.fna
5405	Enterococcus faecium	GCA_002442955.1_ASM244295v1_genomic.fna
5406	Klebsiella pneumoniae	GCA_000739495.1_ASM73949v1_genomic.fna
5407	Clostridium botulinum	GCA_001889345.1_ASM188934v1_genomic.fna
5408	Bacillus subtilis	GCA_000827065.1_ASM82706v1_genomic.fna
5409	Xanthomonas citri	GCA_001719145.1_ASM171914v1_genomic.fna
5410	Xanthomonas oryzae	GCA_001746735.1_ASM174673v1_genomic.fna
5411	Francisella tularensis	GCA_000833495.1_ASM83349v1_genomic.fna
5412	Vibrio cholerae	GCA_001887495.1_ASM188749v1_genomic.fna
5413	Burkholderia mallei	GCA_002346125.1_ASM234612v1_genomic.fna
5414	Bacillus thuringiensis	GCA_001182785.1_ASM118278v1_genomic.fna
5415	Burkholderia pseudomallei	GCA_000756185.1_ASM75618v1_genomic.fna
5416	Chlamydia trachomatis	GCA_000318645.1_ASM31864v1_genomic.fna
5417	Legionella pneumophila	GCA_001752785.1_ASM175278v1_genomic.fna
5418	Acinetobacter baumannii	GCA_000939415.2_HGAP_assembly_of_Acinetobacter_baumannii_ORAB01_genomic.fna
5419	Pseudomonas aeruginosa	GCA_000981825.1_ASM98182v1_genomic.fna
5420	Streptococcus suis	GCA_001721625.1_ASM172162v1_genomic.fna
5421	Bacillus anthracis	GCA_000832965.1_ASM83296v1_genomic.fna
5422	Streptococcus agalactiae	GCA_001683515.1_ASM168351v1_genomic.fna
5423	Streptococcus pneumoniae	GCA_000211095.1_ASM21109v1_genomic.fna
5424	Streptococcus pyogenes	GCA_000772185.1_ASM77218v1_genomic.fna
5425	Pseudomonas putida	GCA_002736125.1_ASM273612v1_genomic.fna
5426	Neisseria meningitidis	GCA_001029835.1_ASM102983v1_genomic.fna
5427	Buchnera aphidicola	GCA_000521585.1_ASM52158v1_genomic.fna
5428	Helicobacter pylori	GCA_000213135.1_ASM21313v1_genomic.fna
5429	Escherichia coli	GCA_000022225.1_ASM2222v1_genomic.fna
5430	Mycobacterium tuberculosis	GCA_000706665.1_ASM70666v1_genomic.fna
5431	Listeria monocytogenes	GCA_000307085.1_ASM30708v1_genomic.fna
5432	Lactococcus lactis	GCA_002078855.1_ASM207885v1_genomic.fna
5433	Bacillus cereus	GCA_000835185.1_ASM83518v1_genomic.fna
5434	Yersinia pestis	GCA_000834985.1_ASM83498v1_genomic.fna
5435	Staphylococcus aureus	GCA_000210315.1_ASM21031v1_genomic.fna
5436	Salmonella enterica	GCA_000245535.1_ASM24553v1_genomic.fna
5437	Campylobacter jejuni	GCA_000830805.1_ASM83080v1_genomic.fna

5438	Campylobacter jejuni	GCA_000830825.1_ASM83082v1_genomic.fna
5439	Salmonella enterica	GCA_000252875.1_ASM25287v1_genomic.fna
5440	Staphylococcus aureus	GCA_000237125.1_ASM23712v1_genomic.fna
5441	Yersinia pestis	GCA_001188695.1_ASM118869v1_genomic.fna
5442	Bacillus cereus	GCA_000978375.1_ASM97837v1_genomic.fna
5443	Lactococcus lactis	GCA_002078895.1_ASM207889v1_genomic.fna
5444	Listeria monocytogenes	GCA_000307615.1_ASM30761v1_genomic.fna
5445	Mycobacterium tuberculosis	GCA_000738445.1_ASM73844v1_genomic.fna
5446	Escherichia coli	GCA_000022345.1_ASM2234v1_genomic.fna
5447	Helicobacter pylori	GCA_000224535.1_ASM22453v1_genomic.fna
5448	Pseudomonas putida	GCA_002741075.1_IEC330191.0_genomic.fna
5449	Neisseria meningitidis	GCA_001697105.1_ASM169710v1_genomic.fna
5450	Streptococcus pyogenes	GCA_000772245.1_ASM77224v1_genomic.fna
5451	Streptococcus pneumoniae	GCA_000251085.2_ASM25108v2_genomic.fna
5452	Bacillus anthracis	GCA_000833065.1_ASM83306v1_genomic.fna
5453	Streptococcus suis	GCA_001936415.1_ASM193641v1_genomic.fna
5454	Pseudomonas aeruginosa	GCA_001045685.1_ASM104568v1_genomic.fna
5455	Streptococcus agalactiae	GCA_001712835.1_ASM171283v1_genomic.fna
5456	Acinetobacter baumannii	GCA_000963815.1_ASM96381v1_genomic.fna
5457	Legionella pneumophila	GCA_001752805.1_ASM175280v1_genomic.fna
5458	Chlamydia trachomatis	GCA_000318765.1_ASM31876v1_genomic.fna
5459	Bacillus thuringiensis	GCA_001183785.1_ASM118378v1_genomic.fna
5460	Burkholderia mallei	GCA_002346145.1_ASM234614v1_genomic.fna
5461	Burkholderia pseudomallei	GCA_000757015.2_ASM75701v2_genomic.fna
5462	Vibrio cholerae	GCA_001887515.1_ASM188751v1_genomic.fna
5463	Francisella tularensis	GCA_000833515.1_ASM83351v1_genomic.fna
5464	Xanthomonas citri	GCA_001854145.1_ASM185414v1_genomic.fna
5465	Xanthomonas oryzae	GCA_002023005.1_ASM202300v1_genomic.fna
5466	Bacillus subtilis	GCA_000953615.1_BS49Ch_genomic.fna
5467	Klebsiella pneumoniae	GCA_000742755.1_ASM74275v1_genomic.fna
5468	Enterococcus faecium	GCA_900066025.1_Enterococcus_faecium_E1002_isolate_genomic.fna
5469	Brucella melitensis	GCA_002214285.1_ASM221428v1_genomic.fna
5470	Bordetella pertussis	GCA_001605135.1_ASM160513v1_genomic.fna
5471	Mycoplasma pneumoniae	GCA_002090275.1_ASM209027v1_genomic.fna

5472	Lactobacillus plantarum	GCA_001908455.1_ASM190845v1_genomic.fna
5473	Enterobacter cloacae	GCA_002237465.1_ASM223746v1_genomic.fna
5474	Mycobacterium abscessus	GCA_001677195.1_ASM167719v1_genomic.fna
5475	Corynebacterium pseudotuberculosis	GCA_001186445.1_ASM118644v1_genomic.fna
5476	Bacillus velezensis	GCA_002057535.1_ASM205753v1_genomic.fna
5477	Bacillus velezensis	GCA_002072695.1_ASM207269v1_genomic.fna
5478	Corynebacterium pseudotuberculosis	GCA_001298505.1_ASM129850v1_genomic.fna
5479	Mycobacterium abscessus	GCA_001677215.1_ASM167721v1_genomic.fna
5480	Enterobacter cloacae	GCA_002303275.1_ASM230327v1_genomic.fna
5481	Lactobacillus plantarum	GCA_001990145.1_ASM199014v1_genomic.fna
5482	Mycoplasma pneumoniae	GCA_002090295.1_ASM209029v1_genomic.fna
5483	Bordetella pertussis	GCA_001605155.1_ASM160515v1_genomic.fna
5484	Brucella melitensis	GCA_002262955.1_ASM226295v1_genomic.fna
5485	Enterococcus faecium	GCA_900092475.1_Ef_aus00233_genomic.fna
5486	Klebsiella pneumoniae	GCA_000764615.1_ASM76461v1_genomic.fna
5487	Bacillus subtilis	GCA_000971925.1_ASM97192v1_genomic.fna
5488	Xanthomonas citri	GCA_001922045.1_ASM192204v1_genomic.fna
5489	Francisella tularensis	GCA_000833535.1_ASM83353v1_genomic.fna
5490	Vibrio cholerae	GCA_001887615.1_ASM188761v1_genomic.fna
5491	Burkholderia pseudomallei	GCA_000757035.2_ASM75703v2_genomic.fna
5492	Burkholderia mallei	GCA_002346165.1_ASM234616v1_genomic.fna
5493	Bacillus thuringiensis	GCA_001420855.1_ASM142085v1_genomic.fna
5494	Chlamydia trachomatis	GCA_000318785.1_ASM31878v1_genomic.fna
5495	Legionella pneumophila	GCA_001752825.1_ASM175282v1_genomic.fna
5496	Acinetobacter baumannii	GCA_001026965.1_ASM102696v1_genomic.fna
5497	Streptococcus agalactiae	GCA_001729925.1_ASM172992v1_genomic.fna
5498	Pseudomonas aeruginosa	GCA_001077475.1_ASM107747v1_genomic.fna
5499	Streptococcus suis	GCA_002111405.1_ASM211140v1_genomic.fna
5500	Bacillus anthracis	GCA_000833125.1_ASM83312v1_genomic.fna
5501	Streptococcus pyogenes	GCA_000993765.1_ASM99376v1_genomic.fna
5502	Neisseria meningitidis	GCA_001697125.1_ASM169712v1_genomic.fna
5503	Streptococcus pneumoniae	GCA_000299015.1_ASM29901v1_genomic.fna
5504	Helicobacter pylori	GCA_000224555.1_ASM22455v1_genomic.fna
5505	Escherichia coli	GCA_000022665.2_ASM2266v1_genomic.fna

5506	Mycobacterium tuberculosis	GCA_000738475.1_ASM73847v1_genomic.fna
5507	Listeria monocytogenes	GCA_000318055.1_ASM31805v1_genomic.fna
5508	Lactococcus lactis	GCA_002078915.1_ASM207891v1_genomic.fna
5509	Bacillus cereus	GCA_001277915.1_ASM127791v1_genomic.fna
5510	Yersinia pestis	GCA_001188715.1_ASM118871v1_genomic.fna
5511	Staphylococcus aureus	GCA_000237265.1_ASM23726v1_genomic.fna
5512	Salmonella enterica	GCA_000258365.1_ASM25836v1_genomic.fna
5513	Campylobacter jejuni	GCA_000830845.1_ASM83084v1_genomic.fna
5514	Campylobacter jejuni	GCA_000830865.1_ASM83086v1_genomic.fna
5515	Staphylococcus aureus	GCA_000239235.1_ASM23923v1_genomic.fna
5516	Salmonella enterica	GCA_000271885.2_ASM27188v2_genomic.fna
5517	Yersinia pestis	GCA_001188755.1_ASM118875v1_genomic.fna
5518	Bacillus cereus	GCA_001518875.1_ASM151887v1_genomic.fna
5519	Lactococcus lactis	GCA_002078935.1_ASM207893v1_genomic.fna
5520	Listeria monocytogenes	GCA_000438585.1_ASM43858v1_genomic.fna
5521	Mycobacterium tuberculosis	GCA_000756525.1_ASM75652v1_genomic.fna
5522	Escherichia coli	GCA_000023365.1_ASM2336v1_genomic.fna
5523	Helicobacter pylori	GCA_000224575.1_ASM22457v1_genomic.fna
5524	Streptococcus pneumoniae	GCA_000817005.1_ASM81700v1_genomic.fna
5525	Neisseria meningitidis	GCA_001697165.1_ASM169716v1_genomic.fna
5526	Streptococcus pyogenes	GCA_001014285.1_ASM101428v1_genomic.fna
5527	Bacillus anthracis	GCA_000833275.1_ASM83327v1_genomic.fna
5528	Streptococcus suis	GCA_002189695.1_ASM218969v1_genomic.fna
5529	Streptococcus agalactiae	GCA_001932715.1_ASM193271v1_genomic.fna
5530	Pseudomonas aeruginosa	GCA_001293085.1_ASM129308v1_genomic.fna
5531	Legionella pneumophila	GCA_001752845.1_ASM175284v1_genomic.fna
5532	Acinetobacter baumannii	GCA_001077555.2_ASM107755v2_genomic.fna
5533	Chlamydia trachomatis	GCA_000318825.1_ASM31882v1_genomic.fna
5534	Bacillus thuringiensis	GCA_001455345.1_ASM145534v1_genomic.fna
5535	Burkholderia mallei	GCA_002346185.1_ASM234618v1_genomic.fna
5536	Burkholderia pseudomallei	GCA_000764575.1_ASM76457v1_genomic.fna
5537	Vibrio cholerae	GCA_001887635.1_ASM188763v1_genomic.fna
5538	Francisella tularensis	GCA_000978785.2_ASM97878v2_genomic.fna
5539	Xanthomonas citri	GCA_001922065.1_ASM192206v1_genomic.fna

5540	Bacillus subtilis	GCA_000973605.1_ASM97360v1_genomic.fna
5541	Klebsiella pneumoniae	GCA_000775375.1_ASM77537v1_genomic.fna
5542	Streptococcus dysgalactiae	GCA_000493775.1_ASM49377v1_genomic.fna
5543	Brucella melitensis	GCA_002263015.1_ASM226301v1_genomic.fna
5544	Bordetella pertussis	GCA_001605175.1_ASM160517v1_genomic.fna
5545	Mycoplasma pneumoniae	GCA_002090315.1_ASM209031v1_genomic.fna
5546	Lactobacillus plantarum	GCA_002024845.1_ASM202484v1_genomic.fna
5547	Enterobacter cloacae	GCA_002356475.1_E_cloacae_1.0_genomic.fna
5548	Mycobacterium abscessus	GCA_002140035.1_ASM214003v1_genomic.fna
5549	Corynebacterium pseudotuberculosis	GCA_001433475.1_ASM143347v1_genomic.fna
5550	Bacillus velezensis	GCA_002105595.1_ASM210559v1_genomic.fna
5551	Bacillus velezensis	GCA_002117165.1_ASM211716v1_genomic.fna
5552	Corynebacterium pseudotuberculosis	GCA_001447295.1_ASM144729v1_genomic.fna
5553	Lactobacillus plantarum	GCA_002028365.1_ASM202836v1_genomic.fna
5554	Dehalococcoides mccartyi	GCA_001761445.1_ASM176144v1_genomic.fna
5555	Mycoplasma pneumoniae	GCA_002095995.1_ASM209599v1_genomic.fna
5556	Bordetella pertussis	GCA_001605195.1_ASM160519v1_genomic.fna
5557	Klebsiella pneumoniae	GCA_000775395.1_ASM77539v1_genomic.fna
5558	Bacillus subtilis	GCA_001015095.1_ASM101509v1_genomic.fna
5559	Xanthomonas citri	GCA_001922085.1_ASM192208v1_genomic.fna
5560	Francisella tularensis	GCA_001011135.1_ASM101113v1_genomic.fna
5561	Vibrio cholerae	GCA_001887655.1_ASM188765v1_genomic.fna
5562	Bacillus thuringiensis	GCA_001548175.1_ASM154817v1_genomic.fna
5563	Burkholderia pseudomallei	GCA_000770395.1_ASM77039v1_genomic.fna
5564	Burkholderia mallei	GCA_002346205.1_ASM234620v1_genomic.fna
5565	Chlamydia trachomatis	GCA_000318845.1_ASM31884v1_genomic.fna
5566	Legionella pneumophila	GCA_001752865.1_ASM175286v1_genomic.fna
5567	Acinetobacter baumannii	GCA_001077655.1_ASM107765v1_genomic.fna
5568	Pseudomonas aeruginosa	GCA_001447845.1_ASM144784v1_genomic.fna
5569	Streptococcus agalactiae	GCA_002025005.1_ASM202500v1_genomic.fna
5570	Streptococcus suis	GCA_002688665.1_ASM268866v1_genomic.fna
5571	Bacillus anthracis	GCA_000875715.1_ASM87571v1_genomic.fna
5572	Streptococcus pyogenes	GCA_001014305.1_ASM101430v1_genomic.fna
5573	Neisseria meningitidis	GCA_001697205.1_ASM169720v1_genomic.fna

5574	Streptococcus pneumoniae	GCA_001255215.1_S_pneumo_A66_v1_genomic.fna
5575	Helicobacter pylori	GCA_000255955.1_ASM25595v1_genomic.fna
5576	Escherichia coli	GCA_000023665.1_ASM2366v1_genomic.fna
5577	Mycobacterium tuberculosis	GCA_000756545.1_ASM75654v1_genomic.fna
5578	Listeria monocytogenes	GCA_000438605.1_ASM43860v1_genomic.fna
5579	Lactococcus lactis	GCA_002078955.1_ASM207895v1_genomic.fna
5580	Bacillus cereus	GCA_001635915.1_ASM163591v1_genomic.fna
5581	Yersinia pestis	GCA_001188775.1_ASM118877v1_genomic.fna
5582	Salmonella enterica	GCA_000271905.2_ASM27190v2_genomic.fna
5583	Staphylococcus aureus	GCA_000245495.1_ASM24549v1_genomic.fna
5584	Campylobacter jejuni	GCA_000835285.1_ASM83528v1_genomic.fna
5585	Campylobacter jejuni	GCA_000835305.1_ASM83530v1_genomic.fna
5586	Staphylococcus aureus	GCA_000253135.1_ASM25313v1_genomic.fna
5587	Yersinia pestis	GCA_001188795.1_ASM118879v1_genomic.fna
5588	Salmonella enterica	GCA_000272715.4_ASM27271v4_genomic.fna
5589	Bacillus cereus	GCA_001635955.1_ASM163595v1_genomic.fna
5590	Lactococcus lactis	GCA_002078975.1_ASM207897v1_genomic.fna
5591	Listeria monocytogenes	GCA_000438625.1_ASM43862v1_genomic.fna
5592	Mycobacterium tuberculosis	GCA_000786505.1_MT49-02_genomic.fna
5593	Escherichia coli	GCA_000025165.1_ASM2516v1_genomic.fna
5594	Helicobacter pylori	GCA_000259235.1_ASM25923v1_genomic.fna
5595	Neisseria meningitidis	GCA_001697305.1_ASM169730v1_genomic.fna
5596	Streptococcus pyogenes	GCA_001019635.1_ASM101963v1_genomic.fna
5597	Bacillus anthracis	GCA_001543225.1_ASM154322v1_genomic.fna
5598	Streptococcus pneumoniae	GCA_001457635.1_NCTC7465_genomic.fna
5599	Streptococcus suis	GCA_002736265.1_ASM273626v1_genomic.fna
5600	Streptococcus agalactiae	GCA_002025025.1_ASM202502v1_genomic.fna
5601	Pseudomonas aeruginosa	GCA_001457615.1_NCTC10332_genomic.fna
5602	Acinetobacter baumannii	GCA_001077675.1_ASM107767v1_genomic.fna
5603	Legionella pneumophila	GCA_001752885.1_ASM175288v1_genomic.fna
5604	Chlamydia trachomatis	GCA_000318865.1_ASM31886v1_genomic.fna
5605	Bacillus thuringiensis	GCA_001595725.1_ASM159572v1_genomic.fna
5606	Burkholderia pseudomallei	GCA_000770455.1_ASM77045v1_genomic.fna
5607	Vibrio cholerae	GCA_002073335.1_ASM207333v1_genomic.fna

5608	Francisella tularensis	GCA_001262115.1_ASM126211v1_genomic.fna
5609	Xanthomonas citri	GCA_001922105.1_ASM192210v1_genomic.fna
5610	Bacillus subtilis	GCA_001037985.1_ASM103798v1_genomic.fna
5611	Klebsiella pneumoniae	GCA_000775955.1_ASM77595v1_genomic.fna
5612	Bordetella pertussis	GCA_001605215.1_ASM160521v1_genomic.fna
5613	Mycoplasma pneumoniae	GCA_002096015.1_ASM209601v1_genomic.fna
5614	Lactobacillus plantarum	GCA_002109405.1_ASM210940v1_genomic.fna
5615	Corynebacterium pseudotuberculosis	GCA_001456175.1_ASM145617v1_genomic.fna
5616	Bacillus velezensis	GCA_002157265.1_ASM215726v1_genomic.fna
5617	Bacillus velezensis	GCA_002192235.1_ASM219223v1_genomic.fna
5618	Mycobacterium africanum	GCA_001544855.1_ASM154485v1_genomic.fna
5619	Riemerella anatipestifer	GCA_001051075.1_ASM105107v1_genomic.fna
5620	Corynebacterium pseudotuberculosis	GCA_001481675.1_ASM148167v1_genomic.fna
5621	Lactobacillus plantarum	GCA_002109425.1_ASM210942v1_genomic.fna
5622	Mycoplasma pneumoniae	GCA_002096035.1_ASM209603v1_genomic.fna
5623	Bordetella pertussis	GCA_001605235.1_ASM160523v1_genomic.fna
5624	Klebsiella pneumoniae	GCA_000784945.1_ASM78494v1_genomic.fna
5625	Bacillus subtilis	GCA_001465815.1_ASM146581v1_genomic.fna
5626	Xanthomonas citri	GCA_002139955.1_ASM213995v1_genomic.fna
5627	Francisella tularensis	GCA_001267475.1_ASM126747v1_genomic.fna
5628	Vibrio cholerae	GCA_002313025.1_ASM231302v1_genomic.fna
5629	Burkholderia pseudomallei	GCA_000770495.1_ASM77049v1_genomic.fna
5630	Bacillus thuringiensis	GCA_001598095.1_ASM159809v1_genomic.fna
5631	Chlamydia trachomatis	GCA_000318885.1_ASM31888v1_genomic.fna
5632	Legionella pneumophila	GCA_001752905.1_ASM175290v1_genomic.fna
5633	Acinetobacter baumannii	GCA_001077965.2_ASM107796v2_genomic.fna
5634	Pseudomonas aeruginosa	GCA_001482325.1_ASM148232v1_genomic.fna
5635	Streptococcus agalactiae	GCA_002073695.1_ASM207369v1_genomic.fna
5636	Streptococcus pneumoniae	GCA_001896045.1_ASM189604v1_genomic.fna
5637	Bacillus anthracis	GCA_001654475.1_ASM165447v1_genomic.fna
5638	Streptococcus pyogenes	GCA_001019675.1_ASM101967v1_genomic.fna
5639	Neisseria meningitidis	GCA_001697325.1_ASM169732v1_genomic.fna
5640	Helicobacter pylori	GCA_000270005.1_ASM27000v1_genomic.fna
5641	Escherichia coli	GCA_000025745.1_ASM2574v1_genomic.fna

5642	Mycobacterium tuberculosis	GCA_000827085.1_ASM82708v1_genomic.fna
5643	Listeria monocytogenes	GCA_000438645.1_ASM43864v1_genomic.fna
5644	Lactococcus lactis	GCA_002078995.1_ASM207899v1_genomic.fna
5645	Bacillus cereus	GCA_001635995.1_ASM163599v1_genomic.fna
5646	Salmonella enterica	GCA_000272735.4_ASM27273v4_genomic.fna
5647	Yersinia pestis	GCA_001188815.1_ASM118881v1_genomic.fna
5648	Staphylococcus aureus	GCA_000284535.1_ASM28453v1_genomic.fna
5649	Campylobacter jejuni	GCA_000835345.1_ASM83534v1_genomic.fna
5650	Campylobacter jejuni	GCA_000835365.1_ASM83536v1_genomic.fna
5651	Staphylococcus aureus	GCA_000296595.1_ASM29659v1_genomic.fna
5652	Yersinia pestis	GCA_001188935.1_ASM118893v1_genomic.fna
5653	Salmonella enterica	GCA_000272755.4_ASM27275v4_genomic.fna
5654	Bacillus cereus	GCA_001721145.1_ASM172114v1_genomic.fna
5655	Lactococcus lactis	GCA_002148215.1_ASM214821v1_genomic.fna
5656	Listeria monocytogenes	GCA_000438665.1_ASM43866v1_genomic.fna
5657	Mycobacterium tuberculosis	GCA_000828995.1_ASM82899v1_genomic.fna
5658	Escherichia coli	GCA_000026245.1_ASM2624v1_genomic.fna
5659	Helicobacter pylori	GCA_000270025.1_ASM27002v1_genomic.fna
5660	Neisseria meningitidis	GCA_001697345.1_ASM169734v1_genomic.fna
5661	Streptococcus pyogenes	GCA_001019695.1_ASM101969v1_genomic.fna
5662	Bacillus anthracis	GCA_001683065.1_ASM168306v1_genomic.fna
5663	Streptococcus pneumoniae	GCA_001896065.1_ASM189606v1_genomic.fna
5664	Pseudomonas aeruginosa	GCA_001515845.2_ASM151584v2_genomic.fna
5665	Streptococcus agalactiae	GCA_002197205.1_ASM219720v1_genomic.fna
5666	Acinetobacter baumannii	GCA_001261895.2_R2090_genomic.fna
5667	Legionella pneumophila	GCA_001752925.1_ASM175292v1_genomic.fna
5668	Chlamydia trachomatis	GCA_000318905.1_ASM31890v1_genomic.fna
5669	Bacillus thuringiensis	GCA_001618665.1_ASM161866v1_genomic.fna
5670	Burkholderia pseudomallei	GCA_000770515.1_ASM77051v1_genomic.fna
5671	Francisella tularensis	GCA_001729565.1_ASM172956v1_genomic.fna
5672	Xanthomonas citri	GCA_002139975.1_ASM213997v1_genomic.fna
5673	Bacillus subtilis	GCA_001534785.1_ASM153478v1_genomic.fna
5674	Klebsiella pneumoniae	GCA_000784985.1_ASM78498v1_genomic.fna
5675	Bordetella pertussis	GCA_001605255.1_ASM160525v1_genomic.fna

5676	Mycoplasma pneumoniae	GCA_002127985.1_ASM212798v1_genomic.fna
5677	Lactobacillus plantarum	GCA_002116955.1_ASM211695v1_genomic.fna
5678	Corynebacterium pseudotuberculosis	GCA_001481715.1_ASM148171v1_genomic.fna
5679	Bacillus velezensis	GCA_002205715.1_ASM220571v1_genomic.fna
5680	Bacillus velezensis	GCA_002216755.1_ASM221675v1_genomic.fna
5681	Corynebacterium pseudotuberculosis	GCA_001481755.1_ASM148175v1_genomic.fna
5682	Streptococcus equinus	GCA_002073515.1_ASM207351v1_genomic.fna
5683	Lactobacillus plantarum	GCA_002117245.1_ASM211724v1_genomic.fna
5684	Mycoplasma pneumoniae	GCA_002128005.1_ASM212800v1_genomic.fna
5685	Bordetella pertussis	GCA_001605275.1_ASM160527v1_genomic.fna
5686	Klebsiella pneumoniae	GCA_000785005.1_ASM78500v1_genomic.fna
5687	Bacillus subtilis	GCA_001541905.1_ASM154190v1_genomic.fna
5688	Xanthomonas citri	GCA_002139995.1_ASM213999v1_genomic.fna
5689	Francisella tularensis	GCA_001729585.1_ASM172958v1_genomic.fna
5690	Burkholderia pseudomallei	GCA_000770535.1_ASM77053v1_genomic.fna
5691	Bacillus thuringiensis	GCA_001640965.1_ASM164096v1_genomic.fna
5692	Chlamydia trachomatis	GCA_000318925.1_ASM31892v1_genomic.fna
5693	Legionella pneumophila	GCA_001752945.1_ASM175294v1_genomic.fna
5694	Acinetobacter baumannii	GCA_001399655.1_ASM139965v1_genomic.fna
5695	Streptococcus agalactiae	GCA_002197245.1_ASM219724v1_genomic.fna
5696	Pseudomonas aeruginosa	GCA_001515915.2_ASM151591v2_genomic.fna
5697	Streptococcus pneumoniae	GCA_001896085.1_ASM189608v1_genomic.fna
5698	Bacillus anthracis	GCA_001683095.1_ASM168309v1_genomic.fna
5699	Streptococcus pyogenes	GCA_001020185.2_ASM102018v2_genomic.fna
5700	Neisseria meningitidis	GCA_001697365.1_ASM169736v1_genomic.fna
5701	Helicobacter pylori	GCA_000270045.1_ASM27004v1_genomic.fna
5702	Escherichia coli	GCA_000026265.1_ASM2626v1_genomic.fna
5703	Mycobacterium tuberculosis	GCA_000831245.1_ASM83124v1_genomic.fna
5704	Listeria monocytogenes	GCA_000438685.2_ASM43868v2_genomic.fna
5705	Bacillus cereus	GCA_001880305.1_ASM188030v1_genomic.fna
5706	Lactococcus lactis	GCA_900088425.1_A12_genomic.fna
5707	Salmonella enterica	GCA_000272775.4_ASM27277v4_genomic.fna
5708	Yersinia pestis	GCA_001293415.1_ASM129341v1_genomic.fna
5709	Staphylococcus aureus	GCA_000382965.1_ASM38296v1_genomic.fna

5710	Campylobacter jejuni	GCA_000934305.1_ASM93430v1_genomic.fna
5711	Campylobacter jejuni	GCA_001299565.1_ASM129956v1_genomic.fna
5712	Salmonella enterica	GCA_000272815.2_ASM27281v2_genomic.fna
5713	Staphylococcus aureus	GCA_000382985.1_ASM38298v1_genomic.fna
5714	Yersinia pestis	GCA_001693595.1_ASM169359v1_genomic.fna
5715	Bacillus cereus	GCA_001941885.1_ASM194188v1_genomic.fna
5716	Listeria monocytogenes	GCA_000438705.2_ASM43870v2_genomic.fna
5717	Mycobacterium tuberculosis	GCA_001275565.2_ASM127556v2_genomic.fna
5718	Helicobacter pylori	GCA_000270065.1_ASM27006v1_genomic.fna
5719	Escherichia coli	GCA_000026285.2_ASM2628v2_genomic.fna
5720	Neisseria meningitidis	GCA_001697385.1_ASM169738v1_genomic.fna
5721	Streptococcus pyogenes	GCA_001021955.1_ASM102195v1_genomic.fna
5722	Bacillus anthracis	GCA_001683135.1_ASM168313v1_genomic.fna
5723	Streptococcus pneumoniae	GCA_001902455.1_ASM190245v1_genomic.fna
5724	Streptococcus agalactiae	GCA_002197265.1_ASM219726v1_genomic.fna
5725	Pseudomonas aeruginosa	GCA_001516005.1_ASM151600v1_genomic.fna
5726	Acinetobacter baumannii	GCA_001457535.1_CIP70.10_genomic.fna
5727	Legionella pneumophila	GCA_001752965.1_ASM175296v1_genomic.fna
5728	Chlamydia trachomatis	GCA_000318985.1_ASM31898v1_genomic.fna
5729	Bacillus thuringiensis	GCA_001685565.1_ASM168556v1_genomic.fna
5730	Burkholderia pseudomallei	GCA_000770565.1_ASM77056v1_genomic.fna
5731	Francisella tularensis	GCA_001729605.1_ASM172960v1_genomic.fna
5732	Xanthomonas citri	GCA_002163775.1_ASM216377v1_genomic.fna
5733	Bacillus subtilis	GCA_001565875.1_ASM156587v1_genomic.fna
5734	Klebsiella pneumoniae	GCA_000807395.3_ASM80739v3_genomic.fna
5735	Bordetella pertussis	GCA_001605295.1_ASM160529v1_genomic.fna
5736	Mycoplasma pneumoniae	GCA_002128025.1_ASM212802v1_genomic.fna
5737	Lactobacillus plantarum	GCA_002117265.1_ASM211726v1_genomic.fna
5738	Corynebacterium pseudotuberculosis	GCA_001579825.1_ASM157982v1_genomic.fna
5739	Bacillus velezensis	GCA_002237515.1_ASM223751v1_genomic.fna
5740	Bacillus velezensis	GCA_002238395.1_ASM223839v1_genomic.fna
5741	Corynebacterium pseudotuberculosis	GCA_001579865.1_ASM157986v1_genomic.fna
5742	Lactobacillus plantarum	GCA_002117285.1_ASM211728v1_genomic.fna
5743	Mycoplasma pneumoniae	GCA_002128045.1_ASM212804v1_genomic.fna

5744	Bordetella pertussis	GCA_001605345.1_ASM160534v1_genomic.fna
5745	Klebsiella pneumoniae	GCA_000813205.1_ASM81320v1_genomic.fna
5746	Bacillus subtilis	GCA_001597265.1_ASM159726v1_genomic.fna
5747	Xanthomonas citri	GCA_002218265.1_ASM221826v1_genomic.fna
5748	Francisella tularensis	GCA_001865695.1_ASM186569v1_genomic.fna
5749	Burkholderia pseudomallei	GCA_000953095.1_BP_3921g_genomic.fna
5750	Bacillus thuringiensis	GCA_001692675.1_ASM169267v1_genomic.fna
5751	Chlamydia trachomatis	GCA_000319105.1_ASM31910v1_genomic.fna
5752	Legionella pneumophila	GCA_001753065.1_ASM175306v1_genomic.fna
5753	Acinetobacter baumannii	GCA_001514375.1_ASM151437v1_genomic.fna
5754	Pseudomonas aeruginosa	GCA_001516105.1_ASM151610v1_genomic.fna
5755	Streptococcus agalactiae	GCA_002197285.1_ASM219728v1_genomic.fna
5756	Bacillus anthracis	GCA_001683155.1_ASM168315v1_genomic.fna
5757	Streptococcus pneumoniae	GCA_002357995.1_ASM235799v1_genomic.fna
5758	Streptococcus pyogenes	GCA_001023495.1_ASM102349v1_genomic.fna
5759	Neisseria meningitidis	GCA_001697405.1_ASM169740v1_genomic.fna
5760	Helicobacter pylori	GCA_000277365.1_ASM27736v1_genomic.fna
5761	Mycobacterium tuberculosis	GCA_001544705.1_ASM154470v1_genomic.fna
5762	Escherichia coli	GCA_000026545.1_ASM2654v1_genomic.fna
5763	Listeria monocytogenes	GCA_000438725.2_ASM43872v2_genomic.fna
5764	Bacillus cereus	GCA_001941905.1_ASM194190v1_genomic.fna
5765	Salmonella enterica	GCA_000272835.4_ASM27283v4_genomic.fna
5766	Staphylococcus aureus	GCA_000383005.1_ASM38300v1_genomic.fna
5767	Campylobacter jejuni	GCA_001299595.1_ASM129959v1_genomic.fna
5768	Campylobacter jejuni	GCA_001314285.1_ASM131428v1_genomic.fna
5769	Salmonella enterica	GCA_000272895.3_ASM27289v3_genomic.fna
5770	Staphylococcus aureus	GCA_000412775.1_ASM41277v1_genomic.fna
5771	Bacillus cereus	GCA_001941925.1_ASM194192v1_genomic.fna
5772	Listeria monocytogenes	GCA_000438745.2_ASM43874v2_genomic.fna
5773	Escherichia coli	GCA_000027125.1_ASM2712v1_genomic.fna
5774	Mycobacterium tuberculosis	GCA_001544955.1_ASM154495v1_genomic.fna
5775	Helicobacter pylori	GCA_000277385.1_ASM27738v1_genomic.fna
5776	Neisseria meningitidis	GCA_001697425.1_ASM169742v1_genomic.fna
5777	Streptococcus pyogenes	GCA_001039695.2_emm89-1_genomic.fna

5778	Bacillus anthracis	GCA_001683175.1_ASM168317v1_genomic.fna
5779	Pseudomonas aeruginosa	GCA_001516165.2_ASM151616v2_genomic.fna
5780	Streptococcus agalactiae	GCA_002197305.1_ASM219730v1_genomic.fna
5781	Acinetobacter baumannii	GCA_001517645.1_R2091_genomic.fna
5782	Legionella pneumophila	GCA_001753085.1_ASM175308v1_genomic.fna
5783	Chlamydia trachomatis	GCA_000364765.1_ASM36476v1_genomic.fna
5784	Burkholderia pseudomallei	GCA_000954175.1_ASM95417v1_genomic.fna
5785	Bacillus thuringiensis	GCA_001721165.1_ASM172116v1_genomic.fna
5786	Xanthomonas citri	GCA_002218285.1_ASM221828v1_genomic.fna
5787	Bacillus subtilis	GCA_001604995.1_ASM160499v1_genomic.fna
5788	Klebsiella pneumoniae	GCA_000814305.1_ASM81430v1_genomic.fna
5789	Bordetella pertussis	GCA_001605365.1_ASM160536v1_genomic.fna
5790	Mycoplasma pneumoniae	GCA_002128065.1_ASM212806v1_genomic.fna
5791	Lactobacillus plantarum	GCA_002117305.1_ASM211730v1_genomic.fna
5792	Achromobacter xylosoxidans	GCA_001558915.1_ASM155891v1_genomic.fna
5793	Corynebacterium pseudotuberculosis	GCA_001579885.2_ASM157988v2_genomic.fna
5794	Bacillus velezensis	GCA_002243325.2_ASM224332v2_genomic.fna
5795	Bacillus velezensis	GCA_002310315.1_ASM231031v1_genomic.fna
5796	Corynebacterium pseudotuberculosis	GCA_001579925.1_ASM157992v1_genomic.fna
5797	Achromobacter xylosoxidans	GCA_001559195.1_ASM155919v1_genomic.fna
5798	Lactobacillus plantarum	GCA_002173655.1_ASM217365v1_genomic.fna
5799	Mycoplasma pneumoniae	GCA_002128085.1_ASM212808v1_genomic.fna
5800	Bordetella pertussis	GCA_001605385.1_ASM160538v1_genomic.fna
5801	Klebsiella pneumoniae	GCA_000814805.1_ASM81480v1_genomic.fna
5802	Bacillus subtilis	GCA_001660525.1_ASM166052v1_genomic.fna
5803	Xanthomonas citri	GCA_002224525.1_ASM222452v1_genomic.fna
5804	Bacillus thuringiensis	GCA_002025105.1_ASM202510v1_genomic.fna
5805	Burkholderia pseudomallei	GCA_000959125.1_ASM95912v1_genomic.fna
5806	Chlamydia trachomatis	GCA_000364785.1_ASM36478v1_genomic.fna
5807	Legionella pneumophila	GCA_001753105.1_ASM175310v1_genomic.fna
5808	Acinetobacter baumannii	GCA_001543995.1_ASM154399v1_genomic.fna
5809	Streptococcus agalactiae	GCA_002197325.1_ASM219732v1_genomic.fna
5810	Pseudomonas aeruginosa	GCA_001516185.2_ASM151618v2_genomic.fna
5811	Bacillus anthracis	GCA_001683195.1_ASM168319v1_genomic.fna

5812	Streptococcus pyogenes	GCA_001051095.1_ASM105109v1_genomic.fna
5813	Neisseria meningitidis	GCA_001697445.1_ASM169744v1_genomic.fna
5814	Helicobacter pylori	GCA_000277405.1_ASM27740v1_genomic.fna
5815	Mycobacterium tuberculosis	GCA_001545015.1_ASM154501v1_genomic.fna
5816	Escherichia coli	GCA_000091005.1_ASM9100v1_genomic.fna
5817	Listeria monocytogenes	GCA_000513595.1_ASM51359v1_genomic.fna
5818	Bacillus cereus	GCA_002000005.1_ASM200000v1_genomic.fna
5819	Staphylococcus aureus	GCA_000418345.1_ASM41834v1_genomic.fna
5820	Salmonella enterica	GCA_000280315.2_ASM28031v2_genomic.fna
5821	Campylobacter jejuni	GCA_001412295.1_ASM141229v1_genomic.fna
5822	Salmonella enterica	GCA_000283735.1_ASM28373v1_genomic.fna
5823	Campylobacter jejuni	GCA_001457695.1_NCTC11351_genomic.fna
5824	Staphylococcus aureus	GCA_000452385.2_ASM45238v2_genomic.fna
5825	Bacillus cereus	GCA_002214705.1_ASM221470v1_genomic.fna
5826	Listeria monocytogenes	GCA_000513615.1_ASM51361v1_genomic.fna
5827	Escherichia coli	GCA_000147855.3_ASM14785v3_genomic.fna
5828	Mycobacterium tuberculosis	GCA_001702435.1_ASM170243v1_genomic.fna
5829	Helicobacter pylori	GCA_000277425.1_ASM27742v1_genomic.fna
5830	Neisseria meningitidis	GCA_001697465.1_ASM169746v1_genomic.fna
5831	Streptococcus pyogenes	GCA_001267805.1_ASM126780v1_genomic.fna
5832	Bacillus anthracis	GCA_001683215.1_ASM168321v1_genomic.fna
5833	Pseudomonas aeruginosa	GCA_001516205.2_ASM151620v2_genomic.fna
5834	Streptococcus agalactiae	GCA_002197365.1_ASM219736v1_genomic.fna
5835	Acinetobacter baumannii	GCA_001573065.1_ASM157306v1_genomic.fna
5836	Legionella pneumophila	GCA_001753125.1_ASM175312v1_genomic.fna
5837	Chlamydia trachomatis	GCA_000441585.1_ASM44158v1_genomic.fna
5838	Burkholderia pseudomallei	GCA_000959145.1_ASM95914v1_genomic.fna
5839	Bacillus thuringiensis	GCA_002119445.1_ASM211944v1_genomic.fna
5840	Xanthomonas citri	GCA_002224545.1_ASM222454v1_genomic.fna
5841	Bacillus subtilis	GCA_001697265.1_ASM169726v1_genomic.fna
5842	Klebsiella pneumoniae	GCA_000968155.1_ASM96815v1_genomic.fna
5843	Bordetella pertussis	GCA_001605405.1_ASM160540v1_genomic.fna
5844	Mycoplasma pneumoniae	GCA_002128105.1_ASM212810v1_genomic.fna
5845	Lactobacillus plantarum	GCA_002174195.1_ASM217419v1_genomic.fna

5846	Corynebacterium pseudotuberculosis	GCA_001653295.1_ASM165329v1_genomic.fna
5847	Aeromonas veronii	GCA_001634345.1_ASM163434v1_genomic.fna
5848	Bacillus velezensis	GCA_002327165.1_ASM232716v1_genomic.fna
5849	Corynebacterium pseudotuberculosis	GCA_001653315.1_ASM165331v1_genomic.fna
5850	Aggregatibacter actinomycetemcomitans	GCA_000241025.2_ASM24102v2_genomic.fna
5851	Lactobacillus plantarum	GCA_002205775.2_ASM220577v2_genomic.fna
5852	Mycoplasma pneumoniae	GCA_002128125.1_ASM212812v1_genomic.fna
5853	Bordetella pertussis	GCA_001605425.1_ASM160542v1_genomic.fna
5854	Haemophilus parasuis	GCA_000439395.1_ASM43939v1_genomic.fna
5855	Klebsiella pneumoniae	GCA_000981845.1_ASM98184v1_genomic.fna
5856	Bacillus subtilis	GCA_001703495.1_ASM170349v1_genomic.fna
5857	Xanthomonas citri	GCA_002288565.1_ASM228856v1_genomic.fna
5858	Bacillus thuringiensis	GCA_002173755.1_ASM217375v1_genomic.fna
5859	Burkholderia pseudomallei	GCA_000959185.1_ASM95918v1_genomic.fna
5860	Chlamydia trachomatis	GCA_000441615.1_ASM44161v1_genomic.fna
5861	Legionella pneumophila	GCA_001753145.1_ASM175314v1_genomic.fna
5862	Acinetobacter baumannii	GCA_001573085.1_ASM157308v1_genomic.fna
5863	Streptococcus agalactiae	GCA_002197385.1_ASM219738v1_genomic.fna
5864	Pseudomonas aeruginosa	GCA_001516225.2_ASM151622v2_genomic.fna
5865	Bacillus anthracis	GCA_001683235.1_ASM168323v1_genomic.fna
5866	Streptococcus pyogenes	GCA_001267845.1_ASM126784v1_genomic.fna
5867	Neisseria meningitidis	GCA_001697485.1_ASM169748v1_genomic.fna
5868	Helicobacter pylori	GCA_000307795.1_ASM30779v1_genomic.fna
5869	Mycobacterium tuberculosis	GCA_001708265.1_ASM170826v1_genomic.fna
5870	Escherichia coli	GCA_000148365.1_ASM14836v1_genomic.fna
5871	Listeria monocytogenes	GCA_000513635.1_ASM51363v1_genomic.fna
5872	Bacillus cereus	GCA_002214725.1_ASM221472v1_genomic.fna
5873	Staphylococcus aureus	GCA_000462955.1_ASM46295v1_genomic.fna
5874	Campylobacter jejuni	GCA_001506185.1_ASM150618v1_genomic.fna
5875	Salmonella enterica	GCA_000329365.2_ASM32936v2_genomic.fna
5876	Salmonella enterica	GCA_000330485.2_ASM33048v2_genomic.fna
5877	Campylobacter jejuni	GCA_001506205.1_ASM150620v1_genomic.fna
5878	Staphylococcus aureus	GCA_000463055.1_ASM46305v1_genomic.fna
5879	Bacillus cereus	GCA_002214765.1_ASM221476v1_genomic.fna

5880	Listeria monocytogenes	GCA_000513655.1_ASM51365v1_genomic.fna
5881	Escherichia coli	GCA_000148605.1_ASM14860v1_genomic.fna
5882	Prochlorococcus marinus	GCA_000015705.1_ASM1570v1_genomic.fna
5883	Mycobacterium tuberculosis	GCA_001750865.1_ASM175086v1_genomic.fna
5884	Helicobacter pylori	GCA_000307815.1_ASM30781v1_genomic.fna
5885	Neisseria meningitidis	GCA_001697505.1_ASM169750v1_genomic.fna
5886	Streptococcus pyogenes	GCA_001535565.1_ASM153556v1_genomic.fna
5887	Bacillus anthracis	GCA_001683255.1_ASM168325v1_genomic.fna
5888	Pseudomonas aeruginosa	GCA_001516245.2_ASM151624v2_genomic.fna
5889	Streptococcus agalactiae	GCA_002197425.1_ASM219742v1_genomic.fna
5890	Acinetobacter baumannii	GCA_001573105.1_ASM157310v1_genomic.fna
5891	Legionella pneumophila	GCA_001753265.1_ASM175326v1_genomic.fna
5892	Chlamydia trachomatis	GCA_000441635.1_ASM44163v1_genomic.fna
5893	Burkholderia pseudomallei	GCA_000959205.1_ASM95920v1_genomic.fna
5894	Bacillus thuringiensis	GCA_002184245.1_ASM218424v1_genomic.fna
5895	Xanthomonas citri	GCA_002288585.1_ASM228858v1_genomic.fna
5896	Bacillus subtilis	GCA_001704095.1_ASM170409v1_genomic.fna
5897	Klebsiella pneumoniae	GCA_001022035.1_ASM102203v1_genomic.fna
5898	Bordetella pertussis	GCA_001605445.1_ASM160544v1_genomic.fna
5899	Mycoplasma pneumoniae	GCA_002128145.1_ASM212814v1_genomic.fna
5900	Lactobacillus plantarum	GCA_002220175.1_ASM222017v1_genomic.fna
5901	Aggregatibacter actinomycetemcomitans	GCA_001547775.1_ASM154777v1_genomic.fna
5902	Corynebacterium pseudotuberculosis	GCA_001663495.1_ASM166349v1_genomic.fna
5903	Corynebacterium pseudotuberculosis	GCA_001682255.1_ASM168225v1_genomic.fna
5904	Lactobacillus plantarum	GCA_002220815.1_ASM222081v1_genomic.fna
5905	Mycoplasma pneumoniae	GCA_002128165.1_ASM212816v1_genomic.fna
5906	Bordetella pertussis	GCA_001605465.1_ASM160546v1_genomic.fna
5907	Klebsiella pneumoniae	GCA_001022175.1_ASM102217v1_genomic.fna
5908	Bacillus subtilis	GCA_001720505.1_ASM172050v1_genomic.fna
5909	Bacillus thuringiensis	GCA_002192515.1_ASM219251v1_genomic.fna
5910	Burkholderia pseudomallei	GCA_000959225.1_ASM95922v1_genomic.fna
5911	Chlamydia trachomatis	GCA_000441655.1_ASM44165v1_genomic.fna
5912	Legionella pneumophila	GCA_001753285.1_ASM175328v1_genomic.fna
5913	Acinetobacter baumannii	GCA_001573125.1_ASM157312v1_genomic.fna

5914	Streptococcus agalactiae	GCA_002214425.1_ASM221442v1_genomic.fna
5915	Pseudomonas aeruginosa	GCA_001516265.1_ASM151626v1_genomic.fna
5916	Bacillus anthracis	GCA_001683275.1_ASM168327v1_genomic.fna
5917	Streptococcus pyogenes	GCA_001547715.1_ASM154771v1_genomic.fna
5918	Neisseria meningitidis	GCA_001697525.1_ASM169752v1_genomic.fna
5919	Helicobacter pylori	GCA_000307835.1_ASM30783v1_genomic.fna
5920	Mycobacterium tuberculosis	GCA_001855255.1_ASM185525v1_genomic.fna
5921	Escherichia coli	GCA_000184185.1_ASM18418v1_genomic.fna
5922	Listeria monocytogenes	GCA_000513675.1_ASM51367v1_genomic.fna
5923	Bacillus cereus	GCA_002215175.1_ASM221517v1_genomic.fna
5924	Campylobacter jejuni	GCA_001506225.1_ASM150622v1_genomic.fna
5925	Salmonella enterica	GCA_000335875.2_ASM33587v2_genomic.fna
5926	Staphylococcus aureus	GCA_000470845.1_ASM47084v1_genomic.fna
5927	Staphylococcus aureus	GCA_000470865.1_ASM47086v1_genomic.fna
5928	Salmonella enterica	GCA_000335995.2_ASM33599v2_genomic.fna
5929	Campylobacter jejuni	GCA_001506245.1_ASM150624v1_genomic.fna
5930	Bacillus cereus	GCA_002216125.1_ASM221612v1_genomic.fna
5931	Listeria monocytogenes	GCA_000513695.1_ASM51369v1_genomic.fna
5932	Escherichia coli	GCA_000210475.1_ASM21047v1_genomic.fna
5933	Mycobacterium tuberculosis	GCA_001870145.1_ASM187014v1_genomic.fna
5934	Helicobacter pylori	GCA_000315955.1_ASM31595v1_genomic.fna
5935	Neisseria meningitidis	GCA_001697545.1_ASM169754v1_genomic.fna
5936	Streptococcus pyogenes	GCA_001547835.1_ASM154783v1_genomic.fna
5937	Bacillus anthracis	GCA_001683295.1_ASM168329v1_genomic.fna
5938	Pseudomonas aeruginosa	GCA_001516305.2_ASM151630v2_genomic.fna
5939	Streptococcus agalactiae	GCA_002289205.1_ASM228920v1_genomic.fna
5940	Acinetobacter baumannii	GCA_001578145.1_ASM157814v1_genomic.fna
5941	Legionella pneumophila	GCA_001753305.1_ASM175330v1_genomic.fna
5942	Chlamydia trachomatis	GCA_000441675.1_ASM44167v1_genomic.fna
5943	Burkholderia pseudomallei	GCA_000959265.1_ASM95926v1_genomic.fna
5944	Bacillus thuringiensis	GCA_002222555.1_ASM222255v1_genomic.fna
5945	Bacillus subtilis	GCA_001746575.1_ASM174657v1_genomic.fna
5946	Klebsiella pneumoniae	GCA_001022235.1_ASM102223v1_genomic.fna
5947	Bordetella pertussis	GCA_001605485.1_ASM160548v1_genomic.fna

5948	Mycoplasma pneumoniae	GCA_002128185.1_ASM212818v1_genomic.fna
5949	Lactobacillus plantarum	GCA_002286275.1_ASM228627v1_genomic.fna
5950	Burkholderia vietnamiensis	GCA_000016205.1_ASM1620v1_genomic.fna
5951	Staphylococcus saprophyticus	GCA_002208905.1_ASM220890v1_genomic.fna
5952	Corynebacterium pseudotuberculosis	GCA_001682275.1_ASM168227v1_genomic.fna
5953	Corynebacterium pseudotuberculosis	GCA_001865765.1_ASM186576v1_genomic.fna
5954	Staphylococcus saprophyticus	GCA_002209265.1_ASM220926v1_genomic.fna
5955	Lactobacillus plantarum	GCA_002290185.1_ASM229018v1_genomic.fna
5956	Mycoplasma pneumoniae	GCA_002128205.1_ASM212820v1_genomic.fna
5957	Bordetella pertussis	GCA_001605505.1_ASM160550v1_genomic.fna
5958	Rhizobium leguminosarum	GCA_001679785.1_ASM167978v1_genomic.fna
5959	Klebsiella pneumoniae	GCA_001280925.1_ASM128092v1_genomic.fna
5960	Bacillus subtilis	GCA_001747445.1_ASM174744v1_genomic.fna
5961	Bacillus thuringiensis	GCA_002243685.1_ASM224368v1_genomic.fna
5962	Burkholderia pseudomallei	GCA_000959285.1_ASM95928v1_genomic.fna
5963	Chlamydia trachomatis	GCA_000441695.1_ASM44169v1_genomic.fna
5964	Legionella pneumophila	GCA_001753325.1_ASM175332v1_genomic.fna
5965	Acinetobacter baumannii	GCA_001593425.2_ASM159342v2_genomic.fna
5966	Streptococcus agalactiae	GCA_900078265.1_SA111_genomic.fna
5967	Pseudomonas aeruginosa	GCA_001516325.2_ASM151632v2_genomic.fna
5968	Bacillus anthracis	GCA_001936375.1_ASM193637v1_genomic.fna
5969	Streptococcus pyogenes	GCA_001559175.1_ASM155917v1_genomic.fna
5970	Neisseria meningitidis	GCA_001697565.1_ASM169756v1_genomic.fna
5971	Helicobacter pylori	GCA_000317875.1_ASM31787v1_genomic.fna
5972	Mycobacterium tuberculosis	GCA_001895765.1_ASM189576v1_genomic.fna
5973	Escherichia coli	GCA_000212715.2_ASM21271v2_genomic.fna
5974	Listeria monocytogenes	GCA_000568475.1_ASM56847v1_genomic.fna
5975	Bacillus cereus	GCA_002220285.1_ASM222028v1_genomic.fna
5976	Campylobacter jejuni	GCA_001506265.1_ASM150626v1_genomic.fna
5977	Salmonella enterica	GCA_000341425.1_ASM34142v1_genomic.fna
5978	Staphylococcus aureus	GCA_000485885.1_ASM48588v1_genomic.fna
5979	Staphylococcus aureus	GCA_000568455.1_ASM56845v1_genomic.fna
5980	Salmonella enterica	GCA_000380325.1_ASM38032v1_genomic.fna
5981	Campylobacter jejuni	GCA_001506285.1_ASM150628v1_genomic.fna

5982	Bacillus cereus	GCA_002224345.1_ASM222434v1_genomic.fna
5983	Listeria monocytogenes	GCA_000568935.1_ASM56893v1_genomic.fna
5984	Escherichia coli	GCA_000219515.3_ASM21951v3_genomic.fna
5985	Mycobacterium tuberculosis	GCA_001895785.1_ASM189578v1_genomic.fna
5986	Helicobacter pylori	GCA_000348865.1_ASM34886v1_genomic.fna
5987	Neisseria meningitidis	GCA_001697585.1_ASM169758v1_genomic.fna
5988	Streptococcus pyogenes	GCA_001620285.1_ASM162028v1_genomic.fna
5989	Bacillus anthracis	GCA_001990245.1_ASM199024v1_genomic.fna
5990	Streptococcus agalactiae	GCA_900155855.1_BM110_genomic.fna
5991	Pseudomonas aeruginosa	GCA_001516345.1_ASM151634v1_genomic.fna
5992	Acinetobacter baumannii	GCA_001636235.1_ASM163623v1_genomic.fna
5993	Legionella pneumophila	GCA_001753345.1_ASM175334v1_genomic.fna
5994	Chlamydia trachomatis	GCA_000441715.1_ASM44171v1_genomic.fna
5995	Burkholderia pseudomallei	GCA_000959305.1_ASM95930v1_genomic.fna
5996	Bacillus subtilis	GCA_001808235.1_ASM180823v1_genomic.fna
5997	Klebsiella pneumoniae	GCA_001307175.1_ASM130717v1_genomic.fna
5998	Bordetella pertussis	GCA_001605525.1_ASM160552v1_genomic.fna
5999	Mycoplasma pneumoniae	GCA_002128235.1_ASM212823v1_genomic.fna
6000	Lactobacillus plantarum	GCA_002576835.1_ASM257683v1_genomic.fna
6001	Corynebacterium pseudotuberculosis	GCA_001866985.1_ASM186698v1_genomic.fna
6002	Corynebacterium pseudotuberculosis	GCA_001867005.1_ASM186700v1_genomic.fna
6003	Mycoplasma pneumoniae	GCA_002128265.1_ASM212826v1_genomic.fna
6004	Bordetella pertussis	GCA_001605545.1_ASM160554v1_genomic.fna
6005	Klebsiella pneumoniae	GCA_001455995.1_MS6671.v1_genomic.fna
6006	Bacillus subtilis	GCA_001889385.1_ASM188938v1_genomic.fna
6007	Burkholderia pseudomallei	GCA_000959345.1_ASM95934v1_genomic.fna
6008	Chlamydia trachomatis	GCA_000441735.1_ASM44173v1_genomic.fna
6009	Legionella pneumophila	GCA_001753365.1_ASM175336v1_genomic.fna
6010	Acinetobacter baumannii	GCA_001806345.1_ASM180634v1_genomic.fna
6011	Pseudomonas aeruginosa	GCA_001516365.2_ASM151636v2_genomic.fna
6012	Bacillus anthracis	GCA_002277915.1_ASM227791v1_genomic.fna
6013	Streptococcus pyogenes	GCA_001635895.1_ASM163589v1_genomic.fna
6014	Neisseria meningitidis	GCA_001697605.1_ASM169760v1_genomic.fna
6015	Helicobacter pylori	GCA_000348885.1_ASM34888v1_genomic.fna

6016	Mycobacterium tuberculosis	GCA_001895805.1_ASM189580v1_genomic.fna
6017	Escherichia coli	GCA_000227625.1_ASM22762v1_genomic.fna
6018	Listeria monocytogenes	GCA_000577745.1_6179_18022011_genomic.fna
6019	Bacillus cereus	GCA_002290105.1_ASM229010v1_genomic.fna
6020	Campylobacter jejuni	GCA_001506305.1_ASM150630v1_genomic.fna
6021	Salmonella enterica	GCA_000385905.1_ASM38590v1_genomic.fna
6022	Staphylococcus aureus	GCA_000597965.1_ASM59796v1_genomic.fna
6023	Staphylococcus aureus	GCA_000626615.1_ASM62661v1_genomic.fna
6024	Salmonella enterica	GCA_000430085.2_ASM43008v2_genomic.fna
6025	Campylobacter jejuni	GCA_001506345.1_ASM150634v1_genomic.fna
6026	Listeria monocytogenes	GCA_000582845.1_SV_1_genomic.fna
6027	Escherichia coli	GCA_000233875.1_ASM23387v1_genomic.fna
6028	Mycobacterium tuberculosis	GCA_001895825.1_ASM189582v1_genomic.fna
6029	Helicobacter pylori	GCA_000392455.3_ASM39245v3_genomic.fna
6030	Neisseria meningitidis	GCA_001697625.1_ASM169762v1_genomic.fna
6031	Streptococcus pyogenes	GCA_001635935.1_ASM163593v1_genomic.fna
6032	Bacillus anthracis	GCA_002356575.1_ASM235657v1_genomic.fna
6033	Pseudomonas aeruginosa	GCA_001516385.1_ASM151638v1_genomic.fna
6034	Acinetobacter baumannii	GCA_001806365.1_ASM180636v1_genomic.fna
6035	Legionella pneumophila	GCA_001753385.1_ASM175338v1_genomic.fna
6036	Chlamydia trachomatis	GCA_000441755.1_ASM44175v1_genomic.fna
6037	Burkholderia pseudomallei	GCA_001277875.1_ASM127787v1_genomic.fna
6038	Bacillus subtilis	GCA_001889625.1_ASM188962v1_genomic.fna
6039	Klebsiella pneumoniae	GCA_001456055.3_ASM145605v3_genomic.fna
6040	Bordetella pertussis	GCA_001605565.1_ASM160556v1_genomic.fna
6041	Mycoplasma pneumoniae	GCA_002128285.1_ASM212828v1_genomic.fna
6042	Pectobacterium carotovorum	GCA_000769535.1_ASM76953v1_genomic.fna
6043	Corynebacterium pseudotuberculosis	GCA_001867025.1_ASM186702v1_genomic.fna
6044	Kingella kingae	GCA_001458475.1_KKKWG1_genomic.fna
6045	Corynebacterium pseudotuberculosis	GCA_001867045.1_ASM186704v1_genomic.fna
6046	Mycoplasma pneumoniae	GCA_002147855.1_ASM214785v1_genomic.fna
6047	Bordetella pertussis	GCA_001605585.1_ASM160558v1_genomic.fna
6048	Klebsiella pneumoniae	GCA_001456095.3_ASM145609v3_genomic.fna
6049	Bacillus subtilis	GCA_001890405.1_ASM189040v1_genomic.fna

6050	Burkholderia pseudomallei	GCA_001277895.1_ASM127789v1_genomic.fna
6051	Chlamydia trachomatis	GCA_000441775.1_ASM44177v1_genomic.fna
6052	Legionella pneumophila	GCA_001753405.1_ASM175340v1_genomic.fna
6053	Acinetobacter baumannii	GCA_001806385.1_ASM180638v1_genomic.fna
6054	Pseudomonas aeruginosa	GCA_001518975.1_ASM151897v1_genomic.fna
6055	Streptococcus pyogenes	GCA_001635975.1_ASM163597v1_genomic.fna
6056	Neisseria meningitidis	GCA_001697645.1_ASM169764v1_genomic.fna
6057	Helicobacter pylori	GCA_000392475.3_ASM39247v3_genomic.fna
6058	Mycobacterium tuberculosis	GCA_001895845.1_ASM189584v1_genomic.fna
6059	Escherichia coli	GCA_000233895.1_ASM23389v1_genomic.fna
6060	Listeria monocytogenes	GCA_000600015.1_ASM60001v1_genomic.fna
6061	Campylobacter jejuni	GCA_001506365.1_ASM150636v1_genomic.fna
6062	Staphylococcus aureus	GCA_000695215.1_ASM69521v1_genomic.fna
6063	Salmonella enterica	GCA_000430105.1_ASM43010v1_genomic.fna
6064	Salmonella enterica	GCA_000430125.1_ASM43012v1_genomic.fna
6065	Campylobacter jejuni	GCA_001506385.1_ASM150638v1_genomic.fna
6066	Staphylococcus aureus	GCA_000695875.1_ASM69587v1_genomic.fna
6067	Listeria monocytogenes	GCA_000613085.1_R479A_18022011_genomic.fna
6068	Escherichia coli	GCA_000245515.1_ASM24551v1_genomic.fna
6069	Mycobacterium tuberculosis	GCA_001895865.1_ASM189586v1_genomic.fna
6070	Helicobacter pylori	GCA_000392515.3_ASM39251v3_genomic.fna
6071	Neisseria meningitidis	GCA_001697665.1_ASM169766v1_genomic.fna
6072	Streptococcus pyogenes	GCA_001638845.2_ASM163884v2_genomic.fna
6073	Pseudomonas aeruginosa	GCA_001542795.2_ASM154279v2_genomic.fna
6074	Acinetobacter baumannii	GCA_001806405.1_ASM180640v1_genomic.fna
6075	Legionella pneumophila	GCA_001766275.1_ASM176627v1_genomic.fna
6076	Chlamydia trachomatis	GCA_000441795.1_ASM44179v1_genomic.fna
6077	Burkholderia pseudomallei	GCA_001277975.1_ASM127797v1_genomic.fna
6078	Bacillus subtilis	GCA_001902555.1_ASM190255v1_genomic.fna
6079	Porphyromonas gingivalis	GCA_001444325.1_ASM144432v1_genomic.fna
6080	Klebsiella pneumoniae	GCA_001456135.1_ASM145613v1_genomic.fna
6081	Bordetella pertussis	GCA_001605605.1_ASM160560v1_genomic.fna
6082	Mycoplasma pneumoniae	GCA_002355695.1_ASM235569v1_genomic.fna
6083	Corynebacterium pseudotuberculosis	GCA_001867065.1_ASM186706v1_genomic.fna

6084	Corynebacterium pseudotuberculosis	GCA_001867085.1_ASM186708v1_genomic.fna
6085	Mycoplasma pneumoniae	GCA_002355715.1_ASM235571v1_genomic.fna
6086	Bordetella pertussis	GCA_001605625.1_ASM160562v1_genomic.fna
6087	Klebsiella pneumoniae	GCA_001482345.1_ASM148234v1_genomic.fna
6088	Bacillus subtilis	GCA_002055965.1_ASM205596v1_genomic.fna
6089	Burkholderia pseudomallei	GCA_001318245.1_ASM131824v1_genomic.fna
6090	Chlamydia trachomatis	GCA_000441815.1_ASM44181v1_genomic.fna
6091	Legionella pneumophila	GCA_001766295.1_ASM176629v1_genomic.fna
6092	Acinetobacter baumannii	GCA_001806425.1_ASM180642v1_genomic.fna
6093	Pseudomonas aeruginosa	GCA_001542835.1_ASM154283v1_genomic.fna
6094	Streptococcus pyogenes	GCA_001678885.1_ASM167888v1_genomic.fna
6095	Neisseria meningitidis	GCA_001697685.1_ASM169768v1_genomic.fna
6096	Helicobacter pylori	GCA_000392535.3_ASM39253v3_genomic.fna
6097	Mycobacterium tuberculosis	GCA_001922485.1_ASM192248v1_genomic.fna
6098	Escherichia coli	GCA_000257275.1_ASM25727v1_genomic.fna
6099	Listeria monocytogenes	GCA_000681515.1_ASM68151v1_genomic.fna
6100	Staphylococcus aureus	GCA_000737615.1_ASM73761v1_genomic.fna
6101	Campylobacter jejuni	GCA_001506405.1_ASM150640v1_genomic.fna
6102	Salmonella enterica	GCA_000430145.3_ASM43014v3_genomic.fna
6103	Campylobacter jejuni	GCA_001506425.1_ASM150642v1_genomic.fna
6104	Salmonella enterica	GCA_000430165.1_ASM43016v1_genomic.fna
6105	Staphylococcus aureus	GCA_000746505.1_ASM74650v1_genomic.fna
6106	Listeria monocytogenes	GCA_000746625.1_ASM74662v1_genomic.fna
6107	Escherichia coli	GCA_000258025.1_ASM25802v1_genomic.fna
6108	Mycobacterium tuberculosis	GCA_001938725.1_ASM193872v1_genomic.fna
6109	Helicobacter pylori	GCA_000439295.2_ASM43929v2_genomic.fna
6110	Neisseria meningitidis	GCA_001697705.1_ASM169770v1_genomic.fna
6111	Streptococcus pyogenes	GCA_002055535.1_NCTC8198_genomic.fna
6112	Pseudomonas aeruginosa	GCA_001547955.1_ASM154795v1_genomic.fna
6113	Acinetobacter baumannii	GCA_001806445.1_ASM180644v1_genomic.fna
6114	Legionella pneumophila	GCA_001766315.1_ASM176631v1_genomic.fna
6115	Chlamydia trachomatis	GCA_000507225.1_ASM50722v1_genomic.fna
6116	Burkholderia pseudomallei	GCA_001695715.1_ASM169571v1_genomic.fna
6117	Coxiella burnetii	GCA_000359545.5_G255_GS_genomic.fna

6118	Bacillus subtilis	GCA_002072735.1_ASM207273v1_genomic.fna
6119	Klebsiella pneumoniae	GCA_001521895.1_ASM152189v1_genomic.fna
6120	Bordetella pertussis	GCA_001605645.1_ASM160564v1_genomic.fna
6121	Corynebacterium pseudotuberculosis	GCA_001867105.1_ASM186710v1_genomic.fna
6122	Enterobacter asburiae	GCA_001617645.1_ASM161764v1_genomic.fna
6123	Corynebacterium pseudotuberculosis	GCA_001867125.1_ASM186712v1_genomic.fna
6124	Bordetella pertussis	GCA_001605665.1_ASM160566v1_genomic.fna
6125	Treponema pallidum	GCA_000008605.1_ASM860v1_genomic.fna
6126	Klebsiella pneumoniae	GCA_001529935.1_ASM152993v1_genomic.fna
6127	Bacillus subtilis	GCA_002096095.1_ASM209609v1_genomic.fna
6128	Burkholderia pseudomallei	GCA_001695735.1_ASM169573v1_genomic.fna
6129	Chlamydia trachomatis	GCA_000590575.1_ASM59057v1_genomic.fna
6130	Legionella pneumophila	GCA_001766335.1_ASM176633v1_genomic.fna
6131	Acinetobacter baumannii	GCA_001806465.1_ASM180646v1_genomic.fna
6132	Pseudomonas aeruginosa	GCA_001548135.1_ASM154813v1_genomic.fna
6133	Streptococcus pyogenes	GCA_002163545.1_ASM216354v1_genomic.fna
6134	Neisseria meningitidis	GCA_001697725.1_ASM169772v1_genomic.fna
6135	Helicobacter pylori	GCA_000498315.1_ASM49831v1_genomic.fna
6136	Escherichia coli	GCA_000258145.1_ASM25814v1_genomic.fna
6137	Mycobacterium tuberculosis	GCA_002072775.2_ASM207277v2_genomic.fna
6138	Listeria monocytogenes	GCA_000800335.1_ASM80033v1_genomic.fna
6139	Staphylococcus aureus	GCA_000756205.1_ASM75620v1_genomic.fna
6140	Salmonella enterica	GCA_000439415.1_ASM43941v1_genomic.fna
6141	Campylobacter jejuni	GCA_001506445.1_ASM150644v1_genomic.fna
6142	Campylobacter jejuni	GCA_001506465.1_ASM150646v1_genomic.fna
6143	Salmonella enterica	GCA_000442415.1_ASM44241v1_genomic.fna
6144	Staphylococcus aureus	GCA_000769575.1_ASM76957v1_genomic.fna
6145	Listeria monocytogenes	GCA_000808055.1_ASM80805v1_genomic.fna
6146	Escherichia coli	GCA_000262125.1_ASM26212v1_genomic.fna
6147	Mycobacterium tuberculosis	GCA_002116755.1_ASM211675v1_genomic.fna
6148	Helicobacter pylori	GCA_000498335.1_ASM49833v1_genomic.fna
6149	Neisseria meningitidis	GCA_001697745.1_ASM169774v1_genomic.fna
6150	Streptococcus pyogenes	GCA_002236855.1_ASM223685v1_genomic.fna
6151	Pseudomonas aeruginosa	GCA_001548335.1_ASM154833v1_genomic.fna

6152	Acinetobacter baumannii	GCA_001806485.1_ASM180648v1_genomic.fna
6153	Legionella pneumophila	GCA_001766355.1_ASM176635v1_genomic.fna
6154	Chlamydia trachomatis	GCA_000590615.1_ASM59061v1_genomic.fna
6155	Burkholderia pseudomallei	GCA_001885195.1_ASM188519v1_genomic.fna
6156	Bacillus subtilis	GCA_002142595.1_ASM214259v1_genomic.fna
6157	Klebsiella pneumoniae	GCA_001530015.1_ASM153001v1_genomic.fna
6158	Bordetella pertussis	GCA_001605685.1_ASM160568v1_genomic.fna
6159	Corynebacterium pseudotuberculosis	GCA_001867145.1_ASM186714v1_genomic.fna
6160	Corynebacterium pseudotuberculosis	GCA_001867165.1_ASM186716v1_genomic.fna
6161	Bordetella pertussis	GCA_001628735.1_ASM162873v1_genomic.fna
6162	Klebsiella pneumoniae	GCA_001548315.1_ASM154831v1_genomic.fna
6163	Bacillus subtilis	GCA_002163815.1_ASM216381v1_genomic.fna
6164	Burkholderia pseudomallei	GCA_001887555.1_ASM188755v1_genomic.fna
6165	Chlamydia trachomatis	GCA_000590635.1_ASM59063v1_genomic.fna
6166	Legionella pneumophila	GCA_001766375.1_ASM176637v1_genomic.fna
6167	Acinetobacter baumannii	GCA_001887305.1_ASM188730v1_genomic.fna
6168	Pseudomonas aeruginosa	GCA_001594325.2_ASM159432v2_genomic.fna
6169	Streptococcus pyogenes	GCA_002236875.1_ASM223687v1_genomic.fna
6170	Neisseria meningitidis	GCA_001697765.1_ASM169776v1_genomic.fna
6171	Helicobacter pylori	GCA_000590775.1_ASM59077v1_genomic.fna
6172	Mycobacterium tuberculosis	GCA_002116775.1_ASM211677v1_genomic.fna
6173	Escherichia coli	GCA_000270105.1_ASM27010v1_genomic.fna
6174	Listeria monocytogenes	GCA_000950775.1_ASM95077v1_genomic.fna
6175	Staphylococcus aureus	GCA_000772025.1_ASM77202v1_genomic.fna
6176	Salmonella enterica	GCA_000462995.1_ASM46299v1_genomic.fna
6177	Campylobacter jejuni	GCA_001506485.1_ASM150648v1_genomic.fna
6178	Salmonella enterica	GCA_000473275.1_ASM47327v1_genomic.fna
6179	Campylobacter jejuni	GCA_001506505.1_ASM150650v1_genomic.fna
6180	Staphylococcus aureus	GCA_000815045.1_ASM81504v1_genomic.fna
6181	Listeria monocytogenes	GCA_001005925.1_ASM100592v1_genomic.fna
6182	Mycobacterium tuberculosis	GCA_002116795.1_ASM211679v1_genomic.fna
6183	Escherichia coli	GCA_000284495.1_ASM28449v1_genomic.fna
6184	Helicobacter pylori	GCA_000600045.1_ASM60004v1_genomic.fna
6185	Neisseria meningitidis	GCA_001697785.1_ASM169778v1_genomic.fna

6186	Streptococcus pyogenes	GCA_002238295.1_ASM223829v1_genomic.fna
6187	Fusobacterium nucleatum	GCA_001296125.1_ASM129612v1_genomic.fna
6188	Pseudomonas aeruginosa	GCA_001606045.1_ASM160604v1_genomic.fna
6189	Acinetobacter baumannii	GCA_001895985.1_ASM189598v1_genomic.fna
6190	Legionella pneumophila	GCA_001886795.1_ASM188679v1_genomic.fna
6191	Chlamydia trachomatis	GCA_000590675.1_ASM59067v1_genomic.fna
6192	Burkholderia pseudomallei	GCA_001887575.1_ASM188757v1_genomic.fna
6193	Bacillus subtilis	GCA_002173695.1_ASM217369v1_genomic.fna
6194	Klebsiella pneumoniae	GCA_001611055.1_ASM161105v1_genomic.fna
6195	Brucella suis	GCA_000755085.1_ASM75508v1_genomic.fna
6196	Bordetella pertussis	GCA_001686365.1_ASM168636v1_genomic.fna
6197	Corynebacterium pseudotuberculosis	GCA_001889145.1_ASM188914v1_genomic.fna
6198	Corynebacterium pseudotuberculosis	GCA_001922265.1_ASM192226v1_genomic.fna
6199	Bordetella pertussis	GCA_001686405.1_ASM168640v1_genomic.fna
6200	Brucella suis	GCA_000755105.1_ASM75510v1_genomic.fna
6201	Klebsiella pneumoniae	GCA_001611095.1_ASM161109v1_genomic.fna
6202	Bacillus subtilis	GCA_002173715.1_ASM217371v1_genomic.fna
6203	Burkholderia pseudomallei	GCA_001975065.1_ASM197506v1_genomic.fna
6204	Chlamydia trachomatis	GCA_000590755.1_ASM59075v1_genomic.fna
6205	Legionella pneumophila	GCA_001886835.1_ASM188683v1_genomic.fna
6206	Acinetobacter baumannii	GCA_001896005.1_ASM189600v1_genomic.fna
6207	Pseudomonas aeruginosa	GCA_001618925.1_ASM161892v1_genomic.fna
6208	Fusobacterium nucleatum	GCA_001296185.1_ASM129618v1_genomic.fna
6209	Streptococcus pyogenes	GCA_002238315.1_ASM223831v1_genomic.fna
6210	Neisseria meningitidis	GCA_001697805.1_ASM169780v1_genomic.fna
6211	Helicobacter pylori	GCA_000600085.1_ASM60008v1_genomic.fna
6212	Mycobacterium tuberculosis	GCA_002116815.1_ASM211681v1_genomic.fna
6213	Escherichia coli	GCA_000285655.3_EC958.v1_genomic.fna
6214	Listeria monocytogenes	GCA_001005985.1_ASM100598v1_genomic.fna
6215	Staphylococcus aureus	GCA_000815085.1_ASM81508v1_genomic.fna
6216	Campylobacter jejuni	GCA_001506525.1_ASM150652v1_genomic.fna
6217	Salmonella enterica	GCA_000483715.2_ASM48371v2_genomic.fna
6218	Campylobacter jejuni	GCA_001506545.1_ASM150654v1_genomic.fna
6219	Salmonella enterica	GCA_000484195.2_ASM48419v2_genomic.fna

6220	Staphylococcus aureus	GCA_000815125.1_ASM81512v1_genomic.fna
6221	Listeria monocytogenes	GCA_001027065.1_ASM102706v1_genomic.fna
6222	Mycobacterium tuberculosis	GCA_002116835.1_ASM211683v1_genomic.fna
6223	Escherichia coli	GCA_000299255.1_ASM29925v1_genomic.fna
6224	Helicobacter pylori	GCA_000600125.1_ASM60012v1_genomic.fna
6225	Neisseria meningitidis	GCA_001697825.1_ASM169782v1_genomic.fna
6226	Streptococcus pyogenes	GCA_002355175.1_ASM235517v1_genomic.fna
6227	Fusobacterium nucleatum	GCA_002211645.1_ASM221164v1_genomic.fna
6228	Pseudomonas aeruginosa	GCA_001632245.1_ASM163224v1_genomic.fna
6229	Acinetobacter baumannii	GCA_001902375.1_ASM190237v1_genomic.fna
6230	Legionella pneumophila	GCA_001941585.1_ASM194158v1_genomic.fna
6231	Chlamydia trachomatis	GCA_000971705.1_ASM97170v1_genomic.fna
6232	Burkholderia pseudomallei	GCA_001975085.1_ASM197508v1_genomic.fna
6233	Bacillus subtilis	GCA_002201955.1_ASM220195v1_genomic.fna
6234	Klebsiella pneumoniae	GCA_001644765.1_ASM164476v1_genomic.fna
6235	Chlamydia psittaci	GCA_000298375.2_ASM29837v2_genomic.fna
6236	Bordetella pertussis	GCA_001686425.1_ASM168642v1_genomic.fna
6237	Corynebacterium pseudotuberculosis	GCA_001922285.1_ASM192228v1_genomic.fna
6238	Corynebacterium pseudotuberculosis	GCA_001969145.2_ASM196914v2_genomic.fna
6239	Bordetella pertussis	GCA_001686445.1_ASM168644v1_genomic.fna
6240	Chlamydia psittaci	GCA_000338695.1_ASM33869v1_genomic.fna
6241	Klebsiella pneumoniae	GCA_001646625.1_ASM164662v1_genomic.fna
6242	Bacillus subtilis	GCA_002201995.1_ASM220199v1_genomic.fna
6243	Burkholderia pseudomallei	GCA_001975105.1_ASM197510v1_genomic.fna
6244	Chlamydia trachomatis	GCA_000971725.1_ASM97172v1_genomic.fna
6245	Legionella pneumophila	GCA_001969405.1_ASM196940v1_genomic.fna
6246	Acinetobacter baumannii	GCA_001908295.1_ASM190829v1_genomic.fna
6247	Pseudomonas aeruginosa	GCA_001679685.1_ASM167968v1_genomic.fna
6248	Streptococcus pyogenes	GCA_002355815.1_ASM235581v1_genomic.fna
6249	Neisseria meningitidis	GCA_001697845.1_ASM169784v1_genomic.fna
6250	Helicobacter pylori	GCA_000600145.1_ASM60014v1_genomic.fna
6251	Escherichia coli	GCA_000299475.1_ASM29947v1_genomic.fna
6252	Mycobacterium tuberculosis	GCA_002116855.1_ASM211685v1_genomic.fna
6253	Listeria monocytogenes	GCA_001027085.1_ASM102708v1_genomic.fna

6254	Staphylococcus aureus	GCA_000815165.1_ASM81516v1_genomic.fna
6255	Campylobacter jejuni	GCA_001506565.1_ASM150656v1_genomic.fna
6256	Salmonella enterica	GCA_000486045.2_ASM48604v2_genomic.fna
6257	Salmonella enterica	GCA_000486165.2_ASM48616v2_genomic.fna
6258	Campylobacter jejuni	GCA_001506585.1_ASM150658v1_genomic.fna
6259	Staphylococcus aureus	GCA_000815205.1_ASM81520v1_genomic.fna
6260	Listeria monocytogenes	GCA_001027125.1_ASM102712v1_genomic.fna
6261	Mycobacterium tuberculosis	GCA_002208235.1_ASM220823v1_genomic.fna
6262	Escherichia coli	GCA_000332755.1_ASM33275v1_genomic.fna
6263	Helicobacter pylori	GCA_000600165.1_ASM60016v1_genomic.fna
6264	Neisseria meningitidis	GCA_001697865.1_ASM169786v1_genomic.fna
6265	Streptococcus pyogenes	GCA_002557755.1_ASM255775v1_genomic.fna
6266	Pseudomonas aeruginosa	GCA_001721745.1_ASM172174v1_genomic.fna
6267	Acinetobacter baumannii	GCA_001922205.1_ASM192220v1_genomic.fna
6268	Legionella pneumophila	GCA_001989475.1_ASM198947v1_genomic.fna
6269	Chlamydia trachomatis	GCA_001183765.1_ASM118376v1_genomic.fna
6270	Burkholderia pseudomallei	GCA_001975125.1_ASM197512v1_genomic.fna
6271	Bacillus subtilis	GCA_002202035.1_ASM220203v1_genomic.fna
6272	Klebsiella pneumoniae	GCA_001648215.1_ASM164821v1_genomic.fna
6273	Bordetella pertussis	GCA_001686465.1_ASM168646v1_genomic.fna
6274	Corynebacterium pseudotuberculosis	GCA_001969165.2_ASM196916v2_genomic.fna
6275	Corynebacterium pseudotuberculosis	GCA_001975165.1_ASM197516v1_genomic.fna
6276	Bordetella pertussis	GCA_001686485.1_ASM168648v1_genomic.fna
6277	Klebsiella pneumoniae	GCA_001663195.1_ASM166319v1_genomic.fna
6278	Bacillus subtilis	GCA_002202055.1_ASM220205v1_genomic.fna
6279	Burkholderia pseudomallei	GCA_001975145.1_ASM197514v1_genomic.fna
6280	Chlamydia trachomatis	GCA_001183805.1_ASM118380v1_genomic.fna
6281	Legionella pneumophila	GCA_900092465.1_Lpm7613_v1_genomic.fna
6282	Acinetobacter baumannii	GCA_001922225.1_ASM192222v1_genomic.fna
6283	Pseudomonas aeruginosa	GCA_001721765.1_ASM172176v1_genomic.fna
6284	Neisseria meningitidis	GCA_001697885.1_ASM169788v1_genomic.fna
6285	Helicobacter pylori	GCA_000600185.1_ASM60018v1_genomic.fna
6286	Escherichia coli	GCA_000350185.1_ASM35018v1_genomic.fna
6287	Mycobacterium tuberculosis	GCA_002356015.1_ASM235601v1_genomic.fna

6288	Listeria monocytogenes	GCA_001027165.1_ASM102716v1_genomic.fna
6289	Staphylococcus aureus	GCA_000815245.1_ASM81524v1_genomic.fna
6290	Campylobacter jejuni	GCA_001506605.1_ASM150660v1_genomic.fna
6291	Salmonella enterica	GCA_000486345.2_ASM48634v2_genomic.fna
6292	Campylobacter jejuni	GCA_001506625.1_ASM150662v1_genomic.fna
6293	Salmonella enterica	GCA_000486365.2_ASM48636v2_genomic.fna
6294	Staphylococcus aureus	GCA_000828035.1_ASM82803v1_genomic.fna
6295	Listeria monocytogenes	GCA_001027205.1_ASM102720v1_genomic.fna
6296	Mycobacterium tuberculosis	GCA_002356255.1_ASM235625v1_genomic.fna
6297	Helicobacter pylori	GCA_000600205.1_ASM60020v1_genomic.fna
6298	Escherichia coli	GCA_000464955.2_ASM46495v2_genomic.fna
6299	Neisseria meningitidis	GCA_001697905.1_ASM169790v1_genomic.fna
6300	Pseudomonas aeruginosa	GCA_001721785.1_ASM172178v1_genomic.fna
6301	Acinetobacter baumannii	GCA_001922245.1_ASM192224v1_genomic.fna
6302	Legionella pneumophila	GCA_900119755.1_2531STDY5467379_genomic.fna
6303	Chlamydia trachomatis	GCA_001183825.1_ASM118382v1_genomic.fna
6304	Burkholderia pseudomallei	GCA_002110945.1_ASM211094v1_genomic.fna
6305	Bacillus subtilis	GCA_002216085.1_ASM221608v1_genomic.fna
6306	Klebsiella pneumoniae	GCA_001663295.1_ASM166329v1_genomic.fna
6307	Bordetella pertussis	GCA_001686505.1_ASM168650v1_genomic.fna
6308	Corynebacterium pseudotuberculosis	GCA_001989595.1_ASM198959v1_genomic.fna
6309	Corynebacterium pseudotuberculosis	GCA_002009385.1_ASM200938v1_genomic.fna
6310	Bordetella pertussis	GCA_001686525.1_ASM168652v1_genomic.fna
6311	Klebsiella pneumoniae	GCA_001663435.1_ASM166343v1_genomic.fna
6312	Bacillus subtilis	GCA_002269175.1_ASM226917v1_genomic.fna
6313	Burkholderia pseudomallei	GCA_002110965.1_ASM211096v1_genomic.fna
6314	Chlamydia trachomatis	GCA_001183845.1_ASM118384v1_genomic.fna
6315	Legionella pneumophila	GCA_900119765.1_2532STDY5467631_genomic.fna
6316	Acinetobacter baumannii	GCA_001941765.1_ASM194176v1_genomic.fna
6317	Pseudomonas aeruginosa	GCA_001721805.1_ASM172180v1_genomic.fna
6318	Neisseria meningitidis	GCA_001697925.1_ASM169792v1_genomic.fna
6319	Helicobacter pylori	GCA_000600225.1_ASM60022v1_genomic.fna
6320	Mycobacterium tuberculosis	GCA_002357935.1_ASM235793v1_genomic.fna
6321	Escherichia coli	GCA_000468515.1_ASM46851v1_genomic.fna

6322	Listeria monocytogenes	GCA_001027245.1_ASM102724v1_genomic.fna
6323	Staphylococcus aureus	GCA_000953255.1_Staphylococcus_aureus_Sa_ILRI_217_genomic.fna
6324	Salmonella enterica	GCA_000486405.2_ASM48640v2_genomic.fna
6325	Campylobacter jejuni	GCA_001506645.1_ASM150664v1_genomic.fna
6326	Campylobacter jejuni	GCA_001506665.1_ASM150666v1_genomic.fna
6327	Salmonella enterica	GCA_000486445.2_ASM48644v2_genomic.fna
6328	Staphylococcus aureus	GCA_000967325.1_ASM96732v1_genomic.fna
6329	Listeria monocytogenes	GCA_001047715.1_ASM104771v1_genomic.fna
6330	Mycobacterium tuberculosis	GCA_002357955.1_ASM235795v1_genomic.fna
6331	synthetic Escherichia	GCA_000474035.1_ASM47403v1_genomic.fna
6332	Helicobacter pylori	GCA_000685625.1_ASM68562v1_genomic.fna
6333	Neisseria meningitidis	GCA_001697945.1_ASM169794v1_genomic.fna
6334	Pseudomonas aeruginosa	GCA_001721825.1_ASM172182v1_genomic.fna
6335	Acinetobacter baumannii	GCA_002082625.1_ASM208262v1_genomic.fna
6336	Legionella pneumophila	GCA_900119775.1_2531STDY5467446_genomic.fna
6337	Chlamydia trachomatis	GCA_001655335.1_ASM165533v1_genomic.fna
6338	Burkholderia pseudomallei	GCA_002111005.1_ASM211100v1_genomic.fna
6339	Bacillus subtilis	GCA_002269195.1_ASM226919v1_genomic.fna
6340	Klebsiella pneumoniae	GCA_001663455.1_ASM166345v1_genomic.fna
6341	Bordetella pertussis	GCA_001686545.1_ASM168654v1_genomic.fna
6342	Corynebacterium pseudotuberculosis	GCA_002009415.1_ASM200941v1_genomic.fna
6343	Corynebacterium pseudotuberculosis	GCA_002072715.1_ASM207271v1_genomic.fna
6344	Flavobacterium psychrophilum	GCA_001431805.1_ASM143180v1_genomic.fna
6345	Bordetella pertussis	GCA_001686565.1_ASM168656v1_genomic.fna
6346	Klebsiella pneumoniae	GCA_001675125.1_ASM167512v1_genomic.fna
6347	Bacillus subtilis	GCA_002290305.1_ASM229030v1_genomic.fna
6348	Burkholderia pseudomallei	GCA_002111025.1_ASM211102v1_genomic.fna
6349	Chlamydia trachomatis	GCA_001655375.1_ASM165537v1_genomic.fna
6350	Legionella pneumophila	GCA_900119785.1_2531STDY5467415_genomic.fna
6351	Acinetobacter baumannii	GCA_002082645.1_ASM208264v1_genomic.fna
6352	Pseudomonas aeruginosa	GCA_001721845.1_ASM172184v1_genomic.fna
6353	Neisseria meningitidis	GCA_001697965.1_ASM169796v1_genomic.fna
6354	Helicobacter pylori	GCA_000685665.1_ASM68566v1_genomic.fna
6355	Mycobacterium tuberculosis	GCA_002357975.1_ASM235797v1_genomic.fna

6356	Escherichia coli	GCA_000493755.1_ASM49375v1_genomic.fna
6357	Listeria monocytogenes	GCA_001188655.1_ASM118865v1_genomic.fna
6358	Staphylococcus aureus	GCA_000967345.1_ASM96734v1_genomic.fna
6359	Campylobacter jejuni	GCA_001506685.1_ASM150668v1_genomic.fna
6360	Salmonella enterica	GCA_000486585.2_ASM48658v2_genomic.fna
6361	Salmonella enterica	GCA_000486725.2_ASM48672v2_genomic.fna
6362	Campylobacter jejuni	GCA_001506705.1_ASM150670v1_genomic.fna
6363	Staphylococcus aureus	GCA_000967365.1_ASM96736v1_genomic.fna
6364	Listeria monocytogenes	GCA_001454845.1_ASM145484v1_genomic.fna
6365	Escherichia coli	GCA_000499485.1_MYMC4100_genomic.fna
6366	Mycobacterium tuberculosis	GCA_002446875.1_ASM244687v1_genomic.fna
6367	Helicobacter pylori	GCA_000685705.1_ASM68570v1_genomic.fna
6368	Neisseria meningitidis	GCA_001697985.1_ASM169798v1_genomic.fna
6369	Pseudomonas aeruginosa	GCA_001722005.2_ASM172200v2_genomic.fna
6370	Acinetobacter baumannii	GCA_002082685.1_ASM208268v1_genomic.fna
6371	Legionella pneumophila	GCA_900186855.1_48290_H02_genomic.fna
6372	Chlamydia trachomatis	GCA_001655415.1_ASM165541v1_genomic.fna
6373	Burkholderia pseudomallei	GCA_002111045.1_ASM211104v1_genomic.fna
6374	Bacillus subtilis	GCA_002556525.1_ASM255652v1_genomic.fna
6375	Klebsiella pneumoniae	GCA_001676825.1_ASM167682v1_genomic.fna
6376	Bordetella pertussis	GCA_001686585.1_ASM168658v1_genomic.fna
6377	Flavobacterium psychrophilum	GCA_001708385.1_ASM170838v1_genomic.fna
6378	Corynebacterium pseudotuberculosis	GCA_002155265.1_ASM215526v1_genomic.fna
6379	Bordetella pertussis	GCA_001686605.1_ASM168660v1_genomic.fna
6380	Klebsiella pneumoniae	GCA_001704235.1_ASM170423v1_genomic.fna
6381	Burkholderia pseudomallei	GCA_002111085.1_ASM211108v1_genomic.fna
6382	Chlamydia trachomatis	GCA_001655455.1_ASM165545v1_genomic.fna
6383	Legionella pneumophila	GCA_900186925.1_48450_B02_genomic.fna
6384	Acinetobacter baumannii	GCA_002082705.1_ASM208270v1_genomic.fna
6385	Pseudomonas aeruginosa	GCA_001722025.1_ASM172202v1_genomic.fna
6386	Neisseria meningitidis	GCA_001698005.1_ASM169800v1_genomic.fna
6387	Helicobacter pylori	GCA_000685745.1_ASM68574v1_genomic.fna
6388	Mycobacterium tuberculosis	GCA_002446895.1_ASM244689v1_genomic.fna
6389	Escherichia coli	GCA_000520035.1_ASM52003v1_genomic.fna

6390	Listeria monocytogenes	GCA_001454865.1_ASM145486v1_genomic.fna
6391	Staphylococcus aureus	GCA_000967385.1_ASM96738v1_genomic.fna
6392	Campylobacter jejuni	GCA_001506725.1_ASM150672v1_genomic.fna
6393	Salmonella enterica	GCA_000486765.2_ASM48676v2_genomic.fna
6394	Campylobacter jejuni	GCA_001506745.1_ASM150674v1_genomic.fna
6395	Salmonella enterica	GCA_000486855.2_ASM48685v2_genomic.fna
6396	Staphylococcus aureus	GCA_000967405.1_ASM96740v1_genomic.fna
6397	Listeria monocytogenes	GCA_001454885.1_ASM145488v1_genomic.fna
6398	Escherichia coli	GCA_000520055.1_ASM52005v1_genomic.fna
6399	Mycobacterium tuberculosis	GCA_002446915.1_ASM244691v1_genomic.fna
6400	Helicobacter pylori	GCA_000817025.1_ASM81702v1_genomic.fna
6401	Neisseria meningitidis	GCA_001698025.1_ASM169802v1_genomic.fna
6402	Pseudomonas aeruginosa	GCA_001722045.1_ASM172204v1_genomic.fna
6403	Acinetobacter baumannii	GCA_002082725.1_ASM208272v1_genomic.fna
6404	Chlamydia trachomatis	GCA_001655495.1_ASM165549v1_genomic.fna
6405	Burkholderia pseudomallei	GCA_002111105.1_ASM211110v1_genomic.fna
6406	Klebsiella pneumoniae	GCA_001705385.1_ASM170538v1_genomic.fna
6407	Bordetella bronchiseptica	GCA_001559055.1_ASM155905v1_genomic.fna
6408	Bordetella pertussis	GCA_001686625.1_ASM168662v1_genomic.fna
6409	Bordetella pertussis	GCA_001687385.1_ASM168738v1_genomic.fna
6410	Klebsiella pneumoniae	GCA_001708225.1_ASM170822v1_genomic.fna
6411	Burkholderia pseudomallei	GCA_002111125.1_ASM211112v1_genomic.fna
6412	Chlamydia trachomatis	GCA_001655535.1_ASM165553v1_genomic.fna
6413	Acinetobacter baumannii	GCA_002082745.1_ASM208274v1_genomic.fna
6414	Pseudomonas aeruginosa	GCA_001729505.1_ASM172950v1_genomic.fna
6415	Neisseria meningitidis	GCA_001698045.1_ASM169804v1_genomic.fna
6416	Helicobacter pylori	GCA_000826985.1_ASM82698v1_genomic.fna
6417	Mycobacterium tuberculosis	GCA_002446935.1_ASM244693v1_genomic.fna
6418	Escherichia coli	GCA_000597845.1_ASM59784v1_genomic.fna
6419	Listeria monocytogenes	GCA_001454905.1_ASM145490v1_genomic.fna
6420	Staphylococcus aureus	GCA_000969225.1_ASM96922v1_genomic.fna
6421	Salmonella enterica	GCA_000486915.2_ASM48691v2_genomic.fna
6422	Campylobacter jejuni	GCA_001506765.1_ASM150676v1_genomic.fna
6423	Campylobacter jejuni	GCA_001506785.1_ASM150678v1_genomic.fna

6424	Salmonella enterica	GCA_000487035.2_ASM48703v2_genomic.fna
6425	Staphylococcus aureus	GCA_001021875.1_ASM102187v1_genomic.fna
6426	Listeria monocytogenes	GCA_001454925.1_ASM145492v1_genomic.fna
6427	Escherichia coli	GCA_000599625.1_ASM59962v1_genomic.fna
6428	Mycobacterium tuberculosis	GCA_002446955.1_ASM244695v1_genomic.fna
6429	Helicobacter pylori	GCA_000827025.1_ASM82702v1_genomic.fna
6430	Neisseria meningitidis	GCA_001698065.1_ASM169806v1_genomic.fna
6431	Pseudomonas aeruginosa	GCA_001750705.1_ASM175070v1_genomic.fna
6432	Acinetobacter baumannii	GCA_002082785.1_ASM208278v1_genomic.fna
6433	Chlamydia trachomatis	GCA_001655575.1_ASM165557v1_genomic.fna
6434	Burkholderia pseudomallei	GCA_002111145.1_ASM211114v1_genomic.fna
6435	Klebsiella pneumoniae	GCA_001708245.1_ASM170824v1_genomic.fna
6436	Bordetella pertussis	GCA_001687405.1_ASM168740v1_genomic.fna
6437	Bordetella pertussis	GCA_001831395.1_ASM183139v1_genomic.fna
6438	Klebsiella pneumoniae	GCA_001709275.1_ASM170927v1_genomic.fna
6439	Burkholderia pseudomallei	GCA_002111165.1_ASM211116v1_genomic.fna
6440	Chlamydia trachomatis	GCA_001885175.1_ASM188517v1_genomic.fna
6441	Acinetobacter baumannii	GCA_002082805.1_ASM208280v1_genomic.fna
6442	Pseudomonas aeruginosa	GCA_001792835.1_ASM179283v1_genomic.fna
6443	Neisseria meningitidis	GCA_001698085.1_ASM169808v1_genomic.fna
6444	Helicobacter pylori	GCA_000828955.1_ASM82895v1_genomic.fna
6445	Mycobacterium tuberculosis	GCA_002446975.1_ASM244697v1_genomic.fna
6446	Escherichia coli	GCA_000599645.1_ASM59964v1_genomic.fna
6447	Listeria monocytogenes	GCA_001483405.1_ASM148340v1_genomic.fna
6448	Staphylococcus aureus	GCA_001021895.1_ASM102189v1_genomic.fna
6449	Salmonella enterica	GCA_000487155.2_ASM48715v2_genomic.fna
6450	Campylobacter jejuni	GCA_001506805.1_ASM150680v1_genomic.fna
6451	Campylobacter jejuni	GCA_001506825.1_ASM150682v1_genomic.fna
6452	Salmonella enterica	GCA_000487255.2_ASM48725v2_genomic.fna
6453	Staphylococcus aureus	GCA_001027045.1_ASM102704v1_genomic.fna
6454	Listeria monocytogenes	GCA_001483425.1_ASM148342v1_genomic.fna
6455	Escherichia coli	GCA_000599665.1_ASM59966v1_genomic.fna
6456	Mycobacterium tuberculosis	GCA_002446995.1_ASM244699v1_genomic.fna
6457	Helicobacter pylori	GCA_000829095.1_ASM82909v1_genomic.fna

6458	Neisseria meningitidis	GCA_001698105.1_ASM169810v1_genomic.fna
6459	Pseudomonas aeruginosa	GCA_001792855.1_ASM179285v1_genomic.fna
6460	Acinetobacter baumannii	GCA_002082825.1_ASM208282v1_genomic.fna
6461	Chlamydia trachomatis	GCA_002088315.1_ASM208831v1_genomic.fna
6462	Burkholderia pseudomallei	GCA_002111205.1_ASM211120v1_genomic.fna
6463	Klebsiella pneumoniae	GCA_001709295.1_ASM170929v1_genomic.fna
6464	Bordetella pertussis	GCA_001831415.1_ASM183141v1_genomic.fna
6465	Bordetella pertussis	GCA_001831435.1_ASM183143v1_genomic.fna
6466	Klebsiella pneumoniae	GCA_001721725.1_ASM172172v1_genomic.fna
6467	Burkholderia pseudomallei	GCA_002111245.1_ASM211124v1_genomic.fna
6468	Chlamydia trachomatis	GCA_002088335.1_ASM208833v1_genomic.fna
6469	Acinetobacter baumannii	GCA_002082845.1_ASM208284v1_genomic.fna
6470	Pseudomonas aeruginosa	GCA_001792875.1_ASM179287v1_genomic.fna
6471	Neisseria meningitidis	GCA_001750825.1_ASM175082v1_genomic.fna
6472	Helicobacter pylori	GCA_000829115.1_ASM82911v1_genomic.fna
6473	Mycobacterium tuberculosis	GCA_002447015.1_ASM244701v1_genomic.fna
6474	Escherichia coli	GCA_000599685.1_ASM59968v1_genomic.fna
6475	Listeria monocytogenes	GCA_001483445.1_ASM148344v1_genomic.fna
6476	Staphylococcus aureus	GCA_001027105.1_ASM102710v1_genomic.fna
6477	Campylobacter jejuni	GCA_001506845.1_ASM150684v1_genomic.fna
6478	Salmonella enterica	GCA_000487295.2_ASM48729v2_genomic.fna
6479	Campylobacter jejuni	GCA_001506865.1_ASM150686v1_genomic.fna
6480	Salmonella enterica	GCA_000487515.2_ASM48751v2_genomic.fna
6481	Staphylococcus aureus	GCA_001045795.2_ASM104579v2_genomic.fna
6482	Listeria monocytogenes	GCA_001513695.1_ASM151369v1_genomic.fna
6483	Escherichia coli	GCA_000599705.1_ASM59970v1_genomic.fna
6484	Mycobacterium tuberculosis	GCA_002447035.1_ASM244703v1_genomic.fna
6485	Helicobacter pylori	GCA_000829135.1_ASM82913v1_genomic.fna
6486	Neisseria meningitidis	GCA_001974965.1_ASM197496v1_genomic.fna
6487	Pseudomonas aeruginosa	GCA_001870265.1_ASM187026v1_genomic.fna
6488	Acinetobacter baumannii	GCA_002082865.1_ASM208286v1_genomic.fna
6489	Chlamydia trachomatis	GCA_002088355.1_ASM208835v1_genomic.fna
6490	Burkholderia pseudomallei	GCA_002111265.1_ASM211126v1_genomic.fna
6491	Klebsiella pneumoniae	GCA_001741545.1_ASM174154v1_genomic.fna

6492	Bordetella pertussis	GCA_001831455.1_ASM183145v1_genomic.fna
6493	Bordetella pertussis	GCA_001984925.1_ASM198492v1_genomic.fna
6494	Klebsiella pneumoniae	GCA_001741665.1_ASM174166v1_genomic.fna
6495	Burkholderia pseudomallei	GCA_002111305.1_ASM211130v1_genomic.fna
6496	Acinetobacter baumannii	GCA_002082885.1_ASM208288v1_genomic.fna
6497	Pseudomonas aeruginosa	GCA_001874465.1_ASM187446v1_genomic.fna
6498	Neisseria meningitidis	GCA_002073235.1_ASM207323v1_genomic.fna
6499	Helicobacter pylori	GCA_000982695.1_ASM98269v1_genomic.fna
6500	Mycobacterium tuberculosis	GCA_002447055.1_ASM244705v1_genomic.fna
6501	Escherichia coli	GCA_000662395.1_ASM66239v1_genomic.fna
6502	Listeria monocytogenes	GCA_001548585.1_ASM154858v1_genomic.fna
6503	Staphylococcus aureus	GCA_001045995.2_ASM104599v2_genomic.fna
6504	Campylobacter jejuni	GCA_001506885.1_ASM150688v1_genomic.fna
6505	Salmonella enterica	GCA_000487575.2_ASM48757v2_genomic.fna
6506	Salmonella enterica	GCA_000487615.2_ASM48761v2_genomic.fna
6507	Campylobacter jejuni	GCA_001506905.1_ASM150690v1_genomic.fna
6508	Staphylococcus aureus	GCA_001046095.2_ASM104609v2_genomic.fna
6509	Listeria monocytogenes	GCA_001548635.1_ASM154863v1_genomic.fna
6510	Escherichia coli	GCA_000671295.1_ASM67129v1_genomic.fna
6511	Mycobacterium tuberculosis	GCA_002447075.1_ASM244707v1_genomic.fna
6512	Helicobacter pylori	GCA_001433495.1_ASM143349v1_genomic.fna
6513	Neisseria meningitidis	GCA_002073275.1_ASM207327v1_genomic.fna
6514	Pseudomonas aeruginosa	GCA_001879525.1_ASM187952v1_genomic.fna
6515	Acinetobacter baumannii	GCA_002116925.1_ASM211692v1_genomic.fna
6516	Burkholderia pseudomallei	GCA_002111325.1_ASM211132v1_genomic.fna
6517	Ralstonia solanacearum	GCA_001267515.1_ASM126751v1_genomic.fna
6518	Klebsiella pneumoniae	GCA_001746535.1_ASM174653v1_genomic.fna
6519	Bordetella pertussis	GCA_001984945.1_ASM198494v1_genomic.fna
6520	Bordetella pertussis	GCA_001984965.1_ASM198496v1_genomic.fna
6521	Klebsiella pneumoniae	GCA_001750805.1_ASM175080v1_genomic.fna
6522	Burkholderia pseudomallei	GCA_002111345.1_ASM211134v1_genomic.fna
6523	Acinetobacter baumannii	GCA_002210065.1_ASM221006v1_genomic.fna
6524	Pseudomonas aeruginosa	GCA_001900195.1_ASM190019v1_genomic.fna
6525	Neisseria meningitidis	GCA_002073555.1_ASM207355v1_genomic.fna

6526	Helicobacter pylori	GCA_001433515.1_ASM143351v1_genomic.fna
6527	Mycobacterium tuberculosis	GCA_002447095.1_ASM244709v1_genomic.fna
6528	Escherichia coli	GCA_000714595.1_ASM71459v1_genomic.fna
6529	Listeria monocytogenes	GCA_001551855.1_ASM155185v1_genomic.fna
6530	Staphylococcus aureus	GCA_001183705.1_ASM118370v1_genomic.fna
6531	Campylobacter jejuni	GCA_001506925.1_ASM150692v1_genomic.fna
6532	Salmonella enterica	GCA_000487775.2_ASM48777v2_genomic.fna
6533	Campylobacter jejuni	GCA_001506945.1_ASM150694v1_genomic.fna
6534	Salmonella enterica	GCA_000487915.2_ASM48791v2_genomic.fna
6535	Staphylococcus aureus	GCA_001183725.1_ASM118372v1_genomic.fna
6536	Listeria monocytogenes	GCA_001596775.2_ASM159677v2_genomic.fna
6537	Escherichia coli	GCA_000725265.1_ASM72526v1_genomic.fna
6538	Mycobacterium tuberculosis	GCA_002447115.1_ASM244711v1_genomic.fna
6539	Helicobacter pylori	GCA_001549715.1_ASM154971v1_genomic.fna
6540	Neisseria meningitidis	GCA_002073575.1_ASM207357v1_genomic.fna
6541	Pseudomonas aeruginosa	GCA_001900225.1_ASM190022v1_genomic.fna
6542	Acinetobacter baumannii	GCA_002504145.1_ASM250414v1_genomic.fna
6543	Burkholderia pseudomallei	GCA_002111365.1_ASM211136v1_genomic.fna
6544	Klebsiella pneumoniae	GCA_001855315.1_ASM185531v1_genomic.fna
6545	Bordetella pertussis	GCA_001984985.1_ASM198498v1_genomic.fna
6546	Bordetella pertussis	GCA_001985005.1_ASM198500v1_genomic.fna
6547	Klebsiella pneumoniae	GCA_001870165.1_ASM187016v1_genomic.fna
6548	Burkholderia pseudomallei	GCA_002111385.1_ASM211138v1_genomic.fna
6549	Acinetobacter baumannii	GCA_900088705.1_26872B01_genomic.fna
6550	Pseudomonas aeruginosa	GCA_001900265.1_ASM190026v1_genomic.fna
6551	Neisseria meningitidis	GCA_002073595.1_ASM207359v1_genomic.fna
6552	Helicobacter pylori	GCA_001653375.1_ASM165337v1_genomic.fna
6553	Mycobacterium tuberculosis	GCA_002447135.1_ASM244713v1_genomic.fna
6554	Escherichia coli	GCA_000725305.1_ASM72530v1_genomic.fna
6555	Listeria monocytogenes	GCA_001644625.1_ASM164462v1_genomic.fna
6556	Staphylococcus aureus	GCA_001278745.1_ASM127874v1_genomic.fna
6557	Campylobacter jejuni	GCA_001506965.1_ASM150696v1_genomic.fna
6558	Salmonella enterica	GCA_000487935.2_ASM48793v2_genomic.fna
6559	Salmonella enterica	GCA_000493535.2_DT2_genomic.fna

6560	Campylobacter jejuni	GCA_001506985.1_ASM150698v1_genomic.fna
6561	Staphylococcus aureus	GCA_001281145.1_ASM128114v1_genomic.fna
6562	Listeria monocytogenes	GCA_001908335.1_ASM190833v1_genomic.fna
6563	Escherichia coli	GCA_000730345.1_ASM73034v1_genomic.fna
6564	Mycobacterium tuberculosis	GCA_002447155.1_ASM244715v1_genomic.fna
6565	Helicobacter pylori	GCA_001653395.1_ASM165339v1_genomic.fna
6566	Neisseria meningitidis	GCA_002073675.1_ASM207367v1_genomic.fna
6567	Pseudomonas aeruginosa	GCA_002075065.1_ASM207506v1_genomic.fna
6568	Klebsiella pneumoniae	GCA_001885255.2_ASM188525v2_genomic.fna
6569	Bordetella pertussis	GCA_001985025.1_ASM198502v1_genomic.fna
6570	Bordetella pertussis	GCA_001985045.1_ASM198504v1_genomic.fna
6571	Klebsiella pneumoniae	GCA_001902195.1_ASM190219v1_genomic.fna
6572	Pseudomonas aeruginosa	GCA_002085605.1_ASM208560v1_genomic.fna
6573	Neisseria meningitidis	GCA_002075815.1_ASM207581v1_genomic.fna
6574	Helicobacter pylori	GCA_001653415.1_ASM165341v1_genomic.fna
6575	Mycobacterium tuberculosis	GCA_002447175.1_ASM244717v1_genomic.fna
6576	Escherichia coli	GCA_000732965.1_ASM73296v1_genomic.fna
6577	Listeria monocytogenes	GCA_001913195.1_ASM191319v1_genomic.fna
6578	Staphylococcus aureus	GCA_001296985.1_ASM129698v1_genomic.fna
6579	Campylobacter jejuni	GCA_001507005.1_ASM150700v1_genomic.fna
6580	Salmonella enterica	GCA_000493675.1_DT104_genomic.fna
6581	Campylobacter jejuni	GCA_001507025.1_ASM150702v1_genomic.fna
6582	Salmonella enterica	GCA_000494385.2_ASM49438v2_genomic.fna
6583	Staphylococcus aureus	GCA_001298325.2_ASM129832v2_genomic.fna
6584	Listeria monocytogenes	GCA_001952735.1_ASM195273v1_genomic.fna
6585	Escherichia coli	GCA_000743255.1_ASM74325v1_genomic.fna
6586	Mycobacterium tuberculosis	GCA_002447195.1_ASM244719v1_genomic.fna
6587	Helicobacter pylori	GCA_001653435.1_ASM165343v1_genomic.fna
6588	Neisseria meningitidis	GCA_002556585.1_ASM255658v1_genomic.fna
6589	Pseudomonas putida	GCA_000281215.1_ASM28121v1_genomic.fna
6590	Pseudomonas aeruginosa	GCA_002085755.1_ASM208575v1_genomic.fna
6591	Klebsiella pneumoniae	GCA_001902215.1_ASM190221v1_genomic.fna
6592	Bordetella pertussis	GCA_001985065.1_ASM198506v1_genomic.fna
6593	Bordetella pertussis	GCA_001985085.1_ASM198508v1_genomic.fna

6594	Klebsiella pneumoniae	GCA_001902235.1_ASM190223v1_genomic.fna
6595	Pseudomonas aeruginosa	GCA_002104595.1_ASM210459v1_genomic.fna
6596	Helicobacter pylori	GCA_001653455.1_ASM165345v1_genomic.fna
6597	Mycobacterium tuberculosis	GCA_002447215.1_ASM244721v1_genomic.fna
6598	Escherichia coli	GCA_000750555.1_ASM75055v1_genomic.fna
6599	Listeria monocytogenes	GCA_001952755.1_ASM195275v1_genomic.fna
6600	Staphylococcus aureus	GCA_001307235.1_ASM130723v1_genomic.fna
6601	Campylobacter jejuni	GCA_001507045.1_ASM150704v1_genomic.fna
6602	Salmonella enterica	GCA_000503845.1_ASM50384v1_genomic.fna
6603	Campylobacter jejuni	GCA_001507065.1_ASM150706v1_genomic.fna
6604	Salmonella enterica	GCA_000505365.2_ASM50536v2_genomic.fna
6605	Staphylococcus aureus	GCA_001444345.1_ASM144434v1_genomic.fna
6606	Listeria monocytogenes	GCA_001952775.1_ASM195277v1_genomic.fna
6607	Escherichia coli	GCA_000784925.1_ASM78492v1_genomic.fna
6608	Mycobacterium tuberculosis	GCA_002447235.1_ASM244723v1_genomic.fna
6609	Helicobacter pylori	GCA_001653475.1_ASM165347v1_genomic.fna
6610	Pseudomonas aeruginosa	GCA_002104615.1_ASM210461v1_genomic.fna
6611	Klebsiella pneumoniae	GCA_001902255.1_ASM190225v1_genomic.fna
6612	Bordetella pertussis	GCA_001985105.1_ASM198510v1_genomic.fna
6613	Bordetella pertussis	GCA_001985125.1_ASM198512v1_genomic.fna
6614	Klebsiella pneumoniae	GCA_001902335.1_ASM190233v1_genomic.fna
6615	Pseudomonas aeruginosa	GCA_002192475.1_ASM219247v1_genomic.fna
6616	Helicobacter pylori	GCA_001991095.1_ASM199109v1_genomic.fna
6617	Mycobacterium tuberculosis	GCA_002447255.1_ASM244725v1_genomic.fna
6618	Escherichia coli	GCA_000800215.1_ASM80021v1_genomic.fna
6619	Listeria monocytogenes	GCA_001952795.1_ASM195279v1_genomic.fna
6620	Staphylococcus aureus	GCA_001456215.1_ASM145621v1_genomic.fna
6621	Campylobacter jejuni	GCA_001507085.1_ASM150708v1_genomic.fna
6622	Salmonella enterica	GCA_000505705.1_ASM50570v1_genomic.fna
6623	Salmonella enterica	GCA_000612325.1_ASM61232v1_genomic.fna
6624	Campylobacter jejuni	GCA_001507105.1_ASM150710v1_genomic.fna
6625	Staphylococcus aureus	GCA_001457495.1_NCTC13435_genomic.fna
6626	Listeria monocytogenes	GCA_001998915.1_ASM199891v1_genomic.fna
6627	Escherichia coli	GCA_000800765.1_ASM80076v1_genomic.fna

6628	Mycobacterium tuberculosis	GCA_002447275.1_ASM244727v1_genomic.fna
6629	Helicobacter pylori	GCA_002005525.1_ASM200552v1_genomic.fna
6630	Pseudomonas aeruginosa	GCA_002192495.1_ASM219249v1_genomic.fna
6631	Klebsiella pneumoniae	GCA_001902355.1_ASM190235v1_genomic.fna
6632	Bordetella pertussis	GCA_001985145.1_ASM198514v1_genomic.fna
6633	Bordetella pertussis	GCA_001985165.1_ASM198516v1_genomic.fna
6634	Klebsiella pneumoniae	GCA_001902395.1_ASM190239v1_genomic.fna
6635	Pseudomonas aeruginosa	GCA_002205335.1_ASM220533v1_genomic.fna
6636	Helicobacter pylori	GCA_002222575.1_ASM222257v1_genomic.fna
6637	Mycobacterium tuberculosis	GCA_002447295.1_ASM244729v1_genomic.fna
6638	Escherichia coli	GCA_000800845.2_ASM80084v2_genomic.fna
6639	Listeria monocytogenes	GCA_001998945.1_ASM199894v1_genomic.fna
6640	Staphylococcus aureus	GCA_001465635.1_ASM146563v1_genomic.fna
6641	Campylobacter jejuni	GCA_001507125.1_ASM150712v1_genomic.fna
6642	Salmonella enterica	GCA_000623055.2_ASM62305v2_genomic.fna
6643	Campylobacter jejuni	GCA_001507145.1_ASM150714v1_genomic.fna
6644	Salmonella enterica	GCA_000623095.2_ASM62309v2_genomic.fna
6645	Staphylococcus aureus	GCA_001465675.1_ASM146567v1_genomic.fna
6646	Listeria monocytogenes	GCA_001998985.1_ASM199898v1_genomic.fna
6647	Mycobacterium tuberculosis	GCA_002447315.1_ASM244731v1_genomic.fna
6648	Escherichia coli	GCA_000801165.1_ASM80116v1_genomic.fna
6649	Helicobacter pylori	GCA_002356595.1_ASM235659v1_genomic.fna
6650	Pseudomonas aeruginosa	GCA_002205355.1_ASM220535v1_genomic.fna
6651	Klebsiella pneumoniae	GCA_001902415.1_ASM190241v1_genomic.fna
6652	Bordetella pertussis	GCA_001985205.1_ASM198520v1_genomic.fna
6653	Bordetella pertussis	GCA_001985225.1_ASM198522v1_genomic.fna
6654	Klebsiella pneumoniae	GCA_001902435.1_ASM190243v1_genomic.fna
6655	Pseudomonas aeruginosa	GCA_002205375.1_ASM220537v1_genomic.fna
6656	Helicobacter pylori	GCA_900119955.1_HE171_09_genomic.fna
6657	Escherichia coli	GCA_000801185.2_ASM80118v2_genomic.fna
6658	Mycobacterium tuberculosis	GCA_002447335.1_ASM244733v1_genomic.fna
6659	Listeria monocytogenes	GCA_001999005.1_ASM199900v1_genomic.fna
6660	Staphylococcus aureus	GCA_001465755.1_ASM146575v1_genomic.fna
6661	Salmonella enterica	GCA_000623115.2_ASM62311v2_genomic.fna

6662	Campylobacter jejuni	GCA_001507165.1_ASM150716v1_genomic.fna
6663	Campylobacter jejuni	GCA_001507185.1_ASM150718v1_genomic.fna
6664	Salmonella enterica	GCA_000623135.2_ASM62313v2_genomic.fna
6665	Staphylococcus aureus	GCA_001515665.1_ASM151566v1_genomic.fna
6666	Listeria monocytogenes	GCA_001999025.1_ASM199902v1_genomic.fna
6667	Mycobacterium tuberculosis	GCA_002447355.1_ASM244735v1_genomic.fna
6668	Escherichia coli	GCA_000801205.1_ASM80120v1_genomic.fna
6669	Helicobacter pylori	GCA_900119995.1_HE143_09_genomic.fna
6670	Pseudomonas aeruginosa	GCA_002208645.1_ASM220864v1_genomic.fna
6671	Klebsiella pneumoniae	GCA_001902475.1_ASM190247v1_genomic.fna
6672	Bordetella pertussis	GCA_001985245.1_ASM198524v1_genomic.fna
6673	Bacillus velezensis	GCA_001634675.1_ASM163467v1_genomic.fna
6674	Bordetella pertussis	GCA_001985265.1_ASM198526v1_genomic.fna
6675	Klebsiella pneumoniae	GCA_001902515.1_ASM190251v1_genomic.fna
6676	Pseudomonas aeruginosa	GCA_002215345.1_ASM221534v1_genomic.fna
6677	Helicobacter pylori	GCA_900120055.1_HE178_09_genomic.fna
6678	Escherichia coli	GCA_000803705.1_ASM80370v1_genomic.fna
6679	Mycobacterium tuberculosis	GCA_002447375.1_ASM244737v1_genomic.fna
6680	Listeria monocytogenes	GCA_001999045.1_ASM199904v1_genomic.fna
6681	Staphylococcus aureus	GCA_001515685.1_ASM151568v1_genomic.fna
6682	Campylobacter jejuni	GCA_001507205.1_ASM150720v1_genomic.fna
6683	Salmonella enterica	GCA_000623195.2_ASM62319v2_genomic.fna
6684	Salmonella enterica	GCA_000623275.1_ASM62327v1_genomic.fna
6685	Campylobacter jejuni	GCA_001507225.1_ASM150722v1_genomic.fna
6686	Staphylococcus aureus	GCA_001515705.1_ASM151570v1_genomic.fna
6687	Listeria monocytogenes	GCA_001999065.1_ASM199906v1_genomic.fna
6688	Mycobacterium tuberculosis	GCA_002447395.1_ASM244739v1_genomic.fna
6689	Escherichia coli	GCA_000813165.1_ASM81316v1_genomic.fna
6690	Helicobacter pylori	GCA_900120135.1_HE132_09_genomic.fna
6691	Pseudomonas aeruginosa	GCA_002223805.1_ASM222380v1_genomic.fna
6692	Klebsiella pneumoniae	GCA_001902535.1_ASM190253v1_genomic.fna
6693	Bordetella pertussis	GCA_001985285.1_ASM198528v1_genomic.fna
6694	Bacillus velezensis	GCA_002201935.1_ASM220193v1_genomic.fna
6695	Bacillus velezensis	GCA_002201975.1_ASM220197v1_genomic.fna

6696	Bordetella pertussis	GCA_001985305.1_ASM198530v1_genomic.fna
6697	Klebsiella pneumoniae	GCA_001908515.1_ASM190851v1_genomic.fna
6698	Pseudomonas aeruginosa	GCA_002237405.1_ASM223740v1_genomic.fna
6699	Helicobacter pylori	GCA_900120185.1_HE134_09_genomic.fna
6700	Escherichia coli	GCA_000814145.2_ASM81414v2_genomic.fna
6701	Mycobacterium tuberculosis	GCA_002447415.1_ASM244741v1_genomic.fna
6702	Listeria monocytogenes	GCA_001999085.1_ASM199908v1_genomic.fna
6703	Staphylococcus aureus	GCA_001515745.1_ASM151574v1_genomic.fna
6704	Campylobacter jejuni	GCA_001507245.1_ASM150724v1_genomic.fna
6705	Salmonella enterica	GCA_000623295.1_ASM62329v1_genomic.fna
6706	Salmonella enterica	GCA_000623315.1_ASM62331v1_genomic.fna
6707	Campylobacter jejuni	GCA_001507265.1_ASM150726v1_genomic.fna
6708	Staphylococcus aureus	GCA_001515765.1_ASM151576v1_genomic.fna
6709	Listeria monocytogenes	GCA_001999105.1_ASM199910v1_genomic.fna
6710	Mycobacterium tuberculosis	GCA_002447435.1_ASM244743v1_genomic.fna
6711	Escherichia coli	GCA_000819645.1_ASM81964v1_genomic.fna
6712	Helicobacter pylori	GCA_900120205.1_HE141_09_genomic.fna
6713	Pseudomonas aeruginosa	GCA_002237425.1_ASM223742v1_genomic.fna
6714	Klebsiella pneumoniae	GCA_001908595.1_ASM190859v1_genomic.fna
6715	Bordetella pertussis	GCA_001985325.1_ASM198532v1_genomic.fna
6716	Bordetella pertussis	GCA_001985345.1_ASM198534v1_genomic.fna
6717	Klebsiella pneumoniae	GCA_001908625.1_ASM190862v1_genomic.fna
6718	Pseudomonas aeruginosa	GCA_002442855.1_ASM244285v1_genomic.fna
6719	Helicobacter pylori	GCA_900120225.1_HE136_09_genomic.fna
6720	Escherichia coli	GCA_000827105.1_ASM82710v1_genomic.fna
6721	Mycobacterium tuberculosis	GCA_002447455.1_ASM244745v1_genomic.fna
6722	Listeria monocytogenes	GCA_001999125.1_ASM199912v1_genomic.fna
6723	Staphylococcus aureus	GCA_001548295.1_ASM154829v1_genomic.fna
6724	Campylobacter jejuni	GCA_001563565.1_ASM156356v1_genomic.fna
6725	Salmonella enterica	GCA_000623335.1_ASM62333v1_genomic.fna
6726	Salmonella enterica	GCA_000623355.1_ASM62335v1_genomic.fna
6727	Campylobacter jejuni	GCA_001587015.1_ASM158701v1_genomic.fna
6728	Staphylococcus aureus	GCA_001548415.1_ASM154841v1_genomic.fna
6729	Listeria monocytogenes	GCA_001999145.1_ASM199914v1_genomic.fna

6730	Mycobacterium tuberculosis	GCA_002447475.1_ASM244747v1_genomic.fna
6731	Escherichia coli	GCA_000829985.1_ASM82998v1_genomic.fna
6732	Helicobacter pylori	GCA_900120255.1_HE101_09_genomic.fna
6733	Pseudomonas aeruginosa	GCA_900069025.1_ASM90006902v1_genomic.fna
6734	Klebsiella pneumoniae	GCA_001908655.1_ASM190865v1_genomic.fna
6735	Bordetella pertussis	GCA_001985365.1_ASM198536v1_genomic.fna
6736	Bordetella pertussis	GCA_001985385.1_ASM198538v1_genomic.fna
6737	Klebsiella pneumoniae	GCA_001908675.1_ASM190867v1_genomic.fna
6738	Pseudomonas aeruginosa	GCA_900070375.1_PAO1OR_genomic.fna
6739	Helicobacter pylori	GCA_900120275.1_HE142_09_genomic.fna
6740	Mycobacterium bovis	GCA_001544735.1_ASM154473v1_genomic.fna
6741	Escherichia coli	GCA_000830035.1_ASM83003v1_genomic.fna
6742	Mycobacterium tuberculosis	GCA_002447495.1_ASM244749v1_genomic.fna
6743	Listeria monocytogenes	GCA_001999165.1_ASM199916v1_genomic.fna
6744	Staphylococcus aureus	GCA_001549655.1_ASM154965v1_genomic.fna
6745	Campylobacter jejuni	GCA_001587035.1_ASM158703v1_genomic.fna
6746	Salmonella enterica	GCA_000623375.1_ASM62337v1_genomic.fna
6747	Campylobacter jejuni	GCA_001686905.1_ASM168690v1_genomic.fna
6748	Salmonella enterica	GCA_000623395.2_ASM62339v2_genomic.fna
6749	Staphylococcus aureus	GCA_001549675.1_ASM154967v1_genomic.fna
6750	Listeria monocytogenes	GCA_002043045.1_ASM204304v1_genomic.fna
6751	Mycobacterium tuberculosis	GCA_002447515.1_ASM244751v1_genomic.fna
6752	Escherichia coli	GCA_000831565.1_ASM83156v1_genomic.fna
6753	Mycobacterium bovis	GCA_001544775.1_ASM154477v1_genomic.fna
6754	Helicobacter pylori	GCA_900120315.1_HE170_09_genomic.fna
6755	Pseudomonas aeruginosa	GCA_900095805.1_PA14Or_genomic.fna
6756	Klebsiella pneumoniae	GCA_001908695.1_ASM190869v1_genomic.fna
6757	Bordetella pertussis	GCA_001985405.1_ASM198540v1_genomic.fna
6758	Bordetella pertussis	GCA_001985425.1_ASM198542v1_genomic.fna
6759	Klebsiella pneumoniae	GCA_001908715.1_ASM190871v1_genomic.fna
6760	Pseudomonas aeruginosa	GCA_900149285.1_PcyII-10_genomic.fna
6761	Helicobacter pylori	GCA_900120335.1_HE147_09_genomic.fna
6762	Escherichia coli	GCA_000833145.1_ASM83314v1_genomic.fna
6763	Mycobacterium tuberculosis	GCA_002447535.1_ASM244753v1_genomic.fna

6764	Listeria monocytogenes	GCA_002101275.1_ASM210127v1_genomic.fna
6765	Staphylococcus aureus	GCA_001558795.1_ASM155879v1_genomic.fna
6766	Salmonella enterica	GCA_000623455.2_ASM62345v2_genomic.fna
6767	Campylobacter jejuni	GCA_001717625.1_ASM171762v1_genomic.fna
6768	Campylobacter jejuni	GCA_001721945.1_ASM172194v1_genomic.fna
6769	Salmonella enterica	GCA_000623475.2_ASM62347v2_genomic.fna
6770	Staphylococcus aureus	GCA_001580495.1_ASM158049v1_genomic.fna
6771	Listeria monocytogenes	GCA_002105615.1_ASM210561v1_genomic.fna
6772	Mycobacterium tuberculosis	GCA_002447555.1_ASM244755v1_genomic.fna
6773	Helicobacter pylori	GCA_900149805.1_BCM-300_genomic.fna
6774	Escherichia coli	GCA_000833635.2_ASM83363v2_genomic.fna
6775	Pseudomonas aeruginosa	GCA_900185255.1_NN2_genomic.fna
6776	Klebsiella pneumoniae	GCA_001908875.1_ASM190887v1_genomic.fna
6777	Bordetella pertussis	GCA_001985445.1_ASM198544v1_genomic.fna
6778	Bordetella pertussis	GCA_001985465.1_ASM198546v1_genomic.fna
6779	Klebsiella pneumoniae	GCA_001908895.1_ASM190889v1_genomic.fna
6780	Helicobacter pylori	GCA_900176185.1_HE93_10_genomic.fna
6781	Mycobacterium tuberculosis	GCA_002447575.1_ASM244757v1_genomic.fna
6782	Escherichia coli	GCA_000931565.1_ASM93156v1_genomic.fna
6783	Listeria monocytogenes	GCA_002105635.1_ASM210563v1_genomic.fna
6784	Staphylococcus aureus	GCA_001580515.1_ASM158051v1_genomic.fna
6785	Salmonella enterica	GCA_000623615.2_ASM62361v2_genomic.fna
6786	Campylobacter jejuni	GCA_001721965.1_ASM172196v1_genomic.fna
6787	Campylobacter jejuni	GCA_001721985.1_ASM172198v1_genomic.fna
6788	Salmonella enterica	GCA_000623655.2_ASM62365v2_genomic.fna
6789	Staphylococcus aureus	GCA_001594205.1_ASM159420v1_genomic.fna
6790	Listeria monocytogenes	GCA_002105655.1_ASM210565v1_genomic.fna
6791	Escherichia coli	GCA_000952955.1_EcRV308Chr_genomic.fna
6792	Mycobacterium tuberculosis	GCA_002447595.1_ASM244759v1_genomic.fna
6793	Klebsiella pneumoniae	GCA_001913175.1_ASM191317v1_genomic.fna
6794	Bordetella pertussis	GCA_001985485.1_ASM198548v1_genomic.fna
6795	Bordetella pertussis	GCA_001985505.1_ASM198550v1_genomic.fna
6796	Klebsiella pneumoniae	GCA_001922465.1_ASM192246v1_genomic.fna
6797	Mycobacterium tuberculosis	GCA_002447615.1_ASM244761v1_genomic.fna

6798	Escherichia coli	GCA_000953515.1_EcHMS174Chr_genomic.fna
6799	Listeria monocytogenes	GCA_002105675.1_ASM210567v1_genomic.fna
6800	Staphylococcus aureus	GCA_001611325.1_ASM161132v1_genomic.fna
6801	Salmonella enterica	GCA_000623675.2_ASM62367v2_genomic.fna
6802	Campylobacter jejuni	GCA_001767215.1_ASM176721v1_genomic.fna
6803	Campylobacter jejuni	GCA_001865395.1_ASM186539v1_genomic.fna
6804	Salmonella enterica	GCA_000623715.2_ASM62371v2_genomic.fna
6805	Staphylococcus aureus	GCA_001611345.1_ASM161134v1_genomic.fna
6806	Listeria monocytogenes	GCA_002105695.1_ASM210569v1_genomic.fna
6807	Mycobacterium tuberculosis	GCA_002447635.1_ASM244763v1_genomic.fna
6808	Escherichia coli	GCA_000967155.1_HUSEC2011CHR1_genomic.fna
6809	Klebsiella pneumoniae	GCA_001936035.1_ASM193603v1_genomic.fna
6810	Bordetella pertussis	GCA_001985525.1_ASM198552v1_genomic.fna
6811	Bordetella pertussis	GCA_001985545.1_ASM198554v1_genomic.fna
6812	Klebsiella pneumoniae	GCA_001952835.1_ASM195283v1_genomic.fna
6813	Mycobacterium tuberculosis	GCA_002447655.1_ASM244765v1_genomic.fna
6814	Escherichia coli	GCA_000968515.1_ASM96851v1_genomic.fna
6815	Listeria monocytogenes	GCA_002105715.1_ASM210571v1_genomic.fna
6816	Staphylococcus aureus	GCA_001611365.1_ASM161136v1_genomic.fna
6817	Salmonella enterica	GCA_000623735.2_ASM62373v2_genomic.fna
6818	Campylobacter jejuni	GCA_001865415.1_ASM186541v1_genomic.fna
6819	Campylobacter jejuni	GCA_001865435.1_ASM186543v1_genomic.fna
6820	Salmonella enterica	GCA_000624055.2_ASM62405v2_genomic.fna
6821	Staphylococcus aureus	GCA_001611385.1_ASM161138v1_genomic.fna
6822	Listeria monocytogenes	GCA_002208215.1_ASM220821v1_genomic.fna
6823	Escherichia coli	GCA_000971615.1_ASM97161v1_genomic.fna
6824	Mycobacterium tuberculosis	GCA_002447675.1_ASM244767v1_genomic.fna
6825	Klebsiella pneumoniae	GCA_001952875.1_ASM195287v1_genomic.fna
6826	Bordetella pertussis	GCA_001985565.1_ASM198556v1_genomic.fna
6827	Bordetella pertussis	GCA_001985585.1_ASM198558v1_genomic.fna
6828	Klebsiella pneumoniae	GCA_001952895.1_ASM195289v1_genomic.fna
6829	Mycobacterium tuberculosis	GCA_002447695.1_ASM244769v1_genomic.fna
6830	Escherichia coli	GCA_000974405.1_ASM97440v1_genomic.fna
6831	Listeria monocytogenes	GCA_002213505.1_ASM221350v1_genomic.fna

6832	Staphylococcus aureus	GCA_001611405.1_ASM161140v1_genomic.fna
6833	Campylobacter jejuni	GCA_001865595.1_ASM186559v1_genomic.fna
6834	Salmonella enterica	GCA_000624155.1_ASM62415v1_genomic.fna
6835	Campylobacter jejuni	GCA_001865615.1_ASM186561v1_genomic.fna
6836	Salmonella enterica	GCA_000624175.2_ASM62417v2_genomic.fna
6837	Staphylococcus aureus	GCA_001611425.1_ASM161142v1_genomic.fna
6838	Listeria monocytogenes	GCA_002213545.1_ASM221354v1_genomic.fna
6839	Escherichia coli	GCA_000974465.1_ASM97446v1_genomic.fna
6840	Mycobacterium tuberculosis	GCA_002447715.1_ASM244771v1_genomic.fna
6841	Klebsiella pneumoniae	GCA_001952915.1_ASM195291v1_genomic.fna
6842	Bordetella pertussis	GCA_001985605.1_ASM198560v1_genomic.fna
6843	Bordetella pertussis	GCA_001985625.1_ASM198562v1_genomic.fna
6844	Klebsiella pneumoniae	GCA_001956965.1_ASM195696v1_genomic.fna
6845	Xanthomonas citri	GCA_002240395.1_ASM224039v1_genomic.fna
6846	Mycobacterium tuberculosis	GCA_002447735.1_ASM244773v1_genomic.fna
6847	Escherichia coli	GCA_000974505.1_ASM97450v1_genomic.fna
6848	Listeria monocytogenes	GCA_002213565.1_ASM221356v1_genomic.fna
6849	Staphylococcus aureus	GCA_001618305.1_ASM161830v1_genomic.fna
6850	Campylobacter jejuni	GCA_001870085.1_ASM187008v1_genomic.fna
6851	Salmonella enterica	GCA_000624395.2_ASM62439v2_genomic.fna
6852	Campylobacter jejuni	GCA_001870105.1_ASM187010v1_genomic.fna
6853	Salmonella enterica	GCA_000625855.2_ASM62585v2_genomic.fna
6854	Staphylococcus aureus	GCA_001640885.1_ASM164088v1_genomic.fna
6855	Listeria monocytogenes	GCA_002213585.1_ASM221358v1_genomic.fna
6856	Escherichia coli	GCA_000974535.1_ASM97453v1_genomic.fna
6857	Mycobacterium tuberculosis	GCA_002447755.1_ASM244775v1_genomic.fna
6858	Klebsiella pneumoniae	GCA_002009075.1_ASM200907v1_genomic.fna
6859	Bordetella pertussis	GCA_001985645.1_ASM198564v1_genomic.fna
6860	Bordetella pertussis	GCA_001985665.1_ASM198566v1_genomic.fna
6861	Klebsiella pneumoniae	GCA_002055765.1_ASM205576v1_genomic.fna
6862	Mycobacterium tuberculosis	GCA_002447775.1_ASM244777v1_genomic.fna
6863	Escherichia coli	GCA_000974575.1_ASM97457v1_genomic.fna
6864	Listeria monocytogenes	GCA_002213605.1_ASM221360v1_genomic.fna
6865	Staphylococcus aureus	GCA_001640905.1_ASM164090v1_genomic.fna

6866	Salmonella enterica	GCA_000626095.2_ASM62609v2_genomic.fna
6867	Campylobacter jejuni	GCA_001951235.1_ASM195123v1_genomic.fna
6868	Campylobacter jejuni	GCA_001951255.1_ASM195125v1_genomic.fna
6869	Salmonella enterica	GCA_000626175.1_ASM62617v1_genomic.fna
6870	Staphylococcus aureus	GCA_001640925.1_ASM164092v1_genomic.fna
6871	Listeria monocytogenes	GCA_002213625.1_ASM221362v1_genomic.fna
6872	Escherichia coli	GCA_000974825.1_ASM97482v1_genomic.fna
6873	Mycobacterium tuberculosis	GCA_002447795.1_ASM244779v1_genomic.fna
6874	Klebsiella pneumoniae	GCA_002055855.1_ASM205585v1_genomic.fna
6875	Bordetella pertussis	GCA_001985685.1_ASM198568v1_genomic.fna
6876	Bordetella pertussis	GCA_001985705.1_ASM198570v1_genomic.fna
6877	Klebsiella pneumoniae	GCA_002056385.1_ASM205638v1_genomic.fna
6878	Mycobacterium tuberculosis	GCA_002447815.1_ASM244781v1_genomic.fna
6879	Escherichia coli	GCA_000974865.1_ASM97486v1_genomic.fna
6880	Listeria monocytogenes	GCA_002213645.1_ASM221364v1_genomic.fna
6881	Staphylococcus aureus	GCA_001641025.1_ASM164102v1_genomic.fna
6882	Salmonella enterica	GCA_000626195.1_ASM62619v1_genomic.fna
6883	Campylobacter jejuni	GCA_001951275.1_ASM195127v1_genomic.fna
6884	Campylobacter jejuni	GCA_001951295.1_ASM195129v1_genomic.fna
6885	Salmonella enterica	GCA_000626215.1_ASM62621v1_genomic.fna
6886	Staphylococcus aureus	GCA_001656045.1_ASM165604v1_genomic.fna
6887	Listeria monocytogenes	GCA_002213665.1_ASM221366v1_genomic.fna
6888	Escherichia coli	GCA_000974885.1_ASM97488v1_genomic.fna
6889	Mycobacterium tuberculosis	GCA_002447835.1_ASM244783v1_genomic.fna
6890	Klebsiella pneumoniae	GCA_002056475.1_ASM205647v1_genomic.fna
6891	Bordetella pertussis	GCA_001985725.1_ASM198572v1_genomic.fna
6892	Bordetella pertussis	GCA_001985745.1_ASM198574v1_genomic.fna
6893	Klebsiella pneumoniae	GCA_002113865.1_ASM211386v1_genomic.fna
6894	Mycobacterium tuberculosis	GCA_002447855.1_ASM244785v1_genomic.fna
6895	Escherichia coli	GCA_000981485.1_EcoliK12AG100_genomic.fna
6896	Listeria monocytogenes	GCA_002213685.1_ASM221368v1_genomic.fna
6897	Staphylococcus aureus	GCA_001656075.1_ASM165607v1_genomic.fna
6898	Salmonella enterica	GCA_000626235.1_ASM62623v1_genomic.fna
6899	Campylobacter jejuni	GCA_001951315.1_ASM195131v1_genomic.fna

6900	Campylobacter jejuni	GCA_001951335.1_ASM195133v1_genomic.fna
6901	Salmonella enterica	GCA_000626255.1_ASM62625v1_genomic.fna
6902	Staphylococcus aureus	GCA_001725965.1_ASM172596v1_genomic.fna
6903	Listeria monocytogenes	GCA_002213705.1_ASM221370v1_genomic.fna
6904	Escherichia coli	GCA_000986765.1_ASM98676v1_genomic.fna
6905	Mycobacterium tuberculosis	GCA_002447875.1_ASM244787v1_genomic.fna
6906	Klebsiella pneumoniae	GCA_002116885.1_ASM211688v1_genomic.fna
6907	Bordetella pertussis	GCA_001985765.1_ASM198576v1_genomic.fna
6908	Bordetella pertussis	GCA_001985785.1_ASM198578v1_genomic.fna
6909	Klebsiella pneumoniae	GCA_002156725.1_ASM215672v1_genomic.fna
6910	Mycobacterium tuberculosis	GCA_002447895.1_ASM244789v1_genomic.fna
6911	Escherichia coli	GCA_000987875.1_ASM98787v1_genomic.fna
6912	Listeria monocytogenes	GCA_002213725.1_ASM221372v1_genomic.fna
6913	Staphylococcus aureus	GCA_001879545.1_ASM187954v1_genomic.fna
6914	Campylobacter jejuni	GCA_002024325.1_ASM202432v1_genomic.fna
6915	Salmonella enterica	GCA_000626275.2_ASM62627v2_genomic.fna
6916	Campylobacter jejuni	GCA_002028305.1_ASM202830v1_genomic.fna
6917	Salmonella enterica	GCA_000626295.1_ASM62629v1_genomic.fna
6918	Staphylococcus aureus	GCA_001880265.1_ASM188026v1_genomic.fna
6919	Listeria monocytogenes	GCA_002213745.1_ASM221374v1_genomic.fna
6920	Escherichia coli	GCA_000988355.1_ASM98835v1_genomic.fna
6921	Mycobacterium tuberculosis	GCA_002447915.1_ASM244791v1_genomic.fna
6922	Klebsiella pneumoniae	GCA_002156745.1_ASM215674v1_genomic.fna
6923	Bordetella pertussis	GCA_001985805.1_ASM198580v1_genomic.fna
6924	Bordetella pertussis	GCA_001985825.1_ASM198582v1_genomic.fna
6925	Klebsiella pneumoniae	GCA_002156765.1_ASM215676v1_genomic.fna
6926	Mycobacterium tuberculosis	GCA_002447935.1_ASM244793v1_genomic.fna
6927	Escherichia coli	GCA_000988385.1_ASM98838v1_genomic.fna
6928	Listeria monocytogenes	GCA_002213765.1_ASM221376v1_genomic.fna
6929	Staphylococcus aureus	GCA_001887075.1_ASM188707v1_genomic.fna
6930	Salmonella enterica	GCA_000626335.1_ASM62633v1_genomic.fna
6931	Campylobacter jejuni	GCA_002101355.1_ASM210135v1_genomic.fna
6932	Salmonella enterica	GCA_000626355.1_ASM62635v1_genomic.fna
6933	Campylobacter jejuni	GCA_002209005.1_ASM220900v1_genomic.fna

6934	Staphylococcus aureus	GCA_001900185.1_ASM190018v1_genomic.fna
6935	Listeria monocytogenes	GCA_002213785.1_ASM221378v1_genomic.fna
6936	Escherichia coli	GCA_000988465.1_ASM98846v1_genomic.fna
6937	Mycobacterium tuberculosis	GCA_002447955.1_ASM244795v1_genomic.fna
6938	Klebsiella pneumoniae	GCA_002156785.1_ASM215678v1_genomic.fna
6939	Bordetella pertussis	GCA_001985845.1_ASM198584v1_genomic.fna
6940	Bordetella pertussis	GCA_001985865.1_ASM198586v1_genomic.fna
6941	Pasteurella multocida	GCA_001956675.1_ASM195667v1_genomic.fna
6942	Klebsiella pneumoniae	GCA_002163895.1_ASM216389v1_genomic.fna
6943	Mycobacterium tuberculosis	GCA_002447975.1_ASM244797v1_genomic.fna
6944	Escherichia coli	GCA_001007915.1_ASM100791v1_genomic.fna
6945	Listeria monocytogenes	GCA_002213805.1_ASM221380v1_genomic.fna
6946	Staphylococcus aureus	GCA_001956755.1_ASM195675v1_genomic.fna
6947	Campylobacter jejuni	GCA_002209025.1_ASM220902v1_genomic.fna
6948	Salmonella enterica	GCA_000626375.1_ASM62637v1_genomic.fna
6949	Campylobacter jejuni	GCA_002209045.1_ASM220904v1_genomic.fna
6950	Salmonella enterica	GCA_000626695.2_ASM62669v2_genomic.fna
6951	Staphylococcus aureus	GCA_001956815.1_ASM195681v1_genomic.fna
6952	Listeria monocytogenes	GCA_002213825.1_ASM221382v1_genomic.fna
6953	Mycobacterium tuberculosis	GCA_002447995.1_ASM244799v1_genomic.fna
6954	Escherichia coli	GCA_001020945.2_ASM102094v2_genomic.fna
6955	Klebsiella pneumoniae	GCA_002180075.1_ASM218007v1_genomic.fna
6956	Pasteurella multocida	GCA_002068175.1_ASM206817v1_genomic.fna
6957	Bordetella pertussis	GCA_001985885.1_ASM198588v1_genomic.fna
6958	Bordetella pertussis	GCA_001985905.1_ASM198590v1_genomic.fna
6959	Klebsiella pneumoniae	GCA_002180155.1_ASM218015v1_genomic.fna
6960	Escherichia coli	GCA_001021005.2_ASM102100v2_genomic.fna
6961	Mycobacterium tuberculosis	GCA_002448015.1_ASM244801v1_genomic.fna
6962	Listeria monocytogenes	GCA_002213845.1_ASM221384v1_genomic.fna
6963	Staphylococcus aureus	GCA_001975005.1_ASM197500v1_genomic.fna
6964	Campylobacter jejuni	GCA_002209065.1_ASM220906v1_genomic.fna
6965	Salmonella enterica	GCA_000636135.1_ASM63613v1_genomic.fna
6966	Campylobacter jejuni	GCA_002214785.1_ASM221478v1_genomic.fna
6967	Salmonella enterica	GCA_000715155.2_ASM71515v2_genomic.fna

6968	Staphylococcus aureus	GCA_001975045.1_ASM197504v1_genomic.fna
6969	Listeria monocytogenes	GCA_002213865.1_ASM221386v1_genomic.fna
6970	Mycobacterium tuberculosis	GCA_002448035.1_ASM244803v1_genomic.fna
6971	Escherichia coli	GCA_001021595.1_ASM102159v1_genomic.fna
6972	Klebsiella pneumoniae	GCA_002180175.1_ASM218017v1_genomic.fna
6973	Bordetella pertussis	GCA_001985925.1_ASM198592v1_genomic.fna
6974	Bordetella pertussis	GCA_001985945.1_ASM198594v1_genomic.fna
6975	Klebsiella pneumoniae	GCA_002180255.1_ASM218025v1_genomic.fna
6976	Escherichia coli	GCA_001021615.1_APECO18_genomic.fna
6977	Mycobacterium tuberculosis	GCA_002448055.1_ASM244805v1_genomic.fna
6978	Listeria monocytogenes	GCA_002213885.1_ASM221388v1_genomic.fna
6979	Staphylococcus aureus	GCA_001986135.1_ASM198613v1_genomic.fna
6980	Salmonella enterica	GCA_000742815.1_ASM74281v1_genomic.fna
6981	Campylobacter jejuni	GCA_002224325.1_ASM222432v1_genomic.fna
6982	Campylobacter jejuni	GCA_002224385.1_ASM222438v1_genomic.fna
6983	Salmonella enterica	GCA_000743055.1_ASM74305v1_genomic.fna
6984	Staphylococcus aureus	GCA_002000565.1_ASM200056v1_genomic.fna
6985	Listeria monocytogenes	GCA_002213905.1_ASM221390v1_genomic.fna
6986	Mycobacterium tuberculosis	GCA_002448075.1_ASM244807v1_genomic.fna
6987	Escherichia coli	GCA_001021635.1_ASM102163v1_genomic.fna
6988	Klebsiella pneumoniae	GCA_002180295.1_ASM218029v1_genomic.fna
6989	Bordetella pertussis	GCA_001985965.1_ASM198596v1_genomic.fna
6990	Bordetella pertussis	GCA_001985985.1_ASM198598v1_genomic.fna
6991	Klebsiella pneumoniae	GCA_002180315.1_ASM218031v1_genomic.fna
6992	Mycobacterium tuberculosis	GCA_002448095.1_ASM244809v1_genomic.fna
6993	Escherichia coli	GCA_001029125.1_ASM102912v1_genomic.fna
6994	Listeria monocytogenes	GCA_002213925.1_ASM221392v1_genomic.fna
6995	Staphylococcus aureus	GCA_002000585.1_ASM200058v1_genomic.fna
6996	Salmonella enterica	GCA_000750215.1_ASM75021v1_genomic.fna
6997	Campylobacter jejuni	GCA_002234455.1_ASM223445v1_genomic.fna
6998	Campylobacter jejuni	GCA_002238355.1_ASM223835v1_genomic.fna
6999	Salmonella enterica	GCA_000750255.1_ASM75025v1_genomic.fna
7000	Staphylococcus aureus	GCA_002000605.1_ASM200060v1_genomic.fna
7001	Listeria monocytogenes	GCA_002213945.1_ASM221394v1_genomic.fna

7002	Escherichia coli	GCA_001039415.1_ASM103941v1_genomic.fna
7003	Mycobacterium tuberculosis	GCA_002448115.1_ASM244811v1_genomic.fna
7004	Klebsiella pneumoniae	GCA_002192315.1_ASM219231v1_genomic.fna
7005	Bordetella pertussis	GCA_001986005.1_ASM198600v1_genomic.fna
7006	Bordetella pertussis	GCA_001986025.1_ASM198602v1_genomic.fna
7007	Klebsiella pneumoniae	GCA_002192335.1_ASM219233v1_genomic.fna
7008	Mycobacterium tuberculosis	GCA_002448135.1_ASM244813v1_genomic.fna
7009	Escherichia coli	GCA_001043215.1_ASM104321v1_genomic.fna
7010	Listeria monocytogenes	GCA_002213965.1_ASM221396v1_genomic.fna
7011	Staphylococcus aureus	GCA_002000625.1_ASM200062v1_genomic.fna
7012	Salmonella enterica	GCA_000750295.1_ASM75029v1_genomic.fna
7013	Campylobacter jejuni	GCA_002238375.1_ASM223837v1_genomic.fna
7014	Campylobacter jejuni	GCA_002407125.1_ASM240712v1_genomic.fna
7015	Salmonella enterica	GCA_000750335.1_ASM75033v1_genomic.fna
7016	Staphylococcus aureus	GCA_002000645.1_ASM200064v1_genomic.fna
7017	Listeria monocytogenes	GCA_002213985.1_ASM221398v1_genomic.fna
7018	Escherichia coli	GCA_001051135.1_ASM105113v1_genomic.fna
7019	Mycobacterium tuberculosis	GCA_002448155.1_ASM244815v1_genomic.fna
7020	Klebsiella pneumoniae	GCA_002192375.1_ASM219237v1_genomic.fna
7021	Bordetella pertussis	GCA_001986045.1_ASM198604v1_genomic.fna
7022	Bordetella pertussis	GCA_001986065.1_ASM198606v1_genomic.fna
7023	Klebsiella pneumoniae	GCA_002197225.1_ASM219722v1_genomic.fna
7024	Mycobacterium tuberculosis	GCA_002448175.1_ASM244817v1_genomic.fna
7025	Escherichia coli	GCA_001183645.1_ASM118364v1_genomic.fna
7026	Listeria monocytogenes	GCA_002214005.1_ASM221400v1_genomic.fna
7027	Staphylococcus aureus	GCA_002000665.1_ASM200066v1_genomic.fna
7028	Salmonella enterica	GCA_000750375.1_ASM75037v1_genomic.fna
7029	Campylobacter jejuni	GCA_002587105.1_ASM258710v1_genomic.fna
7030	Campylobacter jejuni	GCA_002587225.1_ASM258722v1_genomic.fna
7031	Salmonella enterica	GCA_000750395.2_ASM75039v2_genomic.fna
7032	Staphylococcus aureus	GCA_002000685.1_ASM200068v1_genomic.fna
7033	Listeria monocytogenes	GCA_002214045.1_ASM221404v1_genomic.fna
7034	Escherichia coli	GCA_001183665.1_ASM118366v1_genomic.fna
7035	Mycobacterium tuberculosis	GCA_002448195.1_ASM244819v1_genomic.fna

7036	Klebsiella pneumoniae	GCA_002197505.1_ASM219750v1_genomic.fna
7037	Bordetella pertussis	GCA_001986085.1_ASM198608v1_genomic.fna
7038	Bordetella pertussis	GCA_001986105.1_ASM198610v1_genomic.fna
7039	Klebsiella pneumoniae	GCA_002202195.1_ASM220219v1_genomic.fna
7040	Mycobacterium tuberculosis	GCA_002448215.1_ASM244821v1_genomic.fna
7041	Escherichia coli	GCA_001183685.1_ASM118368v1_genomic.fna
7042	Listeria monocytogenes	GCA_002214065.1_ASM221406v1_genomic.fna
7043	Staphylococcus aureus	GCA_002000705.1_ASM200070v1_genomic.fna
7044	Salmonella enterica	GCA_000750415.2_ASM75041v2_genomic.fna
7045	Salmonella enterica	GCA_000750435.1_ASM75043v1_genomic.fna
7046	Staphylococcus aureus	GCA_002000725.1_ASM200072v1_genomic.fna
7047	Listeria monocytogenes	GCA_002214085.1_ASM221408v1_genomic.fna
7048	Escherichia coli	GCA_001276585.2_ASM127658v2_genomic.fna
7049	Klebsiella pneumoniae	GCA_002202215.1_ASM220221v1_genomic.fna
7050	Bordetella pertussis	GCA_001986155.1_ASM198615v1_genomic.fna
7051	Bordetella pertussis	GCA_001986175.1_ASM198617v1_genomic.fna
7052	Klebsiella pneumoniae	GCA_002202235.1_ASM220223v1_genomic.fna
7053	Escherichia coli	GCA_001280325.1_ASM128032v1_genomic.fna
7054	Listeria monocytogenes	GCA_002214105.1_ASM221410v1_genomic.fna
7055	Staphylococcus aureus	GCA_002000745.1_ASM200074v1_genomic.fna
7056	Salmonella enterica	GCA_000750455.1_ASM75045v1_genomic.fna
7057	Salmonella enterica	GCA_000750475.1_ASM75047v1_genomic.fna
7058	Staphylococcus aureus	GCA_002000765.1_ASM200076v1_genomic.fna
7059	Listeria monocytogenes	GCA_002214125.1_ASM221412v1_genomic.fna
7060	Escherichia coli	GCA_001280345.1_ASM128034v1_genomic.fna
7061	Klebsiella pneumoniae	GCA_002202255.1_ASM220225v1_genomic.fna
7062	Bordetella pertussis	GCA_001986195.1_ASM198619v1_genomic.fna
7063	Bordetella pertussis	GCA_001986215.1_ASM198621v1_genomic.fna
7064	Klebsiella pneumoniae	GCA_002202275.1_ASM220227v1_genomic.fna
7065	Escherichia coli	GCA_001280385.1_ASM128038v1_genomic.fna
7066	Listeria monocytogenes	GCA_002214145.1_ASM221414v1_genomic.fna
7067	Staphylococcus aureus	GCA_002000785.1_ASM200078v1_genomic.fna
7068	Salmonella enterica	GCA_000750495.1_ASM75049v1_genomic.fna
7069	Salmonella enterica	GCA_000754375.1_ASM75437v1_genomic.fna

7070	Staphylococcus aureus	GCA_002000805.1_ASM200080v1_genomic.fna
7071	Listeria monocytogenes	GCA_002220325.1_ASM222032v1_genomic.fna
7072	Escherichia coli	GCA_001280405.1_ASM128040v1_genomic.fna
7073	Klebsiella pneumoniae	GCA_002209405.1_ASM220940v1_genomic.fna
7074	Bordetella pertussis	GCA_001986235.1_ASM198623v1_genomic.fna
7075	Bordetella pertussis	GCA_001986255.1_ASM198625v1_genomic.fna
7076	Klebsiella pneumoniae	GCA_002211905.1_ASM221190v1_genomic.fna
7077	Escherichia coli	GCA_001307215.1_ASM130721v1_genomic.fna
7078	Listeria monocytogenes	GCA_002285655.1_ASM228565v1_genomic.fna
7079	Staphylococcus aureus	GCA_002000825.1_ASM200082v1_genomic.fna
7080	Salmonella enterica	GCA_000756465.1_ASM75646v1_genomic.fna
7081	Salmonella enterica	GCA_000818075.1_ASM81807v1_genomic.fna
7082	Staphylococcus aureus	GCA_002000845.1_ASM200084v1_genomic.fna
7083	Listeria monocytogenes	GCA_002285835.1_ASM228583v1_genomic.fna
7084	Escherichia coli	GCA_001308065.1_ASM130806v1_genomic.fna
7085	Klebsiella pneumoniae	GCA_002243445.1_ASM224344v1_genomic.fna
7086	Bordetella pertussis	GCA_001986275.1_ASM198627v1_genomic.fna
7087	Bordetella pertussis	GCA_001986295.1_ASM198629v1_genomic.fna
7088	Klebsiella pneumoniae	GCA_002249975.1_ASM224997v1_genomic.fna
7089	Haemophilus influenzae	GCA_000016465.1_ASM1646v1_genomic.fna
7090	Listeria monocytogenes	GCA_002557735.1_ASM255773v1_genomic.fna
7091	Escherichia coli	GCA_001308125.1_ASM130812v1_genomic.fna
7092	Staphylococcus aureus	GCA_002000865.1_ASM200086v1_genomic.fna
7093	Salmonella enterica	GCA_000818115.1_ASM81811v1_genomic.fna
7094	Salmonella enterica	GCA_000828595.1_ASM82859v1_genomic.fna
7095	Staphylococcus aureus	GCA_002025125.1_ASM202512v1_genomic.fna
7096	Escherichia coli	GCA_001308165.1_ASM130816v1_genomic.fna
7097	Listeria monocytogenes	GCA_002557815.1_ASM255781v1_genomic.fna
7098	Haemophilus influenzae	GCA_000016485.1_ASM1648v1_genomic.fna
7099	Xanthomonas oryzae	GCA_000007385.1_ASM738v1_genomic.fna
7100	Klebsiella pneumoniae	GCA_002269295.1_ASM226929v1_genomic.fna
7101	Bordetella pertussis	GCA_001986315.1_ASM198631v1_genomic.fna
7102	Bordetella pertussis	GCA_001986335.1_ASM198633v1_genomic.fna
7103	Klebsiella pneumoniae	GCA_002278055.1_ASM227805v1_genomic.fna

7104	Listeria monocytogenes	GCA_900187225.1_51881_G01_genomic.fna
7105	Escherichia coli	GCA_001420935.1_ASM142093v1_genomic.fna
7106	Staphylococcus aureus	GCA_002025145.1_ASM202514v1_genomic.fna
7107	Salmonella enterica	GCA_000940895.2_ASM94089v2_genomic.fna
7108	Salmonella enterica	GCA_000940935.1_ASM94093v1_genomic.fna
7109	Staphylococcus aureus	GCA_002085525.1_ASM208552v1_genomic.fna
7110	Escherichia coli	GCA_001420955.1_ASM142095v1_genomic.fna
7111	Klebsiella pneumoniae	GCA_002307335.1_ASM230733v1_genomic.fna
7112	Bordetella pertussis	GCA_001986355.1_ASM198635v1_genomic.fna
7113	Bordetella pertussis	GCA_001986375.1_ASM198637v1_genomic.fna
7114	Klebsiella pneumoniae	GCA_002386225.1_ASM238622v1_genomic.fna
7115	Escherichia coli	GCA_001442495.1_ASM144249v1_genomic.fna
7116	Staphylococcus aureus	GCA_002088995.1_ASM208899v1_genomic.fna
7117	Salmonella enterica	GCA_000940975.1_ASM94097v1_genomic.fna
7118	Salmonella enterica	GCA_000941015.2_ASM94101v2_genomic.fna
7119	Staphylococcus aureus	GCA_002089035.1_ASM208903v1_genomic.fna
7120	Escherichia coli	GCA_001455385.1_ASM145538v1_genomic.fna
7121	Klebsiella pneumoniae	GCA_002587165.1_ASM258716v1_genomic.fna
7122	Bordetella pertussis	GCA_001986415.1_ASM198641v1_genomic.fna
7123	Bordetella pertussis	GCA_001986435.1_ASM198643v1_genomic.fna
7124	Klebsiella pneumoniae	GCA_002587885.1_ASM258788v1_genomic.fna
7125	Escherichia coli	GCA_001469815.1_ASM146981v1_genomic.fna
7126	Staphylococcus aureus	GCA_002089055.1_ASM208905v1_genomic.fna
7127	Salmonella enterica	GCA_000953495.1_SINFA_genomic.fna
7128	Salmonella enterica	GCA_000963535.2_ASM96353v2_genomic.fna
7129	Staphylococcus aureus	GCA_002089075.1_ASM208907v1_genomic.fna
7130	Escherichia coli	GCA_001485455.1_ASM148545v1_genomic.fna
7131	Klebsiella pneumoniae	GCA_002591075.1_ASM259107v1_genomic.fna
7132	Bordetella pertussis	GCA_001986455.1_ASM198645v1_genomic.fna
7133	Bordetella pertussis	GCA_001986475.1_ASM198647v1_genomic.fna
7134	Escherichia coli	GCA_001513615.1_ASM151361v1_genomic.fna
7135	Staphylococcus aureus	GCA_002089095.1_ASM208909v1_genomic.fna
7136	Salmonella enterica	GCA_000973645.1_ASM97364v1_genomic.fna
7137	Salmonella enterica	GCA_000973665.2_ASM97366v2_genomic.fna

7138	Staphylococcus aureus	GCA_002089115.1_ASM208911v1_genomic.fna
7139	Escherichia coli	GCA_001513635.1_ASM151363v1_genomic.fna
7140	Bordetella pertussis	GCA_001986495.1_ASM198649v1_genomic.fna
7141	Bordetella pertussis	GCA_001986515.1_ASM198651v1_genomic.fna
7142	Escherichia coli	GCA_001515725.1_ASM151572v1_genomic.fna
7143	Staphylococcus aureus	GCA_002097595.1_ASM209759v1_genomic.fna
7144	Salmonella enterica	GCA_000973685.1_ASM97368v1_genomic.fna
7145	Salmonella enterica	GCA_000988525.3_ASM98852v3_genomic.fna
7146	Staphylococcus aureus	GCA_002140115.1_ASM214011v1_genomic.fna
7147	Escherichia coli	GCA_001542675.2_ASM154267v2_genomic.fna
7148	Bordetella pertussis	GCA_001986535.1_ASM198653v1_genomic.fna
7149	Bordetella pertussis	GCA_001986555.1_ASM198655v1_genomic.fna
7150	Escherichia coli	GCA_001544635.1_ASM154463v1_genomic.fna
7151	Staphylococcus aureus	GCA_002202075.1_ASM220207v1_genomic.fna
7152	Salmonella enterica	GCA_000993725.1_ASM99372v1_genomic.fna
7153	Salmonella enterica	GCA_001006525.1_ASM100652v1_genomic.fna
7154	Staphylococcus aureus	GCA_002202095.1_ASM220209v1_genomic.fna
7155	Escherichia coli	GCA_001558995.2_ASM155899v2_genomic.fna
7156	Bordetella pertussis	GCA_001986575.1_ASM198657v1_genomic.fna
7157	Bordetella pertussis	GCA_001986595.1_ASM198659v1_genomic.fna
7158	Escherichia coli	GCA_001559615.2_ASM155961v2_genomic.fna
7159	Staphylococcus aureus	GCA_002204555.1_ASM220455v1_genomic.fna
7160	Salmonella enterica	GCA_001048035.2_ERL103914_genomic.fna
7161	Salmonella enterica	GCA_001048375.2_M223_genomic.fna
7162	Staphylococcus aureus	GCA_002204575.1_ASM220457v1_genomic.fna
7163	Escherichia coli	GCA_001559635.1_ASM155963v1_genomic.fna
7164	Bordetella pertussis	GCA_001986615.1_ASM198661v1_genomic.fna
7165	Bordetella pertussis	GCA_001986635.1_ASM198663v1_genomic.fna
7166	Escherichia coli	GCA_001559655.1_ASM155965v1_genomic.fna
7167	Staphylococcus aureus	GCA_002209325.1_ASM220932v1_genomic.fna
7168	Salmonella enterica	GCA_001079465.2_STyphi_1553_1_genomic.fna
7169	Salmonella enterica	GCA_001088345.2_052042_2_genomic.fna
7170	Staphylococcus aureus	GCA_002214665.1_ASM221466v1_genomic.fna
7171	Escherichia coli	GCA_001559675.1_ASM155967v1_genomic.fna

7172	Bacillus subtilis	GCA_001596535.1_ASM159653v1_genomic.fna
7173	Bordetella pertussis	GCA_001986655.1_ASM198665v1_genomic.fna
7174	Bordetella pertussis	GCA_001986675.1_ASM198667v1_genomic.fna
7175	Escherichia coli	GCA_001566335.1_ASM156633v1_genomic.fna
7176	Staphylococcus aureus	GCA_002310395.1_ASM231039v1_genomic.fna
7177	Salmonella enterica	GCA_001095585.2_ERL114000_genomic.fna
7178	Salmonella enterica	GCA_001104165.2_E00-7866_genomic.fna
7179	Staphylococcus aureus	GCA_002310435.1_ASM231043v1_genomic.fna
7180	Escherichia coli	GCA_001566615.1_ASM156661v1_genomic.fna
7181	Bordetella pertussis	GCA_001986695.1_ASM198669v1_genomic.fna
7182	Bordetella pertussis	GCA_001986715.1_ASM198671v1_genomic.fna
7183	Escherichia coli	GCA_001566635.1_ASM156663v1_genomic.fna
7184	Staphylococcus aureus	GCA_002355355.1_ASM235535v1_genomic.fna
7185	Salmonella enterica	GCA_001104885.3_10349_1_89_2_genomic.fna
7186	Salmonella enterica	GCA_001106745.2_UI_2120_genomic.fna
7187	Staphylococcus aureus	GCA_002356675.1_ASM235667v1_genomic.fna
7188	Escherichia coli	GCA_001566655.1_ASM156665v1_genomic.fna
7189	Bordetella pertussis	GCA_001986735.1_ASM198673v1_genomic.fna
7190	Bordetella pertussis	GCA_001986755.1_ASM198675v1_genomic.fna
7191	Escherichia coli	GCA_001566675.1_ASM156667v1_genomic.fna
7192	Staphylococcus aureus	GCA_002386245.1_ASM238624v1_genomic.fna
7193	Salmonella enterica	GCA_001118185.2_H12ESR00394-001A_genomic.fna
7194	Salmonella enterica	GCA_001119245.2_76_1292_genomic.fna
7195	Staphylococcus aureus	GCA_002442975.1_ASM244297v1_genomic.fna
7196	Escherichia coli	GCA_001577325.1_ASM157732v1_genomic.fna
7197	Bordetella pertussis	GCA_001986775.1_ASM198677v1_genomic.fna
7198	Staphylococcus haemolyticus	GCA_000972725.1_ASM97272v1_genomic.fna
7199	Bordetella pertussis	GCA_001986795.1_ASM198679v1_genomic.fna
7200	Escherichia coli	GCA_001593565.1_ASM159356v1_genomic.fna
7201	Staphylococcus aureus	GCA_002633765.1_ASM263376v1_genomic.fna
7202	Salmonella enterica	GCA_001121865.2_404Ty_genomic.fna
7203	Salmonella enterica	GCA_001127485.2_ERL041834_genomic.fna
7204	Staphylococcus aureus	GCA_002633785.1_ASM263378v1_genomic.fna
7205	Escherichia coli	GCA_001610755.1_ASM161075v1_genomic.fna

7206	Bordetella pertussis	GCA_001986815.1_ASM198681v1_genomic.fna
7207	Bordetella pertussis	GCA_001986835.1_ASM198683v1_genomic.fna
7208	Escherichia coli	GCA_001612475.1_ASM161247v1_genomic.fna
7209	Staphylococcus aureus	GCA_002633805.1_ASM263380v1_genomic.fna
7210	Salmonella enterica	GCA_001135805.2_ERL072973_genomic.fna
7211	Salmonella enterica	GCA_001148125.2_ERL024120_genomic.fna
7212	Staphylococcus aureus	GCA_002633825.1_ASM263382v1_genomic.fna
7213	Escherichia coli	GCA_001612495.1_ASM161249v1_genomic.fna
7214	Bordetella pertussis	GCA_001986855.1_ASM198685v1_genomic.fna
7215	Bordetella pertussis	GCA_001986875.1_ASM198687v1_genomic.fna
7216	Escherichia coli	GCA_001617565.1_ASM161756v1_genomic.fna
7217	Staphylococcus aureus	GCA_002633845.1_ASM263384v1_genomic.fna
7218	Salmonella enterica	GCA_001148305.2_ERL082356_genomic.fna
7219	Salmonella enterica	GCA_001157245.2_ERL12148_genomic.fna
7220	Staphylococcus aureus	GCA_002633865.1_ASM263386v1_genomic.fna
7221	Escherichia coli	GCA_001618325.1_ASM161832v1_genomic.fna
7222	Bordetella pertussis	GCA_001986895.1_ASM198689v1_genomic.fna
7223	Bordetella pertussis	GCA_001986915.1_ASM198691v1_genomic.fna
7224	Escherichia coli	GCA_001618345.2_ASM161834v2_genomic.fna
7225	Salmonella enterica	GCA_001163025.2_H12ESR04734-001A_genomic.fna
7226	Staphylococcus aureus	GCA_900004855.1_BB155_genomic.fna
7227	Staphylococcus aureus	GCA_900017775.1_NZAK3_genomic.fna
7228	Salmonella enterica	GCA_001165785.2_ERL024919_genomic.fna
7229	Escherichia coli	GCA_001618365.1_ASM161836v1_genomic.fna
7230	Bordetella pertussis	GCA_001986935.1_ASM198693v1_genomic.fna
7231	Bordetella pertussis	GCA_001986955.1_ASM198695v1_genomic.fna
7232	Escherichia coli	GCA_001644725.1_ASM164472v1_genomic.fna
7233	Staphylococcus aureus	GCA_900092595.1_Sa_Newman_UoM_genomic.fna
7234	Salmonella enterica	GCA_001185215.1_ASM118521v1_genomic.fna
7235	Salmonella enterica	GCA_001185245.1_ASM118524v1_genomic.fna
7236	Staphylococcus aureus	GCA_900096745.1_AUS0325_genomic.fna
7237	Escherichia coli	GCA_001644745.1_ASM164474v1_genomic.fna
7238	Bordetella pertussis	GCA_001986975.1_ASM198697v1_genomic.fna
7239	Bordetella pertussis	GCA_001986995.1_ASM198699v1_genomic.fna

7240	Escherichia coli	GCA_001650275.1_ASM165027v1_genomic.fna
7241	Staphylococcus aureus	GCA_900129335.1_PP_HGAG_QV30_2SC_T4_genomic.fna
7242	Salmonella enterica	GCA_001240865.2_Ty3_193_genomic.fna
7243	Salmonella enterica	GCA_001278315.1_ASM127831v1_genomic.fna
7244	Escherichia coli	GCA_001650295.1_ASM165029v1_genomic.fna
7245	Bordetella pertussis	GCA_001987015.1_ASM198701v1_genomic.fna
7246	Bordetella pertussis	GCA_001987035.1_ASM198703v1_genomic.fna
7247	Escherichia coli	GCA_001651925.2_ASM165192v2_genomic.fna
7248	Salmonella enterica	GCA_001293505.1_ASM129350v1_genomic.fna
7249	Salmonella enterica	GCA_001302605.1_ASM130260v1_genomic.fna
7250	Escherichia coli	GCA_001651945.1_ASM165194v1_genomic.fna
7251	Bordetella pertussis	GCA_001987055.1_ASM198705v1_genomic.fna
7252	Bordetella pertussis	GCA_001987075.1_ASM198707v1_genomic.fna
7253	Shigella flexneri	GCA_001579965.1_ASM157996v1_genomic.fna
7254	Escherichia coli	GCA_001651965.2_ASM165196v2_genomic.fna
7255	Salmonella enterica	GCA_001305235.1_ASM130523v1_genomic.fna
7256	Salmonella enterica	GCA_001305815.1_ASM130581v1_genomic.fna
7257	Escherichia coli	GCA_001660565.1_ASM166056v1_genomic.fna
7258	Bordetella pertussis	GCA_001987095.1_ASM198709v1_genomic.fna
7259	Bordetella pertussis	GCA_001987115.1_ASM198711v1_genomic.fna
7260	Escherichia coli	GCA_001660585.1_ASM166058v1_genomic.fna
7261	Salmonella enterica	GCA_001305835.1_ASM130583v1_genomic.fna
7262	Salmonella enterica	GCA_001356455.2_1016889_genomic.fna
7263	Escherichia coli	GCA_001663075.1_ASM166307v1_genomic.fna
7264	Bordetella pertussis	GCA_001987135.1_ASM198713v1_genomic.fna
7265	Bordetella pertussis	GCA_001987155.1_ASM198715v1_genomic.fna
7266	Escherichia coli	GCA_001663475.1_ASM166347v1_genomic.fna
7267	Salmonella enterica	GCA_001357935.2_80_2002_genomic.fna
7268	Salmonella enterica	GCA_001359015.2_129_0238_genomic.fna
7269	Escherichia coli	GCA_001675145.1_ASM167514v1_genomic.fna
7270	Bordetella pertussis	GCA_001987175.1_ASM198717v1_genomic.fna
7271	Bordetella pertussis	GCA_001987195.1_ASM198719v1_genomic.fna
7272	Escherichia coli	GCA_001677475.1_ASM167747v1_genomic.fna
7273	Salmonella enterica	GCA_001360555.2_2010_7898_genomic.fna

7274	Salmonella enterica	GCA_001361075.2_Ty585_genomic.fna
7275	Escherichia coli	GCA_001677495.1_ASM167749v1_genomic.fna
7276	Bordetella pertussis	GCA_001987215.1_ASM198721v1_genomic.fna
7277	Bordetella pertussis	GCA_001987235.1_ASM198723v1_genomic.fna
7278	Escherichia coli	GCA_001677515.1_ASM167751v1_genomic.fna
7279	Salmonella enterica	GCA_001362095.2_034151_4_genomic.fna
7280	Salmonella enterica	GCA_001362135.2_11909_3_genomic.fna
7281	Escherichia coli	GCA_001678925.1_ASM167892v1_genomic.fna
7282	Bordetella pertussis	GCA_001987255.1_ASM198725v1_genomic.fna
7283	Bordetella pertussis	GCA_001987275.1_ASM198727v1_genomic.fna
7284	Escherichia coli	GCA_001678965.1_ASM167896v1_genomic.fna
7285	Salmonella enterica	GCA_001362175.2_ERL12960_genomic.fna
7286	Salmonella enterica	GCA_001362195.2_H12ESR00755-001A_genomic.fna
7287	Escherichia coli	GCA_001679985.1_ASM167998v1_genomic.fna
7288	Bordetella pertussis	GCA_001987295.1_ASM198729v1_genomic.fna
7289	Bordetella pertussis	GCA_001987315.1_ASM198731v1_genomic.fna
7290	Escherichia coli	GCA_001682305.2_ASM168230v2_genomic.fna
7291	Salmonella enterica	GCA_001362335.2_Ty2_genomic.fna
7292	Salmonella enterica	GCA_001364695.2_Ty3_243_genomic.fna
7293	Escherichia coli	GCA_001683435.1_ASM168343v1_genomic.fna
7294	Bordetella pertussis	GCA_001987335.1_ASM198733v1_genomic.fna
7295	Bordetella pertussis	GCA_001987355.1_ASM198735v1_genomic.fna
7296	Clostridium botulinum	GCA_000816945.1_ASM81694v1_genomic.fna
7297	Escherichia coli	GCA_001693315.1_ASM169331v1_genomic.fna
7298	Salmonella enterica	GCA_001409135.1_10259_genomic.fna
7299	Salmonella enterica	GCA_001409155.1_98_11262_genomic.fna
7300	Escherichia coli	GCA_001693635.1_ASM169363v1_genomic.fna
7301	Bordetella pertussis	GCA_001987375.1_ASM198737v1_genomic.fna
7302	Bordetella pertussis	GCA_001987395.1_ASM198739v1_genomic.fna
7303	Escherichia coli	GCA_001695515.1_ASM169551v1_genomic.fna
7304	Salmonella enterica	GCA_001409175.1_99_3134_genomic.fna
7305	Salmonella enterica	GCA_001409195.1_C2346_genomic.fna
7306	Escherichia coli	GCA_001721125.1_ASM172112v1_genomic.fna
7307	Bordetella pertussis	GCA_001987415.1_ASM198741v1_genomic.fna

7308	Bordetella pertussis	GCA_001987435.1_ASM198743v1_genomic.fna
7309	Escherichia coli	GCA_001721205.1_ASM172120v1_genomic.fna
7310	Salmonella enterica	GCA_001441205.1_ASM144120v1_genomic.fna
7311	Salmonella enterica	GCA_001441225.1_ASM144122v1_genomic.fna
7312	Escherichia coli	GCA_001721225.1_ASM172122v1_genomic.fna
7313	Bordetella pertussis	GCA_001987455.1_ASM198745v1_genomic.fna
7314	Bordetella pertussis	GCA_001987475.1_ASM198747v1_genomic.fna
7315	Escherichia coli	GCA_001721525.1_ASM172152v1_genomic.fna
7316	Salmonella enterica	GCA_001441245.1_ASM144124v1_genomic.fna
7317	Salmonella enterica	GCA_001447095.1_ASM144709v1_genomic.fna
7318	Escherichia coli	GCA_001723505.1_ASM172350v1_genomic.fna
7319	Bordetella pertussis	GCA_001987495.1_ASM198749v1_genomic.fna
7320	Bordetella pertussis	GCA_001987515.1_ASM198751v1_genomic.fna
7321	Escherichia coli	GCA_001735705.1_ASM173570v1_genomic.fna
7322	Salmonella enterica	GCA_001447115.1_ASM144711v1_genomic.fna
7323	Salmonella enterica	GCA_001454965.1_ASM145496v1_genomic.fna
7324	Escherichia coli	GCA_001750845.1_ASM175084v1_genomic.fna
7325	Bacillus anthracis	GCA_000512775.1_ASM51277v1_genomic.fna
7326	Bordetella pertussis	GCA_001987535.1_ASM198753v1_genomic.fna
7327	Bordetella pertussis	GCA_001987555.1_ASM198755v1_genomic.fna
7328	Bacillus anthracis	GCA_000747335.1_ASM74733v1_genomic.fna
7329	Escherichia coli	GCA_001753445.1_ASM175344v1_genomic.fna
7330	Salmonella enterica	GCA_001457675.1_NCTC10384_genomic.fna
7331	Salmonella enterica	GCA_001484025.1_ASM148402v1_genomic.fna
7332	Escherichia coli	GCA_001753465.1_ASM175346v1_genomic.fna
7333	Bacillus anthracis	GCA_000747375.1_ASM74737v1_genomic.fna
7334	Bordetella pertussis	GCA_001987575.1_ASM198757v1_genomic.fna
7335	Bordetella pertussis	GCA_001987595.1_ASM198759v1_genomic.fna
7336	Bacillus anthracis	GCA_002208785.1_ASM220878v1_genomic.fna
7337	Escherichia coli	GCA_001753485.1_ASM175348v1_genomic.fna
7338	Salmonella enterica	GCA_001535315.2_ASM153531v2_genomic.fna
7339	Salmonella enterica	GCA_001540845.1_SO4698_09_genomic.fna
7340	Escherichia coli	GCA_001753505.1_ASM175350v1_genomic.fna
7341	Bordetella pertussis	GCA_001987615.1_ASM198761v1_genomic.fna

7342	Bordetella pertussis	GCA_001987635.1_ASM198763v1_genomic.fna
7343	Escherichia coli	GCA_001753525.1_ASM175352v1_genomic.fna
7344	Salmonella enterica	GCA_001558355.1_ASM155835v1_genomic.fna
7345	Salmonella enterica	GCA_001576255.1_ASM157625v1_genomic.fna
7346	Escherichia coli	GCA_001753545.1_ASM175354v1_genomic.fna
7347	Bordetella pertussis	GCA_001987655.1_ASM198765v1_genomic.fna
7348	Bordetella pertussis	GCA_001987675.1_ASM198767v1_genomic.fna
7349	Escherichia coli	GCA_001753565.1_ASM175356v1_genomic.fna
7350	Salmonella enterica	GCA_001576275.1_ASM157627v1_genomic.fna
7351	Salmonella enterica	GCA_001577505.1_ASM157750v1_genomic.fna
7352	Escherichia coli	GCA_001806265.1_ASM180626v1_genomic.fna
7353	Bordetella pertussis	GCA_001987695.1_ASM198769v1_genomic.fna
7354	Bordetella pertussis	GCA_001987715.1_ASM198771v1_genomic.fna
7355	Escherichia coli	GCA_001806285.1_ASM180628v1_genomic.fna
7356	Salmonella enterica	GCA_001612515.1_ASM161251v1_genomic.fna
7357	Salmonella enterica	GCA_001617585.1_ASM161758v1_genomic.fna
7358	Escherichia coli	GCA_001860505.1_ASM186050v1_genomic.fna
7359	Bordetella pertussis	GCA_001987735.1_ASM198773v1_genomic.fna
7360	Bordetella pertussis	GCA_001987755.1_ASM198775v1_genomic.fna
7361	Escherichia coli	GCA_001865295.1_ASM186529v1_genomic.fna
7362	Salmonella enterica	GCA_001620115.1_ASM162011v1_genomic.fna
7363	Salmonella enterica	GCA_001620135.2_ASM162013v2_genomic.fna
7364	Escherichia coli	GCA_001874485.1_ASM187448v1_genomic.fna
7365	Bordetella pertussis	GCA_001987775.1_ASM198777v1_genomic.fna
7366	Bordetella pertussis	GCA_001987795.1_ASM198779v1_genomic.fna
7367	Escherichia coli	GCA_001886535.1_ASM188653v1_genomic.fna
7368	Salmonella enterica	GCA_001620155.1_ASM162015v1_genomic.fna
7369	Salmonella enterica	GCA_001620205.2_ASM162020v2_genomic.fna
7370	Escherichia coli	GCA_001886555.1_ASM188655v1_genomic.fna
7371	Bordetella pertussis	GCA_001987815.1_ASM198781v1_genomic.fna
7372	Bordetella pertussis	GCA_001987835.1_ASM198783v1_genomic.fna
7373	Escherichia coli	GCA_001886575.1_ASM188657v1_genomic.fna
7374	Salmonella enterica	GCA_001620225.1_ASM162022v1_genomic.fna
7375	Salmonella enterica	GCA_001623545.2_ASM162354v2_genomic.fna

7376	Escherichia coli	GCA_001886755.1_ASM188675v1_genomic.fna
7377	Bordetella pertussis	GCA_001987855.1_ASM198785v1_genomic.fna
7378	Bordetella pertussis	GCA_001987875.1_ASM198787v1_genomic.fna
7379	Escherichia coli	GCA_001886895.1_ASM188689v1_genomic.fna
7380	Salmonella enterica	GCA_001623585.1_ASM162358v1_genomic.fna
7381	Salmonella enterica	GCA_001623625.1_ASM162362v1_genomic.fna
7382	Escherichia coli	GCA_001886935.1_ASM188693v1_genomic.fna
7383	Bordetella pertussis	GCA_001987895.1_ASM198789v1_genomic.fna
7384	Bordetella pertussis	GCA_001987915.1_ASM198791v1_genomic.fna
7385	Escherichia coli	GCA_001888075.1_ASM188807v1_genomic.fna
7386	Salmonella enterica	GCA_001623645.1_ASM162364v1_genomic.fna
7387	Salmonella enterica	GCA_001623665.2_ASM162366v2_genomic.fna
7388	Escherichia coli	GCA_001890205.1_ASM189020v1_genomic.fna
7389	Bordetella pertussis	GCA_001987935.1_ASM198793v1_genomic.fna
7390	Bordetella pertussis	GCA_001987955.1_ASM198795v1_genomic.fna
7391	Escherichia coli	GCA_001890225.1_ASM189022v1_genomic.fna
7392	Salmonella enterica	GCA_001623685.1_ASM162368v1_genomic.fna
7393	Salmonella enterica	GCA_001623705.1_ASM162370v1_genomic.fna
7394	Escherichia coli	GCA_001890245.1_ASM189024v1_genomic.fna
7395	Bordetella pertussis	GCA_001987975.1_ASM198797v1_genomic.fna
7396	Bordetella pertussis	GCA_001987995.1_ASM198799v1_genomic.fna
7397	Escherichia coli	GCA_001890265.1_ASM189026v1_genomic.fna
7398	Salmonella enterica	GCA_001623725.1_ASM162372v1_genomic.fna
7399	Salmonella enterica	GCA_001623745.2_ASM162374v2_genomic.fna
7400	Escherichia coli	GCA_001890285.1_ASM189028v1_genomic.fna
7401	Bordetella pertussis	GCA_001988015.1_ASM198801v1_genomic.fna
7402	Bordetella pertussis	GCA_001988035.1_ASM198803v1_genomic.fna
7403	Escherichia coli	GCA_001890305.1_ASM189030v1_genomic.fna
7404	Salmonella enterica	GCA_001623765.2_ASM162376v2_genomic.fna
7405	Salmonella enterica	GCA_001623775.1_ASM162377v1_genomic.fna
7406	Escherichia coli	GCA_001890325.1_ASM189032v1_genomic.fna
7407	Bordetella pertussis	GCA_001988055.1_ASM198805v1_genomic.fna
7408	Bordetella pertussis	GCA_001988075.1_ASM198807v1_genomic.fna
7409	Escherichia coli	GCA_001890345.1_ASM189034v1_genomic.fna

7410	Salmonella enterica	GCA_001623805.1_ASM162380v1_genomic.fna
7411	Salmonella enterica	GCA_001623825.2_ASM162382v2_genomic.fna
7412	Escherichia coli	GCA_001890365.1_ASM189036v1_genomic.fna
7413	Bordetella pertussis	GCA_001988095.1_ASM198809v1_genomic.fna
7414	Bordetella pertussis	GCA_001988115.1_ASM198811v1_genomic.fna
7415	Escherichia coli	GCA_001900295.1_ASM190029v1_genomic.fna
7416	Salmonella enterica	GCA_001623845.1_ASM162384v1_genomic.fna
7417	Salmonella enterica	GCA_001628755.1_ASM162875v1_genomic.fna
7418	Escherichia coli	GCA_001900315.1_ASM190031v1_genomic.fna
7419	Bordetella pertussis	GCA_001988135.1_ASM198813v1_genomic.fna
7420	Bordetella pertussis	GCA_001988155.1_ASM198815v1_genomic.fna
7421	Escherichia coli	GCA_001900335.1_ASM190033v1_genomic.fna
7422	Salmonella enterica	GCA_001629755.1_ASM162975v1_genomic.fna
7423	Salmonella enterica	GCA_001629775.1_ASM162977v1_genomic.fna
7424	Escherichia coli	GCA_001900355.1_ASM190035v1_genomic.fna
7425	Bordetella pertussis	GCA_001988175.1_ASM198817v1_genomic.fna
7426	Bordetella pertussis	GCA_001988195.1_ASM198819v1_genomic.fna
7427	Escherichia coli	GCA_001900375.1_ASM190037v1_genomic.fna
7428	Salmonella enterica	GCA_001647045.1_ASM164704v1_genomic.fna
7429	Salmonella enterica	GCA_001647755.1_ASM164775v1_genomic.fna
7430	Escherichia coli	GCA_001900395.1_ASM190039v1_genomic.fna
7431	Bordetella pertussis	GCA_001988215.1_ASM198821v1_genomic.fna
7432	Bordetella pertussis	GCA_001988235.1_ASM198823v1_genomic.fna
7433	Escherichia coli	GCA_001900415.1_ASM190041v1_genomic.fna
7434	Salmonella enterica	GCA_001689935.1_ASM168993v1_genomic.fna
7435	Salmonella enterica	GCA_001690005.1_ASM169000v1_genomic.fna
7436	Escherichia coli	GCA_001900435.1_ASM190043v1_genomic.fna
7437	Bordetella pertussis	GCA_001988255.1_ASM198825v1_genomic.fna
7438	Bordetella pertussis	GCA_001988275.1_ASM198827v1_genomic.fna
7439	Escherichia coli	GCA_001900455.1_ASM190045v1_genomic.fna
7440	Salmonella enterica	GCA_001690035.1_ASM169003v1_genomic.fna
7441	Salmonella enterica	GCA_001690055.1_ASM169005v1_genomic.fna
7442	Escherichia coli	GCA_001900475.1_ASM190047v1_genomic.fna
7443	Bordetella pertussis	GCA_001988295.1_ASM198829v1_genomic.fna

7444	Bordetella pertussis	GCA_001988315.1_ASM198831v1_genomic.fna
7445	Escherichia coli	GCA_001900495.1_ASM190049v1_genomic.fna
7446	Salmonella enterica	GCA_001690075.1_ASM169007v1_genomic.fna
7447	Salmonella enterica	GCA_001690095.1_ASM169009v1_genomic.fna
7448	Escherichia coli	GCA_001900515.1_ASM190051v1_genomic.fna
7449	Bordetella pertussis	GCA_001988335.1_ASM198833v1_genomic.fna
7450	Bordetella pertussis	GCA_001988355.1_ASM198835v1_genomic.fna
7451	Escherichia coli	GCA_001900535.1_ASM190053v1_genomic.fna
7452	Salmonella enterica	GCA_001690115.1_ASM169011v1_genomic.fna
7453	Salmonella enterica	GCA_001690135.1_ASM169013v1_genomic.fna
7454	Escherichia coli	GCA_001900555.1_ASM190055v1_genomic.fna
7455	Bordetella pertussis	GCA_001988375.1_ASM198837v1_genomic.fna
7456	Bordetella pertussis	GCA_001988395.1_ASM198839v1_genomic.fna
7457	Escherichia coli	GCA_001900575.1_ASM190057v1_genomic.fna
7458	Salmonella enterica	GCA_001692495.1_ASM169249v1_genomic.fna
7459	Salmonella enterica	GCA_001692515.1_ASM169251v1_genomic.fna
7460	Escherichia coli	GCA_001900595.1_ASM190059v1_genomic.fna
7461	Bordetella pertussis	GCA_001988415.1_ASM198841v1_genomic.fna
7462	Bordetella pertussis	GCA_001988435.1_ASM198843v1_genomic.fna
7463	Escherichia coli	GCA_001900615.1_ASM190061v1_genomic.fna
7464	Salmonella enterica	GCA_001692535.1_ASM169253v1_genomic.fna
7465	Salmonella enterica	GCA_001692555.1_ASM169255v1_genomic.fna
7466	Escherichia coli	GCA_001900635.1_ASM190063v1_genomic.fna
7467	Bordetella pertussis	GCA_001988455.1_ASM198845v1_genomic.fna
7468	Bordetella pertussis	GCA_001988475.1_ASM198847v1_genomic.fna
7469	Escherichia coli	GCA_001900655.1_ASM190065v1_genomic.fna
7470	Salmonella enterica	GCA_001692575.1_ASM169257v1_genomic.fna
7471	Salmonella enterica	GCA_001692595.1_ASM169259v1_genomic.fna
7472	Escherichia coli	GCA_001900675.1_ASM190067v1_genomic.fna
7473	Streptococcus equi	GCA_002202295.1_ASM220229v1_genomic.fna
7474	Bordetella pertussis	GCA_001988495.1_ASM198849v1_genomic.fna
7475	Bordetella pertussis	GCA_001988515.1_ASM198851v1_genomic.fna
7476	Escherichia coli	GCA_001900695.1_ASM190069v1_genomic.fna
7477	Salmonella enterica	GCA_001692615.1_ASM169261v1_genomic.fna

7478	Salmonella enterica	GCA_001692635.1_ASM169263v1_genomic.fna
7479	Escherichia coli	GCA_001900715.1_ASM190071v1_genomic.fna
7480	Bordetella pertussis	GCA_001988535.1_ASM198853v1_genomic.fna
7481	Bordetella pertussis	GCA_001988555.1_ASM198855v1_genomic.fna
7482	Escherichia coli	GCA_001900735.1_ASM190073v1_genomic.fna
7483	Salmonella enterica	GCA_001692655.1_ASM169265v1_genomic.fna
7484	Salmonella enterica	GCA_001705055.1_ASM170505v1_genomic.fna
7485	Escherichia coli	GCA_001900775.1_ASM190077v1_genomic.fna
7486	Burkholderia cenocepacia	GCA_001718515.1_ASM171851v1_genomic.fna
7487	Bordetella pertussis	GCA_001988575.1_ASM198857v1_genomic.fna
7488	Bordetella pertussis	GCA_001988595.1_ASM198859v1_genomic.fna
7489	Escherichia coli	GCA_001900795.1_ASM190079v1_genomic.fna
7490	Salmonella enterica	GCA_001705365.1_ASM170536v1_genomic.fna
7491	Salmonella enterica	GCA_001729225.1_ASM172922v1_genomic.fna
7492	Escherichia coli	GCA_001900815.1_ASM190081v1_genomic.fna
7493	Bordetella pertussis	GCA_001988615.1_ASM198861v1_genomic.fna
7494	Bordetella pertussis	GCA_001988635.1_ASM198863v1_genomic.fna
7495	Escherichia coli	GCA_001900835.1_ASM190083v1_genomic.fna
7496	Salmonella enterica	GCA_001886995.1_ASM188699v1_genomic.fna
7497	Salmonella enterica	GCA_001887015.1_ASM188701v1_genomic.fna
7498	Escherichia coli	GCA_001900885.1_ASM190088v1_genomic.fna
7499	Bordetella pertussis	GCA_001988655.1_ASM198865v1_genomic.fna
7500	Bordetella pertussis	GCA_001988675.1_ASM198867v1_genomic.fna
7501	Escherichia coli	GCA_001900905.1_ASM190090v1_genomic.fna
7502	Salmonella enterica	GCA_001890165.1_ASM189016v1_genomic.fna
7503	Salmonella enterica	GCA_001890185.1_ASM189018v1_genomic.fna
7504	Escherichia coli	GCA_001900925.1_ASM190092v1_genomic.fna
7505	Bordetella pertussis	GCA_001988695.1_ASM198869v1_genomic.fna
7506	Bordetella pertussis	GCA_001988715.1_ASM198871v1_genomic.fna
7507	Escherichia coli	GCA_001900945.1_ASM190094v1_genomic.fna
7508	Salmonella enterica	GCA_001890445.1_ASM189044v1_genomic.fna
7509	Salmonella enterica	GCA_001922165.1_ASM192216v1_genomic.fna
7510	Escherichia coli	GCA_001900965.1_ASM190096v1_genomic.fna
7511	Bordetella pertussis	GCA_001988735.1_ASM198873v1_genomic.fna

7512	Bordetella pertussis	GCA_001988755.1_ASM198875v1_genomic.fna
7513	Escherichia coli	GCA_001900985.1_ASM190098v1_genomic.fna
7514	Salmonella enterica	GCA_001922185.1_ASM192218v1_genomic.fna
7515	Salmonella enterica	GCA_001931555.1_ASM193155v1_genomic.fna
7516	Escherichia coli	GCA_001901005.1_ASM190100v1_genomic.fna
7517	Bordetella pertussis	GCA_001988775.1_ASM198877v1_genomic.fna
7518	Bordetella pertussis	GCA_001988795.1_ASM198879v1_genomic.fna
7519	Escherichia coli	GCA_001901025.1_ASM190102v1_genomic.fna
7520	Salmonella enterica	GCA_001931575.1_ASM193157v1_genomic.fna
7521	Salmonella enterica	GCA_001931595.1_ASM193159v1_genomic.fna
7522	Escherichia coli	GCA_001901045.1_ASM190104v1_genomic.fna
7523	Bordetella pertussis	GCA_001988815.1_ASM198881v1_genomic.fna
7524	Bordetella pertussis	GCA_001988835.1_ASM198883v1_genomic.fna
7525	Escherichia coli	GCA_001901065.1_ASM190106v1_genomic.fna
7526	Salmonella enterica	GCA_001931615.1_ASM193161v1_genomic.fna
7527	Salmonella enterica	GCA_001941405.1_ASM194140v1_genomic.fna
7528	Escherichia coli	GCA_001901085.1_ASM190108v1_genomic.fna
7529	Bordetella pertussis	GCA_001988855.1_ASM198885v1_genomic.fna
7530	Bordetella pertussis	GCA_001988875.1_ASM198887v1_genomic.fna
7531	Escherichia coli	GCA_001901105.1_ASM190110v1_genomic.fna
7532	Salmonella enterica	GCA_001952995.1_ASM195299v1_genomic.fna
7533	Salmonella enterica	GCA_001953015.1_ASM195301v1_genomic.fna
7534	Escherichia coli	GCA_001901125.1_ASM190112v1_genomic.fna
7535	Bordetella pertussis	GCA_001988895.1_ASM198889v1_genomic.fna
7536	Bordetella pertussis	GCA_001988915.1_ASM198891v1_genomic.fna
7537	Escherichia coli	GCA_001901145.1_ASM190114v1_genomic.fna
7538	Salmonella enterica	GCA_001953035.1_ASM195303v1_genomic.fna
7539	Salmonella enterica	GCA_001953675.1_ASM195367v1_genomic.fna
7540	Escherichia coli	GCA_001901165.1_ASM190116v1_genomic.fna
7541	Bordetella pertussis	GCA_001988995.1_ASM198899v1_genomic.fna
7542	Bordetella pertussis	GCA_001989015.1_ASM198901v1_genomic.fna
7543	Escherichia coli	GCA_001901185.1_ASM190118v1_genomic.fna
7544	Salmonella enterica	GCA_001953695.1_ASM195369v1_genomic.fna
7545	Salmonella enterica	GCA_001956735.1_ASM195673v1_genomic.fna

7546	Escherichia coli	GCA_001901215.1_ASM190121v1_genomic.fna
7547	Bordetella pertussis	GCA_001989035.1_ASM198903v1_genomic.fna
7548	Bordetella pertussis	GCA_001989055.1_ASM198905v1_genomic.fna
7549	Escherichia coli	GCA_001901315.1_ASM190131v1_genomic.fna
7550	Salmonella enterica	GCA_001969425.1_ASM196942v1_genomic.fna
7551	Salmonella enterica	GCA_001973275.2_ASM197327v2_genomic.fna
7552	Escherichia coli	GCA_001901365.1_ASM190136v1_genomic.fna
7553	Bordetella pertussis	GCA_001989075.1_ASM198907v1_genomic.fna
7554	Bordetella pertussis	GCA_001989095.1_ASM198909v1_genomic.fna
7555	Escherichia coli	GCA_001901405.1_ASM190140v1_genomic.fna
7556	Salmonella enterica	GCA_001975245.1_ASM197524v1_genomic.fna
7557	Salmonella enterica	GCA_001975265.1_ASM197526v1_genomic.fna
7558	Escherichia coli	GCA_001901425.1_ASM190142v1_genomic.fna
7559	Bordetella pertussis	GCA_001989115.1_ASM198911v1_genomic.fna
7560	Bordetella pertussis	GCA_001989135.1_ASM198913v1_genomic.fna
7561	Escherichia coli	GCA_001901445.1_ASM190144v1_genomic.fna
7562	Salmonella enterica	GCA_001975285.1_ASM197528v1_genomic.fna
7563	Salmonella enterica	GCA_001975305.1_ASM197530v1_genomic.fna
7564	Escherichia coli	GCA_001901465.1_ASM190146v1_genomic.fna
7565	Bordetella pertussis	GCA_001989155.1_ASM198915v1_genomic.fna
7566	Bordetella pertussis	GCA_001989175.1_ASM198917v1_genomic.fna
7567	Escherichia coli	GCA_001932515.1_ASM193251v1_genomic.fna
7568	Salmonella enterica	GCA_001975325.1_ASM197532v1_genomic.fna
7569	Salmonella enterica	GCA_001975345.1_ASM197534v1_genomic.fna
7570	Escherichia coli	GCA_001936315.1_ASM193631v1_genomic.fna
7571	Bordetella pertussis	GCA_001989195.1_ASM198919v1_genomic.fna
7572	Bordetella pertussis	GCA_001989215.1_ASM198921v1_genomic.fna
7573	Escherichia coli	GCA_001969285.1_ASM196928v1_genomic.fna
7574	Salmonella enterica	GCA_001975365.1_ASM197536v1_genomic.fna
7575	Salmonella enterica	GCA_001975385.1_ASM197538v1_genomic.fna
7576	Escherichia coli	GCA_001999185.1_ASM199918v1_genomic.fna
7577	Bordetella pertussis	GCA_001989235.1_ASM198923v1_genomic.fna
7578	Bordetella pertussis	GCA_001989255.1_ASM198925v1_genomic.fna
7579	Escherichia coli	GCA_002007705.1_ASM200770v1_genomic.fna

7580	Salmonella enterica	GCA_001975405.1_ASM197540v1_genomic.fna
7581	Salmonella enterica	GCA_001975425.1_ASM197542v1_genomic.fna
7582	Escherichia coli	GCA_002009315.1_ASM200931v1_genomic.fna
7583	Bordetella pertussis	GCA_001989275.1_ASM198927v1_genomic.fna
7584	Bordetella pertussis	GCA_001989295.1_ASM198929v1_genomic.fna
7585	Escherichia coli	GCA_002011945.1_ASM201194v1_genomic.fna
7586	Salmonella enterica	GCA_001975445.1_ASM197544v1_genomic.fna
7587	Salmonella enterica	GCA_001975515.1_ASM197551v1_genomic.fna
7588	Escherichia coli	GCA_002011965.1_ASM201196v1_genomic.fna
7589	Bordetella pertussis	GCA_001989315.1_ASM198931v1_genomic.fna
7590	Bordetella pertussis	GCA_001989335.1_ASM198933v1_genomic.fna
7591	Escherichia coli	GCA_002011985.1_ASM201198v1_genomic.fna
7592	Salmonella enterica	GCA_001975555.1_ASM197555v1_genomic.fna
7593	Salmonella enterica	GCA_001975605.1_ASM197560v1_genomic.fna
7594	Escherichia coli	GCA_002012005.1_ASM201200v1_genomic.fna
7595	Bordetella pertussis	GCA_002018515.1_ASM201851v1_genomic.fna
7596	Bordetella pertussis	GCA_002021795.1_ASM202179v1_genomic.fna
7597	Escherichia coli	GCA_002012025.1_ASM201202v1_genomic.fna
7598	Salmonella enterica	GCA_001975625.1_ASM197562v1_genomic.fna
7599	Salmonella enterica	GCA_001975645.1_ASM197564v1_genomic.fna
7600	Escherichia coli	GCA_002012045.1_ASM201204v1_genomic.fna
7601	Bordetella pertussis	GCA_002068675.1_ASM206867v1_genomic.fna
7602	Bordetella pertussis	GCA_002068975.1_ASM206897v1_genomic.fna
7603	Escherichia coli	GCA_002012065.1_ASM201206v1_genomic.fna
7604	Salmonella enterica	GCA_001989635.1_ASM198963v1_genomic.fna
7605	Salmonella enterica	GCA_002009155.1_ASM200915v1_genomic.fna
7606	Escherichia coli	GCA_002012085.1_ASM201208v1_genomic.fna
7607	Bordetella pertussis	GCA_002069155.1_ASM206915v1_genomic.fna
7608	Bordetella pertussis	GCA_002240435.1_ASM224043v1_genomic.fna
7609	Escherichia coli	GCA_002012105.1_ASM201210v1_genomic.fna
7610	Salmonella enterica	GCA_002119485.1_ASM211948v1_genomic.fna
7611	Salmonella enterica	GCA_002128365.1_ASM212836v1_genomic.fna
7612	Escherichia coli	GCA_002012125.1_ASM201212v1_genomic.fna
7613	Bordetella pertussis	GCA_002240475.1_ASM224047v1_genomic.fna

7614	Bordetella pertussis	GCA_002240495.1_ASM224049v1_genomic.fna
7615	Escherichia coli	GCA_002012145.1_ASM201214v1_genomic.fna
7616	Salmonella enterica	GCA_002128385.1_ASM212838v1_genomic.fna
7617	Salmonella enterica	GCA_002163995.1_ASM216399v1_genomic.fna
7618	Escherichia coli	GCA_002012165.1_ASM201216v1_genomic.fna
7619	Bordetella pertussis	GCA_002240515.1_ASM224051v1_genomic.fna
7620	Bordetella pertussis	GCA_002285955.1_ASM228595v1_genomic.fna
7621	Escherichia coli	GCA_002012185.1_ASM201218v1_genomic.fna
7622	Salmonella enterica	GCA_002196535.1_ASM219653v1_genomic.fna
7623	Salmonella enterica	GCA_002201855.1_ASM220185v1_genomic.fna
7624	Escherichia coli	GCA_002012205.1_ASM201220v1_genomic.fna
7625	Bordetella pertussis	GCA_002285975.1_ASM228597v1_genomic.fna
7626	Bordetella pertussis	GCA_002285995.1_ASM228599v1_genomic.fna
7627	Escherichia coli	GCA_002012225.1_ASM201222v1_genomic.fna
7628	Salmonella enterica	GCA_002208255.1_ASM220825v1_genomic.fna
7629	Salmonella enterica	GCA_002208315.1_ASM220831v1_genomic.fna
7630	Escherichia coli	GCA_002012245.1_ASM201224v1_genomic.fna
7631	Bordetella pertussis	GCA_002286015.1_ASM228601v1_genomic.fna
7632	Bordetella pertussis	GCA_002286035.1_ASM228603v1_genomic.fna
7633	Escherichia coli	GCA_002012265.1_ASM201226v1_genomic.fna
7634	Yersinia pestis	GCA_002005285.1_ASM200528v1_genomic.fna
7635	Salmonella enterica	GCA_002209205.1_ASM220920v1_genomic.fna
7636	Salmonella enterica	GCA_002210025.1_ASM221002v1_genomic.fna
7637	Escherichia coli	GCA_002012305.1_ASM201230v1_genomic.fna
7638	Bordetella pertussis	GCA_002286055.1_ASM228605v1_genomic.fna
7639	Bordetella pertussis	GCA_002286075.1_ASM228607v1_genomic.fna
7640	Escherichia coli	GCA_002024865.1_ASM202486v1_genomic.fna
7641	Salmonella enterica	GCA_002211565.1_ASM221156v1_genomic.fna
7642	Salmonella enterica	GCA_002211585.1_ASM221158v1_genomic.fna
7643	Escherichia coli	GCA_002055605.1_ASM205560v1_genomic.fna
7644	Bordetella pertussis	GCA_002286095.1_ASM228609v1_genomic.fna
7645	Bordetella pertussis	GCA_002286115.1_ASM228611v1_genomic.fna
7646	Escherichia coli	GCA_002055635.1_ASM205563v1_genomic.fna
7647	Salmonella enterica	GCA_002211825.1_ASM221182v1_genomic.fna

7648	Salmonella enterica	GCA_002211845.1_ASM221184v1_genomic.fna
7649	Escherichia coli	GCA_002056065.1_ASM205606v1_genomic.fna
7650	Bordetella pertussis	GCA_002286135.1_ASM228613v1_genomic.fna
7651	Bordetella pertussis	GCA_002286155.1_ASM228615v1_genomic.fna
7652	Escherichia coli	GCA_002056145.1_ASM205614v1_genomic.fna
7653	Salmonella enterica	GCA_002211865.1_ASM221186v1_genomic.fna
7654	Salmonella enterica	GCA_002211965.1_ASM221196v1_genomic.fna
7655	Escherichia coli	GCA_002056635.1_ASM205663v1_genomic.fna
7656	Bordetella pertussis	GCA_002286175.1_ASM228617v1_genomic.fna
7657	Bordetella pertussis	GCA_002286295.1_ASM228629v1_genomic.fna
7658	Escherichia coli	GCA_002057245.1_ASM205724v1_genomic.fna
7659	Salmonella enterica	GCA_002220345.1_ASM222034v1_genomic.fna
7660	Salmonella enterica	GCA_002224405.1_ASM222440v1_genomic.fna
7661	Escherichia coli	GCA_002057355.1_ASM205735v1_genomic.fna
7662	Bordetella pertussis	GCA_002286315.1_ASM228631v1_genomic.fna
7663	Bordetella pertussis	GCA_002286335.1_ASM228633v1_genomic.fna
7664	Escherichia coli	GCA_002058765.1_ASM205876v1_genomic.fna
7665	Salmonella enterica	GCA_002234475.1_ASM223447v1_genomic.fna
7666	Salmonella enterica	GCA_002234515.1_ASM223451v1_genomic.fna
7667	Escherichia coli	GCA_002077195.1_ASM207719v1_genomic.fna
7668	Bordetella pertussis	GCA_002286355.1_ASM228635v1_genomic.fna
7669	Bordetella pertussis	GCA_002286375.1_ASM228637v1_genomic.fna
7670	Escherichia coli	GCA_002078275.1_ASM207827v1_genomic.fna
7671	Salmonella enterica	GCA_002234735.1_ASM223473v1_genomic.fna
7672	Salmonella enterica	GCA_002234755.1_ASM223475v1_genomic.fna
7673	Escherichia coli	GCA_002078295.1_ASM207829v1_genomic.fna
7674	Bordetella pertussis	GCA_002310915.1_ASM231091v1_genomic.fna
7675	Bordetella pertussis	GCA_002442615.1_ASM244261v1_genomic.fna
7676	Escherichia coli	GCA_002079225.1_ASM207922v1_genomic.fna
7677	Salmonella enterica	GCA_002234775.1_ASM223477v1_genomic.fna
7678	Salmonella enterica	GCA_002234795.1_ASM223479v1_genomic.fna
7679	Escherichia coli	GCA_002090355.1_ASM209035v1_genomic.fna
7680	Bordetella pertussis	GCA_002442635.1_ASM244263v1_genomic.fna
7681	Bordetella pertussis	GCA_002442655.1_ASM244265v1_genomic.fna

7682	Escherichia coli	GCA_002105735.1_ASM210573v1_genomic.fna
7683	Salmonella enterica	GCA_002234855.1_ASM223485v1_genomic.fna
7684	Salmonella enterica	GCA_002238275.1_ASM223827v1_genomic.fna
7685	Escherichia coli	GCA_002116715.1_ASM211671v1_genomic.fna
7686	Bordetella pertussis	GCA_002442675.1_ASM244267v1_genomic.fna
7687	Bordetella pertussis	GCA_002442695.1_ASM244269v1_genomic.fna
7688	Escherichia coli	GCA_002118095.1_ASM211809v1_genomic.fna
7689	Salmonella enterica	GCA_002290205.1_ASM229020v1_genomic.fna
7690	Salmonella enterica	GCA_002313085.1_ASM231308v1_genomic.fna
7691	Escherichia coli	GCA_002142675.1_ASM214267v1_genomic.fna
7692	Bordetella pertussis	GCA_002442715.1_ASM244271v1_genomic.fna
7693	Bordetella pertussis	GCA_002442735.1_ASM244273v1_genomic.fna
7694	Escherichia coli	GCA_002142695.1_ASM214269v1_genomic.fna
7695	Salmonella enterica	GCA_002313105.1_ASM231310v1_genomic.fna
7696	Salmonella enterica	GCA_002313125.1_ASM231312v1_genomic.fna
7697	Escherichia coli	GCA_002142715.1_ASM214271v1_genomic.fna
7698	Bordetella pertussis	GCA_002442755.1_ASM244275v1_genomic.fna
7699	Bordetella pertussis	GCA_002442775.1_ASM244277v1_genomic.fna
7700	Escherichia coli	GCA_002156825.1_ASM215682v1_genomic.fna
7701	Salmonella enterica	GCA_002386165.1_ASM238616v1_genomic.fna
7702	Salmonella enterica	GCA_002386325.1_ASM238632v1_genomic.fna
7703	Escherichia coli	GCA_002156845.1_ASM215684v1_genomic.fna
7704	Bordetella pertussis	GCA_002442795.1_ASM244279v1_genomic.fna
7705	Bordetella pertussis	GCA_002442815.1_ASM244281v1_genomic.fna
7706	Escherichia coli	GCA_002157245.1_ASM215724v1_genomic.fna
7707	Salmonella enterica	GCA_002504105.1_ASM250410v1_genomic.fna
7708	Salmonella enterica	GCA_002504125.1_ASM250412v1_genomic.fna
7709	Escherichia coli	GCA_002163655.1_ASM216365v1_genomic.fna
7710	Bordetella pertussis	GCA_900186845.1_46338_E02_genomic.fna
7711	Bordetella pertussis	GCA_900187345.1_42650_A02_genomic.fna
7712	Escherichia coli	GCA_002163695.1_ASM216369v1_genomic.fna
7713	Salmonella enterica	GCA_900086565.1_39641_H02_genomic.fna
7714	Salmonella enterica	GCA_900166885.1_39674_D01_genomic.fna
7715	Escherichia coli	GCA_002163935.1_ASM216393v1_genomic.fna

7716	Escherichia coli	GCA_002163955.1_ASM216395v1_genomic.fna
7717	Salmonella enterica	GCA_900184385.1_STMU2UK_genomic.fna
7718	Salmonella enterica	GCA_900185485.1_BL60006_genomic.fna
7719	Escherichia coli	GCA_002180055.1_ASM218005v1_genomic.fna
7720	Escherichia coli	GCA_002180095.1_ASM218009v1_genomic.fna
7721	Salmonella enterica	GCA_900205245.1_ISP_03_07467_98_genomic.fna
7722	Salmonella enterica	GCA_900205255.1_1554_genomic.fna
7723	Escherichia coli	GCA_002180135.1_ASM218013v1_genomic.fna
7724	Escherichia coli	GCA_002180195.1_ASM218019v1_genomic.fna
7725	Salmonella enterica	GCA_900205265.1_lupe_GEN0059_5_genomic.fna
7726	Salmonella enterica	GCA_900205275.1_403Ty_genomic.fna
7727	Escherichia coli	GCA_002180215.1_ASM218021v1_genomic.fna
7728	Escherichia coli	GCA_002180275.1_ASM218027v1_genomic.fna
7729	Salmonella enterica	GCA_900205295.1_E98-3139_genomic.fna
7730	Escherichia coli	GCA_002192275.1_ASM219227v1_genomic.fna
7731	Escherichia coli	GCA_002192295.1_ASM219229v1_genomic.fna
7732	Escherichia coli	GCA_002193095.1_ASM219309v1_genomic.fna
7733	Escherichia coli	GCA_002196475.1_ASM219647v1_genomic.fna
7734	Escherichia coli	GCA_002196495.1_ASM219649v1_genomic.fna
7735	Streptococcus pyogenes	GCA_000732385.1_ASM73238v1_genomic.fna
7736	Streptococcus pyogenes	GCA_001535505.1_ASM153550v1_genomic.fna
7737	Escherichia coli	GCA_002201835.1_ASM220183v1_genomic.fna
7738	Escherichia coli	GCA_002202175.1_ASM220217v1_genomic.fna
7739	Streptococcus pyogenes	GCA_001547815.1_ASM154781v1_genomic.fna
7740	Escherichia coli	GCA_002208865.1_ASM220886v1_genomic.fna
7741	Escherichia coli	GCA_002209105.1_ASM220910v1_genomic.fna
7742	Escherichia coli	GCA_002211725.1_ASM221172v1_genomic.fna
7743	Escherichia coli	GCA_002214205.1_ASM221420v1_genomic.fna
7744	Escherichia coli	GCA_002214745.1_ASM221474v1_genomic.fna
7745	Escherichia coli	GCA_002215095.1_ASM221509v1_genomic.fna
7746	Escherichia coli	GCA_002215115.1_ASM221511v1_genomic.fna
7747	Escherichia coli	GCA_002220215.1_ASM222021v1_genomic.fna
7748	Escherichia coli	GCA_002220265.1_ASM222026v1_genomic.fna
7749	Escherichia coli	GCA_002237285.1_ASM223728v1_genomic.fna

7750	Escherichia coli	GCA_002237305.1_ASM223730v1_genomic.fna
7751	Escherichia coli	GCA_002237325.1_ASM223732v1_genomic.fna
7752	Escherichia coli	GCA_002249955.1_ASM224995v1_genomic.fna
7753	Escherichia coli	GCA_002269325.1_ASM226932v1_genomic.fna
7754	Escherichia coli	GCA_002278115.2_ASM227811v2_genomic.fna
7755	Escherichia coli	GCA_002285755.1_ASM228575v1_genomic.fna
7756	Escherichia coli	GCA_002302315.1_ASM230231v1_genomic.fna
7757	Escherichia coli	GCA_002302335.1_ASM230233v1_genomic.fna
7758	Escherichia coli	GCA_002310555.1_ASM231055v1_genomic.fna
7759	Escherichia coli	GCA_002310575.1_ASM231057v1_genomic.fna
7760	Escherichia coli	GCA_002310595.1_ASM231059v1_genomic.fna
7761	Escherichia coli	GCA_002310615.1_ASM231061v1_genomic.fna
7762	Escherichia coli	GCA_002310635.1_ASM231063v1_genomic.fna
7763	Escherichia coli	GCA_002310655.1_ASM231065v1_genomic.fna
7764	Escherichia coli	GCA_002310675.1_ASM231067v1_genomic.fna
7765	Escherichia coli	GCA_002310695.1_ASM231069v1_genomic.fna
7766	Escherichia coli	GCA_002310715.1_ASM231071v1_genomic.fna
7767	Escherichia coli	GCA_002310735.1_ASM231073v1_genomic.fna
7768	Escherichia coli	GCA_002357875.1_ASM235787v1_genomic.fna
7769	Escherichia coli	GCA_002357895.1_ASM235789v1_genomic.fna
7770	Escherichia coli	GCA_002357915.1_ASM235791v1_genomic.fna
7771	Escherichia coli	GCA_002393365.1_ASM239336v1_genomic.fna
7772	Escherichia coli	GCA_002393465.1_ASM239346v1_genomic.fna
7773	Escherichia coli	GCA_002587005.1_ASM258700v1_genomic.fna
7774	Escherichia coli	GCA_002589795.1_ASM258979v1_genomic.fna
7775	Escherichia coli	GCA_002591135.1_ASM259113v1_genomic.fna
7776	Escherichia coli	GCA_002716885.1_ASM271688v1_genomic.fna
7777	Escherichia coli	GCA_900092615.1_PRJEB14041_genomic.fna
7778	Escherichia coli	GCA_900096795.1_Ecoli_AG100_Sample3_Doxycycline_Assembly_genomic.fna
7779	Escherichia coli	GCA_900096845.1_Ecoli_AG100_Sample3_M9_Assembly_genomic.fna
7780	Escherichia coli	GCA_900149875.1_EPEC1_genomic.fna
7781	Escherichia coli	GCA_900174625.1_WI1-reUpload_genomic.fna
7782	Escherichia coli	GCA_900186905.1_49923_G01_genomic.fna
7783	Bacillus thuringiensis	GCA_000774075.2_ASM77407v2_genomic.fna

7784	Bacillus thuringiensis	GCA_001675515.1_ASM167551v1_genomic.fna
7785	Legionella pneumophila	GCA_000277025.1_ASM27702v1_genomic.fna
7786	Legionella pneumophila	GCA_000277065.1_ASM27706v1_genomic.fna
7787	Legionella pneumophila	GCA_000404245.1_ASM40424v1_genomic.fna
7788	Enterobacter cloacae	GCA_001620245.1_ASM162024v1_genomic.fna
7789	Bordetella pertussis	GCA_001558395.1_ASM155839v1_genomic.fna
7790	Vibrio cholerae	GCA_001045415.1_ASM104541v1_genomic.fna
7791	Burkholderia pseudomallei	GCA_000494855.1_ASM49485v1_genomic.fna
7792	Burkholderia pseudomallei	GCA_002111225.1_ASM211122v1_genomic.fna
7793	Burkholderia pseudomallei	GCA_002111285.1_ASM211128v1_genomic.fna
7794	Enterococcus faecium	GCA_001594345.1_ASM159434v1_genomic.fna
7795	Enterococcus faecium	GCA_900094185.1_Ef_DMG1500501_genomic.fna
7796	Helicobacter pylori	GCA_000262655.1_ASM26265v1_genomic.fna
7797	Helicobacter pylori	GCA_001549855.1_ASM154985v1_genomic.fna
7798	Helicobacter pylori	GCA_001549875.1_ASM154987v1_genomic.fna
7799	Campylobacter coli	GCA_000583775.1_ASM58377v1_genomic.fna
7800	Campylobacter coli	GCA_001305715.1_ASM130571v1_genomic.fna
7801	Streptococcus agalactiae	GCA_000427055.1_ILRI112_genomic.fna
7802	Streptococcus agalactiae	GCA_000599965.1_ASM59996v1_genomic.fna
7803	Streptococcus agalactiae	GCA_000636115.1_ASM63611v1_genomic.fna
7804	Streptococcus agalactiae	GCA_000967445.1_ASM96744v1_genomic.fna
7805	Streptococcus agalactiae	GCA_001190865.1_ASM119086v1_genomic.fna
7806	Streptococcus agalactiae	GCA_001655175.1_ASM165517v1_genomic.fna
7807	Streptococcus agalactiae	GCA_001908255.1_ASM190825v1_genomic.fna
7808	Streptococcus suis	GCA_000014305.1_ASM1430v1_genomic.fna
7809	Streptococcus suis	GCA_000014325.1_ASM1432v1_genomic.fna
7810	Streptococcus suis	GCA_900143575.1_LS9N_genomic.fna
7811	Bacillus cereus	GCA_000724585.1_ASM72458v1_genomic.fna
7812	Shigella sonnei	GCA_002224465.1_ASM222446v1_genomic.fna
7813	Clostridioides difficile	GCA_000210415.1_ASM21041v1_genomic.fna
7814	Clostridioides difficile	GCA_000210455.1_ASM21045v1_genomic.fna
7815	Clostridioides difficile	GCA_000211235.1_ASM21123v1_genomic.fna
7816	Clostridioides difficile	GCA_001010825.1_ASM101082v1_genomic.fna
7817	Clostridioides difficile	GCA_001447175.1_ASM144717v1_genomic.fna

7818	Clostridioides difficile	GCA_001984465.1_ASM198446v1_genomic.fna
7819	Campylobacter jejuni	GCA_000184085.1_ASM18408v1_genomic.fna
7820	Campylobacter jejuni	GCA_001506325.1_ASM150632v1_genomic.fna
7821	Listeria monocytogenes	GCA_000382925.1_ASM38292v1_genomic.fna
7822	Listeria monocytogenes	GCA_000382945.1_ASM38294v1_genomic.fna
7823	Acinetobacter baumannii	GCA_000015425.1_ASM1542v1_genomic.fna
7824	Acinetobacter baumannii	GCA_000189735.2_ASM18973v2_genomic.fna
7825	Acinetobacter baumannii	GCA_000307975.2_ASM30797v2_genomic.fna
7826	Acinetobacter baumannii	GCA_002009115.1_ASM200911v1_genomic.fna
7827	Pseudomonas aeruginosa	GCA_001465155.1_ASM146515v1_genomic.fna
7828	Klebsiella pneumoniae	GCA_000714635.1_ASM71463v1_genomic.fna
7829	Mycobacterium tuberculosis	GCA_000270345.1_ASM27034v1_genomic.fna
7830	Mycobacterium tuberculosis	GCA_000277085.1_ASM27708v1_genomic.fna
7831	Mycobacterium tuberculosis	GCA_000277105.1_ASM27710v1_genomic.fna
7832	Mycobacterium tuberculosis	GCA_000389905.1_ASM38990v1_genomic.fna
7833	Mycobacterium tuberculosis	GCA_000389925.1_ASM38992v1_genomic.fna
7834	Mycobacterium tuberculosis	GCA_000954155.1_ASM95415v1_genomic.fna
7835	Mycobacterium tuberculosis	GCA_001544675.1_ASM154467v1_genomic.fna
7836	Mycobacterium tuberculosis	GCA_001544895.1_ASM154489v1_genomic.fna
7837	Mycobacterium tuberculosis	GCA_001544935.1_ASM154493v1_genomic.fna
7838	Mycobacterium tuberculosis	GCA_001544985.1_ASM154498v1_genomic.fna
7839	Mycobacterium tuberculosis	GCA_001545055.1_ASM154505v1_genomic.fna
7840	Salmonella enterica	GCA_000272795.2_ASM27279v2_genomic.fna
7841	Salmonella enterica	GCA_000444445.1_ASM44444v1_genomic.fna
7842	Salmonella enterica	GCA_000486525.2_ASM48652v2_genomic.fna
7843	Salmonella enterica	GCA_001278275.1_ASM127827v1_genomic.fna
7844	Salmonella enterica	GCA_001278295.1_ASM127829v1_genomic.fna
7845	Salmonella enterica	GCA_001302625.1_ASM130262v1_genomic.fna
7846	Salmonella enterica	GCA_002079245.1_ASM207924v1_genomic.fna
7847	Salmonella enterica	GCA_002208195.1_ASM220819v1_genomic.fna
7848	Salmonella enterica	GCA_002208965.1_ASM220896v1_genomic.fna
7849	Salmonella enterica	GCA_002209225.1_ASM220922v1_genomic.fna
7850	Staphylococcus aureus	GCA_000709475.1_ASM70947v1_genomic.fna
7851	Staphylococcus aureus	GCA_001641045.1_ASM164104v1_genomic.fna

7852	Staphylococcus aureus	GCA_001717665.1_ASM171766v1_genomic.fna
7853	Staphylococcus aureus	GCA_001717685.1_ASM171768v1_genomic.fna
7854	Staphylococcus aureus	GCA_001717705.1_ASM171770v1_genomic.fna
7855	Staphylococcus aureus	GCA_001717725.1_ASM171772v1_genomic.fna
7856	Staphylococcus aureus	GCA_001717975.1_ASM171797v1_genomic.fna
7857	Staphylococcus aureus	GCA_900155335.1_NZ15MR0322_genomic.fna
7858	Escherichia coli	GCA_000988425.1_ASM98842v1_genomic.fna
7859	Escherichia coli	GCA_000988445.1_ASM98844v1_genomic.fna
7860	Escherichia coli	GCA_001513655.1_ASM151365v1_genomic.fna
7861	Escherichia coli	GCA_002215155.1_ASM221515v1_genomic.fna
