# Supplementary material for: A New Genome-to-Genome Comparison Approach for Large-Scale Revisiting of Current Microbial Taxonomy
Source: Microorganisms. 2019 Jun 3;7(6):161. doi: 10.3390/microorganisms7060161 (PMC6616886; doi:10.3390/microorganisms7060161)
Supplement: Supplementary file 1 [file microorganisms-07-00161-s001.zip › supplementary_files/Supplemental_TableS2.pdf]

Table S2. The observed 1220 genus pairs of ambiguous classification.

genus A	genus B	minimum HCR of intra-genus	maximum HCR of cross-genus	Phylum A	Phylum B
Mesoplasma	Mycoplasma	0.025477347	0.393024522	Tenericutes	Tenericutes
Mycoplasma	Spiroplasma	0.025477347	0.292072093	Tenericutes	Tenericutes
Corynebacterium	Brevibacterium	0.125961995	0.999978422	Actinobacteria	Actinobacteria
Clostridium	Ruminiclostridium	0.062850588	0.394926701	Firmicutes	Firmicutes
Arsenophonus	Serratia	0.093374951	0.567689795	Proteobacteria	Proteobacteria
Mycoplasma	Ureaplasma	0.025477347	0.153290362	Tenericutes	Tenericutes
Alkaliphilus	Clostridium	0.062850588	0.37022779	Firmicutes	Firmicutes
Buchnera	Serratia	0.093374951	0.543884533	Proteobacteria	Proteobacteria
Chania	Serratia	0.093374951	0.539749427	Proteobacteria	Proteobacteria
Wigglesworthia	Serratia	0.093374951	0.536775217	Proteobacteria	Proteobacteria
Gibbsiella	Serratia	0.093374951	0.535694404	Proteobacteria	Proteobacteria
Serratia	Raoultella	0.093374951	0.474124623	Proteobacteria	Proteobacteria
Pluralibacter	Serratia	0.093374951	0.470894243	Proteobacteria	Proteobacteria
Erwinia	Serratia	0.093374951	0.470282266	Proteobacteria	Proteobacteria
Enterobacter	Serratia	0.093374951	0.47023974	Proteobacteria	Proteobacteria
Rahnella	Serratia	0.093374951	0.465615613	Proteobacteria	Proteobacteria
Cedecea	Serratia	0.093374951	0.463121787	Proteobacteria	Proteobacteria
Cronobacter	Serratia	0.093374951	0.461351074	Proteobacteria	Proteobacteria
Serratia	Klebsiella	0.093374951	0.458439613	Proteobacteria	Proteobacteria
Yersinia	Serratia	0.093374951	0.45825119	Proteobacteria	Proteobacteria
Pantoea	Serratia	0.093374951	0.458031964	Proteobacteria	Proteobacteria
Leclercia	Serratia	0.093374951	0.451964621	Proteobacteria	Proteobacteria
Lelliottia	Serratia	0.093374951	0.449856021	Proteobacteria	Proteobacteria
Serratia	Enterobacteriaceae	0.093374951	0.445376785	Proteobacteria	Proteobacteria
Desulfovibrio	Pseudodesulfovibrio	0.143678238	0.676764659	Proteobacteria	Proteobacteria
Serratia	Kosakonia	0.093374951	0.437942693	Proteobacteria	Proteobacteria
Kluyvera	Serratia	0.093374951	0.437425467	Proteobacteria	Proteobacteria
Plautia	Serratia	0.093374951	0.432232945	Proteobacteria	Proteobacteria
Mycoplasma	Maize	0.025477347	0.117101967	Tenericutes	Tenericutes
Shimwellia	Serratia	0.093374951	0.427930381	Proteobacteria	Proteobacteria

Hafnia	Serratia	0.093374951	0.42743639	Proteobacteria	Proteobacteria
Serratia	Blochmannia	0.093950708	0.428671932	Proteobacteria	Proteobacteria
Serratia	Dickeya	0.093374951	0.425491246	Proteobacteria	Proteobacteria
Geosporobacter	Clostridium	0.062850588	0.285266859	Firmicutes	Firmicutes
Citrobacter	Serratia	0.093374951	0.419559679	Proteobacteria	Proteobacteria
Burkholderia	Pseudomonas	0.175575691	0.787928009	Proteobacteria	Proteobacteria
Serratia	Pectobacterium	0.093374951	0.416512347	Proteobacteria	Proteobacteria
Serratia	Obesumbacterium	0.093374951	0.413693906	Proteobacteria	Proteobacteria
Edwardsiella	Serratia	0.093374951	0.413338404	Proteobacteria	Proteobacteria
Salmonella	Serratia	0.093374951	0.409131688	Proteobacteria	Proteobacteria
synthetic	Serratia	0.093374951	0.409107419	Proteobacteria	Proteobacteria
Escherichia	Serratia	0.093374951	0.407647176	Proteobacteria	Proteobacteria
Shigella	Serratia	0.093374951	0.398945239	Proteobacteria	Proteobacteria
Serratia	Brenneria	0.093374951	0.395474454	Proteobacteria	Proteobacteria
Mycoplasma	Aster	0.025477347	0.106899034	Tenericutes	Tenericutes
Sodalis	Serratia	0.093374951	0.390155916	Proteobacteria	Proteobacteria
Mycoplasma	Sneathia	0.025477347	0.106300298	Tenericutes	Fusobacteria
Mycoplasma	Lactobacillus	0.025477347	0.104827405	Tenericutes	Firmicutes
Baumannia	Serratia	0.093950708	0.383007697	Proteobacteria	Proteobacteria
Tatumella	Serratia	0.093374951	0.369375987	Proteobacteria	Proteobacteria
Mycoplasma	Streptobacillus	0.025477347	0.100348214	Tenericutes	Fusobacteria
Cutibacterium	Propionibacterium	0.255521499	0.999909386	Actinobacteria	Actinobacteria
Gottschalkia	Clostridium	0.062850588	0.244964684	Firmicutes	Firmicutes
Mycoplasma	Parvimonas	0.025477347	0.098476219	Tenericutes	Firmicutes
Roseburia	Eubacterium	0.078449324	0.302253966	Firmicutes	Firmicutes
Mycoplasma	Onion	0.025477347	0.098036715	Tenericutes	Tenericutes
Morganella	Serratia	0.093374951	0.355256354	Proteobacteria	Proteobacteria
Mycoplasma	Strawberry	0.025477347	0.096644537	Tenericutes	Tenericutes
Mycoplasma	Erysipelothrix	0.025477347	0.095434994	Tenericutes	Firmicutes
Mycoplasma	Melissococcus	0.025477347	0.09255834	Tenericutes	Firmicutes
Mycoplasma	Serratia	0.025477347	0.092527209	Tenericutes	Proteobacteria
Mycoplasma	Buchnera	0.025477347	0.092317533	Tenericutes	Proteobacteria
Clostridium	Ethanoligenens	0.062850588	0.226698757	Firmicutes	Firmicutes
Clostridioides	Clostridium	0.062850588	0.226177551	Firmicutes	Firmicutes

Pragia	Serratia	0.093374951	0.334996939	Proteobacteria	Proteobacteria
Clostridium	Thermoanaerobacter	0.062850588	0.222513278	Firmicutes	Firmicutes
Mycoplasma	Auricoccus	0.025477347	0.088526757	Tenericutes	Firmicutes
Vagococcus	Mycoplasma	0.025477347	0.087751112	Firmicutes	Tenericutes
Acetoanaerobium	Clostridium	0.062850588	0.213054995	Firmicutes	Firmicutes
Anaerostipes	Eubacterium	0.078449324	0.264337349	Firmicutes	Firmicutes
Xenorhabdus	Serratia	0.093374951	0.312062601	Proteobacteria	Proteobacteria
Mycoplasma	Fusobacterium	0.025477347	0.084570551	Tenericutes	Fusobacteria
Mycoplasma	Carnobacterium	0.025477347	0.084476001	Tenericutes	Firmicutes
Providencia	Serratia	0.093374951	0.309382588	Proteobacteria	Proteobacteria
Clostridium	Thermoanaerobacteriu	0.062850588	0.206437865	Firmicutes	Firmicutes
Photorhabdus	Serratia	0.093374951	0.301450623	Proteobacteria	Proteobacteria
Mycoplasma	Pediococcus	0.025477347	0.082076153	Tenericutes	Firmicutes
Mycoplasma	Staphylococcus	0.025477347	0.081956648	Tenericutes	Firmicutes
Mycoplasma	Halanaerobium	0.025477347	0.081759369	Tenericutes	Firmicutes
Lachnoclostridium	Clostridium	0.062850588	0.201076862	Firmicutes	Firmicutes
Halanaerobium	Clostridium	0.062850588	0.199315336	Firmicutes	Firmicutes
Mycoplasma	Wigglesworthia	0.025477347	0.080126783	Tenericutes	Proteobacteria
Aeromonas	Serratia	0.093374951	0.293073629	Proteobacteria	Proteobacteria
Proteus	Serratia	0.093374951	0.293027824	Proteobacteria	Proteobacteria
Plesiomonas	Serratia	0.093374951	0.29252767	Proteobacteria	Proteobacteria
Roseburia	Clostridium	0.062850588	0.195253275	Firmicutes	Firmicutes
Finegoldia	Mycoplasma	0.025477347	0.077824273	Firmicutes	Tenericutes
Blautia	Clostridium	0.062850588	0.191603505	Firmicutes	Firmicutes
Acholeplasma	Mycoplasma	0.025477347	0.077462386	Tenericutes	Tenericutes
Mycoplasma	Jeotgalibaca	0.025477347	0.077270615	Tenericutes	Firmicutes
Acetobacterium	Eubacterium	0.078449324	0.233426914	Firmicutes	Firmicutes
Tetragenococcus	Mycoplasma	0.025477347	0.075761655	Firmicutes	Tenericutes
Caldanaerobacter	Clostridium	0.062850588	0.186838324	Firmicutes	Firmicutes
Halobacteroides	Clostridium	0.062850588	0.185239875	Firmicutes	Firmicutes
Blattabacterium	Mycoplasma	0.025477347	0.074857236	Bacteroidetes	Tenericutes
Amphibacillus	Mycoplasma	0.025477347	0.074576734	Firmicutes	Tenericutes
Clostridium	Fusobacterium	0.062850588	0.18339051	Firmicutes	Fusobacteria
Zobellella	Serratia	0.093374951	0.27060128	Proteobacteria	Proteobacteria

Lactococcus	Mycoplasma	0.025477347	0.073451158	Firmicutes	Tenericutes
Tepidanaerobacter	Clostridium	0.062850588	0.180362159	Firmicutes	Firmicutes
Mycoplasma	Aerococcus	0.025477347	0.073020224	Tenericutes	Firmicutes
Mycoplasma	Dialister	0.025477347	0.07266113	Tenericutes	Firmicutes
Clostridium	Ruminococcaceae	0.062850588	0.178049583	Firmicutes	Firmicutes
Mycoplasma	Brochothrix	0.025477347	0.072092987	Tenericutes	Firmicutes
Mycoplasma	Clostridium	0.025477347	0.071650761	Tenericutes	Firmicutes
Leuconostoc	Mycoplasma	0.025477347	0.070530926	Firmicutes	Tenericutes
Acetohalobium	Clostridium	0.062850588	0.173931923	Firmicutes	Firmicutes
Mycoplasma	Macrococcus	0.025477347	0.070441636	Tenericutes	Firmicutes
Halobacteroides	Mycoplasma	0.025477347	0.070251101	Firmicutes	Tenericutes
Mycoplasma	Arsenophonus	0.025477347	0.069753878	Tenericutes	Proteobacteria
Streptococcus	Mycoplasma	0.025477347	0.069737266	Firmicutes	Tenericutes
Mycoplasma	Borreliella	0.025477347	0.069350994	Tenericutes	Spirochaetes
Blautia	Eubacterium	0.078449324	0.213542842	Firmicutes	Firmicutes
Rhodoluna	Actinobacteria	0.072679127	0.197279466	Actinobacteria	Actinobacteria
Herbinix	Clostridium	0.062850588	0.170353412	Firmicutes	Firmicutes
Mycoplasma	Borrelia	0.025477347	0.068924713	Tenericutes	Spirochaetes
Clostridium	Parvimonas	0.062850588	0.169809286	Firmicutes	Firmicutes
Bacillus	Parageobacillus	0.132343801	0.355950243	Firmicutes	Firmicutes
Mycoplasma	Megamonas	0.025477347	0.068194906	Tenericutes	Firmicutes
Mycoplasma	Listeria	0.025477347	0.068062839	Tenericutes	Firmicutes
Bacillus	Anoxybacillus	0.132343801	0.350887969	Firmicutes	Firmicutes
Chryseobacterium	Flavobacteriaceae	0.161380659	0.42647256	Bacteroidetes	Bacteroidetes
Peptoclostridium	Clostridium	0.062850588	0.165929701	Firmicutes	Firmicutes
Anaerostipes	Clostridium	0.062850588	0.165920574	Firmicutes	Firmicutes
Mycoplasma	Clostridioides	0.025477347	0.067143787	Tenericutes	Firmicutes
Pelosinus	Clostridium	0.062850588	0.165178975	Firmicutes	Firmicutes
Eubacterium	Clostridium	0.062850588	0.165078552	Firmicutes	Firmicutes
Mycoplasma	Enterococcus	0.025477347	0.066882238	Tenericutes	Firmicutes
Mycoplasma	Anaerococcus	0.025477347	0.066503232	Tenericutes	Firmicutes
Serratia	Pseudomonas	0.093374951	0.242770525	Proteobacteria	Proteobacteria
Finegoldia	Clostridium	0.062850588	0.163338893	Firmicutes	Firmicutes
Bacillus	Aeribacillus	0.132343801	0.3437568	Firmicutes	Firmicutes

Lachnoclostridium	Eubacterium	0.078449324	0.201144273	Firmicutes	Firmicutes
Mycoplasma	Thermoanaerobacter	0.025477347	0.065038352	Tenericutes	Firmicutes
Histophilus	Haemophilus	0.356592956	0.908143774	Proteobacteria	Proteobacteria
Mycoplasma	Baumannia	0.025477347	0.064260727	Tenericutes	Proteobacteria
Herbinix	Eubacterium	0.078449324	0.197535648	Firmicutes	Firmicutes
Aurantimicrobium	Actinobacteria	0.072679127	0.182326373	Actinobacteria	Actinobacteria
Paraburkholderia	Pseudomonas	0.175575691	0.439935146	Proteobacteria	Proteobacteria
Butyrivibrio	Eubacterium	0.078449324	0.19575761	Firmicutes	Firmicutes
Caldicellulosiruptor	Clostridium	0.062850588	0.156451071	Firmicutes	Firmicutes
Mycoplasma	Filifactor	0.025477347	0.063345272	Tenericutes	Firmicutes
Anaerotignum	Clostridium	0.062850588	0.155193538	Firmicutes	Firmicutes
Mycoplasma	Leptotrichia	0.025477347	0.062684426	Tenericutes	Fusobacteria
Thermotoga	Pseudothermotoga	0.268941602	0.661348822	Thermotogae	Thermotogae
Serratia	Vibrio	0.093374951	0.229546008	Proteobacteria	Proteobacteria
Azotobacter	Pseudomonas	0.175575691	0.431245418	Proteobacteria	Proteobacteria
Cupriavidus	Ralstonia	0.330578046	0.811448755	Proteobacteria	Proteobacteria
Tolumonas	Serratia	0.093374951	0.22913193	Proteobacteria	Proteobacteria
Ilyobacter	Clostridium	0.062850588	0.154172699	Fusobacteria	Firmicutes
Thermosipho	Mycoplasma	0.025477347	0.06207076	Thermotogae	Tenericutes
Vagococcus	Clostridium	0.062850588	0.151373291	Firmicutes	Firmicutes
Ilyobacter	Mycoplasma	0.025477347	0.061169499	Fusobacteria	Tenericutes
Chlorobium	Chlorobaculum	0.269516596	0.646959688	Bacteroidetes	Bacteroidetes
Mycoplasma	Anoxybacillus	0.025477347	0.061146577	Tenericutes	Firmicutes
Clostridium	Filifactor	0.062850588	0.150637725	Firmicutes	Firmicutes
Haemophilus	Serratia	0.093374951	0.223295566	Proteobacteria	Proteobacteria
Acetoanaerobium	Mycoplasma	0.025477347	0.060886317	Firmicutes	Tenericutes
Chlamydia	Chlamydophila	0.41910917	0.999983732	Chlamydiae	Chlamydiae
Halothermothrix	Clostridium	0.062850588	0.149502543	Firmicutes	Firmicutes
Clostridium	Natranaerobius	0.062850588	0.149472605	Firmicutes	Firmicutes
Megamonas	Clostridium	0.062850588	0.149396922	Firmicutes	Firmicutes
Grimontia	Serratia	0.093374951	0.221841296	Proteobacteria	Proteobacteria
Bacillus	Geobacillus	0.132343801	0.314041514	Firmicutes	Firmicutes
Carnobacterium	Clostridium	0.062850588	0.14876032	Firmicutes	Firmicutes
Marinitoga	Mycoplasma	0.025477347	0.060104469	Thermotogae	Tenericutes

Mycoplasma	Blochmannia	0.025477347	0.059927766	Tenericutes	Proteobacteria
Mahella	Clostridium	0.062850588	0.147452815	Firmicutes	Firmicutes
Mycoplasma	Rummeliibacillus	0.025477347	0.059569882	Tenericutes	Firmicutes
Anaerococcus	Clostridium	0.062850588	0.145782905	Firmicutes	Firmicutes
Butyrivibrio	Clostridium	0.062850588	0.14554169	Firmicutes	Firmicutes
Mycoplasma	Oenococcus	0.025477347	0.058886888	Tenericutes	Firmicutes
Desulfotomaculum	Clostridium	0.062850588	0.14433377	Firmicutes	Firmicutes
Mycoplasma	Desulfurobacterium	0.025477347	0.058463639	Tenericutes	Aquificae
Mycoplasma	Arcobacter	0.025477347	0.058455972	Tenericutes	Proteobacteria
Ruminococcus	Clostridium	0.062850588	0.144105956	Firmicutes	Firmicutes
Mesoplasma	Spiroplasma	0.159751234	0.365244672	Tenericutes	Tenericutes
Mycoplasma	Campylobacter	0.025477347	0.058155823	Tenericutes	Proteobacteria
Defluviitoga	Mycoplasma	0.025477347	0.058091186	Thermotogae	Tenericutes
Mycoplasma	Thermoanaerobacteriu	0.025477347	0.057785089	Tenericutes	Firmicutes
Clostridium	Dehalobacterium	0.062850588	0.141637437	Firmicutes	Firmicutes
Mycoplasma	Thermodesulfobacteriu	0.025477347	0.057119952	Tenericutes	Thermodesulfobacteria
Aliivibrio	Vibrio	0.271195069	0.605076732	Proteobacteria	Proteobacteria
Clostridium	Flavonifractor	0.062850588	0.139893291	Firmicutes	Firmicutes
Mycoplasma	Oceanobacillus	0.025477347	0.056658086	Tenericutes	Firmicutes
Desulfosporosinus	Clostridium	0.062850588	0.139735072	Firmicutes	Firmicutes
Planococcus	Mycoplasma	0.025477347	0.056577585	Firmicutes	Tenericutes
Allofrancisella	Mycoplasma	0.025477347	0.056487228	Proteobacteria	Tenericutes
Bacillus	Clostridium	0.062850588	0.139302737	Firmicutes	Firmicutes
Gottschalkia	Mycoplasma	0.025477347	0.056436897	Firmicutes	Tenericutes
Acetobacterium	Clostridium	0.062850588	0.139206894	Firmicutes	Firmicutes
Ferrimonas	Serratia	0.093950708	0.20804697	Proteobacteria	Proteobacteria
Intestinimonas	Clostridium	0.062850588	0.138988992	Firmicutes	Firmicutes
Sneathia	Clostridium	0.062850588	0.138430009	Fusobacteria	Firmicutes
Deferribacter	Mycoplasma	0.025477347	0.055736062	Deferribacteres	Tenericutes
Bacillus	Mycoplasma	0.025477347	0.055643465	Firmicutes	Tenericutes
Leptotrichia	Clostridium	0.062850588	0.137134366	Fusobacteria	Firmicutes
Mycoplasma	Dictyoglomus	0.025477347	0.055055089	Tenericutes	Dictyoglomi
Mycoplasma	Lawsonia	0.025477347	0.054930781	Tenericutes	Proteobacteria
Fictibacillus	Bacillus	0.132343801	0.285294649	Firmicutes	Firmicutes

Amphibacillus	Clostridium	0.062850588	0.135464601	Firmicutes	Firmicutes
Riemerella	Flavobacteriaceae	0.161380659	0.347239911	Bacteroidetes	Bacteroidetes
Streptobacillus	Clostridium	0.068824019	0.147892936	Fusobacteria	Firmicutes
Clostridium	Thermincola	0.062850588	0.134938911	Firmicutes	Firmicutes
Burkholderiales	Laribacter	0.092214558	0.197974696	Proteobacteria	Proteobacteria
Staphylococcus	Clostridium	0.062850588	0.134274401	Firmicutes	Firmicutes
Clostridium	Lactobacillus	0.062850588	0.133962926	Firmicutes	Firmicutes
Oceanobacillus	Clostridium	0.062850588	0.13366524	Firmicutes	Firmicutes
Mycoplasma	Lysinibacillus	0.025477347	0.053848143	Tenericutes	Firmicutes
Actinobacillus	Serratia	0.093374951	0.196633434	Proteobacteria	Proteobacteria
Francisella	Mycoplasma	0.025477347	0.053472041	Proteobacteria	Tenericutes
Kocuria	Brevibacterium	0.125961995	0.264062871	Actinobacteria	Actinobacteria
Clostridium	Melissococcus	0.062850588	0.131673615	Firmicutes	Firmicutes
Paraphotobacterium	Mycoplasma	0.025477347	0.053272222	Proteobacteria	Tenericutes
Mycoplasma	Prochlorococcus	0.025477347	0.052970717	Tenericutes	Cyanobacteria
Listeria	Clostridium	0.062850588	0.130519333	Firmicutes	Firmicutes
Thermosediminibacter	Clostridium	0.062850588	0.130431617	Firmicutes	Firmicutes
Photobacterium	Serratia	0.093374951	0.193621755	Proteobacteria	Proteobacteria
Desulfitobacterium	Clostridium	0.062850588	0.130095828	Firmicutes	Firmicutes
Sulfurihydrogenibium	Mycoplasma	0.025477347	0.052735121	Aquificae	Tenericutes
Erwinia	Sodalis	0.189313104	0.391743909	Proteobacteria	Proteobacteria
Oscillibacter	Clostridium	0.062850588	0.129913709	Firmicutes	Firmicutes
Serratia	Aggregatibacter	0.093374951	0.192563213	Proteobacteria	Proteobacteria
Acidothermus	Actinobacteria	0.072679127	0.149688992	Actinobacteria	Actinobacteria
Coxiella	Mycoplasma	0.025477347	0.052446592	Proteobacteria	Tenericutes
Pantoea	Sodalis	0.189313104	0.388520644	Proteobacteria	Proteobacteria
Burkholderiales	Rugosibacter	0.092214558	0.18924114	Proteobacteria	Proteobacteria
Kitasatospora	Streptomyces	0.354202047	0.726292879	Actinobacteria	Actinobacteria
Paraglaciecola	Pseudoalteromonas	0.161098934	0.330079459	Proteobacteria	Proteobacteria
Mycoplasma	Virgibacillus	0.025477347	0.052191217	Tenericutes	Firmicutes
Anoxybacillus	Clostridium	0.062850588	0.128620836	Firmicutes	Firmicutes
Mycoplasma	Ichthyobacterium	0.025477347	0.051916627	Tenericutes	Bacteroidetes
Mycoplasma	Weissella	0.025477347	0.051846601	Tenericutes	Firmicutes
Oceanisphaera	Serratia	0.093937707	0.191116347	Proteobacteria	Proteobacteria

Mycoplasma	Ezakiella	0.025477347	0.0516976	Tenericutes	Firmicutes
Dialister	Clostridium	0.062850588	0.127419873	Firmicutes	Firmicutes
Mycoplasma	Rickettsia	0.025477347	0.051540251	Tenericutes	Proteobacteria
Mycoplasma	Brachyspira	0.025477347	0.051259824	Tenericutes	Spirochaetes
Cronobacter	Sodalis	0.189313104	0.380317179	Proteobacteria	Proteobacteria
Mycoplasma	Solibacillus	0.025477347	0.051134801	Tenericutes	Firmicutes
Thermococcus	Pyrococcus	0.351634878	0.705169843	Euryarchaeota	Euryarchaeota
Clostridium	Carboxydothermus	0.062850588	0.125900226	Firmicutes	Firmicutes
Brochothrix	Clostridium	0.062850588	0.12587094	Firmicutes	Firmicutes
Bacillus	Virgibacillus	0.132343801	0.264910237	Firmicutes	Firmicutes
Veillonella	Mycoplasma	0.025477347	0.050991793	Firmicutes	Tenericutes
Bacillus	Oceanobacillus	0.132343801	0.264694914	Firmicutes	Firmicutes
Jeotgalibaca	Clostridium	0.062850588	0.125627878	Firmicutes	Firmicutes
Pediococcus	Lactobacillus	0.158222465	0.316065418	Firmicutes	Firmicutes
Desulfurella	Mycoplasma	0.025477347	0.050815793	Proteobacteria	Tenericutes
Bacillus	Terribacillus	0.132343801	0.263846126	Firmicutes	Firmicutes
Sodalis	Enterobacter	0.189313104	0.376766613	Proteobacteria	Proteobacteria
Desulfovibrio	Desulfomicrobium	0.143678238	0.285622396	Proteobacteria	Proteobacteria
Mycoplasma	Caldicellulosiruptor	0.025477347	0.050594935	Tenericutes	Firmicutes
Paraburkholderia	Burkholderia	0.232579852	0.461546577	Proteobacteria	Proteobacteria
Mycoplasma	Sporosarcina	0.025477347	0.050537485	Tenericutes	Firmicutes
Bacillus	Lysinibacillus	0.132343801	0.262471597	Firmicutes	Firmicutes
Dictyoglomus	Clostridium	0.062850588	0.124458355	Dictyoglomi	Firmicutes
Pasteurella	Serratia	0.093374951	0.184895938	Proteobacteria	Proteobacteria
Virgibacillus	Clostridium	0.062850588	0.124078811	Firmicutes	Firmicutes
Sodalis	Plautia	0.189313104	0.372080498	Proteobacteria	Proteobacteria
Deferribacter	Clostridium	0.062850588	0.123522279	Deferribacteres	Firmicutes
Sodalis	Gibbsiella	0.189313104	0.371938132	Proteobacteria	Proteobacteria
Mycoplasma	Acetohalobium	0.025477347	0.05003134	Tenericutes	Firmicutes
Bacillus	Planococcus	0.132343801	0.259807239	Firmicutes	Firmicutes
Mycoplasma	Herbinix	0.025477347	0.049762002	Tenericutes	Firmicutes
Bacillus	Sporosarcina	0.132343801	0.257738812	Firmicutes	Firmicutes
Rahnella	Sodalis	0.189313104	0.368639601	Proteobacteria	Proteobacteria
Mycoplasma	Eubacterium	0.025477347	0.049577839	Tenericutes	Firmicutes

Brevibacterium	Micrococcus	0.125961995	0.244936426	Actinobacteria	Actinobacteria
Pandoraea	Pseudomonas	0.175575691	0.340789917	Proteobacteria	Proteobacteria
Lelliottia	Sodalis	0.189313104	0.366222921	Proteobacteria	Proteobacteria
Sinomonas	Brevibacterium	0.125961995	0.24359065	Actinobacteria	Actinobacteria
Sodalis	Klebsiella	0.189313104	0.366070485	Proteobacteria	Proteobacteria
Mycoplasma	Alkaliphilus	0.025477347	0.049263315	Tenericutes	Firmicutes
Tetragenococcus	Clostridium	0.062850588	0.121426359	Firmicutes	Firmicutes
Shewanella	Serratia	0.093374951	0.180303738	Proteobacteria	Proteobacteria
Mycoplasma	Mageeibacillus	0.025477347	0.049067051	Tenericutes	Firmicutes
Sodalis	Cedecea	0.189313104	0.364244919	Proteobacteria	Proteobacteria
Sodalis	Kosakonia	0.189313104	0.36323503	Proteobacteria	Proteobacteria
Cupriavidus	Pseudomonas	0.175575691	0.336690155	Proteobacteria	Proteobacteria
Mycoplasma	Caldanaerobacter	0.025477347	0.048829488	Tenericutes	Firmicutes
Gallibacterium	Serratia	0.093374951	0.178960809	Proteobacteria	Proteobacteria
Sodalis	Leclercia	0.189313104	0.362455977	Proteobacteria	Proteobacteria
Bacillus	Rummeliibacillus	0.132343801	0.253165639	Firmicutes	Firmicutes
Fictibacillus	Clostridium	0.062850588	0.120200552	Firmicutes	Firmicutes
Alteromonas	Pseudoalteromonas	0.161098934	0.308016317	Proteobacteria	Proteobacteria
Sodalis	Raoultella	0.189313104	0.361868755	Proteobacteria	Proteobacteria
Faecalibacterium	Clostridium	0.062850588	0.120080944	Firmicutes	Firmicutes
Caldisericum	Mycoplasma	0.025477347	0.04863718	Caldiserica	Tenericutes
Serratia	Mannheimia	0.093374951	0.177593219	Proteobacteria	Proteobacteria
Sodalis	Dickeya	0.189313104	0.358904543	Proteobacteria	Proteobacteria
Marinitoga	Clostridium	0.062850588	0.119062607	Thermotogae	Firmicutes
Planococcus	Clostridium	0.062850588	0.119044108	Firmicutes	Firmicutes
Clostridium	Rummeliibacillus	0.062850588	0.118930094	Firmicutes	Firmicutes
Dehalobacter	Clostridium	0.062850588	0.118850955	Firmicutes	Firmicutes
Sodalis	Citrobacter	0.189313104	0.357911196	Proteobacteria	Proteobacteria
Auricoccus	Clostridium	0.062850588	0.118685439	Firmicutes	Firmicutes
Mycoplasma	Halothermothrix	0.025477347	0.048035324	Tenericutes	Firmicutes
Pluralibacter	Sodalis	0.189313104	0.356600532	Proteobacteria	Proteobacteria
Bacillus	Jeotgalibacillus	0.132343801	0.248727168	Firmicutes	Firmicutes
Sodalis	Enterobacteriaceae	0.189313104	0.355534119	Proteobacteria	Proteobacteria
Syntrophobotulus	Clostridium	0.062850588	0.117649647	Firmicutes	Firmicutes

Flavobacteriaceae	Elizabethkingia	0.161380659	0.302060317	Bacteroidetes	Bacteroidetes
Mycoplasma	Haemophilus	0.025477347	0.047578548	Tenericutes	Proteobacteria
Anaerostipes	Mycoplasma	0.025477347	0.047543996	Firmicutes	Tenericutes
Macrococcus	Clostridium	0.062850588	0.117186633	Firmicutes	Firmicutes
Defluviitoga	Clostridium	0.062850588	0.11712072	Thermotogae	Firmicutes
Veillonella	Clostridium	0.062850588	0.117014562	Firmicutes	Firmicutes
Escherichia	Sodalis	0.189313104	0.35202389	Proteobacteria	Proteobacteria
Salmonella	Sodalis	0.189313104	0.351809762	Proteobacteria	Proteobacteria
Bacillus	Solibacillus	0.132343801	0.245427912	Firmicutes	Firmicutes
Sodalis	Brenneria	0.189313104	0.350509193	Proteobacteria	Proteobacteria
Diaphorobacter	Acidovorax	0.464019965	0.858878301	Proteobacteria	Proteobacteria
Shimwellia	Sodalis	0.189313104	0.349803394	Proteobacteria	Proteobacteria
Mycoplasma	Fervidobacterium	0.025477347	0.04706694	Tenericutes	Thermotogae
Sodalis	Shigella	0.189313104	0.347718415	Proteobacteria	Proteobacteria
Enterococcus	Clostridium	0.062850588	0.115116126	Firmicutes	Firmicutes
Exiguobacterium	Mycoplasma	0.025477347	0.046663167	Firmicutes	Tenericutes
Sodalis	synthetic	0.189313104	0.346605252	Proteobacteria	Proteobacteria
Glutamicibacter	Brevibacterium	0.125961995	0.230480835	Actinobacteria	Actinobacteria
Neomicrococcus	Actinobacteria	0.072679127	0.132734379	Actinobacteria	Actinobacteria
Sodalis	Pectobacterium	0.189313104	0.345681002	Proteobacteria	Proteobacteria
Yersinia	Sodalis	0.189313104	0.345591518	Proteobacteria	Proteobacteria
Anaerotignum	Eubacterium	0.078449324	0.143128624	Firmicutes	Firmicutes
Gilliamella	Serratia	0.093374951	0.170319816	Proteobacteria	Proteobacteria
Delftia	Serratia	0.093374951	0.170050344	Proteobacteria	Proteobacteria
Kluyvera	Sodalis	0.189313104	0.343696081	Proteobacteria	Proteobacteria
Bacillus	Halobacillus	0.132343801	0.239834925	Firmicutes	Firmicutes
Acholeplasma	Clostridium	0.062850588	0.113849827	Tenericutes	Firmicutes
Janibacter	Brevibacterium	0.125961995	0.228159604	Actinobacteria	Actinobacteria
Mycoplasma	Jeotgalibacillus	0.025477347	0.04603116	Tenericutes	Firmicutes
Cedecea	Klebsiella	0.459467233	0.829742343	Proteobacteria	Proteobacteria
Thermodesulfovibrio	Clostridium	0.062850588	0.113341622	Nitrospirae	Firmicutes
Sodalis	Edwardsiella	0.189313104	0.341194298	Proteobacteria	Proteobacteria
Mycoplasma	Petrotoga	0.025477347	0.045857376	Tenericutes	Thermotogae
Mycoplasma	Tenacibaculum	0.025477347	0.045730159	Tenericutes	Bacteroidetes

Aeribacillus	Clostridium	0.062850588	0.112598785	Firmicutes	Firmicutes
Lysinibacillus	Clostridium	0.062850588	0.111909051	Firmicutes	Firmicutes
Aliivibrio	Serratia	0.093374951	0.166153859	Proteobacteria	Proteobacteria
Pseudarthrobacter	Brevibacterium	0.125961995	0.223933403	Actinobacteria	Actinobacteria
Brevibacterium	Isoptericola	0.125961995	0.223566468	Actinobacteria	Actinobacteria
Clostridium	Sebaldella	0.068824019	0.12215169	Firmicutes	Fusobacteria
Jonesia	Actinobacteria	0.072679127	0.128876572	Actinobacteria	Actinobacteria
Mycoplasma	Nautilia	0.025477347	0.045131723	Tenericutes	Proteobacteria
Brachybacterium	Brevibacterium	0.125961995	0.222974104	Actinobacteria	Actinobacteria
Mycoplasma	Thermodesulfovibrio	0.025477347	0.044913956	Tenericutes	Nitrospirae
Eubacterium	Filifactor	0.078449324	0.138242455	Firmicutes	Firmicutes
Aeribacillus	Mycoplasma	0.025477347	0.044853374	Firmicutes	Tenericutes
Ezakiella	Clostridium	0.062850588	0.110563806	Firmicutes	Firmicutes
Streptococcus	Clostridium	0.062850588	0.110535028	Firmicutes	Firmicutes
Frischella	Serratia	0.093374951	0.163834129	Proteobacteria	Proteobacteria
Bibersteinia	Serratia	0.093374951	0.163708532	Proteobacteria	Proteobacteria
Lactococcus	Clostridium	0.062850588	0.110029702	Firmicutes	Firmicutes
Herbaspirillum	Pseudomonas	0.175575691	0.306938684	Proteobacteria	Proteobacteria
Fictibacillus	Mycoplasma	0.025477347	0.0444956	Firmicutes	Tenericutes
Ralstonia	Pseudomonas	0.175575691	0.306591152	Proteobacteria	Proteobacteria
Microbacterium	Brevibacterium	0.125961995	0.219931789	Actinobacteria	Actinobacteria
Mycoplasma	Cardinium	0.025477347	0.044407386	Tenericutes	Bacteroidetes
Terribacillus	Mycoplasma	0.025477347	0.044377413	Firmicutes	Tenericutes
Thermosipho	Clostridium	0.068824019	0.119607561	Thermotogae	Firmicutes
Rhizorhabdus	Sphingomonas	0.292558438	0.508278627	Proteobacteria	Proteobacteria
Brevibacillus	Clostridium	0.062850588	0.109073872	Firmicutes	Firmicutes
Chlorobium	Pelodictyon	0.269516596	0.467435234	Bacteroidetes	Bacteroidetes
Lawsonella	Actinobacteria	0.072679127	0.125905818	Actinobacteria	Actinobacteria
Mycoplasma	Histophilus	0.025477347	0.044065437	Tenericutes	Proteobacteria
Sodalis	Hafnia	0.189313104	0.327381214	Proteobacteria	Proteobacteria
Weissella	Lactobacillus	0.158222465	0.273555815	Firmicutes	Firmicutes
Jeotgalibacillus	Clostridium	0.062850588	0.107870687	Firmicutes	Firmicutes
Mycoplasma	Taylorella	0.025477347	0.043629096	Tenericutes	Proteobacteria
Mycoplasma	Natranaerobius	0.025477347	0.043624119	Tenericutes	Firmicutes

Desulfurobacterium	Clostridium	0.062850588	0.10737445	Aquificae	Firmicutes
Microterricola	Brevibacterium	0.125961995	0.215173259	Actinobacteria	Actinobacteria
Solibacillus	Clostridium	0.062850588	0.107313166	Firmicutes	Firmicutes
Pasteurella	Mycoplasma	0.025477347	0.043492105	Proteobacteria	Tenericutes
Thermobacillus	Paenibacillus	0.161111346	0.274919624	Firmicutes	Firmicutes
Microcella	Actinobacteria	0.072679127	0.123782533	Actinobacteria	Actinobacteria
Clostridium	Parageobacillus	0.062850588	0.106795798	Firmicutes	Firmicutes
Lachnoclostridium	Herbinix	0.132857063	0.225564058	Firmicutes	Firmicutes
Mycoplasma	Parageobacillus	0.025477347	0.043249272	Tenericutes	Firmicutes
Dermacoccus	Actinobacteria	0.072679127	0.122987209	Actinobacteria	Actinobacteria
Desulfurella	Clostridium	0.062850588	0.106319659	Proteobacteria	Firmicutes
Clostridium	Thermodesulfobacteriu	0.063423572	0.107214395	Firmicutes	Thermodesulfobacteria
Chania	Sodalis	0.189313104	0.319997694	Proteobacteria	Proteobacteria
Sodalis	Obesumbacterium	0.189313104	0.31978679	Proteobacteria	Proteobacteria
Actinobacteria	Rathayibacter	0.072679127	0.122730081	Actinobacteria	Actinobacteria
Frischella	Mycoplasma	0.025477347	0.043002703	Proteobacteria	Tenericutes
Sodalis	Tatumella	0.189313104	0.319177657	Proteobacteria	Proteobacteria
Chlorobium	Prosthecochloris	0.269516596	0.454248031	Bacteroidetes	Bacteroidetes
Lachnoclostridium	Roseburia	0.132857063	0.223897095	Firmicutes	Firmicutes
Faecalibacterium	Eubacterium	0.078449324	0.132059753	Firmicutes	Firmicutes
Terribacillus	Clostridium	0.062850588	0.105744749	Firmicutes	Firmicutes
Mycoplasma	Bartonella	0.025477347	0.042831883	Tenericutes	Proteobacteria
Blautia	Mycoplasma	0.025477347	0.042831608	Firmicutes	Tenericutes
Collimonas	Pseudomonas	0.175575691	0.295121963	Proteobacteria	Proteobacteria
Gilliamella	Mycoplasma	0.025477347	0.042764658	Proteobacteria	Tenericutes
Blautia	Lachnoclostridium	0.132857063	0.222893437	Firmicutes	Firmicutes
Neomicrococcus	Brevibacterium	0.125961995	0.211316674	Actinobacteria	Actinobacteria
Bacillus	Lentibacillus	0.132343801	0.221997209	Firmicutes	Firmicutes
Erysipelothrix	Clostridium	0.062850588	0.105401675	Firmicutes	Firmicutes
Mycoplasma	Thermodesulfobium	0.025477347	0.042673509	Tenericutes	Firmicutes
Clostridium	Pediococcus	0.062850588	0.105200105	Firmicutes	Firmicutes
Sporosarcina	Clostridium	0.062850588	0.105194804	Firmicutes	Firmicutes
Renibacterium	Actinobacteria	0.072679127	0.121447344	Actinobacteria	Actinobacteria
Bacillus	Salimicrobium	0.132343801	0.221119042	Firmicutes	Firmicutes

Coxiella	Coxiella-like	0.263574886	0.439951348	Proteobacteria	Proteobacteria
Desulfohalobium	Desulfovibrio	0.143678238	0.239814788	Proteobacteria	Proteobacteria
Mycoplasma	Liberibacter	0.025477347	0.04240628	Tenericutes	Proteobacteria
Eubacterium	Parvimonas	0.078449324	0.130353622	Firmicutes	Firmicutes
Pseudomonas	Variovorax	0.175575691	0.291723605	Proteobacteria	Proteobacteria
Dermacoccus	Brevibacterium	0.125961995	0.209091219	Actinobacteria	Actinobacteria
Rickettsiales	Mycoplasma	0.025477347	0.042277415	Proteobacteria	Tenericutes
Eubacterium	Ruminococcus	0.078449324	0.129980695	Firmicutes	Firmicutes
Vagococcus	Lactobacillus	0.158222465	0.262084489	Firmicutes	Firmicutes
Dermatophilus	Actinobacteria	0.072679127	0.120335771	Actinobacteria	Actinobacteria
Corynebacterium	Actinobacteria	0.072679127	0.120127625	Actinobacteria	Actinobacteria
Leifsonia	Brevibacterium	0.125961995	0.20770773	Actinobacteria	Actinobacteria
Janibacter	Actinobacteria	0.072679127	0.119829801	Actinobacteria	Actinobacteria
Clavibacter	Brevibacterium	0.125961995	0.207589607	Actinobacteria	Actinobacteria
Actinobacteria	Cutibacterium	0.072679127	0.119757454	Actinobacteria	Actinobacteria
Sulfurihydrogenibium	Clostridium	0.062850588	0.103514614	Aquificae	Firmicutes
Carnobacterium	Lactobacillus	0.158222465	0.26035462	Firmicutes	Firmicutes
Mycoplasma	Tepidanaerobacter	0.025477347	0.041881649	Tenericutes	Firmicutes
Lactobacillus	Melissococcus	0.158222465	0.260068326	Firmicutes	Firmicutes
Mycoplasma	Flavobacterium	0.025477347	0.041814122	Tenericutes	Bacteroidetes
Glaciecola	Pseudoalteromonas	0.161098934	0.263984039	Proteobacteria	Proteobacteria
Heliobacterium	Clostridium	0.062850588	0.102968879	Firmicutes	Firmicutes
Aerococcus	Clostridium	0.062850588	0.102826216	Firmicutes	Firmicutes
Mycoplasma	Hippea	0.025477347	0.041647728	Tenericutes	Proteobacteria
Agromyces	Brevibacterium	0.125961995	0.205841682	Actinobacteria	Actinobacteria
Leifsonia	Actinobacteria	0.072679127	0.118714204	Actinobacteria	Actinobacteria
Syntrophomonas	Clostridium	0.062850588	0.102611844	Firmicutes	Firmicutes
Gottschalkia	Eubacterium	0.078449324	0.12796072	Firmicutes	Firmicutes
Aneurinibacillus	Clostridium	0.062850588	0.102461527	Firmicutes	Firmicutes
Phoenicibacter	Mycoplasma	0.025477347	0.041431374	Actinobacteria	Tenericutes
Arthrobacter	Brevibacterium	0.125961995	0.204769382	Actinobacteria	Actinobacteria
Halobacillus	Clostridium	0.062850588	0.101969603	Firmicutes	Firmicutes
Lacimicrobium	Pseudoalteromonas	0.164337017	0.266546796	Proteobacteria	Proteobacteria
Bacillus	Staphylococcus	0.132343801	0.214450372	Firmicutes	Firmicutes

Clostridium	Paenibacillus	0.062850588	0.101839923	Firmicutes	Firmicutes
Comamonas	Delftia	0.306827503	0.49709349	Proteobacteria	Proteobacteria
Acetoanaerobium	Eubacterium	0.078449324	0.127002288	Firmicutes	Firmicutes
Actinobacteria	Propionibacterium	0.072679127	0.11732634	Actinobacteria	Actinobacteria
Brevibacillus	Bacillus	0.132343801	0.213231342	Firmicutes	Firmicutes
Brevibacterium	Cryobacterium	0.125961995	0.202790682	Actinobacteria	Actinobacteria
Histophilus	Serratia	0.093374951	0.15028029	Proteobacteria	Proteobacteria
Dichelobacter	Mycoplasma	0.025477347	0.040949236	Proteobacteria	Tenericutes
Paenarthrobacter	Brevibacterium	0.125961995	0.202417632	Actinobacteria	Actinobacteria
Clostridioides	Eubacterium	0.078449324	0.125939012	Firmicutes	Firmicutes
Bacillus	Amphibacillus	0.132343801	0.2124237	Firmicutes	Firmicutes
Kytococcus	Brevibacterium	0.125961995	0.201968538	Actinobacteria	Actinobacteria
Mycoplasma	Halobacillus	0.025477347	0.040817048	Tenericutes	Firmicutes
Eubacterium	Thermoanaerobacteriu	0.078449324	0.125203704	Firmicutes	Firmicutes
Halomonas	Pseudomonas	0.175575691	0.279941239	Proteobacteria	Proteobacteria
Lutibacter	Flavobacteriaceae	0.161380659	0.257093349	Bacteroidetes	Bacteroidetes
Paraphotobacterium	Serratia	0.093374951	0.148444626	Proteobacteria	Proteobacteria
Eubacterium	Thermoanaerobacter	0.078449324	0.124500272	Firmicutes	Firmicutes
Bacillus	Paenibacillus	0.132343801	0.209984491	Firmicutes	Firmicutes
Tetragenococcus	Lactobacillus	0.158222465	0.250900318	Firmicutes	Firmicutes
Xylanimonas	Brevibacterium	0.125961995	0.199353453	Actinobacteria	Actinobacteria
Curtobacterium	Brevibacterium	0.125961995	0.199121515	Actinobacteria	Actinobacteria
Thermobispora	Actinobacteria	0.072679127	0.114885073	Actinobacteria	Actinobacteria
Alkaliphilus	Eubacterium	0.078449324	0.123846531	Firmicutes	Firmicutes
Bacillus	Exiguobacterium	0.132343801	0.208709461	Firmicutes	Firmicutes
Thermobifida	Actinobacteria	0.072679127	0.114571366	Actinobacteria	Actinobacteria
Halotalea	Pseudomonas	0.175575691	0.276582709	Proteobacteria	Proteobacteria
Actinobacteria	Isoptericola	0.072679127	0.114369111	Actinobacteria	Actinobacteria
Mycoplasma	Lutibacter	0.025477347	0.039912982	Tenericutes	Bacteroidetes
Peptoclostridium	Eubacterium	0.078449324	0.12286954	Firmicutes	Firmicutes
Intrasporangium	Brevibacterium	0.125961995	0.197215641	Actinobacteria	Actinobacteria
Mycoplasma	Lentibacillus	0.025477347	0.039870249	Tenericutes	Firmicutes
Massilia	Pseudomonas	0.175575691	0.274710086	Proteobacteria	Proteobacteria
Cellulosimicrobium	Brevibacterium	0.125961995	0.197064684	Actinobacteria	Actinobacteria

Sanguibacter	Brevibacterium	0.125961995	0.196765709	Actinobacteria	Actinobacteria
Clostridium	Megasphaera	0.062850588	0.09817325	Firmicutes	Firmicutes
Bacillus	Aneurinibacillus	0.132343801	0.206446506	Firmicutes	Firmicutes
Aeromicrobium	Actinobacteria	0.072679127	0.113317464	Actinobacteria	Actinobacteria
Mycoplasma	Mannheimia	0.025477347	0.039716057	Tenericutes	Proteobacteria
Arcanobacterium	Actinobacteria	0.072679127	0.113204361	Actinobacteria	Actinobacteria
Intrasporangium	Actinobacteria	0.072679127	0.112903283	Actinobacteria	Actinobacteria
Moritella	Serratia	0.093950708	0.145936939	Proteobacteria	Proteobacteria
Clostridium	Fervidobacterium	0.062850588	0.097552384	Firmicutes	Thermotogae
Actinobacteria	Micrococcus	0.072679127	0.112787558	Actinobacteria	Actinobacteria
Methanothermococcus	Methanococcus	0.360893548	0.559991956	Euryarchaeota	Euryarchaeota
Erysipelotrichaceae	Clostridium	0.062850588	0.097506784	Firmicutes	Firmicutes
Brevibacterium	Cnuibacter	0.125961995	0.195269636	Actinobacteria	Actinobacteria
Mycoplasma	Salimicrobium	0.025477347	0.039473801	Tenericutes	Firmicutes
Mycoplasma	Flavobacteriaceae	0.025477347	0.039447403	Tenericutes	Bacteroidetes
Sodalis	Morganella	0.189313104	0.292951154	Proteobacteria	Proteobacteria
Finegoldia	Eubacterium	0.078449324	0.121324913	Firmicutes	Firmicutes
Mycoplasma	Faecalibaculum	0.025477347	0.039348231	Tenericutes	Firmicutes
Anaerotignum	Mycoplasma	0.025477347	0.039335888	Firmicutes	Tenericutes
Calditerrivibrio	Mycoplasma	0.025477347	0.039313	Deferribacteres	Tenericutes
Cellulomonas	Brevibacterium	0.125961995	0.194252187	Actinobacteria	Actinobacteria
Rhizobacter	Pseudomonas	0.175575691	0.270595234	Proteobacteria	Proteobacteria
Zobellella	Pseudomonas	0.175575691	0.270591992	Proteobacteria	Proteobacteria
Negativicoccus	Mycoplasma	0.025477347	0.039130801	Firmicutes	Tenericutes
Treponema	Spirochaeta	0.076369812	0.117289981	Spirochaetes	Spirochaetes
Microcella	Brevibacterium	0.125961995	0.193249558	Actinobacteria	Actinobacteria
Kineococcus	Brevibacterium	0.125961995	0.193198945	Actinobacteria	Actinobacteria
Flavobacteriaceae	Tenacibaculum	0.161380659	0.247131813	Bacteroidetes	Bacteroidetes
Flavobacteriaceae	Lacinutrix	0.161380659	0.247090611	Bacteroidetes	Bacteroidetes
Weeksella	Mycoplasma	0.025477347	0.038911555	Bacteroidetes	Tenericutes
Brachyspira	Clostridium	0.068824019	0.105089696	Spirochaetes	Firmicutes
Ilumatobacter	Actinobacteria	0.072679127	0.110694553	Actinobacteria	Actinobacteria
Actinomyces	Brevibacterium	0.125961995	0.191770093	Actinobacteria	Actinobacteria
Enterococcus	Lactobacillus	0.158222465	0.24068499	Firmicutes	Firmicutes

Mycoplasma	Salinicoccus	0.025477347	0.038737093	Tenericutes	Firmicutes
Dietzia	Corynebacterium	0.177688971	0.270115664	Actinobacteria	Actinobacteria
Kocuria	Actinobacteria	0.072679127	0.110467095	Actinobacteria	Actinobacteria
Bacillus	Listeria	0.132343801	0.200583575	Firmicutes	Firmicutes
Berkelbacteria	Mycoplasma	0.025477347	0.038603031	null	Tenericutes
Achromobacter	Pseudomonas	0.175575691	0.26602314	Proteobacteria	Proteobacteria
Rothia	Actinobacteria	0.072679127	0.110054027	Actinobacteria	Actinobacteria
Desulfotomaculum	Pelotomaculum	0.198423348	0.300368461	Firmicutes	Firmicutes
Chromohalobacter	Pseudomonas	0.175575691	0.26529852	Proteobacteria	Proteobacteria
Rhodococcus	Brevibacterium	0.125961995	0.190224253	Actinobacteria	Actinobacteria
Arcobacter	Clostridium	0.062850588	0.094852418	Proteobacteria	Firmicutes
Dermabacter	Actinobacteria	0.072679127	0.109633846	Actinobacteria	Actinobacteria
Geosporobacter	Eubacterium	0.078449324	0.118245747	Firmicutes	Firmicutes
Escherichia	synthetic	0.662579626	0.997637044	Proteobacteria	Proteobacteria
Agrobacterium	Rhizobium	0.368942819	0.55508118	Proteobacteria	Proteobacteria
Treponema	Salinispira	0.076369812	0.114837532	Spirochaetes	Spirochaetes
Pelotomaculum	Clostridium	0.062850588	0.094466116	Firmicutes	Firmicutes
Acidovorax	Pseudomonas	0.175575691	0.263386858	Proteobacteria	Proteobacteria
Dietzia	Brevibacterium	0.125961995	0.188675998	Actinobacteria	Actinobacteria
Mycoplasma	Endomicrobium	0.025477347	0.038138026	Tenericutes	Elusimicrobia
Actinobacteria	Clavibacter	0.072679127	0.108779247	Actinobacteria	Actinobacteria
Peptoclostridium	Mycoplasma	0.025477347	0.038120393	Firmicutes	Tenericutes
Eubacterium	Ruminiclostridium	0.078449324	0.117350208	Firmicutes	Firmicutes
Clostridium	Salimicrobium	0.062850588	0.093976588	Firmicutes	Firmicutes
Erythrobacter	Altererythrobacter	0.319203322	0.477016621	Proteobacteria	Proteobacteria
Mycoplasma	Actinobacillus	0.025477347	0.038061584	Tenericutes	Proteobacteria
Eubacterium	Flavonifractor	0.078449324	0.117197089	Firmicutes	Firmicutes
Lachnoclostridium	Mycoplasma	0.025477347	0.038027077	Firmicutes	Tenericutes
Thermodesulfobium	Clostridium	0.062850588	0.093715739	Firmicutes	Firmicutes
Rathayibacter	Brevibacterium	0.125961995	0.187658179	Actinobacteria	Actinobacteria
Actinobacteria	Cellulomonas	0.072679127	0.108238115	Actinobacteria	Actinobacteria
Mycoplasma	Sebaldella	0.025477347	0.037901224	Tenericutes	Fusobacteria
Thermococcus	Palaeococcus	0.351634878	0.522811522	Euryarchaeota	Euryarchaeota
Mycoplasma	Bibersteinia	0.025477347	0.03785587	Tenericutes	Proteobacteria

Shewanella	Pseudoalteromonas	0.161098934	0.239194878	Proteobacteria	Proteobacteria
Geobacillus	Mycoplasma	0.025477347	0.037785601	Firmicutes	Tenericutes
Achromobacter	Bordetella	0.361544995	0.536155965	Proteobacteria	Proteobacteria
Actinobacillus	Haemophilus	0.356592956	0.52873453	Proteobacteria	Proteobacteria
Mycoplasma	Aneurinibacillus	0.025477347	0.037773097	Tenericutes	Firmicutes
Bacillus	Macrococcus	0.132343801	0.195961754	Firmicutes	Firmicutes
Actinomyces	Actinobacteria	0.072679127	0.107504174	Actinobacteria	Actinobacteria
Hippea	Clostridium	0.062850588	0.092936601	Proteobacteria	Firmicutes
Bacillus	Brochothrix	0.132343801	0.195576364	Firmicutes	Firmicutes
Actinobacillus	Mannheimia	0.384896686	0.568171296	Proteobacteria	Proteobacteria
Geobacter	Pelobacter	0.212486283	0.313660732	Proteobacteria	Proteobacteria
Tessaracoccus	Actinobacteria	0.072679127	0.107276707	Actinobacteria	Actinobacteria
Lentibacillus	Clostridium	0.062850588	0.092725668	Firmicutes	Firmicutes
Haemophilus	Aggregatibacter	0.356592956	0.525645469	Proteobacteria	Proteobacteria
Colwellia	Pseudoalteromonas	0.161098934	0.237454402	Proteobacteria	Proteobacteria
Janthinobacterium	Pseudomonas	0.175575691	0.258771654	Proteobacteria	Proteobacteria
Mycobacterium	Rhodococcus	0.255167927	0.375829427	Actinobacteria	Actinobacteria
Raoultella	Klebsiella	0.459467233	0.676632627	Proteobacteria	Proteobacteria
Mycoplasma	Desulfitobacterium	0.025477347	0.037497377	Tenericutes	Firmicutes
Glutamicibacter	Actinobacteria	0.072679127	0.10690643	Actinobacteria	Actinobacteria
Pseudomonas	Rubrivivax	0.175575691	0.258142303	Proteobacteria	Proteobacteria
Campylobacter	Arcobacter	0.172715367	0.253869809	Proteobacteria	Proteobacteria
Mycoplasma	Carboxydothermus	0.025477347	0.037429622	Tenericutes	Firmicutes
Geobacillus	Clostridium	0.062850588	0.092303835	Firmicutes	Firmicutes
Leuconostoc	Lactobacillus	0.158222465	0.232334122	Firmicutes	Firmicutes
Thermobacillus	Clostridium	0.062850588	0.092272332	Firmicutes	Firmicutes
Actinobacteria	Nocardioides	0.072679127	0.10664891	Actinobacteria	Actinobacteria
Rhodococcus	Corynebacterium	0.177688971	0.260419229	Actinobacteria	Actinobacteria
Delftia	Pseudomonas	0.175575691	0.257245366	Proteobacteria	Proteobacteria
Actinobacteria	Trueperella	0.072679127	0.106184548	Actinobacteria	Actinobacteria
Borreliella	Borrelia	0.685713958	0.999872023	Spirochaetes	Spirochaetes
Polaribacter	Flavobacteriaceae	0.161380659	0.235121754	Bacteroidetes	Bacteroidetes
Beutenbergia	Brevibacterium	0.125961995	0.183437073	Actinobacteria	Actinobacteria
Anaerococcus	Eubacterium	0.078449324	0.11375791	Firmicutes	Firmicutes

Paraburkholderia	Pandoraea	0.232579852	0.336960041	Proteobacteria	Proteobacteria
Mycoplasma	Pseudothermotoga	0.025477347	0.036877759	Tenericutes	Thermotogae
Idiomarina	Pseudoalteromonas	0.161098934	0.232957064	Proteobacteria	Proteobacteria
Mycoplasma	Lacinutrix	0.025477347	0.036820784	Tenericutes	Bacteroidetes
Mycoplasma	Gallibacterium	0.025477347	0.036793308	Tenericutes	Proteobacteria
Modestobacter	Brevibacterium	0.125961995	0.181302218	Actinobacteria	Actinobacteria
Christensenella	Eubacterium	0.078449324	0.112894712	Firmicutes	Firmicutes
Pseudarthrobacter	Actinobacteria	0.072679127	0.104483633	Actinobacteria	Actinobacteria
Bordetella	Pseudomonas	0.175575691	0.25233918	Proteobacteria	Proteobacteria
Alcanivorax	Pseudomonas	0.175575691	0.252085346	Proteobacteria	Proteobacteria
Actinobacteria	Microterricola	0.072679127	0.104285706	Actinobacteria	Actinobacteria
Syntrophothermus	Clostridium	0.062850588	0.090141241	Firmicutes	Firmicutes
Mycoplasma	Tumebacillus	0.025477347	0.036518739	Tenericutes	Firmicutes
Sulfurospirillum	Campylobacter	0.172965651	0.247846562	Proteobacteria	Proteobacteria
Xylanimonas	Actinobacteria	0.072679127	0.104111799	Actinobacteria	Actinobacteria
Siansivirga	Flavobacteriaceae	0.161380659	0.231092809	Bacteroidetes	Bacteroidetes
Brevibacterium	Streptomyces	0.125961995	0.180062712	Actinobacteria	Actinobacteria
Devriesea	Actinobacteria	0.072679127	0.103814273	Actinobacteria	Actinobacteria
Kytococcus	Actinobacteria	0.072679127	0.103763713	Actinobacteria	Actinobacteria
Castellaniella	Pseudomonas	0.175575691	0.250519384	Proteobacteria	Proteobacteria
Acidipropionibacteriur	Propionibacterium	0.255521499	0.364496262	Actinobacteria	Actinobacteria
Thermacetogenium	Clostridium	0.062850588	0.089639542	Firmicutes	Firmicutes
Rhodospirillum	Pararhodospirillum	0.265465699	0.37845423	Proteobacteria	Proteobacteria
Eubacterium	Caldicellulosiruptor	0.078449324	0.111787728	Firmicutes	Firmicutes
Thalassotalea	Pseudoalteromonas	0.161098934	0.229435065	Proteobacteria	Proteobacteria
Sodalis	Pragia	0.189313104	0.269325047	Proteobacteria	Proteobacteria
Actinobacteria	Arthrobacter	0.072679127	0.103371417	Actinobacteria	Actinobacteria
Oblitimonas	Pseudomonas	0.175575691	0.249230189	Proteobacteria	Proteobacteria
Marinobacter	Pseudomonas	0.175575691	0.248922825	Proteobacteria	Proteobacteria
Mycoplasma	Sulfurimonas	0.025477347	0.036093317	Tenericutes	Proteobacteria
Alicycliphilus	Pseudomonas	0.175575691	0.248719903	Proteobacteria	Proteobacteria
Megamonas	Eubacterium	0.078449324	0.110966528	Firmicutes	Firmicutes
Brevibacillus	Mycoplasma	0.025477347	0.036030589	Firmicutes	Tenericutes
Weissella	Clostridium	0.062850588	0.088854726	Firmicutes	Firmicutes

Anaerostipes	Lachnoclostridium	0.132857063	0.187814787	Firmicutes	Firmicutes
Caldanaerobacter	Eubacterium	0.078449324	0.11089832	Firmicutes	Firmicutes
Persephonella	Clostridium	0.062850588	0.088760305	Aquificae	Firmicutes
Flavobacteriaceae	Cellulophaga	0.161380659	0.227650437	Bacteroidetes	Bacteroidetes
Actinobacteria	Microbacterium	0.072679127	0.102485571	Actinobacteria	Actinobacteria
Azoarcus	Pseudomonas	0.175575691	0.246777416	Proteobacteria	Proteobacteria
Sodalis	Xenorhabdus	0.189313104	0.265986342	Proteobacteria	Proteobacteria
Christensenella	Clostridium	0.062850588	0.088242814	Firmicutes	Firmicutes
Mycoplasma	Polaribacter	0.025477347	0.035745439	Tenericutes	Bacteroidetes
Chromobacterium	Pseudomonas	0.175575691	0.246299394	Proteobacteria	Proteobacteria
Exiguobacterium	Clostridium	0.062850588	0.088131012	Firmicutes	Firmicutes
Tatlockia	Legionella	0.280731876	0.393534822	Proteobacteria	Proteobacteria
Comamonas	Acidovorax	0.306827503	0.430029976	Proteobacteria	Proteobacteria
Acetobacterium	Mycoplasma	0.025477347	0.035692934	Firmicutes	Tenericutes
Porphyrobacter	Erythrobacter	0.338583594	0.474322565	Proteobacteria	Proteobacteria
Marinobacterium	Pseudomonas	0.175575691	0.245926988	Proteobacteria	Proteobacteria
Clostridium	Lawsonia	0.063423572	0.08883293	Firmicutes	Proteobacteria
Leuconostoc	Clostridium	0.062850588	0.087981204	Firmicutes	Firmicutes
Methylibium	Pseudomonas	0.175575691	0.245506439	Proteobacteria	Proteobacteria
Paraburkholderia	Cupriavidus	0.232579852	0.325134225	Proteobacteria	Proteobacteria
Weeksella	Flavobacteriaceae	0.161380659	0.225568026	Bacteroidetes	Bacteroidetes
Paenarthrobacter	Actinobacteria	0.072679127	0.101566914	Actinobacteria	Actinobacteria
Acidipropionibacteriur	Brevibacterium	0.125961995	0.175712631	Actinobacteria	Actinobacteria
Treponema	Sphaerochaeta	0.076369812	0.10649538	Spirochaetes	Spirochaetes
Mycoplasma	Caldimicrobium	0.025477347	0.035504456	Tenericutes	Thermodesulfobacteria
Acidaminococcus	Clostridium	0.062850588	0.087535159	Firmicutes	Firmicutes
Cobetia	Pseudomonas	0.175575691	0.244250886	Proteobacteria	Proteobacteria
Basilea	Mycoplasma	0.025477347	0.035433338	Proteobacteria	Tenericutes
Pasteurella	Haemophilus	0.356592956	0.495849295	Proteobacteria	Proteobacteria
Photobacterium	Vibrio	0.271195069	0.376739209	Proteobacteria	Proteobacteria
Jeotgalibaca	Lactobacillus	0.158222465	0.219712615	Firmicutes	Firmicutes
Bacillus	Novibacillus	0.132343801	0.183700264	Firmicutes	Firmicutes
Mycobacterium	Nocardia	0.255167927	0.353993054	Actinobacteria	Actinobacteria
Actinobacteria	Acidimicrobium	0.072679127	0.100791448	Actinobacteria	Actinobacteria

Brevibacterium	Nocardiopsis	0.125961995	0.174255274	Actinobacteria	Actinobacteria
Riemerella	Mycoplasma	0.025477347	0.035236872	Bacteroidetes	Tenericutes
Bibersteinia	Mannheimia	0.384896686	0.532239386	Proteobacteria	Proteobacteria
Xanthomonas	Stenotrophomonas	0.330541251	0.456387654	Proteobacteria	Proteobacteria
Haemophilus	Mannheimia	0.356592956	0.492106189	Proteobacteria	Proteobacteria
Moorella	Clostridium	0.062850588	0.08665908	Firmicutes	Firmicutes
Paraburkholderia	Ralstonia	0.232579852	0.320340866	Proteobacteria	Proteobacteria
Sporosarcina	Lysinibacillus	0.284988147	0.392131991	Firmicutes	Firmicutes
Mycoplasma	Helicobacter	0.025477347	0.035031196	Tenericutes	Proteobacteria
Mycoplasma	Siansivirga	0.025477347	0.034994452	Tenericutes	Bacteroidetes
Alicycliphilus	Comamonas	0.306827503	0.42112668	Proteobacteria	Proteobacteria
Leptothrix	Pseudomonas	0.175575691	0.240846387	Proteobacteria	Proteobacteria
Clostridium	Spiroplasma	0.062850588	0.086209942	Firmicutes	Tenericutes
Serratia	Pseudoalteromonas	0.093950708	0.128750823	Proteobacteria	Proteobacteria
Kushneria	Pseudomonas	0.175575691	0.24056274	Proteobacteria	Proteobacteria
Kocuria	Corynebacterium	0.201379737	0.275900753	Actinobacteria	Actinobacteria
Sanguibacter	Actinobacteria	0.072679127	0.099538999	Actinobacteria	Actinobacteria
Bacillus	Salinicoccus	0.132343801	0.181205095	Firmicutes	Firmicutes
Mycoplasma	Geosporobacter	0.025477347	0.034865396	Tenericutes	Firmicutes
Flavobacterium	Flavobacteriaceae	0.161380659	0.220744555	Bacteroidetes	Bacteroidetes
Actinobacteria	Brevibacterium	0.072679127	0.0991609	Actinobacteria	Actinobacteria
Cyanobium	Prochlorococcus	0.216386652	0.295032518	Cyanobacteria	Cyanobacteria
Bibersteinia	Haemophilus	0.356592956	0.485451493	Proteobacteria	Proteobacteria
Rhodococcus	Nocardia	0.313040186	0.425800527	Actinobacteria	Actinobacteria
Thauera	Pseudomonas	0.175575691	0.238440028	Proteobacteria	Proteobacteria
Polyangium	Pseudomonas	0.175575691	0.238422811	Proteobacteria	Proteobacteria
Campylobacter	Clostridium	0.062850588	0.085046346	Proteobacteria	Firmicutes
Xanthomonas	Pseudoxanthomonas	0.330541251	0.447010634	Proteobacteria	Proteobacteria
Wolinella	Helicobacter	0.162576286	0.219557176	Proteobacteria	Proteobacteria
Diaphorobacter	Comamonas	0.306827503	0.414317764	Proteobacteria	Proteobacteria
Bacillus	Auricoccus	0.132343801	0.178547788	Firmicutes	Firmicutes
Clostridium	Endomicrobium	0.063423572	0.085532933	Firmicutes	Elusimicrobia
Actinobacteria	Mobiluncus	0.072679127	0.097943194	Actinobacteria	Actinobacteria
Mesoplasma	Clostridium	0.062850588	0.084465183	Tenericutes	Firmicutes

Stenotrophomonas	Pseudomonas	0.175575691	0.23581169	Proteobacteria	Proteobacteria
Ramlibacter	Pseudomonas	0.175575691	0.235567586	Proteobacteria	Proteobacteria
Paraphotobacterium	Clostridium	0.062850588	0.0841907	Proteobacteria	Firmicutes
Bacillus	Carnobacterium	0.132343801	0.177125052	Firmicutes	Firmicutes
Calditerrivibrio	Clostridium	0.062850588	0.0840818	Deferribacteres	Firmicutes
Gordonia	Mycobacterium	0.255167927	0.340634265	Actinobacteria	Actinobacteria
Acetohalobium	Eubacterium	0.078449324	0.104642045	Firmicutes	Firmicutes
Mycoplasma	Desulfotomaculum	0.025477347	0.033977022	Tenericutes	Firmicutes
Eubacterium	Ruminococcaceae	0.078449324	0.104573601	Firmicutes	Firmicutes
Actinobacteria	Tropheryma	0.072679127	0.096779004	Actinobacteria	Actinobacteria
Renibacterium	Brevibacterium	0.125961995	0.167591308	Actinobacteria	Actinobacteria
Intestinimonas	Eubacterium	0.088134527	0.117233285	Firmicutes	Firmicutes
Mycoplasma	Wenyingzhuangia	0.025477347	0.033842977	Tenericutes	Bacteroidetes
Escherichia	Shigella	0.662579626	0.87900418	Proteobacteria	Proteobacteria
Eubacterium	Paenibacillus	0.078449324	0.104046715	Firmicutes	Firmicutes
Mycoplasma	Aggregatibacter	0.025477347	0.033769422	Tenericutes	Proteobacteria
Blastococcus	Actinobacteria	0.072679127	0.096321865	Actinobacteria	Actinobacteria
Wenyingzhuangia	Flavobacteriaceae	0.161380659	0.213716375	Bacteroidetes	Bacteroidetes
Gordonia	Brevibacterium	0.125961995	0.166502	Actinobacteria	Actinobacteria
Campylobacter	Helicobacter	0.162576286	0.214872583	Proteobacteria	Proteobacteria
Providencia	Sodalis	0.189313104	0.250208466	Proteobacteria	Proteobacteria
Pelosinus	Mycoplasma	0.025477347	0.033657878	Firmicutes	Tenericutes
Pantoea	Plautia	0.477738095	0.630965473	Proteobacteria	Proteobacteria
Lawsonella	Brevibacterium	0.125961995	0.166279214	Actinobacteria	Actinobacteria
Porphyrobacter	Altererythrobacter	0.319203322	0.421151005	Proteobacteria	Proteobacteria
Kineococcus	Actinobacteria	0.072679127	0.095879401	Actinobacteria	Actinobacteria
Geoglobus	Archaeoglobus	0.272030401	0.358818922	Euryarchaeota	Euryarchaeota
Ensifer	Sinorhizobium	0.503379987	0.663326698	Proteobacteria	Proteobacteria
Caldisericum	Clostridium	0.062850588	0.082815844	Caldiserica	Firmicutes
Sodalis	Plesiomonas	0.189313104	0.249356202	Proteobacteria	Proteobacteria
Desulfuromonas	Pelobacter	0.212486283	0.27980406	Proteobacteria	Proteobacteria
Alicycliphilus	Acidovorax	0.464019965	0.610766852	Proteobacteria	Proteobacteria
Pluralibacter	Pseudomonas	0.175575691	0.231041022	Proteobacteria	Proteobacteria
Proteus	Sodalis	0.189313104	0.248949842	Proteobacteria	Proteobacteria

Clostridium	Mageeibacillus	0.062850588	0.082580564	Firmicutes	Firmicutes
Actinobacteria	Bifidobacterium	0.072679127	0.095455853	Actinobacteria	Actinobacteria
Actinotignum	Actinobacteria	0.072679127	0.095308536	Actinobacteria	Actinobacteria
Wigglesworthia	Clostridium	0.062850588	0.08237671	Proteobacteria	Firmicutes
Halomonas	Chromohalobacter	0.348529712	0.456460313	Proteobacteria	Proteobacteria
Brevibacterium	Tsukamurella	0.125961995	0.164785032	Actinobacteria	Actinobacteria
Sinomonas	Actinobacteria	0.072679127	0.094918139	Actinobacteria	Actinobacteria
Mycoplasma	Ehrlichia	0.025477347	0.033270463	Tenericutes	Proteobacteria
Actinobacteria	Cryobacterium	0.072679127	0.094730203	Actinobacteria	Actinobacteria
Nakamurella	Brevibacterium	0.125961995	0.164168214	Actinobacteria	Actinobacteria
Acidipropionibacteriur	Actinobacteria	0.072679127	0.094659533	Actinobacteria	Actinobacteria
Hoyosella	Actinobacteria	0.072679127	0.094591676	Actinobacteria	Actinobacteria
Thioalkalivibrio	Pseudomonas	0.175575691	0.228390459	Proteobacteria	Proteobacteria
Saccharomonospora	Actinobacteria	0.072679127	0.094528262	Actinobacteria	Actinobacteria
Mycoplasma	Wolbachia	0.025477347	0.033109108	Tenericutes	Proteobacteria
Clostridium	Selenomonas	0.062850588	0.081601674	Firmicutes	Firmicutes
Nautilia	Clostridium	0.062850588	0.081443808	Proteobacteria	Firmicutes
Lysinibacillus	Solibacillus	0.284988147	0.36918018	Firmicutes	Firmicutes
Diaphorobacter	Pseudomonas	0.175575691	0.226419101	Proteobacteria	Proteobacteria
Mycoplasma	Chlamydia	0.025477347	0.03284315	Tenericutes	Chlamydiae
Tumebacillus	Clostridium	0.062850588	0.081002819	Firmicutes	Firmicutes
Polynucleobacter	beta	0.518661102	0.668352402	Proteobacteria	Proteobacteria
Grimontia	Vibrio	0.271195069	0.348913094	Proteobacteria	Proteobacteria
Flexistipes	Clostridium	0.062850588	0.080644068	Deferribacteres	Firmicutes
Oenococcus	Lactobacillus	0.158222465	0.202842512	Firmicutes	Firmicutes
Enterobacter	Klebsiella	0.459467233	0.588752424	Proteobacteria	Proteobacteria
Immundisolibacter	Pseudomonas	0.175575691	0.224970654	Proteobacteria	Proteobacteria
Geoalkalibacter	Pelobacter	0.212486283	0.272087734	Proteobacteria	Proteobacteria
Corynebacterium	Micrococcus	0.177688971	0.227382938	Actinobacteria	Actinobacteria
Aromatoleum	Pseudomonas	0.175575691	0.224296485	Proteobacteria	Proteobacteria
Lelliottia	Enterobacter	0.557933943	0.712689741	Proteobacteria	Proteobacteria
Aeromonas	Pseudomonas	0.175575691	0.223789053	Proteobacteria	Proteobacteria
Alkalilimnicola	Pseudomonas	0.175575691	0.223293712	Proteobacteria	Proteobacteria
Mycoplasma	Moraxella	0.025477347	0.032389485	Tenericutes	Proteobacteria

Eubacterium	Dehalobacterium	0.078449324	0.099728845	Firmicutes	Firmicutes
Curtobacterium	Actinobacteria	0.072679127	0.092387011	Actinobacteria	Actinobacteria
Mycoplasma	Ornithobacterium	0.025477347	0.032365823	Tenericutes	Bacteroidetes
Rhodococcus	Actinobacteria	0.072679127	0.092305667	Actinobacteria	Actinobacteria
Kluyvera	Klebsiella	0.459467233	0.582870127	Proteobacteria	Proteobacteria
Novibacillus	Clostridium	0.062850588	0.079721226	Firmicutes	Firmicutes
Cellulosimicrobium	Actinobacteria	0.072679127	0.092169021	Actinobacteria	Actinobacteria
Nonlabens	Flavobacteriaceae	0.161380659	0.204601585	Bacteroidetes	Bacteroidetes
Butyrivibrio	Mycoplasma	0.025477347	0.032298362	Firmicutes	Tenericutes
Luteipulveratus	Actinobacteria	0.072679127	0.09210128	Actinobacteria	Actinobacteria
Photorhabdus	Sodalis	0.189313104	0.239883287	Proteobacteria	Proteobacteria
Bacillus	Tumebacillus	0.132343801	0.167601796	Firmicutes	Firmicutes
Orientia	Mycoplasma	0.025477347	0.032261359	Proteobacteria	Tenericutes
Mycobacterium	Actinobacteria	0.072679127	0.092019114	Actinobacteria	Actinobacteria
Citrobacter	Klebsiella	0.459467233	0.581223958	Proteobacteria	Proteobacteria
Pantoea	Erwinia	0.467795284	0.591147238	Proteobacteria	Proteobacteria
Ezakiella	Eubacterium	0.078449324	0.099126298	Firmicutes	Firmicutes
Aerococcus	Lactobacillus	0.158222465	0.199852965	Firmicutes	Firmicutes
Chryseobacterium	Elizabethkingia	0.314554333	0.397076561	Bacteroidetes	Bacteroidetes
Mycoplasma	Ignavibacterium	0.025477347	0.032128464	Tenericutes	Bacteroidetes
Mycoplasma	Chlamydophila	0.025477347	0.032126309	Tenericutes	Chlamydiae
Salinicoccus	Clostridium	0.062850588	0.079242237	Firmicutes	Firmicutes
Agromyces	Actinobacteria	0.072679127	0.091581543	Actinobacteria	Actinobacteria
Brevibacillus	Paenibacillus	0.161111346	0.202964701	Firmicutes	Firmicutes
Klebsiella	Enterobacteriaceae	0.459467233	0.57806237	Proteobacteria	Proteobacteria
Geodermatophilus	Actinobacteria	0.072679127	0.091437305	Actinobacteria	Actinobacteria
Ornithobacterium	Flavobacteriaceae	0.161380659	0.203017183	Bacteroidetes	Bacteroidetes
Halanaerobium	Eubacterium	0.078449324	0.098673196	Firmicutes	Firmicutes
Eubacterium	Megasphaera	0.078449324	0.098620655	Firmicutes	Firmicutes
Mycoplasma	Nonlabens	0.025477347	0.032018928	Tenericutes	Bacteroidetes
Aliivibrio	Pseudoalteromonas	0.161098934	0.202357986	Proteobacteria	Proteobacteria
Streptococcus	Eubacterium	0.078449324	0.098466795	Firmicutes	Firmicutes
Blattabacterium	Clostridium	0.062850588	0.078799513	Bacteroidetes	Firmicutes
Phaeobacter	Ruegeria	0.394096176	0.494094108	Proteobacteria	Proteobacteria

Actinobacteria	Tsukamurella	0.072679127	0.091117946	Actinobacteria	Actinobacteria
Sulfurospirillum	Mycoplasma	0.025477347	0.031930486	Proteobacteria	Tenericutes
Flavobacteriaceae	Gramella	0.161380659	0.202237586	Bacteroidetes	Bacteroidetes
Allofrancisella	Clostridium	0.062850588	0.07871971	Proteobacteria	Firmicutes
Actinobacteria	Thermomonospora	0.072679127	0.090932413	Actinobacteria	Actinobacteria
Dermabacter	Brevibacterium	0.125961995	0.157420716	Actinobacteria	Actinobacteria
Laribacter	Pseudomonas	0.175575691	0.219350618	Proteobacteria	Proteobacteria
Brachybacterium	Actinobacteria	0.072679127	0.090729228	Actinobacteria	Actinobacteria
Mycoplasma	Croceibacter	0.025477347	0.031778571	Tenericutes	Bacteroidetes
Gardnerella	Actinobacteria	0.072679127	0.090642326	Actinobacteria	Actinobacteria
Pseudoxanthomonas	Pseudomonas	0.175575691	0.218906646	Proteobacteria	Proteobacteria
Brevibacterium	Propionibacterium	0.125961995	0.157025744	Actinobacteria	Actinobacteria
Legionella	Mycoplasma	0.025477347	0.031753638	Proteobacteria	Tenericutes
Flavobacteriaceae	Dokdonia	0.161380659	0.200880695	Bacteroidetes	Bacteroidetes
Mycoplasma	Persephonella	0.025477347	0.031677318	Tenericutes	Aquificae
Marichromatium	Pseudomonas	0.175575691	0.21826831	Proteobacteria	Proteobacteria
Thiohalobacter	Pseudomonas	0.175575691	0.218256826	Proteobacteria	Proteobacteria
Oceanisphaera	Pseudoalteromonas	0.161098934	0.200045467	Proteobacteria	Proteobacteria
Buchnera	Clostridium	0.062850588	0.077957067	Proteobacteria	Firmicutes
Leclercia	Klebsiella	0.459467233	0.569722222	Proteobacteria	Proteobacteria
Polaromonas	Pseudomonas	0.175575691	0.217645034	Proteobacteria	Proteobacteria
Actinobacteria	Streptomyces	0.072679127	0.090016324	Actinobacteria	Actinobacteria
Roseateles	Pseudomonas	0.175575691	0.217402937	Proteobacteria	Proteobacteria
Salinispora	Actinobacteria	0.072679127	0.089990368	Actinobacteria	Actinobacteria
Mycoplasma	Coxiella-like	0.025477347	0.031529816	Tenericutes	Proteobacteria
Actinobacteria	Cnuibacter	0.072679127	0.089935479	Actinobacteria	Actinobacteria
Brochothrix	Lactobacillus	0.158222465	0.195729913	Firmicutes	Firmicutes
Vibrio	Pseudoalteromonas	0.161098934	0.199101963	Proteobacteria	Proteobacteria
Lelliottia	Klebsiella	0.459467233	0.56783135	Proteobacteria	Proteobacteria
Photobacterium	Pseudoalteromonas	0.161098934	0.19896605	Proteobacteria	Proteobacteria
Bacillus	Vagococcus	0.132343801	0.163131441	Firmicutes	Firmicutes
Burkholderiales	Basilea	0.092214558	0.113606398	Proteobacteria	Proteobacteria
Pseudopropionibacteri	Actinobacteria	0.072679127	0.089489387	Actinobacteria	Actinobacteria
Kluyvera	Enterobacteriaceae	0.511696136	0.629420579	Proteobacteria	Proteobacteria

Bibersteinia	Actinobacillus	0.409596236	0.503761533	Proteobacteria	Proteobacteria
Flavobacterium	Lacinutrix	0.260303566	0.319814055	Bacteroidetes	Bacteroidetes
Hoyosella	Brevibacterium	0.125961995	0.154735233	Actinobacteria	Actinobacteria
Mycoplasma	Murdochiella	0.025477347	0.031278085	Tenericutes	Firmicutes
Kitasatospora	Brevibacterium	0.125961995	0.154413228	Actinobacteria	Actinobacteria
Segniliparus	Actinobacteria	0.072679127	0.088984844	Actinobacteria	Actinobacteria
Mycobacterium	Tsukamurella	0.255167927	0.312319742	Actinobacteria	Actinobacteria
Corynebacterium	Lawsonella	0.177688971	0.217378199	Actinobacteria	Actinobacteria
Thermosediminibacter	Eubacterium	0.078449324	0.09580293	Firmicutes	Firmicutes
Beutenbergia	Actinobacteria	0.072679127	0.08870471	Actinobacteria	Actinobacteria
Mycoplasma	Flexistipes	0.025477347	0.03108187	Tenericutes	Deferribacteres
Corynebacterium	Tsukamurella	0.206499705	0.251741397	Actinobacteria	Actinobacteria
Borrelia	Clostridium	0.062850588	0.076568021	Spirochaetes	Firmicutes
Thiomonas	Pseudomonas	0.175575691	0.213786004	Proteobacteria	Proteobacteria
Borreliella	Clostridium	0.062850588	0.07651537	Spirochaetes	Firmicutes
Clostridium	Faecalibaculum	0.062850588	0.076361935	Firmicutes	Firmicutes
Moritella	Pseudoalteromonas	0.161098934	0.195489072	Proteobacteria	Proteobacteria
Mycoplasma	Neorickettsia	0.025477347	0.03086468	Tenericutes	Proteobacteria
Modestobacter	Actinobacteria	0.072679127	0.087890931	Actinobacteria	Actinobacteria
Leclercia	Enterobacter	0.557933943	0.674541613	Proteobacteria	Proteobacteria
Tepidanaerobacter	Eubacterium	0.078449324	0.094803348	Firmicutes	Firmicutes
Celeribacter	Pelagibaca	0.319579628	0.385809234	Proteobacteria	Proteobacteria
Capnocytophaga	Flavobacteriaceae	0.161380659	0.194336724	Bacteroidetes	Bacteroidetes
Mycoplasma	cyanobacterium	0.025477347	0.030669709	Tenericutes	Cyanobacteria
Hafnia	Obesumbacterium	0.699265576	0.841609104	Proteobacteria	Proteobacteria
Raoultella	Pseudomonas	0.175575691	0.211232378	Proteobacteria	Proteobacteria
Desulfitobacterium	Eubacterium	0.078449324	0.094352996	Firmicutes	Firmicutes
Aminobacterium	Clostridium	0.062850588	0.075589094	Synergistetes	Firmicutes
Eubacterium	Ethanoligenens	0.078449324	0.094288123	Firmicutes	Firmicutes
Xanthomonas	Pseudomonas	0.175575691	0.210977774	Proteobacteria	Proteobacteria
Dietzia	Actinobacteria	0.072679127	0.087316618	Actinobacteria	Actinobacteria
Negativicoccus	Clostridium	0.062850588	0.075446696	Firmicutes	Firmicutes
Pluralibacter	Klebsiella	0.459467233	0.550925064	Proteobacteria	Proteobacteria
Clostridium	Pseudothermotoga	0.062850588	0.075358911	Firmicutes	Thermotogae

Faecalibacterium	Lachnoclostridium	0.132857063	0.159260912	Firmicutes	Firmicutes
Acidianus	Sulfolobus	0.34651521	0.41512082	Crenarchaeota	Crenarchaeota
Pimelobacter	Actinobacteria	0.072679127	0.087033655	Actinobacteria	Actinobacteria
Eubacterium	Lactobacillus	0.078449324	0.09391684	Firmicutes	Firmicutes
Dehalobacter	Eubacterium	0.078449324	0.093818177	Firmicutes	Firmicutes
Desulfosporosinus	Eubacterium	0.078449324	0.093800785	Firmicutes	Firmicutes
Agarivorans	Pseudoalteromonas	0.161098934	0.192562435	Proteobacteria	Proteobacteria
Jonesia	Brevibacterium	0.125961995	0.150499933	Actinobacteria	Actinobacteria
Erysipelotrichaceae	Eubacterium	0.078449324	0.093721414	Firmicutes	Firmicutes
Celeribacter	Thiobacimonas	0.319579628	0.381763073	Proteobacteria	Proteobacteria
Burkholderiales	Taylorella	0.092214558	0.110005522	Proteobacteria	Proteobacteria
Halothermothrix	Eubacterium	0.078449324	0.093524053	Firmicutes	Firmicutes
Sphingobium	Sphingomonas	0.292558438	0.348739834	Proteobacteria	Proteobacteria
Comamonas	Pseudomonas	0.175575691	0.209245364	Proteobacteria	Proteobacteria
Lutibacter	Clostridium	0.063423572	0.075559169	Bacteroidetes	Firmicutes
Listeria	Lactobacillus	0.158222465	0.188394972	Firmicutes	Firmicutes
Corynebacterium	Nocardia	0.201379737	0.239447569	Actinobacteria	Actinobacteria
Enterococcus	Melissococcus	0.302178503	0.359248851	Firmicutes	Firmicutes
Kluyvera	Kosakonia	0.511696136	0.608103351	Proteobacteria	Proteobacteria
Corynebacterium	Actinomyces	0.177688971	0.210880889	Actinobacteria	Actinobacteria
Allochromatium	Pseudomonas	0.175575691	0.208360855	Proteobacteria	Proteobacteria
Kitasatospora	Actinobacteria	0.072679127	0.086249421	Actinobacteria	Actinobacteria
Leisingera	Ruegeria	0.394096176	0.467524527	Proteobacteria	Proteobacteria
Devriesea	Brevibacterium	0.125961995	0.149300907	Actinobacteria	Actinobacteria
Klebsiella	Pseudomonas	0.175575691	0.208030998	Proteobacteria	Proteobacteria
Bacillus	Thermobacillus	0.132343801	0.156659279	Firmicutes	Firmicutes
Aggregatibacter	Mannheimia	0.396279068	0.468947162	Proteobacteria	Proteobacteria
Frischella	Clostridium	0.063423572	0.074990014	Proteobacteria	Firmicutes
Mycoplasma	Photobacterium	0.025477347	0.030123488	Tenericutes	Proteobacteria
Azospirillum	Rhodospirillum	0.265465699	0.313760753	Proteobacteria	Proteobacteria
Arsenophonus	Clostridium	0.062850588	0.074131822	Proteobacteria	Firmicutes
Streptococcus	Lactobacillus	0.158222465	0.186600817	Firmicutes	Firmicutes
Mycoplasma	Kosmotoga	0.025477347	0.030026356	Tenericutes	Thermotogae
Mycoplasma	Aminobacterium	0.025477347	0.030017827	Tenericutes	Synergistetes

Verminephrobacter	Pseudomonas	0.175575691	0.206807336	Proteobacteria	Proteobacteria
Mycoplasma	Ruminiclostridium	0.025477347	0.029986288	Tenericutes	Firmicutes
Celeribacter	Defluviimonas	0.319579628	0.376067168	Proteobacteria	Proteobacteria
Clostridium	Serratia	0.062850588	0.073958181	Firmicutes	Proteobacteria
Treponema	Clostridium	0.062850588	0.07395543	Spirochaetes	Firmicutes
Riemerella	Chryseobacterium	0.314554333	0.370080288	Bacteroidetes	Bacteroidetes
Erwinia	Pseudomonas	0.175575691	0.206430243	Proteobacteria	Proteobacteria
Agrobacterium	Neorhizobium	0.368942819	0.433699427	Proteobacteria	Proteobacteria
Mycoplasma	Thermosulfidibacter	0.025477347	0.029938552	Tenericutes	Aquificae
Mycobacterium	Corynebacterium	0.206499705	0.242550624	Actinobacteria	Actinobacteria
Eubacterium	Selenomonas	0.078449324	0.092113846	Firmicutes	Firmicutes
Kosakonia	Klebsiella	0.459467233	0.539416143	Proteobacteria	Proteobacteria
Amphibacillus	Eubacterium	0.078449324	0.092072302	Firmicutes	Firmicutes
Halorhodospira	Pseudomonas	0.175575691	0.205940902	Proteobacteria	Proteobacteria
Betaproteobacteria	Pseudomonas	0.175575691	0.205774716	Proteobacteria	Proteobacteria
Algibacter	Mycoplasma	0.025477347	0.029837289	Bacteroidetes	Tenericutes
Microbulbifer	Pseudomonas	0.175575691	0.205440828	Proteobacteria	Proteobacteria
Archaeoglobus	Ferroglobus	0.272030401	0.318097427	Euryarchaeota	Euryarchaeota
Dyella	Pseudomonas	0.175575691	0.205210198	Proteobacteria	Proteobacteria
Clostridium	Thermovirga	0.062850588	0.073446312	Firmicutes	Synergistetes
Grimontia	Pseudoalteromonas	0.161098934	0.188233743	Proteobacteria	Proteobacteria
Dialister	Eubacterium	0.078449324	0.091636015	Firmicutes	Firmicutes
Mycoplasma	Atopobium	0.025477347	0.029752888	Tenericutes	Actinobacteria
Lysinibacillus	Rummeliibacillus	0.284988147	0.332580617	Firmicutes	Firmicutes
Rothia	Brevibacterium	0.125961995	0.146859551	Actinobacteria	Actinobacteria
Desulfotomaculum	Thermincola	0.198423348	0.231225797	Firmicutes	Firmicutes
Eubacterium	Fusobacterium	0.078449324	0.091374493	Firmicutes	Fusobacteria
Thiobacillus	Pseudomonas	0.175575691	0.204458784	Proteobacteria	Proteobacteria
Azospirillum	Pseudomonas	0.175575691	0.204401886	Proteobacteria	Proteobacteria
Gibbsiella	Pseudomonas	0.175575691	0.204340476	Proteobacteria	Proteobacteria
Mycoplasma	Paenibacillus	0.025477347	0.029651262	Tenericutes	Firmicutes
Celeribacter	Marinovum	0.319579628	0.371566395	Proteobacteria	Proteobacteria
Erysipelotrichaceae	Mycoplasma	0.025477347	0.029602539	Firmicutes	Tenericutes
Aneurinibacillus	Paenibacillus	0.161111346	0.186953849	Firmicutes	Firmicutes

Oenococcus	Clostridium	0.062850588	0.072864771	Firmicutes	Firmicutes
Phoenicibacter	Clostridium	0.062850588	0.072736128	Actinobacteria	Firmicutes
Sulfuritalea	Pseudomonas	0.175575691	0.202857904	Proteobacteria	Proteobacteria
Tenacibaculum	Clostridium	0.062850588	0.072571526	Bacteroidetes	Firmicutes
Cutibacterium	Brevibacterium	0.125961995	0.14538884	Actinobacteria	Actinobacteria
Siansivirga	Flavobacterium	0.260303566	0.300158558	Bacteroidetes	Bacteroidetes
Mycobacterium	Amycolatopsis	0.255167927	0.293906907	Actinobacteria	Actinobacteria
Actinobacteria	Nocardiopsis	0.072679127	0.083593747	Actinobacteria	Actinobacteria
Rhodanobacter	Pseudomonas	0.175575691	0.201786107	Proteobacteria	Proteobacteria
Altererythrobacter	Croceicoccus	0.319203322	0.36659749	Proteobacteria	Proteobacteria
Nocardia	Brevibacterium	0.125961995	0.144659422	Actinobacteria	Actinobacteria
Mycoplasma	Dehalobacterium	0.025477347	0.029255645	Tenericutes	Firmicutes
Mycoplasma	Moritella	0.025477347	0.029253025	Tenericutes	Proteobacteria
Flavobacterium	Tenacibaculum	0.260303566	0.298542889	Bacteroidetes	Bacteroidetes
Bacillus	Lactobacillus	0.132343801	0.151775842	Firmicutes	Firmicutes
Mycoplasma	Desulfosporosinus	0.025477347	0.029210983	Tenericutes	Firmicutes
Vagococcus	Enterococcus	0.302178503	0.346387509	Firmicutes	Firmicutes
Murdochiella	Clostridium	0.062850588	0.071960915	Firmicutes	Firmicutes
Plautia	Pseudomonas	0.175575691	0.200964124	Proteobacteria	Proteobacteria
Pelosinus	Eubacterium	0.078449324	0.089789834	Firmicutes	Firmicutes
Flavobacteriaceae	Clostridium	0.062850588	0.071918553	Bacteroidetes	Firmicutes
Pseudoxanthomonas	Stenotrophomonas	0.411250316	0.470070181	Proteobacteria	Proteobacteria
Leclercia	Pseudomonas	0.175575691	0.200480314	Proteobacteria	Proteobacteria
Komagataeibacter	Acetobacter	0.328547737	0.374485312	Proteobacteria	Proteobacteria
Rhodoferax	Pseudomonas	0.175575691	0.200081223	Proteobacteria	Proteobacteria
Kluyvera	Enterobacter	0.511696136	0.58292406	Proteobacteria	Proteobacteria
Escherichia	Klebsiella	0.459467233	0.523361367	Proteobacteria	Proteobacteria
Ramlibacter	Betaproteobacteria	0.23303541	0.265390911	Proteobacteria	Proteobacteria
Mycoplasma	Ndongobacter	0.025477347	0.028988527	Tenericutes	Firmicutes
Eubacterium	Desulfotomaculum	0.078449324	0.089176179	Firmicutes	Firmicutes
Baumannia	Clostridium	0.062850588	0.071423359	Proteobacteria	Firmicutes
Halobacteroides	Eubacterium	0.088134527	0.10012125	Firmicutes	Firmicutes
Ndongobacter	Clostridium	0.062850588	0.071356932	Firmicutes	Firmicutes
Novosphingobium	Altererythrobacter	0.319203322	0.362210681	Proteobacteria	Proteobacteria

Psychromonas	Pseudoalteromonas	0.161098934	0.182787606	Proteobacteria	Proteobacteria
Gilliamella	Clostridium	0.063423572	0.071945445	Proteobacteria	Firmicutes
Bacillus	Enterococcus	0.132343801	0.150118893	Firmicutes	Firmicutes
Salmonella	Klebsiella	0.459467233	0.52053946	Proteobacteria	Proteobacteria
Bacillus	Tetragenococcus	0.132343801	0.149790259	Firmicutes	Firmicutes
Actinobacteria	Microlunatus	0.072679127	0.082237636	Actinobacteria	Actinobacteria
Planococcus	Lysinibacillus	0.284988147	0.32246613	Firmicutes	Firmicutes
Rhodospirillum	Nitrospirillum	0.265465699	0.300207643	Proteobacteria	Proteobacteria
Listeria	Eubacterium	0.078449324	0.088609495	Firmicutes	Firmicutes
Pasteurella	Mannheimia	0.384896686	0.434742035	Proteobacteria	Proteobacteria
Mycoplasma	Myroides	0.025477347	0.028775996	Tenericutes	Bacteroidetes
Tetragenococcus	Enterococcus	0.302178503	0.341184375	Firmicutes	Firmicutes
Gluconacetobacter	Acetobacter	0.328547737	0.370942914	Proteobacteria	Proteobacteria
Mycoplasma	Snodgrassella	0.025477347	0.028753773	Tenericutes	Proteobacteria
Bacterioplanes	Pseudomonas	0.175575691	0.197945174	Proteobacteria	Proteobacteria
Kosakonia	Pseudomonas	0.175575691	0.197940611	Proteobacteria	Proteobacteria
Lysobacter	Pseudomonas	0.175575691	0.197799606	Proteobacteria	Proteobacteria
synthetic	Klebsiella	0.459467233	0.516706188	Proteobacteria	Proteobacteria
Celeribacter	Roseibacterium	0.319579628	0.35877619	Proteobacteria	Proteobacteria
Mycoplasma	Kingella	0.025477347	0.028601543	Tenericutes	Proteobacteria
Kluyvera	Raoultella	0.511696136	0.574338319	Proteobacteria	Proteobacteria
Gallibacterium	Haemophilus	0.356592956	0.399894927	Proteobacteria	Proteobacteria
Cronobacter	Pseudomonas	0.175575691	0.196778176	Proteobacteria	Proteobacteria
Simiduia	Pseudomonas	0.175575691	0.196748886	Proteobacteria	Proteobacteria
Mycobacterium	Brevibacterium	0.125961995	0.141009142	Actinobacteria	Actinobacteria
Symbiobacterium	Clostridium	0.062850588	0.070353553	Firmicutes	Firmicutes
Eubacterium	Thermincola	0.078449324	0.087709511	Firmicutes	Firmicutes
Mycoplasma	Ruminococcaceae	0.025477347	0.028459178	Tenericutes	Firmicutes
Neisseria	Eikenella	0.397279617	0.443629046	Proteobacteria	Proteobacteria
Buchnera	Wigglesworthia	0.371396499	0.414669546	Proteobacteria	Proteobacteria
Mycoplasma	Thermovirga	0.025477347	0.028435549	Tenericutes	Synergistetes
Ferrimonas	Pseudomonas	0.175575691	0.195711629	Proteobacteria	Proteobacteria
Desulfobulbus	Desulfovibrio	0.143678238	0.16012336	Proteobacteria	Proteobacteria
Bacillus	Jeotgalibaca	0.132343801	0.1474754	Firmicutes	Firmicutes

Flavobacteriaceae	Myroides	0.161380659	0.179816671	Bacteroidetes	Bacteroidetes
Comamonas	Ramlibacter	0.306827503	0.341748374	Proteobacteria	Proteobacteria
Actinopolyspora	Actinobacteria	0.072679127	0.080948363	Actinobacteria	Actinobacteria
Thermotoga	Clostridium	0.062850588	0.069996565	Thermotogae	Firmicutes
Dermatophilus	Brevibacterium	0.125961995	0.140195074	Actinobacteria	Actinobacteria
Dehalobacter	Mycoplasma	0.025477347	0.028343042	Firmicutes	Tenericutes
Cronobacter	Klebsiella	0.459467233	0.511046422	Proteobacteria	Proteobacteria
Shigella	Klebsiella	0.459467233	0.510947643	Proteobacteria	Proteobacteria
Mycoplasma	Thiomicrospira	0.025477347	0.028325265	Tenericutes	Proteobacteria
Scardovia	Actinobacteria	0.072679127	0.08063877	Actinobacteria	Actinobacteria
Dickeya	Pseudomonas	0.175575691	0.19479597	Proteobacteria	Proteobacteria
Kluyvera	Lelliottia	0.511696136	0.567436638	Proteobacteria	Proteobacteria
Mycoplasma	Fermentimonas	0.025477347	0.028246936	Tenericutes	Bacteroidetes
Gordonia	Actinobacteria	0.072679127	0.080560618	Actinobacteria	Actinobacteria
Mycoplasma	Cycloclasticus	0.025477347	0.028219316	Tenericutes	Proteobacteria
Flavobacterium	Clostridium	0.062850588	0.069597437	Bacteroidetes	Firmicutes
Thioalkalivibrio	Thiohalobacter	0.305293198	0.337564749	Proteobacteria	Proteobacteria
Chromatiaceae	Pseudomonas	0.175575691	0.194008627	Proteobacteria	Proteobacteria
Roseomonas	Pseudomonas	0.175575691	0.193654289	Proteobacteria	Proteobacteria
Mycoplasma	Aliivibrio	0.025477347	0.028088829	Tenericutes	Proteobacteria
Comamonadaceae	Pseudomonas	0.175575691	0.193567399	Proteobacteria	Proteobacteria
Kluyvera	Leclercia	0.511696136	0.563772463	Proteobacteria	Proteobacteria
Burkholderia	Ralstonia	0.330578046	0.364204367	Proteobacteria	Proteobacteria
Dechloromonas	Pseudomonas	0.175575691	0.193294563	Proteobacteria	Proteobacteria
Mycoplasma	Cellulophaga	0.025477347	0.028028581	Tenericutes	Bacteroidetes
Mycoplasma	Ruminococcus	0.025477347	0.028028443	Tenericutes	Firmicutes
Dokdonella	Pseudomonas	0.175575691	0.193051604	Proteobacteria	Proteobacteria
Frankia	Actinobacteria	0.072679127	0.079845789	Actinobacteria	Actinobacteria
Actinobacillus	Aggregatibacter	0.409596236	0.449936732	Proteobacteria	Proteobacteria
Pantoea	Pseudomonas	0.175575691	0.192646264	Proteobacteria	Proteobacteria
Murdochiella	Eubacterium	0.078449324	0.086037261	Firmicutes	Firmicutes
Mycoplasma	Halobacteriovorax	0.025477347	0.027938612	Tenericutes	Proteobacteria
Kluyvera	Citrobacter	0.511696136	0.560817093	Proteobacteria	Proteobacteria
Pasteurella	Clostridium	0.062850588	0.068850046	Proteobacteria	Firmicutes

Celeribacter	Thioclava	0.319579628	0.349997992	Proteobacteria	Proteobacteria
Mycoplasma	Psychroflexus	0.025477347	0.027899035	Tenericutes	Bacteroidetes
Parabacteroides	Bacteroides	0.250700346	0.274465019	Bacteroidetes	Bacteroidetes
Carnobacterium	Eubacterium	0.078449324	0.085884181	Firmicutes	Firmicutes
Mycoplasma	Thermotoga	0.025477347	0.027885384	Tenericutes	Thermotogae
Lactococcus	Lactobacillus	0.158222465	0.172902323	Firmicutes	Firmicutes
Bacillus	Thermoanaerobacter	0.132343801	0.144581377	Firmicutes	Firmicutes
Thermacetogenium	Desulfotomaculum	0.198423348	0.216729662	Firmicutes	Firmicutes
Paraburkholderia	Bordetella	0.232579852	0.253954515	Proteobacteria	Proteobacteria
Mycoplasma	Gardnerella	0.025477347	0.027782537	Tenericutes	Actinobacteria
Mycoplasma	Thermincola	0.025477347	0.027777562	Tenericutes	Firmicutes
Enterobacter	Enterobacteriaceae	0.557933943	0.608097907	Proteobacteria	Proteobacteria
Veillonella	Eubacterium	0.078449324	0.085477466	Firmicutes	Firmicutes
Methylibium	Betaproteobacteria	0.23303541	0.253906009	Proteobacteria	Proteobacteria
Enterobacter	Pseudomonas	0.175575691	0.191186356	Proteobacteria	Proteobacteria
Anoxybacillus	Eubacterium	0.078449324	0.085401034	Firmicutes	Firmicutes
Betaproteobacteria	Rubrivivax	0.23303541	0.253507654	Proteobacteria	Proteobacteria
Celeribacter	Dinoroseobacter	0.319579628	0.347546818	Proteobacteria	Proteobacteria
Celeribacter	Sulfitobacter	0.319579628	0.347238583	Proteobacteria	Proteobacteria
Gluconobacter	Acetobacter	0.328547737	0.356694004	Proteobacteria	Proteobacteria
Herminiimonas	Pseudomonas	0.175575691	0.190598849	Proteobacteria	Proteobacteria
Vagococcus	Eubacterium	0.078449324	0.085075291	Firmicutes	Firmicutes
Dyella	Rhodanobacter	0.451840659	0.489904719	Proteobacteria	Proteobacteria
Actinobacteria	Verrucosispora	0.072679127	0.078798379	Actinobacteria	Actinobacteria
synthetic	Shigella	0.796390611	0.863383992	Proteobacteria	Proteobacteria
Heliobacterium	Eubacterium	0.078449324	0.085029365	Firmicutes	Firmicutes
Mycoplasma	Thalassotalea	0.025477347	0.02761356	Tenericutes	Proteobacteria
Salmonella	Citrobacter	0.573559266	0.621616606	Proteobacteria	Proteobacteria
Rhizobacter	Betaproteobacteria	0.23303541	0.252521119	Proteobacteria	Proteobacteria
Shimwellia	Klebsiella	0.459467233	0.497866515	Proteobacteria	Proteobacteria
Tessaracoccus	Propionibacterium	0.255521499	0.276749404	Actinobacteria	Actinobacteria
Agrobacterium	Sinorhizobium	0.368942819	0.398602272	Proteobacteria	Proteobacteria
Frateuria	Pseudomonas	0.175575691	0.189646868	Proteobacteria	Proteobacteria
Sulfurifustis	Pseudomonas	0.175575691	0.189488767	Proteobacteria	Proteobacteria

Spirochaeta	Salinispira	0.151497491	0.163471739	Spirochaetes	Spirochaetes
Luteibacter	Pseudomonas	0.175575691	0.189381971	Proteobacteria	Proteobacteria
Bernardetia	Mycoplasma	0.025477347	0.02747873	Bacteroidetes	Tenericutes
Flavobacterium	Aequorivita	0.260303566	0.280700961	Bacteroidetes	Bacteroidetes
Mycobacterium	Pseudonocardia	0.255167927	0.275021926	Actinobacteria	Actinobacteria
Enterobacter	Kosakonia	0.557933943	0.601030206	Proteobacteria	Proteobacteria
Desulfotomaculum	Carboxydothermus	0.198423348	0.213633938	Firmicutes	Firmicutes
Lelliottia	Pseudomonas	0.175575691	0.189010863	Proteobacteria	Proteobacteria
Lutibacter	Flavobacterium	0.260303566	0.280161141	Bacteroidetes	Bacteroidetes
Citrobacter	synthetic	0.573559266	0.61715463	Proteobacteria	Proteobacteria
Syntrophobotulus	Eubacterium	0.078449324	0.084409555	Firmicutes	Firmicutes
Cedecea	Pseudomonas	0.175575691	0.188907195	Proteobacteria	Proteobacteria
Celeribacter	Rhodobacter	0.319579628	0.343784221	Proteobacteria	Proteobacteria
Ferrimonas	Pseudoalteromonas	0.161098934	0.173125041	Proteobacteria	Proteobacteria
Halomonas	Cobetia	0.348529712	0.374447673	Proteobacteria	Proteobacteria
Mycoplasma	Coprothermobacter	0.025477347	0.027362281	Tenericutes	Firmicutes
Haloterrigena	Natrinema	0.513144559	0.550958999	Euryarchaeota	Euryarchaeota
Petrotoga	Clostridium	0.062850588	0.06745518	Thermotogae	Firmicutes
Escherichia	Citrobacter	0.573559266	0.615250375	Proteobacteria	Proteobacteria
Erythrobacter	Croceicoccus	0.338583594	0.363064632	Proteobacteria	Proteobacteria
Sphingopyxis	Altererythrobacter	0.319203322	0.342164774	Proteobacteria	Proteobacteria
Sodalis	Pseudomonas	0.175575691	0.188062952	Proteobacteria	Proteobacteria
Burkholderiales	Paraburkholderia	0.092214558	0.098733609	Proteobacteria	Proteobacteria
Gordonia	Corynebacterium	0.206499705	0.220987666	Actinobacteria	Actinobacteria
Sediminispirochaeta	Treponema	0.092132037	0.098580592	Spirochaetes	Spirochaetes
Celeribacter	Rhodovulum	0.319579628	0.341753363	Proteobacteria	Proteobacteria
Histophilus	Mannheimia	0.384896686	0.411384809	Proteobacteria	Proteobacteria
Collinsella	Clostridium	0.062850588	0.06716907	Actinobacteria	Firmicutes
Plesiomonas	Pseudoalteromonas	0.161098934	0.172094515	Proteobacteria	Proteobacteria
Haloterrigena	Halopiger	0.513144559	0.548088427	Euryarchaeota	Euryarchaeota
Mycoplasma	Pseudoalteromonas	0.025477347	0.027187443	Tenericutes	Proteobacteria
Desulfuromonas	Geobacter	0.269183825	0.28721738	Proteobacteria	Proteobacteria
Jeotgalibacillus	Eubacterium	0.078449324	0.083699356	Firmicutes	Firmicutes
Celeribacter	Ruegeria	0.319579628	0.340958987	Proteobacteria	Proteobacteria

Thioflavicoccus	Pseudomonas	0.175575691	0.187246347	Proteobacteria	Proteobacteria
Bordetella	Betaproteobacteria	0.23303541	0.248432289	Proteobacteria	Proteobacteria
Alicycliphilus	Betaproteobacteria	0.23303541	0.248367273	Proteobacteria	Proteobacteria
Celeribacter	Phaeobacter	0.319579628	0.340474547	Proteobacteria	Proteobacteria
Enterobacteriaceae	Pseudomonas	0.175575691	0.187009842	Proteobacteria	Proteobacteria
Mycoplasma	Cyanobacterium	0.025477347	0.027125361	Tenericutes	Cyanobacteria
Carnobacterium	Aerococcus	0.245695723	0.261424963	Firmicutes	Firmicutes
Thermodesulfatator	Mycoplasma	0.025477347	0.027105765	Thermodesulfobacter	Tenericutes
Azoarcus	Betaproteobacteria	0.23303541	0.2478888	Proteobacteria	Proteobacteria
Mycobacterium	Hoyosella	0.255167927	0.271375475	Actinobacteria	Actinobacteria
Starkeya	Pseudomonas	0.175575691	0.18666301	Proteobacteria	Proteobacteria
Eubacterium	Melissococcus	0.078449324	0.08339707	Firmicutes	Firmicutes
Bdellovibrio	Mycoplasma	0.025477347	0.027083294	Proteobacteria	Tenericutes
Ndongobacter	Eubacterium	0.078449324	0.083378881	Firmicutes	Firmicutes
Cupriavidus	Betaproteobacteria	0.23303541	0.247421066	Proteobacteria	Proteobacteria
Clostridium	Maize	0.062850588	0.066680795	Firmicutes	Tenericutes
Hydrogenophaga	Pseudomonas	0.175575691	0.186185248	Proteobacteria	Proteobacteria
Acetomicrobium	Clostridium	0.068824019	0.072830862	Synergistetes	Firmicutes
Celeribacter	Rhodobacteraceae	0.319579628	0.338077027	Proteobacteria	Proteobacteria
Agrobacterium	Ensifer	0.368942819	0.39026163	Proteobacteria	Proteobacteria
Mycoplasma	Marivirga	0.025477347	0.02694657	Tenericutes	Bacteroidetes
Betaproteobacteria	Variovorax	0.23303541	0.246417592	Proteobacteria	Proteobacteria
Eubacterium	Jeotgalibaca	0.078449324	0.08294845	Firmicutes	Firmicutes
Celeribacter	Paracoccus	0.319579628	0.337709494	Proteobacteria	Proteobacteria
Acidihalobacter	Pseudomonas	0.175575691	0.185525192	Proteobacteria	Proteobacteria
Macrococcus	Lactobacillus	0.158222465	0.167090178	Firmicutes	Firmicutes
Clostridium	Vibrio	0.062850588	0.066347885	Firmicutes	Proteobacteria
Actinobacteria	Kribbella	0.072679127	0.076721216	Actinobacteria	Actinobacteria
Ralstonia	Betaproteobacteria	0.23303541	0.245837146	Proteobacteria	Proteobacteria
Pasteurella	Actinobacillus	0.409596236	0.431890314	Proteobacteria	Proteobacteria
Shimwellia	Pseudomonas	0.175575691	0.185053042	Proteobacteria	Proteobacteria
Francisella	Clostridium	0.062850588	0.066212949	Proteobacteria	Firmicutes
Sulfuricaulis	Pseudomonas	0.175575691	0.184922604	Proteobacteria	Proteobacteria
Citrobacter	Enterobacter	0.557933943	0.586974347	Proteobacteria	Proteobacteria

Azorhizobium	Pseudomonas	0.175575691	0.184593712	Proteobacteria	Proteobacteria
Micromonospora	Actinobacteria	0.072679127	0.076410742	Actinobacteria	Actinobacteria
Pandoraea	Ralstonia	0.330578046	0.347499442	Proteobacteria	Proteobacteria
Proteus	Mycoplasma	0.025477347	0.026778732	Proteobacteria	Tenericutes
Lachnoclostridium	Intestinimonas	0.132857063	0.139325883	Firmicutes	Firmicutes
Xanthomonas	Lysobacter	0.330541251	0.346241902	Proteobacteria	Proteobacteria
Methylococcus	Pseudomonas	0.175575691	0.18379546	Proteobacteria	Proteobacteria
Methanocaldococcus	Methanotorris	0.493039083	0.515428455	Euryarchaeota	Euryarchaeota
Auricoccus	Lactobacillus	0.158222465	0.165034766	Firmicutes	Firmicutes
Hydrogenovibrio	Mycoplasma	0.025477347	0.026570482	Proteobacteria	Tenericutes
Macrococcus	Eubacterium	0.078449324	0.081812282	Firmicutes	Firmicutes
Moorella	Eubacterium	0.078449324	0.081802868	Firmicutes	Firmicutes
Nitrospirillum	Pseudomonas	0.175575691	0.182896767	Proteobacteria	Proteobacteria
Acidipropionibacteriur	Corynebacterium	0.177688971	0.184934584	Actinobacteria	Actinobacteria
Wenyingzhuangia	Clostridium	0.063423572	0.065909903	Bacteroidetes	Firmicutes
Flavobacterium	Cellulophaga	0.263900411	0.274107449	Bacteroidetes	Bacteroidetes
Halioglobus	Pseudomonas	0.175575691	0.182350801	Proteobacteria	Proteobacteria
Saccharopolyspora	Mycobacterium	0.255167927	0.264934814	Actinobacteria	Actinobacteria
Desulfitobacterium	Desulfosporosinus	0.292685201	0.303714576	Firmicutes	Firmicutes
Celeribacter	Tateyamaria	0.319579628	0.331552123	Proteobacteria	Proteobacteria
Bacillus	Eubacterium	0.078449324	0.081377207	Firmicutes	Firmicutes
Enterobacter	Raoultella	0.557933943	0.578742489	Proteobacteria	Proteobacteria
Polaribacter	Tenacibaculum	0.353544316	0.366727674	Bacteroidetes	Bacteroidetes
Mycoplasma	Actinobacteria	0.025477347	0.026422418	Tenericutes	Actinobacteria
Histophilus	Clostridium	0.062850588	0.065174358	Proteobacteria	Firmicutes
Marinovum	Ruegeria	0.394096176	0.408597488	Proteobacteria	Proteobacteria
Crinalium	Oscillatoria	0.30678895	0.317950946	Cyanobacteria	Cyanobacteria
Eubacterium	Mageeibacillus	0.078449324	0.081280011	Firmicutes	Firmicutes
Kozakia	Acetobacter	0.328547737	0.34004339	Proteobacteria	Proteobacteria
Bordetella	Castellaniella	0.361544995	0.374144876	Proteobacteria	Proteobacteria
Citrobacter	Shigella	0.573559266	0.593244969	Proteobacteria	Proteobacteria
Kluyvera	Escherichia	0.511696136	0.529169754	Proteobacteria	Proteobacteria
Thioclava	Rhodobacter	0.394282391	0.407538709	Proteobacteria	Proteobacteria
Amphibacillus	Lactobacillus	0.158222465	0.163469721	Firmicutes	Firmicutes

Burkholderiales	Bordetella	0.092214558	0.095269727	Proteobacteria	Proteobacteria
Cronobacter	Enterobacter	0.557933943	0.575356913	Proteobacteria	Proteobacteria
Achromobacter	Betaproteobacteria	0.23303541	0.240165923	Proteobacteria	Proteobacteria
Actinobacteria	Nakamurella	0.072679127	0.074881259	Actinobacteria	Actinobacteria
Brucella	Ochrobactrum	0.552844089	0.569223732	Proteobacteria	Proteobacteria
Bacillus	Melissococcus	0.132343801	0.136213407	Firmicutes	Firmicutes
Corynebacterium	Propionibacterium	0.177688971	0.182834915	Actinobacteria	Actinobacteria
Mycobacterium	Pimelobacter	0.255167927	0.262501746	Actinobacteria	Actinobacteria
Neoasaia	Acetobacter	0.328547737	0.33778714	Proteobacteria	Proteobacteria
Acidovorax	Betaproteobacteria	0.23303541	0.239506936	Proteobacteria	Proteobacteria
Kluyvera	Pseudomonas	0.175575691	0.180412044	Proteobacteria	Proteobacteria
Actinomyces	Micrococcus	0.249688528	0.256468283	Actinobacteria	Actinobacteria
Novosphingobium	Erythrobacter	0.338583594	0.347487489	Proteobacteria	Proteobacteria
Rickettsiales	Clostridium	0.063423572	0.065088596	Proteobacteria	Firmicutes
Aster	Clostridium	0.062850588	0.064482288	Tenericutes	Firmicutes
Enterococcus	Eubacterium	0.078449324	0.080479475	Firmicutes	Firmicutes
Alicycliphilus	Paraburkholderia	0.232579852	0.238533131	Proteobacteria	Proteobacteria
Saccharomonospora	Brevibacterium	0.125961995	0.128998406	Actinobacteria	Actinobacteria
Celeribacter	Leisingera	0.319579628	0.327228644	Proteobacteria	Proteobacteria
Cronobacter	Pantoea	0.477738095	0.48895972	Proteobacteria	Proteobacteria
Kluyvera	Salmonella	0.511696136	0.523631516	Proteobacteria	Proteobacteria
Tetragenococcus	Eubacterium	0.078449324	0.080268376	Firmicutes	Firmicutes
Kluyvera	synthetic	0.511696136	0.523403802	Proteobacteria	Proteobacteria
Syntrophobotulus	Mycoplasma	0.025477347	0.026044505	Firmicutes	Tenericutes
Nocardia	Actinobacteria	0.072679127	0.074256895	Actinobacteria	Actinobacteria
Staphylococcus	Eubacterium	0.078449324	0.080079628	Firmicutes	Firmicutes
Blattabacterium	Flavobacteriaceae	0.161380659	0.164732826	Bacteroidetes	Bacteroidetes
Eubacterium	Carboxydothermus	0.078449324	0.079987629	Firmicutes	Firmicutes
Celeribacter	Loktanella	0.319579628	0.325839429	Proteobacteria	Proteobacteria
Rhodobacteraceae	Rhodobacter	0.394282391	0.401883596	Proteobacteria	Proteobacteria
Syntrophomonas	Mycoplasma	0.025477347	0.025960223	Firmicutes	Tenericutes
Mycoplasma	Providencia	0.025477347	0.025954335	Tenericutes	Proteobacteria
Herbaspirillum	Betaproteobacteria	0.23303541	0.237384532	Proteobacteria	Proteobacteria
Clostridium	Lacinutrix	0.068824019	0.070086003	Firmicutes	Bacteroidetes

Kyrpidia	Clostridium	0.062850588	0.063979041	Firmicutes	Firmicutes
Staphylococcus	Macrococcus	0.379265117	0.386011914	Firmicutes	Firmicutes
Aeromicrobium	Brevibacterium	0.125961995	0.128193676	Actinobacteria	Actinobacteria
Ilyobacter	Eubacterium	0.088134527	0.089653548	Fusobacteria	Firmicutes
Tannerella	Porphyromonas	0.221955156	0.225775088	Bacteroidetes	Bacteroidetes
Corynebacterium	Glutamicibacter	0.177688971	0.180652675	Actinobacteria	Actinobacteria
Weeksella	Clostridium	0.062850588	0.063869077	Bacteroidetes	Firmicutes
Actinoalloteichus	Actinobacteria	0.072679127	0.073776297	Actinobacteria	Actinobacteria
Pseudomonas	Spiribacter	0.175575691	0.178182682	Proteobacteria	Proteobacteria
Xanthobacter	Pseudomonas	0.175575691	0.178151827	Proteobacteria	Proteobacteria
Bacillus	Kyrpidia	0.132343801	0.134258694	Firmicutes	Firmicutes
Algibacter	Flavobacterium	0.260303566	0.26381399	Bacteroidetes	Bacteroidetes
Rahnella	Pseudomonas	0.175575691	0.177830332	Proteobacteria	Proteobacteria
Prauserella	Actinobacteria	0.072679127	0.07359407	Actinobacteria	Actinobacteria
Magnetospirillum	Rhodospirillum	0.265465699	0.268658083	Proteobacteria	Proteobacteria
Flavobacterium	Myroides	0.260303566	0.263246422	Bacteroidetes	Bacteroidetes
Polaribacter	Flavobacterium	0.263900411	0.26679763	Bacteroidetes	Bacteroidetes
Clostridium	Mannheimia	0.062850588	0.063525919	Firmicutes	Proteobacteria
Celeribacter	Antarctobacter	0.319579628	0.32290219	Proteobacteria	Proteobacteria
Methylobacillus	Pseudomonas	0.175575691	0.176983571	Proteobacteria	Proteobacteria
Flavobacterium	Dokdonia	0.263900411	0.266002513	Bacteroidetes	Bacteroidetes
Hahella	Pseudomonas	0.175575691	0.176894211	Proteobacteria	Proteobacteria
Actinobacteria	Parascardovia	0.072679127	0.073182232	Actinobacteria	Actinobacteria
Kluyvera	Cronobacter	0.511696136	0.514529795	Proteobacteria	Proteobacteria
Mycoplasma	Megasphaera	0.025477347	0.025616279	Tenericutes	Firmicutes
Sulfuritalea	Betaproteobacteria	0.23303541	0.234149246	Proteobacteria	Proteobacteria
Enterobacter	synthetic	0.557933943	0.560574234	Proteobacteria	Proteobacteria
Desulfovibrio	Desulfarculus	0.143678238	0.144253227	Proteobacteria	Proteobacteria
Cellvibrio	Pseudomonas	0.175575691	0.176173081	Proteobacteria	Proteobacteria
Mycoplasma	Psychromonas	0.025477347	0.025544448	Tenericutes	Proteobacteria
Steroidobacter	Pseudomonas	0.175575691	0.175937487	Proteobacteria	Proteobacteria
Salmonella	Enterobacter	0.557933943	0.559031393	Proteobacteria	Proteobacteria
Amycolatopsis	Actinobacteria	0.072679127	0.072801349	Actinobacteria	Actinobacteria
Phaeobacter	Leisingera	0.513279234	0.514045664	Proteobacteria	Proteobacteria
