# Supplementary material for: A New Genome-to-Genome Comparison Approach for Large-Scale Revisiting of Current Microbial Taxonomy
Source: Microorganisms. 2019 Jun 3;7(6):161. doi: 10.3390/microorganisms7060161 (PMC6616886; doi:10.3390/microorganisms7060161)
Supplement: Supplementary file 1 [file microorganisms-07-00161-s001.zip › supplementary_files/Supplemental_TableS3.pdf]

Table S3. 2227 microbial genomes differ more than 2-fold between interphylum and intraphylum HCR.

No.	File name (assembly accession)	Genus	Phylum
1	GCA_002276165.1_ASM227616v1_genomic.fna	Bacillus	Firmicutes
2	GCA_900186985.1_50377_C01_genomic.fna	Staphylococcus	Firmicutes
3	GCA_900187285.1_51342_C02_genomic.fna	Veillonella	Firmicutes
4	GCA_900187075.1_50432_F01_genomic.fna	Staphylococcus	Firmicutes
5	GCA_000759555.1_ASM75955v1_genomic.fna	Staphylococcus	Firmicutes
6	GCA_000025045.1_ASM2504v1_genomic.fna	Lactococcus	Firmicutes
7	GCA_002208945.1_ASM220894v1_genomic.fna	Enterococcus	Firmicutes
8	GCA_000009645.1_ASM964v1_genomic.fna	Staphylococcus	Firmicutes
9	GCA_001593785.1_ASM159378v1_genomic.fna	Bacillus	Firmicutes
10	GCA_000015525.1_ASM1552v1_genomic.fna	Campylobacter	Proteobacteria
11	GCA_001886635.1_ASM188663v1_genomic.fna	Enterococcus	Firmicutes
12	GCA_001865535.1_ASM186553v1_genomic.fna	Campylobacter	Proteobacteria
13	GCA_000017485.1_ASM1748v1_genomic.fna	Campylobacter	Proteobacteria
14	GCA_001272835.1_ASM127283v1_genomic.fna	Mycoplasma	Tenericutes
15	GCA_000009665.1_ASM966v1_genomic.fna	Staphylococcus	Firmicutes
16	GCA_000143205.1_ASM14320v1_genomic.fna	Lactococcus	Firmicutes
17	GCA_001956655.2_ASM195665v2_genomic.fna	Staphylococcus	Firmicutes
18	GCA_000021205.1_ASM2120v1_genomic.fna	Bacillus	Firmicutes
19	GCA_001596095.1_ASM159609v1_genomic.fna	Lactobacillus	Firmicutes
20	GCA_001579765.1_ASM157976v1_genomic.fna	Clostridium	Firmicutes
21	GCA_002220615.1_ASM222061v1_genomic.fna	Serratia	Proteobacteria
22	GCA_000026705.1_ASM2670v1_genomic.fna	Listeria	Firmicutes
23	GCA_000017965.1_ASM1796v1_genomic.fna	Lysinibacillus	Firmicutes
24	GCA_000011285.1_ASM1128v1_genomic.fna	Streptococcus	Firmicutes
25	GCA_000018985.1_ASM1898v1_genomic.fna	Streptococcus	Firmicutes
26	GCA_001865555.1_ASM186555v1_genomic.fna	Campylobacter	Proteobacteria
27	GCA_002355215.1_ASM235521v1_genomic.fna	Streptococcus	Firmicutes
28	GCA_000012645.1_ASM1264v1_genomic.fna	Prochlorococcus	Cyanobacteria
29	GCA_001990205.1_ASM199020v1_genomic.fna	Staphylococcus	Firmicutes
30	GCA_001023595.1_ASM102359v1_genomic.fna	Bacillus	Firmicutes
31	GCA_000090965.1_ASM9096v1_genomic.fna	Buchnera	Proteobacteria

32	GCA_001536925.1_BMC_PRJEB12378v1_genomic.fna	Bacillus	Firmicutes
33	GCA_000400855.1_ASM40085v1_genomic.fna	Mycoplasma	Tenericutes
34	GCA_000168355.3_ASM16835v3_genomic.fna	Streptococcus	Firmicutes
35	GCA_000017425.1_ASM1742v1_genomic.fna	Bacillus	Firmicutes
36	GCA_002442895.1_ASM244289v1_genomic.fna	Staphylococcus	Firmicutes
37	GCA_002441935.1_ASM244193v1_genomic.fna	Mesoplasma	Tenericutes
38	GCA_000024945.1_ASM2494v1_genomic.fna	Veillonella	Firmicutes
39	GCA_002369955.1_ASM236995v1_genomic.fna	Ichthyobacterium	Bacteroidetes
40	GCA_000178835.2_ASM17883v2_genomic.fna	Clostridium	Firmicutes
41	GCA_000092505.1_ASM9250v1_genomic.fna	Leuconostoc	Firmicutes
42	GCA_000400875.1_ASM40087v1_genomic.fna	Fusobacterium	Fusobacteria
43	GCA_002220635.1_ASM222063v1_genomic.fna	Serratia	Proteobacteria
44	GCA_000022865.1_ASM2286v1_genomic.fna	Bacillus	Firmicutes
45	GCA_001617525.1_ASM161752v1_genomic.fna	Lactobacillus	Firmicutes
46	GCA_001272855.1_ASM127285v1_genomic.fna	Mycoplasma	Tenericutes
47	GCA_000410995.1_ASM41099v1_genomic.fna	Lactobacillus	Firmicutes
48	GCA_001191605.1_ASM119160v1_genomic.fna	Bacillus	Firmicutes
49	GCA_000191545.1_ASM19154v1_genomic.fna	Lactobacillus	Firmicutes
50	GCA_002442935.1_ASM244293v1_genomic.fna	Staphylococcus	Firmicutes
51	GCA_000196215.1_ASM19621v1_genomic.fna	Borreliella	Spirochaetes
52	GCA_000302475.3_ASM30247v3_genomic.fna	Streptococcus	Firmicutes
53	GCA_002074075.1_ASM207407v1_genomic.fna	Bacillus	Firmicutes
54	GCA_000182875.1_ASM18287v1_genomic.fna	Streptococcus	Firmicutes
55	GCA_000190515.1_ASM19051v1_genomic.fna	Bacillus	Firmicutes
56	GCA_000448685.2_ASM44868v2_genomic.fna	Streptococcus	Firmicutes
57	GCA_000166415.1_ASM16641v1_genomic.fna	Halanaerobium	Firmicutes
58	GCA_002080475.1_ASM208047v1_genomic.fna	Clostridium	Firmicutes
59	GCA_900187165.1_52121_G02_genomic.fna	Clostridium	Firmicutes
60	GCA_001936295.1_ASM193629v1_genomic.fna	Borrelia	Spirochaetes
61	GCA_000463395.1_ASM46339v1_genomic.fna	Streptococcus	Firmicutes
62	GCA_002021945.1_ASM202194v1_genomic.fna	Campylobacter	Proteobacteria
63	GCA_000025085.1_ASM2508v1_genomic.fna	Staphylococcus	Firmicutes
64	GCA_000147695.3_ASM14769v3_genomic.fna	Thermoanaerobacte	Firmicutes
65	GCA_002355135.1_ASM235513v1_genomic.fna	Blattabacterium	Bacteroidetes

66	GCA_000292505.1_ASM29250v1_genomic.fna	Mycoplasma	Tenericutes
67	GCA_000724775.3_ASM72477v3_genomic.fna	Lysinibacillus	Firmicutes
68	GCA_000225325.1_ASM22532v1_genomic.fna	Lactobacillus	Firmicutes
69	GCA_001687625.2_ASM168762v2_genomic.fna	Planococcus	Firmicutes
70	GCA_000179035.2_ASM17903v2_genomic.fna	Mycoplasma	Tenericutes
71	GCA_002290025.1_ASM229002v1_genomic.fna	Enterococcus	Firmicutes
72	GCA_001663655.1_ASM166365v1_genomic.fna	Lactobacillus	Firmicutes
73	GCA_000463505.1_ASM46350v1_genomic.fna	Streptococcus	Firmicutes
74	GCA_000014605.1_ASM1460v1_genomic.fna	Francisella	Proteobacteria
75	GCA_000224105.1_ASM22410v1_genomic.fna	Mycoplasma	Tenericutes
76	GCA_000876525.1_ASM87652v1_genomic.fna	Bacillus	Firmicutes
77	GCA_002443095.1_ASM244309v1_genomic.fna	Bacillus	Firmicutes
78	GCA_000010985.1_ASM1098v1_genomic.fna	Deferribacter	Deferribacteres
79	GCA_001465115.1_ASM146511v1_genomic.fna	Enterococcus	Firmicutes
80	GCA_002000985.1_ASM200098v1_genomic.fna	Streptococcus	Firmicutes
81	GCA_001188875.1_ASM118887v1_genomic.fna	Staphylococcus	Firmicutes
82	GCA_001513715.1_ASM151371v1_genomic.fna	Spiroplasma	Tenericutes
83	GCA_001702115.1_ASM170211v1_genomic.fna	Lactobacillus	Firmicutes
84	GCA_001702155.1_ASM170215v1_genomic.fna	Lactobacillus	Firmicutes
85	GCA_000164675.2_ASM16467v2_genomic.fna	Streptococcus	Firmicutes
86	GCA_000325665.1_ASM32566v1_genomic.fna	Brachyspira	Spirochaetes
87	GCA_002082765.1_ASM208276v1_genomic.fna	Veillonella	Firmicutes
88	GCA_001543175.1_ASM154317v1_genomic.fna	Aerococcus	Firmicutes
89	GCA_000189775.3_ASM18977v3_genomic.fna	Thermoanaerobacte	Firmicutes
90	GCA_002633765.1_ASM263376v1_genomic.fna	Staphylococcus	Firmicutes
91	GCA_002633785.1_ASM263378v1_genomic.fna	Staphylococcus	Firmicutes
92	GCA_002633825.1_ASM263382v1_genomic.fna	Staphylococcus	Firmicutes
93	GCA_002633805.1_ASM263380v1_genomic.fna	Staphylococcus	Firmicutes
94	GCA_900017775.1_NZAK3_genomic.fna	Staphylococcus	Firmicutes
95	GCA_900004855.1_BB155_genomic.fna	Staphylococcus	Firmicutes
96	GCA_002633845.1_ASM263384v1_genomic.fna	Staphylococcus	Firmicutes
97	GCA_002633865.1_ASM263386v1_genomic.fna	Staphylococcus	Firmicutes
98	GCA_900092595.1_Sa_Newman_UoM_genomic.fna	Staphylococcus	Firmicutes
99	GCA_900096745.1_AUS0325_genomic.fna	Staphylococcus	Firmicutes

100	GCA_000009085.1_ASM908v1_genomic.fna	Campylobacter	Proteobacteria
101	GCA_000013425.1_ASM1342v1_genomic.fna	Staphylococcus	Firmicutes
102	GCA_000007645.1_ASM764v1_genomic.fna	Staphylococcus	Firmicutes
103	GCA_000006865.1_ASM686v1_genomic.fna	Lactococcus	Firmicutes
104	GCA_000007825.1_ASM782v1_genomic.fna	Bacillus	Firmicutes
105	GCA_000013285.1_ASM1328v1_genomic.fna	Clostridium	Firmicutes
106	GCA_900129335.1_PP_HGAG_QV30_2SC_T4_genomic.fna	Staphylococcus	Firmicutes
107	GCA_000196035.1_ASM19603v1_genomic.fna	Listeria	Firmicutes
108	GCA_000007925.1_ASM792v1_genomic.fna	Prochlorococcus	Cyanobacteria
109	GCA_000007365.1_ASM736v1_genomic.fna	Buchnera	Proteobacteria
110	GCA_000006785.2_ASM678v2_genomic.fna	Streptococcus	Firmicutes
111	GCA_000007045.1_ASM704v1_genomic.fna	Streptococcus	Firmicutes
112	GCA_000007325.1_ASM732v1_genomic.fna	Fusobacterium	Fusobacteria
113	GCA_000007845.1_ASM784v1_genomic.fna	Bacillus	Firmicutes
114	GCA_000007265.1_ASM726v1_genomic.fna	Streptococcus	Firmicutes
115	GCA_000816945.1_ASM81694v1_genomic.fna	Clostridium	Firmicutes
116	GCA_000759485.1_ASM75948v1_genomic.fna	Campylobacter	Proteobacteria
117	GCA_000953275.1_CD630DERM_genomic.fna	Clostridioides	Firmicutes
118	GCA_000019885.1_ASM1988v1_genomic.fna	Coxiella	Proteobacteria
119	GCA_000309565.2_ASM30956v2_genomic.fna	Lactobacillus	Firmicutes
120	GCA_001469775.1_ASM146977v1_genomic.fna	Lactobacillus	Firmicutes
121	GCA_000953235.1_ILRI181.1_genomic.fna	Mycoplasma	Tenericutes
122	GCA_001742205.1_ASM174220v1_genomic.fna	Lactobacillus	Firmicutes
123	GCA_000253075.1_ASM25307v1_genomic.fna	Mycoplasma	Tenericutes
124	GCA_000227465.1_ASM22746v1_genomic.fna	Bacillus	Firmicutes
125	GCA_000283815.1_ASM28381v1_genomic.fna	Rickettsia	Proteobacteria
126	GCA_000422165.1_ASM42216v1_genomic.fna	Lactobacillus	Firmicutes
127	GCA_000281195.1_ASM28119v1_genomic.fna	Enterococcus	Firmicutes
128	GCA_000277205.1_ASM27720v1_genomic.fna	Rickettsia	Proteobacteria
129	GCA_000017025.1_ASM1702v1_genomic.fna	Clostridium	Firmicutes
130	GCA_000444465.1_ASM44446v1_genomic.fna	Borrelia	Spirochaetes
131	GCA_000737555.1_ASM73755v1_genomic.fna	Enterococcus	Firmicutes
132	GCA_002104575.1_ASM210457v1_genomic.fna	Bacillus	Firmicutes
133	GCA_001922405.1_ASM192240v1_genomic.fna	Staphylococcus	Firmicutes

134	GCA_000242855.2_ASM24285v2_genomic.fna	Bacillus	Firmicutes
135	GCA_001483985.1_ASM148398v1_genomic.fna	Campylobacter	Proteobacteria
136	GCA_000817065.1_ASM81706v1_genomic.fna	Streptococcus	Firmicutes
137	GCA_000418475.1_ASM41847v1_genomic.fna	Lactobacillus	Firmicutes
138	GCA_001991075.1_ASM199107v1_genomic.fna	Clostridium	Firmicutes
139	GCA_001455105.1_ASM145510v1_genomic.fna	Fusobacterium	Fusobacteria
140	GCA_000696505.1_ASM69650v1_genomic.fna	Streptococcus	Firmicutes
141	GCA_000833295.1_ASM83329v1_genomic.fna	Francisella	Proteobacteria
142	GCA_000319675.2_ASM31967v2_genomic.fna	Mycoplasma	Tenericutes
143	GCA_000829335.1_ASM82933v1_genomic.fna	Mycoplasma	Tenericutes
144	GCA_000225265.1_ASM22526v1_genomic.fna	Bacillus	Firmicutes
145	GCA_002386285.1_ASM238628v1_genomic.fna	Streptococcus	Firmicutes
146	GCA_000283695.1_ASM28369v1_genomic.fna	Bacillus	Firmicutes
147	GCA_002117325.1_ASM211732v1_genomic.fna	Lactobacillus	Firmicutes
148	GCA_001022215.1_ASM102221v1_genomic.fna	Serratia	Proteobacteria
149	GCA_002327185.1_ASM232718v1_genomic.fna	Clostridium	Firmicutes
150	GCA_000816305.1_ASM81630v1_genomic.fna	Campylobacter	Proteobacteria
151	GCA_000085865.1_ASM8586v1_genomic.fna	Mycoplasma	Tenericutes
152	GCA_000024105.1_ASM2410v1_genomic.fna	Anaerococcus	Firmicutes
153	GCA_000191045.1_ASM19104v1_genomic.fna	Desulfurobacterium	Aquificae
154	GCA_001583825.1_ASM158382v1_genomic.fna	Leuconostoc	Firmicutes
155	GCA_000392485.2_ASM39248v2_genomic.fna	Lactobacillus	Firmicutes
156	GCA_002286215.1_ASM228621v1_genomic.fna	Lactobacillus	Firmicutes
157	GCA_001559115.1_ASM155911v1_genomic.fna	Staphylococcus	Firmicutes
158	GCA_002202155.1_ASM220215v1_genomic.fna	Pediococcus	Firmicutes
159	GCA_002003345.1_ASM200334v1_genomic.fna	Clostridium	Firmicutes
160	GCA_001723525.1_ASM172352v1_genomic.fna	Lactobacillus	Firmicutes
161	GCA_001514455.1_ASM151445v1_genomic.fna	Serratia	Proteobacteria
162	GCA_001698145.1_ASM169814v1_genomic.fna	Leuconostoc	Firmicutes
163	GCA_002290085.1_ASM229008v1_genomic.fna	Mesoplasma	Tenericutes
164	GCA_000213825.1_ASM21382v1_genomic.fna	Streptococcus	Firmicutes
165	GCA_001484725.1_ASM148472v1_genomic.fna	Clostridium	Firmicutes
166	GCA_002003285.1_ASM200328v1_genomic.fna	Clostridium	Firmicutes
167	GCA_000286675.1_ASM28667v1_genomic.fna	Mycoplasma	Tenericutes

168	GCA_000583795.1_ASM58379v1_genomic.fna	Campylobacter	Proteobacteria
169	GCA_001043135.1_ASM104313v1_genomic.fna	Mycoplasma	Tenericutes
170	GCA_000816405.1_ASM81640v1_genomic.fna	Campylobacter	Proteobacteria
171	GCA_001039495.1_ASM103949v1_genomic.fna	Bacillus	Firmicutes
172	GCA_001308145.2_ASM130814v2_genomic.fna	Weissella	Firmicutes
173	GCA_002442915.1_ASM244291v1_genomic.fna	Staphylococcus	Firmicutes
174	GCA_001543205.1_ASM154320v1_genomic.fna	Aerococcus	Firmicutes
175	GCA_000807295.1_ASM80729v1_genomic.fna	Borrelia	Spirochaetes
176	GCA_000221985.1_ASM22198v1_genomic.fna	Streptococcus	Firmicutes
177	GCA_000385925.1_ASM38592v1_genomic.fna	Streptococcus	Firmicutes
178	GCA_002250055.1_ASM225005v1_genomic.fna	Bacillus	Firmicutes
179	GCA_000763515.1_ASM76351v1_genomic.fna	Listeria	Firmicutes
180	GCA_000024565.1_ASM2456v1_genomic.fna	Streptobacillus	Fusobacteria
181	GCA_000975245.1_ASM97524v1_genomic.fna	Serratia	Proteobacteria
182	GCA_000196855.1_ASM19685v1_genomic.fna	Leuconostoc	Firmicutes
183	GCA_002381265.1_ASM238126v1_genomic.fna	Brochothrix	Firmicutes
184	GCA_000732905.1_ASM73290v1_genomic.fna	Weissella	Firmicutes
185	GCA_001886875.1_ASM188687v1_genomic.fna	Clostridium	Firmicutes
186	GCA_001714745.1_ASM171474v1_genomic.fna	Lactobacillus	Firmicutes
187	GCA_000092845.1_ASM9284v1_genomic.fna	Brachyspira	Spirochaetes
188	GCA_000185885.1_ASM18588v1_genomic.fna	Staphylococcus	Firmicutes
189	GCA_000023905.1_ASM2390v1_genomic.fna	Leptotrichia	Fusobacteria
190	GCA_001687665.2_ASM168766v2_genomic.fna	Planococcus	Firmicutes
191	GCA_000008205.1_ASM820v1_genomic.fna	Mycoplasma	Tenericutes
192	GCA_000026745.1_ASM2674v1_genomic.fna	Streptococcus	Firmicutes
193	GCA_000011645.1_ASM1164v1_genomic.fna	Bacillus	Firmicutes
194	GCA_000253395.1_ASM25339v1_genomic.fna	Streptococcus	Firmicutes
195	GCA_000016825.1_ASM1682v1_genomic.fna	Lactobacillus	Firmicutes
196	GCA_001578205.1_ASM157820v1_genomic.fna	Bacillus	Firmicutes
197	GCA_000027325.1_ASM2732v1_genomic.fna	Mycoplasma	Tenericutes
198	GCA_000008505.1_ASM850v1_genomic.fna	Bacillus	Firmicutes
199	GCA_000785515.1_ASM78551v1_genomic.fna	Streptococcus	Firmicutes
200	GCA_000008985.1_ASM898v1_genomic.fna	Francisella	Proteobacteria
201	GCA_000056065.1_ASM5606v1_genomic.fna	Lactobacillus	Firmicutes

202	GCA_000063605.1_ASM6360v1_genomic.fna	Mycoplasma	Tenericutes
203	GCA_000012765.1_ASM1276v1_genomic.fna	Mycoplasma	Tenericutes
204	GCA_000495505.1_ASM49550v1_genomic.fna	Campylobacter	Proteobacteria
205	GCA_000027165.1_ASM2716v1_genomic.fna	Streptococcus	Firmicutes
206	GCA_000512775.1_ASM51277v1_genomic.fna	Bacillus	Firmicutes
207	GCA_000009205.1_ASM920v1_genomic.fna	Clostridioides	Firmicutes
208	GCA_000014385.1_ASM1438v1_genomic.fna	Oenococcus	Firmicutes
209	GCA_000007765.2_ASM776v2_genomic.fna	Coxiella	Proteobacteria
210	GCA_000747375.1_ASM74737v1_genomic.fna	Bacillus	Firmicutes
211	GCA_002128265.1_ASM212826v1_genomic.fna	Mycoplasma	Tenericutes
212	GCA_001889385.1_ASM188938v1_genomic.fna	Bacillus	Firmicutes
213	GCA_002208785.1_ASM220878v1_genomic.fna	Bacillus	Firmicutes
214	GCA_000019245.3_ASM1924v3_genomic.fna	Lactobacillus	Firmicutes
215	GCA_000012385.1_ASM1238v1_genomic.fna	Rickettsia	Proteobacteria
216	GCA_000009045.1_ASM904v1_genomic.fna	Bacillus	Firmicutes
217	GCA_001308285.1_ASM130828v1_genomic.fna	Lactobacillus	Firmicutes
218	GCA_000017445.3_ASM1744v3_genomic.fna	Rickettsia	Proteobacteria
219	GCA_000747335.1_ASM74733v1_genomic.fna	Bacillus	Firmicutes
220	GCA_001635895.1_ASM163589v1_genomic.fna	Streptococcus	Firmicutes
221	GCA_002277915.1_ASM227791v1_genomic.fna	Bacillus	Firmicutes
222	GCA_000304735.1_ASM30473v1_genomic.fna	Borrelia	Spirochaetes
223	GCA_000269925.1_ASM26992v1_genomic.fna	Lactococcus	Firmicutes
224	GCA_000597965.1_ASM59796v1_genomic.fna	Staphylococcus	Firmicutes
225	GCA_000626615.1_ASM62661v1_genomic.fna	Staphylococcus	Firmicutes
226	GCA_000582845.1_SV_1_genomic.fna	Listeria	Firmicutes
227	GCA_001506345.1_ASM150634v1_genomic.fna	Campylobacter	Proteobacteria
228	GCA_000010145.1_ASM1014v1_genomic.fna	Lactobacillus	Firmicutes
229	GCA_000016505.1_ASM1650v1_genomic.fna	Clostridium	Firmicutes
230	GCA_000011445.1_ASM1144v1_genomic.fna	Mycoplasma	Tenericutes
231	GCA_002290105.1_ASM229010v1_genomic.fna	Bacillus	Firmicutes
232	GCA_000577745.1_6179_18022011_genomic.fna	Listeria	Firmicutes
233	GCA_001506305.1_ASM150630v1_genomic.fna	Campylobacter	Proteobacteria
234	GCA_002356575.1_ASM235657v1_genomic.fna	Bacillus	Firmicutes
235	GCA_001889625.1_ASM188962v1_genomic.fna	Bacillus	Firmicutes

236	GCA_000017045.1_ASM1704v1_genomic.fna	Clostridium	Firmicutes
237	GCA_001635935.1_ASM163593v1_genomic.fna	Streptococcus	Firmicutes
238	GCA_000195735.1_ASM19573v1_genomic.fna	Rickettsia	Proteobacteria
239	GCA_000008685.2_ASM868v2_genomic.fna	Borrelia	Spirochaetes
240	GCA_002147855.1_ASM214785v1_genomic.fna	Mycoplasma	Tenericutes
241	GCA_001890405.1_ASM189040v1_genomic.fna	Bacillus	Firmicutes
242	GCA_002128285.1_ASM212828v1_genomic.fna	Mycoplasma	Tenericutes
243	GCA_001635975.1_ASM163597v1_genomic.fna	Streptococcus	Firmicutes
244	GCA_000600015.1_ASM60001v1_genomic.fna	Listeria	Firmicutes
245	GCA_000695875.1_ASM69587v1_genomic.fna	Staphylococcus	Firmicutes
246	GCA_001506385.1_ASM150638v1_genomic.fna	Campylobacter	Proteobacteria
247	GCA_000613085.1_R479A_18022011_genomic.fna	Listeria	Firmicutes
248	GCA_000695215.1_ASM69521v1_genomic.fna	Staphylococcus	Firmicutes
249	GCA_001506365.1_ASM150636v1_genomic.fna	Campylobacter	Proteobacteria
250	GCA_001902555.1_ASM190255v1_genomic.fna	Bacillus	Firmicutes
251	GCA_001638845.2_ASM163884v2_genomic.fna	Streptococcus	Firmicutes
252	GCA_002055965.1_ASM205596v1_genomic.fna	Bacillus	Firmicutes
253	GCA_002355695.1_ASM235569v1_genomic.fna	Mycoplasma	Tenericutes
254	GCA_002355715.1_ASM235571v1_genomic.fna	Mycoplasma	Tenericutes
255	GCA_900016775.1_STsPazieg1.0_genomic.fna	Serratia	Proteobacteria
256	GCA_002356055.1_ASM235605v1_genomic.fna	Streptococcus	Firmicutes
257	GCA_000496815.1_ASM49681v1_genomic.fna	Mycoplasma	Tenericutes
258	GCA_001191565.1_ASM119156v1_genomic.fna	Lactobacillus	Firmicutes
259	GCA_002370375.1_ASM237037v1_genomic.fna	Lactobacillus	Firmicutes
260	GCA_000681515.1_ASM68151v1_genomic.fna	Listeria	Firmicutes
261	GCA_000737615.1_ASM73761v1_genomic.fna	Staphylococcus	Firmicutes
262	GCA_001678885.1_ASM167888v1_genomic.fna	Streptococcus	Firmicutes
263	GCA_002174215.1_ASM217421v1_genomic.fna	Pediococcus	Firmicutes
264	GCA_002073395.1_ASM207339v1_genomic.fna	Staphylococcus	Firmicutes
265	GCA_001412635.1_ASM141263v1_genomic.fna	Streptococcus	Firmicutes
266	GCA_002688645.1_ASM268864v1_genomic.fna	Bacillus	Firmicutes
267	GCA_002243305.1_ASM224330v1_genomic.fna	Weissella	Firmicutes
268	GCA_001685665.2_ASM168566v2_genomic.fna	Staphylococcus	Firmicutes
269	GCA_001477575.1_ASM147757v1_genomic.fna	Streptococcus	Firmicutes

270	GCA_002356075.1_ASM235607v1_genomic.fna	Mycoplasma	Tenericutes
271	GCA_002357155.1_ASM235715v1_genomic.fna	Rickettsia	Proteobacteria
272	GCA_900187085.1_50624_E01_genomic.fna	Streptococcus	Firmicutes
273	GCA_002224505.1_ASM222450v1_genomic.fna	Lactobacillus	Firmicutes
274	GCA_001611155.1_ASM161115v1_genomic.fna	Pediococcus	Firmicutes
275	GCA_001938665.1_ASM193866v1_genomic.fna	Bacillus	Firmicutes
276	GCA_001856645.1_ASM185664v1_genomic.fna	Clostridium	Firmicutes
277	GCA_002077215.1_ASM207721v1_genomic.fna	Bacillus	Firmicutes
278	GCA_001682235.1_ASM168223v1_genomic.fna	Staphylococcus	Firmicutes
279	GCA_001042565.2_ASM104256v2_genomic.fna	Francisella	Proteobacteria
280	GCA_000214235.1_ASM21423v1_genomic.fna	Serratia	Proteobacteria
281	GCA_001663115.1_ASM166311v1_genomic.fna	Serratia	Proteobacteria
282	GCA_001663755.1_ASM166375v1_genomic.fna	Lactobacillus	Firmicutes
283	GCA_000271325.1_ASM27132v1_genomic.fna	Solibacillus	Firmicutes
284	GCA_002003385.1_ASM200338v1_genomic.fna	Clostridium	Firmicutes
285	GCA_000341875.1_ASM34187v1_genomic.fna	Bacillus	Firmicutes
286	GCA_001548255.1_ASM154825v1_genomic.fna	Staphylococcus	Firmicutes
287	GCA_002216705.1_ASM221670v1_genomic.fna	Enterococcus	Firmicutes
288	GCA_000505725.1_ASM50572v1_genomic.fna	Francisella	Proteobacteria
289	GCA_001442475.1_ASM144247v1_genomic.fna	Rickettsia	Proteobacteria
290	GCA_000988065.1_ASM98806v1_genomic.fna	Mycoplasma	Tenericutes
291	GCA_002022005.1_ASM202200v1_genomic.fna	Campylobacter	Proteobacteria
292	GCA_000455565.1_ucmb5033v1_genomic.fna	Bacillus	Firmicutes
293	GCA_001275365.1_ASM127536v1_genomic.fna	Francisella	Proteobacteria
294	GCA_001663135.1_ASM166313v1_genomic.fna	Serratia	Proteobacteria
295	GCA_900187055.1_50377_D01_genomic.fna	Staphylococcus	Firmicutes
296	GCA_001267865.1_ASM126786v1_genomic.fna	Enterococcus	Firmicutes
297	GCA_001643975.1_ASM164397v1_genomic.fna	Campylobacter	Proteobacteria
298	GCA_000747585.1_ASM74758v1_genomic.fna	Melissococcus	Firmicutes
299	GCA_002220755.1_ASM222075v1_genomic.fna	Campylobacter	Proteobacteria
300	GCA_001273795.1_ASM127379v1_genomic.fna	Rickettsia	Proteobacteria
301	GCA_001190905.2_ASM119090v2_genomic.fna	Francisella	Proteobacteria
302	GCA_002357175.1_ASM235717v1_genomic.fna	Rickettsia	Proteobacteria
303	GCA_001856695.1_ASM185669v1_genomic.fna	Clostridium	Firmicutes

304	GCA_000214785.1_ASM21478v1_genomic.fna	Lactobacillus	Firmicutes
305	GCA_000221205.1_ASM22120v1_genomic.fna	Rickettsia	Proteobacteria
306	GCA_002003305.1_ASM200330v1_genomic.fna	Clostridium	Firmicutes
307	GCA_001244395.1_ASM124439v1_genomic.fna	Lactobacillus	Firmicutes
308	GCA_001705605.1_ASM170560v1_genomic.fna	Mycoplasma	Tenericutes
309	GCA_002356695.1_ASM235669v1_genomic.fna	Rickettsia	Proteobacteria
310	GCA_002243645.1_ASM224364v1_genomic.fna	Bacillus	Firmicutes
311	GCA_001702215.1_ASM170221v1_genomic.fna	Pediococcus	Firmicutes
312	GCA_002028345.1_ASM202834v1_genomic.fna	Spiroplasma	Tenericutes
313	GCA_000219805.1_ASM21980v1_genomic.fna	Weissella	Firmicutes
314	GCA_000223215.1_ASM22321v1_genomic.fna	Brachyspira	Spirochaetes
315	GCA_000517365.1_ASM51736v1_genomic.fna	Spiroplasma	Tenericutes
316	GCA_000270165.1_ASM27016v1_genomic.fna	Streptococcus	Firmicutes
317	GCA_001697145.1_ASM169714v1_genomic.fna	Streptococcus	Firmicutes
318	GCA_002250095.2_ASM225009v2_genomic.fna	Staphylococcus	Firmicutes
319	GCA_000271405.2_ASM27140v2_genomic.fna	Enterococcus	Firmicutes
320	GCA_001611035.1_ASM161103v1_genomic.fna	Pediococcus	Firmicutes
321	GCA_000875755.1_ASM87575v1_genomic.fna	Mycoplasma	Tenericutes
322	GCA_000283615.1_ASM28361v1_genomic.fna	Tetragenococcus	Firmicutes
323	GCA_000389635.1_ASM38963v1_genomic.fna	Clostridium	Firmicutes
324	GCA_000222305.1_ASM22230v1_genomic.fna	Borreliella	Spirochaetes
325	GCA_000252365.1_ASM25236v1_genomic.fna	Rickettsia	Proteobacteria
326	GCA_000709415.1_ASM70941v1_genomic.fna	Staphylococcus	Firmicutes
327	GCA_002176835.1_ASM217683v1_genomic.fna	Lactobacillus	Firmicutes
328	GCA_002209165.1_ASM220916v1_genomic.fna	Staphylococcus	Firmicutes
329	GCA_001641305.1_ASM164130v1_genomic.fna	Enterococcus	Firmicutes
330	GCA_002224425.1_ASM222442v1_genomic.fna	Lactobacillus	Firmicutes
331	GCA_001712815.1_ASM171281v1_genomic.fna	Tetragenococcus	Firmicutes
332	GCA_900116935.1_Chromosome_genomic.fna	Lactobacillus	Firmicutes
333	GCA_002211885.1_ASM221188v1_genomic.fna	Lactobacillus	Firmicutes
334	GCA_001663835.1_ASM166383v1_genomic.fna	Lactobacillus	Firmicutes
335	GCA_000724485.1_ASM72448v1_genomic.fna	Bacillus	Firmicutes
336	GCA_000284055.1_ASM28405v1_genomic.fna	Rickettsia	Proteobacteria
337	GCA_000283975.1_ASM28397v1_genomic.fna	Melissococcus	Firmicutes

338	GCA_002706745.1_ASM270674v1_genomic.fna	Lactobacillus	Firmicutes
339	GCA_001611135.1_ASM161113v1_genomic.fna	Pediococcus	Firmicutes
340	GCA_000189495.1_ASM18949v1_genomic.fna	Staphylococcus	Firmicutes
341	GCA_000807255.1_ASM80725v1_genomic.fna	Clostridium	Firmicutes
342	GCA_000283595.1_ASM28359v1_genomic.fna	Rickettsia	Proteobacteria
343	GCA_001543105.1_ASM154310v1_genomic.fna	Aerococcus	Firmicutes
344	GCA_000706685.1_ASM70668v1_genomic.fna	Staphylococcus	Firmicutes
345	GCA_000237845.1_ASM23784v1_genomic.fna	Rickettsia	Proteobacteria
346	GCA_000007785.1_ASM778v1_genomic.fna	Enterococcus	Firmicutes
347	GCA_000317855.1_ASM31785v1_genomic.fna	Streptococcus	Firmicutes
348	GCA_001922545.1_ASM192254v1_genomic.fna	Borreliella	Spirochaetes
349	GCA_000196735.1_ASM19673v1_genomic.fna	Bacillus	Firmicutes
350	GCA_000007465.2_ASM746v2_genomic.fna	Streptococcus	Firmicutes
351	GCA_000174395.2_ASM17439v2_genomic.fna	Enterococcus	Firmicutes
352	GCA_000832605.1_ASM83260v1_genomic.fna	Bacillus	Firmicutes
353	GCA_000026605.1_ASM2660v1_genomic.fna	Streptococcus	Firmicutes
354	GCA_000834965.1_ASM83496v1_genomic.fna	Francisella	Proteobacteria
355	GCA_000026505.1_ASM2650v1_genomic.fna	Lactobacillus	Firmicutes
356	GCA_000019285.1_ASM1928v1_genomic.fna	Francisella	Proteobacteria
357	GCA_001558875.1_ASM155887v1_genomic.fna	Enterococcus	Firmicutes
358	GCA_000157355.2_ASM15735v2_genomic.fna	Enterococcus	Firmicutes
359	GCA_000973705.1_ASM97370v1_genomic.fna	Clostridium	Firmicutes
360	GCA_000246835.1_ASM24683v1_genomic.fna	Streptococcus	Firmicutes
361	GCA_000800295.1_ASM80029v1_genomic.fna	Parvimonas	Firmicutes
362	GCA_001506405.1_ASM150640v1_genomic.fna	Campylobacter	Proteobacteria
363	GCA_001506425.1_ASM150642v1_genomic.fna	Campylobacter	Proteobacteria
364	GCA_000746505.1_ASM74650v1_genomic.fna	Staphylococcus	Firmicutes
365	GCA_000007025.1_ASM702v1_genomic.fna	Rickettsia	Proteobacteria
366	GCA_002055535.1_NCTC8198_genomic.fna	Streptococcus	Firmicutes
367	GCA_000017005.1_ASM1700v1_genomic.fna	Streptococcus	Firmicutes
368	GCA_000008765.1_ASM876v1_genomic.fna	Clostridium	Firmicutes
369	GCA_000014425.1_ASM1442v1_genomic.fna	Lactobacillus	Firmicutes
370	GCA_000746625.1_ASM74662v1_genomic.fna	Listeria	Firmicutes
371	GCA_002096095.1_ASM209609v1_genomic.fna	Bacillus	Firmicutes

372	GCA_000195795.1_ASM19579v1_genomic.fna	Listeria	Firmicutes
373	GCA_000027345.1_ASM2734v1_genomic.fna	Mycoplasma	Tenericutes
374	GCA_000195875.1_ASM19587v1_genomic.fna	Mycoplasma	Tenericutes
375	GCA_000011225.1_ASM1122v1_genomic.fna	Mycoplasma	Tenericutes
376	GCA_002072735.1_ASM207273v1_genomic.fna	Bacillus	Firmicutes
377	GCA_000359545.5_G255_GS_genomic.fna	Coxiella	Proteobacteria
378	GCA_002163545.1_ASM216354v1_genomic.fna	Streptococcus	Firmicutes
379	GCA_000800335.1_ASM80033v1_genomic.fna	Listeria	Firmicutes
380	GCA_001274875.1_ASM127487v1_genomic.fna	Spiroplasma	Tenericutes
381	GCA_000011145.1_ASM1114v1_genomic.fna	Bacillus	Firmicutes
382	GCA_001506465.1_ASM150646v1_genomic.fna	Campylobacter	Proteobacteria
383	GCA_002202295.1_ASM220229v1_genomic.fna	Streptococcus	Firmicutes
384	GCA_000808055.1_ASM80805v1_genomic.fna	Listeria	Firmicutes
385	GCA_000769575.1_ASM76957v1_genomic.fna	Staphylococcus	Firmicutes
386	GCA_001506445.1_ASM150644v1_genomic.fna	Campylobacter	Proteobacteria
387	GCA_000756205.1_ASM75620v1_genomic.fna	Staphylococcus	Firmicutes
388	GCA_002142595.1_ASM214259v1_genomic.fna	Bacillus	Firmicutes
389	GCA_002236855.1_ASM223685v1_genomic.fna	Streptococcus	Firmicutes
390	GCA_002236875.1_ASM223687v1_genomic.fna	Streptococcus	Firmicutes
391	GCA_002163815.1_ASM216381v1_genomic.fna	Bacillus	Firmicutes
392	GCA_000772025.1_ASM77202v1_genomic.fna	Staphylococcus	Firmicutes
393	GCA_001506485.1_ASM150648v1_genomic.fna	Campylobacter	Proteobacteria
394	GCA_000815045.1_ASM81504v1_genomic.fna	Staphylococcus	Firmicutes
395	GCA_001506505.1_ASM150650v1_genomic.fna	Campylobacter	Proteobacteria
396	GCA_000950775.1_ASM95077v1_genomic.fna	Listeria	Firmicutes
397	GCA_001296125.1_ASM129612v1_genomic.fna	Fusobacterium	Fusobacteria
398	GCA_002238295.1_ASM223829v1_genomic.fna	Streptococcus	Firmicutes
399	GCA_001005925.1_ASM100592v1_genomic.fna	Listeria	Firmicutes
400	GCA_001870065.1_ASM187006v1_genomic.fna	Bacillus	Firmicutes
401	GCA_900187025.1_49923_F01_genomic.fna	Listeria	Firmicutes
402	GCA_002162055.1_ASM216205v1_genomic.fna	Lactobacillus	Firmicutes
403	GCA_002173695.1_ASM217369v1_genomic.fna	Bacillus	Firmicutes
404	GCA_001611865.1_ASM161186v1_genomic.fna	Mycoplasma	Tenericutes
405	GCA_000954195.1_ASM95419v1_genomic.fna	Campylobacter	Proteobacteria

406	GCA_000286695.1_ASM28669v1_genomic.fna	Mycoplasma	Tenericutes
407	GCA_002117345.1_ASM211734v1_genomic.fna	Lactobacillus	Firmicutes
408	GCA_000412205.1_ASM41220v1_genomic.fna	Lactobacillus	Firmicutes
409	GCA_000816425.1_ASM81642v1_genomic.fna	Campylobacter	Proteobacteria
410	GCA_000331085.2_ASM33108v2_genomic.fna	Mycoplasma	Tenericutes
411	GCA_001280365.1_ASM128036v1_genomic.fna	Serratia	Proteobacteria
412	GCA_001886915.1_ASM188691v1_genomic.fna	Leuconostoc	Firmicutes
413	GCA_000298875.1_ASM29887v1_genomic.fna	Leuconostoc	Firmicutes
414	GCA_000418495.1_ASM41849v1_genomic.fna	Lactobacillus	Firmicutes
415	GCA_002706425.1_ASM270642v1_genomic.fna	Lactobacillus	Firmicutes
416	GCA_000833315.1_ASM83331v1_genomic.fna	Francisella	Proteobacteria
417	GCA_001932695.1_ASM193269v1_genomic.fna	Weissella	Firmicutes
418	GCA_001050455.1_ASM105045v1_genomic.fna	Bacillus	Firmicutes
419	GCA_002386185.1_ASM238618v1_genomic.fna	Staphylococcus	Firmicutes
420	GCA_000317915.1_ASM31791v1_genomic.fna	Enterococcus	Firmicutes
421	GCA_000193205.1_ASM19320v1_genomic.fna	Aerococcus	Firmicutes
422	GCA_001298485.1_ASM129848v1_genomic.fna	Enterococcus	Firmicutes
423	GCA_001558215.1_ASM155821v1_genomic.fna	Streptococcus	Firmicutes
424	GCA_000767725.1_ASM76772v1_genomic.fna	Mycoplasma	Tenericutes
425	GCA_000262385.1_ASM26238v1_genomic.fna	Bacillus	Firmicutes
426	GCA_000143725.1_ASM14372v1_genomic.fna	Brachyspira	Spirochaetes
427	GCA_000262145.1_ASM26214v1_genomic.fna	Streptococcus	Firmicutes
428	GCA_002151465.1_ASM215146v1_genomic.fna	Borreliella	Spirochaetes
429	GCA_000376625.1_ASM37662v1_genomic.fna	Mycoplasma	Tenericutes
430	GCA_000017065.1_ASM1706v1_genomic.fna	Clostridium	Firmicutes
431	GCA_000277245.1_ASM27724v1_genomic.fna	Rickettsia	Proteobacteria
432	GCA_001577795.1_ASM157779v1_genomic.fna	Clostridioides	Firmicutes
433	GCA_000318035.1_ASM31803v1_genomic.fna	Lactobacillus	Firmicutes
434	GCA_001578185.1_ASM157818v1_genomic.fna	Bacillus	Firmicutes
435	GCA_000463465.1_ASM46346v1_genomic.fna	Streptococcus	Firmicutes
436	GCA_000168875.3_ASM16887v3_genomic.fna	Coxiella	Proteobacteria
437	GCA_000203215.1_ASM20321v1_genomic.fna	Mycoplasma	Tenericutes
438	GCA_000027185.1_ASM2718v1_genomic.fna	Streptococcus	Firmicutes
439	GCA_000800785.1_ASM80078v1_genomic.fna	Mycoplasma	Tenericutes

440	GCA_001941785.1_ASM194178v1_genomic.fna	Lactobacillus	Firmicutes
441	GCA_000237995.2_ASM23799v2_genomic.fna	Pediococcus	Firmicutes
442	GCA_000194115.1_ASM19411v1_genomic.fna	Lactobacillus	Firmicutes
443	GCA_000283935.1_ASM28393v1_genomic.fna	Rickettsia	Proteobacteria
444	GCA_000307165.1_ASM30716v1_genomic.fna	Amphibacillus	Firmicutes
445	GCA_000270465.1_ASM27046v1_genomic.fna	Staphylococcus	Firmicutes
446	GCA_000525715.1_ASM52571v1_genomic.fna	Lactobacillus	Firmicutes
447	GCA_000463425.1_ASM46342v1_genomic.fna	Streptococcus	Firmicutes
448	GCA_000227485.1_ASM22748v1_genomic.fna	Bacillus	Firmicutes
449	GCA_001543085.1_ASM154308v1_genomic.fna	Streptococcus	Firmicutes
450	GCA_000815065.1_ASM81506v1_genomic.fna	Mycoplasma	Tenericutes
451	GCA_000524555.1_ASM52455v1_genomic.fna	Mycoplasma	Tenericutes
452	GCA_002005185.1_ASM200518v1_genomic.fna	Veillonella	Firmicutes
453	GCA_001029265.1_ASM102926v1_genomic.fna	Spiroplasma	Tenericutes
454	GCA_000759515.1_ASM75951v1_genomic.fna	Campylobacter	Proteobacteria
455	GCA_000953375.1_F38.1_genomic.fna	Mycoplasma	Tenericutes
456	GCA_001888905.1_ASM188890v1_genomic.fna	Lactobacillus	Firmicutes
457	GCA_000014645.1_ASM1464v1_genomic.fna	Francisella	Proteobacteria
458	GCA_001431145.1_ASM143114v1_genomic.fna	Bacillus	Firmicutes
459	GCA_000439275.1_ASM43927v1_genomic.fna	Lactobacillus	Firmicutes
460	GCA_900187035.1_50279_G02_genomic.fna	Megamonas	Firmicutes
461	GCA_000565195.1_ASM56519v1_genomic.fna	Spiroplasma	Tenericutes
462	GCA_001922325.1_ASM192232v1_genomic.fna	Pediococcus	Firmicutes
463	GCA_000328625.1_ASM32862v1_genomic.fna	Halobacteroides	Firmicutes
464	GCA_000193355.1_ASM19335v1_genomic.fna	Bacillus	Firmicutes
465	GCA_002356175.1_ASM235617v1_genomic.fna	Staphylococcus	Firmicutes
466	GCA_000404205.1_ASM40420v1_genomic.fna	Erysipelothrix	Firmicutes
467	GCA_000165925.1_ASM16592v1_genomic.fna	Bacillus	Firmicutes
468	GCA_001983955.1_ASM198395v1_genomic.fna	Streptococcus	Firmicutes
469	GCA_000262675.1_ASM26267v1_genomic.fna	Streptococcus	Firmicutes
470	GCA_002074095.1_ASM207409v1_genomic.fna	Bacillus	Firmicutes
471	GCA_000427035.1_09mas018883_genomic.fna	Streptococcus	Firmicutes
472	GCA_002238335.1_ASM223833v1_genomic.fna	Campylobacter	Proteobacteria
473	GCA_000816185.1_ASM81618v1_genomic.fna	Campylobacter	Proteobacteria

474	GCA_001701005.1_ASM170100v1_genomic.fna	Staphylococcus	Firmicutes
475	GCA_002139935.1_ASM213993v1_genomic.fna	Campylobacter	Proteobacteria
476	GCA_001267395.1_ASM126739v1_genomic.fna	Enterococcus	Firmicutes
477	GCA_002386265.1_ASM238626v1_genomic.fna	Weissella	Firmicutes
478	GCA_000340885.1_ASM34088v1_genomic.fna	Clostridium	Firmicutes
479	GCA_000829035.1_ASM82903v1_genomic.fna	Lactobacillus	Firmicutes
480	GCA_000504125.1_ASM50412v1_genomic.fna	Enterococcus	Firmicutes
481	GCA_000022105.1_ASM2210v1_genomic.fna	Brachyspira	Spirochaetes
482	GCA_000300135.1_ASM30013v1_genomic.fna	Leuconostoc	Firmicutes
483	GCA_000328545.1_ASM32854v1_genomic.fna	Thermoanaerobacte	Firmicutes
484	GCA_000816345.1_ASM81634v1_genomic.fna	Campylobacter	Proteobacteria
485	GCA_000306805.1_ASM30680v1_genomic.fna	Streptococcus	Firmicutes
486	GCA_002220735.1_ASM222073v1_genomic.fna	Campylobacter	Proteobacteria
487	GCA_001643955.1_ASM164395v1_genomic.fna	Campylobacter	Proteobacteria
488	GCA_002104335.1_ASM210433v1_genomic.fna	Campylobacter	Proteobacteria
489	GCA_002080395.1_ASM208039v1_genomic.fna	Campylobacter	Proteobacteria
490	GCA_000145705.1_ASM14570v1_genomic.fna	Mycoplasma	Tenericutes
491	GCA_000317975.2_ASM31797v2_genomic.fna	Carnobacterium	Firmicutes
492	GCA_000277795.1_ASM27779v1_genomic.fna	Mycoplasma	Tenericutes
493	GCA_001653955.1_ASM165395v1_genomic.fna	Francisella	Proteobacteria
494	GCA_000441535.1_ASM44153v1_genomic.fna	Streptococcus	Firmicutes
495	GCA_002157855.1_ASM215785v1_genomic.fna	Bacillus	Firmicutes
496	GCA_000284155.1_ASM28415v1_genomic.fna	Rickettsia	Proteobacteria
497	GCA_000284195.1_ASM28419v1_genomic.fna	Rickettsia	Proteobacteria
498	GCA_000283995.1_ASM28399v1_genomic.fna	Rickettsia	Proteobacteria
499	GCA_001885235.1_ASM188523v1_genomic.fna	Francisella	Proteobacteria
500	GCA_000262205.1_ASM26220v1_genomic.fna	Francisella	Proteobacteria
501	GCA_000238995.1_ASM23899v1_genomic.fna	Mycoplasma	Tenericutes
502	GCA_000307585.2_ASM30758v2_genomic.fna	Thermoanaerobacte	Firmicutes
503	GCA_000284075.1_ASM28407v1_genomic.fna	Rickettsia	Proteobacteria
504	GCA_000284175.1_ASM28417v1_genomic.fna	Rickettsia	Proteobacteria
505	GCA_000816085.1_ASM81608v1_genomic.fna	Staphylococcus	Firmicutes
506	GCA_000008885.1_ASM888v1_genomic.fna	Wigglesworthia	Proteobacteria
507	GCA_000011245.1_ASM1124v1_genomic.fna	Oceanobacillus	Firmicutes

508	GCA_000014445.1_ASM1444v1_genomic.fna	Leuconostoc	Firmicutes
509	GCA_000009545.1_ASM954v1_genomic.fna	Streptococcus	Firmicutes
510	GCA_000007625.1_ASM762v1_genomic.fna	Clostridium	Firmicutes
511	GCA_000011985.1_ASM1198v1_genomic.fna	Lactobacillus	Firmicutes
512	GCA_000055945.1_ASM5594v1_genomic.fna	Lawsonia	Proteobacteria
513	GCA_000203855.3_ASM20385v3_genomic.fna	Lactobacillus	Firmicutes
514	GCA_000014505.1_ASM1450v1_genomic.fna	Pediococcus	Firmicutes
515	GCA_000014465.1_ASM1446v1_genomic.fna	Lactobacillus	Firmicutes
516	GCA_000513215.1_DB11_genomic.fna	Serratia	Proteobacteria
517	GCA_000092585.1_ASM9258v1_genomic.fna	Mycoplasma	Tenericutes
518	GCA_000020065.1_ASM2006v1_genomic.fna	Mycoplasma	Tenericutes
519	GCA_000008305.1_ASM830v1_genomic.fna	Mesoplasma	Tenericutes
520	GCA_000013185.1_ASM1318v1_genomic.fna	Baumannia	Proteobacteria
521	GCA_002173715.1_ASM217371v1_genomic.fna	Bacillus	Firmicutes
522	GCA_000009865.1_ASM986v1_genomic.fna	Staphylococcus	Firmicutes
523	GCA_002024185.1_ASM202418v1_genomic.fna	Campylobacter	Proteobacteria
524	GCA_000183385.1_ASM18338v1_genomic.fna	Mycoplasma	Tenericutes
525	GCA_000019205.1_ASM1920v1_genomic.fna	Campylobacter	Proteobacteria
526	GCA_000021545.1_ASM2154v1_genomic.fna	Sulfurihydrogenibiu	Aquificae
527	GCA_000815085.1_ASM81508v1_genomic.fna	Staphylococcus	Firmicutes
528	GCA_001005985.1_ASM100598v1_genomic.fna	Listeria	Firmicutes
529	GCA_000833105.2_ASM83310v2_genomic.fna	Clostridium	Firmicutes
530	GCA_000014345.1_ASM1434v1_genomic.fna	Rickettsia	Proteobacteria
531	GCA_000018205.1_ASM1820v1_genomic.fna	Rickettsia	Proteobacteria
532	GCA_001296185.1_ASM129618v1_genomic.fna	Fusobacterium	Fusobacteria
533	GCA_002238315.1_ASM223831v1_genomic.fna	Streptococcus	Firmicutes
534	GCA_001027065.1_ASM102706v1_genomic.fna	Listeria	Firmicutes
535	GCA_002355175.1_ASM235517v1_genomic.fna	Streptococcus	Firmicutes
536	GCA_001506525.1_ASM150652v1_genomic.fna	Campylobacter	Proteobacteria
537	GCA_001506545.1_ASM150654v1_genomic.fna	Campylobacter	Proteobacteria
538	GCA_000815125.1_ASM81512v1_genomic.fna	Staphylococcus	Firmicutes
539	GCA_002201955.1_ASM220195v1_genomic.fna	Bacillus	Firmicutes
540	GCA_000008925.1_ASM892v1_genomic.fna	Lactobacillus	Firmicutes
541	GCA_000009825.1_ASM982v1_genomic.fna	Bacillus	Firmicutes

542	GCA_002211645.1_ASM221164v1_genomic.fna	Fusobacterium	Fusobacteria
543	GCA_002201995.1_ASM220199v1_genomic.fna	Bacillus	Firmicutes
544	GCA_002355815.1_ASM235581v1_genomic.fna	Streptococcus	Firmicutes
545	GCA_001027085.1_ASM102708v1_genomic.fna	Listeria	Firmicutes
546	GCA_001506565.1_ASM150656v1_genomic.fna	Campylobacter	Proteobacteria
547	GCA_000815165.1_ASM81516v1_genomic.fna	Staphylococcus	Firmicutes
548	GCA_000815205.1_ASM81520v1_genomic.fna	Staphylococcus	Firmicutes
549	GCA_001506585.1_ASM150658v1_genomic.fna	Campylobacter	Proteobacteria
550	GCA_002557755.1_ASM255775v1_genomic.fna	Streptococcus	Firmicutes
551	GCA_001027125.1_ASM102712v1_genomic.fna	Listeria	Firmicutes
552	GCA_002202055.1_ASM220205v1_genomic.fna	Bacillus	Firmicutes
553	GCA_002202035.1_ASM220203v1_genomic.fna	Bacillus	Firmicutes
554	GCA_001027165.1_ASM102716v1_genomic.fna	Listeria	Firmicutes
555	GCA_001506605.1_ASM150660v1_genomic.fna	Campylobacter	Proteobacteria
556	GCA_000815245.1_ASM81524v1_genomic.fna	Staphylococcus	Firmicutes
557	GCA_001506625.1_ASM150662v1_genomic.fna	Campylobacter	Proteobacteria
558	GCA_001027205.1_ASM102720v1_genomic.fna	Listeria	Firmicutes
559	GCA_000828035.1_ASM82803v1_genomic.fna	Staphylococcus	Firmicutes
560	GCA_000956315.1_ASM95631v1_genomic.fna	Borrelia	Spirochaetes
561	GCA_002197485.1_ASM219748v1_genomic.fna	Anoxybacillus	Firmicutes
562	GCA_000211375.1_ASM21137v1_genomic.fna	Lactobacillus	Firmicutes
563	GCA_000008245.1_ASM824v1_genomic.fna	Mycoplasma	Tenericutes
564	GCA_000026065.1_ASM2606v1_genomic.fna	Lactobacillus	Firmicutes
565	GCA_000200735.1_ASM20073v1_genomic.fna	Mycoplasma	Tenericutes
566	GCA_000215345.3_ASM21534v3_genomic.fna	Arcobacter	Proteobacteria
567	GCA_002119825.1_ASM211982v1_genomic.fna	Macrococcus	Firmicutes
568	GCA_000091405.1_ASM9140v1_genomic.fna	Lactobacillus	Firmicutes
569	GCA_000277285.1_ASM27728v1_genomic.fna	Rickettsia	Proteobacteria
570	GCA_001581875.1_ASM158187v1_genomic.fna	Lysinibacillus	Firmicutes
571	GCA_001465175.1_ASM146517v1_genomic.fna	Clostridium	Firmicutes
572	GCA_001885145.1_ASM188514v1_genomic.fna	Streptococcus	Firmicutes
573	GCA_000017725.2_ASM1772v2_genomic.fna	Campylobacter	Proteobacteria
574	GCA_001553195.1_ASM155319v1_genomic.fna	Mycoplasma	Tenericutes
575	GCA_001941385.1_ASM194138v1_genomic.fna	Thermosipho	Thermotogae

576	GCA_000299355.1_ASM29935v1_genomic.fna	Gottschalkia	Firmicutes
577	GCA_000248095.3_ASM24809v3_genomic.fna	Lactobacillus	Firmicutes
578	GCA_000169195.2_ASM16919v2_genomic.fna	Bacillus	Firmicutes
579	GCA_000565155.1_ASM56515v1_genomic.fna	Listeria	Firmicutes
580	GCA_900187315.1_52087_C01_genomic.fna	Listeria	Firmicutes
581	GCA_000283635.1_ASM28363v1_genomic.fna	Streptococcus	Firmicutes
582	GCA_000148625.1_ASM14862v1_genomic.fna	Mycoplasma	Tenericutes
583	GCA_000016965.1_ASM1696v1_genomic.fna	Clostridium	Firmicutes
584	GCA_000283915.1_ASM28391v1_genomic.fna	Rickettsia	Proteobacteria
585	GCA_000143435.1_ASM14343v1_genomic.fna	Lactobacillus	Firmicutes
586	GCA_000737305.2_ASM73730v2_genomic.fna	Bacillus	Firmicutes
587	GCA_000479355.1_ASM47935v1_genomic.fna	Mesoplasma	Tenericutes
588	GCA_001190755.1_ASM119075v1_genomic.fna	Campylobacter	Proteobacteria
589	GCA_001611955.1_ASM161195v1_genomic.fna	Staphylococcus	Firmicutes
590	GCA_000465235.1_ASM46523v1_genomic.fna	Campylobacter	Proteobacteria
591	GCA_001586195.1_ASM158619v1_genomic.fna	Solibacillus	Firmicutes
592	GCA_000816225.1_ASM81622v1_genomic.fna	Campylobacter	Proteobacteria
593	GCA_000219375.1_ASM21937v1_genomic.fna	Mycoplasma	Tenericutes
594	GCA_000389675.2_ASM38967v2_genomic.fna	Lactobacillus	Firmicutes
595	GCA_000331715.1_ASM33171v1_genomic.fna	Lawsonia	Proteobacteria
596	GCA_000176835.2_ASM17683v2_genomic.fna	Serratia	Proteobacteria
597	GCA_000359625.1_ASM35962v1_genomic.fna	Lactobacillus	Firmicutes
598	GCA_000496265.1_ASM49626v1_genomic.fna	Pediococcus	Firmicutes
599	GCA_000330865.1_ASM33086v1_genomic.fna	Serratia	Proteobacteria
600	GCA_000025365.1_ASM2536v1_genomic.fna	Mycoplasma	Tenericutes
601	GCA_001432245.1_ASM143224v1_genomic.fna	Staphylococcus	Firmicutes
602	GCA_000967115.1_CT1_genomic.fna	Clostridium	Firmicutes
603	GCA_000981525.1_L_piscium_genomic.fna	Lactococcus	Firmicutes
604	GCA_001895885.1_ASM189588v1_genomic.fna	Bacillus	Firmicutes
605	GCA_002443015.1_ASM244301v1_genomic.fna	Bacillus	Firmicutes
606	GCA_001547975.1_ASM154797v1_genomic.fna	Mycoplasma	Tenericutes
607	GCA_001687565.2_ASM168756v2_genomic.fna	Planococcus	Firmicutes
608	GCA_001886855.1_ASM188685v1_genomic.fna	Spiroplasma	Tenericutes
609	GCA_000186005.1_ASM18600v1_genomic.fna	Mycoplasma	Tenericutes

610	GCA_000252975.1_ASM25297v1_genomic.fna	Listeria	Firmicutes
611	GCA_000027145.1_ASM2714v1_genomic.fna	Listeria	Firmicutes
612	GCA_000060285.1_ASM6028v1_genomic.fna	Listeria	Firmicutes
613	GCA_000143685.1_ASM14368v1_genomic.fna	Clostridium	Firmicutes
614	GCA_000775975.1_ASM77597v1_genomic.fna	Bacillus	Firmicutes
615	GCA_000012145.1_ASM1214v1_genomic.fna	Rickettsia	Proteobacteria
616	GCA_000014205.1_ASM1420v1_genomic.fna	Streptococcus	Firmicutes
617	GCA_000010125.1_ASM1012v1_genomic.fna	Staphylococcus	Firmicutes
618	GCA_000832905.1_ASM83290v1_genomic.fna	Bacillus	Firmicutes
619	GCA_000026405.1_ASM2640v1_genomic.fna	Leuconostoc	Firmicutes
620	GCA_002269175.1_ASM226917v1_genomic.fna	Bacillus	Firmicutes
621	GCA_002216085.1_ASM221608v1_genomic.fna	Bacillus	Firmicutes
622	GCA_000953255.1_Staphylococcus_aureus_Sa_ILRI_217_genomic.fna	Staphylococcus	Firmicutes
623	GCA_001027245.1_ASM102724v1_genomic.fna	Listeria	Firmicutes
624	GCA_001506645.1_ASM150664v1_genomic.fna	Campylobacter	Proteobacteria
625	GCA_000014125.1_ASM1412v1_genomic.fna	Clostridium	Firmicutes
626	GCA_000831485.1_ASM83148v1_genomic.fna	Streptococcus	Firmicutes
627	GCA_001298465.1_ASM129846v1_genomic.fna	Campylobacter	Proteobacteria
628	GCA_000017465.2_ASM1746v2_genomic.fna	Campylobacter	Proteobacteria
629	GCA_000016905.1_ASM1690v1_genomic.fna	Thermosipho	Thermotogae
630	GCA_001506665.1_ASM150666v1_genomic.fna	Campylobacter	Proteobacteria
631	GCA_001047715.1_ASM104771v1_genomic.fna	Listeria	Firmicutes
632	GCA_000967325.1_ASM96732v1_genomic.fna	Staphylococcus	Firmicutes
633	GCA_000817935.1_ASM81793v1_genomic.fna	Clostridium	Firmicutes
634	GCA_001554095.1_ASM155409v1_genomic.fna	Mycoplasma	Tenericutes
635	GCA_000706705.1_ASM70670v1_genomic.fna	Bacillus	Firmicutes
636	GCA_002290305.1_ASM229030v1_genomic.fna	Bacillus	Firmicutes
637	GCA_001895905.1_ASM189590v1_genomic.fna	Enterococcus	Firmicutes
638	GCA_001596755.1_ASM159675v1_genomic.fna	Bacillus	Firmicutes
639	GCA_002355755.1_ASM235575v1_genomic.fna	Enterococcus	Firmicutes
640	GCA_002269195.1_ASM226919v1_genomic.fna	Bacillus	Firmicutes
641	GCA_000803665.1_ASM80366v1_genomic.fna	Bacillus	Firmicutes
642	GCA_000967345.1_ASM96734v1_genomic.fna	Staphylococcus	Firmicutes
643	GCA_001188655.1_ASM118865v1_genomic.fna	Listeria	Firmicutes

644	GCA_002285775.1_ASM228577v1_genomic.fna	Lactobacillus	Firmicutes
645	GCA_000313385.1_ASM31338v1_genomic.fna	Francisella	Proteobacteria
646	GCA_000967365.1_ASM96736v1_genomic.fna	Staphylococcus	Firmicutes
647	GCA_001190805.1_ASM119080v1_genomic.fna	Streptococcus	Firmicutes
648	GCA_001506705.1_ASM150670v1_genomic.fna	Campylobacter	Proteobacteria
649	GCA_000471985.1_ASM47198v1_genomic.fna	Streptococcus	Firmicutes
650	GCA_001454845.1_ASM145484v1_genomic.fna	Listeria	Firmicutes
651	GCA_000832505.1_ASM83250v1_genomic.fna	Bacillus	Firmicutes
652	GCA_000210935.1_ASM21093v1_genomic.fna	Streptococcus	Firmicutes
653	GCA_000250925.1_ASM25092v1_genomic.fna	Streptococcus	Firmicutes
654	GCA_002012365.1_ASM201236v1_genomic.fna	Streptococcus	Firmicutes
655	GCA_001506685.1_ASM150668v1_genomic.fna	Campylobacter	Proteobacteria
656	GCA_002556525.1_ASM255652v1_genomic.fna	Bacillus	Firmicutes
657	GCA_000218305.1_ASM21830v1_genomic.fna	Listeria	Firmicutes
658	GCA_002078395.1_ASM207839v1_genomic.fna	Lactococcus	Firmicutes
659	GCA_000832385.1_ASM83238v1_genomic.fna	Bacillus	Firmicutes
660	GCA_000021065.1_ASM2106v1_genomic.fna	Buchnera	Proteobacteria
661	GCA_000024585.1_ASM2458v1_genomic.fna	Staphylococcus	Firmicutes
662	GCA_000025145.2_ASM2514v1_genomic.fna	Staphylococcus	Firmicutes
663	GCA_000468915.2_ASM46891v2_genomic.fna	Campylobacter	Proteobacteria
664	GCA_000832405.1_ASM83240v1_genomic.fna	Bacillus	Firmicutes
665	GCA_002078415.1_ASM207841v1_genomic.fna	Lactococcus	Firmicutes
666	GCA_000258905.1_ASM25890v1_genomic.fna	Listeria	Firmicutes
667	GCA_001454865.1_ASM145486v1_genomic.fna	Listeria	Firmicutes
668	GCA_000466105.2_ASM46610v2_genomic.fna	Campylobacter	Proteobacteria
669	GCA_000967405.1_ASM96740v1_genomic.fna	Staphylococcus	Firmicutes
670	GCA_000021085.1_ASM2108v1_genomic.fna	Buchnera	Proteobacteria
671	GCA_001454885.1_ASM145488v1_genomic.fna	Listeria	Firmicutes
672	GCA_000307535.1_ASM30753v1_genomic.fna	Streptococcus	Firmicutes
673	GCA_000210955.1_ASM21095v1_genomic.fna	Streptococcus	Firmicutes
674	GCA_001506725.1_ASM150672v1_genomic.fna	Campylobacter	Proteobacteria
675	GCA_000967385.1_ASM96738v1_genomic.fna	Staphylococcus	Firmicutes
676	GCA_001506745.1_ASM150674v1_genomic.fna	Campylobacter	Proteobacteria
677	GCA_002286255.1_ASM228625v1_genomic.fna	Streptococcus	Firmicutes

678	GCA_000832565.1_ASM83256v1_genomic.fna	Bacillus	Firmicutes
679	GCA_001190885.1_ASM119088v1_genomic.fna	Streptococcus	Firmicutes
680	GCA_000494895.1_ASM49489v1_genomic.fna	Streptococcus	Firmicutes
681	GCA_000524575.1_ASM52457v1_genomic.fna	Francisella	Proteobacteria
682	GCA_900196735.1_Lactobacillus_bulgaricus_ACA-DC_87_genomic.fna	Lactobacillus	Firmicutes
683	GCA_000737405.1_ASM73740v1_genomic.fna	Bacillus	Firmicutes
684	GCA_000832485.1_ASM83248v1_genomic.fna	Bacillus	Firmicutes
685	GCA_000234825.3_ASM23482v3_genomic.fna	Leuconostoc	Firmicutes
686	GCA_000023085.1_ASM2308v1_genomic.fna	Lactobacillus	Firmicutes
687	GCA_000143945.1_ASM14394v1_genomic.fna	Mycoplasma	Tenericutes
688	GCA_000247565.1_ASM24756v1_genomic.fna	Wigglesworthia	Proteobacteria
689	GCA_001953235.1_ASM195323v1_genomic.fna	Enterococcus	Firmicutes
690	GCA_001554115.1_ASM155411v1_genomic.fna	Mycoplasma	Tenericutes
691	GCA_000827935.1_ASM82793v1_genomic.fna	Clostridium	Firmicutes
692	GCA_001705195.1_ASM170519v1_genomic.fna	Bacillus	Firmicutes
693	GCA_002158885.1_ASM215888v1_genomic.fna	Lactobacillus	Firmicutes
694	GCA_000191905.1_ASM19190v1_genomic.fna	Clostridium	Firmicutes
695	GCA_000970665.2_ASM97066v2_genomic.fna	Streptococcus	Firmicutes
696	GCA_000832985.1_ASM83298v1_genomic.fna	Bacillus	Firmicutes
697	GCA_001477615.1_ASM147761v1_genomic.fna	Streptococcus	Firmicutes
698	GCA_001020205.1_ASM102020v1_genomic.fna	Clostridium	Firmicutes
699	GCA_000833195.1_ASM83319v1_genomic.fna	Francisella	Proteobacteria
700	GCA_002393265.1_ASM239326v1_genomic.fna	Enterococcus	Firmicutes
701	GCA_000294775.2_ASM29477v2_genomic.fna	Bacillus	Firmicutes
702	GCA_000972245.3_ASM97224v3_genomic.fna	Bacillus	Firmicutes
703	GCA_001880205.1_ASM188020v1_genomic.fna	Francisella	Proteobacteria
704	GCA_001878675.1_ASM187867v1_genomic.fna	Virgibacillus	Firmicutes
705	GCA_000011045.1_ASM1104v1_genomic.fna	Lactobacillus	Firmicutes
706	GCA_000020765.1_ASM2076v1_genomic.fna	Streptococcus	Firmicutes
707	GCA_000091645.1_ASM9164v1_genomic.fna	Streptococcus	Firmicutes
708	GCA_000250945.1_ASM25094v1_genomic.fna	Enterococcus	Firmicutes
709	GCA_001642655.1_ASM164265v1_genomic.fna	Clostridium	Firmicutes
710	GCA_000018825.1_ASM1882v1_genomic.fna	Bacillus	Firmicutes
711	GCA_000195515.1_ASM19551v1_genomic.fna	Bacillus	Firmicutes

712	GCA_001687585.2_ASM168758v2_genomic.fna	Planococcus	Firmicutes
713	GCA_000010705.1_ASM1070v1_genomic.fna	Streptococcus	Firmicutes
714	GCA_000239475.1_ASM23947v1_genomic.fna	Borrelia	Spirochaetes
715	GCA_001572725.1_ASM157272v1_genomic.fna	Serratia	Proteobacteria
716	GCA_000021405.1_ASM2140v1_genomic.fna	Borrelia	Spirochaetes
717	GCA_000022785.1_ASM2278v1_genomic.fna	Rickettsia	Proteobacteria
718	GCA_000210515.1_ASM21051v1_genomic.fna	Lactobacillus	Firmicutes
719	GCA_000010265.1_ASM1026v1_genomic.fna	Clostridium	Firmicutes
720	GCA_002202015.1_ASM220201v1_genomic.fna	Bacillus	Firmicutes
721	GCA_000023165.1_ASM2316v1_genomic.fna	Mycoplasma	Tenericutes
722	GCA_002109385.1_ASM210938v1_genomic.fna	Bacillus	Firmicutes
723	GCA_000020165.1_ASM2016v1_genomic.fna	Clostridium	Firmicutes
724	GCA_002208805.1_ASM220880v1_genomic.fna	Staphylococcus	Firmicutes
725	GCA_000222835.1_ASM22283v1_genomic.fna	Borrelia	Spirochaetes
726	GCA_000269945.1_ASM26994v1_genomic.fna	Lactococcus	Firmicutes
727	GCA_000422085.1_ASM42208v1_genomic.fna	Serratia	Proteobacteria
728	GCA_000499665.2_RMONA_1_genomic.fna	Rickettsia	Proteobacteria
729	GCA_000328725.1_ASM32872v1_genomic.fna	Mycoplasma	Tenericutes
730	GCA_002310475.1_ASM231047v1_genomic.fna	Lactococcus	Firmicutes
731	GCA_002356415.1_ASM235641v1_genomic.fna	Streptococcus	Firmicutes
732	GCA_000439435.1_ASM43943v1_genomic.fna	Spiroplasma	Tenericutes
733	GCA_000750535.1_ASM75053v1_genomic.fna	Weissella	Firmicutes
734	GCA_001951175.1_ASM195117v1_genomic.fna	Lactobacillus	Firmicutes
735	GCA_000018085.1_ASM1808v1_genomic.fna	Serratia	Proteobacteria
736	GCA_000019685.1_ASM1968v1_genomic.fna	Borrelia	Spirochaetes
737	GCA_000019705.1_ASM1970v1_genomic.fna	Borrelia	Spirochaetes
738	GCA_000023005.1_ASM2300v1_genomic.fna	Rickettsia	Proteobacteria
739	GCA_000016625.1_ASM1662v1_genomic.fna	Rickettsia	Proteobacteria
740	GCA_000010185.1_ASM1018v1_genomic.fna	Finegoldia	Firmicutes
741	GCA_000255135.1_ASM25513v1_genomic.fna	Marinitoga	Thermotogae
742	GCA_001753205.1_ASM175320v1_genomic.fna	Lysinibacillus	Firmicutes
743	GCA_000739475.1_ASM73947v1_genomic.fna	Borreliella	Spirochaetes
744	GCA_001456065.2_ASM145606v2_genomic.fna	Clostridium	Firmicutes
745	GCA_000017585.1_ASM1758v1_genomic.fna	Campylobacter	Proteobacteria

746	GCA_000010585.1_ASM1058v1_genomic.fna	Macrococcus	Firmicutes
747	GCA_000008065.1_ASM806v1_genomic.fna	Lactobacillus	Firmicutes
748	GCA_000969765.1_ASM96976v1_genomic.fna	Mycoplasma	Tenericutes
749	GCA_000008045.1_ASM804v1_genomic.fna	Rickettsia	Proteobacteria
750	GCA_000008365.1_ASM836v1_genomic.fna	Mycoplasma	Tenericutes
751	GCA_000186985.3_ASM18698v3_genomic.fna	Mycoplasma	Tenericutes
752	GCA_000732385.1_ASM73238v1_genomic.fna	Streptococcus	Firmicutes
753	GCA_002224565.1_ASM222456v1_genomic.fna	Lactobacillus	Firmicutes
754	GCA_001535505.1_ASM153550v1_genomic.fna	Streptococcus	Firmicutes
755	GCA_001547815.1_ASM154781v1_genomic.fna	Streptococcus	Firmicutes
756	GCA_001506765.1_ASM150676v1_genomic.fna	Campylobacter	Proteobacteria
757	GCA_001506785.1_ASM150678v1_genomic.fna	Campylobacter	Proteobacteria
758	GCA_000012085.2_ASM1208v2_genomic.fna	Borrelia	Spirochaetes
759	GCA_000014025.1_ASM1402v1_genomic.fna	Arcobacter	Proteobacteria
760	GCA_001454905.1_ASM145490v1_genomic.fna	Listeria	Firmicutes
761	GCA_000969225.1_ASM96922v1_genomic.fna	Staphylococcus	Firmicutes
762	GCA_001272875.1_ASM127287v1_genomic.fna	Mycoplasma	Tenericutes
763	GCA_000183365.1_ASM18336v1_genomic.fna	Mycoplasma	Tenericutes
764	GCA_000021285.1_ASM2128v1_genomic.fna	Thermosipho	Thermotogae
765	GCA_001454925.1_ASM145492v1_genomic.fna	Listeria	Firmicutes
766	GCA_001021875.1_ASM102187v1_genomic.fna	Staphylococcus	Firmicutes
767	GCA_002072755.1_ASM207275v1_genomic.fna	Staphylococcus	Firmicutes
768	GCA_001593395.2_ASM159339v2_genomic.fna	Bacillus	Firmicutes
769	GCA_001685645.1_ASM168564v1_genomic.fna	Bacillus	Firmicutes
770	GCA_001659745.1_ASM165974v1_genomic.fna	Lactobacillus	Firmicutes
771	GCA_002220655.1_ASM222065v1_genomic.fna	Serratia	Proteobacteria
772	GCA_001936355.1_ASM193635v1_genomic.fna	Campylobacter	Proteobacteria
773	GCA_001506825.1_ASM150682v1_genomic.fna	Campylobacter	Proteobacteria
774	GCA_001660025.1_ASM166002v1_genomic.fna	Lactobacillus	Firmicutes
775	GCA_001027045.1_ASM102704v1_genomic.fna	Staphylococcus	Firmicutes
776	GCA_001675515.1_ASM167551v1_genomic.fna	Bacillus	Firmicutes
777	GCA_000774075.2_ASM77407v2_genomic.fna	Bacillus	Firmicutes
778	GCA_001272895.1_ASM127289v1_genomic.fna	Mycoplasma	Tenericutes
779	GCA_001483425.1_ASM148342v1_genomic.fna	Listeria	Firmicutes

780	GCA_001021895.1_ASM102189v1_genomic.fna	Staphylococcus	Firmicutes
781	GCA_001483405.1_ASM148340v1_genomic.fna	Listeria	Firmicutes
782	GCA_001506805.1_ASM150680v1_genomic.fna	Campylobacter	Proteobacteria
783	GCA_002407145.1_ASM240714v1_genomic.fna	Campylobacter	Proteobacteria
784	GCA_002220675.1_ASM222067v1_genomic.fna	Serratia	Proteobacteria
785	GCA_000827955.1_ASM82795v1_genomic.fna	Clostridium	Firmicutes
786	GCA_900094185.1_Ef_DMG1500501_genomic.fna	Enterococcus	Firmicutes
787	GCA_001594345.1_ASM159434v1_genomic.fna	Enterococcus	Firmicutes
788	GCA_000772125.1_ASM77212v1_genomic.fna	Bacillus	Firmicutes
789	GCA_000583775.1_ASM58377v1_genomic.fna	Campylobacter	Proteobacteria
790	GCA_000833165.1_ASM83316v1_genomic.fna	Francisella	Proteobacteria
791	GCA_001953255.1_ASM195325v1_genomic.fna	Enterococcus	Firmicutes
792	GCA_001874385.1_ASM187438v1_genomic.fna	Bacillus	Firmicutes
793	GCA_001483445.1_ASM148344v1_genomic.fna	Listeria	Firmicutes
794	GCA_002443035.1_ASM244303v1_genomic.fna	Streptococcus	Firmicutes
795	GCA_001506845.1_ASM150684v1_genomic.fna	Campylobacter	Proteobacteria
796	GCA_001027105.1_ASM102710v1_genomic.fna	Staphylococcus	Firmicutes
797	GCA_001266635.1_ASM126663v1_genomic.fna	Streptococcus	Firmicutes
798	GCA_001506865.1_ASM150686v1_genomic.fna	Campylobacter	Proteobacteria
799	GCA_001305715.1_ASM130571v1_genomic.fna	Campylobacter	Proteobacteria
800	GCA_000832825.1_ASM83282v1_genomic.fna	Bacillus	Firmicutes
801	GCA_000210975.1_ASM21097v1_genomic.fna	Streptococcus	Firmicutes
802	GCA_000349925.2_ASM34992v2_genomic.fna	Streptococcus	Firmicutes
803	GCA_000183225.1_ASM18322v1_genomic.fna	Buchnera	Proteobacteria
804	GCA_001513695.1_ASM151369v1_genomic.fna	Listeria	Firmicutes
805	GCA_000832585.1_ASM83258v1_genomic.fna	Bacillus	Firmicutes
806	GCA_001045795.2_ASM104579v2_genomic.fna	Staphylococcus	Firmicutes
807	GCA_000732355.1_ASM73235v1_genomic.fna	Streptococcus	Firmicutes
808	GCA_000832525.1_ASM83252v1_genomic.fna	Bacillus	Firmicutes
809	GCA_000493495.1_TS_genomic.fna	Campylobacter	Proteobacteria
810	GCA_000027045.1_ASM2704v1_genomic.fna	Staphylococcus	Firmicutes
811	GCA_000144955.1_ASM14495v1_genomic.fna	Staphylococcus	Firmicutes
812	GCA_000632435.1_ASM63243v1_genomic.fna	Campylobacter	Proteobacteria
813	GCA_000306905.1_ASM30690v1_genomic.fna	Listeria	Firmicutes

814	GCA_002078435.1_ASM207843v1_genomic.fna	Lactococcus	Firmicutes
815	GCA_000306985.1_ASM30698v1_genomic.fna	Listeria	Firmicutes
816	GCA_000183245.1_ASM18324v1_genomic.fna	Buchnera	Proteobacteria
817	GCA_000832765.1_ASM83276v1_genomic.fna	Bacillus	Firmicutes
818	GCA_002078455.1_ASM207845v1_genomic.fna	Lactococcus	Firmicutes
819	GCA_000993745.1_ASM99374v1_genomic.fna	Streptococcus	Firmicutes
820	GCA_001275545.2_ASM127554v2_genomic.fna	Streptococcus	Firmicutes
821	GCA_900094135.1_ASM90009413v1_genomic.fna	Streptococcus	Firmicutes
822	GCA_000422045.1_ASM42204v1_genomic.fna	Streptococcus	Firmicutes
823	GCA_000210995.1_ASM21099v1_genomic.fna	Streptococcus	Firmicutes
824	GCA_000832635.1_ASM83263v1_genomic.fna	Bacillus	Firmicutes
825	GCA_000018225.1_ASM1822v1_genomic.fna	Rickettsia	Proteobacteria
826	GCA_000017105.1_ASM1710v1_genomic.fna	Coxiella	Proteobacteria
827	GCA_000026485.1_ASM2648v1_genomic.fna	Lactobacillus	Firmicutes
828	GCA_000146565.1_ASM14656v1_genomic.fna	Bacillus	Firmicutes
829	GCA_000018245.1_ASM1824v1_genomic.fna	Rickettsia	Proteobacteria
830	GCA_000015385.1_ASM1538v1_genomic.fna	Lactobacillus	Firmicutes
831	GCA_000833235.1_ASM83323v1_genomic.fna	Francisella	Proteobacteria
832	GCA_000772165.1_ASM77216v1_genomic.fna	Bacillus	Firmicutes
833	GCA_000832925.1_ASM83292v1_genomic.fna	Bacillus	Firmicutes
834	GCA_000085225.1_ASM8522v1_genomic.fna	Clostridioides	Firmicutes
835	GCA_000089865.1_ASM8986v1_genomic.fna	Mycoplasma	Tenericutes
836	GCA_000014405.1_ASM1440v1_genomic.fna	Lactobacillus	Firmicutes
837	GCA_000192395.2_ASM19239v2_genomic.fna	Mycoplasma	Tenericutes
838	GCA_001281025.1_ASM128102v1_genomic.fna	Streptococcus	Firmicutes
839	GCA_000009245.1_ASM924v1_genomic.fna	Francisella	Proteobacteria
840	GCA_000497525.2_ASM49752v2_genomic.fna	Bacillus	Firmicutes
841	GCA_000253315.1_ASM25331v1_genomic.fna	Streptococcus	Firmicutes
842	GCA_000400955.1_ASM40095v1_genomic.fna	Spiroplasma	Tenericutes
843	GCA_000400935.1_ASM40093v1_genomic.fna	Spiroplasma	Tenericutes
844	GCA_000439455.1_ASM43945v1_genomic.fna	Spiroplasma	Tenericutes
845	GCA_000010005.1_ASM1000v1_genomic.fna	Lactobacillus	Firmicutes
846	GCA_000017885.4_ASM1788v4_genomic.fna	Bacillus	Firmicutes
847	GCA_000500935.1_ASM50093v1_genomic.fna	Spiroplasma	Tenericutes

848	GCA_000565215.1_ASM56521v1_genomic.fna	Spiroplasma	Tenericutes
849	GCA_000565175.1_ASM56517v1_genomic.fna	Spiroplasma	Tenericutes
850	GCA_000292405.1_ASM29240v1_genomic.fna	Mycoplasma	Tenericutes
851	GCA_000011825.1_ASM1182v1_genomic.fna	Streptococcus	Firmicutes
852	GCA_000008425.1_ASM842v1_genomic.fna	Bacillus	Firmicutes
853	GCA_000494875.1_ASM49487v1_genomic.fna	Staphylococcus	Firmicutes
854	GCA_001518795.1_ASM151879v1_genomic.fna	Aerococcus	Firmicutes
855	GCA_000008225.1_ASM822v1_genomic.fna	Mycoplasma	Tenericutes
856	GCA_000018185.1_ASM1818v1_genomic.fna	Streptococcus	Firmicutes
857	GCA_000445425.4_ASM44542v4_genomic.fna	Borrelia	Spirochaetes
858	GCA_000012705.1_ASM1270v1_genomic.fna	Streptococcus	Firmicutes
859	GCA_000007285.1_ASM728v1_genomic.fna	Streptococcus	Firmicutes
860	GCA_000006885.1_ASM688v1_genomic.fna	Streptococcus	Firmicutes
861	GCA_000158275.2_ASM15827v2_genomic.fna	Fusobacterium	Fusobacteria
862	GCA_000008165.1_ASM816v1_genomic.fna	Bacillus	Firmicutes
863	GCA_002024265.1_ASM202426v1_genomic.fna	Bacillus	Firmicutes
864	GCA_000009685.1_ASM968v1_genomic.fna	Clostridium	Firmicutes
865	GCA_001006005.1_ASM100600v1_genomic.fna	Serratia	Proteobacteria
866	GCA_000011465.1_ASM1146v1_genomic.fna	Prochlorococcus	Cyanobacteria
867	GCA_000007725.1_ASM772v1_genomic.fna	Buchnera	Proteobacteria
868	GCA_000009585.1_ASM958v1_genomic.fna	Staphylococcus	Firmicutes
869	GCA_000473995.1_ASM47399v1_genomic.fna	Clostridium	Firmicutes
870	GCA_000484505.1_ASM48450v1_genomic.fna	Clostridium	Firmicutes
871	GCA_000009005.1_ASM900v1_genomic.fna	Staphylococcus	Firmicutes
872	GCA_000011925.1_ASM1192v1_genomic.fna	Staphylococcus	Firmicutes
873	GCA_000009425.1_ASM942v1_genomic.fna	Lactococcus	Firmicutes
874	GCA_000008285.1_ASM828v1_genomic.fna	Listeria	Firmicutes
875	GCA_000008005.1_ASM800v1_genomic.fna	Bacillus	Firmicutes
876	GCA_000473245.1_ASM47324v1_genomic.fna	Bacillus	Firmicutes
877	GCA_000011625.1_ASM1162v1_genomic.fna	Bacillus	Firmicutes
878	GCA_000021185.1_ASM2118v1_genomic.fna	Listeria	Firmicutes
879	GCA_000014545.1_ASM1454v1_genomic.fna	Lactococcus	Firmicutes
880	GCA_000751035.1_PM221_genomic.fna	Staphylococcus	Firmicutes
881	GCA_000816265.1_ASM81626v1_genomic.fna	Campylobacter	Proteobacteria

882	GCA_000011865.1_ASM1186v1_genomic.fna	Campylobacter	Proteobacteria
883	GCA_900168365.1_JF4335_genomic.fna	Clostridium	Firmicutes
884	GCA_000508245.1_ASM50824v1_genomic.fna	Mycoplasma	Tenericutes
885	GCA_000496285.1_ASM49628v1_genomic.fna	Bacillus	Firmicutes
886	GCA_000477415.1_ASM47741v1_genomic.fna	Mycoplasma	Tenericutes
887	GCA_000512145.2_ASM51214v2_genomic.fna	Borrelia	Spirochaetes
888	GCA_001936255.1_ASM193625v1_genomic.fna	Borrelia	Spirochaetes
889	GCA_000019045.1_ASM1904v1_genomic.fna	Anoxybacillus	Firmicutes
890	GCA_000196455.1_ASM19645v1_genomic.fna	Acetoanaerobium	Firmicutes
891	GCA_000005825.2_ASM582v2_genomic.fna	Bacillus	Firmicutes
892	GCA_000298115.2_ASM29811v2_genomic.fna	Lactobacillus	Firmicutes
893	GCA_000025845.1_ASM2584v1_genomic.fna	Mycoplasma	Tenericutes
894	GCA_000021645.1_ASM2164v1_genomic.fna	Dictyoglomus	Dictyoglomi
895	GCA_001660005.1_ASM166000v1_genomic.fna	Borrelia	Spirochaetes
896	GCA_001038625.1_ASM103862v1_genomic.fna	Clostridium	Firmicutes
897	GCA_000020965.1_ASM2096v1_genomic.fna	Dictyoglomus	Dictyoglomi
898	GCA_000014325.1_ASM1432v1_genomic.fna	Streptococcus	Firmicutes
899	GCA_000014305.1_ASM1430v1_genomic.fna	Streptococcus	Firmicutes
900	GCA_900143575.1_LS9N_genomic.fna	Streptococcus	Firmicutes
901	GCA_000427055.1_ILRI112_genomic.fna	Streptococcus	Firmicutes
902	GCA_000636115.1_ASM63611v1_genomic.fna	Streptococcus	Firmicutes
903	GCA_001676785.2_ASM167678v2_genomic.fna	Brachyspira	Spirochaetes
904	GCA_000599965.1_ASM59996v1_genomic.fna	Streptococcus	Firmicutes
905	GCA_001190865.1_ASM119086v1_genomic.fna	Streptococcus	Firmicutes
906	GCA_002113805.1_ASM211380v1_genomic.fna	Bacillus	Firmicutes
907	GCA_000967445.1_ASM96744v1_genomic.fna	Streptococcus	Firmicutes
908	GCA_001908255.1_ASM190825v1_genomic.fna	Streptococcus	Firmicutes
909	GCA_001655175.1_ASM165517v1_genomic.fna	Streptococcus	Firmicutes
910	GCA_000382925.1_ASM38292v1_genomic.fna	Listeria	Firmicutes
911	GCA_001506325.1_ASM150632v1_genomic.fna	Campylobacter	Proteobacteria
912	GCA_000184085.1_ASM18408v1_genomic.fna	Campylobacter	Proteobacteria
913	GCA_000021525.1_ASM2152v1_genomic.fna	Rickettsia	Proteobacteria
914	GCA_000724585.1_ASM72458v1_genomic.fna	Bacillus	Firmicutes
915	GCA_000165505.1_ASM16550v1_genomic.fna	Ilyobacter	Fusobacteria

916	GCA_000210455.1_ASM21045v1_genomic.fna	Clostridioides	Firmicutes
917	GCA_000175295.2_ASM17529v2_genomic.fna	Thermoanaerobacte	Firmicutes
918	GCA_000210415.1_ASM21041v1_genomic.fna	Clostridioides	Firmicutes
919	GCA_001010825.1_ASM101082v1_genomic.fna	Clostridioides	Firmicutes
920	GCA_000211235.1_ASM21123v1_genomic.fna	Clostridioides	Firmicutes
921	GCA_001984465.1_ASM198446v1_genomic.fna	Clostridioides	Firmicutes
922	GCA_001447175.1_ASM144717v1_genomic.fna	Clostridioides	Firmicutes
923	GCA_000165465.1_ASM16546v1_genomic.fna	Halanaerobium	Firmicutes
924	GCA_000092245.1_ASM9224v1_genomic.fna	Arcobacter	Proteobacteria
925	GCA_000382945.1_ASM38294v1_genomic.fna	Listeria	Firmicutes
926	GCA_000025645.1_ASM2564v1_genomic.fna	Thermoanaerobacte	Firmicutes
927	GCA_000145615.1_ASM14561v1_genomic.fna	Thermoanaerobacte	Firmicutes
928	GCA_000145275.1_ASM14527v1_genomic.fna	Clostridium	Firmicutes
929	GCA_000092965.1_ASM9296v1_genomic.fna	Thermoanaerobacte	Firmicutes
930	GCA_001045995.2_ASM104599v2_genomic.fna	Staphylococcus	Firmicutes
931	GCA_001548585.1_ASM154858v1_genomic.fna	Listeria	Firmicutes
932	GCA_001506885.1_ASM150688v1_genomic.fna	Campylobacter	Proteobacteria
933	GCA_001506905.1_ASM150690v1_genomic.fna	Campylobacter	Proteobacteria
934	GCA_001548635.1_ASM154863v1_genomic.fna	Listeria	Firmicutes
935	GCA_001046095.2_ASM104609v2_genomic.fna	Staphylococcus	Firmicutes
936	GCA_001717665.1_ASM171766v1_genomic.fna	Staphylococcus	Firmicutes
937	GCA_001641045.1_ASM164104v1_genomic.fna	Staphylococcus	Firmicutes
938	GCA_001717705.1_ASM171770v1_genomic.fna	Staphylococcus	Firmicutes
939	GCA_001717685.1_ASM171768v1_genomic.fna	Staphylococcus	Firmicutes
940	GCA_000463355.1_ASM46335v1_genomic.fna	Streptococcus	Firmicutes
941	GCA_000709475.1_ASM70947v1_genomic.fna	Staphylococcus	Firmicutes
942	GCA_000829015.1_ASM82901v1_genomic.fna	Clostridium	Firmicutes
943	GCA_001506925.1_ASM150692v1_genomic.fna	Campylobacter	Proteobacteria
944	GCA_001889285.1_ASM188928v1_genomic.fna	Bacillus	Firmicutes
945	GCA_001506945.1_ASM150694v1_genomic.fna	Campylobacter	Proteobacteria
946	GCA_000009405.1_ASM940v1_genomic.fna	Staphylococcus	Firmicutes
947	GCA_000313635.1_ASM31363v1_genomic.fna	Mycoplasma	Tenericutes
948	GCA_001717975.1_ASM171797v1_genomic.fna	Staphylococcus	Firmicutes
949	GCA_001717725.1_ASM171772v1_genomic.fna	Staphylococcus	Firmicutes

950	GCA_900155335.1_NZ15MR0322_genomic.fna	Staphylococcus	Firmicutes
951	GCA_001551855.1_ASM155185v1_genomic.fna	Listeria	Firmicutes
952	GCA_001183705.1_ASM118370v1_genomic.fna	Staphylococcus	Firmicutes
953	GCA_001672035.1_ASM167203v1_genomic.fna	Lactobacillus	Firmicutes
954	GCA_002220695.1_ASM222069v1_genomic.fna	Serratia	Proteobacteria
955	GCA_002007625.1_ASM200762v1_genomic.fna	Enterococcus	Firmicutes
956	GCA_001596775.2_ASM159677v2_genomic.fna	Listeria	Firmicutes
957	GCA_001183725.1_ASM118372v1_genomic.fna	Staphylococcus	Firmicutes
958	GCA_001272915.1_ASM127291v1_genomic.fna	Mycoplasma	Tenericutes
959	GCA_002220715.1_ASM222071v1_genomic.fna	Serratia	Proteobacteria
960	GCA_001704315.1_ASM170431v1_genomic.fna	Lactobacillus	Firmicutes
961	GCA_001278745.1_ASM127874v1_genomic.fna	Staphylococcus	Firmicutes
962	GCA_001509195.1_ASM150919v1_genomic.fna	Mycoplasma	Tenericutes
963	GCA_001644625.1_ASM164462v1_genomic.fna	Listeria	Firmicutes
964	GCA_001687745.1_ASM168774v1_genomic.fna	Bacillus	Firmicutes
965	GCA_001723585.1_ASM172358v1_genomic.fna	Bacillus	Firmicutes
966	GCA_001281145.1_ASM128114v1_genomic.fna	Staphylococcus	Firmicutes
967	GCA_001879605.1_ASM187960v1_genomic.fna	Clostridium	Firmicutes
968	GCA_001908335.1_ASM190833v1_genomic.fna	Listeria	Firmicutes
969	GCA_000772205.1_ASM77220v1_genomic.fna	Bacillus	Firmicutes
970	GCA_000833335.1_ASM83333v1_genomic.fna	Francisella	Proteobacteria
971	GCA_001506965.1_ASM150696v1_genomic.fna	Campylobacter	Proteobacteria
972	GCA_001506985.1_ASM150698v1_genomic.fna	Campylobacter	Proteobacteria
973	GCA_002024245.1_ASM202424v1_genomic.fna	Enterococcus	Firmicutes
974	GCA_001922005.1_ASM192200v1_genomic.fna	Bacillus	Firmicutes
975	GCA_001448985.1_ASM144898v1_genomic.fna	Streptococcus	Firmicutes
976	GCA_001296985.1_ASM129698v1_genomic.fna	Staphylococcus	Firmicutes
977	GCA_001272635.1_ASM127263v1_genomic.fna	Streptococcus	Firmicutes
978	GCA_001913195.1_ASM191319v1_genomic.fna	Listeria	Firmicutes
979	GCA_000833085.1_ASM83308v1_genomic.fna	Bacillus	Firmicutes
980	GCA_001298325.2_ASM129832v2_genomic.fna	Staphylococcus	Firmicutes
981	GCA_000183285.1_ASM18328v1_genomic.fna	Buchnera	Proteobacteria
982	GCA_001952735.1_ASM195273v1_genomic.fna	Listeria	Firmicutes
983	GCA_000307005.1_ASM30700v1_genomic.fna	Listeria	Firmicutes

984	GCA_002078475.1_ASM207847v1_genomic.fna	Lactococcus	Firmicutes
985	GCA_001507025.1_ASM150702v1_genomic.fna	Campylobacter	Proteobacteria
986	GCA_000832665.1_ASM83266v1_genomic.fna	Bacillus	Firmicutes
987	GCA_000211015.1_ASM21101v1_genomic.fna	Streptococcus	Firmicutes
988	GCA_001507005.1_ASM150700v1_genomic.fna	Campylobacter	Proteobacteria
989	GCA_000732425.1_ASM73242v1_genomic.fna	Streptococcus	Firmicutes
990	GCA_000145595.1_ASM14559v1_genomic.fna	Staphylococcus	Firmicutes
991	GCA_000159535.2_ASM15953v2_genomic.fna	Staphylococcus	Firmicutes
992	GCA_000772225.1_ASM77222v1_genomic.fna	Campylobacter	Proteobacteria
993	GCA_000832845.1_ASM83284v1_genomic.fna	Bacillus	Firmicutes
994	GCA_001307235.1_ASM130723v1_genomic.fna	Staphylococcus	Firmicutes
995	GCA_002078495.1_ASM207849v1_genomic.fna	Lactococcus	Firmicutes
996	GCA_001952755.1_ASM195275v1_genomic.fna	Listeria	Firmicutes
997	GCA_000737085.1_ASM73708v1_genomic.fna	Campylobacter	Proteobacteria
998	GCA_000832805.1_ASM83280v1_genomic.fna	Bacillus	Firmicutes
999	GCA_000183305.1_ASM18330v1_genomic.fna	Buchnera	Proteobacteria
1000	GCA_000743015.1_ASM74301v1_genomic.fna	Streptococcus	Firmicutes
1001	GCA_000211035.2_ASM21103v2_genomic.fna	Streptococcus	Firmicutes
1002	GCA_000832725.1_ASM83272v1_genomic.fna	Bacillus	Firmicutes
1003	GCA_001572685.1_ASM157268v1_genomic.fna	Streptococcus	Firmicutes
1004	GCA_000307025.1_ASM30702v1_genomic.fna	Listeria	Firmicutes
1005	GCA_000835025.1_ASM83502v1_genomic.fna	Bacillus	Firmicutes
1006	GCA_001552035.1_ASM155203v1_genomic.fna	Streptococcus	Firmicutes
1007	GCA_001879625.1_ASM187962v1_genomic.fna	Clostridium	Firmicutes
1008	GCA_002173635.1_ASM217363v1_genomic.fna	Bacillus	Firmicutes
1009	GCA_002025045.1_ASM202504v1_genomic.fna	Enterococcus	Firmicutes
1010	GCA_000833355.1_ASM83335v1_genomic.fna	Francisella	Proteobacteria
1011	GCA_000782835.1_ASM78283v1_genomic.fna	Bacillus	Firmicutes
1012	GCA_000015785.1_ASM1578v1_genomic.fna	Bacillus	Firmicutes
1013	GCA_001314245.2_ASM131424v2_genomic.fna	Lactobacillus	Firmicutes
1014	GCA_000968375.1_ASM96837v1_genomic.fna	Clostridium	Firmicutes
1015	GCA_001314945.1_ASM131494v1_genomic.fna	Lactobacillus	Firmicutes
1016	GCA_000695835.1_ASM69583v1_genomic.fna	Mycoplasma	Tenericutes
1017	GCA_000706725.1_ASM70672v1_genomic.fna	Bacillus	Firmicutes

1018	GCA_001442815.1_ASM144281v1_genomic.fna	Staphylococcus	Firmicutes
1019	GCA_000493735.1_ASM49373v1_genomic.fna	Carnobacterium	Firmicutes
1020	GCA_000600105.1_ASM60010v1_genomic.fna	Lysinibacillus	Firmicutes
1021	GCA_001443645.1_ASM144364v1_genomic.fna	Lactobacillus	Firmicutes
1022	GCA_000785105.2_ASM78510v2_genomic.fna	Lactobacillus	Firmicutes
1023	GCA_001857925.1_ASM185792v1_genomic.fna	Bacillus	Firmicutes
1024	GCA_001187595.1_ASM118759v1_genomic.fna	Anoxybacillus	Firmicutes
1025	GCA_000763575.1_ASM76357v1_genomic.fna	Thermoanaerobacte	Firmicutes
1026	GCA_001455085.1_ASM145508v1_genomic.fna	Fusobacterium	Fusobacteria
1027	GCA_000789395.1_ASM78939v1_genomic.fna	Clostridium	Firmicutes
1028	GCA_000224985.1_ASM22498v1_genomic.fna	Lactobacillus	Firmicutes
1029	GCA_001936235.1_ASM193623v1_genomic.fna	Lactobacillus	Firmicutes
1030	GCA_000163895.2_ASM16389v2_genomic.fna	Filifactor	Firmicutes
1031	GCA_000014525.1_ASM1452v1_genomic.fna	Lactobacillus	Firmicutes
1032	GCA_001028645.1_ASM102864v1_genomic.fna	Staphylococcus	Firmicutes
1033	GCA_001190745.1_ASM119074v1_genomic.fna	Campylobacter	Proteobacteria
1034	GCA_000332735.1_ASM33273v1_genomic.fna	Staphylococcus	Firmicutes
1035	GCA_002356455.1_ASM235645v1_genomic.fna	Fusobacterium	Fusobacteria
1036	GCA_000270085.1_ASM27008v1_genomic.fna	Erysipelothrix	Firmicutes
1037	GCA_002075105.1_ASM207510v1_genomic.fna	Lactobacillus	Firmicutes
1038	GCA_002173495.1_ASM217349v1_genomic.fna	Bacillus	Firmicutes
1039	GCA_000177235.2_ASM17723v2_genomic.fna	Bacillus	Firmicutes
1040	GCA_000253155.1_ASM25315v1_genomic.fna	Streptococcus	Firmicutes
1041	GCA_001543285.1_ASM154328v1_genomic.fna	Aerococcus	Firmicutes
1042	GCA_000019085.1_ASM1908v1_genomic.fna	Thermoanaerobacte	Firmicutes
1043	GCA_001507045.1_ASM150704v1_genomic.fna	Campylobacter	Proteobacteria
1044	GCA_001507065.1_ASM150706v1_genomic.fna	Campylobacter	Proteobacteria
1045	GCA_001952775.1_ASM195277v1_genomic.fna	Listeria	Firmicutes
1046	GCA_001444345.1_ASM144434v1_genomic.fna	Staphylococcus	Firmicutes
1047	GCA_001507085.1_ASM150708v1_genomic.fna	Campylobacter	Proteobacteria
1048	GCA_001050115.1_ASM105011v1_genomic.fna	Bacillus	Firmicutes
1049	GCA_001456215.1_ASM145621v1_genomic.fna	Staphylococcus	Firmicutes
1050	GCA_001952795.1_ASM195279v1_genomic.fna	Listeria	Firmicutes
1051	GCA_001558175.1_ASM155817v1_genomic.fna	Mycoplasma	Tenericutes

1052	GCA_001767275.1_ASM176727v1_genomic.fna	Pediococcus	Firmicutes
1053	GCA_001507105.1_ASM150710v1_genomic.fna	Campylobacter	Proteobacteria
1054	GCA_001998915.1_ASM199891v1_genomic.fna	Listeria	Firmicutes
1055	GCA_001457495.1_NCTC13435_genomic.fna	Staphylococcus	Firmicutes
1056	GCA_001507125.1_ASM150712v1_genomic.fna	Campylobacter	Proteobacteria
1057	GCA_001507145.1_ASM150714v1_genomic.fna	Campylobacter	Proteobacteria
1058	GCA_001704335.1_ASM170433v1_genomic.fna	Lactobacillus	Firmicutes
1059	GCA_900029885.1_Sm_SMB2099_genomic.fna	Serratia	Proteobacteria
1060	GCA_001998945.1_ASM199894v1_genomic.fna	Listeria	Firmicutes
1061	GCA_001752685.1_ASM175268v1_genomic.fna	Bacillus	Firmicutes
1062	GCA_001854345.1_ASM185434v1_genomic.fna	Bacillus	Firmicutes
1063	GCA_001465635.1_ASM146563v1_genomic.fna	Staphylococcus	Firmicutes
1064	GCA_002209305.1_ASM220930v1_genomic.fna	Bacillus	Firmicutes
1065	GCA_001886775.1_ASM188677v1_genomic.fna	Clostridium	Firmicutes
1066	GCA_000789275.1_ASM78927v1_genomic.fna	Bacillus	Firmicutes
1067	GCA_001715615.1_ASM171561v1_genomic.fna	Lactobacillus	Firmicutes
1068	GCA_001998985.1_ASM199898v1_genomic.fna	Listeria	Firmicutes
1069	GCA_001465675.1_ASM146567v1_genomic.fna	Staphylococcus	Firmicutes
1070	GCA_001901705.1_ASM190170v1_genomic.fna	Mycoplasma	Tenericutes
1071	GCA_002025065.1_ASM202506v1_genomic.fna	Enterococcus	Firmicutes
1072	GCA_001507165.1_ASM150716v1_genomic.fna	Campylobacter	Proteobacteria
1073	GCA_000186405.1_ASM18640v1_genomic.fna	Streptococcus	Firmicutes
1074	GCA_001507185.1_ASM150718v1_genomic.fna	Campylobacter	Proteobacteria
1075	GCA_000427215.1_ASM42721v1_genomic.fna	Mycoplasma	Tenericutes
1076	GCA_001999025.1_ASM199902v1_genomic.fna	Listeria	Firmicutes
1077	GCA_001592385.1_ASM159238v1_genomic.fna	Streptococcus	Firmicutes
1078	GCA_001515665.1_ASM151566v1_genomic.fna	Staphylococcus	Firmicutes
1079	GCA_000833375.1_ASM83337v1_genomic.fna	Francisella	Proteobacteria
1080	GCA_001999005.1_ASM199900v1_genomic.fna	Listeria	Firmicutes
1081	GCA_000940785.1_ASM94078v1_genomic.fna	Bacillus	Firmicutes
1082	GCA_001465755.1_ASM146575v1_genomic.fna	Staphylococcus	Firmicutes
1083	GCA_000756485.1_ASM75648v1_genomic.fna	Streptococcus	Firmicutes
1084	GCA_001634675.1_ASM163467v1_genomic.fna	Bacillus	Firmicutes
1085	GCA_000258885.1_ASM25888v1_genomic.fna	Bacillus	Firmicutes

1086	GCA_000019005.1_ASM1900v1_genomic.fna	Streptococcus	Firmicutes
1087	GCA_000011665.1_ASM1166v1_genomic.fna	Streptococcus	Firmicutes
1088	GCA_000521525.1_ASM52152v1_genomic.fna	Buchnera	Proteobacteria
1089	GCA_001610935.1_ASM161093v1_genomic.fna	Streptococcus	Firmicutes
1090	GCA_000832745.1_ASM83274v1_genomic.fna	Bacillus	Firmicutes
1091	GCA_000211055.2_ASM21105v2_genomic.fna	Streptococcus	Firmicutes
1092	GCA_001296085.1_ASM129608v1_genomic.fna	Fusobacterium	Fusobacteria
1093	GCA_001507205.1_ASM150720v1_genomic.fna	Campylobacter	Proteobacteria
1094	GCA_000160335.2_ASM16033v2_genomic.fna	Staphylococcus	Firmicutes
1095	GCA_001515685.1_ASM151568v1_genomic.fna	Staphylococcus	Firmicutes
1096	GCA_000015645.1_ASM1564v1_genomic.fna	Prochlorococcus	Cyanobacteria
1097	GCA_000807355.1_ASM80735v1_genomic.fna	Campylobacter	Proteobacteria
1098	GCA_001515705.1_ASM151570v1_genomic.fna	Staphylococcus	Firmicutes
1099	GCA_000830775.1_ASM83077v1_genomic.fna	Campylobacter	Proteobacteria
1100	GCA_001507225.1_ASM150722v1_genomic.fna	Campylobacter	Proteobacteria
1101	GCA_001579785.1_ASM157978v1_genomic.fna	Clostridium	Firmicutes
1102	GCA_000093125.2_ASM9312v2_genomic.fna	Listeria	Firmicutes
1103	GCA_000204665.1_ASM20466v1_genomic.fna	Staphylococcus	Firmicutes
1104	GCA_001999065.1_ASM199906v1_genomic.fna	Listeria	Firmicutes
1105	GCA_000217635.1_ASM21763v1_genomic.fna	Buchnera	Proteobacteria
1106	GCA_000307045.1_ASM30704v1_genomic.fna	Listeria	Firmicutes
1107	GCA_002078615.1_ASM207861v1_genomic.fna	Lactococcus	Firmicutes
1108	GCA_000832865.1_ASM83286v1_genomic.fna	Bacillus	Firmicutes
1109	GCA_001999045.1_ASM199904v1_genomic.fna	Listeria	Firmicutes
1110	GCA_000307065.1_ASM30706v1_genomic.fna	Listeria	Firmicutes
1111	GCA_000017905.1_ASM1790v1_genomic.fna	Campylobacter	Proteobacteria
1112	GCA_002201975.1_ASM220197v1_genomic.fna	Bacillus	Firmicutes
1113	GCA_002201935.1_ASM220193v1_genomic.fna	Bacillus	Firmicutes
1114	GCA_000521545.1_ASM52154v1_genomic.fna	Buchnera	Proteobacteria
1115	GCA_000025425.1_ASM2542v1_genomic.fna	Campylobacter	Proteobacteria
1116	GCA_000767505.1_ASM76750v1_genomic.fna	Streptococcus	Firmicutes
1117	GCA_000021225.1_ASM2122v1_genomic.fna	Bacillus	Firmicutes
1118	GCA_000192705.1_ASM19270v1_genomic.fna	Lactococcus	Firmicutes
1119	GCA_000833045.1_ASM83304v1_genomic.fna	Bacillus	Firmicutes

1120	GCA_002085695.1_ASM208569v1_genomic.fna	Staphylococcus	Firmicutes
1121	GCA_002078765.1_ASM207876v1_genomic.fna	Lactococcus	Firmicutes
1122	GCA_000010445.1_ASM1044v1_genomic.fna	Staphylococcus	Firmicutes
1123	GCA_000010465.1_ASM1046v1_genomic.fna	Staphylococcus	Firmicutes
1124	GCA_000236475.1_ASM23647v1_genomic.fna	Lactococcus	Firmicutes
1125	GCA_002215535.1_ASM221553v1_genomic.fna	Staphylococcus	Firmicutes
1126	GCA_001592425.1_ASM159242v1_genomic.fna	Streptococcus	Firmicutes
1127	GCA_000021305.1_ASM2130v1_genomic.fna	Bacillus	Firmicutes
1128	GCA_001854085.1_ASM185408v1_genomic.fna	Clostridium	Firmicutes
1129	GCA_000168575.2_ASM16857v2_genomic.fna	Listeria	Firmicutes
1130	GCA_001017635.1_ASM101763v1_genomic.fna	Bacillus	Firmicutes
1131	GCA_001640765.1_ASM164076v1_genomic.fna	Streptococcus	Firmicutes
1132	GCA_000211075.1_ASM21107v1_genomic.fna	Streptococcus	Firmicutes
1133	GCA_000832785.1_ASM83278v1_genomic.fna	Bacillus	Firmicutes
1134	GCA_000789295.1_ASM78929v1_genomic.fna	Bacillus	Firmicutes
1135	GCA_001889325.1_ASM188932v1_genomic.fna	Clostridium	Firmicutes
1136	GCA_000225445.1_ASM22544v1_genomic.fna	Buchnera	Proteobacteria
1137	GCA_002334625.1_ASM233462v1_genomic.fna	Enterococcus	Firmicutes
1138	GCA_000011765.2_ASM1176v2_genomic.fna	Streptococcus	Firmicutes
1139	GCA_002090215.1_ASM209021v1_genomic.fna	Mycoplasma	Tenericutes
1140	GCA_000833475.1_ASM83347v1_genomic.fna	Francisella	Proteobacteria
1141	GCA_000015665.1_ASM1566v1_genomic.fna	Prochlorococcus	Cyanobacteria
1142	GCA_000512835.2_ASM51283v2_genomic.fna	Bacillus	Firmicutes
1143	GCA_001857985.1_ASM185798v1_genomic.fna	Bacillus	Firmicutes
1144	GCA_002005345.1_ASM200534v1_genomic.fna	Bacillus	Firmicutes
1145	GCA_002257505.1_ASM225750v1_genomic.fna	Mycoplasma	Tenericutes
1146	GCA_000204625.1_ASM20462v1_genomic.fna	Streptococcus	Firmicutes
1147	GCA_001296145.1_ASM129614v1_genomic.fna	Fusobacterium	Fusobacteria
1148	GCA_001874125.1_ASM187412v1_genomic.fna	Lactobacillus	Firmicutes
1149	GCA_000019025.1_ASM1902v1_genomic.fna	Streptococcus	Firmicutes
1150	GCA_002074115.1_ASM207411v1_genomic.fna	Bacillus	Firmicutes
1151	GCA_000698885.1_ASM69888v1_genomic.fna	Streptococcus	Firmicutes
1152	GCA_001046835.1_ASM104683v1_genomic.fna	Lactobacillus	Firmicutes
1153	GCA_001431785.1_ASM143178v1_genomic.fna	Bacillus	Firmicutes

1154	GCA_000427075.1_ILRI005_genomic.fna	Streptococcus	Firmicutes
1155	GCA_000292455.1_ASM29245v1_genomic.fna	Bacillus	Firmicutes
1156	GCA_000815025.1_ASM81502v1_genomic.fna	Coxiella	Proteobacteria
1157	GCA_000808095.1_ASM80809v1_genomic.fna	Borreliella	Spirochaetes
1158	GCA_001888925.1_ASM188892v1_genomic.fna	Lactobacillus	Firmicutes
1159	GCA_002009215.1_ASM200921v1_genomic.fna	Mycoplasma	Tenericutes
1160	GCA_000814265.1_ASM81426v1_genomic.fna	Campylobacter	Proteobacteria
1161	GCA_002073835.1_ASM207383v1_genomic.fna	Streptococcus	Firmicutes
1162	GCA_000016105.1_ASM1610v1_genomic.fna	Francisella	Proteobacteria
1163	GCA_000815225.1_ASM81522v1_genomic.fna	Allofrancisella	Proteobacteria
1164	GCA_002074155.1_ASM207415v1_genomic.fna	Clostridium	Firmicutes
1165	GCA_000816785.1_ASM81678v1_genomic.fna	Campylobacter	Proteobacteria
1166	GCA_000831065.1_ASM83106v1_genomic.fna	Bacillus	Firmicutes
1167	GCA_000828855.1_ASM82885v1_genomic.fna	Mycoplasma	Tenericutes
1168	GCA_000831645.3_ASM83164v3_genomic.fna	Lactobacillus	Firmicutes
1169	GCA_000829395.1_ASM82939v1_genomic.fna	Lactobacillus	Firmicutes
1170	GCA_000736415.1_ASM73641v1_genomic.fna	Campylobacter	Proteobacteria
1171	GCA_000941075.1_ASM94107v1_genomic.fna	Mycoplasma	Tenericutes
1172	GCA_001042715.1_ASM104271v1_genomic.fna	Clostridium	Firmicutes
1173	GCA_000953715.1_DTL3_genomic.fna	Defluviitoga	Thermotogae
1174	GCA_000973545.1_ASM97354v1_genomic.fna	Blochmannia	Proteobacteria
1175	GCA_000973505.1_ASM97350v1_genomic.fna	Blochmannia	Proteobacteria
1176	GCA_001902315.1_ASM190231v1_genomic.fna	Staphylococcus	Firmicutes
1177	GCA_000236925.1_ASM23692v1_genomic.fna	Staphylococcus	Firmicutes
1178	GCA_000577895.1_M2_40_genomic.fna	Clostridium	Firmicutes
1179	GCA_001618885.1_ASM161888v1_genomic.fna	Staphylococcus	Firmicutes
1180	GCA_000965765.1_ASM96576v1_genomic.fna	Mycoplasma	Tenericutes
1181	GCA_001999105.1_ASM199910v1_genomic.fna	Listeria	Firmicutes
1182	GCA_001515765.1_ASM151576v1_genomic.fna	Staphylococcus	Firmicutes
1183	GCA_001515745.1_ASM151574v1_genomic.fna	Staphylococcus	Firmicutes
1184	GCA_001999085.1_ASM199908v1_genomic.fna	Listeria	Firmicutes
1185	GCA_001507245.1_ASM150724v1_genomic.fna	Campylobacter	Proteobacteria
1186	GCA_001507265.1_ASM150726v1_genomic.fna	Campylobacter	Proteobacteria
1187	GCA_001880185.1_ASM188018v1_genomic.fna	Lactobacillus	Firmicutes

1188	GCA_001548295.1_ASM154829v1_genomic.fna	Staphylococcus	Firmicutes
1189	GCA_001999125.1_ASM199912v1_genomic.fna	Listeria	Firmicutes
1190	GCA_001563565.1_ASM156356v1_genomic.fna	Campylobacter	Proteobacteria
1191	GCA_001587015.1_ASM158701v1_genomic.fna	Campylobacter	Proteobacteria
1192	GCA_001999145.1_ASM199914v1_genomic.fna	Listeria	Firmicutes
1193	GCA_001548415.1_ASM154841v1_genomic.fna	Staphylococcus	Firmicutes
1194	GCA_000833495.1_ASM83349v1_genomic.fna	Francisella	Proteobacteria
1195	GCA_001999165.1_ASM199916v1_genomic.fna	Listeria	Firmicutes
1196	GCA_002090235.1_ASM209023v1_genomic.fna	Mycoplasma	Tenericutes
1197	GCA_002442955.1_ASM244295v1_genomic.fna	Enterococcus	Firmicutes
1198	GCA_001889345.1_ASM188934v1_genomic.fna	Clostridium	Firmicutes
1199	GCA_000827065.1_ASM82706v1_genomic.fna	Bacillus	Firmicutes
1200	GCA_000832965.1_ASM83296v1_genomic.fna	Bacillus	Firmicutes
1201	GCA_001683515.1_ASM168351v1_genomic.fna	Streptococcus	Firmicutes
1202	GCA_000211095.1_ASM21109v1_genomic.fna	Streptococcus	Firmicutes
1203	GCA_001587035.1_ASM158703v1_genomic.fna	Campylobacter	Proteobacteria
1204	GCA_001182785.1_ASM118278v1_genomic.fna	Bacillus	Firmicutes
1205	GCA_001549655.1_ASM154965v1_genomic.fna	Staphylococcus	Firmicutes
1206	GCA_001686905.1_ASM168690v1_genomic.fna	Campylobacter	Proteobacteria
1207	GCA_001549675.1_ASM154967v1_genomic.fna	Staphylococcus	Firmicutes
1208	GCA_001721625.1_ASM172162v1_genomic.fna	Streptococcus	Firmicutes
1209	GCA_002043045.1_ASM204304v1_genomic.fna	Listeria	Firmicutes
1210	GCA_000307085.1_ASM30708v1_genomic.fna	Listeria	Firmicutes
1211	GCA_002007885.1_ASM200788v1_genomic.fna	Clostridioides	Firmicutes
1212	GCA_002078855.1_ASM207885v1_genomic.fna	Lactococcus	Firmicutes
1213	GCA_000367725.3_Z3055_genomic.fna	Coxiella	Proteobacteria
1214	GCA_000835185.1_ASM83518v1_genomic.fna	Bacillus	Firmicutes
1215	GCA_001558795.1_ASM155879v1_genomic.fna	Staphylococcus	Firmicutes
1216	GCA_000293765.1_ASM29376v1_genomic.fna	Bacillus	Firmicutes
1217	GCA_002101275.1_ASM210127v1_genomic.fna	Listeria	Firmicutes
1218	GCA_000772185.1_ASM77218v1_genomic.fna	Streptococcus	Firmicutes
1219	GCA_000521585.1_ASM52158v1_genomic.fna	Buchnera	Proteobacteria
1220	GCA_002105615.1_ASM210561v1_genomic.fna	Listeria	Firmicutes
1221	GCA_000978375.1_ASM97837v1_genomic.fna	Bacillus	Firmicutes

1222	GCA_002078895.1_ASM207889v1_genomic.fna	Lactococcus	Firmicutes
1223	GCA_000277265.1_ASM27726v1_genomic.fna	Rickettsia	Proteobacteria
1224	GCA_000307615.1_ASM30761v1_genomic.fna	Listeria	Firmicutes
1225	GCA_000019305.1_ASM1930v1_genomic.fna	Clostridium	Firmicutes
1226	GCA_000418515.1_ASM41851v1_genomic.fna	Lactobacillus	Firmicutes
1227	GCA_000210315.1_ASM21031v1_genomic.fna	Staphylococcus	Firmicutes
1228	GCA_001717625.1_ASM171762v1_genomic.fna	Campylobacter	Proteobacteria
1229	GCA_000961015.1_ASM96101v1_genomic.fna	Lactobacillus	Firmicutes
1230	GCA_000283955.1_ASM28395v1_genomic.fna	Rickettsia	Proteobacteria
1231	GCA_000830805.1_ASM83080v1_genomic.fna	Campylobacter	Proteobacteria
1232	GCA_000830825.1_ASM83082v1_genomic.fna	Campylobacter	Proteobacteria
1233	GCA_001721945.1_ASM172194v1_genomic.fna	Campylobacter	Proteobacteria
1234	GCA_002119645.1_ASM211964v1_genomic.fna	Lactobacillus	Firmicutes
1235	GCA_001267815.1_ASM126781v1_genomic.fna	Mycoplasma	Tenericutes
1236	GCA_000237125.1_ASM23712v1_genomic.fna	Staphylococcus	Firmicutes
1237	GCA_001580495.1_ASM158049v1_genomic.fna	Staphylococcus	Firmicutes
1238	GCA_000251085.2_ASM25108v2_genomic.fna	Streptococcus	Firmicutes
1239	GCA_002105635.1_ASM210563v1_genomic.fna	Listeria	Firmicutes
1240	GCA_000833065.1_ASM83306v1_genomic.fna	Bacillus	Firmicutes
1241	GCA_001936415.1_ASM193641v1_genomic.fna	Streptococcus	Firmicutes
1242	GCA_001580515.1_ASM158051v1_genomic.fna	Staphylococcus	Firmicutes
1243	GCA_000494835.1_ASM49483v1_genomic.fna	Bacillus	Firmicutes
1244	GCA_001712835.1_ASM171283v1_genomic.fna	Streptococcus	Firmicutes
1245	GCA_001721985.1_ASM172198v1_genomic.fna	Campylobacter	Proteobacteria
1246	GCA_001721965.1_ASM172196v1_genomic.fna	Campylobacter	Proteobacteria
1247	GCA_002151485.1_ASM215148v1_genomic.fna	Borreliella	Spirochaetes
1248	GCA_000772245.1_ASM77224v1_genomic.fna	Streptococcus	Firmicutes
1249	GCA_000550745.1_ASM55074v1_genomic.fna	Enterococcus	Firmicutes
1250	GCA_000833455.1_ASM83345v1_genomic.fna	Francisella	Proteobacteria
1251	GCA_001721925.1_ASM172192v1_genomic.fna	Lactobacillus	Firmicutes
1252	GCA_000833515.1_ASM83351v1_genomic.fna	Francisella	Proteobacteria
1253	GCA_002009195.1_ASM200919v1_genomic.fna	Bacillus	Firmicutes
1254	GCA_000953615.1_BS49Ch_genomic.fna	Bacillus	Firmicutes
1255	GCA_001412695.1_ASM141269v1_genomic.fna	Enterococcus	Firmicutes

1256	GCA_001594205.1_ASM159420v1_genomic.fna	Staphylococcus	Firmicutes
1257	GCA_001183785.1_ASM118378v1_genomic.fna	Bacillus	Firmicutes
1258	GCA_002105655.1_ASM210565v1_genomic.fna	Listeria	Firmicutes
1259	GCA_002090275.1_ASM209027v1_genomic.fna	Mycoplasma	Tenericutes
1260	GCA_001908455.1_ASM190845v1_genomic.fna	Lactobacillus	Firmicutes
1261	GCA_001891125.1_ASM189112v1_genomic.fna	Leuconostoc	Firmicutes
1262	GCA_001294565.1_ASM129456v1_genomic.fna	Serratia	Proteobacteria
1263	GCA_000604105.1_ASM60410v1_genomic.fna	Lactobacillus	Firmicutes
1264	GCA_002057535.1_ASM205753v1_genomic.fna	Bacillus	Firmicutes
1265	GCA_002072695.1_ASM207269v1_genomic.fna	Bacillus	Firmicutes
1266	GCA_002117375.1_ASM211737v1_genomic.fna	Lactobacillus	Firmicutes
1267	GCA_900066025.1_Enterococcus_faecium_E1002_isolate_genomic.fna	Enterococcus	Firmicutes
1268	GCA_001611325.1_ASM161132v1_genomic.fna	Staphylococcus	Firmicutes
1269	GCA_002105675.1_ASM210567v1_genomic.fna	Listeria	Firmicutes
1270	GCA_000387745.2_ASM38774v2_genomic.fna	Mycoplasma	Tenericutes
1271	GCA_001017575.1_ASM101757v1_genomic.fna	Campylobacter	Proteobacteria
1272	GCA_002090295.1_ASM209029v1_genomic.fna	Mycoplasma	Tenericutes
1273	GCA_001417635.1_ASM141763v1_genomic.fna	Campylobacter	Proteobacteria
1274	GCA_900092475.1_Ef_aus00233_genomic.fna	Enterococcus	Firmicutes
1275	GCA_900094615.1_ASM90009461v1_genomic.fna	Lactobacillus	Firmicutes
1276	GCA_000971925.1_ASM97192v1_genomic.fna	Bacillus	Firmicutes
1277	GCA_900187195.1_51485_F01_genomic.fna	Listeria	Firmicutes
1278	GCA_000833535.1_ASM83353v1_genomic.fna	Francisella	Proteobacteria
1279	GCA_001990145.1_ASM199014v1_genomic.fna	Lactobacillus	Firmicutes
1280	GCA_000286715.1_ASM28671v1_genomic.fna	Mycoplasma	Tenericutes
1281	GCA_001611895.1_ASM161189v1_genomic.fna	Mycoplasma	Tenericutes
1282	GCA_001420855.1_ASM142085v1_genomic.fna	Bacillus	Firmicutes
1283	GCA_002176855.1_ASM217685v1_genomic.fna	Lactobacillus	Firmicutes
1284	GCA_001729925.1_ASM172992v1_genomic.fna	Streptococcus	Firmicutes
1285	GCA_001514415.1_ASM151441v1_genomic.fna	Lactobacillus	Firmicutes
1286	GCA_002111405.1_ASM211140v1_genomic.fna	Streptococcus	Firmicutes
1287	GCA_000833125.1_ASM83312v1_genomic.fna	Bacillus	Firmicutes
1288	GCA_002357195.1_ASM235719v1_genomic.fna	Rickettsia	Proteobacteria
1289	GCA_002407165.1_ASM240716v1_genomic.fna	Staphylococcus	Firmicutes

1290	GCA_000455585.1_ucmb5113v1_genomic.fna	Bacillus	Firmicutes
1291	GCA_001885275.1_ASM188527v1_genomic.fna	Francisella	Proteobacteria
1292	GCA_002214225.1_ASM221422v1_genomic.fna	Francisella	Proteobacteria
1293	GCA_002357215.1_ASM235721v1_genomic.fna	Rickettsia	Proteobacteria
1294	GCA_000493375.1_BAPNAU_WGS_genomic.fna	Bacillus	Firmicutes
1295	GCA_001042405.1_ASM104240v1_genomic.fna	Lactobacillus	Firmicutes
1296	GCA_001029245.1_ASM102924v1_genomic.fna	Spiroplasma	Tenericutes
1297	GCA_001050475.1_ASM105047v1_genomic.fna	Lactobacillus	Firmicutes
1298	GCA_001050435.1_ASM105043v1_genomic.fna	Lactobacillus	Firmicutes
1299	GCA_001188855.1_ASM118885v1_genomic.fna	Staphylococcus	Firmicutes
1300	GCA_001262715.1_ASM126271v1_genomic.fna	Spiroplasma	Tenericutes
1301	GCA_002240375.1_ASM224037v1_genomic.fna	Lactobacillus	Firmicutes
1302	GCA_002005405.1_ASM200540v1_genomic.fna	Jeotgalibaca	Firmicutes
1303	GCA_001267155.1_ASM126715v1_genomic.fna	Spiroplasma	Tenericutes
1304	GCA_001281045.1_ASM128104v1_genomic.fna	Spiroplasma	Tenericutes
1305	GCA_001936335.1_ASM193633v1_genomic.fna	Lactobacillus	Firmicutes
1306	GCA_001702175.1_ASM170217v1_genomic.fna	Lactobacillus	Firmicutes
1307	GCA_001702135.1_ASM170213v1_genomic.fna	Lactobacillus	Firmicutes
1308	GCA_000408885.1_ASM40888v1_genomic.fna	Bacillus	Firmicutes
1309	GCA_900183405.1_ASM90018340v1_genomic.fna	Lactobacillus	Firmicutes
1310	GCA_001922025.1_ASM192202v1_genomic.fna	Lactobacillus	Firmicutes
1311	GCA_001645685.2_ASM164568v2_genomic.fna	Bacillus	Firmicutes
1312	GCA_900093775.1_EVONIK_BGLY_genomic.fna	Bacillus	Firmicutes
1313	GCA_001465835.2_ASM146583v2_genomic.fna	Planococcus	Firmicutes
1314	GCA_001465795.2_ASM146579v2_genomic.fna	Planococcus	Firmicutes
1315	GCA_001767215.1_ASM176721v1_genomic.fna	Campylobacter	Proteobacteria
1316	GCA_001865395.1_ASM186539v1_genomic.fna	Campylobacter	Proteobacteria
1317	GCA_002105695.1_ASM210569v1_genomic.fna	Listeria	Firmicutes
1318	GCA_001611345.1_ASM161134v1_genomic.fna	Staphylococcus	Firmicutes
1319	GCA_001865435.1_ASM186543v1_genomic.fna	Campylobacter	Proteobacteria
1320	GCA_001865415.1_ASM186541v1_genomic.fna	Campylobacter	Proteobacteria
1321	GCA_002105715.1_ASM210571v1_genomic.fna	Listeria	Firmicutes
1322	GCA_001611365.1_ASM161136v1_genomic.fna	Staphylococcus	Firmicutes
1323	GCA_002213505.1_ASM221350v1_genomic.fna	Listeria	Firmicutes

1324	GCA_002208215.1_ASM220821v1_genomic.fna	Listeria	Firmicutes
1325	GCA_001611385.1_ASM161138v1_genomic.fna	Staphylococcus	Firmicutes
1326	GCA_000237265.1_ASM23726v1_genomic.fna	Staphylococcus	Firmicutes
1327	GCA_001277915.1_ASM127791v1_genomic.fna	Bacillus	Firmicutes
1328	GCA_000993765.1_ASM99376v1_genomic.fna	Streptococcus	Firmicutes
1329	GCA_001865595.1_ASM186559v1_genomic.fna	Campylobacter	Proteobacteria
1330	GCA_001611405.1_ASM161140v1_genomic.fna	Staphylococcus	Firmicutes
1331	GCA_000299015.1_ASM29901v1_genomic.fna	Streptococcus	Firmicutes
1332	GCA_001865615.1_ASM186561v1_genomic.fna	Campylobacter	Proteobacteria
1333	GCA_001611425.1_ASM161142v1_genomic.fna	Staphylococcus	Firmicutes
1334	GCA_000318055.1_ASM31805v1_genomic.fna	Listeria	Firmicutes
1335	GCA_002078915.1_ASM207891v1_genomic.fna	Lactococcus	Firmicutes
1336	GCA_002213545.1_ASM221354v1_genomic.fna	Listeria	Firmicutes
1337	GCA_001870105.1_ASM187010v1_genomic.fna	Campylobacter	Proteobacteria
1338	GCA_000438585.1_ASM43858v1_genomic.fna	Listeria	Firmicutes
1339	GCA_000830845.1_ASM83084v1_genomic.fna	Campylobacter	Proteobacteria
1340	GCA_000830865.1_ASM83086v1_genomic.fna	Campylobacter	Proteobacteria
1341	GCA_000239235.1_ASM23923v1_genomic.fna	Staphylococcus	Firmicutes
1342	GCA_002213565.1_ASM221356v1_genomic.fna	Listeria	Firmicutes
1343	GCA_001518875.1_ASM151887v1_genomic.fna	Bacillus	Firmicutes
1344	GCA_001870085.1_ASM187008v1_genomic.fna	Campylobacter	Proteobacteria
1345	GCA_002078935.1_ASM207893v1_genomic.fna	Lactococcus	Firmicutes
1346	GCA_001618305.1_ASM161830v1_genomic.fna	Staphylococcus	Firmicutes
1347	GCA_002213605.1_ASM221360v1_genomic.fna	Listeria	Firmicutes
1348	GCA_002213585.1_ASM221358v1_genomic.fna	Listeria	Firmicutes
1349	GCA_000817005.1_ASM81700v1_genomic.fna	Streptococcus	Firmicutes
1350	GCA_001640885.1_ASM164088v1_genomic.fna	Staphylococcus	Firmicutes
1351	GCA_001014285.1_ASM101428v1_genomic.fna	Streptococcus	Firmicutes
1352	GCA_000833275.1_ASM83327v1_genomic.fna	Bacillus	Firmicutes
1353	GCA_002189695.1_ASM218969v1_genomic.fna	Streptococcus	Firmicutes
1354	GCA_001932715.1_ASM193271v1_genomic.fna	Streptococcus	Firmicutes
1355	GCA_002213625.1_ASM221362v1_genomic.fna	Listeria	Firmicutes
1356	GCA_000493775.1_ASM49377v1_genomic.fna	Streptococcus	Firmicutes
1357	GCA_001455345.1_ASM145534v1_genomic.fna	Bacillus	Firmicutes

1358	GCA_002591215.1_ASM259121v1_genomic.fna	Staphylococcus	Firmicutes
1359	GCA_001640905.1_ASM164090v1_genomic.fna	Staphylococcus	Firmicutes
1360	GCA_001951255.1_ASM195125v1_genomic.fna	Campylobacter	Proteobacteria
1361	GCA_001951235.1_ASM195123v1_genomic.fna	Campylobacter	Proteobacteria
1362	GCA_000978785.2_ASM97878v2_genomic.fna	Francisella	Proteobacteria
1363	GCA_001640925.1_ASM164092v1_genomic.fna	Staphylococcus	Firmicutes
1364	GCA_002079285.1_ASM207928v1_genomic.fna	Lactobacillus	Firmicutes
1365	GCA_000973605.1_ASM97360v1_genomic.fna	Bacillus	Firmicutes
1366	GCA_002117165.1_ASM211716v1_genomic.fna	Bacillus	Firmicutes
1367	GCA_001641025.1_ASM164102v1_genomic.fna	Staphylococcus	Firmicutes
1368	GCA_001951295.1_ASM195129v1_genomic.fna	Campylobacter	Proteobacteria
1369	GCA_000286735.1_ASM28673v1_genomic.fna	Mycoplasma	Tenericutes
1370	GCA_002028365.1_ASM202836v1_genomic.fna	Lactobacillus	Firmicutes
1371	GCA_001951275.1_ASM195127v1_genomic.fna	Campylobacter	Proteobacteria
1372	GCA_001417865.2_ASM141786v2_genomic.fna	Serratia	Proteobacteria
1373	GCA_001656045.1_ASM165604v1_genomic.fna	Staphylococcus	Firmicutes
1374	GCA_002138395.1_ASM213839v1_genomic.fna	Lactobacillus	Firmicutes
1375	GCA_002095995.1_ASM209599v1_genomic.fna	Mycoplasma	Tenericutes
1376	GCA_002090315.1_ASM209031v1_genomic.fna	Mycoplasma	Tenericutes
1377	GCA_002024845.1_ASM202484v1_genomic.fna	Lactobacillus	Firmicutes
1378	GCA_001483845.1_ASM148384v1_genomic.fna	Campylobacter	Proteobacteria
1379	GCA_002105595.1_ASM210559v1_genomic.fna	Bacillus	Firmicutes
1380	GCA_002213645.1_ASM221364v1_genomic.fna	Listeria	Firmicutes
1381	GCA_001611935.1_ASM161193v1_genomic.fna	Mycoplasma	Tenericutes
1382	GCA_001548175.1_ASM154817v1_genomic.fna	Bacillus	Firmicutes
1383	GCA_001656075.1_ASM165607v1_genomic.fna	Staphylococcus	Firmicutes
1384	GCA_002213685.1_ASM221368v1_genomic.fna	Listeria	Firmicutes
1385	GCA_002009375.1_ASM200937v1_genomic.fna	Leuconostoc	Firmicutes
1386	GCA_002213665.1_ASM221366v1_genomic.fna	Listeria	Firmicutes
1387	GCA_000931425.1_ASM93142v1_genomic.fna	Lactobacillus	Firmicutes
1388	GCA_001015095.1_ASM101509v1_genomic.fna	Bacillus	Firmicutes
1389	GCA_001011135.1_ASM101113v1_genomic.fna	Francisella	Proteobacteria
1390	GCA_000733995.1_ASM73399v1_genomic.fna	Mycoplasma	Tenericutes
1391	GCA_000875715.1_ASM87571v1_genomic.fna	Bacillus	Firmicutes

1392	GCA_001014305.1_ASM101430v1_genomic.fna	Streptococcus	Firmicutes
1393	GCA_001518735.1_ASM151873v1_genomic.fna	Enterococcus	Firmicutes
1394	GCA_001255215.1_S_pneumo_A66_v1_genomic.fna	Streptococcus	Firmicutes
1395	GCA_000508265.1_ASM50826v1_genomic.fna	Bacillus	Firmicutes
1396	GCA_001951315.1_ASM195131v1_genomic.fna	Campylobacter	Proteobacteria
1397	GCA_001988935.1_ASM198893v1_genomic.fna	Lactobacillus	Firmicutes
1398	GCA_002025005.1_ASM202500v1_genomic.fna	Streptococcus	Firmicutes
1399	GCA_002688665.1_ASM268866v1_genomic.fna	Streptococcus	Firmicutes
1400	GCA_001951335.1_ASM195133v1_genomic.fna	Campylobacter	Proteobacteria
1401	GCA_000742975.1_ASM74297v1_genomic.fna	Enterococcus	Firmicutes
1402	GCA_000019545.1_ASM1954v1_genomic.fna	Clostridium	Firmicutes
1403	GCA_000245495.1_ASM24549v1_genomic.fna	Staphylococcus	Firmicutes
1404	GCA_000363905.1_ASM36390v1_genomic.fna	Rickettsia	Proteobacteria
1405	GCA_000835285.1_ASM83528v1_genomic.fna	Campylobacter	Proteobacteria
1406	GCA_000835305.1_ASM83530v1_genomic.fna	Campylobacter	Proteobacteria
1407	GCA_000253135.1_ASM25313v1_genomic.fna	Staphylococcus	Firmicutes
1408	GCA_001554055.1_ASM155405v1_genomic.fna	Mycoplasma	Tenericutes
1409	GCA_002192435.1_ASM219243v1_genomic.fna	Lactobacillus	Firmicutes
1410	GCA_000438605.1_ASM43860v1_genomic.fna	Listeria	Firmicutes
1411	GCA_002078955.1_ASM207895v1_genomic.fna	Lactococcus	Firmicutes
1412	GCA_001635915.1_ASM163591v1_genomic.fna	Bacillus	Firmicutes
1413	GCA_002151505.1_ASM215150v1_genomic.fna	Borreliella	Spirochaetes
1414	GCA_000829055.1_ASM82905v1_genomic.fna	Lactobacillus	Firmicutes
1415	GCA_000321395.1_ASM32139v1_genomic.fna	Bacillus	Firmicutes
1416	GCA_001572745.1_ASM157274v1_genomic.fna	Coxiella	Proteobacteria
1417	GCA_002073735.1_ASM207373v1_genomic.fna	Clostridioides	Firmicutes
1418	GCA_001019635.1_ASM101963v1_genomic.fna	Streptococcus	Firmicutes
1419	GCA_000967135.1_CFV_genomic.fna	Campylobacter	Proteobacteria
1420	GCA_001543225.1_ASM154322v1_genomic.fna	Bacillus	Firmicutes
1421	GCA_001457635.1_NCTC7465_genomic.fna	Streptococcus	Firmicutes
1422	GCA_001888945.1_ASM188894v1_genomic.fna	Lactobacillus	Firmicutes
1423	GCA_002736265.1_ASM273626v1_genomic.fna	Streptococcus	Firmicutes
1424	GCA_000438625.1_ASM43862v1_genomic.fna	Listeria	Firmicutes
1425	GCA_001635955.1_ASM163595v1_genomic.fna	Bacillus	Firmicutes

1426	GCA_000831525.1_ASM83152v1_genomic.fna	Rickettsia	Proteobacteria
1427	GCA_002078975.1_ASM207897v1_genomic.fna	Lactococcus	Firmicutes
1428	GCA_001006025.1_ASM100602v1_genomic.fna	Lactobacillus	Firmicutes
1429	GCA_002094955.1_ASM209495v1_genomic.fna	Streptococcus	Firmicutes
1430	GCA_000292705.1_ASM29270v1_genomic.fna	Bacillus	Firmicutes
1431	GCA_002025025.1_ASM202502v1_genomic.fna	Streptococcus	Firmicutes
1432	GCA_000017785.1_ASM1778v1_genomic.fna	Francisella	Proteobacteria
1433	GCA_001548215.1_ASM154821v1_genomic.fna	Bacillus	Firmicutes
1434	GCA_001618905.1_ASM161890v1_genomic.fna	Lactobacillus	Firmicutes
1435	GCA_000971665.1_ASM97166v1_genomic.fna	Streptococcus	Firmicutes
1436	GCA_002074135.1_ASM207413v1_genomic.fna	Bacillus	Firmicutes
1437	GCA_000689235.1_GBCO_p1_genomic.fna	Streptococcus	Firmicutes
1438	GCA_001296165.1_ASM129616v1_genomic.fna	Fusobacterium	Fusobacteria
1439	GCA_000583105.1_ASM58310v1_genomic.fna	Bacillus	Firmicutes
1440	GCA_000231865.1_ASM23186v1_genomic.fna	Streptococcus	Firmicutes
1441	GCA_002213705.1_ASM221370v1_genomic.fna	Listeria	Firmicutes
1442	GCA_001725965.1_ASM172596v1_genomic.fna	Staphylococcus	Firmicutes
1443	GCA_001880265.1_ASM188026v1_genomic.fna	Staphylococcus	Firmicutes
1444	GCA_002213745.1_ASM221374v1_genomic.fna	Listeria	Firmicutes
1445	GCA_002213725.1_ASM221372v1_genomic.fna	Listeria	Firmicutes
1446	GCA_002024325.1_ASM202432v1_genomic.fna	Campylobacter	Proteobacteria
1447	GCA_001879545.1_ASM187954v1_genomic.fna	Staphylococcus	Firmicutes
1448	GCA_002028305.1_ASM202830v1_genomic.fna	Campylobacter	Proteobacteria
1449	GCA_001887075.1_ASM188707v1_genomic.fna	Staphylococcus	Firmicutes
1450	GCA_002213765.1_ASM221376v1_genomic.fna	Listeria	Firmicutes
1451	GCA_001262115.1_ASM126211v1_genomic.fna	Francisella	Proteobacteria
1452	GCA_001037985.1_ASM103798v1_genomic.fna	Bacillus	Firmicutes
1453	GCA_002101355.1_ASM210135v1_genomic.fna	Campylobacter	Proteobacteria
1454	GCA_001900185.1_ASM190018v1_genomic.fna	Staphylococcus	Firmicutes
1455	GCA_002209005.1_ASM220900v1_genomic.fna	Campylobacter	Proteobacteria
1456	GCA_001595725.1_ASM159572v1_genomic.fna	Bacillus	Firmicutes
1457	GCA_002213785.1_ASM221378v1_genomic.fna	Listeria	Firmicutes
1458	GCA_002213825.1_ASM221382v1_genomic.fna	Listeria	Firmicutes
1459	GCA_002109425.1_ASM210942v1_genomic.fna	Lactobacillus	Firmicutes

1460	GCA_001956815.1_ASM195681v1_genomic.fna	Staphylococcus	Firmicutes
1461	GCA_002096015.1_ASM209601v1_genomic.fna	Mycoplasma	Tenericutes
1462	GCA_002213805.1_ASM221380v1_genomic.fna	Listeria	Firmicutes
1463	GCA_002109405.1_ASM210940v1_genomic.fna	Lactobacillus	Firmicutes
1464	GCA_002209025.1_ASM220902v1_genomic.fna	Campylobacter	Proteobacteria
1465	GCA_002157265.1_ASM215726v1_genomic.fna	Bacillus	Firmicutes
1466	GCA_001956755.1_ASM195675v1_genomic.fna	Staphylococcus	Firmicutes
1467	GCA_002192235.1_ASM219223v1_genomic.fna	Bacillus	Firmicutes
1468	GCA_002209045.1_ASM220904v1_genomic.fna	Campylobacter	Proteobacteria
1469	GCA_001975005.1_ASM197500v1_genomic.fna	Staphylococcus	Firmicutes
1470	GCA_000019265.1_ASM1926v1_genomic.fna	Streptococcus	Firmicutes
1471	GCA_002213845.1_ASM221384v1_genomic.fna	Listeria	Firmicutes
1472	GCA_001598095.1_ASM159809v1_genomic.fna	Bacillus	Firmicutes
1473	GCA_002096035.1_ASM209603v1_genomic.fna	Mycoplasma	Tenericutes
1474	GCA_001465815.1_ASM146581v1_genomic.fna	Bacillus	Firmicutes
1475	GCA_001267475.1_ASM126747v1_genomic.fna	Francisella	Proteobacteria
1476	GCA_000438645.1_ASM43864v1_genomic.fna	Listeria	Firmicutes
1477	GCA_000012165.1_ASM1216v1_genomic.fna	Streptococcus	Firmicutes
1478	GCA_002209065.1_ASM220906v1_genomic.fna	Campylobacter	Proteobacteria
1479	GCA_002073695.1_ASM207369v1_genomic.fna	Streptococcus	Firmicutes
1480	GCA_001896045.1_ASM189604v1_genomic.fna	Streptococcus	Firmicutes
1481	GCA_002214785.1_ASM221478v1_genomic.fna	Campylobacter	Proteobacteria
1482	GCA_000225465.1_ASM22546v1_genomic.fna	Buchnera	Proteobacteria
1483	GCA_001654475.1_ASM165447v1_genomic.fna	Bacillus	Firmicutes
1484	GCA_002213865.1_ASM221386v1_genomic.fna	Listeria	Firmicutes
1485	GCA_001019675.1_ASM101967v1_genomic.fna	Streptococcus	Firmicutes
1486	GCA_001975045.1_ASM197504v1_genomic.fna	Staphylococcus	Firmicutes
1487	GCA_002224325.1_ASM222432v1_genomic.fna	Campylobacter	Proteobacteria
1488	GCA_000296595.1_ASM29659v1_genomic.fna	Staphylococcus	Firmicutes
1489	GCA_002224385.1_ASM222438v1_genomic.fna	Campylobacter	Proteobacteria
1490	GCA_000148705.1_ASM14870v1_genomic.fna	Campylobacter	Proteobacteria
1491	GCA_002213905.1_ASM221390v1_genomic.fna	Listeria	Firmicutes
1492	GCA_001721145.1_ASM172114v1_genomic.fna	Bacillus	Firmicutes
1493	GCA_002000565.1_ASM200056v1_genomic.fna	Staphylococcus	Firmicutes

1494	GCA_002078995.1_ASM207899v1_genomic.fna	Lactococcus	Firmicutes
1495	GCA_000168595.2_ASM16859v2_genomic.fna	Listeria	Firmicutes
1496	GCA_001635995.1_ASM163599v1_genomic.fna	Bacillus	Firmicutes
1497	GCA_002312985.1_ASM231298v1_genomic.fna	Clostridium	Firmicutes
1498	GCA_002213885.1_ASM221388v1_genomic.fna	Listeria	Firmicutes
1499	GCA_000021785.1_ASM2178v1_genomic.fna	Bacillus	Firmicutes
1500	GCA_000312685.1_ASM31268v1_genomic.fna	Lactococcus	Firmicutes
1501	GCA_000284535.1_ASM28453v1_genomic.fna	Staphylococcus	Firmicutes
1502	GCA_900086615.1_BPH0662_genomic.fna	Staphylococcus	Firmicutes
1503	GCA_000835345.1_ASM83534v1_genomic.fna	Campylobacter	Proteobacteria
1504	GCA_001986135.1_ASM198613v1_genomic.fna	Staphylococcus	Firmicutes
1505	GCA_000011265.1_ASM1126v1_genomic.fna	Staphylococcus	Firmicutes
1506	GCA_000835365.1_ASM83536v1_genomic.fna	Campylobacter	Proteobacteria
1507	GCA_001019695.1_ASM101969v1_genomic.fna	Streptococcus	Firmicutes
1508	GCA_002355795.1_ASM235579v1_genomic.fna	Clostridium	Firmicutes
1509	GCA_001683065.1_ASM168306v1_genomic.fna	Bacillus	Firmicutes
1510	GCA_002213925.1_ASM221392v1_genomic.fna	Listeria	Firmicutes
1511	GCA_000168635.2_ASM16863v2_genomic.fna	Listeria	Firmicutes
1512	GCA_001896065.1_ASM189606v1_genomic.fna	Streptococcus	Firmicutes
1513	GCA_002197205.1_ASM219720v1_genomic.fna	Streptococcus	Firmicutes
1514	GCA_002000585.1_ASM200058v1_genomic.fna	Staphylococcus	Firmicutes
1515	GCA_000013845.2_ASM1384v2_genomic.fna	Clostridium	Firmicutes
1516	GCA_002148215.1_ASM214821v1_genomic.fna	Lactococcus	Firmicutes
1517	GCA_000171795.2_ASM17179v2_genomic.fna	Campylobacter	Proteobacteria
1518	GCA_000438665.1_ASM43866v1_genomic.fna	Listeria	Firmicutes
1519	GCA_000011505.1_ASM1150v1_genomic.fna	Staphylococcus	Firmicutes
1520	GCA_000344575.1_ASM34457v1_genomic.fna	Lactococcus	Firmicutes
1521	GCA_000022505.1_ASM2250v1_genomic.fna	Bacillus	Firmicutes
1522	GCA_001729565.1_ASM172956v1_genomic.fna	Francisella	Proteobacteria
1523	GCA_001534785.1_ASM153478v1_genomic.fna	Bacillus	Firmicutes
1524	GCA_000013485.1_ASM1348v1_genomic.fna	Streptococcus	Firmicutes
1525	GCA_000019825.1_ASM1982v1_genomic.fna	Streptococcus	Firmicutes
1526	GCA_002127985.1_ASM212798v1_genomic.fna	Mycoplasma	Tenericutes
1527	GCA_000009605.1_ASM960v1_genomic.fna	Buchnera	Proteobacteria

1528	GCA_000011485.1_ASM1148v1_genomic.fna	Prochlorococcus	Cyanobacteria
1529	GCA_002238355.1_ASM223835v1_genomic.fna	Campylobacter	Proteobacteria
1530	GCA_002234455.1_ASM223445v1_genomic.fna	Campylobacter	Proteobacteria
1531	GCA_002000605.1_ASM200060v1_genomic.fna	Staphylococcus	Firmicutes
1532	GCA_001618665.1_ASM161866v1_genomic.fna	Bacillus	Firmicutes
1533	GCA_001280225.1_ASM128022v1_genomic.fna	Buchnera	Proteobacteria
1534	GCA_002073515.1_ASM207351v1_genomic.fna	Streptococcus	Firmicutes
1535	GCA_002117245.1_ASM211724v1_genomic.fna	Lactobacillus	Firmicutes
1536	GCA_002128005.1_ASM212800v1_genomic.fna	Mycoplasma	Tenericutes
1537	GCA_000231885.1_ASM23188v1_genomic.fna	Streptococcus	Firmicutes
1538	GCA_001541905.1_ASM154190v1_genomic.fna	Bacillus	Firmicutes
1539	GCA_000008445.1_ASM844v1_genomic.fna	Bacillus	Firmicutes
1540	GCA_000196055.1_ASM19605v1_genomic.fna	Streptococcus	Firmicutes
1541	GCA_000162235.2_ASM16223v2_genomic.fna	Fusobacterium	Fusobacteria
1542	GCA_000014365.1_ASM1436v1_genomic.fna	Streptococcus	Firmicutes
1543	GCA_000007425.1_ASM742v1_genomic.fna	Streptococcus	Firmicutes
1544	GCA_001433955.1_ASM143395v1_genomic.fna	Fusobacterium	Fusobacteria
1545	GCA_002116955.1_ASM211695v1_genomic.fna	Lactobacillus	Firmicutes
1546	GCA_000730215.2_ASM73021v2_genomic.fna	Streptococcus	Firmicutes
1547	GCA_002205715.1_ASM220571v1_genomic.fna	Bacillus	Firmicutes
1548	GCA_000725325.1_ASM72532v1_genomic.fna	Bacillus	Firmicutes
1549	GCA_002216755.1_ASM221675v1_genomic.fna	Bacillus	Firmicutes
1550	GCA_001640965.1_ASM164096v1_genomic.fna	Bacillus	Firmicutes
1551	GCA_001008015.1_ASM100801v1_genomic.fna	Streptococcus	Firmicutes
1552	GCA_001578165.1_ASM157816v1_genomic.fna	Bacillus	Firmicutes
1553	GCA_001688685.2_ASM168868v2_genomic.fna	Lactobacillus	Firmicutes
1554	GCA_002197245.1_ASM219724v1_genomic.fna	Streptococcus	Firmicutes
1555	GCA_001896085.1_ASM189608v1_genomic.fna	Streptococcus	Firmicutes
1556	GCA_001683095.1_ASM168309v1_genomic.fna	Bacillus	Firmicutes
1557	GCA_000011845.1_ASM1184v1_genomic.fna	Streptococcus	Firmicutes
1558	GCA_001596055.1_ASM159605v1_genomic.fna	Bacillus	Firmicutes
1559	GCA_000026725.1_ASM2672v1_genomic.fna	Streptococcus	Firmicutes
1560	GCA_000008405.1_ASM840v1_genomic.fna	Mycoplasma	Tenericutes
1561	GCA_002173615.1_ASM217361v1_genomic.fna	Bacillus	Firmicutes

1562	GCA_001729585.1_ASM172958v1_genomic.fna	Francisella	Proteobacteria
1563	GCA_002094975.1_ASM209497v1_genomic.fna	Streptococcus	Firmicutes
1564	GCA_000018925.1_ASM1892v1_genomic.fna	Francisella	Proteobacteria
1565	GCA_001888965.1_ASM188896v1_genomic.fna	Lactobacillus	Firmicutes
1566	GCA_000292445.1_ASM29244v1_genomic.fna	Mycoplasma	Tenericutes
1567	GCA_000590455.1_ASM59045v1_genomic.fna	Bacillus	Firmicutes
1568	GCA_000159455.2_ASM15945v2_genomic.fna	Lactobacillus	Firmicutes
1569	GCA_000300475.1_ASM30047v1_genomic.fna	Bacillus	Firmicutes
1570	GCA_001020185.2_ASM102018v2_genomic.fna	Streptococcus	Firmicutes
1571	GCA_002094935.1_ASM209493v1_genomic.fna	Coxiella	Proteobacteria
1572	GCA_000338735.1_ASM33873v1_genomic.fna	Bacillus	Firmicutes
1573	GCA_002192215.1_ASM219221v1_genomic.fna	Lactobacillus	Firmicutes
1574	GCA_001702095.1_ASM170209v1_genomic.fna	Lactobacillus	Firmicutes
1575	GCA_000831545.1_ASM83154v1_genomic.fna	Rickettsia	Proteobacteria
1576	GCA_002356135.1_ASM235613v1_genomic.fna	Lactobacillus	Firmicutes
1577	GCA_000182835.1_ASM18283v1_genomic.fna	Lactobacillus	Firmicutes
1578	GCA_000009325.1_ASM932v1_genomic.fna	Francisella	Proteobacteria
1579	GCA_000253335.1_ASM25333v1_genomic.fna	Streptococcus	Firmicutes
1580	GCA_002080065.1_ASM208006v1_genomic.fna	Clostridioides	Firmicutes
1581	GCA_000015065.1_ASM1506v1_genomic.fna	Bacillus	Firmicutes
1582	GCA_001484645.1_ASM148464v1_genomic.fna	Campylobacter	Proteobacteria
1583	GCA_000020285.1_ASM2028v1_genomic.fna	Clostridium	Firmicutes
1584	GCA_002442595.1_ASM244259v1_genomic.fna	Borreliella	Spirochaetes
1585	GCA_001598635.1_ASM159863v1_genomic.fna	Enterococcus	Firmicutes
1586	GCA_000194765.1_ASM19476v1_genomic.fna	Lactobacillus	Firmicutes
1587	GCA_000018745.1_ASM1874v1_genomic.fna	Coxiella	Proteobacteria
1588	GCA_000210435.1_ASM21043v1_genomic.fna	Clostridioides	Firmicutes
1589	GCA_001560895.1_ASM156089v1_genomic.fna	Streptococcus	Firmicutes
1590	GCA_000015085.1_ASM1508v1_genomic.fna	Campylobacter	Proteobacteria
1591	GCA_000367405.1_ASM36740v1_genomic.fna	Rickettsia	Proteobacteria
1592	GCA_000835085.1_ASM83508v1_genomic.fna	Mycoplasma	Tenericutes
1593	GCA_001554075.1_ASM155407v1_genomic.fna	Mycoplasma	Tenericutes
1594	GCA_002078315.1_ASM207831v1_genomic.fna	Rickettsia	Proteobacteria
1595	GCA_002355575.1_ASM235557v1_genomic.fna	Lactococcus	Firmicutes

1596	GCA_000741005.1_ASM74100v1_genomic.fna	Borrelia	Spirochaetes
1597	GCA_000283775.1_ASM28377v1_genomic.fna	Rickettsia	Proteobacteria
1598	GCA_000165775.3_ASM16577v3_genomic.fna	Lactobacillus	Firmicutes
1599	GCA_000186745.1_ASM18674v1_genomic.fna	Bacillus	Firmicutes
1600	GCA_001902295.1_ASM190229v1_genomic.fna	Clostridium	Firmicutes
1601	GCA_000397165.1_ASM39716v1_genomic.fna	Lactobacillus	Firmicutes
1602	GCA_000063585.1_ASM6358v1_genomic.fna	Clostridium	Firmicutes
1603	GCA_000023685.1_ASM2368v1_genomic.fna	Mycoplasma	Tenericutes
1604	GCA_000166635.2_ASM16663v1_genomic.fna	Borrelia	Spirochaetes
1605	GCA_000277165.1_ASM27716v1_genomic.fna	Rickettsia	Proteobacteria
1606	GCA_000172575.2_ASM17257v2_genomic.fna	Enterococcus	Firmicutes
1607	GCA_000382965.1_ASM38296v1_genomic.fna	Staphylococcus	Firmicutes
1608	GCA_000934305.1_ASM93430v1_genomic.fna	Campylobacter	Proteobacteria
1609	GCA_000438685.2_ASM43868v2_genomic.fna	Listeria	Firmicutes
1610	GCA_001880305.1_ASM188030v1_genomic.fna	Bacillus	Firmicutes
1611	GCA_900088425.1_A12_genomic.fna	Lactococcus	Firmicutes
1612	GCA_001299565.1_ASM129956v1_genomic.fna	Campylobacter	Proteobacteria
1613	GCA_000382985.1_ASM38298v1_genomic.fna	Staphylococcus	Firmicutes
1614	GCA_001941885.1_ASM194188v1_genomic.fna	Bacillus	Firmicutes
1615	GCA_000438705.2_ASM43870v2_genomic.fna	Listeria	Firmicutes
1616	GCA_001729605.1_ASM172960v1_genomic.fna	Francisella	Proteobacteria
1617	GCA_001685565.1_ASM168556v1_genomic.fna	Bacillus	Firmicutes
1618	GCA_001021955.1_ASM102195v1_genomic.fna	Streptococcus	Firmicutes
1619	GCA_001683135.1_ASM168313v1_genomic.fna	Bacillus	Firmicutes
1620	GCA_001902455.1_ASM190245v1_genomic.fna	Streptococcus	Firmicutes
1621	GCA_002197265.1_ASM219726v1_genomic.fna	Streptococcus	Firmicutes
1622	GCA_002117285.1_ASM211728v1_genomic.fna	Lactobacillus	Firmicutes
1623	GCA_002238395.1_ASM223839v1_genomic.fna	Bacillus	Firmicutes
1624	GCA_000833005.1_ASM83300v1_genomic.fna	Bacillus	Firmicutes
1625	GCA_001565875.1_ASM156587v1_genomic.fna	Bacillus	Firmicutes
1626	GCA_001587115.1_ASM158711v1_genomic.fna	Enterococcus	Firmicutes
1627	GCA_002128025.1_ASM212802v1_genomic.fna	Mycoplasma	Tenericutes
1628	GCA_002117265.1_ASM211726v1_genomic.fna	Lactobacillus	Firmicutes
1629	GCA_002076955.1_ASM207695v1_genomic.fna	Lactobacillus	Firmicutes

1630	GCA_002237515.1_ASM223751v1_genomic.fna	Bacillus	Firmicutes
1631	GCA_001672055.1_ASM167205v1_genomic.fna	Serratia	Proteobacteria
1632	GCA_000286755.1_ASM28675v1_genomic.fna	Mycoplasma	Tenericutes
1633	GCA_002173555.1_ASM217355v1_genomic.fna	Lactobacillus	Firmicutes
1634	GCA_002128045.1_ASM212804v1_genomic.fna	Mycoplasma	Tenericutes
1635	GCA_001272715.1_ASM127271v1_genomic.fna	Mycoplasma	Tenericutes
1636	GCA_001597265.1_ASM159726v1_genomic.fna	Bacillus	Firmicutes
1637	GCA_002117185.1_ASM211718v1_genomic.fna	Leuconostoc	Firmicutes
1638	GCA_001865695.1_ASM186569v1_genomic.fna	Francisella	Proteobacteria
1639	GCA_001272315.2_ASM127231v2_genomic.fna	Lactobacillus	Firmicutes
1640	GCA_001692675.1_ASM169267v1_genomic.fna	Bacillus	Firmicutes
1641	GCA_000438725.2_ASM43872v2_genomic.fna	Listeria	Firmicutes
1642	GCA_001941905.1_ASM194190v1_genomic.fna	Bacillus	Firmicutes
1643	GCA_000188715.1_ASM18871v1_genomic.fna	Streptococcus	Firmicutes
1644	GCA_002009275.1_ASM200927v1_genomic.fna	Mycoplasma	Tenericutes
1645	GCA_002197285.1_ASM219728v1_genomic.fna	Streptococcus	Firmicutes
1646	GCA_001639125.1_ASM163912v1_genomic.fna	Campylobacter	Proteobacteria
1647	GCA_001683155.1_ASM168315v1_genomic.fna	Bacillus	Firmicutes
1648	GCA_002357995.1_ASM235799v1_genomic.fna	Streptococcus	Firmicutes
1649	GCA_001023495.1_ASM102349v1_genomic.fna	Streptococcus	Firmicutes
1650	GCA_001941925.1_ASM194192v1_genomic.fna	Bacillus	Firmicutes
1651	GCA_000438745.2_ASM43874v2_genomic.fna	Listeria	Firmicutes
1652	GCA_002357235.1_ASM235723v1_genomic.fna	Rickettsia	Proteobacteria
1653	GCA_000742855.1_ASM74285v1_genomic.fna	Bacillus	Firmicutes
1654	GCA_000336405.1_ASM33640v1_genomic.fna	Enterococcus	Firmicutes
1655	GCA_000271865.1_ASM27186v1_genomic.fna	Streptococcus	Firmicutes
1656	GCA_000204275.1_ASM20427v1_genomic.fna	Bacillus	Firmicutes
1657	GCA_000300045.1_ASM30004v1_genomic.fna	Borrelia	Spirochaetes
1658	GCA_000383005.1_ASM38300v1_genomic.fna	Staphylococcus	Firmicutes
1659	GCA_002257625.1_ASM225762v1_genomic.fna	Lactobacillus	Firmicutes
1660	GCA_001299595.1_ASM129959v1_genomic.fna	Campylobacter	Proteobacteria
1661	GCA_001314285.1_ASM131428v1_genomic.fna	Campylobacter	Proteobacteria
1662	GCA_000412775.1_ASM41277v1_genomic.fna	Staphylococcus	Firmicutes
1663	GCA_002357255.1_ASM235725v1_genomic.fna	Rickettsia	Proteobacteria

1664	GCA_001721165.1_ASM172116v1_genomic.fna	Bacillus	Firmicutes
1665	GCA_000026525.1_ASM2652v1_genomic.fna	Lactobacillus	Firmicutes
1666	GCA_000833215.1_ASM83321v1_genomic.fna	Francisella	Proteobacteria
1667	GCA_001880245.1_ASM188024v1_genomic.fna	Francisella	Proteobacteria
1668	GCA_000026585.1_ASM2658v1_genomic.fna	Streptococcus	Firmicutes
1669	GCA_000583065.1_ASM58306v1_genomic.fna	Bacillus	Firmicutes
1670	GCA_001039695.2_emm89-1_genomic.fna	Streptococcus	Firmicutes
1671	GCA_001683175.1_ASM168317v1_genomic.fna	Bacillus	Firmicutes
1672	GCA_000685725.1_ASM68572v1_genomic.fna	Bacillus	Firmicutes
1673	GCA_002197305.1_ASM219730v1_genomic.fna	Streptococcus	Firmicutes
1674	GCA_001679705.1_ASM167970v1_genomic.fna	Clostridium	Firmicutes
1675	GCA_002117305.1_ASM211730v1_genomic.fna	Lactobacillus	Firmicutes
1676	GCA_000403625.1_ASM40362v1_genomic.fna	Streptococcus	Firmicutes
1677	GCA_002243325.2_ASM224332v2_genomic.fna	Bacillus	Firmicutes
1678	GCA_002310315.1_ASM231031v1_genomic.fna	Bacillus	Firmicutes
1679	GCA_001281105.1_ASM128110v1_genomic.fna	Streptococcus	Firmicutes
1680	GCA_000025805.1_ASM2580v1_genomic.fna	Bacillus	Firmicutes
1681	GCA_001604995.1_ASM160499v1_genomic.fna	Bacillus	Firmicutes
1682	GCA_002128065.1_ASM212806v1_genomic.fna	Mycoplasma	Tenericutes
1683	GCA_002442835.1_ASM244283v1_genomic.fna	Lactobacillus	Firmicutes
1684	GCA_001717605.1_ASM171760v1_genomic.fna	Campylobacter	Proteobacteria
1685	GCA_000286775.1_ASM28677v1_genomic.fna	Mycoplasma	Tenericutes
1686	GCA_002220515.1_ASM222051v1_genomic.fna	Serratia	Proteobacteria
1687	GCA_002174235.1_ASM217423v1_genomic.fna	Lactobacillus	Firmicutes
1688	GCA_000512955.1_ASM51295v1_genomic.fna	Leuconostoc	Firmicutes
1689	GCA_000283755.1_ASM28375v1_genomic.fna	Mycoplasma	Tenericutes
1690	GCA_002287905.1_ASM228790v1_genomic.fna	Lactobacillus	Firmicutes
1691	GCA_000218855.1_ASM21885v1_genomic.fna	Clostridium	Firmicutes
1692	GCA_002173655.1_ASM217365v1_genomic.fna	Lactobacillus	Firmicutes
1693	GCA_002128085.1_ASM212808v1_genomic.fna	Mycoplasma	Tenericutes
1694	GCA_001272735.1_ASM127273v1_genomic.fna	Mycoplasma	Tenericutes
1695	GCA_002158925.1_ASM215892v1_genomic.fna	Lactobacillus	Firmicutes
1696	GCA_000336425.1_ASM33642v1_genomic.fna	Serratia	Proteobacteria
1697	GCA_001676805.1_ASM167680v1_genomic.fna	Lactobacillus	Firmicutes

1698	GCA_001411765.2_ASM141176v2_genomic.fna	Pediococcus	Firmicutes
1699	GCA_000148815.2_ASM14881v1_genomic.fna	Lactobacillus	Firmicutes
1700	GCA_000934625.1_ASM93462v1_genomic.fna	Lactobacillus	Firmicutes
1701	GCA_002148235.1_ASM214823v1_genomic.fna	Leuconostoc	Firmicutes
1702	GCA_001278015.1_ASM127801v1_genomic.fna	Lactobacillus	Firmicutes
1703	GCA_001635875.1_ASM163587v1_genomic.fna	Enterococcus	Firmicutes
1704	GCA_000835145.1_ASM83514v1_genomic.fna	Bacillus	Firmicutes
1705	GCA_001689055.2_ASM168905v2_genomic.fna	Enterococcus	Firmicutes
1706	GCA_000506785.3_ASM50678v3_genomic.fna	Clostridium	Firmicutes
1707	GCA_000270525.1_ASM27052v1_genomic.fna	Mycoplasma	Tenericutes
1708	GCA_000816365.1_ASM81636v1_genomic.fna	Campylobacter	Proteobacteria
1709	GCA_000494775.1_ASM49477v1_genomic.fna	Campylobacter	Proteobacteria
1710	GCA_002504365.1_ASM250436v1_genomic.fna	Mesoplasma	Tenericutes
1711	GCA_000025385.1_ASM2538v1_genomic.fna	Mycoplasma	Tenericutes
1712	GCA_000217835.1_ASM21783v1_genomic.fna	Bacillus	Firmicutes
1713	GCA_001558275.1_ASM155827v1_genomic.fna	Staphylococcus	Firmicutes
1714	GCA_000763475.1_ASM76347v1_genomic.fna	Listeria	Firmicutes
1715	GCA_002250115.1_ASM225011v1_genomic.fna	Bacillus	Firmicutes
1716	GCA_000758365.1_ASM75836v1_genomic.fna	Lactobacillus	Firmicutes
1717	GCA_000020345.1_ASM2034v1_genomic.fna	Clostridium	Firmicutes
1718	GCA_002220115.1_ASM222011v1_genomic.fna	Streptococcus	Firmicutes
1719	GCA_001598075.1_ASM159807v1_genomic.fna	Lysinibacillus	Firmicutes
1720	GCA_000284355.1_ASM28435v1_genomic.fna	Arcobacter	Proteobacteria
1721	GCA_002250035.1_ASM225003v1_genomic.fna	Lactobacillus	Firmicutes
1722	GCA_000277305.1_ASM27730v1_genomic.fna	Rickettsia	Proteobacteria
1723	GCA_000204985.1_ASM20498v1_genomic.fna	Lactobacillus	Firmicutes
1724	GCA_001646605.1_ASM164660v1_genomic.fna	Clostridium	Firmicutes
1725	GCA_002197325.1_ASM219732v1_genomic.fna	Streptococcus	Firmicutes
1726	GCA_001660525.1_ASM166052v1_genomic.fna	Bacillus	Firmicutes
1727	GCA_002025105.1_ASM202510v1_genomic.fna	Bacillus	Firmicutes
1728	GCA_000513595.1_ASM51359v1_genomic.fna	Listeria	Firmicutes
1729	GCA_002000005.1_ASM200000v1_genomic.fna	Bacillus	Firmicutes
1730	GCA_000418345.1_ASM41834v1_genomic.fna	Staphylococcus	Firmicutes
1731	GCA_001683195.1_ASM168319v1_genomic.fna	Bacillus	Firmicutes

1732	GCA_001051095.1_ASM105109v1_genomic.fna	Streptococcus	Firmicutes
1733	GCA_001412295.1_ASM141229v1_genomic.fna	Campylobacter	Proteobacteria
1734	GCA_001457695.1_NCTC11351_genomic.fna	Campylobacter	Proteobacteria
1735	GCA_000452385.2_ASM45238v2_genomic.fna	Staphylococcus	Firmicutes
1736	GCA_002214705.1_ASM221470v1_genomic.fna	Bacillus	Firmicutes
1737	GCA_000513615.1_ASM51361v1_genomic.fna	Listeria	Firmicutes
1738	GCA_001697265.1_ASM169726v1_genomic.fna	Bacillus	Firmicutes
1739	GCA_002109345.1_ASM210934v1_genomic.fna	Coxiella	Proteobacteria
1740	GCA_002119445.1_ASM211944v1_genomic.fna	Bacillus	Firmicutes
1741	GCA_002234355.1_ASM223435v1_genomic.fna	Clostridioides	Firmicutes
1742	GCA_001267805.1_ASM126780v1_genomic.fna	Streptococcus	Firmicutes
1743	GCA_001602215.1_ASM160221v1_genomic.fna	Rickettsia	Proteobacteria
1744	GCA_001683215.1_ASM168321v1_genomic.fna	Bacillus	Firmicutes
1745	GCA_001554155.1_ASM155415v1_genomic.fna	Mycoplasma	Tenericutes
1746	GCA_002197365.1_ASM219736v1_genomic.fna	Streptococcus	Firmicutes
1747	GCA_001950995.1_ASM195099v1_genomic.fna	Rickettsia	Proteobacteria
1748	GCA_001746265.2_ASM174626v2_genomic.fna	Lactobacillus	Firmicutes
1749	GCA_000344745.1_ASM34474v1_genomic.fna	Bacillus	Firmicutes
1750	GCA_002205775.2_ASM220577v2_genomic.fna	Lactobacillus	Firmicutes
1751	GCA_002128125.1_ASM212812v1_genomic.fna	Mycoplasma	Tenericutes
1752	GCA_000306745.1_ASM30674v1_genomic.fna	Bacillus	Firmicutes
1753	GCA_001686885.1_ASM168688v1_genomic.fna	Campylobacter	Proteobacteria
1754	GCA_001888985.1_ASM188898v1_genomic.fna	Lactobacillus	Firmicutes
1755	GCA_002128105.1_ASM212810v1_genomic.fna	Mycoplasma	Tenericutes
1756	GCA_002174195.1_ASM217419v1_genomic.fna	Lactobacillus	Firmicutes
1757	GCA_000023305.1_ASM2330v1_genomic.fna	Francisella	Proteobacteria
1758	GCA_002327165.1_ASM232716v1_genomic.fna	Bacillus	Firmicutes
1759	GCA_000231905.1_ASM23190v1_genomic.fna	Streptococcus	Firmicutes
1760	GCA_002197385.1_ASM219738v1_genomic.fna	Streptococcus	Firmicutes
1761	GCA_001700735.1_ASM170073v1_genomic.fna	Bacillus	Firmicutes
1762	GCA_001703495.1_ASM170349v1_genomic.fna	Bacillus	Firmicutes
1763	GCA_001280285.1_ASM128028v1_genomic.fna	Streptococcus	Firmicutes
1764	GCA_002173675.1_ASM217367v1_genomic.fna	Bacillus	Firmicutes
1765	GCA_002173755.1_ASM217375v1_genomic.fna	Bacillus	Firmicutes

1766	GCA_000513635.1_ASM51363v1_genomic.fna	Listeria	Firmicutes
1767	GCA_002214725.1_ASM221472v1_genomic.fna	Bacillus	Firmicutes
1768	GCA_000462955.1_ASM46295v1_genomic.fna	Staphylococcus	Firmicutes
1769	GCA_001506185.1_ASM150618v1_genomic.fna	Campylobacter	Proteobacteria
1770	GCA_002243705.1_ASM224370v1_genomic.fna	Anoxybacillus	Firmicutes
1771	GCA_000742655.1_ASM74265v1_genomic.fna	Bacillus	Firmicutes
1772	GCA_001683235.1_ASM168323v1_genomic.fna	Bacillus	Firmicutes
1773	GCA_001267845.1_ASM126784v1_genomic.fna	Streptococcus	Firmicutes
1774	GCA_000730255.1_ASM73025v1_genomic.fna	Streptococcus	Firmicutes
1775	GCA_001457555.1_NCTC10562_genomic.fna	Fusobacterium	Fusobacteria
1776	GCA_000019985.1_ASM1998v1_genomic.fna	Streptococcus	Firmicutes
1777	GCA_000013505.1_ASM1350v1_genomic.fna	Streptococcus	Firmicutes
1778	GCA_000015705.1_ASM1570v1_genomic.fna	Prochlorococcus	Cyanobacteria
1779	GCA_000168695.2_ASM16869v2_genomic.fna	Listeria	Firmicutes
1780	GCA_000468955.1_ASM46895v1_genomic.fna	Lactococcus	Firmicutes
1781	GCA_002591175.1_ASM259117v1_genomic.fna	Staphylococcus	Firmicutes
1782	GCA_000155515.2_ASM15551v2_genomic.fna	Lactobacillus	Firmicutes
1783	GCA_000211295.1_ASM21129v1_genomic.fna	Mycoplasma	Tenericutes
1784	GCA_000463385.1_ASM46338v1_genomic.fna	Streptococcus	Firmicutes
1785	GCA_002250075.1_ASM225007v1_genomic.fna	Thermoanaerobacte	Firmicutes
1786	GCA_900016785.1_BTsPazieg1.0_genomic.fna	Buchnera	Proteobacteria
1787	GCA_001506205.1_ASM150620v1_genomic.fna	Campylobacter	Proteobacteria
1788	GCA_000463055.1_ASM46305v1_genomic.fna	Staphylococcus	Firmicutes
1789	GCA_002214765.1_ASM221476v1_genomic.fna	Bacillus	Firmicutes
1790	GCA_000015685.1_ASM1568v1_genomic.fna	Prochlorococcus	Cyanobacteria
1791	GCA_000513655.1_ASM51365v1_genomic.fna	Listeria	Firmicutes
1792	GCA_000302555.4_ASM30255v4_genomic.fna	Campylobacter	Proteobacteria
1793	GCA_002184245.1_ASM218424v1_genomic.fna	Bacillus	Firmicutes
1794	GCA_000012045.1_ASM1204v1_genomic.fna	Staphylococcus	Firmicutes
1795	GCA_001704095.1_ASM170409v1_genomic.fna	Bacillus	Firmicutes
1796	GCA_002173575.1_ASM217357v1_genomic.fna	Pediococcus	Firmicutes
1797	GCA_002386345.1_ASM238634v1_genomic.fna	Streptococcus	Firmicutes
1798	GCA_000830075.1_ASM83007v1_genomic.fna	Bacillus	Firmicutes
1799	GCA_001602155.1_ASM160215v1_genomic.fna	Erysipelothrix	Firmicutes

1800	GCA_000143605.1_ASM14360v1_genomic.fna	Bacillus	Firmicutes
1801	GCA_001535565.1_ASM153556v1_genomic.fna	Streptococcus	Firmicutes
1802	GCA_000011525.1_ASM1152v1_genomic.fna	Staphylococcus	Firmicutes
1803	GCA_001683255.1_ASM168325v1_genomic.fna	Bacillus	Firmicutes
1804	GCA_002197425.1_ASM219742v1_genomic.fna	Streptococcus	Firmicutes
1805	GCA_000184205.1_ASM18420v1_genomic.fna	Campylobacter	Proteobacteria
1806	GCA_000015965.1_ASM1596v1_genomic.fna	Prochlorococcus	Cyanobacteria
1807	GCA_001648115.1_ASM164811v1_genomic.fna	Buchnera	Proteobacteria
1808	GCA_000478925.1_ASM47892v1_genomic.fna	Streptococcus	Firmicutes
1809	GCA_001558775.1_ASM155877v1_genomic.fna	Staphylococcus	Firmicutes
1810	GCA_000463445.1_ASM46344v1_genomic.fna	Streptococcus	Firmicutes
1811	GCA_002706375.1_ASM270637v1_genomic.fna	Lactobacillus	Firmicutes
1812	GCA_900186885.1_48903_D01_genomic.fna	Veillonella	Firmicutes
1813	GCA_000239195.1_ASM23919v1_genomic.fna	Bacillus	Firmicutes
1814	GCA_000478255.2_ASM47825v2_genomic.fna	Lactococcus	Firmicutes
1815	GCA_002128145.1_ASM212814v1_genomic.fna	Mycoplasma	Tenericutes
1816	GCA_002220175.1_ASM222017v1_genomic.fna	Lactobacillus	Firmicutes
1817	GCA_000026665.1_ASM2666v1_genomic.fna	Streptococcus	Firmicutes
1818	GCA_002211605.1_ASM221160v1_genomic.fna	Fusobacterium	Fusobacteria
1819	GCA_000782855.1_ASM78285v1_genomic.fna	Streptococcus	Firmicutes
1820	GCA_000742895.1_ASM74289v1_genomic.fna	Bacillus	Firmicutes
1821	GCA_000947915.1_ASM94791v1_genomic.fna	Mycoplasma	Tenericutes
1822	GCA_000296575.1_ASM29657v1_genomic.fna	Brachyspira	Spirochaetes
1823	GCA_000203195.1_ASM20319v1_genomic.fna	Streptococcus	Firmicutes
1824	GCA_000013525.1_ASM1352v1_genomic.fna	Streptococcus	Firmicutes
1825	GCA_002174255.1_ASM217425v1_genomic.fna	Bacillus	Firmicutes
1826	GCA_001514435.1_ASM151443v1_genomic.fna	Streptococcus	Firmicutes
1827	GCA_001704975.1_ASM170497v1_genomic.fna	Bacillus	Firmicutes
1828	GCA_001682335.1_ASM168233v1_genomic.fna	Staphylococcus	Firmicutes
1829	GCA_001536305.1_Leuconostoc_gelidum_subsp._gasicomitatum_KG16-1_genomic.fna	Leuconostoc	Firmicutes
1830	GCA_002706705.1_ASM270670v1_genomic.fna	Lactobacillus	Firmicutes
1831	GCA_001951075.1_ASM195107v1_genomic.fna	Weissella	Firmicutes
1832	GCA_000231925.1_ASM23192v1_genomic.fna	Streptococcus	Firmicutes
1833	GCA_002278015.1_ASM227801v1_genomic.fna	Enterococcus	Firmicutes

1834	GCA_002243535.1_ASM224353v1_genomic.fna	Tetragenococcus	Firmicutes
1835	GCA_002078335.1_ASM207833v1_genomic.fna	Rickettsia	Proteobacteria
1836	GCA_000338755.1_ASM33875v1_genomic.fna	Bacillus	Firmicutes
1837	GCA_001702235.1_ASM170223v1_genomic.fna	Pediococcus	Firmicutes
1838	GCA_002357115.1_ASM235711v1_genomic.fna	Rickettsia	Proteobacteria
1839	GCA_000953575.1_Staphylococcus_xylosus_C2a_genomic.fna	Staphylococcus	Firmicutes
1840	GCA_001611075.1_ASM161107v1_genomic.fna	Pediococcus	Firmicutes
1841	GCA_001698165.1_ASM169816v1_genomic.fna	Lactobacillus	Firmicutes
1842	GCA_002277935.1_ASM227793v1_genomic.fna	Enterococcus	Firmicutes
1843	GCA_002220775.1_ASM222077v1_genomic.fna	Campylobacter	Proteobacteria
1844	GCA_001042525.2_ASM104252v2_genomic.fna	Francisella	Proteobacteria
1845	GCA_002128165.1_ASM212816v1_genomic.fna	Mycoplasma	Tenericutes
1846	GCA_001720505.1_ASM172050v1_genomic.fna	Bacillus	Firmicutes
1847	GCA_002192515.1_ASM219251v1_genomic.fna	Bacillus	Firmicutes
1848	GCA_002220815.1_ASM222081v1_genomic.fna	Lactobacillus	Firmicutes
1849	GCA_001683275.1_ASM168327v1_genomic.fna	Bacillus	Firmicutes
1850	GCA_001547715.1_ASM154771v1_genomic.fna	Streptococcus	Firmicutes
1851	GCA_002214425.1_ASM221442v1_genomic.fna	Streptococcus	Firmicutes
1852	GCA_000470865.1_ASM47086v1_genomic.fna	Staphylococcus	Firmicutes
1853	GCA_001506245.1_ASM150624v1_genomic.fna	Campylobacter	Proteobacteria
1854	GCA_002216125.1_ASM221612v1_genomic.fna	Bacillus	Firmicutes
1855	GCA_000513675.1_ASM51367v1_genomic.fna	Listeria	Firmicutes
1856	GCA_002215175.1_ASM221517v1_genomic.fna	Bacillus	Firmicutes
1857	GCA_001506225.1_ASM150622v1_genomic.fna	Campylobacter	Proteobacteria
1858	GCA_000470845.1_ASM47084v1_genomic.fna	Staphylococcus	Firmicutes
1859	GCA_001908415.1_ASM190841v1_genomic.fna	Lactobacillus	Firmicutes
1860	GCA_002289205.1_ASM228920v1_genomic.fna	Streptococcus	Firmicutes
1861	GCA_000349795.1_ASM34979v1_genomic.fna	Bacillus	Firmicutes
1862	GCA_000513695.1_ASM51369v1_genomic.fna	Listeria	Firmicutes
1863	GCA_000168775.2_ASM16877v2_genomic.fna	Francisella	Proteobacteria
1864	GCA_001547835.1_ASM154783v1_genomic.fna	Streptococcus	Firmicutes
1865	GCA_001683295.1_ASM168329v1_genomic.fna	Bacillus	Firmicutes
1866	GCA_002208905.1_ASM220890v1_genomic.fna	Staphylococcus	Firmicutes
1867	GCA_001878735.1_ASM187873v1_genomic.fna	Enterococcus	Firmicutes

1868	GCA_002286275.1_ASM228627v1_genomic.fna	Lactobacillus	Firmicutes
1869	GCA_001951015.1_ASM195101v1_genomic.fna	Rickettsia	Proteobacteria
1870	GCA_002222555.1_ASM222255v1_genomic.fna	Bacillus	Firmicutes
1871	GCA_001564435.1_ASM156443v1_genomic.fna	Mycoplasma	Tenericutes
1872	GCA_001746575.1_ASM174657v1_genomic.fna	Bacillus	Firmicutes
1873	GCA_000022765.1_ASM2276v1_genomic.fna	Clostridium	Firmicutes
1874	GCA_002128185.1_ASM212818v1_genomic.fna	Mycoplasma	Tenericutes
1875	GCA_002243685.1_ASM224368v1_genomic.fna	Bacillus	Firmicutes
1876	GCA_001747445.1_ASM174744v1_genomic.fna	Bacillus	Firmicutes
1877	GCA_000973485.1_ASM97348v1_genomic.fna	Bacillus	Firmicutes
1878	GCA_002209265.1_ASM220926v1_genomic.fna	Staphylococcus	Firmicutes
1879	GCA_001720945.1_ASM172094v1_genomic.fna	Enterococcus	Firmicutes
1880	GCA_002290185.1_ASM229018v1_genomic.fna	Lactobacillus	Firmicutes
1881	GCA_002128205.1_ASM212820v1_genomic.fna	Mycoplasma	Tenericutes
1882	GCA_002286235.1_ASM228623v1_genomic.fna	Lactobacillus	Firmicutes
1883	GCA_000286795.1_ASM28679v1_genomic.fna	Mycoplasma	Tenericutes
1884	GCA_002220535.1_ASM222053v1_genomic.fna	Serratia	Proteobacteria
1885	GCA_001683315.1_ASM168331v1_genomic.fna	Staphylococcus	Firmicutes
1886	GCA_000261045.2_ASM26104v2_genomic.fna	Serratia	Proteobacteria
1887	GCA_001272755.1_ASM127275v1_genomic.fna	Mycoplasma	Tenericutes
1888	GCA_900078265.1_SA111_genomic.fna	Streptococcus	Firmicutes
1889	GCA_001296095.1_ASM129609v1_genomic.fna	Lactobacillus	Firmicutes
1890	GCA_001936375.1_ASM193637v1_genomic.fna	Bacillus	Firmicutes
1891	GCA_002370395.1_ASM237039v1_genomic.fna	Leuconostoc	Firmicutes
1892	GCA_001559175.1_ASM155917v1_genomic.fna	Streptococcus	Firmicutes
1893	GCA_001506285.1_ASM150628v1_genomic.fna	Campylobacter	Proteobacteria
1894	GCA_002224345.1_ASM222434v1_genomic.fna	Bacillus	Firmicutes
1895	GCA_000568935.1_ASM56893v1_genomic.fna	Listeria	Firmicutes
1896	GCA_001938685.1_ASM193868v1_genomic.fna	Bacillus	Firmicutes
1897	GCA_001865455.1_ASM186545v1_genomic.fna	Campylobacter	Proteobacteria
1898	GCA_000568475.1_ASM56847v1_genomic.fna	Listeria	Firmicutes
1899	GCA_002220285.1_ASM222028v1_genomic.fna	Bacillus	Firmicutes
1900	GCA_001506265.1_ASM150626v1_genomic.fna	Campylobacter	Proteobacteria
1901	GCA_000485885.1_ASM48588v1_genomic.fna	Staphylococcus	Firmicutes

1902	GCA_000648525.1_ASM64852v1_genomic.fna	Streptococcus	Firmicutes
1903	GCA_000568455.1_ASM56845v1_genomic.fna	Staphylococcus	Firmicutes
1904	GCA_000973585.1_ASM97358v1_genomic.fna	Bacillus	Firmicutes
1905	GCA_002003325.1_ASM200332v1_genomic.fna	Clostridium	Firmicutes
1906	GCA_000750515.1_ASM75051v1_genomic.fna	Weissella	Firmicutes
1907	GCA_002442515.1_ASM244251v1_genomic.fna	Brochothrix	Firmicutes
1908	GCA_001559135.1_ASM155913v1_genomic.fna	Serratia	Proteobacteria
1909	GCA_002357275.1_ASM235727v1_genomic.fna	Rickettsia	Proteobacteria
1910	GCA_001620285.1_ASM162028v1_genomic.fna	Streptococcus	Firmicutes
1911	GCA_000769555.1_ASM76955v1_genomic.fna	Bacillus	Firmicutes
1912	GCA_001990245.1_ASM199024v1_genomic.fna	Bacillus	Firmicutes
1913	GCA_900155855.1_BM110_genomic.fna	Streptococcus	Firmicutes
1914	GCA_001865475.1_ASM186547v1_genomic.fna	Campylobacter	Proteobacteria
1915	GCA_001455145.1_ASM145514v1_genomic.fna	Fusobacterium	Fusobacteria
1916	GCA_000284395.1_ASM28439v1_genomic.fna	Bacillus	Firmicutes
1917	GCA_002327205.1_ASM232720v1_genomic.fna	Clostridium	Firmicutes
1918	GCA_002357295.1_ASM235729v1_genomic.fna	Rickettsia	Proteobacteria
1919	GCA_001808235.1_ASM180823v1_genomic.fna	Bacillus	Firmicutes
1920	GCA_002128235.1_ASM212823v1_genomic.fna	Mycoplasma	Tenericutes
1921	GCA_002576835.1_ASM257683v1_genomic.fna	Lactobacillus	Firmicutes
1922	GCA_000648555.1_ASM64855v1_genomic.fna	Streptococcus	Firmicutes
1923	GCA_001272775.1_ASM127277v1_genomic.fna	Mycoplasma	Tenericutes
1924	GCA_002068155.1_ASM206815v1_genomic.fna	Bacillus	Firmicutes
1925	GCA_001543245.1_ASM154324v1_genomic.fna	Aerococcus	Firmicutes
1926	GCA_001702195.1_ASM170219v1_genomic.fna	Lactobacillus	Firmicutes
1927	GCA_001543145.1_ASM154314v1_genomic.fna	Aerococcus	Firmicutes
1928	GCA_001188895.1_ASM118889v1_genomic.fna	Staphylococcus	Firmicutes
1929	GCA_001534665.1_ASM153466v1_genomic.fna	Arsenophonus	Proteobacteria
1930	GCA_001643995.1_ASM164399v1_genomic.fna	Campylobacter	Proteobacteria
1931	GCA_000286815.1_ASM28681v1_genomic.fna	Mycoplasma	Tenericutes
1932	GCA_002220555.1_ASM222055v1_genomic.fna	Serratia	Proteobacteria
1933	GCA_002370415.1_ASM237041v1_genomic.fna	Leuconostoc	Firmicutes
1934	GCA_001302645.1_ASM130264v1_genomic.fna	Lactobacillus	Firmicutes
1935	GCA_001721065.1_ASM172106v1_genomic.fna	Enterococcus	Firmicutes

1936	GCA_001483885.1_ASM148388v1_genomic.fna	Bacillus	Firmicutes
1937	GCA_001663735.1_ASM166373v1_genomic.fna	Lactobacillus	Firmicutes
1938	GCA_002021985.1_ASM202198v1_genomic.fna	Campylobacter	Proteobacteria
1939	GCA_002021965.1_ASM202196v1_genomic.fna	Campylobacter	Proteobacteria
1940	GCA_001663715.1_ASM166371v1_genomic.fna	Lactobacillus	Firmicutes
1941	GCA_002287945.1_ASM228794v1_genomic.fna	Lactobacillus	Firmicutes
1942	GCA_002003365.1_ASM200336v1_genomic.fna	Clostridium	Firmicutes
1943	GCA_900187015.1_50465_F01_genomic.fna	Serratia	Proteobacteria
1944	GCA_000319475.1_ASM31947v1_genomic.fna	Bacillus	Firmicutes
1945	GCA_001188915.1_ASM118891v1_genomic.fna	Staphylococcus	Firmicutes
1946	GCA_002393225.1_ASM239322v1_genomic.fna	Bacillus	Firmicutes
1947	GCA_001886675.1_ASM188667v1_genomic.fna	Enterococcus	Firmicutes
1948	GCA_000092345.1_ASM9234v1_genomic.fna	Clostridium	Firmicutes
1949	GCA_001938705.1_ASM193870v1_genomic.fna	Bacillus	Firmicutes
1950	GCA_001999905.1_ASM199990v1_genomic.fna	Planococcus	Firmicutes
1951	GCA_001634285.1_ASM163428v1_genomic.fna	Anoxybacillus	Firmicutes
1952	GCA_001042545.2_ASM104254v2_genomic.fna	Francisella	Proteobacteria
1953	GCA_000438825.1_ASM43882v1_genomic.fna	Serratia	Proteobacteria
1954	GCA_001617605.1_ASM161760v1_genomic.fna	Rummeliibacillus	Firmicutes
1955	GCA_001642085.1_ASM164208v1_genomic.fna	Streptococcus	Firmicutes
1956	GCA_001663675.1_ASM166367v1_genomic.fna	Lactobacillus	Firmicutes
1957	GCA_001953135.1_ASM195313v1_genomic.fna	Lactobacillus	Firmicutes
1958	GCA_000195535.1_ASM19553v1_genomic.fna	Francisella	Proteobacteria
1959	GCA_000497485.1_ASM49748v1_genomic.fna	Bacillus	Firmicutes
1960	GCA_001908475.1_ASM190847v1_genomic.fna	Bacillus	Firmicutes
1961	GCA_001663795.1_ASM166379v1_genomic.fna	Streptococcus	Firmicutes
1962	GCA_000341665.1_ASM34166v1_genomic.fna	Bacillus	Firmicutes
1963	GCA_000830095.1_ASM83009v1_genomic.fna	Bacillus	Firmicutes
1964	GCA_002236895.1_ASM223689v1_genomic.fna	Bacillus	Firmicutes
1965	GCA_000233575.1_ASM23357v1_genomic.fna	Streptococcus	Firmicutes
1966	GCA_000807175.1_ASM80717v1_genomic.fna	Clostridium	Firmicutes
1967	GCA_002078255.1_ASM207825v1_genomic.fna	Staphylococcus	Firmicutes
1968	GCA_002357135.1_ASM235713v1_genomic.fna	Rickettsia	Proteobacteria
1969	GCA_002220795.1_ASM222079v1_genomic.fna	Campylobacter	Proteobacteria

1970	GCA_000831105.1_ASM83110v1_genomic.fna	Streptococcus	Firmicutes
1971	GCA_002211625.1_ASM221162v1_genomic.fna	Fusobacterium	Fusobacteria
1972	GCA_000013545.1_ASM1354v1_genomic.fna	Streptococcus	Firmicutes
1973	GCA_000146975.1_ASM14697v1_genomic.fna	Streptococcus	Firmicutes
1974	GCA_001723545.1_ASM172354v1_genomic.fna	Lactobacillus	Firmicutes
1975	GCA_001611115.1_ASM161111v1_genomic.fna	Pediococcus	Firmicutes
1976	GCA_001700895.1_ASM170089v1_genomic.fna	Buchnera	Proteobacteria
1977	GCA_000013465.1_ASM1346v1_genomic.fna	Staphylococcus	Firmicutes
1978	GCA_001951095.1_ASM195109v1_genomic.fna	Weissella	Firmicutes
1979	GCA_001682435.2_ASM168243v2_genomic.fna	Staphylococcus	Firmicutes
1980	GCA_000018065.1_ASM1806v1_genomic.fna	Prochlorococcus	Cyanobacteria
1981	GCA_000195435.4_ASM19543v4_genomic.fna	Listeria	Firmicutes
1982	GCA_000479375.2_ASM47937v2_genomic.fna	Lactococcus	Firmicutes
1983	GCA_001017595.1_ASM101759v1_genomic.fna	Mycoplasma	Tenericutes
1984	GCA_000283675.1_ASM28367v1_genomic.fna	Bacillus	Firmicutes
1985	GCA_000761115.1_ASM76111v1_genomic.fna	Lactococcus	Firmicutes
1986	GCA_000197755.2_ASM19775v2_genomic.fna	Listeria	Firmicutes
1987	GCA_001558815.1_ASM155881v1_genomic.fna	Staphylococcus	Firmicutes
1988	GCA_000831165.1_ASM83116v1_genomic.fna	Streptococcus	Firmicutes
1989	GCA_000270145.1_ASM27014v1_genomic.fna	Streptococcus	Firmicutes
1990	GCA_000304375.1_AINO_genomic.fna	Campylobacter	Proteobacteria
1991	GCA_000430385.1_ASM43038v1_genomic.fna	Campylobacter	Proteobacteria
1992	GCA_000016805.1_ASM1680v1_genomic.fna	Staphylococcus	Firmicutes
1993	GCA_000292415.1_ASM29241v1_genomic.fna	Bacillus	Firmicutes
1994	GCA_000147095.1_ASM14709v1_genomic.fna	Streptococcus	Firmicutes
1995	GCA_000018125.1_ASM1812v1_genomic.fna	Streptococcus	Firmicutes
1996	GCA_001723625.1_ASM172362v1_genomic.fna	Erysipelothrix	Firmicutes
1997	GCA_002243495.1_ASM224349v1_genomic.fna	Bacillus	Firmicutes
1998	GCA_001623565.1_ASM162356v1_genomic.fna	Streptococcus	Firmicutes
1999	GCA_001598035.1_ASM159803v1_genomic.fna	Streptococcus	Firmicutes
2000	GCA_002173595.1_ASM217359v1_genomic.fna	Pediococcus	Firmicutes
2001	GCA_001687605.2_ASM168760v2_genomic.fna	Planococcus	Firmicutes
2002	GCA_900128595.1_BCipseudotaxifoliae_v1.0_genomic.fna	Buchnera	Proteobacteria
2003	GCA_002504325.1_ASM250432v1_genomic.fna	Bacillus	Firmicutes

2004	GCA_001717505.1_ASM171750v1_genomic.fna	Dialister	Firmicutes
2005	GCA_001715535.1_ASM171553v1_genomic.fna	Spiroplasma	Tenericutes
2006	GCA_000238975.1_ASM23897v1_genomic.fna	Serratia	Proteobacteria
2007	GCA_002073355.1_ASM207335v1_genomic.fna	Streptococcus	Firmicutes
2008	GCA_000241125.1_ASM24112v1_genomic.fna	Mycoplasma	Tenericutes
2009	GCA_000582665.1_ASM58266v1_genomic.fna	Lactobacillus	Firmicutes
2010	GCA_000831125.1_ASM83112v1_genomic.fna	Streptococcus	Firmicutes
2011	GCA_000832425.1_ASM83242v1_genomic.fna	Bacillus	Firmicutes
2012	GCA_000294495.1_ASM29449v1_genomic.fna	Streptococcus	Firmicutes
2013	GCA_000688795.1_ASM68879v1_genomic.fna	Bacillus	Firmicutes
2014	GCA_000195555.1_ASM19555v1_genomic.fna	Francisella	Proteobacteria
2015	GCA_001629735.1_ASM162973v1_genomic.fna	Lysinibacillus	Firmicutes
2016	GCA_001746555.1_ASM174655v1_genomic.fna	Clostridium	Firmicutes
2017	GCA_000498675.1_ASM49867v1_genomic.fna	Lactobacillus	Firmicutes
2018	GCA_001735765.1_ASM173576v1_genomic.fna	Clostridium	Firmicutes
2019	GCA_002370355.1_ASM237035v1_genomic.fna	Lactobacillus	Firmicutes
2020	GCA_001721685.1_ASM172168v1_genomic.fna	Bacillus	Firmicutes
2021	GCA_900187115.1_51699_F01_genomic.fna	Arcobacter	Proteobacteria
2022	GCA_001719185.1_ASM171918v1_genomic.fna	Bacillus	Firmicutes
2023	GCA_001685375.1_ASM168537v1_genomic.fna	Streptococcus	Firmicutes
2024	GCA_900186955.1_49386_E02_genomic.fna	Bacillus	Firmicutes
2025	GCA_001558375.1_ASM155837v1_genomic.fna	Staphylococcus	Firmicutes
2026	GCA_000876545.1_ASM87654v1_genomic.fna	Bacillus	Firmicutes
2027	GCA_001854185.1_ASM185418v1_genomic.fna	Clostridium	Firmicutes
2028	GCA_001708305.1_ASM170830v1_genomic.fna	Streptococcus	Firmicutes
2029	GCA_002000885.1_ASM200088v1_genomic.fna	Lactobacillus	Firmicutes
2030	GCA_000696015.1_ASM69601v1_genomic.fna	Mycoplasma	Tenericutes
2031	GCA_000816385.2_ASM81638v2_genomic.fna	Campylobacter	Proteobacteria
2032	GCA_000767745.1_ASM76774v1_genomic.fna	Clostridium	Firmicutes
2033	GCA_001011095.1_ASM101109v1_genomic.fna	Lactobacillus	Firmicutes
2034	GCA_001877035.1_ASM187703v1_genomic.fna	Clostridium	Firmicutes
2035	GCA_001870205.1_ASM187020v1_genomic.fna	Vagococcus	Firmicutes
2036	GCA_002009235.1_ASM200923v1_genomic.fna	Planococcus	Firmicutes
2037	GCA_000763495.1_ASM76349v1_genomic.fna	Listeria	Firmicutes

2038	GCA_900187245.1_51470_D02_genomic.fna	Streptococcus	Firmicutes
2039	GCA_002173535.1_ASM217353v1_genomic.fna	Pediococcus	Firmicutes
2040	GCA_001945665.1_ASM194566v1_genomic.fna	Borrelia	Spirochaetes
2041	GCA_002117225.1_ASM211722v1_genomic.fna	Lactobacillus	Firmicutes
2042	GCA_001932615.1_ASM193261v1_genomic.fna	Weissella	Firmicutes
2043	GCA_000828775.1_ASM82877v1_genomic.fna	Serratia	Proteobacteria
2044	GCA_000211545.6_ASM21154v6_genomic.fna	Mycoplasma	Tenericutes
2045	GCA_002504385.1_ASM250438v1_genomic.fna	Mesoplasma	Tenericutes
2046	GCA_001900245.1_ASM190024v1_genomic.fna	Mycoplasma	Tenericutes
2047	GCA_000583755.1_ASM58375v1_genomic.fna	Campylobacter	Proteobacteria
2048	GCA_900120165.1_PRJEB15652_genomic.fna	Ezakiella	Firmicutes
2049	GCA_002224305.1_ASM222430v1_genomic.fna	Lactobacillus	Firmicutes
2050	GCA_001889165.1_ASM188916v1_genomic.fna	Bacillus	Firmicutes
2051	GCA_002213945.1_ASM221394v1_genomic.fna	Listeria	Firmicutes
2052	GCA_002000625.1_ASM200062v1_genomic.fna	Staphylococcus	Firmicutes
2053	GCA_002407125.1_ASM240712v1_genomic.fna	Campylobacter	Proteobacteria
2054	GCA_002238375.1_ASM223837v1_genomic.fna	Campylobacter	Proteobacteria
2055	GCA_002000645.1_ASM200064v1_genomic.fna	Staphylococcus	Firmicutes
2056	GCA_002213985.1_ASM221398v1_genomic.fna	Listeria	Firmicutes
2057	GCA_002213965.1_ASM221396v1_genomic.fna	Listeria	Firmicutes
2058	GCA_002000665.1_ASM200066v1_genomic.fna	Staphylococcus	Firmicutes
2059	GCA_002214005.1_ASM221400v1_genomic.fna	Listeria	Firmicutes
2060	GCA_002587105.1_ASM258710v1_genomic.fna	Campylobacter	Proteobacteria
2061	GCA_002214045.1_ASM221404v1_genomic.fna	Listeria	Firmicutes
2062	GCA_002000685.1_ASM200068v1_genomic.fna	Staphylococcus	Firmicutes
2063	GCA_002587225.1_ASM258722v1_genomic.fna	Campylobacter	Proteobacteria
2064	GCA_002214085.1_ASM221408v1_genomic.fna	Listeria	Firmicutes
2065	GCA_002000725.1_ASM200072v1_genomic.fna	Staphylococcus	Firmicutes
2066	GCA_002000705.1_ASM200070v1_genomic.fna	Staphylococcus	Firmicutes
2067	GCA_002214065.1_ASM221406v1_genomic.fna	Listeria	Firmicutes
2068	GCA_002000745.1_ASM200074v1_genomic.fna	Staphylococcus	Firmicutes
2069	GCA_002000765.1_ASM200076v1_genomic.fna	Staphylococcus	Firmicutes
2070	GCA_002214125.1_ASM221412v1_genomic.fna	Listeria	Firmicutes
2071	GCA_002214105.1_ASM221410v1_genomic.fna	Listeria	Firmicutes

2072	GCA_002000785.1_ASM200078v1_genomic.fna	Staphylococcus	Firmicutes
2073	GCA_002214145.1_ASM221414v1_genomic.fna	Listeria	Firmicutes
2074	GCA_002000805.1_ASM200080v1_genomic.fna	Staphylococcus	Firmicutes
2075	GCA_002285655.1_ASM228565v1_genomic.fna	Listeria	Firmicutes
2076	GCA_002000825.1_ASM200082v1_genomic.fna	Staphylococcus	Firmicutes
2077	GCA_002220325.1_ASM222032v1_genomic.fna	Listeria	Firmicutes
2078	GCA_002557735.1_ASM255773v1_genomic.fna	Listeria	Firmicutes
2079	GCA_002285835.1_ASM228583v1_genomic.fna	Listeria	Firmicutes
2080	GCA_002000845.1_ASM200084v1_genomic.fna	Staphylococcus	Firmicutes
2081	GCA_000253195.1_ASM25319v1_genomic.fna	Clostridium	Firmicutes
2082	GCA_001989555.1_ASM198955v1_genomic.fna	Enterococcus	Firmicutes
2083	GCA_001586105.1_ASM158610v1_genomic.fna	Bacillus	Firmicutes
2084	GCA_000523045.1_ASM52304v1_genomic.fna	Bacillus	Firmicutes
2085	GCA_002000865.1_ASM200086v1_genomic.fna	Staphylococcus	Firmicutes
2086	GCA_002025125.1_ASM202512v1_genomic.fna	Staphylococcus	Firmicutes
2087	GCA_002557815.1_ASM255781v1_genomic.fna	Listeria	Firmicutes
2088	GCA_900070175.1_Lactobacillus_rhamnosus_CECT_8800_genomic.fna	Lactobacillus	Firmicutes
2089	GCA_001721905.1_ASM172190v1_genomic.fna	Enterococcus	Firmicutes
2090	GCA_002220575.1_ASM222057v1_genomic.fna	Serratia	Proteobacteria
2091	GCA_001865495.1_ASM186549v1_genomic.fna	Campylobacter	Proteobacteria
2092	GCA_001272795.1_ASM127279v1_genomic.fna	Mycoplasma	Tenericutes
2093	GCA_001484005.1_ASM148400v1_genomic.fna	Lactobacillus	Firmicutes
2094	GCA_001047695.1_ASM104769v1_genomic.fna	Leuconostoc	Firmicutes
2095	GCA_000988345.1_ASM98834v1_genomic.fna	Bacillus	Firmicutes
2096	GCA_000338115.2_ASM33811v2_genomic.fna	Lactobacillus	Firmicutes
2097	GCA_000319655.2_ASM31965v2_genomic.fna	Mycoplasma	Tenericutes
2098	GCA_000987825.1_ASM98782v1_genomic.fna	Bacillus	Firmicutes
2099	GCA_002220595.1_ASM222059v1_genomic.fna	Serratia	Proteobacteria
2100	GCA_001865515.1_ASM186551v1_genomic.fna	Campylobacter	Proteobacteria
2101	GCA_002073435.1_ASM207343v1_genomic.fna	Streptococcus	Firmicutes
2102	GCA_001296205.1_ASM129620v1_genomic.fna	Streptococcus	Firmicutes
2103	GCA_000025825.1_ASM2582v1_genomic.fna	Bacillus	Firmicutes
2104	GCA_000219765.1_ASM21976v1_genomic.fna	Streptococcus	Firmicutes
2105	GCA_000233755.1_ASM23375v1_genomic.fna	Lactobacillus	Firmicutes

2106	GCA_000833255.1_ASM83325v1_genomic.fna	Francisella	Proteobacteria
2107	GCA_001581895.1_ASM158189v1_genomic.fna	Lactobacillus	Firmicutes
2108	GCA_001272815.1_ASM127281v1_genomic.fna	Mycoplasma	Tenericutes
2109	GCA_000307125.1_ASM30712v1_genomic.fna	Clostridium	Firmicutes
2110	GCA_000307185.1_ASM30718v1_genomic.fna	Streptococcus	Firmicutes
2111	GCA_000691545.1_ASM69154v1_genomic.fna	Borrelia	Spirochaetes
2112	GCA_001998885.1_ASM199888v1_genomic.fna	Vagococcus	Firmicutes
2113	GCA_001998805.1_ASM199880v1_genomic.fna	Leuconostoc	Firmicutes
2114	GCA_000221645.1_ASM22164v1_genomic.fna	Bacillus	Firmicutes
2115	GCA_001545095.1_ASM154509v1_genomic.fna	Erysipelothrix	Firmicutes
2116	GCA_000284575.1_ASM28457v1_genomic.fna	Streptococcus	Firmicutes
2117	GCA_000444405.1_ASM44440v1_genomic.fna	Enterococcus	Firmicutes
2118	GCA_001989575.1_ASM198957v1_genomic.fna	Auricoccus	Firmicutes
2119	GCA_000832885.1_ASM83288v1_genomic.fna	Bacillus	Firmicutes
2120	GCA_001750885.1_ASM175088v1_genomic.fna	Enterococcus	Firmicutes
2121	GCA_001593765.1_ASM159376v1_genomic.fna	Bacillus	Firmicutes
2122	GCA_002163735.1_ASM216373v1_genomic.fna	Enterococcus	Firmicutes
2123	GCA_000717535.1_ASM71753v1_genomic.fna	Bacillus	Firmicutes
2124	GCA_000204565.1_ASM20456v1_genomic.fna	Clostridium	Firmicutes
2125	GCA_000277185.1_ASM27718v1_genomic.fna	Rickettsia	Proteobacteria
2126	GCA_002021925.1_ASM202192v1_genomic.fna	Campylobacter	Proteobacteria
2127	GCA_900128415.1_PRJEB15655_genomic.fna	Anaerococcus	Firmicutes
2128	GCA_000166655.2_ASM16665v1_genomic.fna	Borrelia	Spirochaetes
2129	GCA_000211255.2_ASM21125v2_genomic.fna	Enterococcus	Firmicutes
2130	GCA_000699465.1_ASM69946v1_genomic.fna	Bacillus	Firmicutes
2131	GCA_002142575.1_ASM214257v1_genomic.fna	Lactobacillus	Firmicutes
2132	GCA_000248415.1_ASM24841v1_genomic.fna	Francisella	Proteobacteria
2133	GCA_000344765.1_ASM34476v1_genomic.fna	Streptococcus	Firmicutes
2134	GCA_000189515.1_ASM18951v1_genomic.fna	Lactobacillus	Firmicutes
2135	GCA_000283795.1_ASM28379v1_genomic.fna	Rickettsia	Proteobacteria
2136	GCA_000962775.1_ASM96277v1_genomic.fna	Borrelia	Spirochaetes
2137	GCA_000466785.3_ASM46678v3_genomic.fna	Lactobacillus	Firmicutes
2138	GCA_000143865.1_ASM14386v1_genomic.fna	Mycoplasma	Tenericutes
2139	GCA_001705585.1_ASM170558v1_genomic.fna	Streptococcus	Firmicutes

2140	GCA_000191165.1_ASM19116v1_genomic.fna	Lactobacillus	Firmicutes
2141	GCA_000952915.1_9231-Abomsa_assembly_V8_genomic.fna	Mycoplasma	Tenericutes
2142	GCA_002101335.1_ASM210133v1_genomic.fna	Staphylococcus	Firmicutes
2143	GCA_000512745.2_ASM51274v2_genomic.fna	Campylobacter	Proteobacteria
2144	GCA_000932055.2_ASM93205v2_genomic.fna	Clostridioides	Firmicutes
2145	GCA_000019865.1_ASM1986v1_genomic.fna	Coxiella	Proteobacteria
2146	GCA_000194785.1_ASM19478v1_genomic.fna	Lactobacillus	Firmicutes
2147	GCA_000348805.1_ASM34880v1_genomic.fna	Blattabacterium	Bacteroidetes
2148	GCA_000209795.2_ASM20979v2_genomic.fna	Bacillus	Firmicutes
2149	GCA_000832445.1_ASM83244v1_genomic.fna	Bacillus	Firmicutes
2150	GCA_000831145.1_ASM83114v1_genomic.fna	Streptococcus	Firmicutes
2151	GCA_000180515.2_ASM18051v2_genomic.fna	Streptococcus	Firmicutes
2152	GCA_000230295.1_ASM23029v1_genomic.fna	Streptococcus	Firmicutes
2153	GCA_002119805.1_ASM211980v1_genomic.fna	Macrococcus	Firmicutes
2154	GCA_000092165.1_ASM9216v1_genomic.fna	Bacillus	Firmicutes
2155	GCA_000305335.2_ASM30533v2_genomic.fna	Streptococcus	Firmicutes
2156	GCA_000011405.1_ASM1140v1_genomic.fna	Francisella	Proteobacteria
2157	GCA_000014485.1_ASM1448v1_genomic.fna	Streptococcus	Firmicutes
2158	GCA_002163565.1_ASM216356v1_genomic.fna	Spiroplasma	Tenericutes
2159	GCA_000236455.2_ASM23645v2_genomic.fna	Lactobacillus	Firmicutes
2160	GCA_000972685.1_ASM97268v1_genomic.fna	Bacillus	Firmicutes
2161	GCA_000292485.1_ASM29248v1_genomic.fna	Mycoplasma	Tenericutes
2162	GCA_002211785.1_ASM221178v1_genomic.fna	Francisella	Proteobacteria
2163	GCA_001726125.1_ASM172612v1_genomic.fna	Bacillus	Firmicutes
2164	GCA_002224365.1_ASM222436v1_genomic.fna	Virgibacillus	Firmicutes
2165	GCA_002216855.1_ASM221685v1_genomic.fna	Paraphotobacteriur	Proteobacteria
2166	GCA_002237575.1_ASM223757v1_genomic.fna	Spiroplasma	Tenericutes
2167	GCA_900187005.1_50569_B01_genomic.fna	Staphylococcus	Firmicutes
2168	GCA_900187225.1_51881_G01_genomic.fna	Listeria	Firmicutes
2169	GCA_002025145.1_ASM202514v1_genomic.fna	Staphylococcus	Firmicutes
2170	GCA_002088995.1_ASM208899v1_genomic.fna	Staphylococcus	Firmicutes
2171	GCA_002085525.1_ASM208552v1_genomic.fna	Staphylococcus	Firmicutes
2172	GCA_002089055.1_ASM208905v1_genomic.fna	Staphylococcus	Firmicutes
2173	GCA_002089035.1_ASM208903v1_genomic.fna	Staphylococcus	Firmicutes

2174	GCA_002089095.1_ASM208909v1_genomic.fna	Staphylococcus	Firmicutes
2175	GCA_002089115.1_ASM208911v1_genomic.fna	Staphylococcus	Firmicutes
2176	GCA_002089075.1_ASM208907v1_genomic.fna	Staphylococcus	Firmicutes
2177	GCA_002097595.1_ASM209759v1_genomic.fna	Staphylococcus	Firmicutes
2178	GCA_002140115.1_ASM214011v1_genomic.fna	Staphylococcus	Firmicutes
2179	GCA_002202095.1_ASM220209v1_genomic.fna	Staphylococcus	Firmicutes
2180	GCA_002204555.1_ASM220455v1_genomic.fna	Staphylococcus	Firmicutes
2181	GCA_002202075.1_ASM220207v1_genomic.fna	Staphylococcus	Firmicutes
2182	GCA_002209325.1_ASM220932v1_genomic.fna	Staphylococcus	Firmicutes
2183	GCA_002214665.1_ASM221466v1_genomic.fna	Staphylococcus	Firmicutes
2184	GCA_002204575.1_ASM220457v1_genomic.fna	Staphylococcus	Firmicutes
2185	GCA_002310395.1_ASM231039v1_genomic.fna	Staphylococcus	Firmicutes
2186	GCA_002310435.1_ASM231043v1_genomic.fna	Staphylococcus	Firmicutes
2187	GCA_001596535.1_ASM159653v1_genomic.fna	Bacillus	Firmicutes
2188	GCA_002356675.1_ASM235667v1_genomic.fna	Staphylococcus	Firmicutes
2189	GCA_002355355.1_ASM235535v1_genomic.fna	Staphylococcus	Firmicutes
2190	GCA_000209755.1_ASM20975v1_genomic.fna	Listeria	Firmicutes
2191	GCA_900128735.1_BCiconfinis_v1.0_genomic.fna	Buchnera	Proteobacteria
2192	GCA_000972725.1_ASM97272v1_genomic.fna	Staphylococcus	Firmicutes
2193	GCA_002386245.1_ASM238624v1_genomic.fna	Staphylococcus	Firmicutes
2194	GCA_002442975.1_ASM244297v1_genomic.fna	Staphylococcus	Firmicutes
2195	GCA_000466065.2_ASM46606v2_genomic.fna	Campylobacter	Proteobacteria
2196	GCA_000466075.2_ASM46607v2_genomic.fna	Campylobacter	Proteobacteria
2197	GCA_000807375.1_ASM80737v1_genomic.fna	Lactococcus	Firmicutes
2198	GCA_000635895.2_ASM63589v2_genomic.fna	Bacillus	Firmicutes
2199	GCA_000017085.1_ASM1708v1_genomic.fna	Staphylococcus	Firmicutes
2200	GCA_000017125.1_ASM1712v1_genomic.fna	Staphylococcus	Firmicutes
2201	GCA_000789315.1_ASM78931v1_genomic.fna	Bacillus	Firmicutes
2202	GCA_002078375.1_ASM207837v1_genomic.fna	Lactococcus	Firmicutes
2203	GCA_000210815.2_ASM21081v2_genomic.fna	Listeria	Firmicutes
2204	GCA_000832465.1_ASM83246v1_genomic.fna	Bacillus	Firmicutes
2205	GCA_000183185.1_ASM18318v1_genomic.fna	Mycoplasma	Tenericutes
2206	GCA_000390245.1_ASM39024v1_genomic.fna	Streptococcus	Firmicutes
2207	GCA_000091905.1_ASM9190v1_genomic.fna	Streptococcus	Firmicutes

2208	GCA_000299135.1_ASM29913v1_genomic.fna	Streptococcus	Firmicutes
2209	GCA_900128725.1_BCifornacula_v1.0_genomic.fna	Buchnera	Proteobacteria
2210	GCA_000021445.1_ASM2144v1_genomic.fna	Bacillus	Firmicutes
2211	GCA_000250905.1_ASM25090v1_genomic.fna	Streptococcus	Firmicutes
2212	GCA_000196595.1_ASM19659v1_genomic.fna	Streptococcus	Firmicutes
2213	GCA_001026925.1_ASM102692v1_genomic.fna	Streptococcus	Firmicutes
2214	GCA_000747545.1_ASM74754v1_genomic.fna	Bacillus	Firmicutes
2215	GCA_000009385.1_ASM938v1_genomic.fna	Streptococcus	Firmicutes
2216	GCA_000018965.1_ASM1896v1_genomic.fna	Streptococcus	Firmicutes
2217	GCA_000163915.2_ASM16391v2_genomic.fna	Fusobacterium	Fusobacteria
2218	GCA_001855705.1_ASM185570v1_genomic.fna	Streptococcus	Firmicutes
2219	GCA_000521565.1_ASM52156v1_genomic.fna	Buchnera	Proteobacteria
2220	GCA_000789355.1_ASM78935v1_genomic.fna	Clostridium	Firmicutes
2221	GCA_000012465.1_ASM1246v1_genomic.fna	Prochlorococcus	Cyanobacteria
2222	GCA_000248435.1_ASM24843v1_genomic.fna	Francisella	Proteobacteria
2223	GCA_002278095.1_ASM227809v1_genomic.fna	Lactobacillus	Firmicutes
2224	GCA_000013065.1_ASM1306v1_genomic.fna	Bacillus	Firmicutes
2225	GCA_000022925.1_ASM2292v1_genomic.fna	Listeria	Firmicutes
2226	GCA_001304735.1_ASM130473v1_genomic.fna	Clostridium	Firmicutes
2227	GCA_000699525.1_ASM69952v1_genomic.fna	Bacillus	Firmicutes
